# Elucidating the Influence of Serum Concentration, Sex, and Particle Size on Iron Oxide Nanoparticle–Lipid Biocorona Formation

**DOI:** 10.3390/nano16110683

**Published:** 2026-06-01

**Authors:** Jenna N. Swihart, Christina R. Ferreira, Akshada Shinde, Jonathan H. Shannahan

**Affiliations:** 1 School of Health Sciences, Purdue University, 550 W Stadium Ave., West Lafayette, IN 47907, USA; 2 Purdue Metabolite Profiling Facility, Purdue University, West Lafayette, IN 47907, USA

**Keywords:** lipid corona, lipidomics, corona formation, serum concentration, particle size, sex

## Abstract

Biocorona (BC) formation is a critical determinant of nanoparticle (NP) biological identity and downstream interactions, yet lipid association within BCs remains comparatively understudied relative to proteins, despite its potential relevance to NP stability, biodistribution, cellular interactions, and clearance. A more complete understanding of NP–lipid interactions is essential for optimizing NP-based therapies and supporting their safe clinical translation. In this study, we evaluated how serum concentration, biological sex, and NP size influence lipid association with iron oxide (Fe_3_O_4_) NP BCs. Lipids associated with 50 or 100 nm Fe_3_O_4_ NPs were characterized following incubation in male or female human serum across increasing serum concentrations of 5%, 10%, 25%, 50%, or 75% (*v*/*v*). Increasing serum concentration promoted greater lipid association and increased BC complexity, with higher serum conditions yielding more compositionally diverse lipid coronas. BCs formed on 50 nm Fe_3_O_4_ NPs consistently contained more lipid species than those formed on 100 nm Fe_3_O_4_ NPs, indicating pronounced size-dependent differences in lipid recruitment. BCs formed in male serum also contained more lipid species and a greater number of unique lipids than corresponding female BCs, demonstrating that biological sex significantly influenced both lipid composition and abundance within the BC. Rank-based comparisons further indicated that lipid association was governed not only by serum abundance but also by selective binding behaviors. Together, these findings demonstrate that lipid corona formation is strongly shaped by both the biofluid environment and NP design variables, emphasizing the importance of considering lipid coronas in NP design and evaluation, particularly for applications in drug delivery, nanomedicine, and precision diagnostics.

## 1. Introduction

Engineered nanoparticles (NPs) are increasingly used in biomedical applications, including drug delivery, imaging, and diagnostics due to their modifiable physicochemical properties and capacity for targeted interactions with biological systems [1,2,3,4]. Among these, iron oxide (Fe_3_O_4_) NPs have been extensively utilized because of their favorable magnetic properties, relatively high biocompatibility, and capacity for versatile surface modification. Fe_3_O_4_ NPs are often applied in magnetic imaging modalities, anemia treatments, gene therapies, vaccine development, and drug delivery systems [5,6,7,8,9]. The number of NP-based therapies also continues to grow. Between 1989 and 2020, more than 30 NP therapeutics and diagnostics received clinical approval from regulatory agencies, including the FDA and EMA [10]. By 2021, over 30 new clinical trials were evaluating previously approved NPs, while more than 35 emerging NP technologies were associated with over 55 new clinical trials [10]. These efforts include NP-enabled therapeutics for fibrosis, bacterial infections, immune disorders, radiation injury, and transplantation, highlighting the rapidly expanding clinical relevance of NP-based technologies [10]. Among these, iron-based nanomaterials represented a notable portion of approved treatments and ongoing clinical studies for iron-deficiency and imaging [10]. Despite recent advances in NP-based therapies, a significant gap remains in our understanding of the initial molecular interactions occurring when these materials first enter the body. Upon exposure to different physiological environments, such as the circulatory system, biomolecules (proteins, lipids, etc.) rapidly adhere to the NP surface, forming a coating referred to as the biocorona (BC) [11,12]. This newly acquired surface alters key NP physicochemical properties, including hydrodynamic size and surface charge, thereby altering how host cells and tissues respond to the NP [11,13]. As a result, the BC can alter NP activity, influencing biodistribution, cellular uptake, immune recognition, and clearance [11,14]. By incorporating biomolecules capable of receptor engagement, BCs may improve NP targeting to specific cells or tissues and modulate immune responses, offering opportunities for therapeutic targeting, vaccine development, and immunotherapy [15,16]. The BC can also obscure the foreign surface of NPs, reducing immune recognition and cytotoxicity, preventing rapid clearance by the immune system, and extending circulation time to increase the therapeutic window [15,17]. Recent clinical success of lipid NP-based mRNA vaccines has further demonstrated the translational value of NP delivery systems and highlighted the importance of understanding how NP surfaces interact with biomolecules [18]. For example, LNP-based mRNA vaccines used against COVID-19 demonstrated the clinical utility of NP platforms for nucleic acid delivery, while LNP-based siRNA therapies such as patisiran illustrate how NP systems can enable delivery of therapeutics that otherwise face biological barriers including degradation, poor membrane permeability, and rapid clearance [18,19,20,21]. To effectively employ NP-based therapeutics such as these, it is necessary to fully understand the molecular interactions that occur when NPs first enter biological systems.

BC formation is governed by a complex interplay between NP properties, time, and biological environment. The adsorption of biomolecules, including lipids, onto NP surfaces is influenced by intrinsic particle properties such as shape, surface charge, hydrophobicity, and surface roughness [11,14]. In addition to NP properties, the biological environment itself has a substantial influence on BC composition. Variations in biofluid composition, including biomolecule concentration, sex, and underlying health status, can alter which molecules preferentially associate with NP surfaces. For example, our previous studies have demonstrated that individual biofluid differences associated with sex, age, exercise, and metabolic syndrome can significantly alter protein and lipid corona composition and downstream biological responses [13,22,23,24]. Together, findings from our work and prior studies indicate that both NP properties and biological factors, including NP dose, sex, and NP size, influence BC formation and subsequent NP interactions.

NP dose, often modeled by varying serum concentration during NP incubation while holding NP mass constant, has emerged as an important factor influencing BC formation as it alters the relative ratio of available biomolecules to NPs. In in vitro studies, this is often modeled by varying serum or plasma concentration during NP incubation, effectively modeling the NP to biofluid ratio. Previous studies have shown that lower serum concentrations may limit biomolecule availability, leading to a reduced concentration of bound proteins, whereas higher serum concentrations resulted in greater saturation of adhered proteins, a greater fraction of low molecular weight corona proteins, and reduced ζ potential [25,26]. These dose-dependent differences in protein corona formation were shown to influence NP-cell interactions, including the amount of NP internalization and the mechanisms of cellular uptake [25,26,27]. Together, this work suggests that NP dose, as reflected by biofluid concentration, can meaningfully shape BC properties and downstream biological responses. However, in vitro incubation conditions may not fully reflect the in vivo environment where NPs encounter high biomolecule concentrations, continuous exchange, and physiological transport that may alter corona composition and biological outcomes [28,29]. Accordingly, defining how NP serum ratios influence BC formation under controlled conditions is a needed step toward interpreting dose-dependent NP-biomolecule interactions and their potential biological consequences.

Biological sex is another source of variability that can shape BC formation because circulating biomolecules differ between males and females. Differences in sex hormones, lipid metabolism, and immune regulation contribute to sex-dependent variations in serum proteins and lipids, including differences in lipoprotein profiles and in the abundance of individual lipid classes, as reported across numerous lipidomic studies [30,31]. Since BCs reflect the biomolecules available in the surrounding biofluid, these sex-dependent differences can translate to differences in male and female-derived BCs. Consistent with this, multiple studies have reported that male and female serum and plasma can yield distinct BCs and downstream differences in cellular interactions [32,33,34,35]. Sex-dependent differences in nanomedicine pharmacokinetics and biodistribution have also been reported. For example, several types of NPs, including PEGylated liposomal doxorubicin, silver NPS, and liposomal topotecan, exhibit faster clearance in males compared to females [33,36,37]. Additionally, sex-specific in vivo biodistribution patterns have been observed, including preferential accumulation of NPs in the female reproductive system during ovulation, which has been shown to affect tissue exposure and therapeutic outcomes [33,38]. Sex-dependent differences in biofluid therefore have the potential to influence BC formation and subsequent biological interactions.

NP size is a key variable that can shape BC formation because it directly alters surface area, curvature, and how biomolecules interact with the NP surface. Several studies have shown that NPs of different sizes, even when composed of identical materials and exposed to the same biological fluid, can develop distinct BCs that differ in both overall composition and relative protein and lipid abundances, resulting in altered NP-cell interactions [39,40,41,42]. Because NP size is easily modifiable and can be precisely controlled during therapeutic development, understanding its influence on BC formation is particularly relevant for optimizing drug delivery systems.

Despite growing recognition that BCs are composed of diverse biomolecular classes, the majority of BC studies have focused primarily on protein adsorption. However, biological fluids, such as serum, also contain abundant lipid species that contribute to membrane structure, immune signaling, and inflammatory regulation. Circulating lipids are highly variable and are influenced by a number of factors such as biological sex, age, exercise, and disease state, which can alter the pool of lipids available to associate with NP surfaces, thereby modulating BC composition and patient response to NP-enabled treatments. Compared to proteins, lipid components of the BC remain poorly characterized, and how biological variability influences lipid association with NP surfaces remains understudied, limiting some biologically relevant information that is not captured through proteomic analyses alone.

In this study, we hypothesized that human serum concentration governs lipid corona formation and that this process is further modulated by NP size and biological sex. To evaluate this, Fe_3_O_4_ NPs with 50 nm or 100 nm diameters were incubated in pooled male or female human serum across varying concentrations. Following incubation, lipids were extracted from the BCs and profiled via multiple reaction monitoring to assess the effects of serum concentration, sex, and NP size on lipid corona formation. By defining how these variables shape BC formation, this work provides insight into the initial interactions between lipids and NP surfaces that influence NP behavior and may inform the design of more predictable and effective NP-based drug delivery systems.

## 2. Materials and Methods

### 2.1. Fe_3_O_4_NP Characterization

Fe_3_O_4_ nanoparticles (Fe_3_O_4_NPs) of 50 and 100 nm, coated in a matrix of crosslinked dextran, were purchased from CD Bioparticles (Shirley, NY, USA). To verify the manufacturer’s specifications, Fe_3_O_4_NPs were diluted to 25 μg/mL in deionized (DI) water and assessed for hydrodynamic size, polydispersion index, and ζ-potential via a Zeta-Sizer Nano (Malvern Pananalytical, Westborough, MA, USA) (n = 4).

### 2.2. Formation of Fe_3_O_4_ NP-BC

Pooled fully deidentified male and female human serum purchased from a commercial vendor (BioChemed Services, Winchester, VA, USA; male serum catalog# 751NS-MP; female serum catalog# 751NS-FP) was diluted with DI water to relative concentrations of 5%, 10%, 25%, 50%, or 75% (*v*/*v*). Then, 125 μL of Fe_3_O_4_NPs (50 nm or 100 nm; 1 mg/mL in DI water) were added to 375 μL of diluted serum (n = 5/group). This resulted in a final Fe_3_O_4_NP suspension at a concentration of 250 μg/mL for all samples. Mass-based normalization was selected because NP dosing in both experimental and clinical contexts is commonly defined by mass concentration, and this approach has been utilized in several other BC formation studies [13,22,23,24,40,43]. NP-only blanks consisted of 125 μL of 50 and 100 nm Fe_3_O_4_NPs (1 mg/mL) and 375 μL of DI water. All samples were incubated at 4 °C for 16 h under constant rotation to facilitate biocorona (BC) formation. Incubation at 4 °C was selected to minimize biomolecular degradation and preserve biomolecular interactions during BC formation, particularly for temperature-sensitive lipid species. This temperature is consistent with previously established NP-BC protocols and has been used to limit thermally induced protein unfolding during BC formation [13,22,23,24,40,44]. Following incubation, samples were centrifuged at 21,130× *g* for 20 min at 4 °C to pellet the NP-BCs. Supernatants were removed and pellets were washed three times with PBS to eliminate free or unassociated biomolecules and collect Fe_3_O_4_ NPs. Samples containing only Fe_3_O_4_ NPs went through the same isolation procedure. Our laboratory has previously established and optimized this protocol for NP-BC formation [13,22,23,24].

### 2.3. Lipidomic Assessment of the Fe_3_O_4_ NP-BC

The lipid components of the NP-BCs were assessed using multiple reaction monitoring (MRM) profiling to evaluate alterations due to sex, Fe_3_O_4_NP size, and serum concentration. This approach was designed to provide broad lipidomic coverage of NP-associated lipid species rather than absolute quantification of a predefined subset of lipids. Lipids were extracted from the Fe_3_O_4_NP surface via a modified Bligh-Dyer lipid extraction method. Briefly, serum-only controls for both male and female groups were prepared at each serum concentration (5%, 10%, 25%, 50%, or 75%) without NPs. Then, NP-only samples and NP-BCs were resuspended in 200 μL of DI water while 200 μL of serum controls were transferred to a 1.5 mL microcentrifuge tube. Each tube received 550 μL of methanol and 250 μL of chloroform, after which samples were vortexed and incubated at 4 °C for 15 min. Phase separation was induced by the addition of 250 μL DI water and 250 μL chloroform, followed by centrifugation at 5000× *g* for 10 min. Following centrifugation, 400 μL of the bottom organic phases were collected into separate tubes. Additionally, pooled samples were created by combining 80 μL from each of the five biological replicates within a group. Solvents were evaporated under vacuum, and dried lipid extracts were stored at −80 °C until analysis. Before mass spectrometric analysis, dried samples were reconstituted in 200 μL methanol and 10 μL chloroform. The extracts were then diluted at a 1:350 ratio (*v*/*v*) in a mixture of acetonitrile, methanol, and 300 mM ammonium acetate (3:6.65:0.35, *v*/*v*) containing EquiSPLASH™ LIPIDOMIX™ internal standards (Avanti Research, Alabaster, AL, USA; #330731) spiked into the solvent at a final concentration of 50 ng/mL for relative quantification. Prepared samples (8 μL) were introduced in a randomized order into the ESI source of an Agilent 6410 QQQ mass spectrometer (Agilent Technologies, Santa Clara, CA, USA) using a micro-autosampler (G1377A). The capillary pump operated at a flow rate of 20 μL/min and maintained a pressure of 150 bar. To prevent carryover, the system was flushed with pure methanol between samples. A quality control sample containing a defined lipid mixture was analyzed at regular intervals to monitor instrument stability through peak intensity and retention time. In addition, blank samples consisting of the injection solvent with internal standards were run to assess background signal. A pooled sample for each Fe_3_O_4_NP-BC and serum control group was produced by combining 80 μL from each sample for a group. Initial MRM profiling was then performed on pooled samples as a discovery phase. Transitions yielding zero intensity across all pooled, control, and blank samples were excluded from the final methods. Individual samples were then run using the refined MRM methods (complete list of ion transitions in Supplemental Table S1).

### 2.4. Lipidomic Data Analysis

Raw MRM data were processed using an in-house script, and lists containing all monitored ion transitions along with their corresponding intensity values were exported to Microsoft Excel. To minimize background interference, only transitions with signal intensity exceeding 25% of that observed in blank samples were retained for further statistical analysis. A lipid was considered present within a group if its intensity exceeded the 25% threshold in at least three of the five biological replicates. Lipids identified as present in each comparison (e.g., serum concentration, sex, and Fe_3_O_4_NP size) were visualized using Venn diagrams generated through the Venn webtool (https://bioinformatics.psb.ugent.be/webtools/Venn/, accessed on 26 August 2025), enabling the identification of unique and shared lipid features across serum concentrations, sexes, and NP sizes. To compare the relative abundance of individual lipids based on concentration, Pearson correlations were computed to evaluate the relationship between serum concentration and the abundance of lipid species. Differences in the relative abundance of individual lipids based on NP size were assessed by calculating fold changes relative to the corresponding 50 nm group within each sex. Similarly, to compare the relative abundance of individual lipids based on sex, fold changes were calculated by dividing the mean signal intensity of each sample by the corresponding mean signal intensity of the male group within each Fe_3_O_4_NP size. Statistical comparisons were evaluated using *t*-tests (*p* < 0.05), an approach consistent with prior discovery-based -omics studies [45,46,47,48]. Specifically, Pascovici et al. demonstrated that standard *t*-tests are appropriate for exploratory -omics datasets when multiple-comparison corrections are overly conservative due to statistically limiting factors, including limited replicates, ratio compression, effect size, and other dataset-specific constraints that may increase the risk of excluding true positive findings [49]. Relative lipid abundances were also ranked from most to least abundant based on mean group intensity values and compared to lipid abundances in serum-only controls to understand BC accumulation. These analytical approaches are consistent with those used in Xia et al., Kobos et al., and Shinde et al. and with prior exploratory -omics workflows [22,23,24]. The lipidomic dataset generated in this study was further utilized in a separate machine learning framework to predict lipid corona formation on Fe_3_O_4_ NPs and identify lipid physicochemical properties associated with adsorption behavior, as reported in Basu et al. [50].

### 2.5. Statistical Analysis

Data are presented as mean ± standard error of means (SEM) with 5 samples/group. For MRM-based lipid profiling, a single analytical run per sample was performed to determine lipid modifications. Correlation significance testing was used to determine whether the associations between serum concentration and BC lipid abundance were statistically significant [13]. Statistical comparisons between sexes and NP sizes were performed using unpaired, two-tailed *t*-tests [22,24]. All analyses and data visualizations were performed using GraphPad Prism 10 (GraphPad, San Diego, CA, USA) and *p* < 0.05 was considered to be statistically significant.

## 3. Results and Discussion

### 3.1. Fe_3_O_4_ NP Characterization

Fe_3_O_4_ NPs were characterized to confirm alignment with manufacturer specifications for both particle sizes. The Fe_3_O_4_ NPs with a manufacturer-reported diameter of 50 nm exhibited a hydrodynamic size of 56.01 ± 0.64 nm, polydispersion index of 0.11 ± 0.01, and a ζ-potential of −5.37 ± 0.25 mV. Similarly, Fe_3_O_4_ NPs with a manufacturer-reported diameter of 100 nm demonstrated a hydrodynamic size of 138.40 ± 10.27 nm, polydispersion index of 0.17 ± 0.02, and a ζ-potential of −4.55 ± 1.07 mV. All parameters are reported as mean ± standard deviation (n = 4). The measured ζ-potential values for both Fe_3_O_4_ NP sizes were low in magnitude, suggesting limited electrostatic stabilization by surface charge alone [51]. Although these values may indicate an increased potential for particle-particle interactions, the low polydispersion index values support relatively uniform particle dispersions. Similar low-magnitude ζ-potential values have also been reported for coated Fe_3_O_4_ NPs in previous studies [52]. Because surface charge influences NP interactions with surrounding molecules, the near-neutral charge observed here may promote biomolecule association at the NP surface and thereby influence BC formation [53,54].

### 3.2. Diversity of Lipid Species Associated with Fe_3_O_4_ NP-BCs

**Figure 1:**
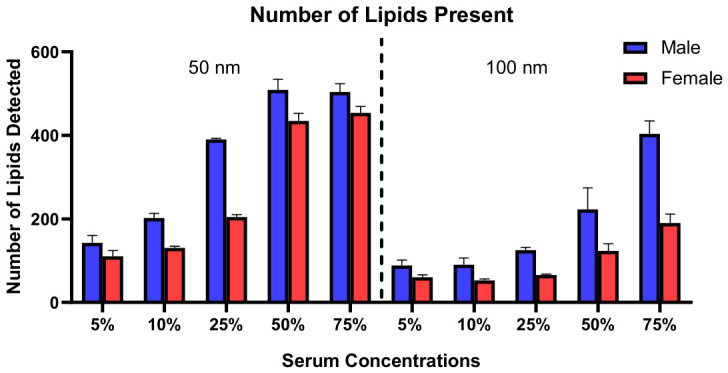
Lipid species diversity associated with Fe_3_O_4_ NP-BCs shown as the average number of individual lipid species detected in Fe_3_O_4_ NP-BCs following incubation of 50 nm or 100 nm Fe_3_O_4_ NPs in male or female serum across increasing serum concentrations (5%, 10%, 25%, 50%, and 75%). Data are presented as the average number of unique lipid species detected per experimental group ± SEM (n = 5/group).

To evaluate how serum concentration, biological sex, and NP size influence the diversity of lipid association with Fe_3_O_4_ NP-BCs, serum concentration was varied to model changes in the biofluid-to-NP ratio, with higher serum concentrations representing greater biomolecule availability per particle and therefore a lower relative NP dose. Thus, in the present study, references to relative NP dose refer to changes in the NP-to-biomolecule ratio rather than changes in the administered NP amount. The number of distinct lipid species detected on the NP surface was quantified and compared across experimental conditions (Figure 1). The average number of lipid species detected on Fe_3_O_4_ NP-BCs increased with increasing serum concentration across all particle sizes and sexes. For 50 nm Fe_3_O_4_ NPs incubated in male serum, an average of 119, 182, 379, 493, and 503 lipid species were detected following incubation in 5%, 10%, 25%, 50%, and 75% serum, respectively. 100 nm Fe_3_O_4_ NPs incubated in male serum exhibited fewer associated lipids across all concentrations, with averages of 67, 72, 113, 146, and 396 lipid species detected at the corresponding serum concentrations. A similar concentration-dependent increase in lipid diversity was observed following incubation in female serum. For 50 nm Fe_3_O_4_ NPs, an average of 92, 113, 191, 424, and 432 lipid species were identified in the BC at 5%, 10%, 25%, 50%, and 75% serum, respectively. 100 nm Fe_3_O_4_ NPs incubated in female serum demonstrated the lowest overall lipid association, with averages of 50, 46, 56, 107, and 179 lipid species detected in the BCs across increasing serum concentrations. This concentration-dependent increase in lipid diversity suggests that BC formation is strongly influenced by biofluid availability, as increasing serum concentrations provide a larger and more competitive pool of circulating biomolecules for adsorption. This trend is consistent with prior BC literature demonstrating that serum concentration is a key determinant of protein corona formation, as changes to the biofluid-to-NP ratio, effectively reflecting differences in relative NP dose, influence the abundance, composition, and overall mass of adsorbed species. At lower NP doses, greater biomolecule availability per particle may promote the formation of more compositionally complex and enriched coronas [25,26,55]. Increasing serum concentration also increases the biomolecule-to-NP surface area ratio, thereby increasing the availability of proteins, lipids, and lipoproteins capable of interacting with the NP surface [26]. As biomolecule availability increases, surface sites may become progressively occupied, while competitive exchange may favor enrichment of lipids with greater surface affinity [56]. Because serum lipids are largely transported within lipoprotein complexes, increased serum availability may also increase lipoprotein adsorption or transfer of lipoprotein-associated lipid species to the NP surface, contributing to the greater lipid diversity observed at higher serum concentrations [40,57].

Across all serum concentrations and NP sizes, BCs that were formed in male serum consistently contained a greater number of adsorbed unique lipid species compared to those formed in female serum. Sex-dependent differences in BC formation have been reported, with variations in plasma composition leading to distinct BC profiles and influencing downstream NP-cell interactions and treatment efficacy [32,35,58]. While these prior studies largely focused on protein coronas, our findings suggest that biological sex also strongly influences lipid association with NPs, highlighting an underexplored factor in BC formation. Differences in circulating lipid profiles, lipoprotein abundance, and associated carrier proteins between male and female serum are well documented and may contribute to the observed disparities in the diversity of lipids incorporated into the NP-BC [31,59].

Additionally, for both male and female conditions, 50 nm Fe_3_O_4_ NPs exhibited higher numbers of associated lipids than 100 nm Fe_3_O_4_ NPs at all serum concentrations. Prior studies demonstrate that NP size influences BC formation, affecting both the composition and abundance of adsorbed biomolecules [28,39,40,42]. Consistent with our findings, others have reported an inverse relationship between NP size and biomolecule adsorption, where smaller NPs exhibit more compositionally diverse binding. For example, studies summarized by Walkey et al. demonstrate increased protein adsorption to smaller NPs, an effect attributed to increased surface curvature and altered interaction energetics at the NP surface [42]. This inverse size-dependent trend aligns with the present findings, where smaller Fe_3_O_4_ NPs displayed greater lipid association, suggesting that increased curvature may enhance interactions with more diverse lipid species. However, size-dependent effects are not universally directional and can vary depending on NP composition and the surrounding biological environment. For example, prior work with polystyrene NPs demonstrated increased total cholesterol and triglyceride adsorption to larger particles compared to smaller NPs [40]. Taken together, these findings indicate that NP size is a critical determinant of lipid corona diversity, with the enhanced lipid species enrichment observed for 50 nm Fe_3_O_4_ NPs likely reflecting curvature-driven and system-specific interactions between the NP surface and lipid species. These size-dependent differences in lipid corona diversity may have important implications for nanomedicine design, as NP size is a readily tunable parameter during development and can be strategically modified to influence BC composition, stability, and downstream biological interactions [42,60,61,62,63,64,65].

### 3.3. Comparison of Lipid Components of the BC Across Serum Concentrations

**Figure 2:**
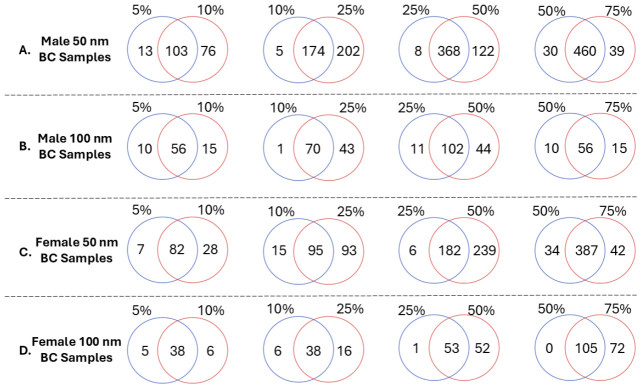
Comparison of lipid profiles between serum concentrations in (**A**) male 50 nm, (**B**) male 100 nm, (**C**) female 50 nm, and (**D**) female 100 nm BC samples. Lipids identified as present in one serum concentration were compared to lipids identified as present in the next highest serum concentration. Venn diagrams were used to illustrate the number of unique and common lipids. Present lipids were determined if intensities were at least 25% above the blank in at least three out of five samples in each group. A comprehensive list of the lipids identified in each group can be found in Tables S2–S5.

To evaluate how serum concentration influenced lipid composition of Fe_3_O_4_ NP-BCs, lipid species found to be present in each serum concentration were compared with those detected at the next highest concentration within each experimental group (Figure 2, complete list of lipids in Supplemental Tables S2–S5). Across all conditions, increasing serum concentration, reflecting decreasing NP dose, resulted in both the retention of previously detected lipid species and the addition of new lipids, indicating a progressive accumulation of lipid components on the NP surface. While each serum concentration contained lipids unique to that condition, the majority of lipid species were shared between concentrations, consistent with a building effect as serum concentration increased and relative NP dose decreased. Such behavior is consistent with established models of BC formation, where adsorption is governed by biomolecule abundance and competitive interactions, leading to retained and newly associated species across conditions [26,28,41].

For the 50 nm Fe_3_O_4_ NPs incubated in male serum, substantial overlap of BC lipids was observed, with 101 lipids shared across all serum concentrations, the majority of which belonged to the triglyceride class. There was a notable increase in lipid diversity with the transition from 10% to 25%. The 10% BCs contained only 5 unique lipid species, while 174 lipids were shared with the 25% condition, which contained 202 unique lipids (Figure 2A and Table S2). At higher serum concentrations, modeling lower NP doses, BC lipid profiles converged, with the 50% and 75% conditions sharing 460 lipid species and containing only 30 and 39 unique lipids, respectively, indicating a high degree of similarity at elevated serum concentrations (Figure 2A and Table S2). BCs formed in female serum displayed a comparable concentration-dependent trend but with a delayed transition toward increased lipid diversity compared to those formed in male serum. For 50 nm Fe_3_O_4_ NPs incubated in female serum, 58 lipids were shared across BCs from all serum concentrations, primarily triglycerides. The transition from 25% to 50% serum was associated with increased lipid diversity. The 25% serum BCs contained only 6 unique lipid species while 182 lipids were shared with the 50% condition, which contained 239 unique lipids (Figure 2C and Table S4). BC lipid profiles converged at higher serum concentrations, with the 50% and 75% conditions having 387 similar lipid species and only 34 and 42 unique lipids, respectively (Figure 2C and Table S4).

A similar pattern was observed for BCs formed on 100 nm Fe_3_O_4_ NPs in male serum, with 55 lipids shared across all concentrations, consisting predominantly of diglycerides and cholesteryl esters. The 10% BC samples contained only 1 unique lipid, TG(44:5)_C20:0, while 70 lipids were shared with the 25% condition, which contained 43 unique lipids (Figure 2B and Table S3). Like the 50 nm Fe_3_O_4_ particles, the two highest serum concentrations, simulating the two lowest NP doses, demonstrated substantial convergence, with the 50% and 75% serum BC samples sharing 56 lipid species and exhibiting only 10 and 15 unique lipids, respectively (Figure 2B and Table S3). A similar trend was observed for female BCs formed on 100 nm Fe_3_O_4_ NPs, which shared 32 lipid species detected across all concentrations, largely comprising diglycerides and cholesteryl esters. The 25% BCs contained a single unique lipid, PG(20:0), LPG(21:0), while 53 lipids were shared with the 50% condition, which contained 52 unique lipids (Figure 2D and Table S5). At higher serum concentrations, lipid profiles again converged, with all lipid species identified in the 50% BCs also present in the 75% BCs, and the 75% condition having 72 additional unique lipids (Figure 2D and Table S5).

Collectively, these findings indicate that serum concentration is a major driver of Fe_3_O_4_ NP-BC development, with all groups exhibiting a shift from a relatively limited lipid corona at lower serum concentrations toward a more compositionally complex and increasingly convergent BC at higher concentrations. Importantly, at a fixed NP concentration, increasing serum concentration also reflects a lower NP dose as each particle is exposed to a larger pool of serum components. In this context, higher serum conditions approximate lower NP dose conditions, where greater biomolecule availability favors formation of a more complexly developed BC.

Across both male and female samples, 50 nm Fe_3_O_4_ NP-BCs were dominated by triglycerides, whereas 100 nm Fe_3_O_4_ NP-BCs displayed enrichment of DGs and CEs. This pattern suggests the formation of relatively stable baseline BCs and may reflect adsorption of lipoprotein-associated lipids rather than free lipids. This aligns with prior lipidomic studies of NP coronas reporting enrichment of TGs, DGs, CEs, and other neutral lipids, as well as evidence that lipid corona composition can arise through lipoprotein-mediated adsorption mechanisms [66,67]. This is particularly relevant because serum-derived BCs enriched with lipoprotein-associated lipids have been shown in prior studies to influence NP biological identity, cellular interactions, downstream delivery, and in vivo efficacy [68].

Sharp increases in lipid diversity from 10% to 25% serum in male samples and from 25% to 50% serum in female samples suggest a transition to more compositionally heterogeneous BCs as biomolecule availability increased [26]. The delayed transition toward increased lipid diversity in female serum compared with male serum further suggests that biological sex influences the concentration threshold at which a more complex BC emerges, which is consistent with reports that male and female biofluids can generate distinct BC profiles [32,33,58,69].

Across all particle sizes and sexes, BCs formed in 50% and 75% serum exhibited extensive overlap in lipid composition, suggesting a plateau in lipid accumulation at higher serum concentrations and lower relative NP doses. A similar pattern was reported by Partikel et al., who observed that protein binding to PLGA NPs increased with increasing human serum exposure but approached a plateau at higher serum concentrations, suggesting saturation of available binding interactions [26]. Among the lipids shared under these higher-serum conditions were multiple CE species, including CE(20:4), CE(20:5), CE(22:4), CE(22:5), and CE(22:6), which in several groups co-occurred with LPC(20:4), PIs, PEs, and PCs. Together, these profiles suggest that higher-serum BCs became increasingly serum-representative and enriched in lipoprotein-associated lipids, consistent with prior lipidomic studies showing that BCs can incorporate lipids through interactions with circulating lipoprotein complexes [66,67].

### 3.4. Comparison of Relative Lipid Abundance of the BC Across Serum Concentrations

Although many lipids were shared across serum concentrations, quantitative differences in relative lipid abundance were observed as serum concentration increased. To evaluate concentration-dependent changes in individual lipid abundance in Fe_3_O_4_ NP-BCs, relative lipid abundances were assessed across increasing serum concentrations, and statistically significant correlations were identified for each experimental group (complete list of *p*-values in Supplemental Table S6). Across all NP sizes and sexes, the strongest correlations demonstrated increasing lipid abundance with increasing serum concentration, reflecting the lipid interactions occurring as NP doses decrease. While concentration-dependent shifts in the abundance of individual biomolecules in BCs remain relatively understudied, prior studies have shown that increasing biofluid exposure can reshape BC composition and promote selective enrichment of distinct protein and lipid classes [26,66,67].

**Figure 3:**
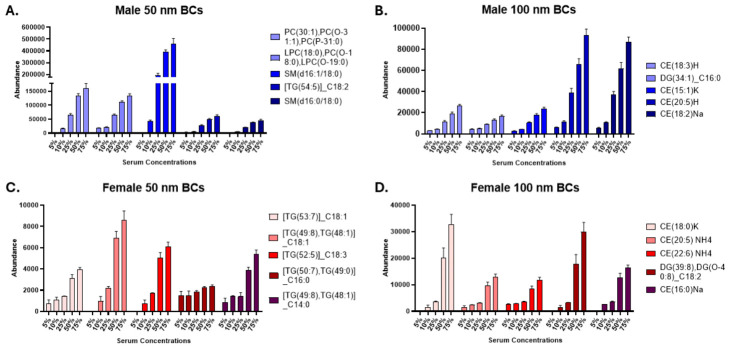
Relative abundance comparison of the top five most significantly correlated lipids in relation to serum concentration for (**A**) male 50 nm, (**B**) male 100 nm, (**C**) female 50 nm, and (**D**) female 100 nm BC samples. Plotted values represent relative lipid abundance based on MRM signal intensity, expressed as mean ± SEM (n = 3–5/group). Associations between serum concentration and lipid abundance were evaluated using Pearson correlation analysis (*p* < 0.05). A comprehensive list of individual *p*-values and R^2^ values is provided in Table S6.

For BCs formed on 50 nm Fe_3_O_4_ NPs in male serum, relative lipid abundance increased in a stepwise manner across serum concentrations (Figure 3A). The most significant correlations were PC(30:1),PC(O-31:1),PC(P-31:0) (R^2^ = 0.96, *p* = 0.0030); LPC(18:0),PC(O-18:0),LPC(O-19:0) (R^2^ = 0.96, *p* = 0.0033); SM(d16:1/18:0) (R^2^ = 0.96, *p* = 0.0036); TG(54:5)_C18:2 (R^2^ = 0.96, *p* = 0.0037); and SM(d16:0/18:0) (R^2^ = 0.95, *p* = 0.0041). A similar concentration-dependent increase in relative lipid abundance was observed for the male 100 nm Fe_3_O_4_ NP-BCs (Figure 3B). The most significant correlations were CE(18:3)H (R^2^ = 0.99, *p* = 0.00016); DG(34:1)_C16:0 (R^2^ = 0.99, *p* = 0.00028); CE(15:1)K (R^2^ = 0.99, *p* = 0.00038); CE(20:5)H (R^2^ = 0.99, *p* = 0.00041); and CE(18:2)Na (R^2^ = 0.99, *p* = 0.00042). For female 50 nm Fe_3_O_4_ NP-BCs (Figure 3C) the most significant correlations were TG(53:7)_C18:1 (R^2^ = 0.97, *p* = 0.0014); TG(49:8),TG(48:1)_C18:1 (R^2^ = 0.97, *p* = 0.0020); TG(52:5)_C18:3 (R^2^ = 0.97, *p* = 0.0026); TG(50:7),TG(49:0)_C16:0 (R^2^ = 0.96, *p* = 0.003); and TG(49:8),TG(48:1)_C14:0 (R^2^ = 0.96, *p* = 0.0032). The most significant correlations for the female 100 nm Fe_3_O_4_ NP-BCs were CE(18:0)K (R^2^ = 0.97, *p* = 0.0019); CE(20:5)NH4 (R^2^ = 0.97, *p* = 0.0021); CE(22:6)NH4 (R^2^ = 0.97, *p* = 0.0021); DG(39:8),DG(O-40:8)_C18:2 (R^2^ = 0.97, *p* = 0.0021); and CE(16:0)Na (R^2^ = 0.97, *p* = 0.0023).

The observed increases in PCs and their lyso counterparts in the male 50 nm Fe_3_O_4_ NP-BCs are especially notable as LPC (18:0) has been shown to alter membrane curvature and induce initial vesicle formation, whereas PCs are known to be major structural lipids involved in membrane fluidity, endocytosis, and have been associated with endosomal escape in lipid NP delivery systems [70,71]. Increases in these lipid species in the BC may alter the lipid interface presented at the NP surface, with potential relevance to membrane interactions, NP uptake, and delivery, based on prior literature. Additionally, concentration-dependent increases in SMs suggest increasing lipid raft membrane interactions as SM is a major component of cholesterol-rich rafts [72]. Recent lipid NP work also shows that increasing SM can improve cellular uptake and protein expression in mRNA delivery systems [73]. The strong concentration-dependent increases in multiple CE species for the male and female 100 nm Fe_3_O_4_ NP-BCs are particularly notable because CEs are characteristic components of circulating lipoprotein cargo, and prior lipidomic studies have identified CEs as recurring components of plasma-derived NP-BCs [66,74]. This may be relevant to drug delivery as prior studies have shown that cholesterol modifications can influence lipid NP performance, and esterified cholesterol improved nucleic acid delivery relative to regular or oxidized cholesterol in a large in vivo screen [75].

Unlike the gradual increases in lipid abundance across concentrations that were observed in the male BCs, female BCs exhibited more pronounced increases at higher serum concentrations, particularly starting at 50% serum. These distinct abundance patterns observed between male and female BCs may reflect underlying sex-dependent differences in the serum lipid environment available for adsorption and sex-specific NP corona profiles have also been reported following exposure to male versus female plasma [30,32]. This, along with the observed delayed increase in lipid diversity, further supports that biological sex influences the concentration threshold at which particular lipid classes become strongly represented within the BC.

### 3.5. Lipid Binding Patterns Within Fe_3_O_4_ NP-BCs

To assess how lipid abundance in serum is related to lipid association with Fe_3_O_4_ NP-BCs, the relative rank of individual lipid species in serum-only controls was compared with their corresponding rank in BC samples. This rank-based comparison revealed some lipid binding patterns across experimental groups were abundance-driven while others were not, indicating that lipid association with the NP surface was sometimes a reflection of serum lipid abundance but also selective binding behaviors. Similar abundance- and affinity-driven interactions have been described in the protein corona literature through the Vroman effect, in which highly abundant serum proteins, such as albumin, may initially dominate NP binding but are then displaced by lower abundance, higher affinity proteins [76,77,78]. Recent lipidomic studies have also shown that BC composition can be partly determined by the biofluid lipid profile while still exhibiting preferential binding of specific lipids, such as glycerolipids, SMs, CERs, and CEs, supporting the coexistence of abundance-driven and affinity-driven behaviors [66,79].

One binding pattern observed in this study across all groups was abundance-driven binding, in which lipid species that were highly ranked in serum due to their relative abundance also ranked highly within corresponding BC samples. This pattern was best exemplified by the male 50 nm group, where the three most abundant lipids in male serum PC(34:2), PC(O-35:2), PC(P-35:1); PC(O-38:9), PC(36:2), PC(O-37:2), PC(P-37:1); and PC(36:4), PC(O-37:4) were generally ranked among the most abundant lipids detected in BCs formed in 5%, 10%, 25%, 50%, and 75% BC serum (Table 1). The persistently high rank of PCs across all BC groups likely reflects the association of prevalent circulating phospholipids with the NP surface, rather than strong selective enrichment, as PCs are major constituents of human plasma and lipoprotein fractions [80,81,82,83]. In this context, abundance-driven binding of common serum phospholipids may contribute to the formation of a more membrane-like BC interface, which could influence how the NP initially interacts with cellular membranes during uptake.

In contrast, high-affinity binding patterns were identified, characterized by lipid species that were low ranked in serum but exhibited substantially higher ranks within BC samples, indicating preferential association with the NP surface despite low serum abundance. This pattern was most clearly observed in the male 100 nm group, where the lipids DG(37:7), DG(36:0)_C18:0; CE(20:3) NH4; and DG(37:7), DG(36:0)_C16:0 were among the lowest ranked species in serum, ranked 95th, 148th, and 234th, respectively, but shifted to markedly higher ranks within BC samples across serum concentrations (Table 2). More broadly, this type of binding implies that relatively minor serum lipids can become disproportionately represented in the BC and contribute to NP identity in ways that would not be predicted from serum abundance alone. For example, although CE(20:3) was only the 148th most abundant in the serum, in the BC samples it ranked between the 19th and 78th most abundant. Because CEs are carried within circulating lipoproteins, the selective recruitment of CE species supports the idea that BCs can become enriched with lipoprotein-derived lipids [84]. This may be relevant to drug delivery because lipoprotein-associated BC features have been shown in previous studies to alter NP biological identity and in vivo performance [66,68].

Low-affinity binding patterns were also observed, in which lipid species that were relatively abundant and highly ranked in serum exhibited lower ranks or were not detected in the corresponding BC samples. This pattern was most evident in female BCs formed on both 50 nm and 100 nm NPs, where lipids including DG(32:0)_C16:0; DG(34:0)_C18:0; DG(34:0)_C16:0; SM(d16:1/18:0); LPC(18:0), PC(O-18:0), LPC(O-19:0); and PC(38:4) were among the most abundant lipids in serum, ranked within the top 12, yet were not present in the corresponding BC samples (Table 3 and Table 4). These interactions demonstrate that abundant serum lipids were not uniformly incorporated into the BC and instead underwent selective exclusion, reinforcing that serum abundance alone was insufficient to predict NP association. For example, the exclusion of PC(38:4), a prevalent PC species in human lipoprotein fractions and membrane bilayers, suggests that even abundant serum phospholipids were not automatically incorporated into the BC [83]. Selective loss of otherwise abundant serum lipids helps shape the final BC and therefore the biological identity presented at the NP surface, with potential downstream consequences for cellular interactions, uptake, and biodistribution.

### 3.6. Comparison of Lipid Components of the BC Between Sexes

**Figure 4:**
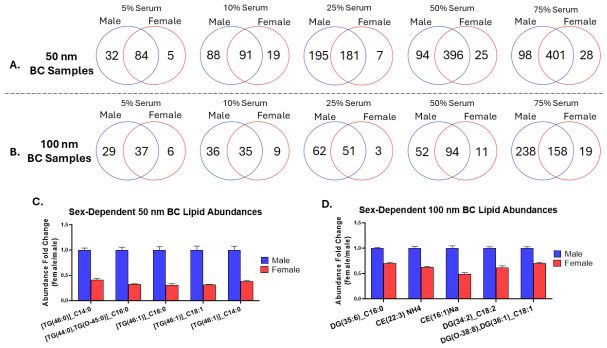
Sex-dependent differences in lipid profiles associated with Fe_3_O_4_ NP-BCs. Lipid profiles were compared between male- and female-derived Fe_3_O_4_ NP-BCs for (**A**) 50 nm BC samples and (**B**) 100 nm BC samples. Venn diagrams were used to illustrate the number of unique and shared lipid species present in male and female BCs at the same serum concentration. Present lipids were determined if intensities were at least 25% above the blank in at least three out of five samples in each group. Relative abundance differences between male and female 75% serum-derived BCs are shown for the five most significantly alerted lipids in (**C**) 50 nm and (**D**) 100 nm BC samples. Plotted values represent fold change in relative lipid abundance compared with male BCs and are expressed as mean ± SEM (n = 3–5/group). Statistical comparisons were performed using *t*-tests (*p* < 0.05). Individual adjusted *p*-values are provided in Table S7.

Sex-dependent differences in lipid association with Fe_3_O_4_ NP-BCs were assessed by comparing the number of lipids detected in male and female BCs at equivalent NP sizes and serum concentrations (complete list of lipids in Supplemental Table S7). Across all conditions, female BCs contained fewer total lipid species compared to male BCs. The majority of lipids determined to be present in female BCs were also present in the corresponding male BCs, whereas male BCs consistently contained a greater number of unique lipid species. For 50 nm Fe_3_O_4_ NPs, this trend was most evident at the 25% serum concentration, where male-derived BCs contained 195 lipid species unique to the male condition, while 181 lipids were shared between male and female BCs. In contrast, female-derived BCs at this concentration had only 7 unique lipids not detected in the corresponding male BCs (Figure 4A). Sex-dependent differences were also observed for the 100 nm Fe_3_O_4_ NPs. At 75% serum, male BCs exhibited 238 lipids unique to the male condition, while 158 lipid species were present in the BCs for both sexes. In comparison, female BCs at this concentration contained merely 19 unique lipids not detected in male BCs (Figure 4B).

The lipids shared between sexes are largely CEs, PCs, DGs, SMs, further supporting the presence of a common serum- or lipoprotein-derived corona core. This is consistent with serum lipid biology as lipoproteins carry CEs and glycolipids in their hydrophobic core and PCs and SMs at the surface [66,84]. Among the lipids unique to male BCs were LPC(16:0), LPC(20:3), LPC(22:5), LPC(22:6), LPI(20:0), PI(38:4), PI(36:2), and Cer(d18:0/21:0). This pattern aligns with human lipidomic studies indicating that males generally exhibit higher circulating lysophospholipid and CER levels compared to females [30,85]. Male BCs enriched in lyso species may have potential relevance to NP delivery as prior studies indicate that lysophospholipids promote positive membrane curvature and membrane remodeling, properties that may influence NP-membrane interactions and uptake [86]. By contrast, female only lipids included PS(38:4), PS(P-37:0), LPE(20:4), PE(38:5), PE(O-38:8)/PE(36:1)/PE(O-37:1)/PE(P-37:0), and TG(58:8)_C22:6. These species are enriched in aminophospholipids and polyunsaturated fatty acids, which is also consistent with broader literature showing that women an exhibit higher circulating phospholipid arachidonate and DHA levels than men [30,85]. This kind of enrichment may have direct implications on NP uptake as PS has been used to enhance macrophage uptake of NPs while PE has been shown to improve delivery of lipid NPs both in vitro and in vivo [87,88,89,90].

Although omics-based studies directly comparing male and female BCs remain limited, prior work suggests that biological sex can significantly alter BC composition and downstream NP behavior [32,35,58,69]. For example, Hayashi et al. demonstrated that 70 nm SiO_2_ NPs formed distinct protein coronas in male and female zebrafish plasma, with female BCs dominated by vitellogenins and male coronas enriched in other proteins including fetuin [69]. Functionally, lymphoid and myeloid blood cells preferentially engulfed NPs bearing the female corona, demonstrating that sex-specific BC composition can directly alter NP uptake [69]. Baseline differences in circulating lipid environments likely contribute to these effects. Current lipidomic literature indicates that women generally exhibit higher circulating PC and SM concentrations and lower CER and lysophospholipid concentrations than men, although these patterns are age- and reproductive stage-dependent [30]. These differences are thought to arise from a combination of sex-chromosome effects and sex-hormone signaling, particularly estrogen-dependent regulation of hepatic and adipose lipid metabolism [91,92]. Given this information, the greater number of unique lipids observed in male Fe_3_O_4_ NP-BCs likely reflects both sex-dependent differences in the serum lipidome available for adsorption as well as sex-dependent variations in NP–lipid interactions. Overall, these comparisons demonstrate that lipid association with Fe_3_O_4_ NPs is strongly influenced by biological sex, with male BCs consistently exhibiting greater lipid diversity and a higher degree of unique lipid association compared to female BCs across all NP sizes and serum concentrations.

### 3.7. Comparison of Relative Lipid Abundance of the BC Between Sexes

Although many lipids were shared between male and female BCs, notable sex-dependent differences in lipid abundance were observed for both NP sizes. For 50 nm Fe_3_O_4_ NP-BCs, a total of 108 lipid species showed significant differences in relative abundance between sexes (Table S7). Among the five most statistically significant abundance differences, male BCs exhibited higher relative abundance compared to the female BCs (Figure 4C). Similarly, for the 100 nm Fe_3_O_4_ NP-BCs, 82 lipids demonstrated significant sex-dependent differences in relative abundance (Table S7). The five most significantly different lipid species were more abundant in male BCs compared to female BCs (Figure 4D). These findings demonstrate that, beyond differences in lipid presence, sex also significantly influences the relative abundance of lipid species associated with BCs, with males consistently exhibiting higher abundance of numerous lipids regardless of NP size. A similar concept has been reported in the protein corona literature by Ashkarran et al. This study observed significant sex differences in the abundance of BC-associated proteins on silica NPs after only 1 h of incubation in male or female mouse plasma. They identified 17, 4, and 4 proteins that differed in concentration between males and females on three different NP types [32]. While their study focused on proteins and a shorter incubation period, it supports the broader concept that biological sex can influence both lipid recruitment and relative abundance of individual lipid species within NP-BCs.

### 3.8. Comparison of Lipid Components of the BC Between NP Sizes

**Figure 5:**
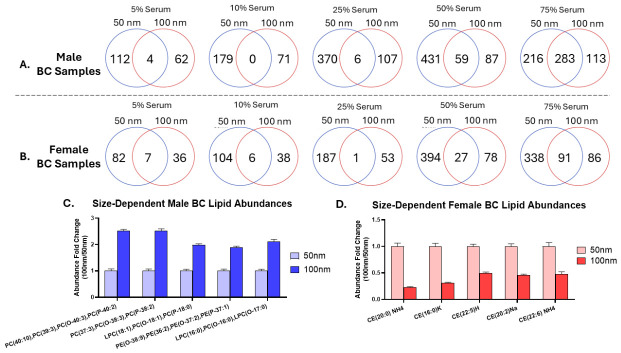
NP size-dependent differences in lipid profiles associated with Fe_3_O_4_ NP-BCs. Lipid profiles were compared between 50 nm and 100 nm Fe_3_O_4_ NP-BCs for (**A**) male BC samples and (**B**) female BC samples. Venn diagrams were used to illustrate the number of unique and shared lipid species present in 50 nm and 100 nm Fe_3_O_4_ NP-BCs at the same serum concentration. Present lipids were determined if intensities were at least 25% above the blank in at least three out of five samples in each group. Relative abundance differences between 50 nm and 100 nm 75% serum-derived BCs are shown for the five most significantly alerted lipids in (**C**) male and (**D**) female BC samples. Plotted values represent fold change in relative lipid abundance compared with 50 nm BCs and are expressed as mean ± SEM (n = 3–5/group). Statistical comparisons were performed using *t*-tests (*p* < 0.05). Individual adjusted *p*-values are provided in Table S8.

Differences in lipid association with Fe_3_O_4_ NP-BCs were assessed by comparing lipid species detected on 50 nm and 100 nm particles within each experimental group (complete list of lipids in Supplemental Table S8). Across all serum concentrations and both sexes, lipid compositions of BCs formed on each size of Fe_3_O_4_ NP were markedly different. Because these comparisons were performed using equal NP mass, as therapeutic and experimental NP dosing is commonly performed on a mass basis, rather than equal total surface area, the differences in lipid diversity may be partly driven by greater available surface area. Therefore, these findings are interpreted as size-associated differences in lipid recruitment, with surface area, curvature, and other size-related factors potentially contributing to the observed difference in BC profiles. In all cases, BCs formed on 50 nm NPs contained substantially more lipids than those formed on the surface of 100 nm NPs. Additionally, comparisons between NP sizes revealed limited overlap in lipids present in 50 nm and 100 nm BC samples. For example, male BCs at 10% serum had no shared lipid species between the two sizes. At this concentration, BCs formed on 50 nm NPs had 179 lipid unique to that size, while BCs formed on 100 nm NPs had 71 unique lipids (Figure 5A). A similarly limited overlap was observed for female BCs. At 25% serum, only a single lipid species, PG(16:0), LPG(17:0), LPG(O-18:0); PG(16:0), LPG(17:0), LPG(O-18:0), was shared between BCs formed on 50 nm and 100 nm NPs. At this concentration, BCs formed on 50 nm NPs contained 187 lipids unique to that size, whereas BCs formed on 100 nm NPs contained 53 unique lipids (Figure 5B). These comparisons illustrate pronounced NP size-dependent differences in lipid association with Fe_3_O_4_ NP-BCs. Across sexes and serum concentrations, BCs formed on 50 nm NPs consistently exhibited greater lipid diversity and limited overlap with BCs formed on 100 nm NPs, indicating that NP size is a major determinant of lipid composition within the BC.

This inverse size-dependent trend is consistent with prior lipid corona work showing that smaller NPs can acquire more lipids than larger particles under the same serum conditions. For example, Kobos et al. reported that 20 nm PVP NPs bound more lipids than 100 nm PVP NPs in both healthy and obese serum, supporting the same overall direction observed here [22]. More broadly, size-dependent BC formation has also been reported for Fe_3_O_4_ NPs and other nanomaterials, further indicating that NP size can significantly alter the composition of associated biomolecules [42,63]. However, because the direction of the NP size effect is known to vary across NP systems, the greater lipid diversity observed in this study for 50 nm Fe_3_O_4_ NP-BCs likely reflects a system-specific trend rather than a universal rule that smaller NPs always adsorb more lipids [39,40,93].

Among the size-dependent lipids identified in both males and females, the 50 nm Fe_3_O_4_ NP-BCs were enriched in membrane phospholipids, including several PC, LPC, SM, and PS lipid species such as PC(38:4), PC(38:6), LPC(18:0), SM(d16:1/18:0), and PS(25:0). This is notable because phospholipid chemistry has been shown to influence membrane fusion and endosomal escape in LNP systems, lysophospholipids promoted membrane curvature and remodeling, high SM formulations have been associated with improved protein expression, and PS-containing nanocarriers have been used to enhance macrophage uptake [71,73,89]. In contrast, the 100 nm Fe_3_O_4_ NP-BCs were enriched in CE and DG species such as CE(20:3), CE(20:4), CE(20:5), DG(34:2)_C18:2, and DG(40:5)_C18:0, suggesting greater association with lipoprotein-derived neutral lipid cargo, potentially relevant to drug delivery because lipoprotein-rich BC, particularly HDL-associated BCs, have been linked to altered NP identity and improved in vivo LNP activity [68]. Together, these findings suggest that NP size influenced not only the number of lipids associated with the BC, but also the lipid interface presented at the NP surface, which may affect cellular interactions, NP uptake, and trafficking based on prior literature.

### 3.9. Comparison of Relative Lipid Abundance of the BC Between NP Sizes

Although lipid overlap between BCs formed on 50 nm and 100 nm Fe_3_O_4_ NPs was limited, BCs formed at 75% serum shared a subset of lipid species and notable NP size-dependent differences in lipid abundance were observed for both sexes. For male BCs, a total of 87 lipid species exhibited significant differences in relative lipid abundance between NP sizes (Table S8). Among the most statistically significant abundance differences, BCs formed on 100 nm NPs exhibited higher relative abundance compared to those formed on 50 nm NPs (Figure 5C). In contrast, female BCs demonstrated 23 significantly different lipids in lipid abundance between 50 nm and 100 nm NPs (Table S8), with the most significantly altered lipids exhibiting higher relative abundance in BCs formed on 50 nm NPs compared to those formed on 100 nm NPs (Figure 5D). Amongst the most significantly different lipid species in the female 50 nm Fe_3_O_4_ NP-BCs and 100 nm Fe_3_O_4_ NP-BCs were several CEs including CE(20:0) NH4, CE(16:0)K, CE(22:5)H, CE(20:2)Na, and CE(22:6) NH4. The observation that female 50 nm Fe_3_O_4_ NP-BCs contained higher abundance of several shared CE species, despite broader CE representation in 100 nm BCs, suggests that lipid diversity and lipid enrichment should be interpreted separately. Larger particles may recruit a wider CE collection, whereas smaller particles may more strongly enrich specific shared CE lipids. These findings demonstrate that NP size influences not only the composition of lipids associated with Fe_3_O_4_ NP-BCs but also the relative abundance of shared lipid species. Notably, the direction of NP size-dependent abundance differences differed by sex, with male BCs having higher abundance of shared lipids on 100 nm NPs, whereas female BCs had higher abundance of shared lipids on 50 nm NPs, suggesting that the abundance differences may also be sex-dependent.

## 4. Conclusions

This study demonstrates Fe_3_O_4_ NP-BCs are sensitive to their surrounding biological environment and NP design variables such as dose and particle size. Increasing serum concentration, reflective of decreasing NP dose, produced markedly more diverse lipid coronas. Biological sex emerged as an important factor influencing BC formation, impacting not only which lipids are associated with the NP surface but also their relative abundance once bound. NP size also had a strong effect as 50 nm Fe_3_O_4_ NPs generally acquired more diverse lipid coronas than their 100 nm Fe_3_O_4_ NP counterparts, while the identities and abundances of shared lipids also differed in a size-dependent manner.

While this study addresses an underexplored aspect of BC research by focusing on lipid corona formation, several limitations should be considered. First, particles were normalized by equal mass in the same volume, reflecting how therapeutic NP dosing is often defined and administered by mass in biomedical applications. This approach is also a common method in NP-BC studies and was also used in prior size-dependent BC studies; however, for nanoscale materials, surface area is also an important dose metric and may better reflect the opportunities available for biomolecule adsorption. Because 50 nm NPs have greater surface area per unit mass than 100 nm NPs, the increased lipid diversity observed for 50 nm Fe_3_O_4_ NP-BCs may be influenced by greater available surface area in addition to particle curvature or other size-related properties. The hydrodynamic size and mass concentration data provided in this study allow readers to consider these relationships mathematically; however, direct experimental normalization by surface area was not performed. Future studies, including ongoing work from our lab, should therefore directly compare equal surface area exposures of different size NPs to distinguish mass-driven from surface area-driven effects more clearly. Second, BC formation is dynamic rather than static, with continuous competitive exchange of biomolecules over time. Because exposure duration and circulation time can alter both corona composition and abundance, time course studies that model circulation through varying serum incubation times will be important for determining how these lipid coronas evolve, stabilize, or rearrange over time. Further, this study utilized pooled male and female human serum to systematically evaluate the effects of serum concentration, biological sex, and NP size while minimizing donor-specific variability. This approach is consistent with prior BC studies and was appropriate for isolating the experimental variables assessed here; however, use of pooled serum limits evaluation of interindividual variability. Future studies using serum from individual donors will be necessary to determine how donor-specific differences in lipid profiles, health status, age, and other biological factors influence Fe_3_O_4_ NP–lipid BC formation. Additional work is also needed to determine how generalizable these findings are across nanomaterials. Prior literature shows that BC composition depends not only on particle size, but also on NP material, surface chemistry, and morphology, making it likely that the trends observed here for Fe_3_O_4_ will not translate identically to all nanomaterials. Expanding these analyses to additional NP types, coatings, and geometries will therefore be important. Finally, this study was designed to characterize lipid corona formation and did not directly assess downstream functional endpoints such as cellular uptake, cytotoxicity, inflammatory activation, or delivery efficiency. Although prior work from our laboratory and others has demonstrated that BC formation can alter NP-cell interactions and uptake, future studies will be needed to determine how the lipid corona profiles identified here influence cellular responses. The present findings provide a foundation for selecting the most distinct serum concentration, sex, and NP size conditions for subsequent functional analyses.

Even with these limitations, the present study provides several important advances. It demonstrates that higher serum conditions generate substantially more diverse lipid coronas, that NP size is a readily tunable design parameter capable of strongly reshaping BC composition, and that biological sex can meaningfully alter both lipid recruitment and enrichment within the corona. Together, these findings emphasize that lipid corona formation should be considered during nanomedicine development, not only to better predict in vivo behavior but also to help guide the design of safer nanocarriers, more reliable delivery systems, and more personalized diagnostic or therapeutic platforms.

## Figures and Tables

**Table 1:** Lipid binding patterns in 50 nm Fe_3_O_4_ NP-BCs formed in male serum. Lipid binding behavior was classified as abundance, high affinity, or low affinity, driven by comparing the relative abundance rank of individual lipid species in male serum to their corresponding rank in Fe_3_O_4_ NP-BCs formed at 5%, 10%, 25%, 50%, and 75% serum concentrations. Abundance-driven lipids maintained similar ranks between serum and NP-BCs, high-affinity-driven lipids exhibited higher ranks in the NP-BCs relative to serum, and low-affinity-driven lipids exhibited lower ranks in the NP-BCs relative to serum.

	Lipid Name	Serum Rank	5% Rank	10% Rank	25% Rank	50% Rank	75% Rank
Abundance	PC(34:2),PC(O-35:2),PC(P-35:1)	1	9	3	1	3	3
	PC(O-38:9),PC(36:2),PC(O-37:2),PC(P-37:1)	2	120	4	3	5	5
	PC(36:4),PC(O-37:4)	3	18	18	6	9	8
High Affinity	[TG(51:8),TG(50:1)]_C18:1	78	26	34	27	33	34
	[TG(49:7),TG(48:0)]_C16:0	173	15	26	50	59	56
	[TG(51:7),TG(50:0)]_C16:0	201	23	38	64	75	73
Low Affinity	LPC(18:1),PC(O-18:1),PC(P-18:0)	32	-	-	139	127	123
	CE(20:5)H	42	-	-	-	-	-
	CE(18:2)Na	49	-	-	-	-	-

**Table 2:** Lipid binding patterns in 100 nm Fe_3_O_4_ NP-BCs formed in male serum. Lipid binding behavior was classified as abundance, high affinity, or low affinity, driven by comparing the relative abundance rank of individual lipid species in male serum to their corresponding rank in Fe_3_O_4_ NP-BCs formed at 5%, 10%, 25%, 50%, and 75% serum concentrations. Abundance-driven lipids maintained similar ranks between serum and NP-BCs, high-affinity-driven lipids exhibited higher ranks in the NP-BCs relative to serum, and low-affinity-driven lipids exhibited lower ranks in the NP-BCs relative to serum.

	Lipid Name	Serum Rank	5% Rank	10% Rank	25% Rank	50% Rank	75% Rank
Abundance	CE(18:2) NH4	12	6	5	1	1	1
	DG(O-40:9),DG(38:2)_C18:2	13	7	6	2	2	2
	CE(20:4) NH4	27	11	9	8	8	22
High Affinity	DG(37:7),DG(36:0)_C18:0	95	4	4	6	9	34
	CE(20:3) NH4	148	37	26	24	19	78
	DG(37:7),DG(36:0)_C16:0	234	23	17	22	18	88
Low Affinity	LPC(16:0),PC(O-16:0),LPC(O-17:0)	4	-	-	-	-	17
	DG(36:7),DG(35:0)_C16:0	87	-	-	-	-	-
	FA(19:0)	98	-	-	-	-	-

**Table 3:** Lipid binding patterns in 50 nm Fe_3_O_4_ NP-BCs formed in female serum. Lipid binding behavior was classified as abundance, high affinity, or low affinity, driven by comparing the relative abundance rank of individual lipid species in female serum to their corresponding rank in Fe_3_O_4_ NP-BCs formed at 5%, 10%, 25%, 50%, and 75% serum concentrations. Abundance-driven lipids maintained similar ranks between serum and NP-BCs, high-affinity-driven lipids exhibited higher ranks in the NP-BCs relative to serum, and low-affinity-driven lipids exhibited lower ranks in the NP-BCs relative to serum.

	Lipid Name	Serum Rank	5% Rank	10% Rank	25% Rank	50% Rank	75% Rank
Abundance	[TG(53:9),TG(52:2)]_C18:1	11	13	18	8	9	10
	[TG(53:10),TG(52:3)]_C18:1	19	18	21	16	14	15
	[TG(53:10),TG(52:3)]_C16:0	20	19	24	15	15	14
High Affinity	[TG(53:8),TG(52:1)]_C18:1	77	32	46	45	45	46
	[TG(55:10),TG(54:3)]_C18:2	136	68	78	75	81	77
	[TG(39:0)]_C20:0	201	93	107	138	106	94
Low Affinity	DG(32:0)_C16:0	5	-	-	-	-	-
	DG(34:0)_C18:0	8	-	-	-	-	-
	DG(34:0)_C16:0	12	-	-	-	-	-

**Table 4:** Lipid binding patterns in 100 nm Fe_3_O_4_ NP-BCs formed in female serum. Lipid binding behavior was classified as abundance, high affinity, or low affinity, driven by comparing the relative abundance rank of individual lipid species in female serum to their corresponding rank in Fe_3_O_4_ NP-BCs formed at 5%, 10%, 25%, 50%, and 75% serum concentrations. Abundance-driven lipids maintained similar ranks between serum and NP-BCs, high-affinity-driven lipids exhibited higher ranks in the NP-BCs relative to serum, and low-affinity-driven lipids exhibited lower ranks in the NP-BCs relative to serum.

	Lipid Name	Serum Rank	5% Rank	10% Rank	25% Rank	50% Rank	75% Rank
Abundance	CE(18:2) NH4	1	11	9	6	2	3
	DG(O-40:9),DG(38:2)_C18:2	2	12	10	7	3	3
	DG(32:0)_C16:0	5	1	1	1	4	6
High Affinity	CE(18:2)Na	41	32	20	11	14	22
	DG(34:1)_C18:1	118	46	41	41	38	62
	DG(32:0)_C18:0	211	27	27	35	72	110
Low Affinity	SM(d16:1/18:0)	3	-	-	-	-	-
	LPC(18:0),PC(O-18:0),LPC(O-19:0)	4	-	-	-	-	-
	PC(38:4)	6	-	-	-	-	-

## Data Availability

The original contributions presented in this study are included in the article/Supplementary Materials. Further inquiries can be directed to the corresponding author.

## Supplementary Materials

The following supporting information can be downloaded at: https://www.mdpi.com/article/10.3390/nano16110683/s1, Table S1. Multiple reaction monitoring (MRMs) list of lipids and identifier. Table S2. Male 50 nm Comparison of Lipid Corona Profiles Between Serum Concentration. Table S3. Male 100 nm Comparison of Lipid Corona Profiles Between Serum Concentration. Table S4. Female 50 nm Comparison of Lipid Corona Profiles Between Serum Concentration. Table S5. Female 100 nm Comparison of Lipid Corona Profiles Between Serum Concentration. Table S6. Correlation Analysis of Fe_3_O_4_ NP-BC Lipid Abundance in Relation to Serum Concentration. Table S7. Comparison of Lipid Corona Profiles Between Sexes. Table S8. Comparison of Lipid Corona Profiles Between Nanoparticle Sizes.

## Abbreviations

NP	Nanoparticle
Fe_3_O_4_ NP	Iron oxide nanoparticle
BC	Biocorona
MRM	Multiple reaction monitoring
mRNA	Messenger RNA
PBS	Phosphate-buffered saline
DI	Deionized
CE	Cholesterol ester
CER	Ceramide
DG	Diacylglycerol
TG	Triglycerol
PC	Phosphatidylcholine
LPC	Lysophosphatidylcholine
PE	Phosphatidylethanolamine
LPE	Lysophosphatidylethanolamine
PI	Phosphatidylinositol
LPI	Lysophosphatidylinositol
PS	Phosphatidylserine
PG	Phosphatidylglycerol
LPG	Lysophosphatidylglycerol
SM	Sphingomyelin
