# Supplementary material for: Elucidating the Influence of Serum Concentration, Sex, and Particle Size on Iron Oxide Nanoparticle–Lipid Biocorona Formation
Source: Nanomaterials (Basel). 2026 Jun 1;16(11):683. doi: 10.3390/nano16110683 (PMC13258708; doi:10.3390/nano16110683)
Supplement: Supplementary file 1 [file nanomaterials-16-00683-s001.zip › nanomaterials-4334647-supplementary - 副本/Table S1. Multiple reaction monitoring (MRMs) list of lipids and identifier..pdf]

Table S1. Multiple reaction monitoring (MRMs) list of lipids and identifier.

Lipid name	MRM (Precursor Ion -> Product Ion)
[TG(53:9),TG(52:2)]_C18:1	876.80197 -> 577.50197
[TG(54:5)]_C18:1	898.78637 -> 599.48637
[TG(55:10),TG(54:3)]_C18:0	902.81767 -> 601.51767
[TG(53:9),TG(52:2)]_C16:0	876.80197 -> 603.50197
[TG(53:8),TG(52:1)]_C16:0	878.81767 -> 605.51767
[TG(55:11),TG(54:4)]_C18:2	900.80197 -> 603.50197
[TG(51:7),TG(50:0)]_C18:0	852.80197 -> 551.50197
[TG(49:7),TG(48:0)]_C16:0	824.77067 -> 551.47067
[TG(51:9),TG(50:2)]_C18:1	848.77067 -> 549.47067
[TG(51:7),TG(50:0)]_C16:0	852.80197 -> 579.50197
[TG(51:9),TG(50:2)]_C18:2	848.77067 -> 551.47067
[TG(51:9),TG(50:2)]_C16:0	848.77067 -> 575.47067
[TG(51:8),TG(50:1)]_C18:1	850.78637 -> 551.48637
[TG(54:6)]_C18:2	896.77067 -> 599.47067
[TG(49:8),TG(48:1)]_C16:0	822.75507 -> 549.45507
[TG(51:8),TG(50:1)]_C16:0	850.78637 -> 577.48637
PS(O-29:0)	680.486625 -> 495.486625
[TG(46:0)]_C16:0	796.73937 -> 523.43937
[TG(50:3)]_C18:1	846.75507 -> 547.45507
[TG(50:3)]_C18:2	846.75507 -> 549.45507
[TG(51:8),TG(50:1)]_C18:0	850.78637 -> 549.48637
[TG(53:7),TG(52:0)]_C18:0	880.83327 -> 579.53327
PC(34:1),PC(O-35:1),PC(P-35:0)	760.622025 -> 184.1
[TG(50:3)]_C16:0	846.75507 -> 573.45507
PC(32:0),PC(O-33:0)	734.606325 -> 184.1
[TG(50:3)]_C16:1	846.75507 -> 575.45507
[TG(51:9),TG(50:2)]_C16:1	848.77067 -> 577.47067
[TG(55:11),TG(54:4)]_C18:0	900.80197 -> 599.50197
[TG(50:7),TG(49:0)]_C16:0	838.78637 -> 565.48637
[TG(53:7),TG(52:0)]_C16:0	880.83327 -> 607.53327
PC(40:6)	834.601325 -> 184.1
[TG(54:10),TG(53:3)]_C18:1	888.80197 -> 589.50197
PC(36:3),PC(P-37:2)	784.622025 -> 184.1
[TG(52:4)]_C18:3	872.77067 -> 577.47067
SM(d16:0/22:0)	761.653625 -> 184.1
PC(O-38:9),PC(36:2),PC(O-37:2),PC(P-37:1)	786.637625 -> 184.1
PC(36:5)	780.554325 -> 184.1
[TG(51:9),TG(50:2)]_C14:0	848.77067 -> 603.57067
[TG(54:9),TG(53:2)]_C18:1	890.81767 -> 591.51767
[TG(50:3)]_C14:0	846.75507 -> 601.55507
[TG(52:9),TG(51:2)]_C18:1	862.78637 -> 563.48637
[TG(49:8),TG(48:1)]_C18:1	822.75507 -> 523.45507
[TG(39:0)]_C20:0	698.62987 -> 369.32987
PC(38:4)	810.601325 -> 184.1
PC(38:5)	808.585625 -> 184.1
[TG(52:5)]_C18:3	870.75507 -> 575.45507
[TG(51:7)]_C18:1	852.80197 -> 553.50197
SM(d18:2/22:1)	783.638025 -> 184.1
LPG(20:0); LPG(20:0)	558.37707 -> 369.37707
[TG(50:4)]_C18:2	844.73937 -> 547.43937

[TG(54:5)]_C18:3	898.78637 -> 603.48637
PC(40:4)	838.632625 -> 184.1
[TG(52:5)]_C16:0	870.75507 -> 597.45507
[TG(48:2)]_C16:0	820.73937 -> 547.43937
[TG(57:12),TG(56:5)]_C18:1	926.81767 -> 627.51767
PC(37:5),PC(O-38:5),PC(P-38:4)	794.606325 -> 184.1
[TG(54:6)]_C18:1	896.77067 -> 597.47067
PC(34:0),PC(O-35:0)	762.637625 -> 184.1
[TG(54:6)]_C20:4	896.77067 -> 575.47067
SM(d16:1/16:0)	675.544125 -> 184.1
[TG(48:2)]_C18:2	820.73937 -> 523.43937
[TG(51:8),TG(50:1)]_C16:1	850.78637 -> 579.48637
PC(28:1),PC(P-29:0)	676.528125 -> 184.1
PC(37:3),PC(O-38:3),PC(P-38:2)	798.637625 -> 184.1
PC(40:10),PC(39:3),PC(O-40:3),PC(P-40:2)	826.668925 -> 184.1
SM(d16:1/20:1)	729.591025 -> 184.1
PC(37:4),PC(O-38:4),PC(P-38:3)	796.622025 -> 184.1
PC(30:2),PC(P-31:1)	702.543725 -> 184.1
SM(d18:2/24:1)	811.669325 -> 184.1
[TG(54:5)]_C20:4	898.78637 -> 577.48637
SM(d16:1/18:0)	703.575425 -> 184.1
SM(d16:1/24:1)	785.653625 -> 184.1
PC(38:3)	812.616925 -> 184.1
SM(d16:0/18:0)	705.591025 -> 184.1
PC(40:5)	836.616925 -> 184.1
SM(d16:1/24:0)	787.669325 -> 184.1
PC(30:1),PC(O-31:1),PC(P-31:0)	704.559425 -> 184.1
PC(O-38:8),PC(36:1),PC(O-37:1),PC(P-37:0)	788.653325 -> 184.1
PC(30:0),PC(O-31:0)	706.575025 -> 184.1
SM(d16:1/17:0)	689.559725 -> 184.1
[TG(55:11),TG(54:4)]_C16:0	900.80197 -> 627.50197
PC(35:4),PC(O-36:4),PC(P-36:3)	768.590725 -> 184.1
SM(d16:0/20:0)	733.622325 -> 184.1
SM(d16:1/20:0)	731.606725 -> 184.1
PC(29:1),PC(O-30:1),PC(P-30:0)	690.543725 -> 184.1
SM(d16:1/18:1)	701.559725 -> 184.1
PC(31:0),PC(O-32:0)	720.590725 -> 184.1
[TG(49:8),TG(48:1)]_C14:0	822.75507 -> 577.55507
[TG(49:6)]_C16:0	826.69247 -> 553.39247
[TG(49:7),TG(48:0)]_C18:0	824.77067 -> 523.47067
[TG(56:6)]_C20:4	924.80197 -> 603.50197
PC(28:0),PC(O-29:0)	678.543725 -> 184.1
[TG(48:2)]_C18:1	820.73937 -> 521.43937
[TG(55:9),TG(54:2)]_C16:0	904.83327 -> 631.53327
[TG(55:9),TG(54:2)]_C18:2	904.83327 -> 607.53327
[TG(56:7)]_C22:6	922.88027 -> 577.58027
[TG(52:10),TG(51:3)]_C18:2	860.77067 -> 563.47067
[TG(52:9),TG(51:2)]_C16:0	862.78637 -> 589.48637
PC(38:9),PC(37:2),PC(O-38:2),PC(P-38:1)	800.653325 -> 184.1
PC(36:8),PC(35:1),PC(O-36:1),PC(P-36:0)	774.637625 -> 184.1
[TG(57:10),TG(56:3)]_C18:1	930.84897 -> 631.54897
[TG(54:10),TG(53:3)]_C18:2	888.80197 -> 591.50197

[TG(50:4)]_C16:1	844.73937 -> 573.43937
PC(35:3),PC(O-36:3),PC(P-36:2)	770.606325 -> 184.1
PC(31:1),PC(O-32:1),PC(P-32:0)	718.575025 -> 184.1
PC(32:1),PC(O-33:1),PC(P-33:0)	732.590725 -> 184.1
SM(d16:0/24:0)	789.684925 -> 184.1
[TG(48:2)]_C16:1	820.73937 -> 549.43937
[TG(49:8),TG(48:1)]_C16:1	822.75507 -> 551.45507
[TG(52:8),TG(51:1)]_C16:0	864.80197 -> 591.50197
[TG(56:7)]_C20:4	922.88027 -> 601.58027
[TG(51:8),TG(50:1)]_C14:0	850.78637 -> 605.58637
PC(40:8),PC(39:1),PC(O-40:1),PC(P-40:0)	830.700225 -> 184.1
[TG(53:9),TG(52:2)]_C16:1	876.80197 -> 605.50197
[TG(53:10),TG(52:3)]_C18:0	874.78637 -> 573.48637
[TG(55:10),TG(54:3)]_C16:0	902.81767 -> 629.51767
[TG(56:7)]_C18:2	922.88027 -> 625.58027
LPC(18:0),PC(O-18:0),LPC(O-19:0)	524.408025 -> 184.1
[TG(48:2)]_C14:0	820.73937 -> 575.53937
[TG(54:6)]_C18:3	896.77067 -> 601.47067
PC(37:7),PC(P-38:6),PC(36:0),PC(O-37:0)	790.668925 -> 184.1
[TG(54:5)]_C16:0	898.78637 -> 625.48637
SM(d18:1/17:0)	717.591025 -> 184.1
Cer(d18:1/24:0)	650.645125 -> 264.4
[TG(49:7),TG(48:0)]_C14:0	824.77067 -> 579.57067
[TG(46:2)]_C18:1	792.70807 -> 493.40807
[TG(50:8),TG(49:1)]_C18:1	836.77067 -> 537.47067
[TG(48:3)]_C16:1	818.72377 -> 547.42377
[TG(54:11),TG(53:4)]_C18:2	886.78637 -> 589.48637
[TG(57:11),TG(56:4)]_C18:2	928.83327 -> 631.53327
[TG(50:4)]_C14:0	844.73937 -> 599.53937
SM(d18:1/24:1(15Z))	813.684925 -> 184.1
PC(O-40:9),PC(38:2),PC(P-39:1)	814.668925 -> 184.1
PC(41:6),PC(O-42:6)	848.653325 -> 184.1
[TG(57:12),TG(56:5)]_C18:2	926.81767 -> 629.51767
[TG(56:10),TG(55:3)]_C18:1	916.83327 -> 617.53327
LPC(20:2),PC(O-20:2)	548.371625 -> 184.1
[TG(52:8),TG(51:1)]_C18:1	864.80197 -> 565.50197
SM(d17:1/24:1)	799.669325 -> 184.1
SM(d17:1/26:1)	827.700625 -> 184.1
PC(41:5),PC(P-42:4)	850.668925 -> 184.1
PC(42:3)	868.679525 -> 184.1
PC(44:12),PC(O-44:5)	878.700225 -> 184.1
[TG(52:10),TG(51:3)]_C18:1	860.77067 -> 561.47067
PC(39:4),PC(O-40:4),PC(P-40:3)	824.653325 -> 184.1
[TG(54:7)]_C18:2	894.84897 -> 597.54897
[TG(48:3)]_C18:2	818.72377 -> 521.42377
[TG(52:4)]_C20:4	872.77067 -> 551.47067
[TG(50:3)]_C18:3	846.75507 -> 551.45507
[TG(54:7)]_C18:3	894.84897 -> 599.54897
[TG(50:9),TG(49:2)]_C18:2	834.75507 -> 537.45507
[TG(51:9),TG(50:2)]_C18:0	848.77067 -> 547.47067
[TG(54:10),TG(53:3)]_C16:0	888.80197 -> 615.50197
[TG(52:5)]_C18:1	870.75507 -> 571.45507

PC(40:3)	840.648225 -> 184.1
[TG(55:8),TG(54:1)]_C16:0	906.84897 -> 633.54897
SM(d16:0/16:0)	677.559725 -> 184.1
SM(d18:0/17:0)	719.606725 -> 184.1
[TG(56:6)]_C16:0	924.80197 -> 651.50197
[TG(57:11),TG(56:4)]_C18:0	928.83327 -> 627.53327
PC(29:0),PC(O-30:0)	692.559425 -> 184.1
[TG(46:1)]_C18:1	794.72377 -> 495.42377
[TG(46:0)]_C14:0	796.73937 -> 551.53937
[TG(52:6)]_C18:3	868.73937 -> 573.43937
[TG(50:8),TG(49:1)]_C16:0	836.77067 -> 563.47067
[TG(57:12),TG(56:5)]_C16:0	926.81767 -> 653.51767
[TG(46:1)]_C16:0	794.72377 -> 521.42377
[TG(54:9),TG(53:2)]_C18:0	890.81767 -> 589.51767
SM(d16:0/25:0)	803.700625 -> 184.1
PC(40:7),PC(39:0),PC(O-40:0)	832.715925 -> 184.1
PC(38:7),PC(37:0),PC(O-38:0)	804.684625 -> 184.1
PC(42:4)	866.663925 -> 184.1
[TG(54:11),TG(53:4)]_C18:1	886.78637 -> 587.48637
SM(d18:2/21:0)	771.638025 -> 184.1
PC(40:1),PC(P-41:0)	844.715925 -> 184.1
[TG(56:8)]_C18:2	920.86457 -> 623.56457
PC(31:2),PC(O-32:2),PC(P-32:1)	716.559425 -> 184.1
PC(39:6),PC(O-40:6),PC(P-40:5)	820.622025 -> 184.1
PC(37:6),PC(O-38:6),PC(P-38:5)	792.590725 -> 184.1
[TG(51:8)]_C18:2	850.78637 -> 553.48637
PC(38:8),PC(37:1),PC(O-38:1),PC(P-38:0)	802.668925 -> 184.1
PC(36:7),PC(35:0),PC(O-36:0)	776.653325 -> 184.1
[TG(57:10),TG(56:3)]_C18:2	930.84897 -> 633.54897
[TG(51:4)]_C18:2	858.75507 -> 561.45507
PI(38:4)	904.5571 -> 627.5571
SM(d18:1/26:1(17Z))	841.716225 -> 184.1
[TG(54:11),TG(53:4)]_C16:0	886.78637 -> 613.48637
PC(28:2)	674.476125 -> 184.1
[TG(56:8),TG(55:1)]_C16:0	920.86457 -> 647.56457
PC(42:11),PC(41:4),PC(O-42:4)	852.684625 -> 184.1
PC(40:9),PC(39:2),PC(O-40:2),PC(P-40:1)	828.684625 -> 184.1
PE(O-38:9),PE(36:2),PE(O-37:2),PE(P-37:1)	744.590725 -> 603.590725
[TG(48:3)]_C16:0	818.72377 -> 545.42377
[TG(50:4)]_C16:0	844.73937 -> 571.43937
[TG(57:9),TG(56:2)]_C18:1	932.86457 -> 633.56457
[TG(50:9),TG(49:2)]_C16:0	834.75507 -> 561.45507
LPI(20:0)	646.3588 -> 369.3588
[TG(55:11),TG(54:4)]_C18:3	900.80197 -> 605.50197
[TG(37:0)]_C18:0	670.59857 -> 369.29857
[TG(48:3)]_C18:1	818.72377 -> 519.42377
[TG(53:10),TG(52:3)]_C18:3	874.78637 -> 579.48637
[TG(52:9),TG(51:2)]_C18:2	862.78637 -> 565.48637
[TG(55:9),TG(54:2)]_C20:0	904.83327 -> 575.53327
[TG(57:9),TG(56:2)]_C20:0	932.86457 -> 603.56457
[TG(57:10),TG(56:3)]_C20:0	930.84897 -> 601.54897
[TG(54:6)]_C16:0	896.77067 -> 623.47067

[TG(52:10),TG(51:3)]_C16:0	860.77067 -> 587.47067
[TG(55:11),TG(54:4)]_C20:4	900.80197 -> 579.50197
PC(33:0),PC(O-34:0)	748.622025 -> 184.1
[TG(52:5)]_C20:4	870.75507 -> 549.45507
LPC(16:0),PC(O-16:0),LPC(O-17:0)	496.376725 -> 184.1
PC(35:5),PC(O-36:5),PC(P-36:4)	766.575025 -> 184.1
SM(d18:0/24:1)	815.700625 -> 184.1
[TG(50:4)]_C18:1	844.73937 -> 545.43937
SM(d16:1/25:0)	801.684925 -> 184.1
SM(d18:1/25:0)	829.716225 -> 184.1
PC(39:8),PC(O-40:8),PC(38:1),PC(O-39:1),PC(P-39:0)	816.684625 -> 184.1
PC(40:2)	842.663925 -> 184.1
SM(d16:1/23:0)	773.653625 -> 184.1
SM(d18:0/15:0)	691.575425 -> 184.1
SM(d18:0/24:0)	817.716225 -> 184.1
[TG(56:8)]_C20:4	920.86457 -> 599.56457
PC(42:10),PC(41:3),PC(O-42:3),PC(P-42:2)	854.700225 -> 184.1
[TG(50:4)]_C18:3	844.73937 -> 549.43937
[TG(57:12),TG(56:5)]_C18:0	926.81767 -> 625.51767
[TG(54:9),TG(53:2)]_C16:0	890.81767 -> 617.51767
[TG(55:8),TG(54:1)]_C20:0	906.84897 -> 577.54897
LPC(18:1),PC(O-18:1),PC(P-18:0)	522.355925 -> 184.1
[TG(54:7)]_C20:4	894.84897 -> 573.54897
PC(35:6),PC(P-36:5)	764.559425 -> 184.1
PC(42:9),PC(41:2),PC(O-42:2),PC(P-42:1)	856.715925 -> 184.1
PC(42:8),PC(41:1),PC(O-42:1),PC(P-42:0)	858.731525 -> 184.1
PC(43:6)	876.648225 -> 184.1
PC(42:0)	874.726525 -> 184.1
[TG(56:12),TG(55:5)]_C18:1	912.80197 -> 613.50197
LPC(22:4)	572.371625 -> 184.1
PC(42:2)	870.695225 -> 184.1
PC(42:7),PC(41:0),PC(O-42:0)	860.747225 -> 184.1
PC(42:5)	864.648225 -> 184.1
PC(43:4),PC(O-44:4)	880.715925 -> 184.1
[TG(56:6)]_C18:0	924.80197 -> 623.50197
[TG(58:7)]_C22:5	950.91157 -> 603.61157
[TG(44:0),TG(O-45:0)]_C16:0	768.74447 -> 495.44447
[TG(54:8),TG(53:1)]_C18:0	892.83327 -> 591.53327
[TG(54:5)]_C22:5	898.78637 -> 551.48637
[TG(38:0)]_C14:0	684.61417 -> 439.41417
[TG(54:8),TG(53:1)]_C16:0	892.83327 -> 619.53327
[TG(57:12),TG(56:5)]_C22:5	926.81767 -> 579.51767
[TG(54:9),TG(53:2)]_C18:2	890.81767 -> 593.51767
Cer(d18:1/23:0)	636.629425 -> 264.4
[TG(58:9)]_C22:6	946.88027 -> 601.58027
[TG(46:2)]_C18:2	792.70807 -> 495.40807
[TG(46:1)]_C14:0	794.72377 -> 549.52377
[TG(49:7)]_C18:1	824.77067 -> 525.47067
[TG(54:5)]_C18:0	898.78637 -> 597.48637
PC(16:0),PC(O-17:0),LPC(O-18:0)	510.392325 -> 184.1
Cer(d18:1/22:0)	622.613825 -> 264.4
PC(42:6)	862.632625 -> 184.1

PC(34:6)	750.507425 -> 184.1
PI(38:3)	906.5728 -> 629.5728
[TG(42:0)]_C16:0	740.67677 -> 467.37677
[TG(50:9),TG(49:2)]_C18:1	834.75507 -> 535.45507
[TG(49:8),TG(48:1)]_C18:0	822.75507 -> 521.45507
[TG(52:8),TG(51:1)]_C18:0	864.80197 -> 563.50197
[TG(55:7),TG(54:0)]_C20:0	908.86457 -> 579.56457
PI(36:2),PI(O-37:2),PI(P-37:1)	880.5935 -> 603.5935
[TG(53:8),TG(52:1)]_C20:0	878.81767 -> 549.51767
[TG(50:5)]_C18:2	842.72377 -> 545.42377
PC(44:10),PC(O-44:3)	882.731525 -> 184.1
[TG(46:1)]_C16:1	794.72377 -> 523.42377
CE(22:6)Na	719.57427 -> 369.2
[TG(46:2)]_C16:0	792.70807 -> 519.40807
[TG(44:1)]_C16:0	766.69247 -> 493.39247
CAR(14:1)	370.295725 -> 85.1
[TG(52:7),TG(51:0)]_C16:0	866.81767 -> 593.51767
[TG(57:10),TG(56:3)]_C18:0	930.84897 -> 629.54897
PC(36:6)	778.538725 -> 184.1
PC(19:1),LPC(20:1),PC(O-20:1),PC(P-20:0)	550.387225 -> 184.1
SM(d18:1/12:0)	647.512825 -> 184.1
PC(32:3),PC(P-33:2)	728.559425 -> 184.1
[TG(58:8)]_C22:5	948.89587 -> 601.59587
Cer(d18:1/24:1(15Z))	648.629425 -> 264.4
[TG(46:0)]_C18:0	796.73937 -> 495.43937
[TG(56:9),TG(55:2)]_C18:1	918.84897 -> 619.54897
[TG(52:7),TG(51:0)]_C18:0	866.81767 -> 565.51767
PC(29:2),PC(P-30:1)	688.528125 -> 184.1
[TG(57:9),TG(56:2)]_C18:0	932.86457 -> 631.56457
[TG(49:8)]_C18:2	822.75507 -> 525.45507
[TG(52:6)]_C18:2	868.73937 -> 571.43937
[TG(57:11),TG(56:4)]_C20:0	928.83327 -> 599.53327
[TG(53:7),TG(52:0)]_C20:0	880.83327 -> 551.53327
[TG(57:8),TG(56:1)]_C20:0	934.88027 -> 605.58027
PE(36:3),PE(P-37:2)	742.575025 -> 601.575025
1-O-tricosanoyl-Cer(d18:1/16:0)	874.859125 -> 264.4
SM(d17:0/27:0)	845.747525 -> 184.1
[TG(58:7)]_C18:1	950.91157 -> 651.61157
[TG(38:1)]_C18:1	682.59857 -> 383.29857
[TG(53:8),TG(52:1)]_C16:1	878.81767 -> 607.51767
[TG(56:12),TG(55:5)]_C18:2	912.80197 -> 615.50197
[TG(44:1)]_C18:1	766.69247 -> 467.39247
[TG(55:7)]_C18:1	908.86457 -> 609.56457
[TG(54:7),TG(53:0)]_C18:0	894.84897 -> 593.54897
[TG(51:6)]_C16:0	854.72377 -> 581.42377
[TG(54:5)]_C16:1	898.78637 -> 627.48637
[TG(48:3)]_C14:0	818.72377 -> 573.52377
[TG(49:7)]_C16:1	824.77067 -> 553.47067
PC(44:0)	902.757825 -> 184.1
PE(40:6),PE(dO-40:0)	792.684625 -> 651.684625
[TG(52:9),TG(51:2)]_C16:1	862.78637 -> 591.48637
PE(36:4),PE(O-37:4)	740.559425 -> 599.559425

[TG(52:6)]_C16:0	868.73937 -> 595.43937
[TG(59:13),TG(58:6)]_C18:1	952.83327 -> 653.53327
[TG(40:0)]_C16:0	712.64547 -> 439.34547
[TG(38:0)]_C18:0	684.61417 -> 383.31417
[TG(52:6)]_C16:1	868.73937 -> 597.43937
PC(42:1)	872.710825 -> 184.1
[TG(58:8),TG(57:1)]_C18:1	948.89587 -> 649.59587
CAR(14:2)	368.280125 -> 85.1
[TG(50:7),TG(49:0)]_C18:0	838.78637 -> 537.48637
[TG(44:2)]_C16:0	764.67677 -> 491.37677
SM(d18:2/15:0)	687.544125 -> 184.1
[TG(48:4)]_C18:2	816.70807 -> 519.40807
[TG(49:3)]_C18:2	832.73937 -> 535.43937
PC(24:0)	622.444825 -> 184.1
[TG(54:12),TG(53:5)]_C18:2	884.77067 -> 587.47067
[TG(51:6)]_C18:0	854.72377 -> 553.42377
[TG(44:0),TG(O-45:0)]_C18:0	768.74447 -> 467.44447
[TG(56:8),TG(55:1)]_C18:1	920.86457 -> 621.56457
[TG(46:3)]_C18:1	790.69247 -> 491.39247
[TG(55:11),TG(54:4)]_C16:1	900.80197 -> 629.50197
[TG(48:7),TG(47:0)]_C16:0	810.75507 -> 537.45507
[TG(44:0),TG(O-45:0)]_C14:0	768.74447 -> 523.54447
LPC(20:3)	546.355925 -> 184.1
[TG(55:10),TG(54:3)]_C20:0	902.81767 -> 573.51767
Cer(d18:1/16:0)	538.519925 -> 264.4
LPE(22:4)	530.324625 -> 389.324625
CE(20:1)H	679.639325 -> 369.2
DG(30:2)_C16:0	554.47847 -> 281.17847
DG(30:3)_C16:1	552.46277 -> 281.16277
DG(36:8),DG(35:1)_C16:1	626.57237 -> 355.27237
[TG(42:0)]_C14:0	740.67677 -> 495.47677
CAR(20:0)	456.405325 -> 85.1
DG(36:7),DG(35:0)_C16:0	628.58797 -> 355.28797
DG(36:6)_C16:0	630.50977 -> 357.20977
DG(41:5)_C16:0	702.60367 -> 429.30367
DG(42:11),DG(41:4)_C16:0	704.61927 -> 431.31927
DG(30:2)_C16:1	554.47847 -> 283.17847
DG(36:7)_C16:1	628.58797 -> 357.28797
CE(15:1) NH4	626.58757 -> 369.2
DG(41:6)_C16:1	700.58797 -> 429.28797
DG(36:8),DG(35:1)_C18:1	626.57237 -> 327.27237
Cer(d18:0/17:0)	554.551225 -> 266.4
DG(36:5)_C16:0	632.52537 -> 359.22537
DG(30:1)_C16:0	556.49407 -> 283.19407
DG(36:6)_C16:1	630.50977 -> 359.20977
DG(32:5)_C18:1	576.46277 -> 277.16277
FA(24:4)	359.294975 -> 359.294975
[TG(57:11),TG(56:4)]_C16:0	928.83327 -> 655.53327
PC(30:3)	700.491725 -> 184.1
FA(30:0)	451.451475 -> 451.451475
FA(18:3)	277.216775 -> 277.216775
DG(34:1)_C16:1	612.55667 -> 341.25667

LPI(19:0),LPI(O-20:0)	632.3795 -> 355.3795
DG(41:6)_C18:1	700.58797 -> 401.28797
CE(15:0) NH4	628.60327 -> 369.2
[TG(48:8),TG(47:1)]_C16:1	808.73937 -> 537.43937
[TG(48:8),TG(47:1)]_C16:0	808.73937 -> 535.43937
PC(31:3),PC(O-32:3)	714.543725 -> 184.1
[TG(58:8)]_C18:2	948.89587 -> 651.59587
[TG(45:3)]_C16:0	776.67677 -> 503.37677
CE(20:1)Na	701.62127 -> 369.2
CE(16:0)H	625.592325 -> 369.2
FA(10:3)	165.091575 -> 165.091575
[TG(50:9),TG(49:2)]_C16:1	834.75507 -> 563.45507
DG(41:5)_C16:1	702.60367 -> 431.30367
CE(22:4)H	701.623625 -> 369.2
LPG(18:0); LPG(18:0)	530.34577 -> 341.34577
[TG(46:2)]_C16:1	792.70807 -> 521.40807
[TG(48:8),TG(47:1)]_C18:1	808.73937 -> 509.43937
CE(17:0)Na	661.58997 -> 369.2
PC(44:1)	900.742125 -> 184.1
[TG(46:3)]_C18:2	790.69247 -> 493.39247
[TG(58:9),TG(57:2)]_C18:1	946.88027 -> 647.58027
[TG(57:8),TG(56:1)]_C18:0	934.88027 -> 633.58027
[TG(49:3)]_C16:0	832.73937 -> 559.43937
PI(34:2),PI(O-35:2),PI(P-35:1)	852.5622 -> 575.5622
[TG(52:10),TG(51:3)]_C16:1	860.77067 -> 589.47067
CE(24:1) NH4	752.72847 -> 369.2
1-O-pentacosanoyl-Cer(d18:1/16:0)	902.890425 -> 264.4
FA(16:0)	255.232375 -> 255.232375
[TG(50:9),TG(49:2)]_C20:0	834.75507 -> 505.45507
[TG(47:6)]_C16:0	798.66117 -> 525.36117
[TG(53:9),TG(52:2)]_C20:0	876.80197 -> 547.50197
[TG(50:5)]_C18:3	842.72377 -> 547.42377
CE(24:1)Na	757.68387 -> 369.2
Cer(d14:2(4E,6E)/16:0)	480.441625 -> 206.4
FA(22:1)	337.310675 -> 337.310675
[TG(51:7),TG(50:0)]_C14:0	852.80197 -> 607.60197
[TG(42:1)]_C18:1	738.66117 -> 439.36117
PI(36:1),PI(O-37:1),PI(P-37:0)	882.6092 -> 605.6092
CAR(20:1)	454.389625 -> 85.1
[TG(50:8),TG(49:1)]_C16:1	836.77067 -> 565.47067
PG(25:0); PG(25:0)	642.43457 -> 453.43457
[TG(45:0)]_C16:0	782.72377 -> 509.42377
[TG(57:8),TG(56:1)]_C18:1	934.88027 -> 635.58027
PG(O-35:1),PG(P-35:0); PG(O-35:1),PG(P-35:0)	766.59617 -> 577.59617
[TG(54:7)]_C18:1	894.84897 -> 595.54897
PE(37:5),PE(O-38:5),PE(P-38:4)	752.559425 -> 611.559425
PC(44:3)	896.710825 -> 184.1
PE(38:3)	770.570025 -> 629.570025
FA(22:0)	339.326275 -> 339.326275
FA(37:0)	549.561075 -> 549.561075
CAR(18:3)	422.327025 -> 85.1
FA(34:6)	495.420175 -> 495.420175

[TG(42:0)]_C18:0	740.67677 -> 439.37677
FA(19:0)	297.279375 -> 297.279375
FA(26:6)	383.294975 -> 383.294975
FA(23:0)	353.341975 -> 353.341975
[TG(55:7),TG(54:0)]_C16:0	908.86457 -> 635.56457
CE(20:0)K	719.610808 -> 369.2
PC(44:2)	898.726525 -> 184.1
[TG(59:10),TG(58:3)]_C18:2	958.88027 -> 661.58027
[TG(44:2)]_C18:2	764.67677 -> 467.37677
[TG(50:8),TG(49:1)]_C14:0	836.77067 -> 591.57067
[TG(52:5)]_C22:5	870.75507 -> 523.45507
PI(38:5)	902.5415 -> 625.5415
[TG(52:9),TG(51:2)]_C18:0	862.78637 -> 561.48637
[TG(52:7)]_C18:1	866.81767 -> 567.51767
LPC(14:0),PC(O-14:0),LPC(O-15:0)	468.345425 -> 184.1
[TG(45:2)]_C16:0	778.69247 -> 505.39247
FA(21:1)	323.294975 -> 323.294975
CAR(18:2)	424.342725 -> 85.1
[TG(45:1)]_C16:1	780.70807 -> 509.40807
FA(26:2)	391.357575 -> 391.357575
Cer(d18:0/21:0)	610.613825 -> 266.4
DG(41:6)_C16:0	700.58797 -> 427.28797
LPS(P-16:0)	482.288325 -> 297.288325
[TG(45:4)]_C20:0	774.66117 -> 445.36117
DG(30:1)_C16:1	556.49407 -> 285.19407
DG(41:5)_C18:0	702.60367 -> 401.30367
DG(35:3)_C18:0	622.54107 -> 321.24107
FA(34:0)	507.514075 -> 507.514075
[TG(47:2)]_C16:1	806.72377 -> 535.42377
[TG(44:1)]_C14:0	766.69247 -> 521.49247
[TG(51:7)]_C16:1	852.80197 -> 581.50197
[TG(38:0)]_C20:0	684.61417 -> 355.31417
[TG(57:9),TG(56:2)]_C18:2	932.86457 -> 635.56457
[TG(57:8),TG(56:1)]_C16:0	934.88027 -> 661.58027
[TG(57:9),TG(56:2)]_C16:0	932.86457 -> 659.56457
PC(19:0),LPC(20:0),PC(O-20:0)	552.402925 -> 184.1
[TG(46:1)]_C18:0	794.72377 -> 493.42377
[TG(48:7),TG(47:0)]_C14:0	810.75507 -> 565.55507
[TG(56:8)]_C18:3	920.86457 -> 625.56457
[TG(48:4)]_C18:1	816.70807 -> 517.40807
[TG(48:6)]_C16:0	812.67677 -> 539.37677
CE(14:1) NH4	612.57197 -> 369.2
[TG(48:4)]_C18:3	816.70807 -> 521.40807
[TG(39:0)]_C16:0	698.62987 -> 425.32987
[TG(56:13),TG(55:6)]_C18:2	910.78637 -> 613.48637
FA(38:6)	551.482775 -> 551.482775
[TG(48:6)]_C18:0	812.67677 -> 511.37677
PI(38:2),PI(P-39:1)	908.6248 -> 631.6248
DG(36:8),DG(35:1)_C18:0	626.57237 -> 325.27237
PC(44:7),PC(43:0)	888.742125 -> 184.1
[TG(37:0)]_C14:0	670.59857 -> 425.39857
[TG(55:10),TG(54:3)]_C16:1	902.81767 -> 631.51767

FA(10:5)	161.060275 -> 161.060275
FA(35:0)	521.529775 -> 521.529775
[TG(53:10),TG(52:3)]_C20:0	874.78637 -> 545.48637
PS(25:0)	638.403325 -> 453.403325
FA(28:0)	423.420175 -> 423.420175
FA(32:0)	479.482775 -> 479.482775
[TG(58:9)]_C20:4	946.88027 -> 625.58027
[TG(54:6)]_C16:1	896.77067 -> 625.47067
PC(44:4)	894.695225 -> 184.1
FA(18:2)	279.232375 -> 279.232375
FA(28:6)	411.326275 -> 411.326275
FA(21:2)	321.279375 -> 321.279375
FA(30:2)	447.420175 -> 447.420175
CAR(26:0)	540.499225 -> 85.1
DG(37:7)_C18:1	642.60367 -> 343.30367
DG(30:2)_C18:1	554.47847 -> 255.17847
FA(24:0)	367.357575 -> 367.357575
FA(24:2)	363.326275 -> 363.326275
FA(20:6)	299.201075 -> 299.201075
FA(24:1)	365.341975 -> 365.341975
PG(24:0); PG(24:0)	628.41897 -> 439.41897
[TG(40:0)]_C18:0	712.64547 -> 411.34547
LPC(22:6)	568.340325 -> 184.1
PC(26:1)	648.460425 -> 184.1
CE(24:1)K	773.657808 -> 369.2
[TG(44:5)]_C20:0	758.62987 -> 429.32987
[TG(59:10),TG(58:3)]_C18:1	958.88027 -> 659.58027
[TG(46:2)]_C14:0	792.70807 -> 547.50807
[TG(50:4)]_C20:4	844.73937 -> 523.43937
PG(32:0),PG(O-33:0); PG(32:0),PG(O-33:0)	740.58057 -> 551.58057
[TG(44:2)]_C18:1	764.67677 -> 465.37677
[TG(59:9),TG(58:2)]_C18:2	960.89587 -> 663.59587
PI(36:4)	876.5258 -> 599.5258
[TG(52:4)]_C18:0	872.77067 -> 571.47067
[TG(53:9),TG(52:2)]_C14:0	876.80197 -> 631.60197
PC(44:8),PC(43:1)	886.726525 -> 184.1
[TG(38:1)]_C16:0	682.59857 -> 409.29857
DG(40:7),DG(39:0)_C18:0	684.65057 -> 383.35057
LPG(15:0),LPG(O-16:0); LPG(15:0),LPG(O-16:0)	488.33527 -> 299.33527
[TG(46:6)]_C16:0	784.64547 -> 511.34547
FA(22:5)	329.248075 -> 329.248075
[TG(51:8)]_C22:5	850.78637 -> 503.48637
FA(32:5)	469.404575 -> 469.404575
FA(30:4)	443.388875 -> 443.388875
FA(10:2)	167.107175 -> 167.107175
FA(23:1)	351.326275 -> 351.326275
FA(20:2)	307.263675 -> 307.263675
CAR(22:5)	474.358325 -> 85.1
[TG(62:14),TG(61:7),TG(60:0)]_C20:0	992.95847 -> 663.65847
CAR(20:2)	452.374025 -> 85.1
[TG(37:0)]_C16:0	670.59857 -> 397.29857
DG(32:1)_C18:1	584.52537 -> 285.22537

DG(33:1),DG(O-34:1)_C16:1	598.57747 -> 327.27747
DG(33:1),DG(O-34:1)_C18:1	598.57747 -> 299.27747
DG(36:7)_C18:1	628.58797 -> 329.28797
FA(19:1)	295.263675 -> 295.263675
LPI(16:0)	590.2962 -> 313.2962
[TG(41:0)]_C16:0	726.66117 -> 453.36117
LPE(18:1)	480.309025 -> 339.309025
FA(25:0)	381.373275 -> 381.373275
FA(6:2)	111.044575 -> 111.044575
FA(33:0)	493.498475 -> 493.498475
DG(30:1)_C18:1	556.49407 -> 257.19407
CAR(18:0)	428.374025 -> 85.1
LPG(16:0); LPG(16:0)	502.31447 -> 313.31447
FA(21:5)	315.232375 -> 315.232375
FA(22:6)	327.232375 -> 327.232375
FA(26:4)	387.326275 -> 387.326275
FA(19:6)	285.185475 -> 285.185475
DG(24:0)_C18:0	474.41587 -> 173.11587
[TG(57:8)]_C18:2	934.88027 -> 637.58027
FA(20:1)	309.279375 -> 309.279375
FA(18:1)	281.248075 -> 281.248075
CAR(22:0)	484.436625 -> 85.1
CAR(5:1)	244.154925 -> 85.1
FA(22:2)	335.294975 -> 335.294975
CAR(10:3)	310.201825 -> 85.1
FA(36:6)	523.451475 -> 523.451475
[TG(43:1)]_C16:1	752.67677 -> 481.37677
FA(16:6)	243.138475 -> 243.138475
[TG(46:3)]_C16:1	790.69247 -> 519.39247
[TG(58:14),TG(57:7),TG(56:0)]_C16:0	936.89587 -> 663.59587
FA(27:3)	403.357575 -> 403.357575
FA(36:4)	527.482775 -> 527.482775
FA(19:5)	287.201075 -> 287.201075
FA(24:5)	357.279375 -> 357.279375
CAR(14:0)	372.311425 -> 85.1
[TG(45:2)]_C16:1	778.69247 -> 507.39247
DG(33:3)_C16:0	594.50977 -> 321.20977
FA(9:0)	157.122875 -> 157.122875
FA(14:6)	215.107175 -> 215.107175
FA(18:6)	271.169775 -> 271.169775
FA(18:5)	273.185475 -> 273.185475
FA(20:3)	305.248075 -> 305.248075
PE(32:1),PE(O-33:1),PE(P-33:0)	690.543725 -> 549.543725
DG(38:7),DG(37:0)_C16:0	656.61927 -> 383.31927
DG(32:1)_C16:1	584.52537 -> 313.22537
	709.686225 -> 369.2
CE(24:1)H	735.701925 -> 369.2
CE(22:0)K	747.642108 -> 369.2
LPE(22:6)	526.293325 -> 385.293325
PC(44:5)	892.679525 -> 184.1
CE(12:0) NH4	586.55627 -> 369.2
DG(40:6),DG(dO-40:0)_C16:0	686.70267 -> 413.40267

CE(17:0) NH4	656.63457 -> 369.2
PE(37:6),PE(O-38:6),PE(P-38:5)	750.543725 -> 609.543725
DG(44:9),DG(43:2)_C18:1	736.68187 -> 437.38187
[TG(58:8)]_C20:4	948.89587 -> 627.59587
DG(44:7),DG(43:0)_C16:0	740.71317 -> 467.41317
CE(18:2)H	649.592325 -> 369.2
DG(44:8),DG(43:1)_C18:1	738.69757 -> 439.39757
CE(22:6)K	735.548208 -> 369.2
DG(34:3)_C16:0	608.52537 -> 335.22537
PE(40:5)	794.570025 -> 653.570025
DG(40:7),DG(39:0)_C16:0	684.65057 -> 411.35057
PE(35:4),PE(O-36:4),PE(P-36:3)	726.543725 -> 585.543725
DG(33:2)_C18:1	596.52537 -> 297.22537
CAR(17:0)	414.358325 -> 85.1
[TG(44:3)]_C20:0	762.66117 -> 433.36117
FA(14:5)	217.122875 -> 217.122875
LPE(18:2),LPE(P-19:1)	478.329725 -> 337.329725
[TG(40:1)]_C18:1	710.62987 -> 411.32987
PC(43:2)	884.710825 -> 184.1
DG(28:2)_C18:1	526.44717 -> 227.14717
DG(28:1)_C18:1	528.46277 -> 229.16277
DG(31:1)_C18:1	570.50977 -> 271.20977
DG(36:4),DG(O-37:4)_C18:1	634.57747 -> 335.27747
CAR(22:2)	480.405325 -> 85.1
[TG(43:1)]_C18:1	752.67677 -> 453.37677
[TG(38:1)]_C14:0	682.59857 -> 437.39857
FA(40:6)	579.514075 -> 579.514075
DG(42:6)_C16:0	714.60367 -> 441.30367
FA(3:0)	73.028975 -> 73.028975
[TG(43:1)]_C16:0	752.67677 -> 479.37677
FA(27:0)	409.404575 -> 409.404575
DG(44:7),DG(43:0)_C18:0	740.71317 -> 439.41317
DG(42:7),DG(41:0)_C16:0	712.68187 -> 439.38187
FA(28:1)	421.404575 -> 421.404575
FA(26:0)	395.388875 -> 395.388875
DG(38:6),DG(dO-40:6)_C16:0	658.61377 -> 385.31377
FA(34:1)	505.498475 -> 505.498475
[TG(55:7),TG(54:0)]_C14:0	908.86457 -> 663.66457
[TG(59:9),TG(58:2)]_C18:1	960.89587 -> 661.59587
[TG(48:3)]_C18:3	818.72377 -> 523.42377
[TG(44:1)]_C16:1	766.69247 -> 495.39247
[TG(48:8),TG(47:1)]_C14:0	808.73937 -> 563.53937
[TG(62:16),TG(61:9),TG(60:2)]_C18:1	988.92717 -> 689.62717
[TG(60:15),TG(59:8),TG(58:1)]_C18:1	962.91157 -> 663.61157
[TG(61:10),TG(60:3)]_C18:1	986.91157 -> 687.61157
[TG(52:4)]_C14:0	872.77067 -> 627.57067
[TG(47:2)]_C18:2	806.72377 -> 509.42377
[TG(50:5)]_C20:4	842.72377 -> 521.42377
[TG(42:1)]_C16:0	738.66117 -> 465.36117
PC(14:0),LPC(15:0),LPC(O-16:0)	482.361025 -> 184.1
[TG(59:11),TG(58:4)]_C18:2	956.86457 -> 659.56457
[TG(50:7)]_C18:1	838.78637 -> 539.48637

[TG(45:0)]_C14:0	782.72377 -> 537.52377
LPC(15:1),LPC(O-16:1),LPC(P-16:0)	480.345425 -> 184.1
[TG(58:10)]_C20:4	944.86457 -> 623.56457
[TG(50:9),TG(49:2)]_C14:0	834.75507 -> 589.55507
PI(36:3),PI(P-37:2)	878.5779 -> 601.5779
[TG(47:6)]_C14:0	798.66117 -> 553.46117
[TG(47:2)]_C16:0	806.72377 -> 533.42377
[TG(59:9),TG(58:2)]_C16:0	960.89587 -> 687.59587
[TG(43:0)]_C16:0	754.69247 -> 481.39247
[TG(46:3)]_C14:0	790.69247 -> 545.49247
[TG(51:8),TG(50:1)]_C20:0	850.78637 -> 521.48637
[TG(42:2)]_C18:2	736.64547 -> 439.34547
[TG(42:1)]_C14:0	738.66117 -> 493.46117
[TG(45:1)]_C16:0	780.70807 -> 507.40807
[TG(44:2)]_C16:1	764.67677 -> 493.37677
[TG(44:2)]_C14:0	764.67677 -> 519.47677
FA(36:5)	525.467175 -> 525.467175
PG(O-37:2),PG(P-37:1); PG(O-37:2),PG(P-37:1)	792.61187 -> 603.61187
[TG(47:2)]_C14:0	806.72377 -> 561.52377
[TG(49:3)]_C18:1	832.73937 -> 533.43937
[TG(45:1)]_C18:1	780.70807 -> 481.40807
[TG(47:2)]_C18:1	806.72377 -> 507.42377
[TG(52:6)]_C14:0	868.73937 -> 623.53937
[TG(54:7)]_C16:1	894.84897 -> 623.54897
DG(32:2)_C18:2	582.50977 -> 285.20977
LPC(17:1),LPC(O-18:1),LPC(P-18:0)	508.376725 -> 184.1
[TG(60:15),TG(59:8),TG(58:1)]_C16:0	962.91157 -> 689.61157
CAR(16:0)	400.342725 -> 85.1
PG(36:3),PG(P-37:2); PG(36:3),PG(P-37:2)	790.59617 -> 601.59617
PC(50:0)	986.851725 -> 184.1
[TG(40:0)]_C14:0	712.64547 -> 467.44547
[TG(47:6)]_C18:0	798.66117 -> 497.36117
DG(44:1)_C18:1	752.71317 -> 453.41317
FA(31:0)	465.467175 -> 465.467175
PI(34:1),PI(O-35:1),PI(P-35:0)	854.5779 -> 577.5779
[TG(49:3)]_C16:1	832.73937 -> 561.43937
DG(38:8),DG(dO-40:8),DG(37:1)_C18:1	654.60367 -> 355.30367
[TG(38:0)]_C16:0	684.61417 -> 411.31417
[TG(45:4)]_C18:1	774.66117 -> 475.36117
[TG(50:9)]_C22:6	834.75507 -> 489.45507
[TG(40:1)]_C16:0	710.62987 -> 437.32987
[TG(60:14),TG(59:7),TG(58:0)]_C18:0	964.92717 -> 663.62717
PG(30:0),PG(O-31:0); PG(30:0),PG(O-31:0)	712.54927 -> 523.54927
[TG(54:12),TG(53:5)]_C22:5	884.77067 -> 537.47067
DG(41:7),DG(40:0)_C16:0	698.66627 -> 425.36627
[TG(44:4)]_C20:0	760.64547 -> 431.34547
FA(28:3)	417.373275 -> 417.373275
[TG(49:3)]_C20:0	832.73937 -> 503.43937
[TG(45:4)]_C16:1	774.66117 -> 503.36117
FA(25:4)	373.310675 -> 373.310675
FA(30:3)	445.404575 -> 445.404575
CE(50:3;O2)H	1128.067325 -> 369.2

PG(26:0); PG(26:0)	656.45027 -> 467.45027
PC(44:6)	890.663925 -> 184.1
[TG(40:1)]_C14:0	710.62987 -> 465.42987
[TG(42:1)]_C16:1	738.66117 -> 467.36117
[TG(45:1)]_C14:0	780.70807 -> 535.50807
[TG(55:7)]_C22:6	908.86457 -> 563.56457
CE(46:3;O2) NH4	1089.03127 -> 369.2
FA(12:6)	187.075875 -> 187.075875
FA(17:1)	267.232375 -> 267.232375
FA(24:3)	361.310675 -> 361.310675
FA(26:3)	389.341975 -> 389.341975
FA(38:4)	555.514075 -> 555.514075
FA(5:1)	99.044575 -> 99.044575
DG(29:2)_C16:0	540.46277 -> 267.16277
PG(28:0),PG(O-29:0); PG(28:0),PG(O-29:0)	684.51797 -> 495.51797
FA(15:5)	231.138475 -> 231.138475
FA(17:6)	257.154175 -> 257.154175
FA(26:5)	385.310675 -> 385.310675
[TG(57:8),TG(56:1)]_C16:1	934.88027 -> 663.58027
LPS(O-20:0)	540.366525 -> 355.366525
LPE(12:0)	398.230725 -> 257.230725
[TG(48:5)]_C22:5	814.69247 -> 467.39247
[TG(50:5)]_C22:5	842.72377 -> 495.42377
[TG(59:12),TG(58:5)]_C22:5	954.84897 -> 607.54897
DG(34:5)_C16:1	604.49407 -> 333.19407
DG(42:0)_C16:0	726.69757 -> 453.39757
FA(27:2)	405.373275 -> 405.373275
DG(44:8),DG(43:1)_C16:0	738.69757 -> 465.39757
CE(16:2) NH4	638.58757 -> 369.2
CE(16:1)K	661.532608 -> 369.2
LPC(22:5)	570.355925 -> 184.1
DG(40:8),DG(39:1)_C18:1	682.63497 -> 383.33497
CE(18:0)H	653.623625 -> 369.2
CE(20:4)K	711.548208 -> 369.2
DG(31:1)_C16:0	570.50977 -> 297.20977
CE(22:5)Na	721.58997 -> 369.2
DG(44:9),DG(43:2)_C18:2	736.68187 -> 439.38187
DG(42:8),DG(41:1)_C18:1	710.66627 -> 411.36627
[TG(37:0)]_C20:0	670.59857 -> 341.29857
PE(40:4)	796.585625 -> 655.585625
DG(30:2)_C18:2	554.47847 -> 257.17847
CE(12:0)Na	591.51167 -> 369.2
CE(22:3)Na	725.62127 -> 369.2
DG(33:3)_C18:2	594.50977 -> 297.20977
DG(44:0)_C16:0	754.72887 -> 481.42887
FA(14:1)	225.185475 -> 225.185475
DG(38:7),DG(37:0)_C18:0	656.61927 -> 355.31927
PC(18:0),LPC(19:0),PC(O-19:0),LPC(O-20:0)	538.423625 -> 184.1
PE(39:6),PE(O-40:6),PE(P-40:5)	778.575025 -> 637.575025
[TG(45:0)]_C18:0	782.72377 -> 481.42377
[TG(51:4)]_C16:1	858.75507 -> 587.45507
[TG(52:9),TG(51:2)]_C14:0	862.78637 -> 617.58637

DG(26:0)_C16:0	502.44717 -> 229.14717
CE(16:3) NH4	636.57197 -> 369.2
CE(22:5)K	737.563908 -> 369.2
SM(d16:0/14:0)	649.528425 -> 184.1
CE(20:3)K	713.563908 -> 369.2
CE(22:0) NH4	726.71277 -> 369.2
FA(26:1)	393.373275 -> 393.373275
DG(33:1),DG(O-34:1)_C16:0	598.57747 -> 325.27747
DG(39:7),DG(38:0),DG(dO-40:0)_C16:0	670.70767 -> 397.40767
[TG(44:4)]_C18:1	760.64547 -> 461.34547
[TG(45:3)]_C20:0	776.67677 -> 447.37677
[TG(51:9)]_C22:6	848.77067 -> 503.47067
DG(32:2)_C18:0	582.50977 -> 281.20977
FA(29:2)	433.404575 -> 433.404575
[TG(39:0)]_C18:0	698.62987 -> 397.32987
[TG(44:3)]_C16:1	762.66117 -> 491.36117
DG(36:8),DG(35:1)_C16:0	626.57237 -> 353.27237
DG(38:9),DG(dO-40:9),DG(37:2)_C18:2	652.58797 -> 355.28797
FA(16:1)	253.216775 -> 253.216775
CAR(10:1)	314.233125 -> 85.1
CAR(20:4)	448.342725 -> 85.1
[TG(44:5)]_C18:2	758.62987 -> 461.32987
FA(11:0)	185.154175 -> 185.154175
FA(29:0)	437.435875 -> 437.435875
CAR	162.2 -> 85.1
CAR(5:0)	246.170525 -> 85.1
CAR(7:0)	274.201825 -> 85.1
CAR(8:1)	286.201825 -> 85.1
CAR(8:0)	288.217525 -> 85.1
CAR(9:0)	302.233125 -> 85.1
CAR(10:0)	316.248825 -> 85.1
CAR(11:0)	330.264425 -> 85.1
CAR(12:0)	344.280125 -> 85.1
CAR(16:2)	396.311425 -> 85.1
CAR(16:1)	398.327025 -> 85.1
CAR(18:4)	420.311425 -> 85.1
CAR(18:1)	426.358325 -> 85.1
LPC(12:0)	440.277725 -> 184.1
LPG(12:0); LPG(12:0)	446.25187 -> 257.25187
CAR(22:6)	472.400225 -> 85.1
LPG(14:0); LPG(14:0)	474.28317 -> 285.28317
LPE(18:3)	476.277725 -> 335.277725
CAR(22:4)	476.374025 -> 85.1
LPC(16:1),LPC(P-17:0)	494.361025 -> 184.1
LPE(20:5)	500.277725 -> 359.277725
LPE(20:3)	504.309025 -> 363.309025
LPC(18:3)	518.324625 -> 184.1
LPE(22:5)	528.309025 -> 387.309025
LPG(18:1); LPG(18:1)	528.33017 -> 339.33017
PC(18:1),LPC(19:1),PC(O-19:1),LPC(P-20:0)	536.408025 -> 184.1
LPC(20:5)	542.324625 -> 184.1
LPS(22:6)	570.283225 -> 385.283225

LPG(22:6); LPG(22:6)	574.31447 -> 385.31447
LPG(22:0); LPG(22:0)	586.40837 -> 397.40837
LPI(19:1),LPI(P-20:0)	630.3639 -> 353.3639
PG(26:1); PG(26:1)	654.43457 -> 465.43457
SM(d18:0/13:0)	663.544125 -> 184.1
Cer(d18:1/25:0)	664.660725 -> 264.4
PG(28:1),PG(P-29:0); PG(28:1),PG(P-29:0)	682.50227 -> 493.50227
PG(29:0),PG(O-30:0); PG(29:0),PG(O-30:0)	698.53357 -> 509.53357
PG(30:1),PG(O-31:1),PG(P-31:0); PG(30:1),PG(O-31:1),PG(P-31:0)	710.53357 -> 521.53357
PE(34:4)	712.491725 -> 571.491725
PG(31:0),PG(O-32:0); PG(31:0),PG(O-32:0)	726.56487 -> 537.56487
PG(32:2),PG(O-33:2),PG(P-33:1); PG(32:2),PG(O-33:2),PG(P-33:1)	736.54927 -> 547.54927
PC(33:5),PC(P-34:4)	738.543725 -> 184.1
PG(32:1),PG(O-33:1),PG(P-33:0); PG(32:1),PG(O-33:1),PG(P-33:0)	738.56487 -> 549.56487
PC(33:4),PC(O-34:4),PC(O-34:4),PC(P-34:3)	740.559425 -> 184.1
PC(34:5)	752.523025 -> 184.1
PG(33:1),PG(O-34:1),PG(P-34:0); PG(33:1),PG(O-34:1),PG(P-34:0)	752.58057 -> 563.58057
PC(34:4),PC(O-35:4)	754.575025 -> 184.1
PE(37:4),PE(O-38:4),PE(P-38:3)	754.575025 -> 613.575025
PG(33:0),PG(O-34:0); PG(33:0),PG(O-34:0)	754.59617 -> 565.59617
PG(34:0),PG(O-35:0); PG(34:0),PG(O-35:0)	768.61187 -> 579.61187
PG(35:6),PG(P-36:5); PG(35:6),PG(P-36:5)	770.53357 -> 581.53357
PG(36:1),PG(O-37:1),PG(P-37:0); PG(36:1),PG(O-37:1),PG(P-37:0)	794.62747 -> 605.62747
PG(37:7),PG(P-38:6),PG(36:0),PG(O-37:0); PG(37:7),PG(P-38:6),PG(36:0),PG(O-37:0)	796.64317 -> 607.64317
PE(40:3)	798.601325 -> 657.601325
PE(42:5)	822.601325 -> 681.601325
PS(40:4)	840.575425 -> 655.575425
PE(44:8),PE(43:1)	844.679525 -> 703.679525
PE(44:7),PE(43:0)	846.695225 -> 705.695225
PS(O-42:0)	862.690125 -> 677.690125
PS(42:5)	866.591125 -> 681.591125
PS(43:1)	888.669325 -> 703.669325
PS(43:0)	890.685025 -> 705.685025
PS(44:6)	892.606725 -> 707.606725
PI(40:6)	928.5571 -> 651.5571
PI(40:5)	930.5728 -> 653.5728
[TG(63:13),TG(62:6)]_C22:6	1008.89587 -> 663.59587
[TG(38:1)]_C16:1	682.59857 -> 411.29857
[TG(39:1)]_C18:1	696.61417 -> 397.31417
[TG(39:1)]_C16:0	696.61417 -> 423.31417
[TG(39:1)]_C16:1	696.61417 -> 425.31417
[TG(39:1)]_C14:0	696.61417 -> 451.41417
[TG(39:0)]_C14:0	698.62987 -> 453.42987
[TG(40:2)]_C18:1	708.61417 -> 409.31417
[TG(40:2)]_C16:1	708.61417 -> 437.31417
[TG(40:1)]_C18:0	710.62987 -> 409.32987
[TG(40:1)]_C16:1	710.62987 -> 439.32987
[TG(41:2)]_C18:1	722.62987 -> 423.32987
[TG(41:1)]_C18:1	724.64547 -> 425.34547
[TG(41:1)]_C16:0	724.64547 -> 451.34547
[TG(41:1)]_C16:1	724.64547 -> 453.34547
[TG(41:1)]_C14:0	724.64547 -> 479.44547

[TG(41:0)]_C18:0	726.66117 -> 425.36117
[TG(41:0)]_C14:0	726.66117 -> 481.46117
[TG(42:2)]_C16:1	736.64547 -> 465.34547
[TG(42:2)]_C14:0	736.64547 -> 491.44547
[TG(43:2)]_C16:1	750.66117 -> 479.36117
[TG(43:1)]_C14:0	752.67677 -> 507.47677
[TG(43:0)]_C18:0	754.69247 -> 453.39247
[TG(43:0)]_C14:0	754.69247 -> 509.49247
[TG(44:5)]_C22:5	758.62987 -> 411.32987
[TG(44:4)]_C16:1	760.64547 -> 489.34547
[TG(45:3)]_C16:1	776.67677 -> 505.37677
[TG(45:2)]_C18:1	778.69247 -> 479.39247
[TG(45:2)]_C18:2	778.69247 -> 481.39247
[TG(45:2)]_C14:0	778.69247 -> 533.49247
[TG(45:1)]_C18:0	780.70807 -> 479.40807
[TG(46:6)]_C18:0	784.64547 -> 483.34547
[TG(46:6)]_C14:0	784.64547 -> 539.44547
[TG(47:3)]_C18:2	804.70807 -> 507.40807
[TG(47:3)]_C16:1	804.70807 -> 533.40807
[TG(48:8),TG(47:1)]_C18:0	808.73937 -> 507.43937
[TG(48:7),TG(47:0)]_C18:0	810.75507 -> 509.45507
[TG(48:7)]_C16:1	810.75507 -> 539.45507
[TG(48:6)]_C14:0	812.67677 -> 567.47677
[TG(48:4)]_C16:1	816.70807 -> 545.40807
[TG(49:6)]_C14:0	826.69247 -> 581.49247
[TG(49:4)]_C16:1	830.72377 -> 559.42377
[TG(50:9),TG(49:2)]_C18:0	834.75507 -> 533.45507
[TG(50:8),TG(49:1)]_C20:0	836.77067 -> 507.47067
[TG(50:8),TG(49:1)]_C18:0	836.77067 -> 535.47067
[TG(50:7)]_C16:1	838.78637 -> 567.48637
[TG(50:7),TG(49:0)]_C14:0	838.78637 -> 593.58637
[TG(50:6)]_C16:0	840.70807 -> 567.40807
[TG(51:7)]_C22:5	852.80197 -> 505.50197
[TG(52:8),TG(51:1)]_C16:1	864.80197 -> 593.50197
[TG(52:8),TG(51:1)]_C14:0	864.80197 -> 619.60197
[TG(52:7),TG(51:0)]_C14:0	866.81767 -> 621.61767
[TG(53:8),TG(52:1)]_C14:0	878.81767 -> 633.61767
[TG(53:7)]_C16:1	880.83327 -> 609.53327
[TG(53:7),TG(52:0)]_C14:0	880.83327 -> 635.63327
[TG(53:6)]_C16:0	882.75507 -> 609.45507
[TG(54:11),TG(53:4)]_C16:1	886.78637 -> 615.48637
[TG(54:10),TG(53:3)]_C16:1	888.80197 -> 617.50197
[TG(54:9),TG(53:2)]_C16:1	890.81767 -> 619.51767
[TG(54:8),TG(53:1)]_C16:1	892.83327 -> 621.53327
[TG(54:7),TG(53:0)]_C14:0	894.84897 -> 649.64897
[TG(55:9),TG(54:2)]_C16:1	904.83327 -> 633.53327
[TG(55:8),TG(54:1)]_C16:1	906.84897 -> 635.54897
[TG(56:10),TG(55:3)]_C16:1	916.83327 -> 645.53327
[TG(56:9),TG(55:2)]_C16:1	918.84897 -> 647.54897
[TG(56:8),TG(55:1)]_C16:1	920.86457 -> 649.56457
[TG(56:8),TG(55:1)]_C14:0	920.86457 -> 675.66457
[TG(56:7)]_C16:1	922.88027 -> 651.58027

[TG(56:7),TG(55:0)]_C14:0	922.88027 -> 677.68027
[TG(57:12),TG(56:5)]_C16:1	926.81767 -> 655.51767
[TG(57:10),TG(56:3)]_C16:1	930.84897 -> 659.54897
[TG(57:9),TG(56:2)]_C16:1	932.86457 -> 661.56457
[TG(57:8),TG(56:1)]_C14:0	934.88027 -> 689.68027
[TG(58:14),TG(57:7),TG(56:0)]_C18:0	936.89587 -> 635.59587
[TG(58:14),TG(57:7)]_C16:1	936.89587 -> 665.59587
[TG(58:14),TG(57:7),TG(56:0)]_C14:0	936.89587 -> 691.69587
[TG(58:9),TG(57:2)]_C16:1	946.88027 -> 675.58027
[TG(58:8),TG(57:1)]_C16:0	948.89587 -> 675.59587
[TG(58:8),TG(57:1)]_C16:1	948.89587 -> 677.59587
[TG(58:8),TG(57:1)]_C14:0	948.89587 -> 703.69587
[TG(58:7),TG(57:0)]_C16:0	950.91157 -> 677.61157
[TG(59:10),TG(58:3)]_C16:1	958.88027 -> 687.58027
[TG(59:9),TG(58:2)]_C16:1	960.89587 -> 689.59587
[TG(60:15),TG(59:8),TG(58:1)]_C18:0	962.91157 -> 661.61157
[TG(60:15),TG(59:8),TG(58:1)]_C16:1	962.91157 -> 691.61157
[TG(60:15),TG(59:8),TG(58:1)]_C14:0	962.91157 -> 717.71157
[TG(60:14),TG(59:7),TG(58:0)]_C16:0	964.92717 -> 691.62717
[TG(60:12),TG(59:5)]_C18:2	968.86457 -> 671.56457
[TG(60:9),TG(59:2)]_C18:1	974.91157 -> 675.61157
[TG(60:9),TG(59:2)]_C16:1	974.91157 -> 703.61157
[TG(60:8),TG(59:1)]_C18:1	976.92717 -> 677.62717
[TG(60:8),TG(59:1)]_C16:0	976.92717 -> 703.62717
[TG(60:8),TG(59:1)]_C16:1	976.92717 -> 705.62717
[TG(61:14),TG(60:7),TG(59:0)]_C16:0	978.94287 -> 705.64287
[TG(61:13),TG(60:6)]_C20:0	980.86457 -> 651.56457
[TG(61:11),TG(60:4)]_C20:4	984.89587 -> 663.59587
[TG(61:11),TG(60:4)]_C18:0	984.89587 -> 683.59587
[TG(61:10),TG(60:3)]_C16:1	986.91157 -> 715.61157
[TG(62:15),TG(61:8),TG(60:1)]_C18:1	990.94287 -> 691.64287
[TG(62:15),TG(61:8),TG(60:1)]_C16:0	990.94287 -> 717.64287
DG(26:0)_C18:0	502.44717 -> 201.14717
DG(28:2)_C18:2	526.44717 -> 229.14717
DG(29:2)_C18:1	540.46277 -> 241.16277
DG(29:1)_C18:1	542.47847 -> 243.17847
DG(29:1)_C16:0	542.47847 -> 269.17847
DG(31:2),DG(P-14:0/18:1)_C18:1	568.53047 -> 269.23047
CE(12:0)H	569.529725 -> 369.2
DG(31:1)_C16:1	570.50977 -> 299.20977
DG(31:0)_C16:0	572.52537 -> 299.22537
DG(32:2)_C16:1	582.50977 -> 311.20977
DG(33:5)_C18:1	590.47847 -> 291.17847
DG(33:2)_C18:2	596.52537 -> 299.22537
DG(33:2)_C16:0	596.52537 -> 323.22537
DG(33:2)_C16:1	596.52537 -> 325.22537
DG(35:3)_C18:1	622.54107 -> 323.24107
DG(dO-38:9),DG(35:2)_C18:1	624.55667 -> 325.25667
DG(dO-38:9),DG(35:2)_C18:2	624.55667 -> 327.25667
DG(O-38:8),DG(36:1)_C16:0	640.58797 -> 367.28797
DG(38:9),DG(dO-40:9),DG(37:2)_C18:1	652.58797 -> 353.28797
DG(38:8),DG(dO-40:8),DG(37:1)_C16:0	654.60367 -> 381.30367

DG(38:8),DG(dO-40:8),DG(37:1)_C16:1	654.60367 -> 383.30367
DG(38:7)_C18:1	656.61927 -> 357.31927
CE(16:3)K	657.501308 -> 369.2
CE(16:2)K	659.516908 -> 369.2
DG(39:8),DG(O-40:8),DG(38:1)_C16:0	668.61927 -> 395.31927
DG(39:8),DG(O-40:8),DG(38:1)_C16:1	668.61927 -> 397.31927
	675.64 -> 369.1__1
CE(17:0)K	677.563908 -> 369.2
DG(40:9),DG(39:2)_C18:1	680.61927 -> 381.31927
DG(40:8),DG(39:1)_C16:0	682.63497 -> 409.33497
DG(40:8),DG(39:1)_C16:1	682.63497 -> 411.33497
DG(40:7)_C18:1	684.65057 -> 385.35057
DG(40:2)_C18:1	694.63497 -> 395.33497
DG(40:2)_C16:1	694.63497 -> 423.33497
DG(40:1)_C18:1	696.65057 -> 397.35057
DG(40:1)_C16:0	696.65057 -> 423.35057
DG(40:1)_C16:1	696.65057 -> 425.35057
DG(41:7)_C18:1	698.66627 -> 399.36627
DG(42:10),DG(41:3)_C18:1	706.63497 -> 407.33497
DG(42:9),DG(41:2)_C18:1	708.65057 -> 409.35057
DG(42:9),DG(41:2)_C18:2	708.65057 -> 411.35057
DG(42:8),DG(41:1)_C16:1	710.66627 -> 439.36627
DG(42:7),DG(41:0)_C18:0	712.68187 -> 411.38187
DG(42:7)_C18:1	712.68187 -> 413.38187
DG(42:2)_C18:1	722.66627 -> 423.36627
DG(42:2)_C16:1	722.66627 -> 451.36627
DG(42:1)_C18:1	724.68187 -> 425.38187
DG(42:1)_C16:0	724.68187 -> 451.38187
DG(42:1)_C16:1	724.68187 -> 453.38187
DG(42:0)_C18:0	726.69757 -> 425.39757
DG(43:6)_C16:0	728.61927 -> 455.31927
DG(44:9),DG(43:2)_C16:1	736.68187 -> 465.38187
DG(44:8),DG(43:1)_C16:1	738.69757 -> 467.39757
DG(44:7)_C18:1	740.71317 -> 441.41317
DG(44:6)_C16:0	742.63497 -> 469.33497
DG(44:2)_C18:1	750.69757 -> 451.39757
DG(44:2)_C16:1	750.69757 -> 479.39757
DG(44:1)_C16:0	752.71317 -> 479.41317
DG(44:1)_C16:1	752.71317 -> 481.41317
DG(44:0)_C18:0	754.72887 -> 453.42887
FA(7:1)	127.075875 -> 127.075875
FA(7:0)	129.091575 -> 129.091575
FA(8:6)	131.013275 -> 131.013275
FA(8:1)	141.091575 -> 141.091575
FA(8:0)	143.107175 -> 143.107175
FA(10:6)	159.044575 -> 159.044575
FA(10:1)	169.122875 -> 169.122875
FA(10:0); FA(10:0)	171.138475 -> 171.138475
FA(11:6)	173.060275 -> 173.060275
FA(12:5)	189.091575 -> 189.091575
FA(12:0)	199.169775 -> 199.169775
FA(14:7)	213.091575 -> 213.091575

FA(13:0)	213.185475 -> 213.185475
FA(14:0)	227.201075 -> 227.201075
FA(15:6)	229.122875 -> 229.122875
FA(16:5)	245.154175 -> 245.154175
FA(17:4)	261.185475 -> 261.185475
FA(18:7)	269.154175 -> 269.154175
FA(17:0)	269.248075 -> 269.248075
FA(19:4)	289.216775 -> 289.216775
FA(20:4)	303.232375 -> 303.232375
FA(21:3)	319.263675 -> 319.263675
FA(22:4)	331.263675 -> 331.263675
FA(22:3)	333.279375 -> 333.279375
FA(28:7)	409.310675 -> 409.310675
FA(28:5)	413.341975 -> 413.341975
FA(29:3)	431.388875 -> 431.388875
FA(30:5)	441.373275 -> 441.373275
FA(31:1)	463.451475 -> 463.451475
FA(32:6)	467.388875 -> 467.388875
FA(32:4)	471.420175 -> 471.420175
FA(34:5)	497.435875 -> 497.435875
FA(46:0)	675.701875 -> 675.701875
FA(4:0)	87.044575 -> 87.044575
DG(O-38:8),DG(36:1)_C16:1	640.58797 -> 369.28797
CE(16:1) NH4	640.60327 -> 369.2
CE(18:3)Na	669.55867 -> 369.2
CE(20:0) NH4	698.68147 -> 369.2
DG(O-38:9),DG(36:2)_C18:1	638.57237 -> 339.27237
CE(16:0)K	663.548208 -> 369.2
CE(20:3) NH4	692.63457 -> 369.2
DG(34:1)_C16:0	612.55667 -> 339.25667
CE(18:3)H	647.576725 -> 369.2
CE(18:0)K	691.579508 -> 369.2
CE(20:5) NH4	688.60327 -> 369.2
CE(22:6) NH4	714.61887 -> 369.2
DG(39:8),DG(O-40:8)_C18:2	668.61927 -> 371.31927
CE(15:1)K	647.516908 -> 369.2
CE(16:0)Na	647.57427 -> 369.2
CE(22:1)H	707.670625 -> 369.2
DG(36:4),DG(O-37:4)_C18:2	634.57747 -> 337.27747
DG(39:8),DG(O-40:8),DG(38:1)_C18:1	668.61927 -> 369.31927
DG(34:2)_C18:2	610.54107 -> 313.24107
DG(36:3)_C18:1	636.55667 -> 337.25667
CE(20:5)H	671.576725 -> 369.2
CE(20:4) NH4	690.61887 -> 369.2
CE(18:3) NH4	664.60327 -> 369.2
CE(18:1) NH4	668.63457 -> 369.2
DG(34:1)_C18:1	612.55667 -> 313.25667
DG(O-40:9),DG(38:2)_C18:2	666.60367 -> 369.30367
CE(18:2) NH4	666.61887 -> 369.2
CE(16:0) NH4	642.61887 -> 369.2
CE(19:0)H	667.639325 -> 369.2
CE(18:2)Na	671.57427 -> 369.2

DG(37:7),DG(36:0)_C16:0	642.60367 -> 369.30367
CE(16:1)Na	645.55867 -> 369.2
PC(34:3),PC(P-35:2)	756.590725 -> 184.1
CE(20:4)Na	695.57427 -> 369.2
[TG(56:8)]_C22:6	920.86457 -> 575.56457
DG(O-38:8),DG(36:1)_C18:0	640.58797 -> 339.28797
CE(22:5)H	699.608025 -> 369.2
CE(20:0)H	681.654925 -> 369.2
DG(O-38:9),DG(36:2)_C18:2	638.57237 -> 341.27237
PS(P-37:0)	790.596225 -> 605.596225
PS(38:4)	812.544125 -> 627.544125
CE(22:3)H	703.639325 -> 369.2
PE(38:4)	768.554325 -> 627.554325
CE(16:3)Na	641.52737 -> 369.2
CE(20:5)K	709.532608 -> 369.2
CE(20:2)Na	699.60557 -> 369.2
CE(22:2) NH4	722.68147 -> 369.2
PC(39:7),PC(P-40:6),PC(38:0),PC(O-39:0)	818.700225 -> 184.1
DG(39:7)_C18:1	670.70767 -> 371.40767
PC(35:2),PC(O-36:2),PC(P-36:1)	772.622025 -> 184.1
CE(22:1) NH4	724.69717 -> 369.2
CE(20:0)Na	703.63687 -> 369.2
PC(34:2),PC(O-35:2),PC(P-35:1)	758.606325 -> 184.1
PC(38:6)	806.570025 -> 184.1
CE(18:0) NH4	670.65017 -> 369.2
CE(20:2)K	715.579508 -> 369.2
CE(22:6)H	697.592325 -> 369.2
PC(39:5),PC(O-40:5),PC(P-40:4)	822.637625 -> 184.1
PE(34:2),PE(O-35:2),PE(P-35:1)	716.559425 -> 575.559425
CE(16:2)Na	643.54297 -> 369.2
CE(20:5)Na	693.55867 -> 369.2
DG(34:2)_C16:0	610.54107 -> 337.24107
CE(18:1)Na	673.58997 -> 369.2
CE(19:0) NH4	684.66587 -> 369.2
CE(18:1)K	689.563908 -> 369.2
CE(19:0)Na	689.62127 -> 369.2
CE(20:4)H	673.592325 -> 369.2
DG(40:9),DG(39:2)_C18:2	680.61927 -> 383.31927
CE(20:1) NH4	696.66587 -> 369.2
DG(36:3)_C18:2	636.55667 -> 339.25667
CE(22:5) NH4	716.63457 -> 369.2
DG(O-40:9),DG(38:2)_C18:1	666.60367 -> 367.30367
CE(19:0)K	705.595208 -> 369.2
DG(38:3)_C18:2	664.58797 -> 367.28797
DG(O-38:8),DG(36:1)_C18:1	640.58797 -> 341.28797
CE(14:0) NH4	614.58757 -> 369.2
CE(20:2) NH4	694.65017 -> 369.2
CE(22:4)Na	723.60557 -> 369.2
CE(22:3) NH4	720.66587 -> 369.2
DG(37:6)_C16:0	644.52537 -> 371.22537
CE(18:2)K	687.548208 -> 369.2
DG(34:4),DG(dO-36:4)_C16:1	606.58247 -> 335.28247

CE(18:3)K	685.532608 -> 369.2
DG(39:7),DG(38:0),DG(dO-40:0)_C18:0	670.70767 -> 369.40767
DG(34:2)_C18:1	610.54107 -> 311.24107
PE(38:5)	766.538725 -> 625.538725
[TG(53:10),TG(52:3)]_C18:1	874.78637 -> 575.48637
[TG(53:8),TG(52:1)]_C18:0	878.81767 -> 577.51767
[TG(53:9),TG(52:2)]_C18:2	876.80197 -> 579.50197
SM(d18:2/20:1)	755.606725 -> 184.1
[TG(52:4)]_C16:1	872.77067 -> 601.47067
SM(d16:0/23:0)	775.669325 -> 184.1
SM(d18:2/18:1)	727.575425 -> 184.1
CE(22:2)H	705.654925 -> 369.2
DG(32:1)_C16:0	584.52537 -> 311.22537
CE(20:3)Na	697.58997 -> 369.2
DG(34:3)_C18:2	608.52537 -> 311.22537
[TG(56:7)]_C22:5	922.88027 -> 575.58027
SM(d18:0/26:1(17Z))	843.731925 -> 184.1
CE(20:1)K	717.595208 -> 369.2
CE(15:0)K	649.532608 -> 369.2
PE(O-38:8),PE(36:1),PE(O-37:1),PE(P-37:0)	746.606325 -> 605.606325
PE(38:6)	764.523025 -> 623.523025
[TG(54:8),TG(53:1)]_C18:1	892.83327 -> 593.53327
[TG(52:4)]_C18:2	872.77067 -> 575.47067
[TG(55:10),TG(54:3)]_C18:1	902.81767 -> 603.51767
[TG(55:11),TG(54:4)]_C18:1	900.80197 -> 601.50197
[TG(52:4)]_C16:0	872.77067 -> 599.47067
[TG(53:8),TG(52:1)]_C18:1	878.81767 -> 579.51767
SM(d16:1/22:1)	757.622325 -> 184.1
[TG(55:9),TG(54:2)]_C18:1	904.83327 -> 605.53327
PC(32:2),PC(O-33:2),PC(P-33:1)	730.575025 -> 184.1
[TG(53:9),TG(52:2)]_C18:0	876.80197 -> 575.50197
[TG(55:10),TG(54:3)]_C18:2	902.81767 -> 605.51767
FA(14:2)	223.169775 -> 223.169775
PC(33:2),PC(O-34:2),PC(P-34:1)	744.590725 -> 184.1
PC(33:1),PC(O-34:1),PC(P-34:0)	746.606325 -> 184.1
SM(d18:1/19:0)	745.622325 -> 184.1
[TG(53:10),TG(52:3)]_C16:1	874.78637 -> 603.48637
LPC(18:2),LPC(P-19:1)	520.376725 -> 184.1
[TG(55:8),TG(54:1)]_C18:0	906.84897 -> 605.54897
[TG(55:8),TG(54:1)]_C18:1	906.84897 -> 607.54897
[TG(52:5)]_C16:1	870.75507 -> 599.45507
DG(34:2)_C16:1	610.54107 -> 339.24107
[TG(57:11),TG(56:4)]_C18:1	928.83327 -> 629.53327
[TG(56:6)]_C18:2	924.80197 -> 627.50197
DG(O-38:9),DG(36:2)_C18:0	638.57237 -> 337.27237
PE(34:1),PE(O-35:1),PE(P-35:0)	718.575025 -> 577.575025
SM(d18:2/14:0)	673.528425 -> 184.1
[TG(56:6)]_C22:5	924.80197 -> 577.50197
[TG(57:12),TG(56:5)]_C20:4	926.81767 -> 605.51767
DG(37:7)_C16:1	642.60367 -> 371.30367
PC(41:7),PC(P-42:6),PC(40:0),PC(O-41:0)	846.731525 -> 184.1
CE(22:4) NH4	718.65017 -> 369.2

CE(22:4)K	739.579508 -> 369.2
[TG(56:11),TG(55:4)]_C18:2	914.81767 -> 617.51767
[TG(56:11),TG(55:4)]_C18:1	914.81767 -> 615.51767
[TG(58:8)]_C22:6	948.89587 -> 603.59587
SM(d16:1/22:0)	759.638025 -> 184.1
PC(36:4),PC(O-37:4)	782.606325 -> 184.1
[TG(53:10),TG(52:3)]_C18:2	874.78637 -> 577.48637
[TG(53:10),TG(52:3)]_C16:0	874.78637 -> 601.48637
[TG(54:5)]_C18:2	898.78637 -> 601.48637
[TG(55:9),TG(54:2)]_C18:0	904.83327 -> 603.53327
[TG(52:4)]_C18:1	872.77067 -> 573.47067
[TG(52:5)]_C18:2	870.75507 -> 573.45507
LPC(20:4)	544.340325 -> 184.1
CE(17:1) NH4	654.61887 -> 369.2
CAR(10:2)	312.217525 -> 85.1
[TG(53:7)]_C18:1	880.83327 -> 581.53327
DG(40:2)_C18:2	694.63497 -> 397.33497
[TG(56:7),TG(55:0)]_C16:0	922.88027 -> 649.58027
[TG(53:8)]_C18:2	878.81767 -> 581.51767
LPE(20:4)	502.293325 -> 361.293325
DG(34:3)_C16:1	608.52537 -> 337.22537
PC(27:0),PC(O-28:0)	664.528125 -> 184.1
PC(33:3),PC(O-34:3),PC(P-34:2)	742.575025 -> 184.1
FA(22:7)	325.216775 -> 325.216775
FA(17:2)	265.216775 -> 265.216775
FA(21:0)	325.310675 -> 325.310675
FA(20:0)	311.294975 -> 311.294975
DG(34:0)_C18:0	614.57237 -> 313.27237
DG(34:3)_C18:1	608.52537 -> 309.22537
DG(34:0)_C16:0	614.57237 -> 341.27237
DG(37:7),DG(36:0)_C18:0	642.60367 -> 341.30367
DG(32:0)_C16:0	586.54107 -> 313.24107
DG(32:0)_C18:0	586.54107 -> 285.24107
DG(40:5)_C18:0	688.58797 -> 387.28797
DG(40:5)_C16:0	688.58797 -> 415.28797
DG(35:6)_C18:0	616.49407 -> 315.19407
DG(30:0)_C16:0	558.50977 -> 285.20977
FA(6:0)	115.075875 -> 115.075875
FA(18:0)	283.263675 -> 283.263675
FA(19:2)	293.248075 -> 293.248075
DG(38:5)_C16:0	660.55667 -> 387.25667
DG(36:7),DG(35:0)_C18:0	628.58797 -> 327.28797
DG(42:5)_C18:0	716.61927 -> 415.31927
DG(37:6)_C18:0	644.52537 -> 343.22537
FA(15:1)	239.201075 -> 239.201075
DG(35:6)_C16:0	616.49407 -> 343.19407
LPG(19:0),LPG(O-20:0); LPG(19:0),LPG(O-20:0)	544.39787 -> 355.39787
PG(20:0),LPG(21:0); PG(20:0),LPG(21:0)	572.39277 -> 383.39277
DG(33:0)_C16:0	600.55667 -> 327.25667
DG(32:2)_C18:1	582.50977 -> 283.20977
PG(16:0),LPG(17:0),LPG(O-18:0); PG(16:0),LPG(17:0),LPG(O-18:0)	516.36657 -> 327.36657
