# Supplementary material for: Elucidating the Influence of Serum Concentration, Sex, and Particle Size on Iron Oxide Nanoparticle–Lipid Biocorona Formation
Source: Nanomaterials (Basel). 2026 Jun 1;16(11):683. doi: 10.3390/nano16110683 (PMC13258708; doi:10.3390/nano16110683)
Supplement: Supplementary file 1 [file nanomaterials-16-00683-s001.zip › nanomaterials-4334647-supplementary - 副本/Table S3. Male 100 nm Comparison of Lipid Corona Profiles Between Serum Concentration.pdf]

Table S3. Male 100 nm Comparison of Lipid Corona Profiles Between Serum Concentration
Male 100 nm BC Samples

Unique Lipids in 5%	Shared Lipids	Unique Lipids in 10%
FA(22:7)	DG(37:7),DG(36:0)_C16:0	CE(20:5)Na
DG(42:6)_C16:0	DG(34:2)_C18:2	CE(20:0) NH4
FA(17:2)	DG(35:6)_C18:0	CE(16:2)Na
CE(20:2)Na	DG(40:5)_C18:0	CE(22:4)Na
FA(6:0)	CE(18:1) NH4	CE(18:2)K
FA(19:2)	DG(39:8),DG(O-40:8)_C18:2	DG(34:2)_C16:0
FA(26:1)	DG(38:5)_C16:0	DG(36:3)_C18:2
LPG(19:0),LPG(O-20:0); LPG(19:0),LPG(O-20:0)	CE(15:1)K	CE(16:1)Na
FA(28:3)	DG(32:0)_C16:0	DG(37:6)_C16:0
PG(16:0),LPG(17:0),LPG(O-18:0); PG(16:0),LPG(17:0),LPG(O-18:0)	CE(18:0) NH4	DG(39:7)_C18:1
	CE(18:3)H	CE(16:3)Na
	DG(30:0)_C16:0	DG(36:4),DG(O-37:4)_C18:2
	CE(18:2) NH4	[TG(44:5)]_C20:0
	DG(39:7),DG(38:0),DG(dO-40:0)_C18:0	CE(19:0)Na
	CE(18:0)K	CE(20:4)H
	DG(O-38:8),DG(36:1)_C16:1	
	DG(34:0)_C18:0	
	CE(20:4) NH4	
	DG(36:3)_C18:1	
	CE(22:1)H	
	DG(33:0)_C16:0	
	CE(16:0)Na	
	CE(19:0)H	
	DG(40:6),DG(dO-40:0)_C16:0	
	CE(18:2)Na	
	CE(22:2) NH4	
	CE(18:3) NH4	
	CE(20:5)H	
	DG(32:0)_C18:0	
	DG(37:7),DG(36:0)_C18:0	
	CE(16:0) NH4	
	DG(O-38:8),DG(36:1)_C18:0	
	CE(20:3) NH4	
	DG(37:6)_C18:0	
	DG(O-38:8),DG(36:1)_C18:1	
	DG(32:1)_C16:0	
	DG(34:1)_C16:0	
	CE(16:1) NH4	
	DG(35:6)_C16:0	
	CE(18:3)Na	
	CE(22:6) NH4	
	CE(16:0)K	
	DG(34:1)_C18:1	
	DG(42:5)_C18:0	
	[TG(38:0)]_C20:0	
	DG(34:0)_C16:0	
	DG(36:7),DG(35:0)_C18:0	
	DG(40:5)_C16:0	
	DG(O-38:9),DG(36:2)_C18:1	
	CE(20:2)K	
	CE(20:5) NH4	
	DG(34:4),DG(dO-36:4)_C16:1	
	CE(22:5) NH4	
	DG(39:8),DG(O-40:8),DG(38:1)_C18:1	
	DG(O-40:9),DG(38:2)_C18:2	
	CE(18:1)Na	

Table S3. Male 100 nm Comparison of Lipid Corona Profiles Between Serum Concentration
Male 100 nm BC Samples

Unique Lipids in 10%	Shared Lipids	Unique Lipids in 25%
[TG(44:5)]_C20:0	DG(37:7),DG(36:0)_C16:0	CE(18:1)K
	CE(20:5)Na	CE(17:0) NH4
	CE(20:0) NH4	CE(19:0) NH4
	DG(35:6)_C18:0	CE(19:0)K
	DG(34:2)_C18:2	CE(18:3)K
	DG(40:5)_C18:0	CE(22:6)H
	CE(18:1) NH4	DG(34:2)_C18:1
	CE(16:2)Na	CE(22:4)K
	DG(39:8),DG(O-40:8)_C18:2	CE(20:5)K
	DG(38:5)_C16:0	709.686225 -> 369.2
	CE(15:1)K	CE(14:0) NH4
	DG(32:0)_C16:0	CE(20:0)H
	CE(18:0) NH4	DG(38:3)_C18:2
	CE(18:3)H	DG(32:2)_C18:1
	DG(39:7),DG(38:0),DG(dO-40:0)_C18:0	CE(20:3)Na
	DG(30:0)_C16:0	DG(34:2)_C16:1
	CE(18:2) NH4	DG(32:1)_C16:1
	CE(18:0)K	[TG(49:7),TG(48:0)]_C16:0
	DG(O-38:8),DG(36:1)_C16:1	FA(14:2)
	CE(22:4)Na	CE(20:2)Na
	DG(34:0)_C18:0	FA(6:0)
	CE(18:2)K	FA(10:3)
	CE(20:4) NH4	CE(20:0)Na
	CE(22:1)H	DG(32:5)_C18:1
	DG(33:0)_C16:0	CE(22:3) NH4
	DG(36:3)_C18:1	CE(20:4)Na
	CE(16:0)Na	DG(40:9),DG(39:2)_C18:2
	CE(19:0)H	FA(15:1)
	DG(40:6),DG(dO-40:0)_C16:0	DG(38:7),DG(37:0)_C16:0
	CE(18:2)Na	CE(22:1) NH4
	CE(22:2) NH4	CE(20:1)K
	CE(18:3) NH4	Cer(d14:2(4E,6E)/16:0)
	DG(34:2)_C16:0	DG(44:7),DG(43:0)_C16:0
	CE(20:5)H	DG(O-40:9),DG(38:2)_C18:1
	DG(32:0)_C18:0	DG(O-38:9),DG(36:2)_C18:2
	DG(36:3)_C18:2	CE(20:1) NH4
	DG(37:7),DG(36:0)_C18:0	CE(15:0)K
	CE(16:0) NH4	DG(32:1)_C18:1
	DG(O-38:8),DG(36:1)_C18:0	CE(20:2) NH4
	CE(20:3) NH4	Cer(d18:0/21:0)
	DG(37:6)_C18:0	CE(22:2)H
	DG(O-38:8),DG(36:1)_C18:1	DG(O-38:9),DG(36:2)_C18:0
	DG(32:1)_C16:0	CE(22:3)H
	DG(34:1)_C16:0	
	CE(16:1)Na	
	DG(37:6)_C16:0	
	CE(16:1) NH4	
	DG(35:6)_C16:0	
	DG(39:7)_C18:1	
	CE(16:3)Na	
	CE(18:3)Na	
	DG(36:4),DG(O-37:4)_C18:2	
	CE(22:6) NH4	
	CE(16:0)K	
	DG(34:1)_C18:1	
	DG(42:5)_C18:0	
	[TG(38:0)]_C20:0	
	DG(34:0)_C16:0	
	CE(20:2)K	
	DG(O-38:9),DG(36:2)_C18:1	
	DG(40:5)_C16:0	
	DG(36:7),DG(35:0)_C18:0	
	CE(20:4)H	
	CE(19:0)Na	
	CE(20:5) NH4	
	DG(34:4),DG(dO-36:4)_C16:1	

CE(22:5) NH4
DG(39:8),DG(O-40:8),DG(38:1)_C18:1
DG(O-40:9),DG(38:2)_C18:2
CE(18:1)Na

Table S3. Male 100 nm Comparison of Lipid Corona Profiles Between Serum Concentration
Male 100 nm BC Samples

Unique Lipids in 25%	Shared Lipids	Unique Lipids in 50%
DG(32:2)_C18:1	CE(18:1)K	[TG(57:12),TG(56:5)]_C16:0
DG(34:2)_C16:1	CE(20:5)Na	CE(18:0)H
FA(10:3)	CE(17:0) NH4	[TG(52:5)]_C20:4
DG(32:5)_C18:1	CE(20:0) NH4	DG(40:2)_C18:2
DG(38:7),DG(37:0)_C16:0	DG(34:2)_C18:2	[TG(56:10),TG(55:3)]_C18:1
Cer(d14:2(4E,6E)/16:0)	DG(40:5)_C18:0	DG(42:7),DG(41:0)_C16:0
CE(15:0)K	CE(19:0)K	[TG(59:10),TG(58:3)]_C18:1
[TG(38:0)]_C20:0	CE(18:1) NH4	CE(20:0)K
Cer(d18:0/21:0)	CE(18:3)K	DG(30:1)_C18:1
FA(14:2)	CE(22:6)H	[TG(55:8),TG(54:1)]_C18:1
FA(6:0)	DG(39:8),DG(O-40:8)_C18:2	PC(39:7),PC(P-40:6),PC(38:0),PC(O-39:0)
	DG(38:5)_C16:0	CE(22:5)H
	CE(15:1)K	CE(20:4)K
	709.686225 -> 369.2	[TG(50:4)]_C16:0
	CE(18:0) NH4	CE(22:4) NH4
	CE(18:3)H	CE(22:6)Na
	DG(38:3)_C18:2	[TG(56:11),TG(55:4)]_C18:1
	DG(30:0)_C16:0	PC(34:3),PC(P-35:2)
	DG(O-38:8),DG(36:1)_C16:1	[TG(57:10),TG(56:3)]_C18:2
	CE(18:2)K	[TG(56:6)]_C22:5
	CE(20:4) NH4	[TG(54:7)]_C20:4
	DG(33:0)_C16:0	DG(24:0)_C18:0
	DG(36:3)_C18:1	[TG(56:8)]_C22:6
	DG(32:1)_C16:1	[TG(54:11),TG(53:4)]_C18:1
	CE(18:2)Na	LPC(20:4)
	CE(22:2) NH4	DG(37:7)_C16:1
	CE(16:0) NH4	CE(16:2) NH4
	DG(O-38:8),DG(36:1)_C18:0	DG(36:4),DG(O-37:4)_C18:1
	CE(20:3) NH4	PC(14:0),LPC(15:0),LPC(O-16:0)
	CE(20:0)Na	[TG(59:10),TG(58:3)]_C18:2
	DG(O-38:8),DG(36:1)_C18:1	DG(32:2)_C18:2
	CE(22:3) NH4	[TG(50:3)]_C18:3
	DG(32:1)_C16:0	[TG(48:8),TG(47:1)]_C16:0
	DG(40:9),DG(39:2)_C18:2	PC(34:2),PC(O-35:2),PC(P-35:1)
	CE(22:1) NH4	DG(40:8),DG(39:1)_C18:1
	DG(39:7)_C18:1	[TG(48:4)]_C18:2
	DG(O-38:9),DG(36:2)_C18:2	[TG(48:3)]_C18:3
	CE(18:3)Na	[TG(51:7),TG(50:0)]_C18:0
	DG(32:1)_C18:1	CE(24:1)H
	DG(34:0)_C16:0	DG(44:8),DG(43:1)_C16:0
	CE(20:2) NH4	[TG(54:5)]_C20:4
	DG(40:5)_C16:0	DG(34:3)_C18:2
	CE(20:4)H	[TG(52:8),TG(51:1)]_C18:0
	CE(20:5) NH4	CE(17:1) NH4
	CE(22:5) NH4	
	DG(34:4),DG(dO-36:4)_C16:1	
	DG(39:8),DG(O-40:8),DG(38:1)_C18:1	
	CE(22:3)H	
	CE(18:1)Na	
	DG(37:7),DG(36:0)_C16:0	
	DG(35:6)_C18:0	
	CE(19:0) NH4	
	CE(16:2)Na	
	DG(34:2)_C18:1	
	CE(22:4)K	
	CE(20:5)K	
	DG(32:0)_C16:0	
	CE(14:0) NH4	

CE(20:0)H
CE(18:2) NH4
DG(39:7),DG(38:0),DG(dO-40:0)_C18:0
CE(18:0)K
DG(34:0)_C18:0
CE(22:4)Na
CE(20:3)Na
CE(22:1)H
CE(16:0)Na
CE(19:0)H
DG(40:6),DG(dO-40:0)_C16:0
CE(18:3) NH4
DG(34:2)_C16:0
CE(20:5)H
[TG(49:7),TG(48:0)]_C16:0
DG(32:0)_C18:0
CE(20:2)Na
DG(37:7),DG(36:0)_C18:0
DG(36:3)_C18:2
DG(37:6)_C18:0
CE(20:4)Na
FA(15:1)
DG(34:1)_C16:0
CE(16:1)Na
DG(37:6)_C16:0
CE(20:1)K
CE(16:1) NH4
DG(35:6)_C16:0
DG(44:7),DG(43:0)_C16:0
CE(16:3)Na
DG(O-40:9),DG(38:2)_C18:1
DG(36:4),DG(O-37:4)_C18:2
CE(20:1) NH4
CE(22:6) NH4
CE(16:0)K
DG(34:1)_C18:1
DG(42:5)_C18:0
DG(36:7),DG(35:0)_C18:0
DG(O-38:9),DG(36:2)_C18:1
CE(20:2)K
CE(19:0)Na
DG(O-38:9),DG(36:2)_C18:0
CE(22:2)H
DG(O-40:9),DG(38:2)_C18:2

Table S3. Male 100 nm Comparison of Lipid Corona Profiles Between Serum Concentration
Male 100 nm BC Samples

Unique Lipids in 50%	Shared Lipids	Unique Lipids in 75%
DG(24:0)_C18:0	CE(20:5)Na	[TG(46:2)]_C16:0
[TG(59:10),TG(58:3)]_C18:1	DG(34:2)_C18:2	PE(38:4)
[TG(52:8),TG(51:1)]_C18:0	CE(18:1) NH4	SM(d18:1/12:0)
	DG(39:8),DG(O-40:8)_C18:2	PC(44:10),PC(O-44:3)
	[TG(57:12),TG(56:5)]_C16:0	[TG(54:6)]_C18:2
	CE(15:1)K	DG(34:3)_C16:1
	CE(18:0)H	PI(36:2),PI(O-37:2),PI(P-37:1)
	CE(18:3)H	SM(d18:0/26:1(17Z))
	DG(30:0)_C16:0	[TG(54:11),TG(53:4)]_C18:2
	DG(36:3)_C18:1	PC(33:2),PC(O-34:2),PC(P-34:1)
	CE(18:2)Na	[TG(57:12),TG(56:5)]_C18:1
	CE(16:0) NH4	PC(35:2),PC(O-36:2),PC(P-36:1)
	DG(42:7),DG(41:0)_C16:0	PC(39:8),PC(O-40:8),PC(38:1),PC(O-39:1),PC(P-39:0)
	CE(20:3) NH4	LPI(20:0)
	DG(O-38:8),DG(36:1)_C18:1	[TG(52:4)]_C16:1
	DG(32:1)_C16:0	[TG(54:5)]_C18:3
	CE(22:1) NH4	[TG(52:4)]_C18:3
	DG(39:7)_C18:1	SM(d16:1/16:0)
	[TG(55:8),TG(54:1)]_C18:1	PC(39:4),PC(O-40:4),PC(P-40:3)
	DG(O-38:9),DG(36:2)_C18:2	[TG(51:7),TG(50:0)]_C16:0
	PC(39:7),PC(P-40:6),PC(38:0),PC(O-39:0)	PC(O-38:9),PC(36:2),PC(O-37:2),PC(P-37:1)
	CE(18:3)Na	PC(35:5),PC(O-36:5),PC(P-36:4)
	CE(22:5)H	LPC(16:0),PC(O-16:0),LPC(O-17:0)
	CE(20:4)K	CE(22:5)Na
	CE(22:4) NH4	[TG(48:2)]_C14:0
	DG(34:0)_C16:0	[TG(52:4)]_C20:4
	CE(20:2) NH4	PC(42:5)
	DG(40:5)_C16:0	[TG(51:9),TG(50:2)]_C16:1
	CE(20:5) NH4	[TG(52:4)]_C18:1
	CE(22:5) NH4	[TG(48:8),TG(47:1)]_C18:1
	CE(22:3)H	PC(30:0),PC(O-31:0)
	DG(37:7),DG(36:0)_C16:0	[TG(57:12),TG(56:5)]_C20:4
	[TG(56:11),TG(55:4)]_C18:1	PC(40:1),PC(P-41:0)
	DG(34:2)_C18:1	[TG(46:1)]_C18:1
	CE(20:5)K	[TG(55:9),TG(54:2)]_C18:0
	DG(32:0)_C16:0	SM(d16:1/24:0)
	CE(14:0) NH4	[TG(54:10),TG(53:3)]_C18:1
	CE(20:0)H	[TG(53:10),TG(52:3)]_C18:2
	DG(39:7),DG(38:0),DG(dO-40:0)_C18:0	PC(36:7),PC(35:0),PC(O-36:0)
	CE(18:0)K	SM(d16:0/25:0)
	DG(34:0)_C18:0	[TG(52:5)]_C16:0
	CE(20:3)Na	[TG(52:5)]_C18:3
	[TG(54:7)]_C20:4	[TG(56:6)]_C20:4
	[TG(56:8)]_C22:6	[TG(49:8),TG(48:1)]_C16:0
	CE(16:0)Na	[TG(55:10),TG(54:3)]_C18:0
	[TG(54:11),TG(53:4)]_C18:1	PC(39:6),PC(O-40:6),PC(P-40:5)
	LPC(20:4)	PI(38:4)
	DG(37:7)_C16:1	[TG(52:10),TG(51:3)]_C18:1
	CE(18:3) NH4	[TG(53:8),TG(52:1)]_C18:1
	DG(32:0)_C18:0	[TG(55:10),TG(54:3)]_C18:2
	CE(20:2)Na	[TG(48:3)]_C18:2
	DG(36:4),DG(O-37:4)_C18:1	SM(d16:1/25:0)
	[TG(59:10),TG(58:3)]_C18:2	[TG(51:8)]_C18:2
	DG(37:6)_C18:0	PC(41:6),PC(O-42:6)
	FA(15:1)	[TG(48:2)]_C16:0
	DG(40:8),DG(39:1)_C18:1	[TG(55:8),TG(54:1)]_C18:0
	DG(34:1)_C16:0	PC(44:5)
	CE(16:1)Na	PE(38:6)
	[TG(48:4)]_C18:2	[TG(56:12),TG(55:5)]_C18:2

DG(37:6)_C16:0	SM(d16:1/23:0)
CE(16:1) NH4	[TG(56:8)]_C18:2
DG(44:7),DG(43:0)_C16:0	Cer(d18:0/21:0)
CE(16:3)Na	LPC(20:3)
DG(O-40:9),DG(38:2)_C18:1	[TG(56:6)]_C18:2
DG(36:4),DG(O-37:4)_C18:2	PC(42:1)
CE(22:6) NH4	PC(36:3),PC(P-37:2)
CE(24:1)H	[TG(50:4)]_C16:1
DG(44:8),DG(43:1)_C16:0	LPC(18:0),PC(O-18:0),LPC(O-19:0)
DG(36:7),DG(35:0)_C18:0	PE(37:6),PE(O-38:6),PE(P-38:5)
DG(O-38:9),DG(36:2)_C18:1	PC(37:6),PC(O-38:6),PC(P-38:5)
[TG(54:5)]_C20:4	LPC(22:5)
CE(17:1) NH4	PC(32:1),PC(O-33:1),PC(P-33:0)
DG(O-38:9),DG(36:2)_C18:0	[TG(39:0)]_C20:0
CE(22:2)H	[TG(55:11),TG(54:4)]_C18:2
DG(O-40:9),DG(38:2)_C18:2	[TG(54:6)]_C18:1
CE(18:1)K	[TG(50:3)]_C14:0
CE(17:0) NH4	[TG(49:8),TG(48:1)]_C18:1
CE(20:0) NH4	[TG(50:4)]_C18:1
DG(40:5)_C18:0	[TG(53:9),TG(52:2)]_C18:2
CE(19:0)K	[TG(48:3)]_C14:0
CE(18:3)K	SM(d16:0/18:0)
CE(22:6)H	[TG(54:9),TG(53:2)]_C18:1
DG(38:5)_C16:0	PC(34:0),PC(O-35:0)
CE(18:0) NH4	SM(d18:0/15:0)
709.686225 -> 369.2	PC(40:5)
DG(38:3)_C18:2	DG(34:3)_C16:0
[TG(52:5)]_C20:4	SM(d18:2/18:1)
DG(O-38:8),DG(36:1)_C16:1	[TG(53:8),TG(52:1)]_C16:0
CE(18:2)K	[TG(56:7),TG(55:0)]_C16:0
CE(20:4) NH4	PC(34:1),PC(O-35:1),PC(P-35:0)
DG(33:0)_C16:0	LPC(22:6)
DG(32:1)_C16:1	SM(d17:1/24:1)
CE(22:2) NH4	FA(14:2)
DG(40:2)_C18:2	PC(32:2),PC(O-33:2),PC(P-33:1)
[TG(56:10),TG(55:3)]_C18:1	[TG(51:8),TG(50:1)]_C14:0
DG(O-38:8),DG(36:1)_C18:0	SM(d18:1/19:0)
CE(20:0)Na	FA(6:0)
CE(22:3) NH4	[TG(56:7)]_C22:6
CE(20:0)K	CE(17:0)Na
DG(40:9),DG(39:2)_C18:2	[TG(48:3)]_C16:1
DG(30:1)_C18:1	[TG(54:7)]_C18:2
[TG(50:4)]_C16:0	SM(d17:1/26:1)
DG(32:1)_C18:1	[TG(54:6)]_C18:3
CE(20:4)H	[TG(52:5)]_C16:1
CE(22:6)Na	PC(35:3),PC(O-36:3),PC(P-36:2)
DG(34:4),DG(dO-36:4)_C16:1	[TG(55:9),TG(54:2)]_C20:0
DG(39:8),DG(O-40:8),DG(38:1)_C18:1	[TG(57:9),TG(56:2)]_C18:0
CE(18:1)Na	PC(38:4)
DG(35:6)_C18:0	FA(10:2)
CE(19:0) NH4	[TG(57:10),TG(56:3)]_C20:0
CE(16:2)Na	[TG(62:16),TG(61:9),TG(60:2)]_C18:1
PC(34:3),PC(P-35:2)	[TG(54:5)]_C18:2
CE(22:4)K	PC(O-40:9),PC(38:2),PC(P-39:1)
[TG(56:6)]_C22:5	SM(d16:1/20:1)
[TG(57:10),TG(56:3)]_C18:2	[TG(51:7)]_C18:1
CE(18:2) NH4	[TG(53:10),TG(52:3)]_C16:0
CE(22:4)Na	[TG(48:2)]_C18:1
CE(22:1)H	SM(d16:1/20:0)
CE(19:0)H	[TG(51:9),TG(50:2)]_C14:0
DG(40:6),DG(dO-40:0)_C16:0	SM(d18:2/20:1)
[TG(49:7),TG(48:0)]_C16:0	[TG(57:9),TG(56:2)]_C18:1
CE(20:5)H	SM(d18:2/24:1)

DG(34:2)_C16:0	PC(37:4),PC(O-38:4),PC(P-38:3)
CE(16:2) NH4	[TG(52:5)]_C18:2
DG(36:3)_C18:2	SM(d18:0/24:0)
DG(37:7),DG(36:0)_C18:0	[TG(50:3)]_C18:1
PC(14:0),LPC(15:0),LPC(O-16:0)	[TG(52:10),TG(51:3)]_C18:2
DG(32:2)_C18:2	SM(d16:0/24:0)
[TG(50:3)]_C18:3	[TG(48:3)]_C18:1
[TG(48:8),TG(47:1)]_C16:0	CE(24:1) NH4
PC(34:2),PC(O-35:2),PC(P-35:1)	[TG(46:2)]_C14:0
CE(20:4)Na	[TG(53:10),TG(52:3)]_C16:1
[TG(48:3)]_C18:3	[TG(46:0)]_C16:0
CE(20:1)K	PC(42:11),PC(41:4),PC(O-42:4)
DG(35:6)_C16:0	PC(40:7),PC(39:0),PC(O-40:0)
[TG(51:7),TG(50:0)]_C18:0	[TG(46:3)]_C18:2
CE(20:1) NH4	PC(38:5)
DG(34:1)_C18:1	LPC(18:2),LPC(P-19:1)
CE(16:0)K	PC(38:3)
DG(42:5)_C18:0	SM(d16:1/17:0)
CE(20:2)K	[TG(53:9),TG(52:2)]_C18:0
CE(19:0)Na	[TG(48:3)]_C16:0
DG(34:3)_C18:2	[TG(50:3)]_C18:2
	[TG(52:8),TG(51:1)]_C18:1
	[TG(57:9),TG(56:2)]_C20:0
	[TG(50:4)]_C14:0
	[TG(54:5)]_C16:0
	PC(O-38:8),PC(36:1),PC(O-37:1),PC(P-37:0)
	PC(32:0),PC(O-33:0)
	[TG(46:2)]_C16:1
	[TG(53:9),TG(52:2)]_C18:1
	DG(44:9),DG(43:2)_C18:2
	[TG(55:9),TG(54:2)]_C18:1
	DG(34:2)_C16:1
	PC(42:3)
	SM(d16:1/18:1)
	PC(36:5)
	[TG(54:6)]_C16:0
	DG(31:1)_C16:0
	[TG(51:8),TG(50:1)]_C16:0
	PC(39:5),PC(O-40:5),PC(P-40:4)
	CE(22:6)K
	[TG(54:6)]_C20:4
	SM(d18:2/22:1)
	[TG(51:9),TG(50:2)]_C18:2
	PC(40:10),PC(39:3),PC(O-40:3),PC(P-40:2)
	[TG(50:4)]_C18:2
	[TG(48:2)]_C18:2
	PC(38:8),PC(37:1),PC(O-38:1),PC(P-38:0)
	[TG(52:4)]_C18:2
	SM(d18:1/17:0)
	DG(38:7),DG(37:0)_C16:0
	[TG(50:3)]_C16:0
	[TG(53:10),TG(52:3)]_C18:1
	[TG(54:7)]_C18:3
	PC(38:7),PC(37:0),PC(O-38:0)
	PC(42:4)
	[TG(55:10),TG(54:3)]_C16:0
	[TG(52:5)]_C18:1
	SM(d18:2/21:0)
	SM(d16:0/20:0)
	CE(15:0)K
	PC(40:4)
	[TG(52:4)]_C16:0
	[TG(38:0)]_C20:0

		[TG(49:3)]_C18:2
		PI(34:2),PI(O-35:2),PI(P-35:1)
		PC(35:4),PC(O-36:4),PC(P-36:3)
		PC(40:2)
		PC(41:7),PC(P-42:6),PC(40:0),PC(O-41:0)
		PC(40:3)
		PC(28:1),PC(P-29:0)
		PC(38:9),PC(37:2),PC(O-38:2),PC(P-38:1)
		SM(d16:1/22:1)
		PC(36:8),PC(35:1),PC(O-36:1),PC(P-36:0)
		[TG(46:2)]_C18:2
		PC(37:7),PC(P-38:6),PC(36:0),PC(O-37:0)
		SM(d18:1/24:1(15Z))
		SM(d16:1/22:0)
		[TG(54:5)]_C18:1
		DG(42:8),DG(41:1)_C18:1
		SM(d18:1/26:1(17Z))
		PC(30:1),PC(O-31:1),PC(P-31:0)
		PC(41:5),PC(P-42:4)
		[TG(51:9),TG(50:2)]_C16:0
		PC(33:1),PC(O-34:1),PC(P-34:0)
		PC(44:2)
		SM(d16:0/23:0)
		[TG(50:9),TG(49:2)]_C18:2
		CE(20:1)H
		[TG(46:3)]_C16:1
		PE(O-38:9),PE(36:2),PE(O-37:2),PE(P-37:1)
		[TG(55:10),TG(54:3)]_C18:1
		[TG(54:10),TG(53:3)]_C18:2
		PE(34:2),PE(O-35:2),PE(P-35:1)
		PC(37:5),PC(O-38:5),PC(P-38:4)
		LPC(18:1),PC(O-18:1),PC(P-18:0)
		[TG(49:7),TG(48:0)]_C18:0
		[TG(50:5)]_C18:2
		PC(36:4),PC(O-37:4)
		SM(d18:1/25:0)
		PI(36:1),PI(O-37:1),PI(P-37:0)
		SM(d16:0/22:0)
		[TG(57:11),TG(56:4)]_C18:2
		SM(d16:1/24:1)
		CE(16:1)K
		[TG(56:7)]_C22:5
		PC(31:1),PC(O-32:1),PC(P-32:0)
		[TG(51:8),TG(50:1)]_C18:1
		[TG(51:9),TG(50:2)]_C18:0
		PC(40:6)
		[TG(52:9),TG(51:2)]_C18:1
		PC(40:8),PC(39:1),PC(O-40:1),PC(P-40:0)
		SM(d18:0/24:1)
		[TG(53:8),TG(52:1)]_C18:0
		PC(38:6)
		PC(33:3),PC(O-34:3),PC(P-34:2)
		SM(d16:1/18:0)
		[TG(52:9),TG(51:2)]_C16:0
		PC(37:3),PC(O-38:3),PC(P-38:2)
		DG(44:8),DG(43:1)_C18:1
		[TG(48:2)]_C16:1
		[TG(53:9),TG(52:2)]_C16:0
		CE(22:0)K
		PE(36:3),PE(P-37:2)
		PC(30:2),PC(P-31:1)
		PC(40:9),PC(39:2),PC(O-40:2),PC(P-40:1)
		[TG(51:9),TG(50:2)]_C18:1

	[TG(50:3)]_C16:1
	[TG(53:9),TG(52:2)]_C16:1
	[TG(56:7)]_C20:4
	[TG(57:10),TG(56:3)]_C18:1
	[TG(55:11),TG(54:4)]_C18:1
