# Supplementary material for: Elucidating the Influence of Serum Concentration, Sex, and Particle Size on Iron Oxide Nanoparticle–Lipid Biocorona Formation
Source: Nanomaterials (Basel). 2026 Jun 1;16(11):683. doi: 10.3390/nano16110683 (PMC13258708; doi:10.3390/nano16110683)
Supplement: Supplementary file 1 [file nanomaterials-16-00683-s001.zip › nanomaterials-4334647-supplementary - 副本/Table S4. Female 50 nm Comparison of Lipid Corona Profiles Between Serum Concentration.pdf]

Table S4. Female 50 nm Comparison of Lipid Corona Profiles Between Serum Concentration
Female 50 nm BC Samples

Unique Lipids in 5%	Shared Lipids	Unique Lipids in 10%
[TG(42:0)]_C16:0	[TG(53:9),TG(52:2)]_C18:0	FA(21:1)
Cer(d18:0/17:0)	[TG(54:6)]_C18:2	[TG(39:0)]_C20:0
CAR(18:3)	PS(25:0)	[TG(50:3)]_C14:0
[TG(49:7),TG(48:0)]_C14:0	DG(30:3)_C16:1	[TG(52:4)]_C18:3
[TG(48:2)]_C18:1	FA(28:6)	[TG(49:8),TG(48:1)]_C18:1
FA(18:3)	[TG(53:7),TG(52:0)]_C16:0	FA(19:0)
[TG(48:2)]_C16:1	[TG(50:3)]_C18:2	PC(O-38:9),PC(36:2),PC(O-37:2),PC(P-37:1)
	DG(30:2)_C16:1	[TG(54:9),TG(53:2)]_C18:1
	[TG(49:6)]_C16:0	PC(32:0),PC(O-33:0)
	[TG(53:7)]_C18:1	PC(36:5)
	[TG(55:11),TG(54:4)]_C18:2	FA(17:2)
	[TG(51:8),TG(50:1)]_C18:0	PC(34:1),PC(O-35:1),PC(P-35:0)
	[TG(54:5)]_C18:1	FA(6:0)
	[TG(52:4)]_C16:1	[TG(54:10),TG(53:3)]_C18:1
	CAR(20:0)	FA(26:6)
	[TG(53:9),TG(52:2)]_C18:2	SM(d16:0/22:0)
	[TG(51:9),TG(50:2)]_C16:0	DG(32:5)_C18:1
	[TG(51:7),TG(50:0)]_C16:0	[TG(52:5)]_C18:3
	FA(35:0)	[TG(50:3)]_C16:0
	DG(36:8),DG(35:1)_C16:1	FA(16:0)
	FA(22:7)	PC(38:4)
	[TG(53:9),TG(52:2)]_C18:1	[TG(52:9),TG(51:2)]_C18:1
	FA(18:0)	PG(20:0),LPG(21:0); PG(20:0),LPG(21:0)
	FA(22:1)	FA(28:0)
	[TG(55:9),TG(54:2)]_C18:1	DG(36:5)_C16:0
	[TG(53:8),TG(52:1)]_C16:0	[TG(40:0)]_C16:0
	[TG(55:10),TG(54:3)]_C18:1	PC(36:3),PC(P-37:2)
	[TG(51:9),TG(50:2)]_C16:1	PC(38:5)
	DG(36:7),DG(35:0)_C16:0	
	[TG(52:4)]_C18:1	
	[TG(49:7),TG(48:0)]_C16:0	
	[TG(49:8),TG(48:1)]_C14:0	
	DG(41:6)_C16:1	
	[TG(49:7),TG(48:0)]_C18:0	
	[TG(51:8),TG(50:1)]_C16:0	
	[TG(55:9),TG(54:2)]_C18:0	
	PC(36:4),PC(O-37:4)	
	FA(19:2)	
	[TG(53:10),TG(52:3)]_C18:2	
	[TG(51:9),TG(50:2)]_C18:2	
	[TG(53:7),TG(52:0)]_C18:0	
	PC(34:2),PC(O-35:2),PC(P-35:1)	
	[TG(49:8),TG(48:1)]_C16:0	
	DG(41:5)_C16:0	
	[TG(52:4)]_C18:2	
	CE(15:1) NH4	
	[TG(55:10),TG(54:3)]_C18:0	
	[TG(53:10),TG(52:3)]_C18:1	
	[TG(51:8),TG(50:1)]_C18:1	
	Cer(d14:2(4E,6E)/16:0)	
	[TG(53:8),TG(52:1)]_C18:1	
	FA(21:0)	
	PC(40:6)	
	[TG(51:7),TG(50:0)]_C18:0	
	[TG(50:7),TG(49:0)]_C16:0	
	DG(42:11),DG(41:4)_C16:0	
	[TG(54:5)]_C18:2	
	LPG(19:0),LPG(O-20:0); LPG(19:0),LPG(O-20:0)	
	[TG(55:10),TG(54:3)]_C18:2	
	[TG(53:8),TG(52:1)]_C18:0	
	[TG(55:8),TG(54:1)]_C18:1	
	[TG(53:10),TG(52:3)]_C16:0	
	PC(38:6)	
	[TG(55:8),TG(54:1)]_C18:0	
	[TG(51:9),TG(50:2)]_C14:0	
	FA(22:0)	
	[TG(52:4)]_C16:0	
	[TG(52:5)]_C18:2	
	[TG(50:3)]_C18:1	
	[TG(53:9),TG(52:2)]_C16:0	
	PS(O-29:0)	

DG(30:2)_C16:0
[TG(51:9),TG(50:2)]_C18:1
[TG(50:3)]_C16:1
DG(36:6)_C16:0
[TG(53:10),TG(52:3)]_C16:1
[TG(55:11),TG(54:4)]_C18:1
FA(20:0)
[TG(46:0)]_C16:0
DG(36:7)_C16:1
[TG(55:11),TG(54:4)]_C18:0
PG(16:0),LPG(17:0),LPG(O-18:0); PG(16:0),LPG(17:0),LPG(O-18:0)

Table S4. Female 50 nm Comparison of Lipid Corona Profiles Between Serum Concentration
Female 50 nm BC Samples

Unique Lipids in 10%	Shared Lipids	Unique Lipids in 25%
FA(21:1)	[TG(53:9),TG(52:2)]_C18:0	PC(38:3)
FA(28:6)	[TG(54:6)]_C18:2	SM(d16:1/17:0)
FA(19:0)	[TG(53:7),TG(52:0)]_C16:0	PC(33:2),PC(O-34:2),PC(P-34:1)
FA(17:2)	[TG(50:3)]_C18:2	[TG(57:12),TG(56:5)]_C18:1
FA(26:6)	DG(30:2)_C16:1	PC(35:2),PC(O-36:2),PC(P-36:1)
DG(32:5)_C18:1	[TG(49:6)]_C16:0	[TG(54:5)]_C18:3
FA(16:0)	[TG(53:7)]_C18:1	[TG(54:5)]_C16:0
PG(20:0),LPG(21:0); PG(20:0),LPG(21:0)	[TG(51:8),TG(50:1)]_C18:0	PC(O-38:8),PC(36:1),PC(O-37:1),PC(P-37:0)
FA(18:0)	[TG(52:4)]_C16:1	SM(d16:1/16:0)
FA(22:1)	[TG(52:4)]_C18:3	[TG(55:11),TG(54:4)]_C16:0
FA(6:0)	[TG(51:7),TG(50:0)]_C16:0	[TG(55:9),TG(54:2)]_C16:0
FA(19:2)	FA(35:0)	[TG(52:8),TG(51:1)]_C16:0
LPG(19:0),LPG(O-20:0); LPG(19:0),LPG(O-20:0)	DG(36:8),DG(35:1)_C16:1	[TG(57:11),TG(56:4)]_C18:1
FA(28:0)	PC(O-38:9),PC(36:2),PC(O-37:2),PC(P-37:1)	[TG(48:2)]_C14:0
[TG(40:0)]_C16:0	PC(32:0),PC(O-33:0)	SM(d16:1/18:1)
	[TG(53:9),TG(52:2)]_C18:1	PC(30:0),PC(O-31:0)
	[TG(55:9),TG(54:2)]_C18:1	[TG(57:12),TG(56:5)]_C20:4
	[TG(51:9),TG(50:2)]_C16:1	SM(d16:1/24:0)
	DG(36:7),DG(35:0)_C16:0	Cer(d18:1/24:0)
	PC(36:5)	[TG(54:6)]_C20:4
	[TG(52:4)]_C18:1	SM(d18:2/22:1)
	[TG(49:8),TG(48:1)]_C14:0	PC(40:10),PC(39:3),PC(O-40:3),PC(P-40:2)
	[TG(51:8),TG(50:1)]_C16:0	[TG(52:5)]_C16:0
	[TG(55:9),TG(54:2)]_C18:0	[TG(50:4)]_C18:2
	[TG(54:10),TG(53:3)]_C18:1	[TG(48:2)]_C18:2
	[TG(53:10),TG(52:3)]_C18:2	[TG(49:8),TG(48:1)]_C16:1
	[TG(51:9),TG(50:2)]_C18:2	[TG(56:6)]_C20:4
	[TG(53:7),TG(52:0)]_C18:0	SM(d18:1/17:0)
	[TG(52:5)]_C18:3	[TG(55:10),TG(54:3)]_C16:0
	DG(41:5)_C16:0	PC(29:1),PC(O-30:1),PC(P-30:0)
	[TG(49:8),TG(48:1)]_C16:0	PC(28:0),PC(O-29:0)
	[TG(52:4)]_C18:2	[TG(48:2)]_C16:0
	[TG(55:10),TG(54:3)]_C18:0	SM(d16:0/20:0)
	[TG(50:3)]_C16:0	PC(40:4)
	[TG(53:10),TG(52:3)]_C18:1	DG(36:6)_C16:1
	Cer(d14:2(4E,6E)/16:0)	PC(35:4),PC(O-36:4),PC(P-36:3)
	[TG(53:8),TG(52:1)]_C18:1	LPC(18:0),PC(O-18:0),LPC(O-19:0)
	DG(42:11),DG(41:4)_C16:0	[TG(50:4)]_C16:1
	[TG(55:10),TG(54:3)]_C18:2	DG(30:1)_C16:0
	[TG(55:8),TG(54:1)]_C18:1	[TG(45:2)]_C16:0
	[TG(55:8),TG(54:1)]_C18:0	PC(28:1),PC(P-29:0)
	[TG(52:4)]_C16:0	PC(38:9),PC(37:2),PC(O-38:2),PC(P-38:1)
	PC(36:3),PC(P-37:2)	PC(32:1),PC(O-33:1),PC(P-33:0)
	DG(36:7)_C16:1	[TG(53:10),TG(52:3)]_C18:0
	PS(25:0)	SM(d16:1/22:1)
	DG(30:3)_C16:1	PC(36:8),PC(35:1),PC(O-36:1),PC(P-36:0)
	[TG(39:0)]_C20:0	[TG(56:6)]_C22:5
	[TG(55:11),TG(54:4)]_C18:2	PC(37:7),PC(P-38:6),PC(36:0),PC(O-37:0)
	[TG(54:5)]_C18:1	[TG(54:6)]_C18:1
	[TG(50:3)]_C14:0	LPG(20:0); LPG(20:0)
	[TG(49:8),TG(48:1)]_C18:1	SM(d16:1/22:0)
	CAR(20:0)	PC(30:1),PC(O-31:1),PC(P-31:0)
	[TG(51:9),TG(50:2)]_C16:0	[TG(56:7)]_C18:2
	[TG(53:9),TG(52:2)]_C18:2	[TG(51:8),TG(50:1)]_C16:1
	FA(22:7)	SM(d16:0/18:0)
	[TG(54:9),TG(53:2)]_C18:1	PC(33:1),PC(O-34:1),PC(P-34:0)
	[TG(53:8),TG(52:1)]_C16:0	SM(d16:0/23:0)
	[TG(55:10),TG(54:3)]_C18:1	PC(34:0),PC(O-35:0)
	PC(34:1),PC(O-35:1),PC(P-35:0)	PC(40:5)
	[TG(49:7),TG(48:0)]_C16:0	FA(30:0)
	[TG(49:7),TG(48:0)]_C18:0	Cer(d18:0/17:0)
	DG(41:6)_C16:1	[TG(54:10),TG(53:3)]_C18:2
	PC(36:4),PC(O-37:4)	PC(37:5),PC(O-38:5),PC(P-38:4)
	SM(d16:0/22:0)	[TG(51:8),TG(50:1)]_C14:0
	PC(34:2),PC(O-35:2),PC(P-35:1)	SM(d18:1/19:0)
	CE(15:1) NH4	[TG(56:7)]_C22:6

[TG(51:8),TG(50:1)]_C18:1	PC(31:0),PC(O-32:0)
PC(38:4)	[TG(44:1)]_C16:0
FA(21:0)	[TG(54:6)]_C18:3
PC(40:6)	[TG(52:5)]_C16:1
[TG(51:7),TG(50:0)]_C18:0	PC(35:3),PC(O-36:3),PC(P-36:2)
[TG(50:7),TG(49:0)]_C16:0	SM(d16:1/24:1)
[TG(54:5)]_C18:2	PC(31:1),PC(O-32:1),PC(P-32:0)
[TG(52:9),TG(51:2)]_C18:1	PC(40:8),PC(39:1),PC(O-40:1),PC(P-40:0)
[TG(53:8),TG(52:1)]_C18:0	SM(d16:1/20:1)
[TG(53:10),TG(52:3)]_C16:0	[TG(51:7)]_C18:1
PC(38:6)	[TG(48:2)]_C18:1
FA(22:0)	[TG(55:9),TG(54:2)]_C18:2
[TG(51:9),TG(50:2)]_C14:0	SM(d16:1/18:0)
[TG(52:5)]_C18:2	SM(d16:1/20:0)
[TG(50:3)]_C18:1	[TG(52:9),TG(51:2)]_C16:0
[TG(53:9),TG(52:2)]_C16:0	SM(d18:2/24:1)
PS(O-29:0)	PC(37:3),PC(O-38:3),PC(P-38:2)
DG(30:2)_C16:0	PC(37:4),PC(O-38:4),PC(P-38:3)
[TG(51:9),TG(50:2)]_C18:1	[TG(48:2)]_C16:1
[TG(50:3)]_C16:1	[TG(52:10),TG(51:3)]_C18:2
DG(36:6)_C16:0	SM(d16:0/24:0)
[TG(53:10),TG(52:3)]_C16:1	PC(30:2),PC(P-31:1)
DG(36:5)_C16:0	[TG(53:9),TG(52:2)]_C16:1
[TG(55:11),TG(54:4)]_C18:1	[TG(56:7)]_C20:4
FA(20:0)	[TG(54:5)]_C20:4
[TG(46:0)]_C16:0	[TG(57:10),TG(56:3)]_C18:1
[TG(55:11),TG(54:4)]_C18:0	FA(37:0)
PG(16:0),LPG(17:0),LPG(O-18:0); PG(16:0),LPG(17:0),LPG(O-18:0)	
PC(38:5)	

Table S4. Female 50 nm Comparison of Lipid Corona Profiles Between Serum Concentration
Female 50 nm BC Samples

Unique Lipids in 25%	Shared Lipids	Unique Lipids in 50%
FA(30:0)	[TG(54:6)]_C18:2	PE(38:4)
FA(37:0)	PC(33:2),PC(O-34:2),PC(P-34:1)	SM(d18:1/12:0)
[TG(45:2)]_C16:0	[TG(53:7),TG(52:0)]_C16:0	SM(d18:0/17:0)
PS(25:0)	[TG(57:12),TG(56:5)]_C18:1	PC(44:10),PC(O-44:3)
FA(22:0)	PC(35:2),PC(O-36:2),PC(P-36:1)	[TG(54:5)]_C18:0
FA(20:0)	DG(30:2)_C16:1	PI(38:3)
	[TG(52:4)]_C16:1	[TG(59:13),TG(58:6)]_C18:1
	[TG(54:5)]_C18:3	CE(18:1) NH4
	[TG(52:4)]_C18:3	PI(36:2),PI(O-37:2),PI(P-37:1)
	SM(d16:1/16:0)	Cer(d18:1/22:0)
	[TG(51:7),TG(50:0)]_C16:0	[TG(54:11),TG(53:4)]_C18:2
	FA(35:0)	SM(d18:0/26:1(17Z))
	PC(O-38:9),PC(36:2),PC(O-37:2),PC(P-37:1)	DG(39:8),DG(O-40:8)_C18:2
	[TG(52:8),TG(51:1)]_C16:0	[TG(56:12),TG(55:5)]_C18:1
	[TG(57:11),TG(56:4)]_C18:1	PC(39:8),PC(O-40:8),PC(38:1),PC(O-39:1),PC(P-39:0)
	[TG(48:2)]_C14:0	[TG(57:12),TG(56:5)]_C16:0
	[TG(51:9),TG(50:2)]_C16:1	LPI(20:0)
	[TG(52:4)]_C18:1	[TG(53:7),TG(52:0)]_C20:0
	[TG(49:8),TG(48:1)]_C14:0	[TG(44:1)]_C18:1
	PC(30:0),PC(O-31:0)	[TG(50:9),TG(49:2)]_C16:0
	[TG(57:12),TG(56:5)]_C20:4	PC(39:4),PC(O-40:4),PC(P-40:3)
	[TG(55:9),TG(54:2)]_C18:0	[TG(50:9),TG(49:2)]_C18:1
	SM(d16:1/24:0)	PC(35:5),PC(O-36:5),PC(P-36:4)
	[TG(54:10),TG(53:3)]_C18:1	PC(31:2),PC(O-32:2),PC(P-32:1)
	[TG(53:10),TG(52:3)]_C18:2	[TG(38:0)]_C18:0
	Cer(d18:1/24:0)	LPC(16:0),PC(O-16:0),LPC(O-17:0)
	[TG(53:7),TG(52:0)]_C18:0	[TG(46:0)]_C18:0
	[TG(52:5)]_C16:0	[TG(52:4)]_C20:4
	[TG(52:5)]_C18:3	[TG(54:10),TG(53:3)]_C16:0
	[TG(49:8),TG(48:1)]_C16:1	PC(42:5)
	[TG(56:6)]_C20:4	PC(43:4),PC(O-44:4)
	[TG(49:8),TG(48:1)]_C16:0	PC(40:1),PC(P-41:0)
	[TG(55:10),TG(54:3)]_C18:0	[TG(46:1)]_C18:1
	[TG(53:8),TG(52:1)]_C18:1	[TG(52:6)]_C18:2
	DG(42:11),DG(41:4)_C16:0	[TG(54:11),TG(53:4)]_C16:0
	[TG(55:10),TG(54:3)]_C18:2	PC(36:7),PC(35:0),PC(O-36:0)
	[TG(55:8),TG(54:1)]_C18:1	SM(d16:0/25:0)
	[TG(48:2)]_C16:0	DG(32:5)_C18:1
	[TG(55:8),TG(54:1)]_C18:0	Cer(d18:1/23:0)
	DG(36:6)_C16:1	PS(38:4)
	PC(36:3),PC(P-37:2)	LPC(22:4)
	LPC(18:0),PC(O-18:0),LPC(O-19:0)	[TG(56:11),TG(55:4)]_C18:2
	[TG(50:4)]_C16:1	[TG(58:9)]_C22:6
	DG(30:1)_C16:0	CAR(14:1)
	PC(32:1),PC(O-33:1),PC(P-33:0)	PC(39:6),PC(O-40:6),PC(P-40:5)
	[TG(53:10),TG(52:3)]_C18:0	PI(38:4)
	[TG(39:0)]_C20:0	[TG(54:8),TG(53:1)]_C18:1
	[TG(55:11),TG(54:4)]_C18:2	[TG(52:10),TG(51:3)]_C18:1
	[TG(54:6)]_C18:1	[TG(55:7)]_C18:1
	LPG(20:0); LPG(20:0)	[TG(48:3)]_C18:2
	[TG(50:3)]_C14:0	SM(d16:1/25:0)
	[TG(49:8),TG(48:1)]_C18:1	[TG(51:8)]_C18:2
	[TG(53:9),TG(52:2)]_C18:2	PC(41:6),PC(O-42:6)
	[TG(56:7)]_C18:2	PC(39:7),PC(P-40:6),PC(38:0),PC(O-39:0)
	SM(d16:0/18:0)	[TG(56:12),TG(55:5)]_C18:2
	[TG(51:8),TG(50:1)]_C16:1	PE(38:6)
	[TG(54:9),TG(53:2)]_C18:1	CE(22:5)H
	PC(34:0),PC(O-35:0)	SM(d16:1/23:0)
	PC(40:5)	[TG(54:12),TG(53:5)]_C18:2
	[TG(53:8),TG(52:1)]_C16:0	[TG(46:1)]_C16:0
	PC(34:1),PC(O-35:1),PC(P-35:0)	[TG(56:8)]_C18:2
	[TG(51:8),TG(50:1)]_C14:0	[TG(56:6)]_C18:2

SM(d18:1/19:0)
[TG(56:7)]_C22:6
[TG(54:6)]_C18:3
CE(15:1) NH4
[TG(52:5)]_C16:1
PC(35:3),PC(O-36:3),PC(P-36:2)
PC(38:4)
[TG(50:7),TG(49:0)]_C16:0
[TG(54:5)]_C18:2
SM(d16:1/20:1)
[TG(51:7)]_C18:1
[TG(53:10),TG(52:3)]_C16:0
[TG(48:2)]_C18:1
SM(d16:1/20:0)
[TG(51:9),TG(50:2)]_C14:0
SM(d18:2/24:1)
PC(37:4),PC(O-38:4),PC(P-38:3)
[TG(52:5)]_C18:2
[TG(50:3)]_C18:1
[TG(52:10),TG(51:3)]_C18:2
SM(d16:0/24:0)
[TG(54:5)]_C20:4
[TG(53:10),TG(52:3)]_C16:1
DG(36:5)_C16:0
[TG(46:0)]_C16:0
[TG(55:11),TG(54:4)]_C18:0
PC(38:5)
PC(38:3)
SM(d16:1/17:0)
[TG(53:9),TG(52:2)]_C18:0
[TG(50:3)]_C18:2
[TG(49:6)]_C16:0
[TG(53:7)]_C18:1
[TG(51:8),TG(50:1)]_C18:0
PC(O-38:8),PC(36:1),PC(O-37:1),PC(P-37:0)
[TG(54:5)]_C16:0
[TG(55:11),TG(54:4)]_C16:0
[TG(55:9),TG(54:2)]_C16:0
DG(36:8),DG(35:1)_C16:1
PC(32:0),PC(O-33:0)
[TG(53:9),TG(52:2)]_C18:1
[TG(55:9),TG(54:2)]_C18:1
PC(36:5)
SM(d16:1/18:1)
DG(36:7),DG(35:0)_C16:0
[TG(51:8),TG(50:1)]_C16:0
SM(d18:2/22:1)
[TG(54:6)]_C20:4
[TG(51:9),TG(50:2)]_C18:2
PC(40:10),PC(39:3),PC(O-40:3),PC(P-40:2)
[TG(50:4)]_C18:2
[TG(48:2)]_C18:2
DG(41:5)_C16:0
[TG(52:4)]_C18:2
SM(d18:1/17:0)
[TG(50:3)]_C16:0
[TG(53:10),TG(52:3)]_C18:1
Cer(d14:2(4E,6E)/16:0)
[TG(55:10),TG(54:3)]_C16:0
PC(29:1),PC(O-30:1),PC(P-30:0)
PC(28:0),PC(O-29:0)
SM(d16:0/20:0)
PC(40:4)
[TG(52:4)]_C16:0
PC(35:4),PC(O-36:4),PC(P-36:3)
DG(36:7)_C16:1
PC(42:1)
SM(d18:2/14:0)
CE(22:5) NH4
PC(37:6),PC(O-38:6),PC(P-38:5)
[TG(44:0),TG(O-45:0)]_C16:0
Cer(d18:1/24:1(15Z))
SM(d17:0/27:0)
[TG(56:11),TG(55:4)]_C18:1
[TG(54:9),TG(53:2)]_C18:0
[TG(50:8),TG(49:1)]_C18:1
[TG(38:1)]_C18:1
[TG(56:8)]_C20:4
[TG(57:8),TG(56:1)]_C20:0
[TG(50:4)]_C18:1
[TG(49:8)]_C18:2
PC(42:6)
[TG(52:7),TG(51:0)]_C18:0
[TG(55:7),TG(54:0)]_C20:0
[TG(54:7)]_C20:4
[TG(56:8)]_C22:6
[TG(54:11),TG(53:4)]_C18:1
SM(d18:0/15:0)
SM(d18:2/18:1)
LPC(20:4)
[TG(56:7),TG(55:0)]_C16:0
PC(42:2)
[TG(58:8),TG(57:1)]_C18:1
[TG(57:10),TG(56:3)]_C18:0
SM(d17:1/24:1)
CE(20:2)Na
PC(32:2),PC(O-33:2),PC(P-33:1)
PC(44:12),PC(O-44:5)
[TG(50:4)]_C18:3
[TG(56:6)]_C16:0
[TG(48:3)]_C16:1
PC(19:1),LPC(20:1),PC(O-20:1),PC(P-20:0)
[TG(54:7)]_C18:2
[TG(52:6)]_C16:1
PC(28:2)
PC(35:6),PC(P-36:5)
PC(32:3),PC(P-33:2)
[TG(54:8),TG(53:1)]_C18:0
SM(d17:1/26:1)
[TG(53:10),TG(52:3)]_C18:3
[TG(57:12),TG(56:5)]_C22:5
[TG(48:4)]_C18:2
[TG(55:9),TG(54:2)]_C20:0
[TG(52:9),TG(51:2)]_C18:2
[TG(57:9),TG(56:2)]_C18:0
[TG(57:10),TG(56:3)]_C20:0
LPG(19:0),LPG(O-20:0); LPG(19:0),LPG(O-20:0)
PC(O-40:9),PC(38:2),PC(P-39:1)
CE(16:3)Na
FA(24:4)
PC(42:10),PC(41:3),PC(O-42:3),PC(P-42:2)
[TG(44:2)]_C16:0
CE(22:6) NH4
[TG(57:9),TG(56:2)]_C18:1
PC(42:9),PC(41:2),PC(O-42:2),PC(P-42:1)
SM(d16:0/16:0)
[TG(37:0)]_C18:0
SM(d18:0/24:0)
[TG(57:11),TG(56:4)]_C16:0
CAR(14:2)
[TG(48:3)]_C18:1
[TG(58:8)]_C22:5

PC(28:1),PC(P-29:0)
PC(38:9),PC(37:2),PC(O-38:2),PC(P-38:1)
DG(30:3)_C16:1
SM(d16:1/22:1)
PC(36:8),PC(35:1),PC(O-36:1),PC(P-36:0)
[TG(56:6)]_C22:5
PC(37:7),PC(P-38:6),PC(36:0),PC(O-37:0)
[TG(54:5)]_C18:1
SM(d16:1/22:0)
PC(30:1),PC(O-31:1),PC(P-31:0)
CAR(20:0)
[TG(51:9),TG(50:2)]_C16:0
FA(22:7)
PC(33:1),PC(O-34:1),PC(P-34:0)
SM(d16:0/23:0)
Cer(d18:0/17:0)
[TG(55:10),TG(54:3)]_C18:1
[TG(54:10),TG(53:3)]_C18:2
PC(37:5),PC(O-38:5),PC(P-38:4)
[TG(49:7),TG(48:0)]_C16:0
[TG(49:7),TG(48:0)]_C18:0
DG(41:6)_C16:1
PC(31:0),PC(O-32:0)
PC(36:4),PC(O-37:4)
SM(d16:0/22:0)
PC(34:2),PC(O-35:2),PC(P-35:1)
[TG(44:1)]_C16:0
SM(d16:1/24:1)
PC(31:1),PC(O-32:1),PC(P-32:0)
[TG(51:8),TG(50:1)]_C18:1
FA(21:0)
[TG(51:7),TG(50:0)]_C18:0
PC(40:6)
[TG(52:9),TG(51:2)]_C18:1
PC(40:8),PC(39:1),PC(O-40:1),PC(P-40:0)
[TG(53:8),TG(52:1)]_C18:0
PC(38:6)
SM(d16:1/18:0)
[TG(55:9),TG(54:2)]_C18:2
[TG(52:9),TG(51:2)]_C16:0
PC(37:3),PC(O-38:3),PC(P-38:2)
[TG(48:2)]_C16:1
[TG(53:9),TG(52:2)]_C16:0
PS(O-29:0)
DG(30:2)_C16:0
PC(30:2),PC(P-31:1)
[TG(51:9),TG(50:2)]_C18:1
[TG(50:3)]_C16:1
[TG(53:9),TG(52:2)]_C16:1
DG(36:6)_C16:0
[TG(56:7)]_C20:4
[TG(57:10),TG(56:3)]_C18:1
[TG(55:11),TG(54:4)]_C18:1
PG(16:0),LPG(17:0),LPG(O-18:0); PG(16:0),LPG(17:0),LPG(O-18:0)

[TG(46:0)]_C14:0
[TG(58:7)]_C22:5
PC(42:11),PC(41:4),PC(O-42:4)
PC(40:7),PC(39:0),PC(O-40:0)
DG(O-40:9),DG(38:2)_C18:2
LPC(18:2),LPC(P-19:1)
[TG(58:8)]_C22:6
[TG(46:1)]_C14:0
[TG(56:9),TG(55:2)]_C18:1
[TG(54:5)]_C22:5
CE(20:0) NH4
[TG(48:3)]_C16:0
[TG(53:8),TG(52:1)]_C16:1
[TG(52:8),TG(51:1)]_C18:1
[TG(57:9),TG(56:2)]_C20:0
[TG(57:11),TG(56:4)]_C18:0
[TG(50:4)]_C14:0
SM(d18:2/15:0)
[TG(42:0)]_C16:0
PC(29:0),PC(O-30:0)
[TG(52:5)]_C20:4
[TG(54:9),TG(53:2)]_C18:2
[TG(52:7),TG(51:0)]_C16:0
[TG(49:8),TG(48:1)]_C18:0
PC(42:3)
[TG(53:8),TG(52:1)]_C20:0
[TG(54:6)]_C16:0
[TG(56:10),TG(55:3)]_C18:1
[TG(55:11),TG(54:4)]_C20:4
PC(39:5),PC(O-40:5),PC(P-40:4)
[TG(57:12),TG(56:5)]_C18:0
[TG(57:11),TG(56:4)]_C20:0
LPC(20:2),PC(O-20:2)
[TG(49:7)]_C18:1
PC(38:8),PC(37:1),PC(O-38:1),PC(P-38:0)
PC(30:3)
[TG(54:8),TG(53:1)]_C16:0
[TG(56:6)]_C18:0
[TG(52:10),TG(51:3)]_C16:0
[TG(54:7)]_C18:3
PC(38:7),PC(37:0),PC(O-38:0)
PC(42:4)
DG(36:8),DG(35:1)_C18:1
[TG(52:5)]_C18:1
SM(d18:2/21:0)
PC(34:6)
[TG(51:4)]_C18:2
[TG(58:7)]_C18:1
[TG(50:4)]_C16:0
[TG(49:3)]_C18:2
[TG(50:8),TG(49:1)]_C16:0
[TG(55:8),TG(54:1)]_C16:0
PC(40:2)
DG(39:8),DG(O-40:8),DG(38:1)_C18:1
PC(41:7),PC(P-42:6),PC(40:0),PC(O-41:0)
[TG(42:0)]_C14:0
PC(40:3)
PC(33:0),PC(O-34:0)
[TG(57:12),TG(56:5)]_C18:2
PC(34:3),PC(P-35:2)
[TG(46:2)]_C18:2
[TG(57:10),TG(56:3)]_C18:2
SM(d18:1/24:1(15Z))
[TG(56:8),TG(55:1)]_C16:0
SM(d18:1/26:1(17Z))
PC(41:5),PC(P-42:4)

		PC(42:7),PC(41:0),PC(O-42:0)
		CE(18:2) NH4
		[TG(50:9),TG(49:2)]_C18:2
		[TG(52:6)]_C18:3
		FA(22:1)
		CE(19:0)H
		PE(O-38:9),PE(36:2),PE(O-37:2),PE(P-37:1)
		PE(34:2),PE(O-35:2),PE(P-35:1)
		[TG(54:9),TG(53:2)]_C16:0
		[TG(42:1)]_C18:1
		LPC(18:1),PC(O-18:1),PC(P-18:0)
		PC(36:6)
		[TG(50:5)]_C18:2
		FA(19:2)
		SM(d18:1/25:0)
		PI(36:1),PI(O-37:1),PI(P-37:0)
		[TG(50:3)]_C18:3
		[TG(57:11),TG(56:4)]_C18:2
		[TG(55:8),TG(54:1)]_C20:0
		PC(42:0)
		[TG(53:10),TG(52:3)]_C20:0
		[TG(53:8)]_C18:2
		[TG(46:2)]_C18:1
		[TG(49:7),TG(48:0)]_C14:0
		[TG(56:7)]_C22:5
		[TG(51:9),TG(50:2)]_C18:0
		PC(24:0)
		PC(42:8),PC(41:1),PC(O-42:1),PC(P-42:0)
		[TG(51:7),TG(50:0)]_C14:0
		[TG(55:11),TG(54:4)]_C18:3
		SM(d18:0/24:1)
		[TG(50:7),TG(49:0)]_C18:0
		PC(16:0),PC(O-17:0),LPC(O-18:0)
		PC(33:3),PC(O-34:3),PC(P-34:2)
		CE(20:1) NH4
		CE(16:0)K
		PE(36:3),PE(P-37:2)
		1-O-tricosanoyl-Cer(d18:1/16:0)
		PC(43:6)
		PC(29:2),PC(P-30:1)
		PC(40:9),PC(39:2),PC(O-40:2),PC(P-40:1)
		CE(20:2)K
		[TG(40:0)]_C16:0
		[TG(38:0)]_C14:0
		[TG(52:8),TG(51:1)]_C18:0

Table S4. Female 50 nm Comparison of Lipid Corona Profiles Between Serum Concentration
Female 50 nm BC Samples

Unique Lipids in 50%	Shared Lipids	Unique Lipids in 75%
DG(30:2)_C16:1	PE(38:4)	[TG(46:2)]_C16:0
FA(35:0)	SM(d18:1/12:0)	CE(20:3) NH4
DG(32:5)_C18:1	SM(d18:0/17:0)	[TG(52:6)]_C16:0
[TG(56:11),TG(55:4)]_C18:2	PC(44:10),PC(O-44:3)	[TG(48:7),TG(47:0)]_C16:0
DG(42:11),DG(41:4)_C16:0	[TG(54:5)]_C18:0	DG(39:7)_C18:1
DG(36:6)_C16:1	PI(38:3)	CE(18:3)Na
DG(30:1)_C16:0	[TG(59:13),TG(58:6)]_C18:1	[TG(49:7)]_C16:1
CE(15:1) NH4	[TG(54:6)]_C18:2	LPC(20:3)
LPG(19:0),LPG(O-20:0); LPG(19:0),LPG(O-20:0)	CE(18:1) NH4	[TG(46:3)]_C18:1
FA(24:4)	PI(36:2),PI(O-37:2),PI(P-37:1)	[TG(48:3)]_C14:0
[TG(57:11),TG(56:4)]_C16:0	Cer(d18:1/22:0)	CE(20:0)H
DG(36:5)_C16:0	[TG(54:11),TG(53:4)]_C18:2	CE(18:0)K
DG(36:8),DG(35:1)_C16:1	SM(d18:0/26:1(17Z))	CE(20:3)Na
DG(36:7),DG(35:0)_C16:0	PC(33:2),PC(O-34:2),PC(P-34:1)	[TG(44:0),TG(O-45:0)]_C18:0
PC(30:3)	DG(39:8),DG(O-40:8)_C18:2	PC(44:0)
DG(41:5)_C16:0	[TG(53:7),TG(52:0)]_C16:0	CE(16:1) NH4
Cer(d14:2(4E,6E)/16:0)	[TG(57:12),TG(56:5)]_C18:1	[TG(44:0),TG(O-45:0)]_C14:0
DG(36:8),DG(35:1)_C18:1	PC(35:2),PC(O-36:2),PC(P-36:1)	PE(36:4),PE(O-37:4)
DG(36:7)_C16:1	[TG(56:12),TG(55:5)]_C18:1	DG(O-40:9),DG(38:2)_C18:1
[TG(42:0)]_C14:0	PC(39:8),PC(O-40:8),PC(38:1),PC(O-39:1),PC(P-39:0)	[TG(55:11),TG(54:4)]_C16:1
DG(30:3)_C16:1	[TG(57:12),TG(56:5)]_C16:0	[TG(46:1)]_C16:1
CAR(20:0)	LPI(20:0)	Cer(d18:1/16:0)
FA(22:7)	[TG(52:4)]_C16:1	CE(22:6)H
FA(22:1)	[TG(53:7),TG(52:0)]_C20:0	[TG(54:7),TG(53:0)]_C18:0
Cer(d18:0/17:0)	[TG(54:5)]_C18:3	[TG(52:9),TG(51:2)]_C16:1
[TG(42:1)]_C18:1	[TG(52:4)]_C18:3	[TG(54:5)]_C16:1
DG(41:6)_C16:1	[TG(44:1)]_C18:1	PE(O-38:8),PE(36:1),PE(O-37:1),PE(P-37:0)
FA(19:2)	[TG(50:9),TG(49:2)]_C16:0	DG(O-38:8),DG(36:1)_C16:1
PI(36:1),PI(O-37:1),PI(P-37:0)	SM(d16:1/16:0)	CE(20:4) NH4
[TG(53:10),TG(52:3)]_C20:0	PC(39:4),PC(O-40:4),PC(P-40:3)	DG(40:9),DG(39:2)_C18:2
FA(21:0)	[TG(50:9),TG(49:2)]_C18:1	[TG(56:8),TG(55:1)]_C18:1
[TG(51:7),TG(50:0)]_C14:0	[TG(51:7),TG(50:0)]_C16:0	CE(22:6)Na
DG(30:2)_C16:0	PC(O-38:9),PC(36:2),PC(O-37:2),PC(P-37:1)	[TG(51:6)]_C16:0
DG(36:6)_C16:0	[TG(52:8),TG(51:1)]_C16:0	[TG(51:6)]_C18:0
	PC(35:5),PC(O-36:5),PC(P-36:4)	PS(P-37:0)
	PC(31:2),PC(O-32:2),PC(P-32:1)	PE(34:1),PE(O-35:1),PE(P-35:0)
	[TG(57:11),TG(56:4)]_C18:1	FA(18:0)
	[TG(38:0)]_C18:0	CE(20:1)H
	LPC(16:0),PC(O-16:0),LPC(O-17:0)	[TG(55:10),TG(54:3)]_C20:0
	[TG(46:0)]_C18:0	PE(40:6),PE(dO-40:0)
	[TG(48:2)]_C14:0	LPE(22:4)
	[TG(52:4)]_C20:4	PE(38:5)
	[TG(54:10),TG(53:3)]_C16:0	
	[TG(51:9),TG(50:2)]_C16:1	
	PC(42:5)	
	[TG(52:4)]_C18:1	
	[TG(49:8),TG(48:1)]_C14:0	
	PC(43:4),PC(O-44:4)	
	PC(30:0),PC(O-31:0)	
	[TG(57:12),TG(56:5)]_C20:4	
	PC(40:1),PC(P-41:0)	
	[TG(46:1)]_C18:1	
	[TG(55:9),TG(54:2)]_C18:0	
	SM(d16:1/24:0)	
	[TG(54:10),TG(53:3)]_C18:1	
	[TG(52:6)]_C18:2	
	[TG(53:10),TG(52:3)]_C18:2	
	[TG(54:11),TG(53:4)]_C16:0	
	PC(36:7),PC(35:0),PC(O-36:0)	
	SM(d16:0/25:0)	
	Cer(d18:1/24:0)	
	[TG(53:7),TG(52:0)]_C18:0	
	Cer(d18:1/23:0)	
	[TG(52:5)]_C16:0	
	[TG(52:5)]_C18:3	
	PS(38:4)	
	[TG(56:6)]_C20:4	
	[TG(49:8),TG(48:1)]_C16:1	
	LPC(22:4)	
	[TG(49:8),TG(48:1)]_C16:0	
	[TG(55:10),TG(54:3)]_C18:0	
	[TG(58:9)]_C22:6	
	CAR(14:1)	
	PC(39:6),PC(O-40:6),PC(P-40:5)	
	PI(38:4)	
	[TG(54:8),TG(53:1)]_C18:1	
	[TG(52:10),TG(51:3)]_C18:1	
	[TG(53:8),TG(52:1)]_C18:1	

[TG(55:7)]_C18:1
[TG(55:10),TG(54:3)]_C18:2
[TG(48:3)]_C18:2
SM(d16:1/25:0)
[TG(51:8)]_C18:2
[TG(55:8),TG(54:1)]_C18:1
PC(41:6),PC(O-42:6)
[TG(48:2)]_C16:0
PC(39:7),PC(P-40:6),PC(38:0),PC(O-39:0)
[TG(55:8),TG(54:1)]_C18:0
[TG(56:12),TG(55:5)]_C18:2
PE(38:6)
CE(22:5)H
SM(d16:1/23:0)
[TG(54:12),TG(53:5)]_C18:2
[TG(46:1)]_C16:0
[TG(56:8)]_C18:2
[TG(56:6)]_C18:2
PC(42:1)
PC(36:3),PC(P-37:2)
SM(d18:2/14:0)
CE(22:5) NH4
[TG(50:4)]_C16:1
LPC(18:0),PC(O-18:0),LPC(O-19:0)
PC(37:6),PC(O-38:6),PC(P-38:5)
[TG(44:0),TG(O-45:0)]_C16:0
Cer(d18:1/24:1(15Z))
SM(d17:0/27:0)
[TG(56:11),TG(55:4)]_C18:1
[TG(54:9),TG(53:2)]_C18:0
[TG(50:8),TG(49:1)]_C18:1
[TG(38:1)]_C18:1
PC(32:1),PC(O-33:1),PC(P-33:0)
[TG(56:8)]_C20:4
[TG(53:10),TG(52:3)]_C18:0
[TG(39:0)]_C20:0
[TG(55:11),TG(54:4)]_C18:2
[TG(57:8),TG(56:1)]_C20:0
[TG(54:6)]_C18:1
LPG(20:0); LPG(20:0)
[TG(50:3)]_C14:0
[TG(50:4)]_C18:1
[TG(49:8),TG(48:1)]_C18:1
[TG(49:8)]_C18:2
[TG(53:9),TG(52:2)]_C18:2
PC(42:6)
[TG(52:7),TG(51:0)]_C18:0
[TG(55:7),TG(54:0)]_C20:0
[TG(56:7)]_C18:2
[TG(51:8),TG(50:1)]_C16:1
SM(d16:0/18:0)
[TG(54:9),TG(53:2)]_C18:1
[TG(54:7)]_C20:4
[TG(56:8)]_C22:6
PC(34:0),PC(O-35:0)
[TG(54:11),TG(53:4)]_C18:1
SM(d18:0/15:0)
PC(40:5)
SM(d18:2/18:1)
[TG(53:8),TG(52:1)]_C16:0
LPC(20:4)
[TG(56:7),TG(55:0)]_C16:0
PC(42:2)
[TG(58:8),TG(57:1)]_C18:1
[TG(57:10),TG(56:3)]_C18:0
PC(34:1),PC(O-35:1),PC(P-35:0)
SM(d17:1/24:1)
CE(20:2)Na
PC(32:2),PC(O-33:2),PC(P-33:1)
[TG(51:8),TG(50:1)]_C14:0
SM(d18:1/19:0)
PC(44:12),PC(O-44:5)
[TG(56:7)]_C22:6
[TG(50:4)]_C18:3
[TG(56:6)]_C16:0
[TG(48:3)]_C16:1
PC(19:1),LPC(20:1),PC(O-20:1),PC(P-20:0)
[TG(54:7)]_C18:2
[TG(52:6)]_C16:1
PC(28:2)
PC(35:6),PC(P-36:5)
PC(32:3),PC(P-33:2)

[TG(54:8),TG(53:1)]_C18:0
SM(d17:1/26:1)
[TG(53:10),TG(52:3)]_C18:3
[TG(54:6)]_C18:3
[TG(57:12),TG(56:5)]_C22:5
[TG(52:5)]_C16:1
[TG(48:4)]_C18:2
PC(35:3),PC(O-36:3),PC(P-36:2)
[TG(55:9),TG(54:2)]_C20:0
[TG(52:9),TG(51:2)]_C18:2
[TG(57:9),TG(56:2)]_C18:0
PC(38:4)
[TG(57:10),TG(56:3)]_C20:0
[TG(50:7),TG(49:0)]_C16:0
[TG(54:5)]_C18:2
PC(O-40:9),PC(38:2),PC(P-39:1)
CE(16:3)Na
SM(d16:1/20:1)
[TG(51:7)]_C18:1
[TG(53:10),TG(52:3)]_C16:0
[TG(48:2)]_C18:1
PC(42:10),PC(41:3),PC(O-42:3),PC(P-42:2)
[TG(44:2)]_C16:0
SM(d16:1/20:0)
[TG(51:9),TG(50:2)]_C14:0
CE(22:6) NH4
[TG(57:9),TG(56:2)]_C18:1
SM(d18:2/24:1)
SM(d16:0/16:0)
PC(37:4),PC(O-38:4),PC(P-38:3)
PC(42:9),PC(41:2),PC(O-42:2),PC(P-42:1)
[TG(52:5)]_C18:2
[TG(37:0)]_C18:0
[TG(50:3)]_C18:1
SM(d18:0/24:0)
[TG(52:10),TG(51:3)]_C18:2
SM(d16:0/24:0)
CAR(14:2)
[TG(48:3)]_C18:1
[TG(58:8)]_C22:5
[TG(54:5)]_C20:4
[TG(53:10),TG(52:3)]_C16:1
[TG(46:0)]_C14:0
[TG(58:7)]_C22:5
[TG(46:0)]_C16:0
PC(40:7),PC(39:0),PC(O-40:0)
PC(42:11),PC(41:4),PC(O-42:4)
[TG(55:11),TG(54:4)]_C18:0
DG(O-40:9),DG(38:2)_C18:2
PC(38:5)
LPC(18:2),LPC(P-19:1)
PC(38:3)
[TG(58:8)]_C22:6
[TG(46:1)]_C14:0
SM(d16:1/17:0)
[TG(53:9),TG(52:2)]_C18:0
[TG(56:9),TG(55:2)]_C18:1
[TG(54:5)]_C22:5
CE(20:0) NH4
[TG(48:3)]_C16:0
[TG(53:8),TG(52:1)]_C16:1
[TG(50:3)]_C18:2
[TG(49:6)]_C16:0
[TG(53:7)]_C18:1
[TG(51:8),TG(50:1)]_C18:0
[TG(52:8),TG(51:1)]_C18:1
[TG(57:9),TG(56:2)]_C20:0
[TG(57:11),TG(56:4)]_C18:0
[TG(50:4)]_C14:0
SM(d18:2/15:0)
[TG(42:0)]_C16:0
[TG(54:5)]_C16:0
PC(O-38:8),PC(36:1),PC(O-37:1),PC(P-37:0)
[TG(55:11),TG(54:4)]_C16:0
PC(29:0),PC(O-30:0)
[TG(55:9),TG(54:2)]_C16:0
PC(32:0),PC(O-33:0)
[TG(52:5)]_C20:4
[TG(53:9),TG(52:2)]_C18:1
[TG(54:9),TG(53:2)]_C18:2
[TG(52:7),TG(51:0)]_C16:0
[TG(55:9),TG(54:2)]_C18:1

[TG(49:8),TG(48:1)]_C18:0
SM(d16:1/18:1)
PC(36:5)
PC(42:3)
[TG(53:8),TG(52:1)]_C20:0
[TG(54:6)]_C16:0
[TG(56:10),TG(55:3)]_C18:1
[TG(51:8),TG(50:1)]_C16:0
[TG(55:11),TG(54:4)]_C20:4
PC(39:5),PC(O-40:5),PC(P-40:4)
[TG(57:12),TG(56:5)]_C18:0
[TG(57:11),TG(56:4)]_C20:0
LPC(20:2),PC(O-20:2)
SM(d18:2/22:1)
[TG(54:6)]_C20:4
[TG(49:7)]_C18:1
PC(40:10),PC(39:3),PC(O-40:3),PC(P-40:2)
[TG(51:9),TG(50:2)]_C18:2
[TG(50:4)]_C18:2
PC(38:8),PC(37:1),PC(O-38:1),PC(P-38:0)
[TG(48:2)]_C18:2
[TG(54:8),TG(53:1)]_C16:0
[TG(56:6)]_C18:0
[TG(52:4)]_C18:2
SM(d18:1/17:0)
[TG(52:10),TG(51:3)]_C16:0
[TG(50:3)]_C16:0
[TG(53:10),TG(52:3)]_C18:1
[TG(54:7)]_C18:3
PC(38:7),PC(37:0),PC(O-38:0)
PC(42:4)
[TG(55:10),TG(54:3)]_C16:0
PC(29:1),PC(O-30:1),PC(P-30:0)
[TG(52:5)]_C18:1
PC(28:0),PC(O-29:0)
SM(d18:2/21:0)
PC(34:6)
[TG(51:4)]_C18:2
SM(d16:0/20:0)
[TG(58:7)]_C18:1
PC(40:4)
[TG(52:4)]_C16:0
[TG(50:4)]_C16:0
[TG(49:3)]_C18:2
PC(35:4),PC(O-36:4),PC(P-36:3)
[TG(50:8),TG(49:1)]_C16:0
[TG(55:8),TG(54:1)]_C16:0
PC(40:2)
DG(39:8),DG(O-40:8),DG(38:1)_C18:1
PC(41:7),PC(P-42:6),PC(40:0),PC(O-41:0)
PC(40:3)
PC(28:1),PC(P-29:0)
PC(33:0),PC(O-34:0)
PC(38:9),PC(37:2),PC(O-38:2),PC(P-38:1)
[TG(57:12),TG(56:5)]_C18:2
SM(d16:1/22:1)
PC(34:3),PC(P-35:2)
PC(36:8),PC(35:1),PC(O-36:1),PC(P-36:0)
[TG(46:2)]_C18:2
[TG(57:10),TG(56:3)]_C18:2
[TG(56:6)]_C22:5
PC(37:7),PC(P-38:6),PC(36:0),PC(O-37:0)
SM(d18:1/24:1(15Z))
SM(d16:1/22:0)
[TG(54:5)]_C18:1
[TG(56:8),TG(55:1)]_C16:0
PC(41:5),PC(P-42:4)
SM(d18:1/26:1(17Z))
PC(30:1),PC(O-31:1),PC(P-31:0)
PC(42:7),PC(41:0),PC(O-42:0)
[TG(51:9),TG(50:2)]_C16:0
CE(18:2) NH4
PC(33:1),PC(O-34:1),PC(P-34:0)
SM(d16:0/23:0)
[TG(50:9),TG(49:2)]_C18:2
[TG(52:6)]_C18:3
CE(19:0)H
PE(O-38:9),PE(36:2),PE(O-37:2),PE(P-37:1)
[TG(55:10),TG(54:3)]_C18:1
PE(34:2),PE(O-35:2),PE(P-35:1)
[TG(54:10),TG(53:3)]_C18:2
PC(37:5),PC(O-38:5),PC(P-38:4)

[TG(54:9),TG(53:2)]_C16:0
[TG(49:7),TG(48:0)]_C16:0
LPC(18:1),PC(O-18:1),PC(P-18:0)
[TG(49:7),TG(48:0)]_C18:0
PC(36:6)
[TG(50:5)]_C18:2
PC(36:4),PC(O-37:4)
PC(31:0),PC(O-32:0)
SM(d18:1/25:0)
SM(d16:0/22:0)
[TG(50:3)]_C18:3
[TG(57:11),TG(56:4)]_C18:2
PC(34:2),PC(O-35:2),PC(P-35:1)
[TG(55:8),TG(54:1)]_C20:0
[TG(44:1)]_C16:0
PC(42:0)
[TG(53:8)]_C18:2
[TG(46:2)]_C18:1
[TG(49:7),TG(48:0)]_C14:0
SM(d16:1/24:1)
[TG(56:7)]_C22:5
PC(31:1),PC(O-32:1),PC(P-32:0)
[TG(51:8),TG(50:1)]_C18:1
[TG(51:9),TG(50:2)]_C18:0
PC(40:6)
[TG(51:7),TG(50:0)]_C18:0
PC(24:0)
PC(42:8),PC(41:1),PC(O-42:1),PC(P-42:0)
[TG(52:9),TG(51:2)]_C18:1
[TG(55:11),TG(54:4)]_C18:3
PC(40:8),PC(39:1),PC(O-40:1),PC(P-40:0)
SM(d18:0/24:1)
[TG(53:8),TG(52:1)]_C18:0
[TG(50:7),TG(49:0)]_C18:0
PC(16:0),PC(O-17:0),LPC(O-18:0)
PC(38:6)
[TG(55:9),TG(54:2)]_C18:2
SM(d16:1/18:0)
PC(33:3),PC(O-34:3),PC(P-34:2)
CE(20:1) NH4
[TG(52:9),TG(51:2)]_C16:0
PC(37:3),PC(O-38:3),PC(P-38:2)
[TG(48:2)]_C16:1
PS(O-29:0)
[TG(53:9),TG(52:2)]_C16:0
CE(16:0)K
PE(36:3),PE(P-37:2)
1-O-tricosanoyl-Cer(d18:1/16:0)
PC(30:2),PC(P-31:1)
PC(43:6)
PC(29:2),PC(P-30:1)
PC(40:9),PC(39:2),PC(O-40:2),PC(P-40:1)
[TG(51:9),TG(50:2)]_C18:1
CE(20:2)K
[TG(50:3)]_C16:1
[TG(53:9),TG(52:2)]_C16:1
[TG(56:7)]_C20:4
[TG(57:10),TG(56:3)]_C18:1
[TG(40:0)]_C16:0
[TG(38:0)]_C14:0
[TG(55:11),TG(54:4)]_C18:1
[TG(52:8),TG(51:1)]_C18:0
PG(16:0),LPG(17:0),LPG(O-18:0); PG(16:0),LPG(17:0),LPG(O-18:0)
