# Supplementary material for: Elucidating the Influence of Serum Concentration, Sex, and Particle Size on Iron Oxide Nanoparticle–Lipid Biocorona Formation
Source: Nanomaterials (Basel). 2026 Jun 1;16(11):683. doi: 10.3390/nano16110683 (PMC13258708; doi:10.3390/nano16110683)
Supplement: Supplementary file 1 [file nanomaterials-16-00683-s001.zip › nanomaterials-4334647-supplementary - 副本/Table S5. Female 100 nm Comparison of Lipid Corona Profiles Between Serum Concentration.pdf]

Table S5. Female 100 nm Comparison of Lipid Corona Profiles Between Serum Concentration
Female 100 nm BC Samples

Unique Lipids in 5%	Shared Lipids	Unique Lipids in 10%
DG(39:7),DG(38:0),DG(dO-40:0)_C18:0	DG(37:7),DG(36:0)_C16:0	DG(34:2)_C18:2
FA(18:0)	DG(35:6)_C18:0	DG(39:8),DG(O-40:8)_C18:2
DG(36:7),DG(35:0)_C18:0	DG(40:5)_C18:0	CE(18:0)K
FA(6:0)	CE(18:1) NH4	CE(16:0)Na
FA(19:2)	FA(15:1)	CE(22:2) NH4
	DG(34:1)_C16:0	DG(39:8),DG(O-40:8),DG(38:1)_C18:1
	DG(38:5)_C16:0	
	FA(21:0)	
	DG(35:6)_C16:0	
	DG(32:0)_C16:0	
	LPG(19:0),LPG(O-20:0); LPG(19:0),LPG(O-20:0)	
	FA(22:7)	
	CE(18:2) NH4	
	DG(30:0)_C16:0	
	DG(32:2)_C18:1	
	DG(34:0)_C18:0	
	DG(34:3)_C18:1	
	CE(20:4) NH4	
	DG(33:0)_C16:0	
	CE(22:6) NH4	
	CE(19:0)H	
	DG(34:1)_C18:1	
	CE(18:2)Na	
	FA(17:2)	
	DG(42:5)_C18:0	
	CE(18:3) NH4	
	DG(34:0)_C16:0	
	DG(40:5)_C16:0	
	CE(20:5)H	
	DG(32:0)_C18:0	
	DG(37:7),DG(36:0)_C18:0	
	CE(16:0) NH4	
	FA(20:0)	
	CE(20:5) NH4	
	DG(34:4),DG(dO-36:4)_C16:1	
	PG(16:0),LPG(17:0),LPG(O-18:0); PG(16:0),LPG(17:0),LPG(O-18:0)	
	DG(O-40:9),DG(38:2)_C18:2	
	DG(37:6)_C18:0	

Table S5. Female 100 nm Comparison of Lipid Corona Profiles Between Serum Concentration

Female 100 nm BC Samples

Unique Lipids in 10%	Shared Lipids	Unique Lipids in 25%
FA(15:1)	DG(37:7),DG(36:0)_C16:0	CE(20:0) NH4
FA(21:0)	DG(34:2)_C18:2	CE(16:1)Na
LPG(19:0),LPG(O-20:0); LPG(19:0),LPG(O-20:0)	DG(35:6)_C18:0	CE(16:1) NH4
FA(22:7)	DG(40:5)_C18:0	CE(15:1)K
FA(17:2)	CE(18:1) NH4	CE(18:3)H
FA(20:0)	DG(39:8),DG(O-40:8)_C18:2	DG(39:7),DG(38:0),DG(dO-40:0)_C18:0
	DG(34:1)_C16:0	DG(O-38:8),DG(36:1)_C16:1
	DG(38:5)_C16:0	CE(18:3)Na
	DG(35:6)_C16:0	PG(20:0),LPG(21:0); PG(20:0),LPG(21:0)
	DG(32:0)_C16:0	DG(36:4),DG(O-37:4)_C18:2
	DG(30:0)_C16:0	DG(36:3)_C18:1
	CE(18:2) NH4	CE(22:1)H
	CE(18:0)K	CE(16:0)K
	DG(32:2)_C18:1	DG(36:7),DG(35:0)_C18:0
	DG(34:0)_C18:0	DG(O-38:9),DG(36:2)_C18:1
	DG(34:3)_C18:1	CE(20:3) NH4
	CE(20:4) NH4	
	DG(33:0)_C16:0	
	CE(22:6) NH4	
	CE(16:0)Na	
	CE(19:0)H	
	DG(34:1)_C18:1	
	CE(18:2)Na	
	DG(42:5)_C18:0	
	CE(22:2) NH4	
	CE(18:3) NH4	
	DG(34:0)_C16:0	
	DG(40:5)_C16:0	
	CE(20:5)H	
	DG(32:0)_C18:0	
	DG(37:7),DG(36:0)_C18:0	
	CE(16:0) NH4	
	CE(20:5) NH4	
	DG(34:4),DG(dO-36:4)_C16:1	
	DG(39:8),DG(O-40:8),DG(38:1)_C18:1	
	PG(16:0),LPG(17:0),LPG(O-18:0); PG(16:0),LPG(17:0),LPG(O-18:0)	
	DG(O-40:9),DG(38:2)_C18:2	
	DG(37:6)_C18:0	

Table S5. Female 100 nm Comparison of Lipid Corona Profiles Between Serum Concentration
Female 100 nm BC Samples

Unique Lipids in 25%	Shared Lipids	Unique Lipids in 50%
PG(20:0),LPG(21:0)	DG(37:7),DG(36:0)_C16:0	CE(18:1)K
	CE(20:0) NH4	PS(P-37:0)
	DG(35:6)_C18:0	PE(38:4)
	DG(34:2)_C18:2	CE(20:5)Na
	DG(40:5)_C18:0	CE(19:0) NH4
	CE(18:1) NH4	CE(19:0)K
	DG(39:8),DG(O-40:8)_C18:2	CE(16:2)Na
	DG(38:5)_C16:0	CE(18:3)K
	CE(15:1)K	CE(22:6)H
	DG(32:0)_C16:0	PC(35:2),PC(O-36:2),PC(P-36:1)
	CE(18:3)H	DG(34:2)_C18:1
	DG(39:7),DG(38:0),DG(dO-40:0)_C18:0	PC(34:3),PC(P-35:2)
	DG(30:0)_C16:0	CE(20:5)K
	CE(18:2) NH4	CE(18:0) NH4
	CE(18:0)K	CE(14:0) NH4
	DG(32:2)_C18:1	CE(20:0)H
	DG(O-38:8),DG(36:1)_C16:1	DG(38:3)_C18:2
	DG(34:0)_C18:0	CE(22:4)Na
	CE(20:4) NH4	CE(18:2)K
	DG(36:3)_C18:1	[TG(56:8)]_C22:6
	CE(22:1)H	PE(34:2),PE(O-35:2),PE(P-35:1)
	DG(33:0)_C16:0	DG(34:2)_C16:0
	CE(16:0)Na	CE(20:2)Na
	CE(19:0)H	DG(36:3)_C18:2
	CE(18:2)Na	PC(39:5),PC(O-40:5),PC(P-40:4)
	CE(22:2) NH4	DG(O-38:8),DG(36:1)_C18:0
	CE(18:3) NH4	CE(20:0)Na
	CE(20:5)H	DG(O-38:8),DG(36:1)_C18:1
	DG(32:0)_C18:0	PS(38:4)
	DG(37:7),DG(36:0)_C18:0	PC(34:2),PC(O-35:2),PC(P-35:1)
	CE(16:0) NH4	CE(22:3) NH4
	CE(20:3) NH4	CE(20:4)Na
	DG(37:6)_C18:0	DG(40:9),DG(39:2)_C18:2
	DG(34:1)_C16:0	FA(15:1)
	CE(16:1)Na	CE(22:1) NH4
	CE(16:1) NH4	DG(37:6)_C16:0
	DG(35:6)_C16:0	DG(39:7)_C18:1
	CE(18:3)Na	LPG(19:0),LPG(O-20:0); LPG(19:0),LPG(O-20:0)
	DG(34:3)_C18:1	CE(16:3)Na
	DG(36:4),DG(O-37:4)_C18:2	DG(O-38:9),DG(36:2)_C18:2
	CE(22:6) NH4	DG(O-40:9),DG(38:2)_C18:1
	DG(34:1)_C18:1	PC(39:7),PC(P-40:6),PC(38:0),PC(O-39:0)
	CE(16:0)K	PC(38:6)
	DG(42:5)_C18:0	CE(20:1) NH4
	DG(34:0)_C16:0	CE(22:5)H
	DG(40:5)_C16:0	CE(20:2) NH4
	DG(O-38:9),DG(36:2)_C18:1	CE(20:2)K
	DG(36:7),DG(35:0)_C18:0	CE(19:0)Na
	CE(20:5) NH4	CE(20:4)H
	DG(34:4),DG(dO-36:4)_C16:1	CE(22:5) NH4
	DG(39:8),DG(O-40:8),DG(38:1)_C18:1	CE(22:3)H
	DG(O-40:9),DG(38:2)_C18:2	CE(18:1)Na
	PG(16:0),LPG(17:0),LPG(O-18:0); PG(16:0),LPG(17:0),LPG(O-18:0)	

Table S5. Female 100 nm Comparison of Lipid Corona Profiles Between Serum Concentration
Female 100 nm BC Samples

Unique Lipids in 50%	Shared Lipids	Unique Lipids in 75%
	CE(18:1)K	LPC(18:2),LPC(P-19:1)
	PE(38:4)	[TG(58:8)]_C22:6
	CE(20:5)Na	[TG(53:9),TG(52:2)]_C18:0
	CE(20:0) NH4	DG(34:3)_C16:1
	DG(34:2)_C18:2	SM(d18:0/26:1(17Z))
	DG(40:5)_C18:0	PC(33:2),PC(O-34:2),PC(P-34:1)
	CE(19:0)K	[TG(53:7)]_C18:1
	CE(18:1) NH4	[TG(52:4)]_C16:1
	CE(18:3)K	PE(O-38:8),PE(36:1),PE(O-37:1),PE(P-37:0)
	CE(22:6)H	[TG(57:11),TG(56:4)]_C18:1
	DG(39:8),DG(O-40:8)_C18:2	[TG(55:9),TG(54:2)]_C18:1
	PC(35:2),PC(O-36:2),PC(P-36:1)	DG(34:2)_C16:1
	DG(38:5)_C16:0	[TG(52:4)]_C18:1
	CE(15:1)K	DG(40:2)_C18:2
	CE(18:0) NH4	[TG(57:12),TG(56:5)]_C20:4
	CE(18:3)H	[TG(55:9),TG(54:2)]_C18:0
	DG(38:3)_C18:2	[TG(53:10),TG(52:3)]_C18:2
	DG(30:0)_C16:0	LPE(20:4)
	DG(32:2)_C18:1	DG(32:1)_C16:0
	DG(O-38:8),DG(36:1)_C16:1	[TG(56:11),TG(55:4)]_C18:2
	CE(18:2)K	[TG(52:4)]_C18:2
	CE(20:4) NH4	PC(27:0),PC(O-28:0)
	DG(33:0)_C16:0	[TG(53:10),TG(52:3)]_C18:1
	DG(36:3)_C18:1	[TG(54:8),TG(53:1)]_C18:1
	CE(18:2)Na	[TG(53:8),TG(52:1)]_C18:1
	CE(22:2) NH4	[TG(55:10),TG(54:3)]_C18:2
	CE(16:0) NH4	[TG(55:8),TG(54:1)]_C18:1
	PC(39:5),PC(O-40:5),PC(P-40:4)	[TG(55:8),TG(54:1)]_C18:0
	DG(O-38:8),DG(36:1)_C18:0	CE(15:0)K
	CE(20:3) NH4	PE(38:6)
	CE(20:0)Na	[TG(52:4)]_C16:0
	DG(O-38:8),DG(36:1)_C18:1	CE(22:4) NH4
	PS(38:4)	[TG(56:6)]_C18:2
	CE(22:3) NH4	SM(d18:2/14:0)
	DG(40:9),DG(39:2)_C18:2	PC(41:7),PC(P-42:6),PC(40:0),PC(O-41:0)
	CE(22:1) NH4	[TG(56:11),TG(55:4)]_C18:1
	DG(39:7)_C18:1	PE(34:1),PE(O-35:1),PE(P-35:0)
	DG(O-38:9),DG(36:2)_C18:2	SM(d16:1/22:1)
	PC(39:7),PC(P-40:6),PC(38:0),PC(O-39:0)	CE(22:4)K
	CE(18:3)Na	[TG(56:6)]_C22:5
	CE(22:5)H	SM(d16:1/22:0)
	DG(34:0)_C16:0	[TG(53:9),TG(52:2)]_C18:2
	CE(20:2) NH4	PC(33:1),PC(O-34:1),PC(P-34:0)
	DG(40:5)_C16:0	CE(20:3)Na
	CE(20:4)H	SM(d16:0/23:0)
	CE(20:5) NH4	SM(d18:2/18:1)
	CE(22:5) NH4	LPC(20:4)
	DG(34:4),DG(dO-36:4)_C16:1	[TG(56:7),TG(55:0)]_C16:0
	DG(39:8),DG(O-40:8),DG(38:1)_C18:1	[TG(55:10),TG(54:3)]_C18:1
	CE(22:3)H	DG(37:7)_C16:1
	CE(18:1)Na	CAR(10:2)
	PS(P-37:0)	FA(14:2)
	DG(37:7),DG(36:0)_C16:0	PC(32:2),PC(O-33:2),PC(P-33:1)
	DG(35:6)_C18:0	SM(d18:1/19:0)
	CE(19:0) NH4	PC(36:4),PC(O-37:4)
	CE(16:2)Na	[TG(53:8)]_C18:2
	DG(34:2)_C18:1	[TG(52:5)]_C16:1
	PC(34:3),PC(P-35:2)	CE(20:1)K
	CE(20:5)K	[TG(56:7)]_C22:5
	DG(32:0)_C16:0	[TG(54:5)]_C18:2
	CE(14:0) NH4	[TG(53:8),TG(52:1)]_C18:0
	CE(20:0)H	[TG(53:10),TG(52:3)]_C16:0
	CE(18:2) NH4	PE(38:5)
	DG(39:7),DG(38:0),DG(dO-40:0)_C18:0	PC(33:3),PC(O-34:3),PC(P-34:2)
	CE(18:0)K	SM(d18:2/20:1)
	DG(34:0)_C18:0	[TG(52:5)]_C18:2
	CE(22:4)Na	[TG(53:10),TG(52:3)]_C16:1
	CE(22:1)H	[TG(55:11),TG(54:4)]_C18:1
	[TG(56:8)]_C22:6	CE(17:1) NH4
	CE(16:0)Na	DG(34:3)_C18:2
	CE(19:0)H	DG(O-38:9),DG(36:2)_C18:0
	PE(34:2),PE(O-35:2),PE(P-35:1)	CE(22:2)H
	CE(18:3) NH4	
	DG(34:2)_C16:0	
	CE(20:5)H	
	DG(32:0)_C18:0	
	CE(20:2)Na	
	DG(37:7),DG(36:0)_C18:0	

DG(36:3)_C18:2
DG(37:6)_C18:0
PC(34:2),PC(O-35:2),PC(P-35:1)
CE(20:4)Na
FA(15:1)
DG(34:1)_C16:0
CE(16:1)Na
DG(37:6)_C16:0
CE(16:1) NH4
DG(35:6)_C16:0
LPG(19:0),LPG(O-20:0); LPG(19:0),LPG(O-20:0)
CE(16:3)Na
DG(O-40:9),DG(38:2)_C18:1
PC(38:6)
CE(20:1) NH4
DG(34:3)_C18:1
DG(36:4),DG(O-37:4)_C18:2
CE(22:6) NH4
DG(34:1)_C18:1
CE(16:0)K
DG(42:5)_C18:0
DG(36:7),DG(35:0)_C18:0
DG(O-38:9),DG(36:2)_C18:1
CE(20:2)K
CE(19:0)Na
DG(O-40:9),DG(38:2)_C18:2
PG(16:0),LPG(17:0),LPG(O-18:0); PG(16:0),LPG(17:0),LPG(O-18:0)
