# Supplementary material for: Elucidating the Influence of Serum Concentration, Sex, and Particle Size on Iron Oxide Nanoparticle–Lipid Biocorona Formation
Source: Nanomaterials (Basel). 2026 Jun 1;16(11):683. doi: 10.3390/nano16110683 (PMC13258708; doi:10.3390/nano16110683)
Supplement: Supplementary file 1 [file nanomaterials-16-00683-s001.zip › nanomaterials-4334647-supplementary - 副本/Table S6. Correlation Analysis of Fe3O4 NP-BC Lipid Abundance in Relation to Serum Concentration.pdf]

Table S6. Correlation Analysis of Fe3O4 NP-BC Lipid Abundance in Relation to Serum Concentration
BC Lipid Abundance Correlation Analysis with Male 50 nm Samples

Serum concentration (% v/v)	5	10	25	50	75			
	Group Mean Relative Lipid Abundance							
Lipid name	Male 5% 50 nm	Male 10% 50nm	Male 25% 50nm	Male 50% 50nm	Male 75% 50nm	Pearson's Correlation	R^2	p-value
PC(30:1),PC(O-31:1),PC(P-31:0)	0	13564.88892	64573.39214	134594.0851	161111.2383	0.981628214	0.963594	0.00298
LPC(18:0),PC(O-18:0),LPC(O-19:	17767.2812	21227.75334	65007.99672	112072.2476	133033.3145	0.980247158	0.960884	0.00332
SM(d16:1/18:0)	0	42707.93123	193265.0535	388896.2189	461115.2662	0.979287005	0.959003	0.00357
[TG(54:5)]_C18:2	3872.608257	6417.596468	28317.79022	49269.20317	59780.23622	0.978902625	0.95825	0.00367
SM(d16:0/18:0)	0	5873.64444	20321.34543	38158.91861	45305.67894	0.977147805	0.954818	0.00413
SM(d16:0/22:0)	3671.508271	8298.732584	38331.0911	66962.92455	79545.13737	0.97480912	0.950253	0.00478
[TG(50:3)]_C18:2	1920.732134	3409.232259	11947.33685	21190.37765	24569.95376	0.974766676	0.95017	0.00479
SM(d16:1/24:1)	0	9703.288729	51076.21925	94777.06262	112030.912	0.974754682	0.950147	0.0048
SM(d16:1/16:0)	0	4793.124329	12968.58895	25322.8097	29232.44176	0.97403064	0.948736	0.005
PC(32:0),PC(O-33:0)	4440.528313	7528.172543	25218.60168	44530.53121	51274.60723	0.97396336	0.948605	0.00502
SM(d16:1/20:0)	0	5172.20836	31321.06618	61195.812	71006.11227	0.973781656	0.948251	0.00508
SM(d18:2/22:1)	3419.256245	7449.240437	43666.83925	76526.90495	91084.0539	0.973552458	0.947804	0.00514
PC(38:3)	0	9397.432658	47982.73498	92962.67083	107460.0681	0.973324598	0.947361	0.00521
SM(d16:1/24:0)	0	14405.67295	90261.2619	164107.0557	194260.1047	0.973178404	0.947076	0.00525
[TG(51:9),TG(50:2)]_C18:1	3568.336255	5048.512328	21213.47747	36148.73461	42349.86297	0.972841368	0.94642	0.00535
PC(36:3),PC(P-37:2)	9647.98866	14620.26509	97289.74309	169736.6487	201735.5286	0.972508382	0.945773	0.00545
[TG(52:4)]_C16:1	1558.9521	1838.272124	5579.380402	8864.39671	10307.12078	0.972322619	0.945411	0.0055
PC(30:2),PC(P-31:1)	0	1793.500131	7497.400484	13453.07299	15726.64516	0.971662309	0.944128	0.0057
SM(d16:1/17:0)	0	2386.104173	7702.388529	13980.83713	16172.0491	0.970886118	0.94262	0.00594
PC(40:6)	1953.000137	3056.840217	10938.7368	19244.12539	21805.09753	0.970236654	0.941359	0.00614
PC(38:4)	0	12568.42872	74979.53686	139404.5534	159989.1512	0.969352526	0.939644	0.00641
PC(37:7),PC(P-38:6),PC(36:0),PC	0	2255.524162	6945.672515	11410.50098	13633.22493	0.96916489	0.939281	0.00647
[TG(50:7),TG(49:0)]_C16:0	1425.152097	1967.660139	2440.008167	3249.864215	3580.240266	0.96858131	0.93815	0.00665
PC(O-38:8),PC(36:1),PC(O-37:1),	0	10031.10474	54960.24426	101044.0085	115545.8238	0.968330344	0.937664	0.00673
[TG(52:5)]_C18:2	1389.056104	1685.836117	5391.832395	9347.908686	10415.64466	0.968319638	0.937643	0.00674
PC(O-38:9),PC(36:2),PC(O-37:2),	0	27332.64209	170146.1514	300015.9496	348632.6332	0.967985243	0.936995	0.00684
PC(34:0),PC(O-35:0)	2277.344155	5613.852376	21746.28549	35635.95455	41447.27512	0.967974368	0.936974	0.00685
[TG(55:10),TG(54:3)]_C18:1	6605.54846	10866.51275	51573.75594	89940.4186	102265.4914	0.96721347	0.935502	0.00709
[TG(52:4)]_C16:0	3570.30825	6629.348512	30809.18254	51162.43928	59099.62046	0.966649784	0.934412	0.00727
SM(d16:1/22:0)	0	17659.40922	101457.967	179815.4368	206393.5003	0.966398312	0.933926	0.00736
SM(d16:0/20:0)	0	1563.468102	5644.228422	10142.86867	11485.3087	0.966184944	0.933513	0.00743
[TG(52:4)]_C18:2	4876.340355	9146.780582	44269.33531	76906.76954	87187.45404	0.965948201	0.933056	0.0075
[TG(46:0)]_C14:0	1116.640076	2085.076151	3508.328246	5588.020399	6133.424438	0.965935338	0.933031	0.00751
PC(28:1),PC(P-29:0)	0	2296.516148	5990.680396	10337.92881	11877.64092	0.96533523	0.931872	0.00771
[TG(50:3)]_C16:0	1865.400133	2770.55217	9957.564648	17530.67327	19366.7294	0.965256451	0.93172	0.00773
PC(34:2),PC(O-35:2),PC(P-35:1)	18533.08942	36078.81844	233344.2055	408336.0167	465272.8968	0.965085515	0.93139	0.00779
[TG(54:5)]_C18:1	2897.444202	4932.55633	20401.98148	35873.73419	39876.13904	0.964516749	0.930293	0.00798
PC(35:4),PC(O-36:4),PC(P-36:3)	0	2061.00415	13073.83687	22423.26161	25855.44176	0.964344802	0.929961	0.00804
[TG(53:10),TG(52:3)]_C18:1	8504.832582	16520.27328	88118.43884	154167.7069	173276.4409	0.963827523	0.928963	0.00821
[TG(50:3)]_C16:1	1772.73613	2104.504144	7331.080545	12049.34083	13439.2569	0.963798731	0.928908	0.00822
PC(36:4),PC(O-37:4)	9959.976672	15210.2451	119237.2161	209127.1772	237591.0012	0.963628551	0.92858	0.00828
[TG(53:10),TG(52:3)]_C16:1	1128.41208	1811.676118	4645.892341	7981.632613	8635.140569	0.963551176	0.928431	0.00831
[TG(52:4)]_C18:1	1939.480143	2400.448177	10156.62475	16393.4492	18581.81721	0.962669746	0.926733	0.00861
[TG(48:2)]_C16:0	2048.968156	2973.012218	9571.360663	15723.82106	17391.6172	0.962471994	0.926352	0.00868
[TG(52:5)]_C18:3	1251.580083	1602.556123	4549.844302	6949.308492	7762.268568	0.961630848	0.924734	0.00897
PC(40:5)	0	2531.912182	8148.692564	14427.13306	16007.96517	0.960998182	0.923518	0.00919
[TG(54:6)]_C18:2	2125.692152	3393.856239	11734.71678	20818.02954	22428.9616	0.960456621	0.922477	0.00938
[TG(53:10),TG(52:3)]_C18:2	8221.744571	15129.61719	81505.12533	138777.645	154818.7563	0.960225343	0.922033	0.00947
[TG(54:6)]_C20:4	0	1568.256114	3279.54424	5232.204356	6176.220473	0.96000115	0.921602	0.00955
[TG(53:9),TG(52:2)]_C18:2	2323.364159	3252.420242	13236.23299	23841.07367	25672.57769	0.959717954	0.921059	0.00965
PC(30:0),PC(O-31:0)	0	2551.480203	7484.760512	14054.37697	15187.99701	0.959248115	0.920157	0.00981
[TG(54:6)]_C18:3	0	1571.564104	3362.56022	5408.944358	6304.284417	0.958856035	0.919405	0.00996
PC(34:1),PC(O-35:1),PC(P-35:0)	9851.596784	24035.04965	127497.0413	229318.9437	249627.5345	0.958774541	0.919249	0.00999
[TG(49:7),TG(48:0)]_C14:0	1177.540076	1632.976105	2273.036173	3504.372254	3624.636253	0.958424886	0.918578	0.01011
[TG(54:6)]_C18:1	1117.760084	1595.93211	3691.632259	5709.784414	6163.816449	0.958396997	0.918525	0.01012
[TG(53:10),TG(52:3)]_C16:0	8584.480612	16594.98913	93368.51888	162627.4554	178293.6412	0.957837051	0.917452	0.01033

[TG(50:3)]_C14:0	1191.772083	1910.612134	5286.196354	8830.612606	9437.672664	0.957559051	0.916919	0.01043
[TG(54:5)]_C20:4	907.532061	1840.520126	5072.416355	7969.836564	8757.92859	0.957293602	0.916411	0.01053
[TG(50:3)]_C18:1	1764.388122	2505.252181	8089.860571	13970.74095	14871.913	0.957184633	0.916202	0.01057
PC(40:4)	0	1997.724141	5673.400428	8609.440666	10092.92076	0.95674516	0.915361	0.01073
PC(37:5),PC(O-38:5),PC(P-38:4)	0	1919.632142	10139.45664	17144.59325	19023.00532	0.95602318	0.91398	0.011
[TG(55:10),TG(54:3)]_C18:2	1924.688138	2796.604195	11708.66871	18588.2974	20528.56958	0.955034424	0.912091	0.01137
[TG(53:9),TG(52:2)]_C18:1	16478.48528	25888.46568	137331.9289	237320.4165	256596.3567	0.954904753	0.911843	0.01142
[TG(55:9),TG(54:2)]_C18:1	4109.380305	4988.288338	18937.84959	31205.59014	33658.20643	0.954855311	0.911749	0.01144
[TG(53:9),TG(52:2)]_C16:0	10381.74475	16968.05327	86287.57877	153604.4115	164098.7919	0.954713491	0.911478	0.01149
[TG(52:4)]_C18:3	1352.276099	2175.568169	6713.308469	10926.33684	11703.06069	0.954186289	0.910471	0.01169
[TG(54:5)]_C18:3	772.6080528	1688.07212	3880.568296	6320.4524	6720.57248	0.954035696	0.910184	0.01175
[TG(55:8),TG(54:1)]_C18:1	1821.920123	2251.088161	4004.580289	5249.176361	5721.240409	0.953966835	0.910053	0.01177
[TG(39:0)]_C20:0	992.6160759	1890.184138	8196.188583	15023.91309	15714.01704	0.952825912	0.907877	0.01221
PC(35:3),PC(O-36:3),PC(P-36:2)	0	1446.260101	6412.148467	9706.776672	11178.76079	0.95271453	0.907665	0.01226
[TG(55:11),TG(54:4)]_C18:1	5628.800397	10332.00875	50386.76394	90734.81842	95535.93858	0.952290528	0.906857	0.01242
[TG(51:9),TG(50:2)]_C16:1	2565.264186	3127.168212	11487.95282	19323.53345	20440.00148	0.952209387	0.906703	0.01245
PC(38:6)	3699.588273	5162.132378	27420.9859	48750.90713	51535.37183	0.951945176	0.9062	0.01255
[TG(51:9),TG(50:2)]_C14:0	1578.768114	1930.188132	5588.588383	9258.728636	9672.488721	0.951704557	0.905742	0.01265
[TG(53:8),TG(52:1)]_C18:0	5013.828326	5142.324371	17550.92934	30552.91781	31749.67828	0.951384153	0.905132	0.01277
[TG(51:9),TG(50:2)]_C16:0	5740.51642	10255.4287	56489.92792	100601.6347	106197.0242	0.951137796	0.904663	0.01287
[TG(53:8),TG(52:1)]_C16:0	5204.768407	5718.812391	21232.62544	35844.84644	37614.88252	0.950461911	0.903378	0.01314
PC(38:5)	2828.612193	4931.50834	28068.87008	50081.95508	52729.04355	0.95042813	0.903314	0.01315
[TG(52:9),TG(51:2)]_C18:1	1030.980067	1712.992121	3822.248268	5301.320354	5858.376423	0.950149155	0.902783	0.01326
[TG(48:3)]_C18:2	0	1151.508083	3088.456204	4701.952343	5331.408376	0.949328132	0.901224	0.01359
[TG(56:6)]_C20:4	0	1117.70008	2831.200195	4340.460322	4914.584341	0.949320225	0.901209	0.01359
[TG(55:10),TG(54:3)]_C18:0	2163.224155	3117.008228	11210.10075	18792.84933	19675.42127	0.949109833	0.900809	0.01368
PC(36:5)	0	1553.836102	5811.248414	8438.02859	9770.044671	0.948631739	0.899902	0.01387
[TG(48:2)]_C16:1	1512.19611	1934.920137	3807.392271	5210.744354	5571.480366	0.948527049	0.899704	0.01391
PC(35:2),PC(O-36:2),PC(P-36:1)	0	1453.544101	6846.524426	11955.40074	12649.47698	0.948458504	0.899574	0.01394
[TG(52:5)]_C16:0	0	1627.860113	4330.568326	7029.496553	7652.828522	0.947535786	0.897824	0.01431
[TG(55:11),TG(54:4)]_C18:2	3641.124249	6451.636472	30103.45817	51607.97935	53729.60755	0.946011665	0.894938	0.01494
[TG(55:9),TG(54:2)]_C18:0	2987.444226	3315.344223	11260.79691	19246.48933	19670.80143	0.945632079	0.89422	0.01509
[TG(51:9),TG(50:2)]_C18:2	2828.200195	4636.776357	23993.16167	41785.69905	43293.85123	0.945321445	0.893633	0.01522
[TG(49:8),TG(48:1)]_C18:1	2905.192203	3821.908254	15386.08518	25821.59795	26659.61368	0.944208546	0.89153	0.01569
[TG(55:11),TG(54:4)]_C18:0	1459.236105	1938.608142	5425.408421	8749.800663	8960.024686	0.943979155	0.891097	0.01578
[TG(53:8),TG(52:1)]_C18:1	5499.960375	5806.04044	23150.82177	39993.79873	40655.9908	0.94281106	0.888893	0.01628
[TG(50:4)]_C14:0	0	1112.87208	2560.416183	3687.408265	4253.012293	0.942730827	0.888741	0.01631
[TG(49:8),TG(48:1)]_C16:0	3844.932261	5118.976361	20747.37339	34897.93852	35817.03874	0.942680792	0.888647	0.01633
[TG(51:8),TG(50:1)]_C16:0	12410.16898	16561.00513	93019.20296	172726.0752	173829.088	0.942265014	0.887863	0.01651
[TG(54:9),TG(53:2)]_C18:1	1142.896084	1460.052102	3061.676217	4129.712293	4396.004336	0.942050199	0.887459	0.0166
[TG(51:7)]_C18:1	924.984063	1676.436109	4443.132314	6664.780499	6993.180455	0.941326029	0.886095	0.01691
[TG(51:8),TG(50:1)]_C18:1	7002.004496	9309.800601	47638.80346	87487.5899	87561.54621	0.940693508	0.884904	0.01718
[TG(46:1)]_C18:1	0	1672.192115	4176.880283	6710.932443	7110.968481	0.938562636	0.8809	0.01811
PC(29:1),PC(O-30:1),PC(P-30:0)	0	1189.468081	3549.652264	5952.684389	6148.192442	0.938352924	0.880506	0.0182
PC(31:1),PC(O-32:1),PC(P-32:0)	0	1296.660091	3940.932278	6735.23648	6882.408476	0.937582958	0.879062	0.01854
[TG(53:10),TG(52:3)]_C18:0	0	854.2920639	2216.428162	3266.05623	3601.856216	0.937191591	0.878328	0.01872
[TG(55:9),TG(54:2)]_C18:2	0	870.3800575	2652.680191	4401.172314	4541.900333	0.936995198	0.87796	0.0188
[TG(48:2)]_C18:1	1799.968128	2145.544146	6520.264445	9304.2967	9800.632693	0.936159436	0.876394	0.01918
[TG(52:5)]_C16:1	0	797.2200562	2174.284161	3059.508211	3385.092231	0.930750847	0.866297	0.02165
[TG(44:0),TG(O-45:0)]_C16:0	0	2371.172169	4830.596367	8320.160632	8434.620634	0.930615973	0.866046	0.02171
[TG(46:1)]_C16:0	0	1966.276137	4959.928346	8028.492583	8222.172603	0.930084223	0.865057	0.02196
[TG(56:7)]_C20:4	0	853.9400558	2660.372198	4314.672312	4364.364279	0.928590009	0.862279	0.02266
[TG(51:8),TG(50:1)]_C16:1	0	1215.336091	3316.812237	4464.716301	5044.748363	0.927926131	0.861047	0.02297
[TG(55:11),TG(54:4)]_C16:0	0	907.8040604	3362.996256	4753.912356	5131.09237	0.926403778	0.858224	0.0237
[TG(52:10),TG(51:3)]_C18:2	0	1068.212072	2225.560178	3053.244244	3419.944235	0.922708081	0.85139	0.02549
[TG(54:10),TG(53:3)]_C18:1	0	830.1000595	2885.7842	3738.128278	4222.864306	0.921715	0.849559	0.02598
[TG(46:1)]_C14:0	0	1150.95209	2669.216196	4035.264253	4150.412289	0.919280191	0.845076	0.02719
[TG(46:2)]_C18:2	0	1339.368098	2441.608158	3771.904265	3929.084297	0.918310644	0.843294	0.02768
[TG(50:3)]_C18:3	0	868.5720634	3052.244212	4509.664302	4585.012356	0.916420219	0.839826	0.02864
[TG(57:12),TG(56:5)]_C18:1	0	770.1960518	2181.388156	3284.324233	3306.352232	0.915175676	0.837547	0.02928
[TG(54:7)]_C18:2	0	1176.704082	2855.216192	4273.008302	4314.544326	0.912597303	0.832834	0.03061
[TG(50:4)]_C16:0	0	787.0960556	1859.64013	2481.188169	2690.8522	0.910916139	0.829768	0.03149
[TG(55:9),TG(54:2)]_C16:0	0	1082.884077	2740.916203	3827.608278	3971.076266	0.90861729	0.825585	0.0327

[TG(52:9),TG(51:2)]_C16:0	0	1208.600084	3059.056223	3995.828288	4318.464288	0.906519089	0.821777	0.03382
[TG(50:4)]_C16:1	0	1071.288074	1977.392141	2805.176203	2957.900215	0.905389743	0.819731	0.03443
[TG(53:8)]_C18:2	0	771.1800499	1595.184107	2312.956165	2334.272155	0.901134577	0.812044	0.03676
[TG(48:3)]_C16:0	0	827.0280556	2183.464154	3100.036211	3103.004205	0.900955909	0.811722	0.03686
[TG(42:0)]_C16:0	0	1304.79609	2409.800172	3606.900254	3624.752254	0.900849637	0.81153	0.03692
[TG(52:4)]_C20:4	0	817.8480598	2770.584197	3812.236285	3814.532265	0.89702222	0.804649	0.03905
LPG(20:0); LPG(20:0)	1967.748134	2253.976158	2780.424199	3061.740221	3078.076225	0.894989031	0.801005	0.0402
[TG(53:9),TG(52:2)]_C16:1	0	1181.444078	2493.708161	3056.832204	3416.104257	0.893612999	0.798544	0.04098
[TG(52:8),TG(51:1)]_C16:0	0	1608.244109	3040.872212	4293.65228	4295.016279	0.887050872	0.786859	0.04479
[TG(48:3)]_C16:1	0	912.6480713	1864.384129	2267.860158	2489.588172	0.883154258	0.779961	0.0471
LPC(20:2),PC(O-20:2)	0	1493.892114	2167.376157	2216.392172	2380.476158	0.7461989	0.556813	0.14751
[TG(51:7),TG(50:0)]_C18:0	4869.872356	4760.792327	6676.452477	9358.344637	10532.23675	0.98555566	0.97132	0.00208
CE(20:1) NH4	0	0	3487.108242	5231.768407	8447.360639	0.985064564	0.970352	0.00219
CE(16:0)K	0	0	9074.364646	20247.20146	24472.47373	0.980151895	0.960698	0.00335
LPC(18:1),PC(O-18:1),PC(P-18:0)	0	0	4765.464307	9646.72467	11896.28078	0.979639548	0.959694	0.00348
[TG(49:7),TG(48:0)]_C16:0	10629.94082	10423.15671	20659.56158	32365.36634	36535.25848	0.976711176	0.953965	0.00425
[TG(49:6)]_C16:0	1645.720114	1622.908113	2096.316143	3092.216228	3321.940217	0.976207992	0.952982	0.00439
SM(d18:1/24:1(15Z))	0	0	68912.95348	134934.789	161983.1121	0.974338245	0.949335	0.00492
LPC(16:0),PC(O-16:0),LPC(O-17:	0	0	51364.63128	94891.09477	115949.3561	0.973757503	0.948204	0.00508
SM(d18:0/24:1)	0	0	28746.82187	54652.09948	65945.01252	0.973549288	0.947798	0.00514
[TG(56:12),TG(55:5)]_C18:1	0	0	766.9360573	1103.836079	1583.480116	0.973214724	0.947147	0.00524
PC(O-40:9),PC(38:2),PC(P-39:1)	0	0	33238.07021	65934.48477	77542.47316	0.971794195	0.944384	0.00566
SM(d18:2/24:1)	0	0	48783.74346	95924.40331	111534.6769	0.969471483	0.939875	0.00637
PC(38:9),PC(37:2),PC(O-38:2),PC	0	0	8835.492649	14817.86116	18294.57726	0.968278759	0.937564	0.00675
PI(38:4)	0	0	2411.52018	6497.400494	7099.38047	0.967835857	0.936706	0.00689
PC(32:1),PC(O-33:1),PC(P-33:0)	0	0	16754.80918	30435.34218	35834.04254	0.966703672	0.934516	0.00726
SM(d18:1/26:1(17Z))	0	0	1260.292088	2740.976207	3036.124213	0.965538581	0.932265	0.00764
PC(39:8),PC(O-40:8),PC(38:1),PC	0	0	14771.1929	26192.56981	30838.18624	0.964914306	0.93106	0.00785
SM(d18:1/17:0)	0	0	4763.43233	8287.984587	9837.500657	0.964825833	0.930889	0.00788
PC(38:7),PC(37:0),PC(O-38:0)	0	0	1653.052113	3200.436224	3596.460242	0.962502784	0.926412	0.00867
SM(d16:1/18:1)	0	0	15705.2651	30398.43022	34137.98651	0.962364739	0.926146	0.00871
PC(16:0),PC(O-17:0),LPC(O-18:0	0	0	1621.032117	3559.120256	3861.716288	0.96200824	0.92546	0.00884
PC(35:5),PC(O-36:5),PC(P-36:4)	0	0	8888.748602	13704.20103	17107.02511	0.961825478	0.925108	0.0089
PC(38:8),PC(37:1),PC(O-38:1),PC	0	0	9910.784666	16578.82124	19668.82139	0.961301707	0.924101	0.00909
[TG(51:7),TG(50:0)]_C16:0	8468.344572	7571.424535	14707.68111	23980.78967	25281.58979	0.961018236	0.923556	0.00918
SM(d16:1/25:0)	0	0	15525.00516	28158.2659	32084.23427	0.960658676	0.922865	0.00931
[TG(44:2)]_C18:2	0	0	954.1560669	1613.668108	1888.564125	0.959818881	0.921252	0.00961
SM(d16:0/24:0)	0	0	15839.56106	25793.7818	30780.70217	0.959749128	0.921118	0.00964
[TG(45:0)]_C16:0	0	0	1056.73207	1737.43213	2057.856143	0.959334428	0.920323	0.00978
PC(32:2),PC(O-33:2),PC(P-33:1)	0	0	6983.744476	11383.10882	13501.713	0.95872569	0.919155	0.01
PC(35:6),PC(P-36:5)	0	0	1988.988141	3099.724209	3749.116283	0.957495797	0.916798	0.01045
[TG(48:3)]_C14:0	0	0	1364.948102	1994.204149	2519.196172	0.957427112	0.916667	0.01048
PC(36:8),PC(35:1),PC(O-36:1),PC	0	0	9378.728617	15181.62108	17900.10928	0.956888169	0.915635	0.01068
SM(d16:1/22:1)	0	0	8670.87261	14153.80504	16581.64919	0.956536794	0.914963	0.01081
SM(d18:0/24:0)	0	0	4582.164315	6782.892473	8401.488634	0.955626814	0.913223	0.01115
SM(d16:1/23:0)	0	0	7545.664545	13529.97694	14968.26503	0.953856189	0.909842	0.01182
PC(37:3),PC(O-38:3),PC(P-38:2)	0	0	9794.720818	15897.88111	18418.38128	0.953748937	0.909637	0.01186
[TG(52:6)]_C18:3	0	0	1106.284076	1838.924133	2101.004145	0.953443813	0.909055	0.01197
PC(37:6),PC(O-38:6),PC(P-38:5)	0	0	4392.764333	6461.428442	7898.236519	0.952353496	0.906977	0.0124
[TG(54:11),TG(53:4)]_C16:0	0	0	876.8000679	1500.700101	1677.264126	0.951797374	0.905918	0.01261
SM(d16:1/20:1)	0	0	10906.1128	20611.32148	22072.74555	0.951058305	0.904512	0.0129
[TG(53:7),TG(52:0)]_C20:0	0	0	946.7360634	1518.164106	1746.052123	0.950924936	0.904258	0.01295
SM(d17:1/24:1)	0	0	11039.58886	20293.86143	21907.07345	0.950665695	0.903765	0.01306
[TG(59:10),TG(58:3)]_C18:2	0	0	1237.460086	1798.12012	2192.58016	0.95022672	0.902931	0.01323
PC(33:3),PC(O-34:3),PC(P-34:2)	0	0	5122.908369	8154.508573	9372.148661	0.949934508	0.902376	0.01335
PC(37:4),PC(O-38:4),PC(P-38:3)	0	0	9133.82068	14722.76505	16776.20919	0.949574665	0.901692	0.01349
[TG(44:1)]_C16:0	1255.18809	1088.532074	2039.544156	2882.512199	3017.764196	0.949482279	0.901517	0.01353
[TG(55:8),TG(54:1)]_C20:0	0	0	2573.580165	4025.748282	4660.632284	0.949376341	0.901315	0.01357
SM(d17:1/26:1)	0	0	4619.160316	6781.44055	8138.280631	0.948501456	0.899655	0.01392
PC(40:3)	0	0	2659.296198	4218.400273	4821.66037	0.948371003	0.899408	0.01397
PC(33:2),PC(O-34:2),PC(P-34:1)	0	0	6263.368442	10234.96478	11500.48081	0.948127593	0.898946	0.01407
[TG(55:7),TG(54:0)]_C20:0	0	0	806.1960632	1423.172102	1540.632104	0.947790535	0.898307	0.01421
[TG(51:8),TG(50:1)]_C18:0	1809.844135	1800.860128	4057.476288	6938.760529	6971.812502	0.947467997	0.897696	0.01434

SM(d16:0/23:0)	0	0	3648.972234	5346.844393	6379.100405	0.947045699	0.896896	0.01451
[TG(53:7)]_C18:1	891.1760651	830.5640579	2339.060165	3110.504208	3489.824237	0.946689461	0.896221	0.01466
PC(28:2)	0	0	1155.700076	1223.636081	2074.808141	0.946543995	0.895946	0.01472
PG(O-35:1),PG(P-35:0); PG(O-35:	0	0	1008.70407	1521.696109	1765.868126	0.945243845	0.893486	0.01525
PC(42:3)	0	0	1111.372074	2101.916149	2192.068153	0.945126372	0.893264	0.0153
PC(40:10),PC(39:3),PC(O-40:3),F	0	0	6198.904443	10001.61271	11143.55274	0.944814987	0.892675	0.01543
[TG(57:9),TG(56:2)]_C18:2	0	0	771.0120583	1607.388112	1624.816103	0.944314661	0.89173	0.01564
[TG(54:7)]_C18:3	0	0	2116.368137	3054.016217	3626.284264	0.943855206	0.890863	0.01583
[TG(57:9),TG(56:2)]_C18:0	0	0	776.7520576	1448.884104	1508.844101	0.943686659	0.890545	0.01591
PC(39:4),PC(O-40:4),PC(P-40:3)	0	0	4797.232339	7559.880514	8472.308606	0.943265602	0.88975	0.01608
PC(39:5),PC(O-40:5),PC(P-40:4)	0	0	5185.292362	7294.020494	8794.060622	0.943200366	0.889627	0.01611
[TG(54:5)]_C16:0	0	0	3277.144223	4802.296343	5617.696419	0.942914011	0.889087	0.01623
PC(42:8),PC(41:1),PC(O-42:1),PC	0	0	1260.184085	2527.528185	2554.764178	0.94192088	0.887215	0.01665
SM(d16:0/25:0)	0	0	3731.860289	5869.504424	6536.764431	0.941598068	0.886607	0.01679
[TG(55:8),TG(54:1)]_C18:0	3290.632232	2330.508157	5120.072341	7104.860485	7636.33652	0.94057599	0.884683	0.01723
PC(31:0),PC(O-32:0)	0	0	5123.964359	7442.212534	8660.192655	0.94045992	0.884465	0.01728
SM(d18:1/25:0)	0	0	3007.924222	4353.848314	5075.716346	0.940351581	0.884261	0.01733
[TG(49:7)]_C16:1	0	0	799.416053	1523.400112	1539.800103	0.938187856	0.880196	0.01828
SM(d18:0/17:0)	0	0	3158.380212	4876.612347	5403.796391	0.938012749	0.879868	0.01835
PC(33:1),PC(O-34:1),PC(P-34:0)	0	0	5213.912375	8372.220564	9068.884634	0.937711204	0.879302	0.01849
[TG(57:11),TG(56:4)]_C18:0	0	0	942.7360741	1529.37611	1638.132111	0.936262176	0.876587	0.01913
[TG(55:10),TG(54:3)]_C16:0	0	0	2641.400176	4169.652283	4529.588304	0.936223539	0.876515	0.01915
[TG(49:8),TG(48:1)]_C14:0	1881.692139	2600.296191	8383.17664	14580.59301	14342.68504	0.93620699	0.876484	0.01916
[TG(46:0)]_C16:0	3612.628268	4428.144323	11610.76884	18379.53332	18346.20938	0.935970689	0.876041	0.01926
SM(d18:0/26:1(17Z))	0	0	2082.312148	2795.10019	3370.964246	0.935542231	0.875239	0.01945
PC(40:2)	0	0	3253.832227	5157.044354	5567.932384	0.935255566	0.874703	0.01958
[TG(55:9),TG(54:2)]_C20:0	0	0	1888.012132	2924.628207	3196.328233	0.935158991	0.874522	0.01963
[TG(54:11),TG(53:4)]_C18:2	0	0	1851.97614	2565.472194	3007.816232	0.934625244	0.873524	0.01987
[TG(51:4)]_C18:2	0	0	1191.732083	1841.196126	2010.020145	0.934528404	0.873343	0.01991
[TG(57:8),TG(56:1)]_C18:1	0	0	1096.900082	1799.73612	1893.020139	0.933148821	0.870767	0.02054
[TG(57:10),TG(56:3)]_C18:2	0	0	1498.900108	2280.752158	2493.124168	0.932698151	0.869926	0.02075
[TG(50:4)]_C18:2	1073.708073	1789.640127	4988.264339	8075.700549	7988.18055	0.932260529	0.86911	0.02095
[TG(48:2)]_C14:0	867.8120651	1858.572137	5183.872369	8645.736594	8496.180574	0.931613083	0.867903	0.02125
[TG(50:9),TG(49:2)]_C16:0	0	0	1820.356125	2985.052209	3121.232219	0.931578736	0.867839	0.02126
PC(33:0),PC(O-34:0)	0	0	2826.648196	4461.876322	4738.20031	0.930590222	0.865998	0.02172
[TG(48:3)]_C18:3	0	0	1301.504099	2107.700147	2202.256149	0.929817649	0.864561	0.02208
[TG(56:8)]_C22:6	0	0	1184.59608	1902.864127	1995.148136	0.929660108	0.864268	0.02216
[TG(54:10),TG(53:3)]_C18:2	0	0	1849.500127	2951.104234	3100.996242	0.929339864	0.863673	0.02231
PC(40:8),PC(39:1),PC(O-40:1),PC	0	0	3313.660221	4752.584351	5313.320372	0.929123216	0.86327	0.02241
[TG(48:2)]_C18:2	1668.920119	2308.492159	8000.9686	14177.89296	13583.96091	0.928723259	0.862527	0.0226
[TG(57:9),TG(56:2)]_C20:0	0	0	1977.94014	3068.800197	3263.836229	0.928592616	0.862284	0.02266
SM(d18:1/19:0)	0	0	5090.936351	8559.576571	8732.764702	0.928235848	0.861622	0.02283
PC(40:9),PC(39:2),PC(O-40:2),PC	0	0	4105.54428	6415.412437	6778.38049	0.927902598	0.861003	0.02299
[TG(53:9),TG(52:2)]_C18:0	2167.512156	3090.104228	12024.99288	20990.47342	20146.08942	0.926675882	0.858728	0.02357
SM(d18:2/18:1)	0	0	1467.060112	2161.768151	2344.016164	0.925795555	0.857097	0.02399
PC(39:6),PC(O-40:6),PC(P-40:5)	0	0	3204.288224	3957.664287	4899.228407	0.925427268	0.856416	0.02417
[TG(44:1)]_C14:0	0	0	1101.904082	1516.696101	1714.26812	0.92474682	0.855157	0.0245
[TG(54:6)]_C16:0	0	0	1862.268134	2627.272197	2909.388206	0.9239418	0.853668	0.02489
[TG(57:10),TG(56:3)]_C18:1	0	0	2547.984175	3512.004257	3944.556283	0.923410308	0.852687	0.02515
PC(39:7),PC(P-40:6),PC(38:0),PC	0	0	3746.452261	6021.944428	6123.356425	0.921607719	0.849361	0.02604
PC(44:12),PC(O-44:5)	0	0	1939.712141	2786.396213	3005.304195	0.92019106	0.846752	0.02674
PC(36:7),PC(35:0),PC(O-36:0)	0	0	2484.116184	3509.808242	3824.564277	0.920016052	0.84643	0.02683
CE(22:5)H	0	0	4735.824338	7370.300543	7558.652528	0.919359596	0.845222	0.02715
SM(d16:0/16:0)	0	0	2956.260217	4122.688285	4496.740335	0.917918863	0.842575	0.02788
[TG(37:0)]_C18:0	0	0	2622.088187	3378.668238	3908.820276	0.917868883	0.842483	0.0279
[TG(49:7),TG(48:0)]_C18:0	1656.860112	1208.116079	3461.800236	4594.760319	4769.780321	0.91705417	0.840988	0.02832
PC(42:11),PC(41:4),PC(O-42:4)	0	0	2367.968163	3402.120253	3621.264246	0.916661882	0.840269	0.02852
[TG(50:4)]_C18:3	0	0	2110.740152	2858.448207	3161.73621	0.916122049	0.83928	0.02879
[TG(57:11),TG(56:4)]_C20:0	0	0	1188.340084	1502.848109	1750.972127	0.915735875	0.838572	0.02899
SM(d18:2/21:0)	0	0	3542.436244	5376.084364	5505.484364	0.915099565	0.837407	0.02931
[TG(52:6)]_C18:2	0	0	1176.508092	1663.356107	1777.960128	0.914947474	0.837129	0.02939
[TG(57:12),TG(56:5)]_C18:2	0	0	1327.248089	2066.244133	2082.276157	0.914510711	0.83633	0.02962
[TG(52:5)]_C18:1	0	0	1648.372112	2187.196154	2438.024188	0.914148062	0.835667	0.0298

[TG(59:9),TG(58:2)]_C18:1	0	0	1707.436109	2380.368168	2559.756194	0.91402041	0.835433	0.02987
[TG(56:7)]_C18:2	0	0	1776.148124	2393.460162	2632.176189	0.913604039	0.834672	0.03009
[TG(56:6)]_C16:0	0	0	1373.428098	2007.280137	2082.064137	0.912459054	0.832582	0.03068
PC(40:1),PC(P-41:0)	0	0	2234.324159	3152.212222	3332.844223	0.911532735	0.830892	0.03116
[TG(57:12),TG(56:5)]_C20:4	0	0	1973.560149	2429.424167	2835.032219	0.910185077	0.828437	0.03187
[TG(48:4)]_C18:2	0	0	1516.532109	2046.084142	2206.488145	0.908439707	0.825263	0.0328
[TG(46:2)]_C18:1	784.0920494	783.7480598	1828.836132	2320.772149	2346.256184	0.907769804	0.824046	0.03315
[TG(52:10),TG(51:3)]_C16:0	0	0	1688.196118	2143.988172	2405.788171	0.906657829	0.822028	0.03375
PC(41:5),PC(P-42:4)	0	0	2768.944202	4042.448281	4097.312309	0.905602693	0.820116	0.03432
[TG(44:1)]_C18:1	0	0	1999.140137	2466.640178	2795.892197	0.902847813	0.815134	0.03582
[TG(50:4)]_C18:1	0	0	1847.268127	2330.908163	2583.316178	0.901732485	0.813121	0.03643
[TG(46:0)]_C18:0	0	0	1680.512124	2209.780154	2369.016169	0.901379886	0.812486	0.03662
PC(42:10),PC(41:3),PC(O-42:3),F	0	0	2066.46414	2828.152192	2926.968236	0.899422927	0.808962	0.03771
[TG(54:9),TG(53:2)]_C16:0	0	0	1510.604102	1962.75214	2106.17215	0.89903159	0.808258	0.03792
PC(40:7),PC(39:0),PC(O-40:0)	0	0	3733.252244	5139.356375	5285.900388	0.898753812	0.807758	0.03808
[TG(48:7),TG(47:0)]_C16:0	0	0	2809.068201	3618.264252	3898.780285	0.898297224	0.806938	0.03833
[TG(52:9),TG(51:2)]_C18:2	0	0	1850.780128	2590.392185	2627.020187	0.897828455	0.806096	0.0386
[TG(55:8),TG(54:1)]_C16:0	0	0	1760.120124	2296.308154	2433.700168	0.896270745	0.803301	0.03947
[TG(57:9),TG(56:2)]_C18:1	0	0	1996.776145	2770.920187	2809.908188	0.896092241	0.802981	0.03957
[TG(56:7)]_C22:6	0	0	1809.448124	2122.888154	2449.968179	0.895501312	0.801923	0.03991
PC(41:6),PC(O-42:6)	0	0	2076.64015	2686.012202	2825.244204	0.892036198	0.795729	0.04189
[TG(48:3)]_C18:1	0	0	2359.752169	3166.424223	3237.368246	0.891549955	0.794861	0.04217
[TG(49:8)]_C18:2	0	0	1495.66411	1736.708126	1988.412142	0.890517549	0.793022	0.04276
Cer(d18:1/24:0)	0	0	2426.464166	3021.152202	3252.788235	0.890084056	0.79225	0.04302
[TG(52:5)]_C20:4	0	0	1789.016129	2267.340179	2404.08417	0.88971207	0.791588	0.04323
[TG(46:2)]_C16:1	0	0	1532.568107	1906.208131	2038.156152	0.88771661	0.788041	0.0444
[TG(50:8),TG(49:1)]_C18:1	0	1452.912101	3130.944231	4474.636338	4365.200307	0.887310223	0.787319	0.04464
[TG(49:8),TG(48:1)]_C16:1	0	2168.176153	4544.140318	7486.712516	6665.336488	0.880324809	0.774972	0.0488
[TG(54:5)]_C22:5	0	0	1539.376114	1667.880107	1966.992142	0.879763289	0.773983	0.04914
[TG(42:0)]_C14:0	0	0	1501.868103	1917.228136	1955.216134	0.879225886	0.773038	0.04946
[TG(57:12),TG(56:5)]_C18:0	0	0	1461.312103	1830.740126	1879.676134	0.876735435	0.768665	0.05098
[TG(56:6)]_C22:5	0	0	1957.764146	2194.264153	2473.752168	0.87604683	0.767458	0.0514
SM(d18:0/15:0)	0	0	2239.724168	2673.504191	2843.98421	0.875471991	0.766451	0.05175
[TG(55:11),TG(54:4)]_C20:4	0	0	1919.064142	2405.040188	2455.304169	0.87496971	0.765572	0.05206
[TG(46:1)]_C16:1	0	1285.780091	2128.256142	3374.720261	3114.448226	0.873640534	0.763248	0.05289
[TG(59:10),TG(58:3)]_C18:1	0	0	1583.544106	1917.716138	1997.88014	0.872627172	0.761478	0.05351
[TG(56:8)]_C20:4	0	0	1813.312132	2245.308167	2290.000154	0.871544428	0.75959	0.05419
[TG(50:4)]_C20:4	0	0	1562.092109	1957.676147	1961.608138	0.868790445	0.754797	0.05592
[TG(56:10),TG(55:3)]_C18:1	0	0	1374.5561	1696.076115	1711.440118	0.867062095	0.751797	0.05701
[TG(54:8),TG(53:1)]_C18:1	854.8200623	0	1352.308097	1811.344135	2101.90416	0.865576699	0.749223	0.05795
[TG(54:5)]_C18:0	0	0	1655.252112	1972.784138	2038.56813	0.865562276	0.749198	0.05796
[TG(50:8),TG(49:1)]_C16:0	0	1699.54812	3649.780247	5336.928411	4857.844357	0.865378402	0.74888	0.05808
[TG(54:10),TG(53:3)]_C16:0	0	0	1614.672113	1969.372142	1993.73215	0.865045452	0.748304	0.05829
[TG(56:7)]_C22:5	0	0	1560.868116	1801.336136	1905.256138	0.864109599	0.746685	0.05889
[TG(57:12),TG(56:5)]_C16:0	0	0	1550.444111	1833.860137	1894.92013	0.863402226	0.745463	0.05935
[TG(57:11),TG(56:4)]_C18:2	0	771.0560516	2078.484147	2633.552177	2562.440192	0.861680566	0.742493	0.06046
[TG(44:2)]_C16:0	0	0	1464.24811	1694.872127	1762.364127	0.859272244	0.738349	0.06202
[TG(51:6)]_C16:0	0	0	1274.956091	1277.72809	1527.340109	0.859159166	0.738154	0.06209
[TG(49:8),TG(48:1)]_C18:0	0	823.5560631	1794.412132	2301.940161	2241.132167	0.857325826	0.735008	0.06329
[TG(59:11),TG(58:4)]_C18:2	0	0	819.1760582	902.9480682	971.3080635	0.855844456	0.73247	0.06426
PC(28:0),PC(O-29:0)	0	1654.832127	2852.964201	3566.372258	3559.012263	0.840148786	0.70585	0.07485
[TG(50:7),TG(49:0)]_C18:0	916.2360672	0	986.1040695	1436.5841	1701.264121	0.818865128	0.67054	0.08999
[TG(56:8),TG(55:1)]_C16:0	0	0	1051.148072	1071.084071	1104.488075	0.813835677	0.662329	0.09368
PS(O-29:0)	8013.78857	9092.516756	9912.968709	9000.556711	11571.04082	0.809538011	0.655352	0.09688
DG(36:5)_C16:0	0	2601.684193	0	2964.904218	3011.544222	0.634808959	0.402982	0.2499
CAR(14:1)	0	3486.11225	0	3699.520274	3727.604277	0.596091716	0.355325	0.28875
CAR(20:0)	3140.588212	0	1868.880136	2617.608189	3377.320253	0.524039107	0.274617	0.36471
CE(22:3)H	0	0	0	2240.872147	3764.420255	0.967900568	0.936832	0.00687
PC(32:3),PC(P-33:2)	0	0	0	1200.220084	1941.684136	0.967678926	0.936403	0.00694
1-O-tricosanoyl-Cer(d18:1/16:0)	0	0	0	940.5800636	1400.968097	0.965626909	0.932435	0.00761
PC(41:7),PC(P-42:6),PC(40:0),PC	0	0	0	1479.788107	2155.564159	0.964679222	0.930606	0.00793
[TG(42:1)]_C16:0	0	0	0	911.0920662	1323.068089	0.9645339	0.930326	0.00797
CE(20:2)K	0	0	0	3680.748261	5259.84034	0.963721942	0.92876	0.00825

[TG(50:8),TG(49:1)]_C14:0	0	0	0	928.2600677	1297.21209	0.962438143	0.926287	0.00869
[TG(46:3)]_C18:1	0	0	0	826.200061	1143.17208	0.961807268	0.925073	0.00891
PC(42:6)	0	0	0	1569.416115	2130.928149	0.960506441	0.922573	0.00937
PC(44:10),PC(O-44:3)	0	0	0	1038.884068	1378.560101	0.958739764	0.919182	0.01
CE(22:5) NH4	0	0	0	3433.776254	4541.216325	0.958464264	0.918654	0.0101
SM(d18:1/12:0)	0	0	0	1617.096103	2134.664147	0.95831003	0.918358	0.01015
PC(34:6)	0	0	0	1235.50008	1626.896111	0.95810202	0.917959	0.01023
[TG(51:6)]_C18:0	0	0	0	1260.476089	1658.308113	0.958026777	0.917815	0.01026
[TG(62:16),TG(61:9),TG(60:2)]_C1	0	0	0	1095.800076	1411.820098	0.956168615	0.914258	0.01094
CE(22:4)Na	0	0	0	3712.480256	4767.980334	0.955871651	0.913691	0.01105
DG(39:7)_C18:1	0	0	0	6591.992476	8455.228554	0.955749441	0.913457	0.0111
[TG(54:8),TG(53:1)]_C16:0	0	0	0	1156.84808	1447.124095	0.953250533	0.908687	0.01205
[TG(54:8),TG(53:1)]_C18:0	0	0	0	1292.992091	1567.936115	0.949799272	0.902119	0.0134
[TG(53:8),TG(52:1)]_C20:0	0	0	0	1282.020086	1543.268107	0.948927472	0.900463	0.01375
[TG(54:9),TG(53:2)]_C18:0	0	0	0	1300.540092	1555.43211	0.94813775	0.898965	0.01407
[TG(52:9),TG(51:2)]_C16:1	0	0	0	941.728067	1107.924075	0.946059992	0.89503	0.01492
CE(22:6) NH4	0	0	0	18329.19324	21328.90559	0.944614275	0.892296	0.01552
LPC(18:2),LPC(P-19:1)	0	0	0	4584.516328	5201.392377	0.941085365	0.885642	0.01701
PC(34:3),PC(P-35:2)	0	0	0	11976.9649	13505.40898	0.940190883	0.883959	0.0174
[TG(56:7),TG(55:0)]_C16:0	0	0	0	1539.988107	1731.704131	0.939781539	0.883189	0.01758
[TG(47:2)]_C18:2	0	0	0	1007.748067	1130.148079	0.93937995	0.882435	0.01775
PC(43:6)	0	0	0	2365.300173	2630.488203	0.938117157	0.880064	0.01831
CE(19:0)Na	0	0	0	4194.928329	4656.920335	0.937843818	0.879551	0.01843
CE(20:0) NH4	0	0	15214.91714	28725.53017	28552.606	0.933175541	0.870817	0.02053
CE(20:5) NH4	0	0	0	12721.5768	13704.41702	0.933050469	0.870583	0.02058
[TG(48:8),TG(47:1)]_C14:0	0	0	753.3360527	1496.992115	1463.72011	0.932949701	0.870395	0.02063
[TG(57:8),TG(56:1)]_C16:0	0	0	0	1560.396108	1678.040117	0.932763187	0.870047	0.02072
Cer(d18:1/23:0)	0	0	0	1802.044131	1935.456138	0.932552514	0.869654	0.02081
PC(42:9),PC(41:2),PC(O-42:2),PC	0	0	1158.780083	2313.784154	2242.356163	0.930975362	0.866715	0.02154
Cer(d18:1/22:0)	0	0	0	1808.800128	1912.548138	0.929894235	0.864703	0.02205
[TG(41:0)]_C16:0	0	0	0	823.3640572	869.9640656	0.929769694	0.864472	0.02211
[TG(47:6)]_C16:0	0	0	983.6680733	1954.292136	1887.280147	0.929669321	0.864285	0.02215
[TG(56:11),TG(55:4)]_C18:2	0	0	0	1091.416073	1146.596075	0.928769898	0.862614	0.02258
CE(16:1) NH4	0	0	0	16445.53314	17220.85305	0.928196412	0.861549	0.02285
[TG(53:8),TG(52:1)]_C16:1	0	0	0	843.2880577	882.7760605	0.928142596	0.861449	0.02287
CE(18:2) NH4	0	0	0	1216041.391	1269357.343	0.927636052	0.860509	0.02311
PC(31:2),PC(O-32:2),PC(P-32:1)	0	0	0	1996.340141	2074.896152	0.926863933	0.859077	0.02348
DG(39:8),DG(O-40:8),DG(38:1)_C	0	0	0	143950.5864	149415.5674	0.92662368	0.858631	0.0236
PI(36:2),PI(O-37:2),PI(P-37:1)	0	0	0	2852.716195	2926.704208	0.924494891	0.854691	0.02462
[TG(44:1)]_C16:1	0	0	942.7240715	1775.420115	1706.228115	0.923948717	0.853681	0.02489
CE(20:0)H	0	0	0	3819.376273	3897.85228	0.923515537	0.852881	0.0251
[TG(56:12),TG(55:5)]_C18:2	0	0	0	1139.764085	1161.252077	0.923204418	0.852306	0.02525
Cer(d18:1/24:1(15Z))	0	0	0	1068.320075	1083.300078	0.922308276	0.850653	0.02569
PC(42:4)	0	0	1080.536076	2226.552155	2071.864156	0.922173087	0.850403	0.02576
[TG(57:8),TG(56:1)]_C20:0	0	0	0	1576.976113	1590.08411	0.921232194	0.848669	0.02622
LPC(20:4)	0	0	0	2724.340188	2744.740195	0.921075369	0.84838	0.0263
PC(42:0)	0	0	0	1934.596135	1944.652145	0.920637519	0.847573	0.02652
[TG(53:7),TG(52:0)]_C16:0	2888.320203	2377.564156	4341.124311	5765.988409	5745.540427	0.917379624	0.841585	0.02815
CE(22:6)Na	0	0	1615.384119	2850.744195	2710.800187	0.914346109	0.836029	0.0297
[TG(51:8)]_C18:2	0	0	2767.940207	4518.672334	4391.068305	0.91088781	0.829717	0.0315
[TG(57:10),TG(56:3)]_C20:0	0	0	1984.088136	3004.348217	2997.124242	0.907192418	0.822998	0.03346
[TG(51:9),TG(50:2)]_C18:0	0	0	1793.368134	2689.96018	2678.696202	0.905076662	0.819164	0.0346
[TG(54:7)]_C20:4	0	0	1077.956073	1704.236119	1647.636107	0.904693767	0.818471	0.03481
[TG(44:0),TG(O-45:0)]_C14:0	0	0	2376.696162	3522.37625	3472.872269	0.899993597	0.809988	0.03739
[TG(54:9),TG(53:2)]_C18:2	0	0	813.9400528	1527.936109	1350.132095	0.898809244	0.807858	0.03805
[TG(52:8),TG(51:1)]_C18:1	0	0	2826.548208	4437.620332	4212.224303	0.897977516	0.806364	0.03851
[TG(49:7)]_C18:1	0	0	1897.588137	2869.876217	2760.268214	0.895888657	0.802616	0.03969
[TG(61:10),TG(60:3)]_C18:1	0	0	797.2800583	1436.02811	1272.672094	0.895199649	0.801382	0.04008
[TG(53:10),TG(52:3)]_C18:3	0	0	2034.776152	3041.05222	2915.7122	0.892809911	0.79711	0.04144
CE(20:2)Na	0	0	4962.380328	7236.928491	7023.188535	0.892139677	0.795913	0.04183
[TG(40:0)]_C16:0	0	0	1292.304092	1846.192127	1796.604127	0.88904143	0.790395	0.04362
[TG(57:11),TG(56:4)]_C18:1	0	0	2037.33214	2956.820204	2842.088226	0.888164309	0.788836	0.04413
[TG(53:7),TG(52:0)]_C18:0	4863.868349	3691.084262	5794.072438	8013.256573	7662.456539	0.886424116	0.785748	0.04516

[TG(52:10),TG(51:3)]_C18:1	0	0	2086.65215	3082.372217	2912.972202	0.886040291	0.785067	0.04538
[TG(48:8),TG(47:1)]_C18:1	0	0	1319.668088	1784.468137	1781.424132	0.885733363	0.784524	0.04556
[TG(46:2)]_C16:0	0	0	2473.856171	3440.996264	3361.344242	0.88482551	0.782916	0.0461
[TG(52:6)]_C16:0	0	0	801.5120552	1421.048096	1224.956088	0.88424361	0.781887	0.04645
[TG(50:9),TG(49:2)]_C18:2	0	0	1934.148142	2705.980181	2622.492188	0.883476835	0.780531	0.0469
[TG(51:8),TG(50:1)]_C14:0	0	0	2543.336177	3499.644239	3427.900245	0.883409507	0.780412	0.04694
[TG(46:2)]_C14:0	0	0	1496.324101	1998.236145	1991.728133	0.882454426	0.778726	0.04752
PC(19:1),LPC(20:1),PC(O-20:1),F	0	0	1431.856106	1973.948135	1916.416137	0.880927168	0.776033	0.04843
[TG(50:5)]_C18:2	0	0	824.5880539	1608.552116	1321.832095	0.8790184	0.772673	0.04959
[TG(55:11),TG(54:4)]_C18:3	0	0	1975.928144	2672.232185	2592.864198	0.876506908	0.768264	0.05112
[TG(57:9),TG(56:2)]_C16:0	0	0	1339.188103	1227.552087	1703.536117	0.872715656	0.761633	0.05346
[TG(56:6)]_C18:2	0	0	1779.756127	2397.872166	2297.240155	0.871385619	0.759313	0.05429
[TG(58:8)]_C22:6	0	0	1059.384074	1501.408105	1389.216095	0.870728703	0.758168	0.0547
[TG(51:7),TG(50:0)]_C14:0	0	0	1113.252081	888.0840614	1475.024099	0.870450903	0.757685	0.05487
PC(42:2)	0	0	1016.344071	1774.400124	1450.644107	0.864007093	0.746508	0.05896
[TG(52:8),TG(51:1)]_C18:0	0	0	1392.036099	1741.176129	1693.152117	0.858034439	0.736223	0.06283
[TG(52:7),TG(51:0)]_C16:0	0	0	1550.996111	1875.82012	1820.324127	0.847995955	0.719097	0.0695
[TG(48:8),TG(47:1)]_C16:0	0	0	1537.928121	1984.852139	1810.200117	0.843411988	0.711344	0.07261
[TG(56:11),TG(55:4)]_C18:1	0	0	1372.1241	1643.324129	1578.968113	0.841584135	0.708264	0.07386
[TG(46:3)]_C18:2	0	0	1406.864096	1619.064114	1592.832111	0.838071673	0.702364	0.07629
LPI(20:0)	0	0	1927.260129	2137.328152	2087.300141	0.823386134	0.677965	0.0867
[TG(42:1)]_C18:1	0	0	746.1360542	1138.552084	882.1920631	0.821081939	0.674176	0.08837
[TG(54:11),TG(53:4)]_C18:1	0	0	1459.852097	1727.488125	1579.784108	0.819500895	0.671582	0.08952
[TG(44:0),TG(O-45:0)]_C18:0	0	0	835.6720566	1574.320109	1099.828076	0.816058495	0.665951	0.09204
FA(35:0)	2551.684185	2564.300188	2693.672191	2507.996179	0	-0.80776339	0.652482	0.09821
[TG(50:9),TG(49:2)]_C18:1	0	0	1773.268122	1977.816135	1832.116122	0.805113231	0.648207	0.1002
LPC(22:4)	0	0	1523.616113	2109.356151	1644.992116	0.801455795	0.642331	0.10298
[TG(48:7),TG(47:0)]_C14:0	0	0	817.8840599	901.3240608	822.2600578	0.794916417	0.631892	0.10799
SM(d18:2/14:0)	0	0	1900.16414	2334.436163	1922.812138	0.789953062	0.624026	0.11185
DG(36:6)_C16:1	0	0	1791.976125	1994.068134	1770.372132	0.7874842	0.620131	0.11378
[TG(56:6)]_C18:0	0	0	823.6840553	0	1355.996087	0.686953522	0.471905	0.20009
Cer(d14:2(4E,6E)/16:0)	2388.500163	3578.060268	2695.560181	2981.236212	1729.388106	-0.589615479	0.347646	0.29539
CAR(14:2)	3185.360237	4048.092293	3532.736249	4087.308277	4012.268279	0.588103753	0.345866	0.29694
DG(30:1)_C16:0	0	5373.152412	4395.836338	6201.920468	5066.976341	0.558072576	0.311445	0.32827
DG(36:7),DG(35:0)_C16:0	42226.943	53072.95999	41615.19514	75756.8015	56055.58364	0.557600778	0.310919	0.32877
DG(42:11),DG(41:4)_C16:0	4361.140311	4844.928361	3456.996242	6732.204516	5262.064371	0.523797107	0.274363	0.36497
DG(36:7)_C16:1	4942.860328	2849.112203	4440.996319	6212.084449	4834.248352	0.488566275	0.238697	0.40366
FA(28:6)	0	4254.296301	4695.940357	0	0	-0.470705673	0.221564	0.42361
DG(41:5)_C16:0	14579.77707	15879.84904	12811.14485	24669.7256	16857.25742	0.469828575	0.220739	0.4246
DG(41:6)_C16:1	3995.724309	0	3635.668261	4760.536362	3783.69228	0.453941907	0.206063	0.44254
DG(36:8),DG(35:1)_C16:1	28011.77401	14141.62903	21086.39354	33393.88667	25470.15775	0.421895169	0.177996	0.47922
DG(30:3)_C16:1	28508.31802	16390.63324	20943.36941	31905.93015	25544.16564	0.379524483	0.144039	0.52864
CE(15:1) NH4	5355.268349	3147.112246	5074.804372	5803.760481	4857.280334	0.334487275	0.111882	0.5822
DG(30:2)_C16:0	23059.68563	26623.92566	23132.82961	30443.06208	24705.7057	0.311111291	0.09679	0.61037
DG(36:6)_C16:0	24218.61766	25842.82584	20192.66964	36165.52255	24071.98983	0.272962533	0.074509	0.65682
CAR(10:2)	0	2294.824164	1671.156121	0	2373.884178	0.240905505	0.058035	0.69626
Cer(d18:0/17:0)	2195.984133	2378.212158	1707.772111	2899.352191	1579.92012	-0.227506833	0.051759	0.71285
FA(22:7)	23446.94558	0	20082.82943	0	13661.00103	-0.188619042	0.035577	0.76127
PG(16:0),LPG(17:0),LPG(O-18:0);	1709.00412	9939.004748	3357.332229	5766.516391	3305.524234	-0.176728867	0.031233	0.77616
PG(20:0),LPG(21:0); PG(20:0),LP(	0	3340.616238	0	2631.804181	0	-0.165584768	0.027418	0.79014
CAR(18:3)	0	2539.280182	0	2700.660184	0	-0.074261312	0.005515	0.90553
LPG(19:0),LPG(O-20:0); LPG(19:(	0	9515.784646	0	5830.248433	2760.62819	-0.007956126	6.33E-05	0.98987
PC(43:4),PC(O-44:4)	0	0	0	1583.528106	1582.060115	0.919451667	0.845391	0.02711
CE(18:1) NH4	0	0	0	163376.5043	163135.7643	0.919344811	0.845195	0.02716
PE(O-38:9),PE(36:2),PE(O-37:2),I	0	0	0	2138.668147	2134.720153	0.91927188	0.845061	0.0272
[TG(56:8)]_C18:2	0	0	0	1853.420129	1849.440132	0.919212813	0.844952	0.02723
[TG(44:2)]_C18:1	0	0	0	1134.332086	1124.476081	0.917917681	0.842573	0.02788
[TG(54:7)]_C18:1	0	0	0	1175.588083	1163.132076	0.917535443	0.841871	0.02807
[TG(53:9),TG(52:2)]_C20:0	0	0	0	790.6440521	775.8160538	0.915875518	0.838828	0.02892
CE(22:1) NH4	0	0	0	2650.900191	2601.076186	0.915867118	0.838813	0.02892
[TG(50:5)]_C20:4	0	0	0	1105.708076	1083.29208	0.91556215	0.838254	0.02908
[TG(55:10),TG(54:3)]_C20:0	0	0	0	793.2480545	775.9840584	0.915253198	0.837688	0.02924
PC(42:5)	0	0	0	1559.840121	1522.488093	0.91479847	0.836856	0.02947

[TG(52:6)]_C16:1	0	0	0	875.5920624	843.5880608	0.912115036	0.831954	0.03086
[TG(49:3)]_C18:2	0	0	0	1491.044104	1435.680103	0.911989255	0.831724	0.03093
FA(22:1)	3403.076238	4629.420335	3515.720241	0	0	-0.907703164	0.823925	0.03319
PG(32:0),PG(O-33:0); PG(32:0),P	0	0	0	1382.688093	1302.408099	0.907306271	0.823205	0.0334
[TG(58:8)]_C22:5	0	0	0	1099.980087	1028.416074	0.905677479	0.820252	0.03428
[TG(50:9),TG(49:2)]_C16:1	0	0	0	1849.984138	1674.912123	0.898433435	0.807183	0.03826
[TG(52:7),TG(51:0)]_C18:0	0	0	0	1760.116132	1589.876111	0.89789977	0.806224	0.03856
DG(39:8),DG(O-40:8)_C18:2	0	0	0	40661.52669	36697.24634	0.897701109	0.805867	0.03867
CE(18:3)Na	0	0	0	24823.19364	21960.18946	0.893002961	0.797454	0.04133
CE(20:4) NH4	0	0	0	174090.727	153563.3779	0.892305834	0.79621	0.04173
CE(18:1)K	0	0	0	5180.252359	4553.088293	0.891445042	0.794674	0.04223
CE(20:3) NH4	0	0	0	25591.77383	22210.60972	0.888373896	0.789208	0.04401
CE(19:0)H	0	0	0	239361.1813	207317.0219	0.88787801	0.788327	0.0443
[TG(52:4)]_C14:0	0	0	0	1394.116097	1180.492086	0.882252708	0.77837	0.04764
[TG(57:10),TG(56:3)]_C18:0	0	0	0	1145.560078	957.6160698	0.878980459	0.772607	0.04961
PI(38:3)	0	0	0	1913.876129	1598.540107	0.87876581	0.772229	0.04974
PC(42:7),PC(41:0),PC(O-42:0)	0	0	0	2087.588143	1732.264123	0.877083142	0.769275	0.05077
SM(d17:0/27:0)	0	0	0	1552.948112	1273.748085	0.874065843	0.763991	0.05262
PC(29:0),PC(O-30:0)	0	0	0	2030.144147	1656.940118	0.87276995	0.761727	0.05343
DG(O-38:8),DG(36:1)_C16:1	0	0	0	15571.91712	12566.55685	0.869783421	0.756523	0.05529
CE(18:0)K	0	0	0	38979.85059	31178.19025	0.86740738	0.752396	0.05679
[TG(46:1)]_C18:0	0	0	0	1002.048073	777.9560509	0.859299325	0.738395	0.062
[TG(58:7)]_C22:5	0	0	0	1099.940077	849.176062	0.857747994	0.735732	0.06301
[TG(58:9)]_C20:4	0	0	0	1107.34008	854.4680603	0.857611518	0.735498	0.0631
[TG(54:5)]_C16:1	0	0	0	1145.368089	870.5200577	0.853385215	0.728266	0.06589
CE(14:0) NH4	0	0	0	3877.108268	2931.144199	0.851893078	0.725722	0.06688
[TG(57:8),TG(56:1)]_C18:0	0	0	0	1092.388081	795.5400574	0.841206234	0.707628	0.07412
DG(O-40:9),DG(38:2)_C18:2	0	0	0	1098429.628	795787.6651	0.839697241	0.705091	0.07517
CE(16:3)Na	0	0	0	4617.032308	3291.360246	0.834983581	0.697198	0.07845
[TG(59:9),TG(58:2)]_C18:2	0	0	0	1428.864098	950.3120674	0.814247993	0.663	0.09338
CE(22:2) NH4	0	0	0	20919.08557	13836.30505	0.812569653	0.660269	0.09462
PC(24:0)	0	0	0	0	2252.064162	0.801783726	0.642857	0.10273
[TG(57:11),TG(56:4)]_C16:0	0	0	0	0	868.3720589	0.801783726	0.642857	0.10273
[TG(58:10)]_C20:4	0	0	0	0	817.8760536	0.801783726	0.642857	0.10273
[TG(56:8),TG(55:1)]_C18:1	0	0	0	0	1307.156096	0.801783726	0.642857	0.10273
PI(38:5)	0	0	0	0	984.9280647	0.801783726	0.642857	0.10273
CE(18:0) NH4	0	0	0	0	5104.008363	0.801783726	0.642857	0.10273
DG(36:3)_C18:2	0	0	0	0	4760.364308	0.801783726	0.642857	0.10273
DG(O-40:9),DG(38:2)_C18:1	0	0	0	0	2514.596175	0.801783726	0.642857	0.10273
DG(37:7)_C16:1	0	0	0	0	2042.416149	0.801783726	0.642857	0.10273
LPC(20:3)	0	0	0	0	1766.424122	0.801783726	0.642857	0.10273
[TG(60:15),TG(59:8),TG(58:1)]_C1	0	0	0	0	1575.440108	0.801783726	0.642857	0.10273
PC(19:0),LPC(20:0),PC(O-20:0)	0	0	0	0	1517.56411	0.801783726	0.642857	0.10273
PI(36:4)	0	0	0	0	1133.176081	0.801783726	0.642857	0.10273
SM(d18:2/15:0)	0	0	0	0	1098.092075	0.801783726	0.642857	0.10273
LPC(15:1),LPC(O-16:1),LPC(P-16	0	0	0	0	1038.528074	0.801783726	0.642857	0.10273
[TG(56:8)]_C18:3	0	0	0	0	805.8720566	0.801783726	0.642857	0.10273
[TG(54:12),TG(53:5)]_C18:2	0	0	0	0	796.2520569	0.801783726	0.642857	0.10273
CE(20:5)Na	0	0	0	0	4724.360338	0.801783726	0.642857	0.10273
CE(22:6)H	0	0	0	0	2120.912146	0.801783726	0.642857	0.10273
PC(36:6)	0	0	0	0	1671.432116	0.801783726	0.642857	0.10273
[TG(52:5)]_C22:5	0	0	0	0	874.7000618	0.801783726	0.642857	0.10273
[TG(45:0)]_C14:0	0	0	0	0	872.2800644	0.801783726	0.642857	0.10273
[TG(58:8)]_C20:4	0	0	0	0	814.0960594	0.801783726	0.642857	0.10273
[TG(48:4)]_C18:1	0	0	0	0	806.8280533	0.801783726	0.642857	0.10273
PC(14:0),LPC(15:0),LPC(O-16:0)	0	0	0	0	2477.912179	0.801783726	0.642857	0.10273
PI(36:1),PI(O-37:1),PI(P-37:0)	0	0	0	0	1126.468085	0.801783726	0.642857	0.10273
[TG(58:9),TG(57:2)]_C18:1	0	0	0	0	1034.240074	0.801783726	0.642857	0.10273
[TG(57:12),TG(56:5)]_C22:5	0	0	0	0	909.6320663	0.801783726	0.642857	0.10273
[TG(59:13),TG(58:6)]_C18:1	0	0	0	0	793.948056	0.801783726	0.642857	0.10273
CE(18:3) NH4	0	0	0	0	36562.02644	0.801783726	0.642857	0.10273
CE(20:1)Na	0	0	0	0	1974.888139	0.801783726	0.642857	0.10273
PE(36:3),PE(P-37:2)	0	0	0	0	1699.236117	0.801783726	0.642857	0.10273

[TG(54:6)]_C16:1	0	0	0	0	794.828054	0.801783726	0.642857	0.10273
[TG(50:7)]_C18:1	0	0	0	0	890.7520561	0.801783726	0.642857	0.10273
[TG(49:3)]_C16:0	0	0	0	0	781.9200554	0.801783726	0.642857	0.10273
[TG(58:9)]_C22:6	0	0	0	1379.800097	860.1440575	0.794366471	0.631018	0.10842
[TG(48:8),TG(47:1)]_C16:1	0	0	0	1610.648112	885.1000633	0.754930972	0.569921	0.14016
DG(30:2)_C16:1	4395.400277	0	0	4877.668311	3965.004272	0.442480809	0.195789	0.45558
CE(22:3) NH4	0	0	0	3805.036261	0	0.324531508	0.105321	0.59417
[TG(55:7),TG(54:0)]_C16:0	0	0	0	836.3640602	0	0.324531508	0.105321	0.59417
[TG(56:9),TG(55:2)]_C18:1	0	0	0	807.180056	0	0.324531508	0.105321	0.59417
DG(36:8),DG(35:1)_C18:1	0	0	0	2205.312156	0	0.324531508	0.105321	0.59417
CE(15:0) NH4	0	0	0	2176.480154	0	0.324531508	0.105321	0.59417
LPG(18:0); LPG(18:0)	0	0	0	937.5760712	0	0.324531508	0.105321	0.59417
[TG(58:14),TG(57:7),TG(56:0)]_C1	0	0	0	907.4440659	0	0.324531508	0.105321	0.59417
[TG(45:1)]_C16:0	0	0	0	833.3360596	0	0.324531508	0.105321	0.59417
[TG(52:9),TG(51:2)]_C18:0	0	0	0	1063.016071	0	0.324531508	0.105321	0.59417
[TG(53:10),TG(52:3)]_C20:0	0	0	0	1166.444081	0	0.324531508	0.105321	0.59417
PI(34:1),PI(O-35:1),PI(P-35:0)	0	0	0	1065.288077	0	0.324531508	0.105321	0.59417
FA(6:0)	0	0	0	11756.76485	0	0.324531508	0.105321	0.59417
DG(37:6)_C16:0	0	0	0	2406.896173	0	0.324531508	0.105321	0.59417
[TG(55:7)]_C18:1	0	0	0	974.9000717	0	0.324531508	0.105321	0.59417
PC(44:0)	0	0	0	1001.428074	0	0.324531508	0.105321	0.59417
PE(O-38:8),PE(36:1),PE(O-37:1),I	0	0	0	1056.268079	0	0.324531508	0.105321	0.59417
[TG(42:0)]_C18:0	0	0	0	760.8440483	0	0.324531508	0.105321	0.59417
[TG(48:4)]_C18:3	0	0	0	864.4000557	0	0.324531508	0.105321	0.59417
[TG(52:4)]_C18:0	0	0	0	834.6760597	0	0.324531508	0.105321	0.59417
[TG(46:3)]_C16:1	0	0	0	809.8800552	0	0.324531508	0.105321	0.59417
[TG(50:9),TG(49:2)]_C14:0	0	0	0	795.98806	0	0.324531508	0.105321	0.59417
FA(31:0)	0	0	0	3609.59225	0	0.324531508	0.105321	0.59417
[TG(38:1)]_C18:1	0	0	0	1339.884091	0	0.324531508	0.105321	0.59417
FA(24:4)	0	0	0	9376.488676	0	0.324531508	0.105321	0.59417
PE(38:4)	0	0	0	1435.340098	0	0.324531508	0.105321	0.59417
[TG(57:8)]_C18:2	0	0	0	943.8040672	0	0.324531508	0.105321	0.59417
FA(37:0)	0	0	2953.992216	0	0	-0.15272071	0.023324	0.80631
FA(30:0)	0	0	3574.488242	0	0	-0.15272071	0.023324	0.80631
[TG(50:8),TG(49:1)]_C16:1	0	0	857.9320534	0	0	-0.15272071	0.023324	0.80631
[TG(54:7),TG(53:0)]_C18:0	0	0	1138.920078	917.3560654	0	0.097357988	0.009479	0.87624
FA(21:0)	19297.19336	0	0	0	14768.55697	0.072116167	0.005201	0.90826
FA(18:3)	2666.712191	0	0	0	0	-0.534522484	0.285714	0.35339
FA(19:2)	3870.464274	0	0	0	0	-0.534522484	0.285714	0.35339
PS(25:0)	2320.640164	0	0	0	0	-0.534522484	0.285714	0.35339
FA(17:2)	3827.800286	0	0	0	0	-0.534522484	0.285714	0.35339
FA(22:0)	12961.88091	0	0	0	0	-0.534522484	0.285714	0.35339
FA(20:0)	24014.68572	0	0	0	0	-0.534522484	0.285714	0.35339
[TG(38:0)]_C14:0	0	0	0	0	0	#DIV/0!	#DIV/0!	#DIV/0!
PS(38:4)	0	0	0	0	0	#DIV/0!	#DIV/0!	#DIV/0!
PE(34:2),PE(O-35:2),PE(P-35:1)	0	0	0	0	0	#DIV/0!	#DIV/0!	#DIV/0!
PE(34:1),PE(O-35:1),PE(P-35:0)	0	0	0	0	0	#DIV/0!	#DIV/0!	#DIV/0!
PC(29:2),PC(P-30:1)	0	0	0	0	0	#DIV/0!	#DIV/0!	#DIV/0!
[TG(58:7)]_C18:1	0	0	0	0	0	#DIV/0!	#DIV/0!	#DIV/0!
PE(38:6)	0	0	0	0	0	#DIV/0!	#DIV/0!	#DIV/0!
PE(40:6),PE(dO-40:0)	0	0	0	0	0	#DIV/0!	#DIV/0!	#DIV/0!
PS(P-37:0)	0	0	0	0	0	#DIV/0!	#DIV/0!	#DIV/0!
PE(36:4),PE(O-37:4)	0	0	0	0	0	#DIV/0!	#DIV/0!	#DIV/0!
[TG(38:0)]_C18:0	0	0	0	0	0	#DIV/0!	#DIV/0!	#DIV/0!
PC(42:1)	0	0	0	0	0	#DIV/0!	#DIV/0!	#DIV/0!
[TG(58:8),TG(57:1)]_C18:1	0	0	0	0	0	#DIV/0!	#DIV/0!	#DIV/0!
DG(40:9),DG(39:2)_C18:2	0	0	0	0	0	#DIV/0!	#DIV/0!	#DIV/0!
[TG(55:11),TG(54:4)]_C16:1	0	0	0	0	0	#DIV/0!	#DIV/0!	#DIV/0!
PE(38:5)	0	0	0	0	0	#DIV/0!	#DIV/0!	#DIV/0!
Cer(d18:1/16:0)	0	0	0	0	0	#DIV/0!	#DIV/0!	#DIV/0!
FA(18:0)	0	0	0	0	0	#DIV/0!	#DIV/0!	#DIV/0!
LPE(22:4)	0	0	0	0	0	#DIV/0!	#DIV/0!	#DIV/0!
CE(20:3)Na	0	0	0	0	0	#DIV/0!	#DIV/0!	#DIV/0!

CE(20:1)H	0	0	0	0	0	#DIV/0!	#DIV/0!	#DIV/0!
DG(32:5)_C18:1	0	0	0	0	0	#DIV/0!	#DIV/0!	#DIV/0!
PC(30:3)	0	0	0	0	0	#DIV/0!	#DIV/0!	#DIV/0!
DG(34:1)_C16:1	0	0	0	0	0	#DIV/0!	#DIV/0!	#DIV/0!
LPI(19:0),LPI(O-20:0)	0	0	0	0	0	#DIV/0!	#DIV/0!	#DIV/0!
DG(41:6)_C18:1	0	0	0	0	0	#DIV/0!	#DIV/0!	#DIV/0!
PC(31:3),PC(O-32:3)	0	0	0	0	0	#DIV/0!	#DIV/0!	#DIV/0!
[TG(58:8)]_C18:2	0	0	0	0	0	#DIV/0!	#DIV/0!	#DIV/0!
[TG(45:3)]_C16:0	0	0	0	0	0	#DIV/0!	#DIV/0!	#DIV/0!
CE(16:0)H	0	0	0	0	0	#DIV/0!	#DIV/0!	#DIV/0!
FA(15:1)	0	0	0	0	0	#DIV/0!	#DIV/0!	#DIV/0!
FA(14:2)	0	0	0	0	0	#DIV/0!	#DIV/0!	#DIV/0!
FA(10:3)	0	0	0	0	0	#DIV/0!	#DIV/0!	#DIV/0!
DG(41:5)_C16:1	0	0	0	0	0	#DIV/0!	#DIV/0!	#DIV/0!
CE(22:4)H	0	0	0	0	0	#DIV/0!	#DIV/0!	#DIV/0!
CE(17:0)Na	0	0	0	0	0	#DIV/0!	#DIV/0!	#DIV/0!
PC(44:1)	0	0	0	0	0	#DIV/0!	#DIV/0!	#DIV/0!
PI(34:2),PI(O-35:2),PI(P-35:1)	0	0	0	0	0	#DIV/0!	#DIV/0!	#DIV/0!
[TG(52:10),TG(51:3)]_C16:1	0	0	0	0	0	#DIV/0!	#DIV/0!	#DIV/0!
CE(24:1) NH4	0	0	0	0	0	#DIV/0!	#DIV/0!	#DIV/0!
1-O-pentacosanoyl-Cer(d18:1/16	0	0	0	0	0	#DIV/0!	#DIV/0!	#DIV/0!
FA(16:0)	0	0	0	0	0	#DIV/0!	#DIV/0!	#DIV/0!
[TG(50:9),TG(49:2)]_C20:0	0	0	0	0	0	#DIV/0!	#DIV/0!	#DIV/0!
[TG(50:5)]_C18:3	0	0	0	0	0	#DIV/0!	#DIV/0!	#DIV/0!
CE(24:1)Na	0	0	0	0	0	#DIV/0!	#DIV/0!	#DIV/0!
CAR(20:1)	0	0	0	0	0	#DIV/0!	#DIV/0!	#DIV/0!
PG(25:0); PG(25:0)	0	0	0	0	0	#DIV/0!	#DIV/0!	#DIV/0!
PE(37:5),PE(O-38:5),PE(P-38:4)	0	0	0	0	0	#DIV/0!	#DIV/0!	#DIV/0!
PC(44:3)	0	0	0	0	0	#DIV/0!	#DIV/0!	#DIV/0!
PE(38:3)	0	0	0	0	0	#DIV/0!	#DIV/0!	#DIV/0!
DG(32:2)_C18:1	0	0	0	0	0	#DIV/0!	#DIV/0!	#DIV/0!
FA(34:6)	0	0	0	0	0	#DIV/0!	#DIV/0!	#DIV/0!
FA(19:0)	0	0	0	0	0	#DIV/0!	#DIV/0!	#DIV/0!
FA(26:6)	0	0	0	0	0	#DIV/0!	#DIV/0!	#DIV/0!
FA(23:0)	0	0	0	0	0	#DIV/0!	#DIV/0!	#DIV/0!
DG(34:2)_C16:1	0	0	0	0	0	#DIV/0!	#DIV/0!	#DIV/0!
CE(20:0)K	0	0	0	0	0	#DIV/0!	#DIV/0!	#DIV/0!
PC(44:2)	0	0	0	0	0	#DIV/0!	#DIV/0!	#DIV/0!
[TG(52:7)]_C18:1	0	0	0	0	0	#DIV/0!	#DIV/0!	#DIV/0!
LPC(14:0),PC(O-14:0),LPC(O-15:	0	0	0	0	0	#DIV/0!	#DIV/0!	#DIV/0!
[TG(45:2)]_C16:0	0	0	0	0	0	#DIV/0!	#DIV/0!	#DIV/0!
FA(21:1)	0	0	0	0	0	#DIV/0!	#DIV/0!	#DIV/0!
CAR(18:2)	0	0	0	0	0	#DIV/0!	#DIV/0!	#DIV/0!
[TG(45:1)]_C16:1	0	0	0	0	0	#DIV/0!	#DIV/0!	#DIV/0!
FA(26:2)	0	0	0	0	0	#DIV/0!	#DIV/0!	#DIV/0!
Cer(d18:0/21:0)	0	0	0	0	0	#DIV/0!	#DIV/0!	#DIV/0!
DG(41:6)_C16:0	0	0	0	0	0	#DIV/0!	#DIV/0!	#DIV/0!
LPS(P-16:0)	0	0	0	0	0	#DIV/0!	#DIV/0!	#DIV/0!
[TG(45:4)]_C20:0	0	0	0	0	0	#DIV/0!	#DIV/0!	#DIV/0!
DG(30:1)_C16:1	0	0	0	0	0	#DIV/0!	#DIV/0!	#DIV/0!
DG(41:5)_C18:0	0	0	0	0	0	#DIV/0!	#DIV/0!	#DIV/0!
DG(35:3)_C18:0	0	0	0	0	0	#DIV/0!	#DIV/0!	#DIV/0!
FA(34:0)	0	0	0	0	0	#DIV/0!	#DIV/0!	#DIV/0!
[TG(47:2)]_C16:1	0	0	0	0	0	#DIV/0!	#DIV/0!	#DIV/0!
CE(18:2)K	0	0	0	0	0	#DIV/0!	#DIV/0!	#DIV/0!
DG(40:2)_C18:2	0	0	0	0	0	#DIV/0!	#DIV/0!	#DIV/0!
CE(22:4) NH4	0	0	0	0	0	#DIV/0!	#DIV/0!	#DIV/0!
PC(27:0),PC(O-28:0)	0	0	0	0	0	#DIV/0!	#DIV/0!	#DIV/0!
[TG(51:7)]_C16:1	0	0	0	0	0	#DIV/0!	#DIV/0!	#DIV/0!
[TG(38:0)]_C20:0	0	0	0	0	0	#DIV/0!	#DIV/0!	#DIV/0!
[TG(48:6)]_C16:0	0	0	0	0	0	#DIV/0!	#DIV/0!	#DIV/0!
CE(14:1) NH4	0	0	0	0	0	#DIV/0!	#DIV/0!	#DIV/0!
[TG(39:0)]_C16:0	0	0	0	0	0	#DIV/0!	#DIV/0!	#DIV/0!

[TG(56:13),TG(55:6)]_C18:2	0	0	0	0	0	#DIV/0!	#DIV/0!	#DIV/0!
FA(38:6)	0	0	0	0	0	#DIV/0!	#DIV/0!	#DIV/0!
[TG(48:6)]_C18:0	0	0	0	0	0	#DIV/0!	#DIV/0!	#DIV/0!
PI(38:2),PI(P-39:1)	0	0	0	0	0	#DIV/0!	#DIV/0!	#DIV/0!
DG(36:8),DG(35:1)_C18:0	0	0	0	0	0	#DIV/0!	#DIV/0!	#DIV/0!
PC(44:7),PC(43:0)	0	0	0	0	0	#DIV/0!	#DIV/0!	#DIV/0!
[TG(37:0)]_C14:0	0	0	0	0	0	#DIV/0!	#DIV/0!	#DIV/0!
[TG(55:10),TG(54:3)]_C16:1	0	0	0	0	0	#DIV/0!	#DIV/0!	#DIV/0!
FA(10:5)	0	0	0	0	0	#DIV/0!	#DIV/0!	#DIV/0!
FA(28:0)	0	0	0	0	0	#DIV/0!	#DIV/0!	#DIV/0!
FA(32:0)	0	0	0	0	0	#DIV/0!	#DIV/0!	#DIV/0!
PC(44:4)	0	0	0	0	0	#DIV/0!	#DIV/0!	#DIV/0!
FA(18:2)	0	0	0	0	0	#DIV/0!	#DIV/0!	#DIV/0!
FA(21:2)	0	0	0	0	0	#DIV/0!	#DIV/0!	#DIV/0!
FA(30:2)	0	0	0	0	0	#DIV/0!	#DIV/0!	#DIV/0!
CAR(26:0)	0	0	0	0	0	#DIV/0!	#DIV/0!	#DIV/0!
DG(37:7)_C18:1	0	0	0	0	0	#DIV/0!	#DIV/0!	#DIV/0!
DG(30:2)_C18:1	0	0	0	0	0	#DIV/0!	#DIV/0!	#DIV/0!
FA(24:0)	0	0	0	0	0	#DIV/0!	#DIV/0!	#DIV/0!
FA(24:2)	0	0	0	0	0	#DIV/0!	#DIV/0!	#DIV/0!
FA(20:6)	0	0	0	0	0	#DIV/0!	#DIV/0!	#DIV/0!
FA(24:1)	0	0	0	0	0	#DIV/0!	#DIV/0!	#DIV/0!
PG(24:0); PG(24:0)	0	0	0	0	0	#DIV/0!	#DIV/0!	#DIV/0!
[TG(40:0)]_C18:0	0	0	0	0	0	#DIV/0!	#DIV/0!	#DIV/0!
DG(39:7),DG(38:0),DG(dO-40:0)_	0	0	0	0	0	#DIV/0!	#DIV/0!	#DIV/0!
CE(20:2) NH4	0	0	0	0	0	#DIV/0!	#DIV/0!	#DIV/0!
LPE(20:4)	0	0	0	0	0	#DIV/0!	#DIV/0!	#DIV/0!
LPC(22:6)	0	0	0	0	0	#DIV/0!	#DIV/0!	#DIV/0!
PC(26:1)	0	0	0	0	0	#DIV/0!	#DIV/0!	#DIV/0!
CE(24:1)K	0	0	0	0	0	#DIV/0!	#DIV/0!	#DIV/0!
[TG(44:5)]_C20:0	0	0	0	0	0	#DIV/0!	#DIV/0!	#DIV/0!
[TG(53:9),TG(52:2)]_C14:0	0	0	0	0	0	#DIV/0!	#DIV/0!	#DIV/0!
PC(44:8),PC(43:1)	0	0	0	0	0	#DIV/0!	#DIV/0!	#DIV/0!
[TG(38:1)]_C16:0	0	0	0	0	0	#DIV/0!	#DIV/0!	#DIV/0!
DG(40:7),DG(39:0)_C18:0	0	0	0	0	0	#DIV/0!	#DIV/0!	#DIV/0!
LPG(15:0),LPG(O-16:0); LPG(15:(	0	0	0	0	0	#DIV/0!	#DIV/0!	#DIV/0!
[TG(46:6)]_C16:0	0	0	0	0	0	#DIV/0!	#DIV/0!	#DIV/0!
FA(22:5)	0	0	0	0	0	#DIV/0!	#DIV/0!	#DIV/0!
[TG(51:8)]_C22:5	0	0	0	0	0	#DIV/0!	#DIV/0!	#DIV/0!
FA(32:5)	0	0	0	0	0	#DIV/0!	#DIV/0!	#DIV/0!
FA(30:4)	0	0	0	0	0	#DIV/0!	#DIV/0!	#DIV/0!
FA(10:2)	0	0	0	0	0	#DIV/0!	#DIV/0!	#DIV/0!
FA(23:1)	0	0	0	0	0	#DIV/0!	#DIV/0!	#DIV/0!
FA(20:2)	0	0	0	0	0	#DIV/0!	#DIV/0!	#DIV/0!
CAR(22:5)	0	0	0	0	0	#DIV/0!	#DIV/0!	#DIV/0!
[TG(62:14),TG(61:7),TG(60:0)]_C2	0	0	0	0	0	#DIV/0!	#DIV/0!	#DIV/0!
CAR(20:2)	0	0	0	0	0	#DIV/0!	#DIV/0!	#DIV/0!
[TG(37:0)]_C16:0	0	0	0	0	0	#DIV/0!	#DIV/0!	#DIV/0!
DG(32:1)_C18:1	0	0	0	0	0	#DIV/0!	#DIV/0!	#DIV/0!
DG(33:1),DG(O-34:1)_C16:1	0	0	0	0	0	#DIV/0!	#DIV/0!	#DIV/0!
DG(33:1),DG(O-34:1)_C18:1	0	0	0	0	0	#DIV/0!	#DIV/0!	#DIV/0!
DG(36:7)_C18:1	0	0	0	0	0	#DIV/0!	#DIV/0!	#DIV/0!
FA(19:1)	0	0	0	0	0	#DIV/0!	#DIV/0!	#DIV/0!
LPI(16:0)	0	0	0	0	0	#DIV/0!	#DIV/0!	#DIV/0!
LPE(18:1)	0	0	0	0	0	#DIV/0!	#DIV/0!	#DIV/0!
FA(25:0)	0	0	0	0	0	#DIV/0!	#DIV/0!	#DIV/0!
FA(6:2)	0	0	0	0	0	#DIV/0!	#DIV/0!	#DIV/0!
FA(33:0)	0	0	0	0	0	#DIV/0!	#DIV/0!	#DIV/0!
DG(30:1)_C18:1	0	0	0	0	0	#DIV/0!	#DIV/0!	#DIV/0!
CAR(18:0)	0	0	0	0	0	#DIV/0!	#DIV/0!	#DIV/0!
LPG(16:0); LPG(16:0)	0	0	0	0	0	#DIV/0!	#DIV/0!	#DIV/0!
FA(21:5)	0	0	0	0	0	#DIV/0!	#DIV/0!	#DIV/0!
FA(22:6)	0	0	0	0	0	#DIV/0!	#DIV/0!	#DIV/0!

FA(26:4)	0	0	0	0	0	#DIV/0!	#DIV/0!	#DIV/0!
FA(19:6)	0	0	0	0	0	#DIV/0!	#DIV/0!	#DIV/0!
DG(24:0)_C18:0	0	0	0	0	0	#DIV/0!	#DIV/0!	#DIV/0!
FA(20:1)	0	0	0	0	0	#DIV/0!	#DIV/0!	#DIV/0!
FA(18:1)	0	0	0	0	0	#DIV/0!	#DIV/0!	#DIV/0!
CAR(22:0)	0	0	0	0	0	#DIV/0!	#DIV/0!	#DIV/0!
CAR(5:1)	0	0	0	0	0	#DIV/0!	#DIV/0!	#DIV/0!
FA(22:2)	0	0	0	0	0	#DIV/0!	#DIV/0!	#DIV/0!
CAR(10:3)	0	0	0	0	0	#DIV/0!	#DIV/0!	#DIV/0!
FA(36:6)	0	0	0	0	0	#DIV/0!	#DIV/0!	#DIV/0!
[TG(43:1)]_C16:1	0	0	0	0	0	#DIV/0!	#DIV/0!	#DIV/0!
FA(16:6)	0	0	0	0	0	#DIV/0!	#DIV/0!	#DIV/0!
FA(27:3)	0	0	0	0	0	#DIV/0!	#DIV/0!	#DIV/0!
FA(36:4)	0	0	0	0	0	#DIV/0!	#DIV/0!	#DIV/0!
FA(19:5)	0	0	0	0	0	#DIV/0!	#DIV/0!	#DIV/0!
FA(24:5)	0	0	0	0	0	#DIV/0!	#DIV/0!	#DIV/0!
CAR(14:0)	0	0	0	0	0	#DIV/0!	#DIV/0!	#DIV/0!
[TG(45:2)]_C16:1	0	0	0	0	0	#DIV/0!	#DIV/0!	#DIV/0!
DG(33:3)_C16:0	0	0	0	0	0	#DIV/0!	#DIV/0!	#DIV/0!
FA(9:0)	0	0	0	0	0	#DIV/0!	#DIV/0!	#DIV/0!
FA(14:6)	0	0	0	0	0	#DIV/0!	#DIV/0!	#DIV/0!
FA(18:6)	0	0	0	0	0	#DIV/0!	#DIV/0!	#DIV/0!
FA(18:5)	0	0	0	0	0	#DIV/0!	#DIV/0!	#DIV/0!
FA(20:3)	0	0	0	0	0	#DIV/0!	#DIV/0!	#DIV/0!
DG(32:0)_C18:0	0	0	0	0	0	#DIV/0!	#DIV/0!	#DIV/0!
DG(32:0)_C16:0	0	0	0	0	0	#DIV/0!	#DIV/0!	#DIV/0!
DG(33:0)_C16:0	0	0	0	0	0	#DIV/0!	#DIV/0!	#DIV/0!
DG(34:4),DG(dO-36:4)_C16:1	0	0	0	0	0	#DIV/0!	#DIV/0!	#DIV/0!
DG(34:0)_C18:0	0	0	0	0	0	#DIV/0!	#DIV/0!	#DIV/0!
DG(34:0)_C16:0	0	0	0	0	0	#DIV/0!	#DIV/0!	#DIV/0!
DG(35:6)_C18:0	0	0	0	0	0	#DIV/0!	#DIV/0!	#DIV/0!
DG(35:6)_C16:0	0	0	0	0	0	#DIV/0!	#DIV/0!	#DIV/0!
DG(37:7),DG(36:0)_C18:0	0	0	0	0	0	#DIV/0!	#DIV/0!	#DIV/0!
DG(37:7),DG(36:0)_C16:0	0	0	0	0	0	#DIV/0!	#DIV/0!	#DIV/0!
CE(16:0) NH4	0	0	0	0	0	#DIV/0!	#DIV/0!	#DIV/0!
DG(37:6)_C18:0	0	0	0	0	0	#DIV/0!	#DIV/0!	#DIV/0!
CE(18:2)Na	0	0	0	0	0	#DIV/0!	#DIV/0!	#DIV/0!
CE(20:5)H	0	0	0	0	0	#DIV/0!	#DIV/0!	#DIV/0!
DG(34:3)_C18:1	0	0	0	0	0	#DIV/0!	#DIV/0!	#DIV/0!
DG(30:0)_C16:0	0	0	0	0	0	#DIV/0!	#DIV/0!	#DIV/0!
DG(40:5)_C16:0	0	0	0	0	0	#DIV/0!	#DIV/0!	#DIV/0!
CE(16:0)Na	0	0	0	0	0	#DIV/0!	#DIV/0!	#DIV/0!
DG(34:1)_C16:0	0	0	0	0	0	#DIV/0!	#DIV/0!	#DIV/0!
DG(34:2)_C18:2	0	0	0	0	0	#DIV/0!	#DIV/0!	#DIV/0!
DG(O-38:9),DG(36:2)_C18:1	0	0	0	0	0	#DIV/0!	#DIV/0!	#DIV/0!
CE(18:3)H	0	0	0	0	0	#DIV/0!	#DIV/0!	#DIV/0!
CE(15:1)K	0	0	0	0	0	#DIV/0!	#DIV/0!	#DIV/0!
CE(22:1)H	0	0	0	0	0	#DIV/0!	#DIV/0!	#DIV/0!
DG(38:5)_C16:0	0	0	0	0	0	#DIV/0!	#DIV/0!	#DIV/0!
DG(34:1)_C18:1	0	0	0	0	0	#DIV/0!	#DIV/0!	#DIV/0!
DG(36:3)_C18:1	0	0	0	0	0	#DIV/0!	#DIV/0!	#DIV/0!
DG(36:4),DG(O-37:4)_C18:2	0	0	0	0	0	#DIV/0!	#DIV/0!	#DIV/0!
CE(16:1)Na	0	0	0	0	0	#DIV/0!	#DIV/0!	#DIV/0!
CE(18:1)Na	0	0	0	0	0	#DIV/0!	#DIV/0!	#DIV/0!
DG(34:2)_C16:0	0	0	0	0	0	#DIV/0!	#DIV/0!	#DIV/0!
CE(20:4)H	0	0	0	0	0	#DIV/0!	#DIV/0!	#DIV/0!
DG(38:3)_C18:2	0	0	0	0	0	#DIV/0!	#DIV/0!	#DIV/0!
CE(19:0) NH4	0	0	0	0	0	#DIV/0!	#DIV/0!	#DIV/0!
CE(16:2)Na	0	0	0	0	0	#DIV/0!	#DIV/0!	#DIV/0!
DG(O-38:8),DG(36:1)_C18:1	0	0	0	0	0	#DIV/0!	#DIV/0!	#DIV/0!
CE(20:0)Na	0	0	0	0	0	#DIV/0!	#DIV/0!	#DIV/0!
DG(42:5)_C18:0	0	0	0	0	0	#DIV/0!	#DIV/0!	#DIV/0!
DG(O-38:8),DG(36:1)_C18:0	0	0	0	0	0	#DIV/0!	#DIV/0!	#DIV/0!

DG(O-38:9),DG(36:2)_C18:2	0	0	0	0	0	#DIV/0!	#DIV/0!	#DIV/0!
CE(20:4)Na	0	0	0	0	0	#DIV/0!	#DIV/0!	#DIV/0!
CE(22:2)H	0	0	0	0	0	#DIV/0!	#DIV/0!	#DIV/0!
DG(40:5)_C18:0	0	0	0	0	0	#DIV/0!	#DIV/0!	#DIV/0!
DG(36:7),DG(35:0)_C18:0	0	0	0	0	0	#DIV/0!	#DIV/0!	#DIV/0!
DG(34:2)_C18:1	0	0	0	0	0	#DIV/0!	#DIV/0!	#DIV/0!
CE(20:5)K	0	0	0	0	0	#DIV/0!	#DIV/0!	#DIV/0!
CE(17:1) NH4	0	0	0	0	0	#DIV/0!	#DIV/0!	#DIV/0!
DG(O-38:9),DG(36:2)_C18:0	0	0	0	0	0	#DIV/0!	#DIV/0!	#DIV/0!
DG(34:3)_C18:2	0	0	0	0	0	#DIV/0!	#DIV/0!	#DIV/0!
DG(32:1)_C16:0	0	0	0	0	0	#DIV/0!	#DIV/0!	#DIV/0!
CE(19:0)K	0	0	0	0	0	#DIV/0!	#DIV/0!	#DIV/0!
CE(18:3)K	0	0	0	0	0	#DIV/0!	#DIV/0!	#DIV/0!
CE(20:1)K	0	0	0	0	0	#DIV/0!	#DIV/0!	#DIV/0!
CE(22:4)K	0	0	0	0	0	#DIV/0!	#DIV/0!	#DIV/0!
DG(34:3)_C16:1	0	0	0	0	0	#DIV/0!	#DIV/0!	#DIV/0!
CE(15:0)K	0	0	0	0	0	#DIV/0!	#DIV/0!	#DIV/0!
SM(d18:2/20:1)	0	0	0	0	0	#DIV/0!	#DIV/0!	#DIV/0!
PE(32:1),PE(O-33:1),PE(P-33:0)	0	0	0	0	0	#DIV/0!	#DIV/0!	#DIV/0!
DG(38:7),DG(37:0)_C16:0	0	0	0	0	0	#DIV/0!	#DIV/0!	#DIV/0!
DG(32:1)_C16:1	0	0	0	0	0	#DIV/0!	#DIV/0!	#DIV/0!
	0	0	0	0	0	#DIV/0!	#DIV/0!	#DIV/0!
CE(24:1)H	0	0	0	0	0	#DIV/0!	#DIV/0!	#DIV/0!
CE(22:0)K	0	0	0	0	0	#DIV/0!	#DIV/0!	#DIV/0!
LPE(22:6)	0	0	0	0	0	#DIV/0!	#DIV/0!	#DIV/0!
PC(44:5)	0	0	0	0	0	#DIV/0!	#DIV/0!	#DIV/0!
CE(12:0) NH4	0	0	0	0	0	#DIV/0!	#DIV/0!	#DIV/0!
DG(40:6),DG(dO-40:0)_C16:0	0	0	0	0	0	#DIV/0!	#DIV/0!	#DIV/0!
CE(17:0) NH4	0	0	0	0	0	#DIV/0!	#DIV/0!	#DIV/0!
PE(37:6),PE(O-38:6),PE(P-38:5)	0	0	0	0	0	#DIV/0!	#DIV/0!	#DIV/0!
DG(44:9),DG(43:2)_C18:1	0	0	0	0	0	#DIV/0!	#DIV/0!	#DIV/0!
DG(44:7),DG(43:0)_C16:0	0	0	0	0	0	#DIV/0!	#DIV/0!	#DIV/0!
CE(18:2)H	0	0	0	0	0	#DIV/0!	#DIV/0!	#DIV/0!
DG(44:8),DG(43:1)_C18:1	0	0	0	0	0	#DIV/0!	#DIV/0!	#DIV/0!
CE(22:6)K	0	0	0	0	0	#DIV/0!	#DIV/0!	#DIV/0!
DG(34:3)_C16:0	0	0	0	0	0	#DIV/0!	#DIV/0!	#DIV/0!
PE(40:5)	0	0	0	0	0	#DIV/0!	#DIV/0!	#DIV/0!
DG(40:7),DG(39:0)_C16:0	0	0	0	0	0	#DIV/0!	#DIV/0!	#DIV/0!
PE(35:4),PE(O-36:4),PE(P-36:3)	0	0	0	0	0	#DIV/0!	#DIV/0!	#DIV/0!
DG(33:2)_C18:1	0	0	0	0	0	#DIV/0!	#DIV/0!	#DIV/0!
CAR(17:0)	0	0	0	0	0	#DIV/0!	#DIV/0!	#DIV/0!
[TG(44:3)]_C20:0	0	0	0	0	0	#DIV/0!	#DIV/0!	#DIV/0!
FA(14:5)	0	0	0	0	0	#DIV/0!	#DIV/0!	#DIV/0!
LPE(18:2),LPE(P-19:1)	0	0	0	0	0	#DIV/0!	#DIV/0!	#DIV/0!
[TG(40:1)]_C18:1	0	0	0	0	0	#DIV/0!	#DIV/0!	#DIV/0!
PC(43:2)	0	0	0	0	0	#DIV/0!	#DIV/0!	#DIV/0!
DG(28:2)_C18:1	0	0	0	0	0	#DIV/0!	#DIV/0!	#DIV/0!
DG(28:1)_C18:1	0	0	0	0	0	#DIV/0!	#DIV/0!	#DIV/0!
DG(31:1)_C18:1	0	0	0	0	0	#DIV/0!	#DIV/0!	#DIV/0!
DG(36:4),DG(O-37:4)_C18:1	0	0	0	0	0	#DIV/0!	#DIV/0!	#DIV/0!
CAR(22:2)	0	0	0	0	0	#DIV/0!	#DIV/0!	#DIV/0!
[TG(43:1)]_C18:1	0	0	0	0	0	#DIV/0!	#DIV/0!	#DIV/0!
[TG(38:1)]_C14:0	0	0	0	0	0	#DIV/0!	#DIV/0!	#DIV/0!
FA(40:6)	0	0	0	0	0	#DIV/0!	#DIV/0!	#DIV/0!
DG(42:6)_C16:0	0	0	0	0	0	#DIV/0!	#DIV/0!	#DIV/0!
FA(3:0)	0	0	0	0	0	#DIV/0!	#DIV/0!	#DIV/0!
[TG(43:1)]_C16:0	0	0	0	0	0	#DIV/0!	#DIV/0!	#DIV/0!
FA(27:0)	0	0	0	0	0	#DIV/0!	#DIV/0!	#DIV/0!
DG(44:7),DG(43:0)_C18:0	0	0	0	0	0	#DIV/0!	#DIV/0!	#DIV/0!
DG(42:7),DG(41:0)_C16:0	0	0	0	0	0	#DIV/0!	#DIV/0!	#DIV/0!
FA(28:1)	0	0	0	0	0	#DIV/0!	#DIV/0!	#DIV/0!
FA(26:0)	0	0	0	0	0	#DIV/0!	#DIV/0!	#DIV/0!
DG(38:6),DG(dO-40:6)_C16:0	0	0	0	0	0	#DIV/0!	#DIV/0!	#DIV/0!

FA(34:1)	0	0	0	0	0	#DIV/0!	#DIV/0!	#DIV/0!
[TG(55:7),TG(54:0)]_C14:0	0	0	0	0	0	#DIV/0!	#DIV/0!	#DIV/0!
PI(36:3),PI(P-37:2)	0	0	0	0	0	#DIV/0!	#DIV/0!	#DIV/0!
[TG(47:6)]_C14:0	0	0	0	0	0	#DIV/0!	#DIV/0!	#DIV/0!
[TG(47:2)]_C16:0	0	0	0	0	0	#DIV/0!	#DIV/0!	#DIV/0!
[TG(59:9),TG(58:2)]_C16:0	0	0	0	0	0	#DIV/0!	#DIV/0!	#DIV/0!
[TG(43:0)]_C16:0	0	0	0	0	0	#DIV/0!	#DIV/0!	#DIV/0!
[TG(46:3)]_C14:0	0	0	0	0	0	#DIV/0!	#DIV/0!	#DIV/0!
[TG(51:8),TG(50:1)]_C20:0	0	0	0	0	0	#DIV/0!	#DIV/0!	#DIV/0!
[TG(42:2)]_C18:2	0	0	0	0	0	#DIV/0!	#DIV/0!	#DIV/0!
[TG(42:1)]_C14:0	0	0	0	0	0	#DIV/0!	#DIV/0!	#DIV/0!
[TG(44:2)]_C16:1	0	0	0	0	0	#DIV/0!	#DIV/0!	#DIV/0!
[TG(44:2)]_C14:0	0	0	0	0	0	#DIV/0!	#DIV/0!	#DIV/0!
FA(36:5)	0	0	0	0	0	#DIV/0!	#DIV/0!	#DIV/0!
PG(O-37:2),PG(P-37:1); PG(O-37:	0	0	0	0	0	#DIV/0!	#DIV/0!	#DIV/0!
[TG(47:2)]_C14:0	0	0	0	0	0	#DIV/0!	#DIV/0!	#DIV/0!
[TG(49:3)]_C18:1	0	0	0	0	0	#DIV/0!	#DIV/0!	#DIV/0!
[TG(45:1)]_C18:1	0	0	0	0	0	#DIV/0!	#DIV/0!	#DIV/0!
[TG(47:2)]_C18:1	0	0	0	0	0	#DIV/0!	#DIV/0!	#DIV/0!
[TG(52:6)]_C14:0	0	0	0	0	0	#DIV/0!	#DIV/0!	#DIV/0!
[TG(54:7)]_C16:1	0	0	0	0	0	#DIV/0!	#DIV/0!	#DIV/0!
DG(32:2)_C18:2	0	0	0	0	0	#DIV/0!	#DIV/0!	#DIV/0!
LPC(17:1),LPC(O-18:1),LPC(P-18	0	0	0	0	0	#DIV/0!	#DIV/0!	#DIV/0!
[TG(60:15),TG(59:8),TG(58:1)]_C1	0	0	0	0	0	#DIV/0!	#DIV/0!	#DIV/0!
CAR(16:0)	0	0	0	0	0	#DIV/0!	#DIV/0!	#DIV/0!
PG(36:3),PG(P-37:2); PG(36:3),P(	0	0	0	0	0	#DIV/0!	#DIV/0!	#DIV/0!
PC(50:0)	0	0	0	0	0	#DIV/0!	#DIV/0!	#DIV/0!
[TG(40:0)]_C14:0	0	0	0	0	0	#DIV/0!	#DIV/0!	#DIV/0!
[TG(47:6)]_C18:0	0	0	0	0	0	#DIV/0!	#DIV/0!	#DIV/0!
DG(44:1)_C18:1	0	0	0	0	0	#DIV/0!	#DIV/0!	#DIV/0!
[TG(49:3)]_C16:1	0	0	0	0	0	#DIV/0!	#DIV/0!	#DIV/0!
DG(38:8),DG(dO-40:8),DG(37:1)_	0	0	0	0	0	#DIV/0!	#DIV/0!	#DIV/0!
[TG(38:0)]_C16:0	0	0	0	0	0	#DIV/0!	#DIV/0!	#DIV/0!
[TG(45:4)]_C18:1	0	0	0	0	0	#DIV/0!	#DIV/0!	#DIV/0!
[TG(50:9)]_C22:6	0	0	0	0	0	#DIV/0!	#DIV/0!	#DIV/0!
[TG(40:1)]_C16:0	0	0	0	0	0	#DIV/0!	#DIV/0!	#DIV/0!
[TG(60:14),TG(59:7),TG(58:0)]_C1	0	0	0	0	0	#DIV/0!	#DIV/0!	#DIV/0!
PG(30:0),PG(O-31:0); PG(30:0),P(	0	0	0	0	0	#DIV/0!	#DIV/0!	#DIV/0!
[TG(54:12),TG(53:5)]_C22:5	0	0	0	0	0	#DIV/0!	#DIV/0!	#DIV/0!
DG(41:7),DG(40:0)_C16:0	0	0	0	0	0	#DIV/0!	#DIV/0!	#DIV/0!
[TG(44:4)]_C20:0	0	0	0	0	0	#DIV/0!	#DIV/0!	#DIV/0!
FA(28:3)	0	0	0	0	0	#DIV/0!	#DIV/0!	#DIV/0!
[TG(49:3)]_C20:0	0	0	0	0	0	#DIV/0!	#DIV/0!	#DIV/0!
[TG(45:4)]_C16:1	0	0	0	0	0	#DIV/0!	#DIV/0!	#DIV/0!
FA(25:4)	0	0	0	0	0	#DIV/0!	#DIV/0!	#DIV/0!
FA(30:3)	0	0	0	0	0	#DIV/0!	#DIV/0!	#DIV/0!
CE(50:3;O2)H	0	0	0	0	0	#DIV/0!	#DIV/0!	#DIV/0!
PG(26:0); PG(26:0)	0	0	0	0	0	#DIV/0!	#DIV/0!	#DIV/0!
PC(44:6)	0	0	0	0	0	#DIV/0!	#DIV/0!	#DIV/0!
[TG(40:1)]_C14:0	0	0	0	0	0	#DIV/0!	#DIV/0!	#DIV/0!
[TG(42:1)]_C16:1	0	0	0	0	0	#DIV/0!	#DIV/0!	#DIV/0!
[TG(45:1)]_C14:0	0	0	0	0	0	#DIV/0!	#DIV/0!	#DIV/0!
[TG(55:7)]_C22:6	0	0	0	0	0	#DIV/0!	#DIV/0!	#DIV/0!
CE(46:3;O2) NH4	0	0	0	0	0	#DIV/0!	#DIV/0!	#DIV/0!
FA(12:6)	0	0	0	0	0	#DIV/0!	#DIV/0!	#DIV/0!
FA(17:1)	0	0	0	0	0	#DIV/0!	#DIV/0!	#DIV/0!
FA(24:3)	0	0	0	0	0	#DIV/0!	#DIV/0!	#DIV/0!
FA(26:3)	0	0	0	0	0	#DIV/0!	#DIV/0!	#DIV/0!
FA(38:4)	0	0	0	0	0	#DIV/0!	#DIV/0!	#DIV/0!
FA(5:1)	0	0	0	0	0	#DIV/0!	#DIV/0!	#DIV/0!
DG(29:2)_C16:0	0	0	0	0	0	#DIV/0!	#DIV/0!	#DIV/0!
PG(28:0),PG(O-29:0); PG(28:0),P(	0	0	0	0	0	#DIV/0!	#DIV/0!	#DIV/0!
FA(15:5)	0	0	0	0	0	#DIV/0!	#DIV/0!	#DIV/0!

FA(17:6)	0	0	0	0	0	#DIV/0!	#DIV/0!	#DIV/0!
FA(26:5)	0	0	0	0	0	#DIV/0!	#DIV/0!	#DIV/0!
[TG(57:8),TG(56:1)]_C16:1	0	0	0	0	0	#DIV/0!	#DIV/0!	#DIV/0!
LPS(O-20:0)	0	0	0	0	0	#DIV/0!	#DIV/0!	#DIV/0!
LPE(12:0)	0	0	0	0	0	#DIV/0!	#DIV/0!	#DIV/0!
[TG(48:5)]_C22:5	0	0	0	0	0	#DIV/0!	#DIV/0!	#DIV/0!
[TG(50:5)]_C22:5	0	0	0	0	0	#DIV/0!	#DIV/0!	#DIV/0!
[TG(59:12),TG(58:5)]_C22:5	0	0	0	0	0	#DIV/0!	#DIV/0!	#DIV/0!
DG(34:5)_C16:1	0	0	0	0	0	#DIV/0!	#DIV/0!	#DIV/0!
DG(42:0)_C16:0	0	0	0	0	0	#DIV/0!	#DIV/0!	#DIV/0!
FA(27:2)	0	0	0	0	0	#DIV/0!	#DIV/0!	#DIV/0!
DG(44:8),DG(43:1)_C16:0	0	0	0	0	0	#DIV/0!	#DIV/0!	#DIV/0!
CE(16:2) NH4	0	0	0	0	0	#DIV/0!	#DIV/0!	#DIV/0!
CE(16:1)K	0	0	0	0	0	#DIV/0!	#DIV/0!	#DIV/0!
LPC(22:5)	0	0	0	0	0	#DIV/0!	#DIV/0!	#DIV/0!
DG(40:8),DG(39:1)_C18:1	0	0	0	0	0	#DIV/0!	#DIV/0!	#DIV/0!
CE(18:0)H	0	0	0	0	0	#DIV/0!	#DIV/0!	#DIV/0!
CE(20:4)K	0	0	0	0	0	#DIV/0!	#DIV/0!	#DIV/0!
DG(31:1)_C16:0	0	0	0	0	0	#DIV/0!	#DIV/0!	#DIV/0!
CE(22:5)Na	0	0	0	0	0	#DIV/0!	#DIV/0!	#DIV/0!
DG(44:9),DG(43:2)_C18:2	0	0	0	0	0	#DIV/0!	#DIV/0!	#DIV/0!
DG(42:8),DG(41:1)_C18:1	0	0	0	0	0	#DIV/0!	#DIV/0!	#DIV/0!
[TG(37:0)]_C20:0	0	0	0	0	0	#DIV/0!	#DIV/0!	#DIV/0!
PE(40:4)	0	0	0	0	0	#DIV/0!	#DIV/0!	#DIV/0!
DG(30:2)_C18:2	0	0	0	0	0	#DIV/0!	#DIV/0!	#DIV/0!
CE(12:0)Na	0	0	0	0	0	#DIV/0!	#DIV/0!	#DIV/0!
CE(22:3)Na	0	0	0	0	0	#DIV/0!	#DIV/0!	#DIV/0!
DG(33:3)_C18:2	0	0	0	0	0	#DIV/0!	#DIV/0!	#DIV/0!
DG(44:0)_C16:0	0	0	0	0	0	#DIV/0!	#DIV/0!	#DIV/0!
FA(14:1)	0	0	0	0	0	#DIV/0!	#DIV/0!	#DIV/0!
DG(38:7),DG(37:0)_C18:0	0	0	0	0	0	#DIV/0!	#DIV/0!	#DIV/0!
PC(18:0),LPC(19:0),PC(O-19:0),L	0	0	0	0	0	#DIV/0!	#DIV/0!	#DIV/0!
PE(39:6),PE(O-40:6),PE(P-40:5)	0	0	0	0	0	#DIV/0!	#DIV/0!	#DIV/0!
[TG(45:0)]_C18:0	0	0	0	0	0	#DIV/0!	#DIV/0!	#DIV/0!
[TG(51:4)]_C16:1	0	0	0	0	0	#DIV/0!	#DIV/0!	#DIV/0!
[TG(52:9),TG(51:2)]_C14:0	0	0	0	0	0	#DIV/0!	#DIV/0!	#DIV/0!
DG(26:0)_C16:0	0	0	0	0	0	#DIV/0!	#DIV/0!	#DIV/0!
CE(16:3) NH4	0	0	0	0	0	#DIV/0!	#DIV/0!	#DIV/0!
CE(22:5)K	0	0	0	0	0	#DIV/0!	#DIV/0!	#DIV/0!
SM(d16:0/14:0)	0	0	0	0	0	#DIV/0!	#DIV/0!	#DIV/0!
CE(20:3)K	0	0	0	0	0	#DIV/0!	#DIV/0!	#DIV/0!
CE(22:0) NH4	0	0	0	0	0	#DIV/0!	#DIV/0!	#DIV/0!
FA(26:1)	0	0	0	0	0	#DIV/0!	#DIV/0!	#DIV/0!
DG(33:1),DG(O-34:1)_C16:0	0	0	0	0	0	#DIV/0!	#DIV/0!	#DIV/0!
DG(39:7),DG(38:0),DG(dO-40:0)_	0	0	0	0	0	#DIV/0!	#DIV/0!	#DIV/0!
[TG(44:4)]_C18:1	0	0	0	0	0	#DIV/0!	#DIV/0!	#DIV/0!
[TG(45:3)]_C20:0	0	0	0	0	0	#DIV/0!	#DIV/0!	#DIV/0!
[TG(51:9)]_C22:6	0	0	0	0	0	#DIV/0!	#DIV/0!	#DIV/0!
DG(32:2)_C18:0	0	0	0	0	0	#DIV/0!	#DIV/0!	#DIV/0!
FA(29:2)	0	0	0	0	0	#DIV/0!	#DIV/0!	#DIV/0!
[TG(39:0)]_C18:0	0	0	0	0	0	#DIV/0!	#DIV/0!	#DIV/0!
[TG(44:3)]_C16:1	0	0	0	0	0	#DIV/0!	#DIV/0!	#DIV/0!
DG(36:8),DG(35:1)_C16:0	0	0	0	0	0	#DIV/0!	#DIV/0!	#DIV/0!
DG(38:9),DG(dO-40:9),DG(37:2)_	0	0	0	0	0	#DIV/0!	#DIV/0!	#DIV/0!
FA(16:1)	0	0	0	0	0	#DIV/0!	#DIV/0!	#DIV/0!
CAR(10:1)	0	0	0	0	0	#DIV/0!	#DIV/0!	#DIV/0!
CAR(20:4)	0	0	0	0	0	#DIV/0!	#DIV/0!	#DIV/0!
[TG(44:5)]_C18:2	0	0	0	0	0	#DIV/0!	#DIV/0!	#DIV/0!
FA(11:0)	0	0	0	0	0	#DIV/0!	#DIV/0!	#DIV/0!
FA(29:0)	0	0	0	0	0	#DIV/0!	#DIV/0!	#DIV/0!
CAR	0	0	0	0	0	#DIV/0!	#DIV/0!	#DIV/0!
CAR(5:0)	0	0	0	0	0	#DIV/0!	#DIV/0!	#DIV/0!
CAR(7:0)	0	0	0	0	0	#DIV/0!	#DIV/0!	#DIV/0!

CAR(8:1)	0	0	0	0	0	#DIV/0!	#DIV/0!	#DIV/0!
CAR(8:0)	0	0	0	0	0	#DIV/0!	#DIV/0!	#DIV/0!
CAR(9:0)	0	0	0	0	0	#DIV/0!	#DIV/0!	#DIV/0!
CAR(10:0)	0	0	0	0	0	#DIV/0!	#DIV/0!	#DIV/0!
CAR(11:0)	0	0	0	0	0	#DIV/0!	#DIV/0!	#DIV/0!
CAR(12:0)	0	0	0	0	0	#DIV/0!	#DIV/0!	#DIV/0!
CAR(16:2)	0	0	0	0	0	#DIV/0!	#DIV/0!	#DIV/0!
CAR(16:1)	0	0	0	0	0	#DIV/0!	#DIV/0!	#DIV/0!
CAR(18:4)	0	0	0	0	0	#DIV/0!	#DIV/0!	#DIV/0!
CAR(18:1)	0	0	0	0	0	#DIV/0!	#DIV/0!	#DIV/0!
LPC(12:0)	0	0	0	0	0	#DIV/0!	#DIV/0!	#DIV/0!
LPG(12:0); LPG(12:0)	0	0	0	0	0	#DIV/0!	#DIV/0!	#DIV/0!
CAR(22:6)	0	0	0	0	0	#DIV/0!	#DIV/0!	#DIV/0!
LPG(14:0); LPG(14:0)	0	0	0	0	0	#DIV/0!	#DIV/0!	#DIV/0!
LPE(18:3)	0	0	0	0	0	#DIV/0!	#DIV/0!	#DIV/0!
CAR(22:4)	0	0	0	0	0	#DIV/0!	#DIV/0!	#DIV/0!
LPC(16:1),LPC(P-17:0)	0	0	0	0	0	#DIV/0!	#DIV/0!	#DIV/0!
LPE(20:5)	0	0	0	0	0	#DIV/0!	#DIV/0!	#DIV/0!
LPE(20:3)	0	0	0	0	0	#DIV/0!	#DIV/0!	#DIV/0!
LPC(18:3)	0	0	0	0	0	#DIV/0!	#DIV/0!	#DIV/0!
LPE(22:5)	0	0	0	0	0	#DIV/0!	#DIV/0!	#DIV/0!
LPG(18:1); LPG(18:1)	0	0	0	0	0	#DIV/0!	#DIV/0!	#DIV/0!
PC(18:1),LPC(19:1),PC(O-19:1),L	0	0	0	0	0	#DIV/0!	#DIV/0!	#DIV/0!
LPC(20:5)	0	0	0	0	0	#DIV/0!	#DIV/0!	#DIV/0!
LPS(22:6)	0	0	0	0	0	#DIV/0!	#DIV/0!	#DIV/0!
LPG(22:6); LPG(22:6)	0	0	0	0	0	#DIV/0!	#DIV/0!	#DIV/0!
LPG(22:0); LPG(22:0)	0	0	0	0	0	#DIV/0!	#DIV/0!	#DIV/0!
LPI(19:1),LPI(P-20:0)	0	0	0	0	0	#DIV/0!	#DIV/0!	#DIV/0!
PG(26:1); PG(26:1)	0	0	0	0	0	#DIV/0!	#DIV/0!	#DIV/0!
SM(d18:0/13:0)	0	0	0	0	0	#DIV/0!	#DIV/0!	#DIV/0!
Cer(d18:1/25:0)	0	0	0	0	0	#DIV/0!	#DIV/0!	#DIV/0!
PG(28:1),PG(P-29:0); PG(28:1),P	0	0	0	0	0	#DIV/0!	#DIV/0!	#DIV/0!
PG(29:0),PG(O-30:0); PG(29:0),P	0	0	0	0	0	#DIV/0!	#DIV/0!	#DIV/0!
PG(30:1),PG(O-31:1),PG(P-31:0);	0	0	0	0	0	#DIV/0!	#DIV/0!	#DIV/0!
PE(34:4)	0	0	0	0	0	#DIV/0!	#DIV/0!	#DIV/0!
PG(31:0),PG(O-32:0); PG(31:0),P	0	0	0	0	0	#DIV/0!	#DIV/0!	#DIV/0!
PG(32:2),PG(O-33:2),PG(P-33:1);	0	0	0	0	0	#DIV/0!	#DIV/0!	#DIV/0!
PC(33:5),PC(P-34:4)	0	0	0	0	0	#DIV/0!	#DIV/0!	#DIV/0!
PG(32:1),PG(O-33:1),PG(P-33:0);	0	0	0	0	0	#DIV/0!	#DIV/0!	#DIV/0!
PC(33:4),PC(O-34:4),PC(O-34:4),	0	0	0	0	0	#DIV/0!	#DIV/0!	#DIV/0!
PC(34:5)	0	0	0	0	0	#DIV/0!	#DIV/0!	#DIV/0!
PG(33:1),PG(O-34:1),PG(P-34:0);	0	0	0	0	0	#DIV/0!	#DIV/0!	#DIV/0!
PC(34:4),PC(O-35:4)	0	0	0	0	0	#DIV/0!	#DIV/0!	#DIV/0!
PE(37:4),PE(O-38:4),PE(P-38:3)	0	0	0	0	0	#DIV/0!	#DIV/0!	#DIV/0!
PG(33:0),PG(O-34:0); PG(33:0),P	0	0	0	0	0	#DIV/0!	#DIV/0!	#DIV/0!
PG(34:0),PG(O-35:0); PG(34:0),P	0	0	0	0	0	#DIV/0!	#DIV/0!	#DIV/0!
PG(35:6),PG(P-36:5); PG(35:6),P	0	0	0	0	0	#DIV/0!	#DIV/0!	#DIV/0!
PG(36:1),PG(O-37:1),PG(P-37:0);	0	0	0	0	0	#DIV/0!	#DIV/0!	#DIV/0!
PG(37:7),PG(P-38:6),PG(36:0),PG	0	0	0	0	0	#DIV/0!	#DIV/0!	#DIV/0!
PE(40:3)	0	0	0	0	0	#DIV/0!	#DIV/0!	#DIV/0!
PE(42:5)	0	0	0	0	0	#DIV/0!	#DIV/0!	#DIV/0!
PS(40:4)	0	0	0	0	0	#DIV/0!	#DIV/0!	#DIV/0!
PE(44:8),PE(43:1)	0	0	0	0	0	#DIV/0!	#DIV/0!	#DIV/0!
PE(44:7),PE(43:0)	0	0	0	0	0	#DIV/0!	#DIV/0!	#DIV/0!
PS(O-42:0)	0	0	0	0	0	#DIV/0!	#DIV/0!	#DIV/0!
PS(42:5)	0	0	0	0	0	#DIV/0!	#DIV/0!	#DIV/0!
PS(43:1)	0	0	0	0	0	#DIV/0!	#DIV/0!	#DIV/0!
PS(43:0)	0	0	0	0	0	#DIV/0!	#DIV/0!	#DIV/0!
PS(44:6)	0	0	0	0	0	#DIV/0!	#DIV/0!	#DIV/0!
PI(40:6)	0	0	0	0	0	#DIV/0!	#DIV/0!	#DIV/0!
PI(40:5)	0	0	0	0	0	#DIV/0!	#DIV/0!	#DIV/0!
[TG(63:13),TG(62:6)]_C22:6	0	0	0	0	0	#DIV/0!	#DIV/0!	#DIV/0!
[TG(38:1)]_C16:1	0	0	0	0	0	#DIV/0!	#DIV/0!	#DIV/0!

[TG(39:1)]_C18:1	0	0	0	0	0	#DIV/0!	#DIV/0!	#DIV/0!
[TG(39:1)]_C16:0	0	0	0	0	0	#DIV/0!	#DIV/0!	#DIV/0!
[TG(39:1)]_C16:1	0	0	0	0	0	#DIV/0!	#DIV/0!	#DIV/0!
[TG(39:1)]_C14:0	0	0	0	0	0	#DIV/0!	#DIV/0!	#DIV/0!
[TG(39:0)]_C14:0	0	0	0	0	0	#DIV/0!	#DIV/0!	#DIV/0!
[TG(40:2)]_C18:1	0	0	0	0	0	#DIV/0!	#DIV/0!	#DIV/0!
[TG(40:2)]_C16:1	0	0	0	0	0	#DIV/0!	#DIV/0!	#DIV/0!
[TG(40:1)]_C18:0	0	0	0	0	0	#DIV/0!	#DIV/0!	#DIV/0!
[TG(40:1)]_C16:1	0	0	0	0	0	#DIV/0!	#DIV/0!	#DIV/0!
[TG(41:2)]_C18:1	0	0	0	0	0	#DIV/0!	#DIV/0!	#DIV/0!
[TG(41:1)]_C18:1	0	0	0	0	0	#DIV/0!	#DIV/0!	#DIV/0!
[TG(41:1)]_C16:0	0	0	0	0	0	#DIV/0!	#DIV/0!	#DIV/0!
[TG(41:1)]_C16:1	0	0	0	0	0	#DIV/0!	#DIV/0!	#DIV/0!
[TG(41:1)]_C14:0	0	0	0	0	0	#DIV/0!	#DIV/0!	#DIV/0!
[TG(41:0)]_C18:0	0	0	0	0	0	#DIV/0!	#DIV/0!	#DIV/0!
[TG(41:0)]_C14:0	0	0	0	0	0	#DIV/0!	#DIV/0!	#DIV/0!
[TG(42:2)]_C16:1	0	0	0	0	0	#DIV/0!	#DIV/0!	#DIV/0!
[TG(42:2)]_C14:0	0	0	0	0	0	#DIV/0!	#DIV/0!	#DIV/0!
[TG(43:2)]_C16:1	0	0	0	0	0	#DIV/0!	#DIV/0!	#DIV/0!
[TG(43:1)]_C14:0	0	0	0	0	0	#DIV/0!	#DIV/0!	#DIV/0!
[TG(43:0)]_C18:0	0	0	0	0	0	#DIV/0!	#DIV/0!	#DIV/0!
[TG(43:0)]_C14:0	0	0	0	0	0	#DIV/0!	#DIV/0!	#DIV/0!
[TG(44:5)]_C22:5	0	0	0	0	0	#DIV/0!	#DIV/0!	#DIV/0!
[TG(44:4)]_C16:1	0	0	0	0	0	#DIV/0!	#DIV/0!	#DIV/0!
[TG(45:3)]_C16:1	0	0	0	0	0	#DIV/0!	#DIV/0!	#DIV/0!
[TG(45:2)]_C18:1	0	0	0	0	0	#DIV/0!	#DIV/0!	#DIV/0!
[TG(45:2)]_C18:2	0	0	0	0	0	#DIV/0!	#DIV/0!	#DIV/0!
[TG(45:2)]_C14:0	0	0	0	0	0	#DIV/0!	#DIV/0!	#DIV/0!
[TG(45:1)]_C18:0	0	0	0	0	0	#DIV/0!	#DIV/0!	#DIV/0!
[TG(46:6)]_C18:0	0	0	0	0	0	#DIV/0!	#DIV/0!	#DIV/0!
[TG(46:6)]_C14:0	0	0	0	0	0	#DIV/0!	#DIV/0!	#DIV/0!
[TG(47:3)]_C18:2	0	0	0	0	0	#DIV/0!	#DIV/0!	#DIV/0!
[TG(47:3)]_C16:1	0	0	0	0	0	#DIV/0!	#DIV/0!	#DIV/0!
[TG(48:8),TG(47:1)]_C18:0	0	0	0	0	0	#DIV/0!	#DIV/0!	#DIV/0!
[TG(48:7),TG(47:0)]_C18:0	0	0	0	0	0	#DIV/0!	#DIV/0!	#DIV/0!
[TG(48:7)]_C16:1	0	0	0	0	0	#DIV/0!	#DIV/0!	#DIV/0!
[TG(48:6)]_C14:0	0	0	0	0	0	#DIV/0!	#DIV/0!	#DIV/0!
[TG(48:4)]_C16:1	0	0	0	0	0	#DIV/0!	#DIV/0!	#DIV/0!
[TG(49:6)]_C14:0	0	0	0	0	0	#DIV/0!	#DIV/0!	#DIV/0!
[TG(49:4)]_C16:1	0	0	0	0	0	#DIV/0!	#DIV/0!	#DIV/0!
[TG(50:9),TG(49:2)]_C18:0	0	0	0	0	0	#DIV/0!	#DIV/0!	#DIV/0!
[TG(50:8),TG(49:1)]_C20:0	0	0	0	0	0	#DIV/0!	#DIV/0!	#DIV/0!
[TG(50:8),TG(49:1)]_C18:0	0	0	0	0	0	#DIV/0!	#DIV/0!	#DIV/0!
[TG(50:7)]_C16:1	0	0	0	0	0	#DIV/0!	#DIV/0!	#DIV/0!
[TG(50:7),TG(49:0)]_C14:0	0	0	0	0	0	#DIV/0!	#DIV/0!	#DIV/0!
[TG(50:6)]_C16:0	0	0	0	0	0	#DIV/0!	#DIV/0!	#DIV/0!
[TG(51:7)]_C22:5	0	0	0	0	0	#DIV/0!	#DIV/0!	#DIV/0!
[TG(52:8),TG(51:1)]_C16:1	0	0	0	0	0	#DIV/0!	#DIV/0!	#DIV/0!
[TG(52:8),TG(51:1)]_C14:0	0	0	0	0	0	#DIV/0!	#DIV/0!	#DIV/0!
[TG(52:7),TG(51:0)]_C14:0	0	0	0	0	0	#DIV/0!	#DIV/0!	#DIV/0!
[TG(53:8),TG(52:1)]_C14:0	0	0	0	0	0	#DIV/0!	#DIV/0!	#DIV/0!
[TG(53:7)]_C16:1	0	0	0	0	0	#DIV/0!	#DIV/0!	#DIV/0!
[TG(53:7),TG(52:0)]_C14:0	0	0	0	0	0	#DIV/0!	#DIV/0!	#DIV/0!
[TG(53:6)]_C16:0	0	0	0	0	0	#DIV/0!	#DIV/0!	#DIV/0!
[TG(54:11),TG(53:4)]_C16:1	0	0	0	0	0	#DIV/0!	#DIV/0!	#DIV/0!
[TG(54:10),TG(53:3)]_C16:1	0	0	0	0	0	#DIV/0!	#DIV/0!	#DIV/0!
[TG(54:9),TG(53:2)]_C16:1	0	0	0	0	0	#DIV/0!	#DIV/0!	#DIV/0!
[TG(54:8),TG(53:1)]_C16:1	0	0	0	0	0	#DIV/0!	#DIV/0!	#DIV/0!
[TG(54:7),TG(53:0)]_C14:0	0	0	0	0	0	#DIV/0!	#DIV/0!	#DIV/0!
[TG(55:9),TG(54:2)]_C16:1	0	0	0	0	0	#DIV/0!	#DIV/0!	#DIV/0!
[TG(55:8),TG(54:1)]_C16:1	0	0	0	0	0	#DIV/0!	#DIV/0!	#DIV/0!
[TG(56:10),TG(55:3)]_C16:1	0	0	0	0	0	#DIV/0!	#DIV/0!	#DIV/0!
[TG(56:9),TG(55:2)]_C16:1	0	0	0	0	0	#DIV/0!	#DIV/0!	#DIV/0!

[TG(56:8),TG(55:1)]_C16:1	0	0	0	0	0	#DIV/0!	#DIV/0!	#DIV/0!
[TG(56:8),TG(55:1)]_C14:0	0	0	0	0	0	#DIV/0!	#DIV/0!	#DIV/0!
[TG(56:7)]_C16:1	0	0	0	0	0	#DIV/0!	#DIV/0!	#DIV/0!
[TG(56:7),TG(55:0)]_C14:0	0	0	0	0	0	#DIV/0!	#DIV/0!	#DIV/0!
[TG(57:12),TG(56:5)]_C16:1	0	0	0	0	0	#DIV/0!	#DIV/0!	#DIV/0!
[TG(57:10),TG(56:3)]_C16:1	0	0	0	0	0	#DIV/0!	#DIV/0!	#DIV/0!
[TG(57:9),TG(56:2)]_C16:1	0	0	0	0	0	#DIV/0!	#DIV/0!	#DIV/0!
[TG(57:8),TG(56:1)]_C14:0	0	0	0	0	0	#DIV/0!	#DIV/0!	#DIV/0!
[TG(58:14),TG(57:7),TG(56:0)]_C1	0	0	0	0	0	#DIV/0!	#DIV/0!	#DIV/0!
[TG(58:14),TG(57:7)]_C16:1	0	0	0	0	0	#DIV/0!	#DIV/0!	#DIV/0!
[TG(58:14),TG(57:7),TG(56:0)]_C1	0	0	0	0	0	#DIV/0!	#DIV/0!	#DIV/0!
[TG(58:9),TG(57:2)]_C16:1	0	0	0	0	0	#DIV/0!	#DIV/0!	#DIV/0!
[TG(58:8),TG(57:1)]_C16:0	0	0	0	0	0	#DIV/0!	#DIV/0!	#DIV/0!
[TG(58:8),TG(57:1)]_C16:1	0	0	0	0	0	#DIV/0!	#DIV/0!	#DIV/0!
[TG(58:8),TG(57:1)]_C14:0	0	0	0	0	0	#DIV/0!	#DIV/0!	#DIV/0!
[TG(58:7),TG(57:0)]_C16:0	0	0	0	0	0	#DIV/0!	#DIV/0!	#DIV/0!
[TG(59:10),TG(58:3)]_C16:1	0	0	0	0	0	#DIV/0!	#DIV/0!	#DIV/0!
[TG(59:9),TG(58:2)]_C16:1	0	0	0	0	0	#DIV/0!	#DIV/0!	#DIV/0!
[TG(60:15),TG(59:8),TG(58:1)]_C1	0	0	0	0	0	#DIV/0!	#DIV/0!	#DIV/0!
[TG(60:15),TG(59:8),TG(58:1)]_C1	0	0	0	0	0	#DIV/0!	#DIV/0!	#DIV/0!
[TG(60:15),TG(59:8),TG(58:1)]_C1	0	0	0	0	0	#DIV/0!	#DIV/0!	#DIV/0!
[TG(60:14),TG(59:7),TG(58:0)]_C1	0	0	0	0	0	#DIV/0!	#DIV/0!	#DIV/0!
[TG(60:12),TG(59:5)]_C18:2	0	0	0	0	0	#DIV/0!	#DIV/0!	#DIV/0!
[TG(60:9),TG(59:2)]_C18:1	0	0	0	0	0	#DIV/0!	#DIV/0!	#DIV/0!
[TG(60:9),TG(59:2)]_C16:1	0	0	0	0	0	#DIV/0!	#DIV/0!	#DIV/0!
[TG(60:8),TG(59:1)]_C18:1	0	0	0	0	0	#DIV/0!	#DIV/0!	#DIV/0!
[TG(60:8),TG(59:1)]_C16:0	0	0	0	0	0	#DIV/0!	#DIV/0!	#DIV/0!
[TG(60:8),TG(59:1)]_C16:1	0	0	0	0	0	#DIV/0!	#DIV/0!	#DIV/0!
[TG(61:14),TG(60:7),TG(59:0)]_C1	0	0	0	0	0	#DIV/0!	#DIV/0!	#DIV/0!
[TG(61:13),TG(60:6)]_C20:0	0	0	0	0	0	#DIV/0!	#DIV/0!	#DIV/0!
[TG(61:11),TG(60:4)]_C20:4	0	0	0	0	0	#DIV/0!	#DIV/0!	#DIV/0!
[TG(61:11),TG(60:4)]_C18:0	0	0	0	0	0	#DIV/0!	#DIV/0!	#DIV/0!
[TG(61:10),TG(60:3)]_C16:1	0	0	0	0	0	#DIV/0!	#DIV/0!	#DIV/0!
[TG(62:15),TG(61:8),TG(60:1)]_C1	0	0	0	0	0	#DIV/0!	#DIV/0!	#DIV/0!
[TG(62:15),TG(61:8),TG(60:1)]_C1	0	0	0	0	0	#DIV/0!	#DIV/0!	#DIV/0!
DG(26:0)_C18:0	0	0	0	0	0	#DIV/0!	#DIV/0!	#DIV/0!
DG(28:2)_C18:2	0	0	0	0	0	#DIV/0!	#DIV/0!	#DIV/0!
DG(29:2)_C18:1	0	0	0	0	0	#DIV/0!	#DIV/0!	#DIV/0!
DG(29:1)_C18:1	0	0	0	0	0	#DIV/0!	#DIV/0!	#DIV/0!
DG(29:1)_C16:0	0	0	0	0	0	#DIV/0!	#DIV/0!	#DIV/0!
DG(31:2),DG(P-14:0/18:1)_C18:1	0	0	0	0	0	#DIV/0!	#DIV/0!	#DIV/0!
CE(12:0)H	0	0	0	0	0	#DIV/0!	#DIV/0!	#DIV/0!
DG(31:1)_C16:1	0	0	0	0	0	#DIV/0!	#DIV/0!	#DIV/0!
DG(31:0)_C16:0	0	0	0	0	0	#DIV/0!	#DIV/0!	#DIV/0!
DG(32:2)_C16:1	0	0	0	0	0	#DIV/0!	#DIV/0!	#DIV/0!
DG(33:5)_C18:1	0	0	0	0	0	#DIV/0!	#DIV/0!	#DIV/0!
DG(33:2)_C18:2	0	0	0	0	0	#DIV/0!	#DIV/0!	#DIV/0!
DG(33:2)_C16:0	0	0	0	0	0	#DIV/0!	#DIV/0!	#DIV/0!
DG(33:2)_C16:1	0	0	0	0	0	#DIV/0!	#DIV/0!	#DIV/0!
DG(35:3)_C18:1	0	0	0	0	0	#DIV/0!	#DIV/0!	#DIV/0!
DG(dO-38:9),DG(35:2)_C18:1	0	0	0	0	0	#DIV/0!	#DIV/0!	#DIV/0!
DG(dO-38:9),DG(35:2)_C18:2	0	0	0	0	0	#DIV/0!	#DIV/0!	#DIV/0!
DG(O-38:8),DG(36:1)_C16:0	0	0	0	0	0	#DIV/0!	#DIV/0!	#DIV/0!
DG(38:9),DG(dO-40:9),DG(37:2)_	0	0	0	0	0	#DIV/0!	#DIV/0!	#DIV/0!
DG(38:8),DG(dO-40:8),DG(37:1)_	0	0	0	0	0	#DIV/0!	#DIV/0!	#DIV/0!
DG(38:8),DG(dO-40:8),DG(37:1)_	0	0	0	0	0	#DIV/0!	#DIV/0!	#DIV/0!
DG(38:7)_C18:1	0	0	0	0	0	#DIV/0!	#DIV/0!	#DIV/0!
CE(16:3)K	0	0	0	0	0	#DIV/0!	#DIV/0!	#DIV/0!
CE(16:2)K	0	0	0	0	0	#DIV/0!	#DIV/0!	#DIV/0!
DG(39:8),DG(O-40:8),DG(38:1)_C	0	0	0	0	0	#DIV/0!	#DIV/0!	#DIV/0!
DG(39:8),DG(O-40:8),DG(38:1)_C	0	0	0	0	0	#DIV/0!	#DIV/0!	#DIV/0!
	0	0	0	0	0	#DIV/0!	#DIV/0!	#DIV/0!
CE(17:0)K	0	0	0	0	0	#DIV/0!	#DIV/0!	#DIV/0!

DG(40:9),DG(39:2)_C18:1	0	0	0	0	0	#DIV/0!	#DIV/0!	#DIV/0!
DG(40:8),DG(39:1)_C16:0	0	0	0	0	0	#DIV/0!	#DIV/0!	#DIV/0!
DG(40:8),DG(39:1)_C16:1	0	0	0	0	0	#DIV/0!	#DIV/0!	#DIV/0!
DG(40:7)_C18:1	0	0	0	0	0	#DIV/0!	#DIV/0!	#DIV/0!
DG(40:2)_C18:1	0	0	0	0	0	#DIV/0!	#DIV/0!	#DIV/0!
DG(40:2)_C16:1	0	0	0	0	0	#DIV/0!	#DIV/0!	#DIV/0!
DG(40:1)_C18:1	0	0	0	0	0	#DIV/0!	#DIV/0!	#DIV/0!
DG(40:1)_C16:0	0	0	0	0	0	#DIV/0!	#DIV/0!	#DIV/0!
DG(40:1)_C16:1	0	0	0	0	0	#DIV/0!	#DIV/0!	#DIV/0!
DG(41:7)_C18:1	0	0	0	0	0	#DIV/0!	#DIV/0!	#DIV/0!
DG(42:10),DG(41:3)_C18:1	0	0	0	0	0	#DIV/0!	#DIV/0!	#DIV/0!
DG(42:9),DG(41:2)_C18:1	0	0	0	0	0	#DIV/0!	#DIV/0!	#DIV/0!
DG(42:9),DG(41:2)_C18:2	0	0	0	0	0	#DIV/0!	#DIV/0!	#DIV/0!
DG(42:8),DG(41:1)_C16:1	0	0	0	0	0	#DIV/0!	#DIV/0!	#DIV/0!
DG(42:7),DG(41:0)_C18:0	0	0	0	0	0	#DIV/0!	#DIV/0!	#DIV/0!
DG(42:7)_C18:1	0	0	0	0	0	#DIV/0!	#DIV/0!	#DIV/0!
DG(42:2)_C18:1	0	0	0	0	0	#DIV/0!	#DIV/0!	#DIV/0!
DG(42:2)_C16:1	0	0	0	0	0	#DIV/0!	#DIV/0!	#DIV/0!
DG(42:1)_C18:1	0	0	0	0	0	#DIV/0!	#DIV/0!	#DIV/0!
DG(42:1)_C16:0	0	0	0	0	0	#DIV/0!	#DIV/0!	#DIV/0!
DG(42:1)_C16:1	0	0	0	0	0	#DIV/0!	#DIV/0!	#DIV/0!
DG(42:0)_C18:0	0	0	0	0	0	#DIV/0!	#DIV/0!	#DIV/0!
DG(43:6)_C16:0	0	0	0	0	0	#DIV/0!	#DIV/0!	#DIV/0!
DG(44:9),DG(43:2)_C16:1	0	0	0	0	0	#DIV/0!	#DIV/0!	#DIV/0!
DG(44:8),DG(43:1)_C16:1	0	0	0	0	0	#DIV/0!	#DIV/0!	#DIV/0!
DG(44:7)_C18:1	0	0	0	0	0	#DIV/0!	#DIV/0!	#DIV/0!
DG(44:6)_C16:0	0	0	0	0	0	#DIV/0!	#DIV/0!	#DIV/0!
DG(44:2)_C18:1	0	0	0	0	0	#DIV/0!	#DIV/0!	#DIV/0!
DG(44:2)_C16:1	0	0	0	0	0	#DIV/0!	#DIV/0!	#DIV/0!
DG(44:1)_C16:0	0	0	0	0	0	#DIV/0!	#DIV/0!	#DIV/0!
DG(44:1)_C16:1	0	0	0	0	0	#DIV/0!	#DIV/0!	#DIV/0!
DG(44:0)_C18:0	0	0	0	0	0	#DIV/0!	#DIV/0!	#DIV/0!
FA(7:1)	0	0	0	0	0	#DIV/0!	#DIV/0!	#DIV/0!
FA(7:0)	0	0	0	0	0	#DIV/0!	#DIV/0!	#DIV/0!
FA(8:6)	0	0	0	0	0	#DIV/0!	#DIV/0!	#DIV/0!
FA(8:1)	0	0	0	0	0	#DIV/0!	#DIV/0!	#DIV/0!
FA(8:0)	0	0	0	0	0	#DIV/0!	#DIV/0!	#DIV/0!
FA(10:6)	0	0	0	0	0	#DIV/0!	#DIV/0!	#DIV/0!
FA(10:1)	0	0	0	0	0	#DIV/0!	#DIV/0!	#DIV/0!
FA(10:0); FA(10:0)	0	0	0	0	0	#DIV/0!	#DIV/0!	#DIV/0!
FA(11:6)	0	0	0	0	0	#DIV/0!	#DIV/0!	#DIV/0!
FA(12:5)	0	0	0	0	0	#DIV/0!	#DIV/0!	#DIV/0!
FA(12:0)	0	0	0	0	0	#DIV/0!	#DIV/0!	#DIV/0!
FA(14:7)	0	0	0	0	0	#DIV/0!	#DIV/0!	#DIV/0!
FA(13:0)	0	0	0	0	0	#DIV/0!	#DIV/0!	#DIV/0!
FA(14:0)	0	0	0	0	0	#DIV/0!	#DIV/0!	#DIV/0!
FA(15:6)	0	0	0	0	0	#DIV/0!	#DIV/0!	#DIV/0!
FA(16:5)	0	0	0	0	0	#DIV/0!	#DIV/0!	#DIV/0!
FA(17:4)	0	0	0	0	0	#DIV/0!	#DIV/0!	#DIV/0!
FA(18:7)	0	0	0	0	0	#DIV/0!	#DIV/0!	#DIV/0!
FA(17:0)	0	0	0	0	0	#DIV/0!	#DIV/0!	#DIV/0!
FA(19:4)	0	0	0	0	0	#DIV/0!	#DIV/0!	#DIV/0!
FA(20:4)	0	0	0	0	0	#DIV/0!	#DIV/0!	#DIV/0!
FA(21:3)	0	0	0	0	0	#DIV/0!	#DIV/0!	#DIV/0!
FA(22:4)	0	0	0	0	0	#DIV/0!	#DIV/0!	#DIV/0!
FA(22:3)	0	0	0	0	0	#DIV/0!	#DIV/0!	#DIV/0!
FA(28:7)	0	0	0	0	0	#DIV/0!	#DIV/0!	#DIV/0!
FA(28:5)	0	0	0	0	0	#DIV/0!	#DIV/0!	#DIV/0!
FA(29:3)	0	0	0	0	0	#DIV/0!	#DIV/0!	#DIV/0!
FA(30:5)	0	0	0	0	0	#DIV/0!	#DIV/0!	#DIV/0!
FA(31:1)	0	0	0	0	0	#DIV/0!	#DIV/0!	#DIV/0!
FA(32:6)	0	0	0	0	0	#DIV/0!	#DIV/0!	#DIV/0!
FA(32:4)	0	0	0	0	0	#DIV/0!	#DIV/0!	#DIV/0!

FA(34:5)	0	0	0	0	0	#DIV/0!	#DIV/0!	#DIV/0!
FA(46:0)	0	0	0	0	0	#DIV/0!	#DIV/0!	#DIV/0!
FA(4:0)	0	0	0	0	0	#DIV/0!	#DIV/0!	#DIV/0!

Table S6. Correlation Analysis of Fe3O4 NP-BC Lipid Abundance in Relation to Serum Concentration
BC Lipid Abundance Correlation Analysis with Male 100 nm Samples

Serum concentration (% v/v)	5	10	25	50	75			
	Group Mean Relative Lipid Abundance							
Lipid name	Male 5% 100nm	Male 10% 100nm	Male 25% 100nm	Male 50% 100nm	Male 75% 100nm	Pearson's Correlation	R^2	p-value
CE(18:3)H	2780.115191	4281.140308	9927.83268	16632.78113	23080.4416	0.997431188	0.994869	0.00016
DG(34:1)_C16:0	2899.415204	4404.812337	11541.31681	18701.60134	26459.13391	0.996198647	0.992412	0.00028
CE(15:1)K	4425.220325	4977.480323	8803.844618	12979.93291	16649.28926	0.995385789	0.990793	0.00038
CE(20:5)H	2710.675192	4170.620303	10562.35277	17735.22128	23921.97367	0.995127208	0.990278	0.00041
CE(18:2)Na	6064.980435	11307.59679	38671.99864	66024.76085	93052.4907	0.995035704	0.990096	0.00042
CE(16:0)Na	5844.730433	10620.00879	36908.77435	61814.5005	86738.53798	0.994031162	0.988098	0.00055
CE(16:0)K	2313.57017	4528.48831	11204.43684	18374.18934	24878.65374	0.993662951	0.987366	0.0006
DG(39:8),DG(O-40:8)_C18:2	2658.250184	3467.160246	7515.640518	10585.95272	15174.24112	0.993461823	0.986966	0.00063
CE(18:0)K	3352.25524	6388.94044	18223.68524	30079.50616	40939.56292	0.993095383	0.986238	0.00069
DG(O-38:9),DG(36:2)_C18:1	4107.40531	6981.976501	21269.08963	35856.58229	48200.71954	0.992762712	0.985578	0.00074
DG(34:1)_C18:1	2283.920177	3808.47626	7745.656555	11543.26081	15594.96913	0.992538524	0.985133	0.00077
CE(19:0)H	3687.730257	4698.500324	9001.612645	13782.78497	17422.62129	0.992490308	0.985037	0.00078
DG(34:2)_C18:2	11056.58081	24489.10974	98787.80283	167789.624	231770.1843	0.992124589	0.984311	0.00084
CE(20:4) NH4	1957.760139	3011.416196	6301.760445	9522.768707	12456.52892	0.991257131	0.982591	0.00098
CE(18:2) NH4	11534.28586	24050.60983	97999.6471	161187.0222	222259.1083	0.990532279	0.981154	0.0011
CE(22:1)H	48346.50359	116750.8323	515365.8031	850704.1179	1179381.418	0.990286743	0.980668	0.00115
CE(18:1) NH4	2074.360147	3146.036233	8055.220559	12703.37693	16531.79316	0.989727719	0.979561	0.00125
DG(36:3)_C18:1	7678.080506	16949.50114	63843.21246	104499.6481	141879.8946	0.989560265	0.97923	0.00128
CE(20:3) NH4	1856.035132	2988.012202	5238.000375	7473.208566	9659.548689	0.989020604	0.978162	0.00138
DG(34:4),DG(dO-36:4)_C16:1	3243.870241	4945.292333	14577.36508	22433.76159	30076.80601	0.989000699	0.978122	0.00138
DG(O-40:9),DG(38:2)_C18:2	4798.525308	8883.380643	29889.2101	48665.5592	64223.10452	0.988265325	0.976668	0.00152
DG(39:8),DG(O-40:8),DG(38:1)	44534.51792	106264.36	469894.676	778834.7119	1043488.794	0.987599608	0.975353	0.00165
CE(18:1)Na	7456.795506	14297.78105	57710.4841	95375.65078	125933.7728	0.987206084	0.974576	0.00173
CE(20:5) NH4	2045.545143	3061.416202	6525.132479	9666.080673	12224.98886	0.986535813	0.973253	0.00187
DG(39:7),DG(38:0),DG(dO-40:(	2312.210162	4625.788337	11990.76484	18495.96527	23870.2857	0.985365335	0.970945	0.00212
DG(O-38:8),DG(36:1)_C16:1	2721.930195	3209.716234	5446.24038	7417.748515	8760.124622	0.982260041	0.964835	0.00283
CE(18:3)Na	2254.245156	3812.200279	9129.980615	13154.57299	16846.47315	0.982033269	0.964389	0.00288
CE(16:1) NH4	3239.795228	5035.404346	16334.88519	22423.07367	30824.71826	0.980627775	0.961631	0.00323
CE(18:0) NH4	3130.955222	4019.360275	9790.528728	14475.14912	17575.73325	0.979122069	0.95868	0.00361
CE(20:4)Na	2892.040205	3098.156225	5191.636363	6923.668494	8001.360563	0.979020355	0.958481	0.00364
CE(16:1)Na	0	2693.936189	6259.704439	9828.528706	12581.32889	0.974105413	0.948881	0.00498
DG(34:2)_C16:0	0	2724.308181	6162.792426	9830.012712	12307.92487	0.971938043	0.944664	0.00562
[TG(51:7),TG(50:0)]_C18:0	0	1761.372133	5368.268374	7674.736549	10149.24472	0.970795868	0.942445	0.00596
PC(34:2),PC(O-35:2),PC(P-35:1	0	2480.072173	4470.588314	6521.268454	8636.836626	0.963940364	0.929181	0.00818
PC(14:0),LPC(15:0),LPC(O-16:(	0	1523.036109	3295.732223	4594.168311	6049.688441	0.962683415	0.926759	0.0086
DG(37:7),DG(36:0)_C16:0	4713.000311	5554.152409	10135.94876	13218.01297	14845.93309	0.962082068	0.925602	0.00881
CE(22:4) NH4	5253.085391	8320.748563	15502.80117	23441.8416	25499.68191	0.961868331	0.925191	0.00889
DG(36:3)_C18:2	5001.570352	8515.724603	15908.10105	25496.37378	27148.53396	0.959415577	0.920478	0.00975
DG(44:8),DG(43:1)_C16:0	0	2312.708163	4802.424323	7434.008512	8871.000618	0.958375344	0.918483	0.01013
[TG(54:7)]_C20:4	0	1895.852145	4855.924364	6748.172475	8425.772578	0.956082181	0.914093	0.01098
CE(17:0) NH4	0	2130.060153	4473.076333	5858.564416	7757.136572	0.952526557	0.907307	0.01233
CE(17:1) NH4	0	1493.652106	4232.836302	5156.404365	7086.768502	0.951508528	0.905368	0.01272
DG(39:7)_C18:1	1503.555108	2345.128169	3424.02424	3921.500284	4730.07634	0.948912762	0.900435	0.01375
[TG(50:4)]_C16:0	0	1990.140141	4444.588331	6011.000418	7459.004502	0.947667323	0.898073	0.01426
CE(20:0)H	0	1618.684114	3722.064245	5182.956376	6123.240451	0.942669938	0.888627	0.01634
CE(22:3) NH4	2757.575201	3118.560217	4458.85231	5017.70836	5330.880392	0.928908494	0.862871	0.02251
DG(30:1)_C18:1	1895.225135	2186.540158	3090.432233	3276.79624	3566.220251	0.913430376	0.834355	0.03018
CE(20:0)Na	0	1510.816102	3312.13625	4123.928291	4533.844326	0.894534945	0.800193	0.04046
DG(O-38:9),DG(36:2)_C18:2	0	3105.304221	4163.616288	5877.152399	6480.508434	0.887210311	0.787142	0.04469
CE(20:4)H	0	0	1625.38411	3379.352222	3988.084277	0.974439356	0.949532	0.00489
[TG(48:4)]_C18:2	0	0	1497.75211	2747.780204	3187.184219	0.964784498	0.930809	0.00789
[TG(49:7),TG(48:0)]_C16:0	0	0	1883.428137	3524.876273	3904.340287	0.95743726	0.916686	0.01047
[TG(48:8),TG(47:1)]_C16:0	0	0	15027.48912	17746.23327	27785.74991	0.956614826	0.915112	0.01078
CE(19:0)Na	0	0	1503.336104	2870.872209	3109.944217	0.954273574	0.910638	0.01166
CE(20:5)K	0	0	1529.940107	2846.2722	3075.560213	0.951821294	0.905964	0.0126
	0	0	1483.628099	2820.008199	3024.640218	0.951814968	0.905952	0.0126
CE(20:5)Na	0	0	1710.68412	1819.508128	3243.620231	0.951766184	0.905859	0.01262
DG(O-38:8),DG(36:1)_C18:0	0	0	1564.404105	3059.156212	3113.116213	0.941805656	0.886998	0.0167
CE(22:5)H	2534.840179	2010.932148	3353.756245	3700.184261	4474.044323	0.94102138	0.885521	0.01704

CE(19:0)K	0	0	4353.332312	5461.632392	7006.472491	0.936246033	0.876557	0.01914
DG(32:1)_C18:1	0	0	2191.82416	3097.160227	3545.704259	0.932402101	0.869374	0.02088
CE(22:5) NH4	0	0	2216.548154	3159.544218	3574.060242	0.9309767	0.866718	0.02154
CE(20:4)K	0	0	3267.200222	4323.68031	5115.116364	0.928587675	0.862275	0.02266
CE(20:2)K	0	0	2362.488154	3329.612238	3639.860254	0.920416897	0.847167	0.02663
DG(32:1)_C16:1	1924.975134	1664.784127	3819.224264	4429.448303	4891.140321	0.918201112	0.843093	0.02774
CE(20:1) NH4	0	0	1896.91613	2083.328137	2787.820192	0.916273161	0.839557	0.02871
DG(38:3)_C18:2	0	0	3569.604242	4316.016296	5240.856355	0.916235771	0.839488	0.02873
DG(32:1)_C16:0	0	0	3245.072234	3790.224275	4705.416342	0.913661799	0.834778	0.03006
CE(18:0)H	0	0	1941.336134	2634.600177	2865.956213	0.912023336	0.831787	0.03091
CE(19:0) NH4	0	0	1954.01614	2210.776157	2810.3882	0.911710879	0.831217	0.03107
CE(16:3)Na	0	0	3495.012259	4012.632277	4898.844346	0.90525023	0.819478	0.03451
DG(O-38:9),DG(36:2)_C18:0	0	0	2695.644188	3051.092216	3614.50825	0.893416561	0.798193	0.0411
DG(44:7),DG(43:0)_C16:0	0	0	2745.608207	3295.868227	3647.904238	0.889227072	0.790725	0.04351
CE(16:2)Na	0	0	3651.25625	4395.828307	4829.112337	0.887733841	0.788071	0.04439
DG(O-40:9),DG(38:2)_C18:1	0	0	2739.800201	3123.376224	3595.54824	0.887140275	0.787018	0.04474
CE(22:2)H	0	0	2869.036198	3193.756226	3744.044254	0.885828032	0.784691	0.04551
CE(14:0) NH4	0	0	2824.176196	2978.844212	3605.740264	0.879374244	0.773299	0.04937
CE(22:3)H	0	0	3163.596223	3688.084264	4024.612282	0.877073664	0.769258	0.05077
CE(22:1) NH4	0	0	2709.220181	3168.092234	3419.644241	0.874476056	0.764708	0.05237
CE(20:2) NH4	0	0	2835.5602	3216.600221	3561.864245	0.873941976	0.763775	0.0527
DG(40:5)_C18:0	0	0	2600.224188	2911.660218	3262.420239	0.873868788	0.763647	0.05274
DG(33:0)_C16:0	4179.650277	4221.864291	4496.26031	4461.340316	4526.396336	0.828541789	0.686481	0.083
SM(d16:1/22:0)	3341.425232	3375.392239	3477.064266	3754.104266	3606.852248	0.8149752	0.664185	0.09284
CE(20:2)Na	0	2052.444144	2820.42019	3091.000224	2885.624203	0.696009225	0.484429	0.19176
DG(40:5)_C16:0	2571.660176	0	3114.796219	3518.36824	3856.440266	0.693616584	0.481104	0.19395
DG(34:2)_C16:1	3940.040275	4145.856299	3550.080243	3618.972257	3652.412276	-0.648210878	0.420177	0.23681
DG(34:0)_C18:0	4059.610286	3749.192262	3909.800287	3979.696292	4042.032301	0.423612162	0.179447	0.47724
[TG(52:8),TG(51:1)]_C18:0	3170.50522	2899.792216	3067.432222	3126.460206	3132.352221	0.375296196	0.140847	0.53362
[TG(52:5)]_C20:4	0	0	0	4699.356374	7881.89254	0.967899681	0.93683	0.00687
[TG(56:11),TG(55:4)]_C18:1	0	0	0	1556.908114	2607.704193	0.967898326	0.936827	0.00687
CE(16:2) NH4	0	0	0	1286.724094	2132.212154	0.967868915	0.93677	0.00688
DG(34:3)_C18:2	0	0	0	1444.664104	2475.880165	0.967850296	0.936734	0.00689
[TG(59:10),TG(58:3)]_C18:2	0	0	0	1522.516106	2646.964189	0.967745763	0.936532	0.00692
DG(36:4),DG(O-37:4)_C18:1	0	0	0	1166.292085	2032.888149	0.967721022	0.936484	0.00693
[TG(48:3)]_C18:3	0	0	0	1896.632128	3311.004228	0.967705356	0.936454	0.00693
[TG(56:10),TG(55:3)]_C18:1	0	0	0	1296.468087	2313.81216	0.967412437	0.935887	0.00703
[TG(55:8),TG(54:1)]_C18:1	0	0	0	1146.692073	2079.348151	0.967123552	0.935328	0.00712
[TG(56:6)]_C22:5	0	0	0	2673.844167	4962.860368	0.966588671	0.934294	0.00729
DG(32:2)_C18:2	0	0	0	1427.212093	2649.132173	0.966587536	0.934291	0.00729
CE(20:3)Na	0	0	0	2033.53214	3047.076215	0.965852105	0.93287	0.00754
PC(34:3),PC(P-35:2)	0	0	0	1336.524097	2628.808203	0.96471889	0.930683	0.00791
[TG(56:8)]_C22:6	0	0	0	11059.92082	21971.31764	0.964326377	0.929925	0.00804
[TG(57:12),TG(56:5)]_C16:0	0	0	0	1638.468127	2363.792157	0.964213968	0.929709	0.00808
CE(20:0) NH4	0	0	0	1131.424081	2262.880159	0.964048889	0.92939	0.00814
[TG(54:5)]_C20:4	0	0	0	652434.5715	934686.2254	0.963855133	0.929017	0.0082
PC(39:7),PC(P-40:6),PC(38:0),F	0	0	0	4999.612368	10059.03674	0.963796989	0.928905	0.00822
CE(22:6)Na	0	0	0	3564.052277	7260.016536	0.96325156	0.927854	0.00841
DG(36:4),DG(O-37:4)_C18:2	0	0	0	1889.316138	2659.504193	0.962876077	0.92713	0.00854
CE(22:6) NH4	0	0	0	2296.144151	3210.964227	0.962479926	0.926368	0.00868
LPC(20:4)	0	0	0	2069.836147	2795.548207	0.960117051	0.921825	0.0095
DG(42:7),DG(41:0)_C16:0	0	0	0	4705.916324	10276.10868	0.959687773	0.921001	0.00966
CE(16:0) NH4	0	0	0	2590.000187	3472.720238	0.959564228	0.920764	0.0097
CE(18:3)K	0	0	0	2370.352164	3101.860208	0.957568265	0.916937	0.01043
CE(18:2)K	0	0	0	1107.41608	1429.040103	0.956314897	0.914538	0.01089
CE(22:6)H	0	0	0	1074.720084	1376.864091	0.955637251	0.913243	0.01114
[TG(57:10),TG(56:3)]_C18:2	0	0	0	2687.196187	3291.448239	0.950955642	0.904317	0.01294
DG(40:2)_C18:2	0	0	0	1396.072099	1706.692111	0.950735699	0.903898	0.01303
DG(O-38:8),DG(36:1)_C18:1	0	0	0	2010.668141	2433.924162	0.949591704	0.901724	0.01348
CE(22:4)Na	0	0	0	2053.848147	2372.832175	0.943637828	0.890452	0.01593
[TG(54:11),TG(53:4)]_C18:1	0	0	0	3505.936249	3883.728267	0.937514652	0.878934	0.01857
CE(24:1)H	0	0	0	1153.096082	1275.040084	0.937234664	0.878409	0.0187
CE(18:1)K	0	0	0	1519.516103	1673.732125	0.936632953	0.877281	0.01897
DG(37:7)_C16:1	0	0	0	1481.424107	1551.456107	0.928218148	0.861589	0.02284
[TG(50:3)]_C18:3	0	0	0	2723.704196	2803.900198	0.925123786	0.855854	0.02432
CE(20:0)K	0	0	0	2889.192213	2950.804223	0.923657942	0.853144	0.02503

DG(40:9),DG(39:2)_C18:2	0	0	0	2065.60014	2100.792146	0.922869112	0.851687	0.02541
DG(40:8),DG(39:1)_C18:1	2988.660206	2885.900208	3006.472207	2996.116204	3322.840224	0.845637095	0.715102	0.07109
DG(30:0)_C16:0	0	0	2739.912199	2008.524139	3221.720227	0.829818417	0.688599	0.08209
CE(15:0)K	0	0	19133.51732	12714.91296	17132.60924	0.723193342	0.523009	0.16737
DG(37:6)_C16:0	0	0	1365.980093	0	2475.472179	0.707470638	0.500515	0.18136
DG(37:6)_C18:0	0	0	3088.664217	0	4200.176316	0.636585601	0.405241	0.24815
DG(40:6),DG(dO-40:0)_C16:0	4960.25035	4685.592312	4920.024339	5058.60033	5015.300372	0.623822589	0.389155	0.26077
DG(32:0)_C16:0	3209.820214	2701.264178	3318.352219	2496.804171	2648.216188	-0.581720281	0.338398	0.30353
DG(35:6)_C18:0	312060.8637	297472.1186	334579.1166	324871.979	324944.1593	0.543873349	0.295798	0.34336
PG(16:0),LPG(17:0),LPG(O-18:(	6498.010429	5765.868417	6903.132491	7130.064548	6708.75649	0.542670089	0.294491	0.34465
FA(14:2)	0	0	2764.028185	0	2725.896197	0.52452343	0.275125	0.36419
[TG(44:5)]_C20:0	0	0	18847.86927	0	16941.74512	0.487299731	0.237461	0.40506
DG(34:0)_C16:0	0	0	8679.77665	0	6925.176492	0.437740975	0.191617	0.461
Cer(d14:2(4E,6E)/16:0)	2739.700198	2662.980194	2725.492202	1645.324113	2719.752187	-0.313757154	0.098444	0.60717
	9967.625694	0	10321.47274	0	8223.164537			
CE(22:4)K	0	0	0	1491.896107	1440.756106	0.912606102	0.83285	0.0306
CE(20:1)K	0	0	0	1548.88811	1490.272107	0.911834302	0.831442	0.03101
DG(34:2)_C18:1	0	0	0	2006.228139	1439.048097	0.836792257	0.700221	0.07718
[TG(50:3)]_C16:0	0	0	0	0	17647.46121	0.801783726	0.642857	0.10273
SM(d18:1/25:0)	0	0	0	0	8092.356538	0.801783726	0.642857	0.10273
[TG(54:9),TG(53:2)]_C18:1	0	0	0	0	330080.6392	0.801783726	0.642857	0.10273
PC(36:3),PC(P-37:2)	0	0	0	0	5113.192403	0.801783726	0.642857	0.10273
SM(d18:2/24:1)	0	0	0	0	408671.1567	0.801783726	0.642857	0.10273
PC(O-40:9),PC(38:2),PC(P-39:1	0	0	0	0	226275.7049	0.801783726	0.642857	0.10273
[TG(55:11),TG(54:4)]_C18:1	0	0	0	0	103095.2355	0.801783726	0.642857	0.10273
SM(d16:1/16:0)	0	0	0	0	72248.74899	0.801783726	0.642857	0.10273
SM(d18:1/17:0)	0	0	0	0	14697.64912	0.801783726	0.642857	0.10273
[TG(52:5)]_C18:2	0	0	0	0	12386.31284	0.801783726	0.642857	0.10273
[TG(52:5)]_C18:3	0	0	0	0	12336.47678	0.801783726	0.642857	0.10273
PI(36:2),PI(O-37:2),PI(P-37:1)	0	0	0	0	3452.092255	0.801783726	0.642857	0.10273
[TG(48:3)]_C14:0	0	0	0	0	2321.584167	0.801783726	0.642857	0.10273
PC(42:1)	0	0	0	0	1577.368109	0.801783726	0.642857	0.10273
[TG(46:3)]_C18:2	0	0	0	0	1206.12409	0.801783726	0.642857	0.10273
PC(O-38:9),PC(36:2),PC(O-37:2	0	0	0	0	1072.956069	0.801783726	0.642857	0.10273
PC(38:3)	0	0	0	0	219843.4628	0.801783726	0.642857	0.10273
[TG(53:10),TG(52:3)]_C16:0	0	0	0	0	217320.9592	0.801783726	0.642857	0.10273
SM(d16:1/23:0)	0	0	0	0	18752.24523	0.801783726	0.642857	0.10273
PC(40:4)	0	0	0	0	17970.86127	0.801783726	0.642857	0.10273
[TG(48:3)]_C18:2	0	0	0	0	6795.45642	0.801783726	0.642857	0.10273
SM(d18:2/18:1)	0	0	0	0	3764.640285	0.801783726	0.642857	0.10273
[TG(54:10),TG(53:3)]_C18:2	0	0	0	0	3134.236204	0.801783726	0.642857	0.10273
[TG(49:7),TG(48:0)]_C18:0	0	0	0	0	2380.884156	0.801783726	0.642857	0.10273
[TG(55:9),TG(54:2)]_C20:0	0	0	0	0	1704.992131	0.801783726	0.642857	0.10273
[TG(52:4)]_C18:2	0	0	0	0	140421.9531	0.801783726	0.642857	0.10273
SM(d16:1/18:1)	0	0	0	0	88250.23818	0.801783726	0.642857	0.10273
PC(35:4),PC(O-36:4),PC(P-36:3	0	0	0	0	52759.80352	0.801783726	0.642857	0.10273
SM(d17:1/24:1)	0	0	0	0	49009.2075	0.801783726	0.642857	0.10273
[TG(55:11),TG(54:4)]_C18:2	0	0	0	0	47867.73934	0.801783726	0.642857	0.10273
PC(38:8),PC(37:1),PC(O-38:1),	0	0	0	0	41033.69111	0.801783726	0.642857	0.10273
SM(d16:1/20:1)	0	0	0	0	40877.28723	0.801783726	0.642857	0.10273
[TG(49:8),TG(48:1)]_C16:0	0	0	0	0	36047.17051	0.801783726	0.642857	0.10273
[TG(52:4)]_C18:1	0	0	0	0	23033.84143	0.801783726	0.642857	0.10273
[TG(51:7),TG(50:0)]_C16:0	0	0	0	0	21149.78558	0.801783726	0.642857	0.10273
[TG(55:9),TG(54:2)]_C18:0	0	0	0	0	20563.2975	0.801783726	0.642857	0.10273
PC(36:5)	0	0	0	0	19640.22936	0.801783726	0.642857	0.10273
[TG(49:8),TG(48:1)]_C18:1	0	0	0	0	17610.48915	0.801783726	0.642857	0.10273
SM(d18:1/19:0)	0	0	0	0	17541.92532	0.801783726	0.642857	0.10273
[TG(48:2)]_C16:0	0	0	0	0	15719.50518	0.801783726	0.642857	0.10273
SM(d16:0/23:0)	0	0	0	0	13337.83294	0.801783726	0.642857	0.10273
[TG(53:10),TG(52:3)]_C16:1	0	0	0	0	10137.32471	0.801783726	0.642857	0.10273
[TG(39:0)]_C20:0	0	0	0	0	7033.036501	0.801783726	0.642857	0.10273
[TG(51:7)]_C18:1	0	0	0	0	4504.728328	0.801783726	0.642857	0.10273
PC(40:2)	0	0	0	0	4124.860267	0.801783726	0.642857	0.10273
[TG(57:10),TG(56:3)]_C18:1	0	0	0	0	4056.376286	0.801783726	0.642857	0.10273
[TG(55:8),TG(54:1)]_C18:0	0	0	0	0	4013.296265	0.801783726	0.642857	0.10273
PC(40:1),PC(P-41:0)	0	0	0	0	2809.996207	0.801783726	0.642857	0.10273

[TG(50:5)]_C18:2	0	0	0	0	1255.492093	0.801783726	0.642857	0.10273
[TG(53:9),TG(52:2)]_C18:1	0	0	0	0	486896.8339	0.801783726	0.642857	0.10273
LPC(18:0),PC(O-18:0),LPC(O-1	0	0	0	0	285217.4925	0.801783726	0.642857	0.10273
LPC(16:0),PC(O-16:0),LPC(O-1	0	0	0	0	277366.4753	0.801783726	0.642857	0.10273
SM(d16:1/24:1)	0	0	0	0	246066.9372	0.801783726	0.642857	0.10273
PC(O-38:8),PC(36:1),PC(O-37:1	0	0	0	0	227138.5451	0.801783726	0.642857	0.10273
[TG(53:10),TG(52:3)]_C18:1	0	0	0	0	205668.9347	0.801783726	0.642857	0.10273
SM(d18:2/22:1)	0	0	0	0	194985.5681	0.801783726	0.642857	0.10273
[TG(53:9),TG(52:2)]_C16:0	0	0	0	0	180974.1731	0.801783726	0.642857	0.10273
SM(d18:0/24:1)	0	0	0	0	180916.832	0.801783726	0.642857	0.10273
PC(38:6)	0	0	0	0	120835.7964	0.801783726	0.642857	0.10273
SM(d16:1/20:0)	0	0	0	0	110997.5005	0.801783726	0.642857	0.10273
SM(d16:0/18:0)	0	0	0	0	70373.66449	0.801783726	0.642857	0.10273
[TG(51:8),TG(50:1)]_C18:1	0	0	0	0	67489.07297	0.801783726	0.642857	0.10273
[TG(54:5)]_C18:1	0	0	0	0	62023.03662	0.801783726	0.642857	0.10273
PC(37:3),PC(O-38:3),PC(P-38:2	0	0	0	0	46495.28306	0.801783726	0.642857	0.10273
PC(36:8),PC(35:1),PC(O-36:1),	0	0	0	0	39374.15883	0.801783726	0.642857	0.10273
PC(40:10),PC(39:3),PC(O-40:3)	0	0	0	0	31929.63441	0.801783726	0.642857	0.10273
[TG(55:9),TG(54:2)]_C18:1	0	0	0	0	28102.44601	0.801783726	0.642857	0.10273
LPC(18:1),PC(O-18:1),PC(P-18	0	0	0	0	24436.70586	0.801783726	0.642857	0.10273
[TG(55:10),TG(54:3)]_C18:0	0	0	0	0	23642.06962	0.801783726	0.642857	0.10273
[TG(53:8),TG(52:1)]_C18:0	0	0	0	0	21947.2374	0.801783726	0.642857	0.10273
PC(35:3),PC(O-36:3),PC(P-36:2	0	0	0	0	21786.48141	0.801783726	0.642857	0.10273
[TG(53:8),TG(52:1)]_C16:0	0	0	0	0	21598.72945	0.801783726	0.642857	0.10273
SM(d16:1/17:0)	0	0	0	0	21556.81742	0.801783726	0.642857	0.10273
PC(37:7),PC(P-38:6),PC(36:0),F	0	0	0	0	19038.99726	0.801783726	0.642857	0.10273
PC(39:4),PC(O-40:4),PC(P-40:3	0	0	0	0	18454.77328	0.801783726	0.642857	0.10273
LPC(18:2),LPC(P-19:1)	0	0	0	0	14771.25706	0.801783726	0.642857	0.10273
[TG(50:3)]_C16:1	0	0	0	0	13609.90494	0.801783726	0.642857	0.10273
[TG(48:2)]_C18:2	0	0	0	0	12112.86093	0.801783726	0.642857	0.10273
[TG(48:2)]_C18:1	0	0	0	0	12045.92092	0.801783726	0.642857	0.10273
PC(33:3),PC(O-34:3),PC(P-34:2	0	0	0	0	11542.2128	0.801783726	0.642857	0.10273
PC(37:6),PC(O-38:6),PC(P-38:5	0	0	0	0	11061.47275	0.801783726	0.642857	0.10273
PI(38:4)	0	0	0	0	10287.03276	0.801783726	0.642857	0.10273
SM(d18:2/21:0)	0	0	0	0	10281.94076	0.801783726	0.642857	0.10273
PC(41:5),PC(P-42:4)	0	0	0	0	8557.812563	0.801783726	0.642857	0.10273
PC(39:6),PC(O-40:6),PC(P-40:5	0	0	0	0	8244.42066	0.801783726	0.642857	0.10273
PC(42:11),PC(41:4),PC(O-42:4)	0	0	0	0	7373.50859	0.801783726	0.642857	0.10273
PC(36:7),PC(35:0),PC(O-36:0)	0	0	0	0	6368.480421	0.801783726	0.642857	0.10273
[TG(48:2)]_C14:0	0	0	0	0	6317.616452	0.801783726	0.642857	0.10273
PC(40:3)	0	0	0	0	6235.832515	0.801783726	0.642857	0.10273
[TG(48:2)]_C16:1	0	0	0	0	5124.160387	0.801783726	0.642857	0.10273
PE(38:4)	0	0	0	0	4545.24431	0.801783726	0.642857	0.10273
PE(O-38:9),PE(36:2),PE(O-37:2	0	0	0	0	4128.624342	0.801783726	0.642857	0.10273
[TG(55:10),TG(54:3)]_C16:0	0	0	0	0	3904.940291	0.801783726	0.642857	0.10273
[TG(48:3)]_C16:0	0	0	0	0	3496.196278	0.801783726	0.642857	0.10273
[TG(46:2)]_C16:0	0	0	0	0	3339.384232	0.801783726	0.642857	0.10273
[TG(52:8),TG(51:1)]_C18:1	0	0	0	0	3303.156222	0.801783726	0.642857	0.10273
[TG(54:11),TG(53:4)]_C18:2	0	0	0	0	3173.420223	0.801783726	0.642857	0.10273
PE(34:2),PE(O-35:2),PE(P-35:1)	0	0	0	0	3080.072238	0.801783726	0.642857	0.10273
[TG(57:11),TG(56:4)]_C18:2	0	0	0	0	3032.436211	0.801783726	0.642857	0.10273
CE(17:0)Na	0	0	0	0	2845.416199	0.801783726	0.642857	0.10273
PC(41:6),PC(O-42:6)	0	0	0	0	2819.628191	0.801783726	0.642857	0.10273
DG(44:8),DG(43:1)_C18:1	0	0	0	0	2737.684206	0.801783726	0.642857	0.10273
[TG(52:10),TG(51:3)]_C18:1	0	0	0	0	2736.612201	0.801783726	0.642857	0.10273
DG(34:3)_C16:1	0	0	0	0	2641.256186	0.801783726	0.642857	0.10273
[TG(51:9),TG(50:2)]_C18:0	0	0	0	0	2549.572193	0.801783726	0.642857	0.10273
[TG(48:3)]_C16:1	0	0	0	0	2402.412181	0.801783726	0.642857	0.10273
[TG(54:6)]_C16:0	0	0	0	0	2372.736183	0.801783726	0.642857	0.10273
PC(42:3)	0	0	0	0	2124.20416	0.801783726	0.642857	0.10273
CE(22:6)K	0	0	0	0	2079.868145	0.801783726	0.642857	0.10273
[TG(46:2)]_C16:1	0	0	0	0	2008.156139	0.801783726	0.642857	0.10273
[TG(57:9),TG(56:2)]_C20:0	0	0	0	0	1770.076135	0.801783726	0.642857	0.10273
CE(22:0)K	0	0	0	0	1532.840096	0.801783726	0.642857	0.10273
DG(42:8),DG(41:1)_C18:1	0	0	0	0	1396.576105	0.801783726	0.642857	0.10273
PC(44:2)	0	0	0	0	1395.812096	0.801783726	0.642857	0.10273

PE(38:6)	0	0	0	0	1345.624091	0.801783726	0.642857	0.10273
[TG(56:12),TG(55:5)]_C18:2	0	0	0	0	1314.684098	0.801783726	0.642857	0.10273
[TG(46:3)]_C16:1	0	0	0	0	1229.416088	0.801783726	0.642857	0.10273
PC(44:10),PC(O-44:3)	0	0	0	0	1203.892083	0.801783726	0.642857	0.10273
PC(44:5)	0	0	0	0	1187.908077	0.801783726	0.642857	0.10273
[TG(57:9),TG(56:2)]_C18:0	0	0	0	0	1185.50009	0.801783726	0.642857	0.10273
SM(d16:1/24:0)	0	0	0	0	1066.636073	0.801783726	0.642857	0.10273
SM(d16:0/22:0)	0	0	0	0	245510.4952	0.801783726	0.642857	0.10273
PC(38:5)	0	0	0	0	152943.2099	0.801783726	0.642857	0.10273
[TG(51:8),TG(50:1)]_C16:0	0	0	0	0	116710.6511	0.801783726	0.642857	0.10273
[TG(52:4)]_C16:0	0	0	0	0	94266.25025	0.801783726	0.642857	0.10273
PC(32:0),PC(O-33:0)	0	0	0	0	68329.00024	0.801783726	0.642857	0.10273
PC(39:8),PC(O-40:8),PC(38:1),	0	0	0	0	58748.72452	0.801783726	0.642857	0.10273
PC(37:5),PC(O-38:5),PC(P-38:4	0	0	0	0	34433.33447	0.801783726	0.642857	0.10273
PC(30:2),PC(P-31:1)	0	0	0	0	29476.02999	0.801783726	0.642857	0.10273
[TG(50:3)]_C18:2	0	0	0	0	28947.84212	0.801783726	0.642857	0.10273
PC(32:2),PC(O-33:2),PC(P-33:1	0	0	0	0	24204.74569	0.801783726	0.642857	0.10273
[TG(54:6)]_C18:2	0	0	0	0	23660.43763	0.801783726	0.642857	0.10273
PC(33:2),PC(O-34:2),PC(P-34:1	0	0	0	0	22704.56171	0.801783726	0.642857	0.10273
SM(d18:2/20:1)	0	0	0	0	20388.30546	0.801783726	0.642857	0.10273
[TG(50:3)]_C18:1	0	0	0	0	18882.1533	0.801783726	0.642857	0.10273
SM(d16:0/20:0)	0	0	0	0	16482.82907	0.801783726	0.642857	0.10273
SM(d17:1/26:1)	0	0	0	0	16044.19708	0.801783726	0.642857	0.10273
PC(40:9),PC(39:2),PC(O-40:2),	0	0	0	0	15950.28503	0.801783726	0.642857	0.10273
[TG(52:4)]_C18:3	0	0	0	0	12657.37679	0.801783726	0.642857	0.10273
PC(40:7),PC(39:0),PC(O-40:0)	0	0	0	0	11227.58879	0.801783726	0.642857	0.10273
[TG(52:4)]_C16:1	0	0	0	0	9013.100551	0.801783726	0.642857	0.10273
[TG(54:5)]_C18:3	0	0	0	0	7699.224627	0.801783726	0.642857	0.10273
[TG(51:8)]_C18:2	0	0	0	0	6872.820529	0.801783726	0.642857	0.10273
[TG(56:7)]_C20:4	0	0	0	0	4473.424297	0.801783726	0.642857	0.10273
[TG(50:4)]_C14:0	0	0	0	0	4371.096279	0.801783726	0.642857	0.10273
[TG(57:12),TG(56:5)]_C18:1	0	0	0	0	3274.348209	0.801783726	0.642857	0.10273
[TG(57:9),TG(56:2)]_C18:1	0	0	0	0	2770.352198	0.801783726	0.642857	0.10273
[TG(50:4)]_C16:1	0	0	0	0	2734.51618	0.801783726	0.642857	0.10273
[TG(52:10),TG(51:3)]_C18:2	0	0	0	0	2509.196178	0.801783726	0.642857	0.10273
PI(36:1),PI(O-37:1),PI(P-37:0)	0	0	0	0	2441.268177	0.801783726	0.642857	0.10273
PC(34:1),PC(O-35:1),PC(P-35:(	0	0	0	0	825.7000587	0.801783726	0.642857	0.10273
[TG(51:9),TG(50:2)]_C16:0	0	0	0	0	96845.03498	0.801783726	0.642857	0.10273
FA(10:2)	0	0	0	0	3598.384256	0.801783726	0.642857	0.10273
[TG(56:8)]_C18:2	0	0	0	0	2512.580168	0.801783726	0.642857	0.10273
LPC(22:6)	0	0	0	0	1909.260132	0.801783726	0.642857	0.10273
DG(34:3)_C16:0	0	0	0	0	1836.89613	0.801783726	0.642857	0.10273
SM(d16:1/18:0)	0	0	0	0	692511.1812	0.801783726	0.642857	0.10273
PC(36:4),PC(O-37:4)	0	0	0	0	682475.0876	0.801783726	0.642857	0.10273
[TG(54:5)]_C18:2	0	0	0	0	79147.72552	0.801783726	0.642857	0.10273
SM(d16:1/25:0)	0	0	0	0	63924.7327	0.801783726	0.642857	0.10273
[TG(51:9),TG(50:2)]_C18:1	0	0	0	0	48170.65149	0.801783726	0.642857	0.10273
PC(34:0),PC(O-35:0)	0	0	0	0	47685.17494	0.801783726	0.642857	0.10273
SM(d16:0/24:0)	0	0	0	0	47318.91124	0.801783726	0.642857	0.10273
PC(40:6)	0	0	0	0	46347.92267	0.801783726	0.642857	0.10273
PC(38:9),PC(37:2),PC(O-38:2),	0	0	0	0	41964.29476	0.801783726	0.642857	0.10273
[TG(53:8),TG(52:1)]_C18:1	0	0	0	0	38206.02678	0.801783726	0.642857	0.10273
PC(37:4),PC(O-38:4),PC(P-38:3	0	0	0	0	37394.97846	0.801783726	0.642857	0.10273
SM(d16:1/22:1)	0	0	0	0	33272.45054	0.801783726	0.642857	0.10273
PC(40:5)	0	0	0	0	27316.45006	0.801783726	0.642857	0.10273
PC(35:2),PC(O-36:2),PC(P-36:1	0	0	0	0	25211.42604	0.801783726	0.642857	0.10273
[TG(55:10),TG(54:3)]_C18:2	0	0	0	0	23182.23747	0.801783726	0.642857	0.10273
PC(39:5),PC(O-40:5),PC(P-40:4	0	0	0	0	19637.70146	0.801783726	0.642857	0.10273
PC(28:1),PC(P-29:0)	0	0	0	0	16957.25716	0.801783726	0.642857	0.10273
[TG(46:0)]_C16:0	0	0	0	0	16285.41309	0.801783726	0.642857	0.10273
SM(d16:0/25:0)	0	0	0	0	12628.47685	0.801783726	0.642857	0.10273
[TG(50:4)]_C18:2	0	0	0	0	8432.224629	0.801783726	0.642857	0.10273
[TG(54:6)]_C18:1	0	0	0	0	7980.844545	0.801783726	0.642857	0.10273
PC(31:1),PC(O-32:1),PC(P-32:(	0	0	0	0	7238.436546	0.801783726	0.642857	0.10273
PC(38:7),PC(37:0),PC(O-38:0)	0	0	0	0	6822.224487	0.801783726	0.642857	0.10273
[TG(54:6)]_C18:3	0	0	0	0	6391.144402	0.801783726	0.642857	0.10273

[TG(54:6)]_C20:4	0	0	0	0	6069.328426	0.801783726	0.642857	0.10273
[TG(52:5)]_C16:0	0	0	0	0	5785.588396	0.801783726	0.642857	0.10273
[TG(54:7)]_C18:2	0	0	0	0	5671.392329	0.801783726	0.642857	0.10273
PC(40:8),PC(39:1),PC(O-40:1),	0	0	0	0	5115.724343	0.801783726	0.642857	0.10273
[TG(46:1)]_C18:1	0	0	0	0	5058.476359	0.801783726	0.642857	0.10273
[TG(56:6)]_C20:4	0	0	0	0	4968.860329	0.801783726	0.642857	0.10273
[TG(46:2)]_C18:2	0	0	0	0	4594.876328	0.801783726	0.642857	0.10273
[TG(52:9),TG(51:2)]_C18:1	0	0	0	0	4501.060343	0.801783726	0.642857	0.10273
[TG(54:5)]_C16:0	0	0	0	0	4049.168272	0.801783726	0.642857	0.10273
[TG(53:9),TG(52:2)]_C16:1	0	0	0	0	3811.716279	0.801783726	0.642857	0.10273
[TG(52:5)]_C16:1	0	0	0	0	3529.036234	0.801783726	0.642857	0.10273
[TG(52:9),TG(51:2)]_C16:0	0	0	0	0	3142.164207	0.801783726	0.642857	0.10273
SM(d18:1/12:0)	0	0	0	0	3029.436211	0.801783726	0.642857	0.10273
[TG(52:5)]_C18:1	0	0	0	0	2786.200208	0.801783726	0.642857	0.10273
SM(d18:1/26:1(17Z))	0	0	0	0	2542.816158	0.801783726	0.642857	0.10273
[TG(56:7),TG(55:0)]_C16:0	0	0	0	0	2136.748164	0.801783726	0.642857	0.10273
DG(31:1)_C16:0	0	0	0	0	1846.088124	0.801783726	0.642857	0.10273
[TG(56:7)]_C22:6	0	0	0	0	1805.508116	0.801783726	0.642857	0.10273
LPI(20:0)	0	0	0	0	1761.408113	0.801783726	0.642857	0.10273
CE(22:5)Na	0	0	0	0	1703.580121	0.801783726	0.642857	0.10273
PE(36:3),PE(P-37:2)	0	0	0	0	1694.988117	0.801783726	0.642857	0.10273
PI(34:2),PI(O-35:2),PI(P-35:1)	0	0	0	0	1465.532105	0.801783726	0.642857	0.10273
DG(44:9),DG(43:2)_C18:2	0	0	0	0	1406.776108	0.801783726	0.642857	0.10273
[TG(56:7)]_C22:5	0	0	0	0	1377.028096	0.801783726	0.642857	0.10273
PC(30:1),PC(O-31:1),PC(P-31:(	0	0	0	0	375023.797	0.801783726	0.642857	0.10273
[TG(55:10),TG(54:3)]_C18:1	0	0	0	0	119125.9571	0.801783726	0.642857	0.10273
PC(32:1),PC(O-33:1),PC(P-33:(	0	0	0	0	42804.39511	0.801783726	0.642857	0.10273
[TG(53:9),TG(52:2)]_C18:2	0	0	0	0	28142.93815	0.801783726	0.642857	0.10273
PC(35:5),PC(O-36:5),PC(P-36:4	0	0	0	0	26191.55379	0.801783726	0.642857	0.10273
[TG(51:9),TG(50:2)]_C16:1	0	0	0	0	23223.77381	0.801783726	0.642857	0.10273
[TG(53:9),TG(52:2)]_C18:0	0	0	0	0	17456.31329	0.801783726	0.642857	0.10273
PC(30:0),PC(O-31:0)	0	0	0	0	16611.56527	0.801783726	0.642857	0.10273
[TG(51:9),TG(50:2)]_C14:0	0	0	0	0	11305.01272	0.801783726	0.642857	0.10273
[TG(54:10),TG(53:3)]_C18:1	0	0	0	0	4211.864317	0.801783726	0.642857	0.10273
LPC(20:3)	0	0	0	0	3968.892252	0.801783726	0.642857	0.10273
[TG(56:6)]_C18:2	0	0	0	0	2988.192175	0.801783726	0.642857	0.10273
PC(41:7),PC(P-42:6),PC(40:0),I	0	0	0	0	2979.752213	0.801783726	0.642857	0.10273
[TG(50:9),TG(49:2)]_C18:2	0	0	0	0	2916.640211	0.801783726	0.642857	0.10273
CE(16:1)K	0	0	0	0	2798.044199	0.801783726	0.642857	0.10273
SM(d18:0/15:0)	0	0	0	0	2785.376196	0.801783726	0.642857	0.10273
[TG(57:12),TG(56:5)]_C20:4	0	0	0	0	2672.040188	0.801783726	0.642857	0.10273
LPC(22:5)	0	0	0	0	2180.892167	0.801783726	0.642857	0.10273
PC(42:5)	0	0	0	0	1937.512137	0.801783726	0.642857	0.10273
PC(42:4)	0	0	0	0	1795.836136	0.801783726	0.642857	0.10273
[TG(46:2)]_C14:0	0	0	0	0	1765.212121	0.801783726	0.642857	0.10273
CE(20:1)H	0	0	0	0	1440.992098	0.801783726	0.642857	0.10273
[TG(49:3)]_C18:2	0	0	0	0	1433.124112	0.801783726	0.642857	0.10273
PE(37:6),PE(O-38:6),PE(P-38:5)	0	0	0	0	1031.592072	0.801783726	0.642857	0.10273
[TG(62:16),TG(61:9),TG(60:2)]_	0	0	0	0	988.7480705	0.801783726	0.642857	0.10273
PC(38:4)	0	0	0	0	511372.908	0.801783726	0.642857	0.10273
[TG(53:10),TG(52:3)]_C18:2	0	0	0	0	308138.8355	0.801783726	0.642857	0.10273
SM(d18:0/24:0)	0	0	0	0	10145.07666	0.801783726	0.642857	0.10273
[TG(54:7)]_C18:3	0	0	0	0	8045.084594	0.801783726	0.642857	0.10273
[TG(48:3)]_C18:1	0	0	0	0	3352.648248	0.801783726	0.642857	0.10273
[TG(52:4)]_C20:4	0	0	0	0	3308.700244	0.801783726	0.642857	0.10273
[TG(57:10),TG(56:3)]_C20:0	0	0	0	0	3242.180239	0.801783726	0.642857	0.10273
SM(d18:0/26:1(17Z))	0	0	0	0	1795.132129	0.801783726	0.642857	0.10273
[TG(51:9),TG(50:2)]_C18:2	0	0	0	0	40526.59867	0.801783726	0.642857	0.10273
PC(33:1),PC(O-34:1),PC(P-34:(	0	0	0	0	16342.18525	0.801783726	0.642857	0.10273
[TG(51:8),TG(50:1)]_C14:0	0	0	0	0	3205.120226	0.801783726	0.642857	0.10273
[TG(50:4)]_C18:1	0	0	0	0	2983.048234	0.801783726	0.642857	0.10273
SM(d18:1/24:1(15Z))	0	0	0	0	323431.6508	0.801783726	0.642857	0.10273
[TG(50:3)]_C14:0	0	0	0	0	149867.0301	0.801783726	0.642857	0.10273
[TG(48:8),TG(47:1)]_C18:1	0	0	0	0	1585.256114	0.801783726	0.642857	0.10273
[TG(38:0)]_C20:0	0	0	0	0	1093.096072	0.801783726	0.642857	0.10273
CE(24:1) NH4	0	0	0	0	2481.792172	0.801783726	0.642857	0.10273

FA(15:1)	94382.09216	55361.60788	30500.29001	0	24199.17379	-0.749473328	0.56171	0.14474
Cer(d18:0/21:0)	0	1318.200092	0	0	0	-0.43907204	0.192784	0.45948
DG(38:5)_C16:0	193230.5438	181858.0058	205251.8106	202732.2701	196528.2469	0.431733553	0.186394	0.46789
DG(35:6)_C16:0	247344.3195	230941.3156	259650.6067	258815.0826	249059.6319	0.401647416	0.161321	0.50271
DG(36:7),DG(35:0)_C18:0	6498.830493	6440.264463	6992.468472	6455.492463	6298.628462	-0.377898034	0.142807	0.53056
[TG(59:10),TG(58:3)]_C18:1	0	0	0	1789.252131	0	0.324531508	0.105321	0.59417
DG(42:5)_C18:0	0	0	0	1198.768084	0	0.324531508	0.105321	0.59417
DG(24:0)_C18:0	0	0	0	1109.620087	0	0.324531508	0.105321	0.59417
DG(32:5)_C18:1	0	0	1609.408106	0	0	-0.15272071	0.023324	0.80631
FA(10:3)	0	0	1727.796122	0	0	-0.15272071	0.023324	0.80631
DG(32:2)_C18:1	0	0	17405.25325	0	0	-0.15272071	0.023324	0.80631
DG(37:7),DG(36:0)_C18:0	0	0	1670.416119	0	0	-0.15272071	0.023324	0.80631
DG(32:0)_C18:0	133558.1648	119753.8291	135513.6292	134482.3456	127242.5932	0.067391254	0.004542	0.91426
FA(6:0)	3988.005298	3987.092278	4262.932293	4081.95628	3986.876281	-0.044765209	0.002004	0.94302
CE(18:3) NH4	6451.960464	5879.224398	5477.600374	4401.088313	3719.864262	-0.988897867	0.977919	0.0014
DG(38:7),DG(37:0)_C16:0	12453.91085	0	0	0	0	-0.534522484	0.285714	0.35339
DG(42:6)_C16:0	1273.470082	0	0	0	0	-0.534522484	0.285714	0.35339
FA(22:7)	1580.480108	0	0	0	0	-0.534522484	0.285714	0.35339
FA(28:3)	17158.61115	0	0	0	0	-0.534522484	0.285714	0.35339
FA(19:2)	1711.205113	0	0	0	0	-0.534522484	0.285714	0.35339
FA(17:2)	26463.69702	0	0	0	0	-0.534522484	0.285714	0.35339
FA(26:1)	6176.365419	0	0	0	0	-0.534522484	0.285714	0.35339
LPG(19:0),LPG(O-20:0); LPG(1	9866.825697	0	0	0	0	-0.534522484	0.285714	0.35339
PS(O-29:0)	0	0	0	0	0	#DIV/0!	#DIV/0!	#DIV/0!
[TG(51:8),TG(50:1)]_C18:0	0	0	0	0	0	#DIV/0!	#DIV/0!	#DIV/0!
[TG(53:7),TG(52:0)]_C18:0	0	0	0	0	0	#DIV/0!	#DIV/0!	#DIV/0!
[TG(55:11),TG(54:4)]_C18:0	0	0	0	0	0	#DIV/0!	#DIV/0!	#DIV/0!
[TG(50:7),TG(49:0)]_C16:0	0	0	0	0	0	#DIV/0!	#DIV/0!	#DIV/0!
[TG(53:7),TG(52:0)]_C16:0	0	0	0	0	0	#DIV/0!	#DIV/0!	#DIV/0!
[TG(53:7)]_C18:1	0	0	0	0	0	#DIV/0!	#DIV/0!	#DIV/0!
LPG(20:0); LPG(20:0)	0	0	0	0	0	#DIV/0!	#DIV/0!	#DIV/0!
[TG(51:8),TG(50:1)]_C16:1	0	0	0	0	0	#DIV/0!	#DIV/0!	#DIV/0!
[TG(55:11),TG(54:4)]_C16:0	0	0	0	0	0	#DIV/0!	#DIV/0!	#DIV/0!
PC(29:1),PC(O-30:1),PC(P-30:(	0	0	0	0	0	#DIV/0!	#DIV/0!	#DIV/0!
PC(31:0),PC(O-32:0)	0	0	0	0	0	#DIV/0!	#DIV/0!	#DIV/0!
[TG(49:8),TG(48:1)]_C14:0	0	0	0	0	0	#DIV/0!	#DIV/0!	#DIV/0!
[TG(49:6)]_C16:0	0	0	0	0	0	#DIV/0!	#DIV/0!	#DIV/0!
[TG(57:11),TG(56:4)]_C18:1	0	0	0	0	0	#DIV/0!	#DIV/0!	#DIV/0!
PC(28:0),PC(O-29:0)	0	0	0	0	0	#DIV/0!	#DIV/0!	#DIV/0!
[TG(55:9),TG(54:2)]_C16:0	0	0	0	0	0	#DIV/0!	#DIV/0!	#DIV/0!
[TG(55:9),TG(54:2)]_C18:2	0	0	0	0	0	#DIV/0!	#DIV/0!	#DIV/0!
[TG(49:8),TG(48:1)]_C16:1	0	0	0	0	0	#DIV/0!	#DIV/0!	#DIV/0!
[TG(52:8),TG(51:1)]_C16:0	0	0	0	0	0	#DIV/0!	#DIV/0!	#DIV/0!
[TG(53:10),TG(52:3)]_C18:0	0	0	0	0	0	#DIV/0!	#DIV/0!	#DIV/0!
[TG(56:7)]_C18:2	0	0	0	0	0	#DIV/0!	#DIV/0!	#DIV/0!
Cer(d18:1/24:0)	0	0	0	0	0	#DIV/0!	#DIV/0!	#DIV/0!
[TG(49:7),TG(48:0)]_C14:0	0	0	0	0	0	#DIV/0!	#DIV/0!	#DIV/0!
[TG(46:2)]_C18:1	0	0	0	0	0	#DIV/0!	#DIV/0!	#DIV/0!
[TG(50:8),TG(49:1)]_C18:1	0	0	0	0	0	#DIV/0!	#DIV/0!	#DIV/0!
[TG(54:8),TG(53:1)]_C18:1	0	0	0	0	0	#DIV/0!	#DIV/0!	#DIV/0!
[TG(57:12),TG(56:5)]_C18:2	0	0	0	0	0	#DIV/0!	#DIV/0!	#DIV/0!
LPC(20:2),PC(O-20:2)	0	0	0	0	0	#DIV/0!	#DIV/0!	#DIV/0!
PC(44:12),PC(O-44:5)	0	0	0	0	0	#DIV/0!	#DIV/0!	#DIV/0!
[TG(54:10),TG(53:3)]_C16:0	0	0	0	0	0	#DIV/0!	#DIV/0!	#DIV/0!
[TG(55:8),TG(54:1)]_C16:0	0	0	0	0	0	#DIV/0!	#DIV/0!	#DIV/0!
SM(d16:0/16:0)	0	0	0	0	0	#DIV/0!	#DIV/0!	#DIV/0!
SM(d18:0/17:0)	0	0	0	0	0	#DIV/0!	#DIV/0!	#DIV/0!
[TG(56:6)]_C16:0	0	0	0	0	0	#DIV/0!	#DIV/0!	#DIV/0!
[TG(57:11),TG(56:4)]_C18:0	0	0	0	0	0	#DIV/0!	#DIV/0!	#DIV/0!
PC(29:0),PC(O-30:0)	0	0	0	0	0	#DIV/0!	#DIV/0!	#DIV/0!
[TG(46:0)]_C14:0	0	0	0	0	0	#DIV/0!	#DIV/0!	#DIV/0!
[TG(52:6)]_C18:3	0	0	0	0	0	#DIV/0!	#DIV/0!	#DIV/0!
[TG(50:8),TG(49:1)]_C16:0	0	0	0	0	0	#DIV/0!	#DIV/0!	#DIV/0!
[TG(46:1)]_C16:0	0	0	0	0	0	#DIV/0!	#DIV/0!	#DIV/0!
[TG(54:9),TG(53:2)]_C18:0	0	0	0	0	0	#DIV/0!	#DIV/0!	#DIV/0!
SM(d18:2/14:0)	0	0	0	0	0	#DIV/0!	#DIV/0!	#DIV/0!

[TG(53:8)]_C18:2	0	0	0	0	0	#DIV/0!	#DIV/0!	#DIV/0!
[TG(58:8)]_C22:6	0	0	0	0	0	#DIV/0!	#DIV/0!	#DIV/0!
PC(31:2),PC(O-32:2),PC(P-32:1	0	0	0	0	0	#DIV/0!	#DIV/0!	#DIV/0!
[TG(51:4)]_C18:2	0	0	0	0	0	#DIV/0!	#DIV/0!	#DIV/0!
[TG(54:11),TG(53:4)]_C16:0	0	0	0	0	0	#DIV/0!	#DIV/0!	#DIV/0!
PC(28:2)	0	0	0	0	0	#DIV/0!	#DIV/0!	#DIV/0!
[TG(56:8),TG(55:1)]_C16:0	0	0	0	0	0	#DIV/0!	#DIV/0!	#DIV/0!
[TG(50:9),TG(49:2)]_C16:0	0	0	0	0	0	#DIV/0!	#DIV/0!	#DIV/0!
[TG(55:11),TG(54:4)]_C18:3	0	0	0	0	0	#DIV/0!	#DIV/0!	#DIV/0!
[TG(37:0)]_C18:0	0	0	0	0	0	#DIV/0!	#DIV/0!	#DIV/0!
[TG(53:10),TG(52:3)]_C18:3	0	0	0	0	0	#DIV/0!	#DIV/0!	#DIV/0!
[TG(52:9),TG(51:2)]_C18:2	0	0	0	0	0	#DIV/0!	#DIV/0!	#DIV/0!
[TG(52:10),TG(51:3)]_C16:0	0	0	0	0	0	#DIV/0!	#DIV/0!	#DIV/0!
[TG(55:11),TG(54:4)]_C20:4	0	0	0	0	0	#DIV/0!	#DIV/0!	#DIV/0!
PC(33:0),PC(O-34:0)	0	0	0	0	0	#DIV/0!	#DIV/0!	#DIV/0!
[TG(56:8)]_C20:4	0	0	0	0	0	#DIV/0!	#DIV/0!	#DIV/0!
PC(42:10),PC(41:3),PC(O-42:3)	0	0	0	0	0	#DIV/0!	#DIV/0!	#DIV/0!
[TG(50:4)]_C18:3	0	0	0	0	0	#DIV/0!	#DIV/0!	#DIV/0!
[TG(57:12),TG(56:5)]_C18:0	0	0	0	0	0	#DIV/0!	#DIV/0!	#DIV/0!
[TG(54:9),TG(53:2)]_C16:0	0	0	0	0	0	#DIV/0!	#DIV/0!	#DIV/0!
[TG(55:8),TG(54:1)]_C20:0	0	0	0	0	0	#DIV/0!	#DIV/0!	#DIV/0!
PC(35:6),PC(P-36:5)	0	0	0	0	0	#DIV/0!	#DIV/0!	#DIV/0!
PC(42:9),PC(41:2),PC(O-42:2),	0	0	0	0	0	#DIV/0!	#DIV/0!	#DIV/0!
PC(42:8),PC(41:1),PC(O-42:1),	0	0	0	0	0	#DIV/0!	#DIV/0!	#DIV/0!
PC(43:6)	0	0	0	0	0	#DIV/0!	#DIV/0!	#DIV/0!
PC(42:0)	0	0	0	0	0	#DIV/0!	#DIV/0!	#DIV/0!
[TG(56:12),TG(55:5)]_C18:1	0	0	0	0	0	#DIV/0!	#DIV/0!	#DIV/0!
LPC(22:4)	0	0	0	0	0	#DIV/0!	#DIV/0!	#DIV/0!
PC(42:2)	0	0	0	0	0	#DIV/0!	#DIV/0!	#DIV/0!
PC(42:7),PC(41:0),PC(O-42:0)	0	0	0	0	0	#DIV/0!	#DIV/0!	#DIV/0!
PC(43:4),PC(O-44:4)	0	0	0	0	0	#DIV/0!	#DIV/0!	#DIV/0!
[TG(56:6)]_C18:0	0	0	0	0	0	#DIV/0!	#DIV/0!	#DIV/0!
[TG(58:7)]_C22:5	0	0	0	0	0	#DIV/0!	#DIV/0!	#DIV/0!
[TG(44:0),TG(O-45:0)]_C16:0	0	0	0	0	0	#DIV/0!	#DIV/0!	#DIV/0!
[TG(54:8),TG(53:1)]_C18:0	0	0	0	0	0	#DIV/0!	#DIV/0!	#DIV/0!
[TG(54:5)]_C22:5	0	0	0	0	0	#DIV/0!	#DIV/0!	#DIV/0!
[TG(38:0)]_C14:0	0	0	0	0	0	#DIV/0!	#DIV/0!	#DIV/0!
[TG(54:8),TG(53:1)]_C16:0	0	0	0	0	0	#DIV/0!	#DIV/0!	#DIV/0!
[TG(57:12),TG(56:5)]_C22:5	0	0	0	0	0	#DIV/0!	#DIV/0!	#DIV/0!
PS(38:4)	0	0	0	0	0	#DIV/0!	#DIV/0!	#DIV/0!
[TG(54:9),TG(53:2)]_C18:2	0	0	0	0	0	#DIV/0!	#DIV/0!	#DIV/0!
Cer(d18:1/23:0)	0	0	0	0	0	#DIV/0!	#DIV/0!	#DIV/0!
[TG(58:9)]_C22:6	0	0	0	0	0	#DIV/0!	#DIV/0!	#DIV/0!
[TG(46:1)]_C14:0	0	0	0	0	0	#DIV/0!	#DIV/0!	#DIV/0!
[TG(49:7)]_C18:1	0	0	0	0	0	#DIV/0!	#DIV/0!	#DIV/0!
[TG(54:5)]_C18:0	0	0	0	0	0	#DIV/0!	#DIV/0!	#DIV/0!
PC(16:0),PC(O-17:0),LPC(O-18	0	0	0	0	0	#DIV/0!	#DIV/0!	#DIV/0!
Cer(d18:1/22:0)	0	0	0	0	0	#DIV/0!	#DIV/0!	#DIV/0!
PC(42:6)	0	0	0	0	0	#DIV/0!	#DIV/0!	#DIV/0!
PC(34:6)	0	0	0	0	0	#DIV/0!	#DIV/0!	#DIV/0!
PI(38:3)	0	0	0	0	0	#DIV/0!	#DIV/0!	#DIV/0!
[TG(42:0)]_C16:0	0	0	0	0	0	#DIV/0!	#DIV/0!	#DIV/0!
[TG(50:9),TG(49:2)]_C18:1	0	0	0	0	0	#DIV/0!	#DIV/0!	#DIV/0!
[TG(49:8),TG(48:1)]_C18:0	0	0	0	0	0	#DIV/0!	#DIV/0!	#DIV/0!
[TG(55:7),TG(54:0)]_C20:0	0	0	0	0	0	#DIV/0!	#DIV/0!	#DIV/0!
[TG(53:8),TG(52:1)]_C20:0	0	0	0	0	0	#DIV/0!	#DIV/0!	#DIV/0!
[TG(46:1)]_C16:1	0	0	0	0	0	#DIV/0!	#DIV/0!	#DIV/0!
PE(34:1),PE(O-35:1),PE(P-35:0)	0	0	0	0	0	#DIV/0!	#DIV/0!	#DIV/0!
[TG(44:1)]_C16:0	0	0	0	0	0	#DIV/0!	#DIV/0!	#DIV/0!
CAR(14:1)	0	0	0	0	0	#DIV/0!	#DIV/0!	#DIV/0!
[TG(52:7),TG(51:0)]_C16:0	0	0	0	0	0	#DIV/0!	#DIV/0!	#DIV/0!
[TG(57:10),TG(56:3)]_C18:0	0	0	0	0	0	#DIV/0!	#DIV/0!	#DIV/0!
PC(36:6)	0	0	0	0	0	#DIV/0!	#DIV/0!	#DIV/0!
PC(19:1),LPC(20:1),PC(O-20:1)	0	0	0	0	0	#DIV/0!	#DIV/0!	#DIV/0!
PC(32:3),PC(P-33:2)	0	0	0	0	0	#DIV/0!	#DIV/0!	#DIV/0!
[TG(58:8)]_C22:5	0	0	0	0	0	#DIV/0!	#DIV/0!	#DIV/0!

Cer(d18:1/24:1(15Z))	0	0	0	0	0	#DIV/0!	#DIV/0!	#DIV/0!
[TG(46:0)]_C18:0	0	0	0	0	0	#DIV/0!	#DIV/0!	#DIV/0!
[TG(56:9),TG(55:2)]_C18:1	0	0	0	0	0	#DIV/0!	#DIV/0!	#DIV/0!
[TG(52:7),TG(51:0)]_C18:0	0	0	0	0	0	#DIV/0!	#DIV/0!	#DIV/0!
PC(29:2),PC(P-30:1)	0	0	0	0	0	#DIV/0!	#DIV/0!	#DIV/0!
[TG(49:8)]_C18:2	0	0	0	0	0	#DIV/0!	#DIV/0!	#DIV/0!
[TG(52:6)]_C18:2	0	0	0	0	0	#DIV/0!	#DIV/0!	#DIV/0!
[TG(57:11),TG(56:4)]_C20:0	0	0	0	0	0	#DIV/0!	#DIV/0!	#DIV/0!
[TG(53:7),TG(52:0)]_C20:0	0	0	0	0	0	#DIV/0!	#DIV/0!	#DIV/0!
[TG(57:8),TG(56:1)]_C20:0	0	0	0	0	0	#DIV/0!	#DIV/0!	#DIV/0!
1-O-tricosanoyl-Cer(d18:1/16:0	0	0	0	0	0	#DIV/0!	#DIV/0!	#DIV/0!
SM(d17:0/27:0)	0	0	0	0	0	#DIV/0!	#DIV/0!	#DIV/0!
[TG(58:7)]_C18:1	0	0	0	0	0	#DIV/0!	#DIV/0!	#DIV/0!
[TG(38:1)]_C18:1	0	0	0	0	0	#DIV/0!	#DIV/0!	#DIV/0!
[TG(53:8),TG(52:1)]_C16:1	0	0	0	0	0	#DIV/0!	#DIV/0!	#DIV/0!
[TG(44:1)]_C18:1	0	0	0	0	0	#DIV/0!	#DIV/0!	#DIV/0!
[TG(55:7)]_C18:1	0	0	0	0	0	#DIV/0!	#DIV/0!	#DIV/0!
[TG(54:7),TG(53:0)]_C18:0	0	0	0	0	0	#DIV/0!	#DIV/0!	#DIV/0!
[TG(51:6)]_C16:0	0	0	0	0	0	#DIV/0!	#DIV/0!	#DIV/0!
[TG(54:5)]_C16:1	0	0	0	0	0	#DIV/0!	#DIV/0!	#DIV/0!
[TG(49:7)]_C16:1	0	0	0	0	0	#DIV/0!	#DIV/0!	#DIV/0!
PC(44:0)	0	0	0	0	0	#DIV/0!	#DIV/0!	#DIV/0!
PE(40:6),PE(dO-40:0)	0	0	0	0	0	#DIV/0!	#DIV/0!	#DIV/0!
PS(P-37:0)	0	0	0	0	0	#DIV/0!	#DIV/0!	#DIV/0!
PE(O-38:8),PE(36:1),PE(O-37:1	0	0	0	0	0	#DIV/0!	#DIV/0!	#DIV/0!
[TG(52:9),TG(51:2)]_C16:1	0	0	0	0	0	#DIV/0!	#DIV/0!	#DIV/0!
PE(36:4),PE(O-37:4)	0	0	0	0	0	#DIV/0!	#DIV/0!	#DIV/0!
[TG(52:6)]_C16:0	0	0	0	0	0	#DIV/0!	#DIV/0!	#DIV/0!
[TG(59:13),TG(58:6)]_C18:1	0	0	0	0	0	#DIV/0!	#DIV/0!	#DIV/0!
[TG(40:0)]_C16:0	0	0	0	0	0	#DIV/0!	#DIV/0!	#DIV/0!
[TG(38:0)]_C18:0	0	0	0	0	0	#DIV/0!	#DIV/0!	#DIV/0!
[TG(52:6)]_C16:1	0	0	0	0	0	#DIV/0!	#DIV/0!	#DIV/0!
[TG(58:8),TG(57:1)]_C18:1	0	0	0	0	0	#DIV/0!	#DIV/0!	#DIV/0!
CAR(14:2)	0	0	0	0	0	#DIV/0!	#DIV/0!	#DIV/0!
[TG(50:7),TG(49:0)]_C18:0	0	0	0	0	0	#DIV/0!	#DIV/0!	#DIV/0!
[TG(44:2)]_C16:0	0	0	0	0	0	#DIV/0!	#DIV/0!	#DIV/0!
SM(d18:2/15:0)	0	0	0	0	0	#DIV/0!	#DIV/0!	#DIV/0!
PC(24:0)	0	0	0	0	0	#DIV/0!	#DIV/0!	#DIV/0!
[TG(54:12),TG(53:5)]_C18:2	0	0	0	0	0	#DIV/0!	#DIV/0!	#DIV/0!
[TG(51:6)]_C18:0	0	0	0	0	0	#DIV/0!	#DIV/0!	#DIV/0!
[TG(44:0),TG(O-45:0)]_C18:0	0	0	0	0	0	#DIV/0!	#DIV/0!	#DIV/0!
[TG(56:8),TG(55:1)]_C18:1	0	0	0	0	0	#DIV/0!	#DIV/0!	#DIV/0!
[TG(46:3)]_C18:1	0	0	0	0	0	#DIV/0!	#DIV/0!	#DIV/0!
[TG(55:11),TG(54:4)]_C16:1	0	0	0	0	0	#DIV/0!	#DIV/0!	#DIV/0!
[TG(48:7),TG(47:0)]_C16:0	0	0	0	0	0	#DIV/0!	#DIV/0!	#DIV/0!
PE(38:5)	0	0	0	0	0	#DIV/0!	#DIV/0!	#DIV/0!
[TG(44:0),TG(O-45:0)]_C14:0	0	0	0	0	0	#DIV/0!	#DIV/0!	#DIV/0!
[TG(55:10),TG(54:3)]_C20:0	0	0	0	0	0	#DIV/0!	#DIV/0!	#DIV/0!
Cer(d18:1/16:0)	0	0	0	0	0	#DIV/0!	#DIV/0!	#DIV/0!
FA(18:0)	0	0	0	0	0	#DIV/0!	#DIV/0!	#DIV/0!
LPE(22:4)	0	0	0	0	0	#DIV/0!	#DIV/0!	#DIV/0!
DG(30:2)_C16:0	0	0	0	0	0	#DIV/0!	#DIV/0!	#DIV/0!
DG(30:3)_C16:1	0	0	0	0	0	#DIV/0!	#DIV/0!	#DIV/0!
DG(36:8),DG(35:1)_C16:1	0	0	0	0	0	#DIV/0!	#DIV/0!	#DIV/0!
[TG(42:0)]_C14:0	0	0	0	0	0	#DIV/0!	#DIV/0!	#DIV/0!
CAR(20:0)	0	0	0	0	0	#DIV/0!	#DIV/0!	#DIV/0!
DG(36:7),DG(35:0)_C16:0	0	0	0	0	0	#DIV/0!	#DIV/0!	#DIV/0!
DG(36:6)_C16:0	0	0	0	0	0	#DIV/0!	#DIV/0!	#DIV/0!
DG(41:5)_C16:0	0	0	0	0	0	#DIV/0!	#DIV/0!	#DIV/0!
DG(42:11),DG(41:4)_C16:0	0	0	0	0	0	#DIV/0!	#DIV/0!	#DIV/0!
DG(30:2)_C16:1	0	0	0	0	0	#DIV/0!	#DIV/0!	#DIV/0!
DG(36:7)_C16:1	0	0	0	0	0	#DIV/0!	#DIV/0!	#DIV/0!
CE(15:1) NH4	0	0	0	0	0	#DIV/0!	#DIV/0!	#DIV/0!
DG(41:6)_C16:1	0	0	0	0	0	#DIV/0!	#DIV/0!	#DIV/0!
DG(36:8),DG(35:1)_C18:1	0	0	0	0	0	#DIV/0!	#DIV/0!	#DIV/0!
Cer(d18:0/17:0)	0	0	0	0	0	#DIV/0!	#DIV/0!	#DIV/0!

FA(21:0)	0	0	0	0	0	#DIV/0!	#DIV/0!	#DIV/0!
DG(36:5)_C16:0	0	0	0	0	0	#DIV/0!	#DIV/0!	#DIV/0!
DG(30:1)_C16:0	0	0	0	0	0	#DIV/0!	#DIV/0!	#DIV/0!
DG(36:6)_C16:1	0	0	0	0	0	#DIV/0!	#DIV/0!	#DIV/0!
FA(24:4)	0	0	0	0	0	#DIV/0!	#DIV/0!	#DIV/0!
[TG(57:11),TG(56:4)]_C16:0	0	0	0	0	0	#DIV/0!	#DIV/0!	#DIV/0!
PC(30:3)	0	0	0	0	0	#DIV/0!	#DIV/0!	#DIV/0!
FA(30:0)	0	0	0	0	0	#DIV/0!	#DIV/0!	#DIV/0!
FA(18:3)	0	0	0	0	0	#DIV/0!	#DIV/0!	#DIV/0!
DG(34:1)_C16:1	0	0	0	0	0	#DIV/0!	#DIV/0!	#DIV/0!
LPI(19:0),LPI(O-20:0)	0	0	0	0	0	#DIV/0!	#DIV/0!	#DIV/0!
DG(41:6)_C18:1	0	0	0	0	0	#DIV/0!	#DIV/0!	#DIV/0!
CE(15:0) NH4	0	0	0	0	0	#DIV/0!	#DIV/0!	#DIV/0!
[TG(48:8),TG(47:1)]_C16:1	0	0	0	0	0	#DIV/0!	#DIV/0!	#DIV/0!
PG(20:0),LPG(21:0); PG(20:0),L	0	0	0	0	0	#DIV/0!	#DIV/0!	#DIV/0!
PC(31:3),PC(O-32:3)	0	0	0	0	0	#DIV/0!	#DIV/0!	#DIV/0!
[TG(58:8)]_C18:2	0	0	0	0	0	#DIV/0!	#DIV/0!	#DIV/0!
FA(20:0)	0	0	0	0	0	#DIV/0!	#DIV/0!	#DIV/0!
[TG(45:3)]_C16:0	0	0	0	0	0	#DIV/0!	#DIV/0!	#DIV/0!
CE(20:1)Na	0	0	0	0	0	#DIV/0!	#DIV/0!	#DIV/0!
CE(16:0)H	0	0	0	0	0	#DIV/0!	#DIV/0!	#DIV/0!
[TG(50:9),TG(49:2)]_C16:1	0	0	0	0	0	#DIV/0!	#DIV/0!	#DIV/0!
DG(41:5)_C16:1	0	0	0	0	0	#DIV/0!	#DIV/0!	#DIV/0!
CE(22:4)H	0	0	0	0	0	#DIV/0!	#DIV/0!	#DIV/0!
LPG(18:0); LPG(18:0)	0	0	0	0	0	#DIV/0!	#DIV/0!	#DIV/0!
PC(44:1)	0	0	0	0	0	#DIV/0!	#DIV/0!	#DIV/0!
[TG(58:9),TG(57:2)]_C18:1	0	0	0	0	0	#DIV/0!	#DIV/0!	#DIV/0!
[TG(57:8),TG(56:1)]_C18:0	0	0	0	0	0	#DIV/0!	#DIV/0!	#DIV/0!
[TG(49:3)]_C16:0	0	0	0	0	0	#DIV/0!	#DIV/0!	#DIV/0!
[TG(52:10),TG(51:3)]_C16:1	0	0	0	0	0	#DIV/0!	#DIV/0!	#DIV/0!
1-O-pentacosanoyl-Cer(d18:1/	0	0	0	0	0	#DIV/0!	#DIV/0!	#DIV/0!
FA(16:0)	0	0	0	0	0	#DIV/0!	#DIV/0!	#DIV/0!
[TG(50:9),TG(49:2)]_C20:0	0	0	0	0	0	#DIV/0!	#DIV/0!	#DIV/0!
[TG(47:6)]_C16:0	0	0	0	0	0	#DIV/0!	#DIV/0!	#DIV/0!
[TG(53:9),TG(52:2)]_C20:0	0	0	0	0	0	#DIV/0!	#DIV/0!	#DIV/0!
[TG(50:5)]_C18:3	0	0	0	0	0	#DIV/0!	#DIV/0!	#DIV/0!
CE(24:1)Na	0	0	0	0	0	#DIV/0!	#DIV/0!	#DIV/0!
[TG(56:11),TG(55:4)]_C18:2	0	0	0	0	0	#DIV/0!	#DIV/0!	#DIV/0!
FA(22:1)	0	0	0	0	0	#DIV/0!	#DIV/0!	#DIV/0!
[TG(51:7),TG(50:0)]_C14:0	0	0	0	0	0	#DIV/0!	#DIV/0!	#DIV/0!
[TG(42:1)]_C18:1	0	0	0	0	0	#DIV/0!	#DIV/0!	#DIV/0!
CAR(20:1)	0	0	0	0	0	#DIV/0!	#DIV/0!	#DIV/0!
[TG(50:8),TG(49:1)]_C16:1	0	0	0	0	0	#DIV/0!	#DIV/0!	#DIV/0!
PG(25:0); PG(25:0)	0	0	0	0	0	#DIV/0!	#DIV/0!	#DIV/0!
[TG(45:0)]_C16:0	0	0	0	0	0	#DIV/0!	#DIV/0!	#DIV/0!
[TG(57:8),TG(56:1)]_C18:1	0	0	0	0	0	#DIV/0!	#DIV/0!	#DIV/0!
PG(O-35:1),PG(P-35:0); PG(O-3	0	0	0	0	0	#DIV/0!	#DIV/0!	#DIV/0!
[TG(54:7)]_C18:1	0	0	0	0	0	#DIV/0!	#DIV/0!	#DIV/0!
PE(37:5),PE(O-38:5),PE(P-38:4)	0	0	0	0	0	#DIV/0!	#DIV/0!	#DIV/0!
PC(44:3)	0	0	0	0	0	#DIV/0!	#DIV/0!	#DIV/0!
PE(38:3)	0	0	0	0	0	#DIV/0!	#DIV/0!	#DIV/0!
FA(22:0)	0	0	0	0	0	#DIV/0!	#DIV/0!	#DIV/0!
FA(37:0)	0	0	0	0	0	#DIV/0!	#DIV/0!	#DIV/0!
CAR(18:3)	0	0	0	0	0	#DIV/0!	#DIV/0!	#DIV/0!
FA(34:6)	0	0	0	0	0	#DIV/0!	#DIV/0!	#DIV/0!
[TG(42:0)]_C18:0	0	0	0	0	0	#DIV/0!	#DIV/0!	#DIV/0!
FA(19:0)	0	0	0	0	0	#DIV/0!	#DIV/0!	#DIV/0!
FA(26:6)	0	0	0	0	0	#DIV/0!	#DIV/0!	#DIV/0!
FA(23:0)	0	0	0	0	0	#DIV/0!	#DIV/0!	#DIV/0!
[TG(55:7),TG(54:0)]_C16:0	0	0	0	0	0	#DIV/0!	#DIV/0!	#DIV/0!
[TG(44:2)]_C18:2	0	0	0	0	0	#DIV/0!	#DIV/0!	#DIV/0!
[TG(50:8),TG(49:1)]_C14:0	0	0	0	0	0	#DIV/0!	#DIV/0!	#DIV/0!
[TG(52:5)]_C22:5	0	0	0	0	0	#DIV/0!	#DIV/0!	#DIV/0!
PI(38:5)	0	0	0	0	0	#DIV/0!	#DIV/0!	#DIV/0!
[TG(52:9),TG(51:2)]_C18:0	0	0	0	0	0	#DIV/0!	#DIV/0!	#DIV/0!
[TG(52:7)]_C18:1	0	0	0	0	0	#DIV/0!	#DIV/0!	#DIV/0!

LPC(14:0),PC(O-14:0),LPC(O-1	0	0	0	0	0	#DIV/0!	#DIV/0!	#DIV/0!
[TG(45:2)]_C16:0	0	0	0	0	0	#DIV/0!	#DIV/0!	#DIV/0!
FA(21:1)	0	0	0	0	0	#DIV/0!	#DIV/0!	#DIV/0!
CAR(18:2)	0	0	0	0	0	#DIV/0!	#DIV/0!	#DIV/0!
[TG(45:1)]_C16:1	0	0	0	0	0	#DIV/0!	#DIV/0!	#DIV/0!
FA(26:2)	0	0	0	0	0	#DIV/0!	#DIV/0!	#DIV/0!
DG(41:6)_C16:0	0	0	0	0	0	#DIV/0!	#DIV/0!	#DIV/0!
LPS(P-16:0)	0	0	0	0	0	#DIV/0!	#DIV/0!	#DIV/0!
[TG(45:4)]_C20:0	0	0	0	0	0	#DIV/0!	#DIV/0!	#DIV/0!
DG(30:1)_C16:1	0	0	0	0	0	#DIV/0!	#DIV/0!	#DIV/0!
DG(41:5)_C18:0	0	0	0	0	0	#DIV/0!	#DIV/0!	#DIV/0!
DG(35:3)_C18:0	0	0	0	0	0	#DIV/0!	#DIV/0!	#DIV/0!
FA(34:0)	0	0	0	0	0	#DIV/0!	#DIV/0!	#DIV/0!
[TG(47:2)]_C16:1	0	0	0	0	0	#DIV/0!	#DIV/0!	#DIV/0!
[TG(44:1)]_C14:0	0	0	0	0	0	#DIV/0!	#DIV/0!	#DIV/0!
PC(27:0),PC(O-28:0)	0	0	0	0	0	#DIV/0!	#DIV/0!	#DIV/0!
[TG(51:7)]_C16:1	0	0	0	0	0	#DIV/0!	#DIV/0!	#DIV/0!
[TG(57:9),TG(56:2)]_C18:2	0	0	0	0	0	#DIV/0!	#DIV/0!	#DIV/0!
[TG(57:8),TG(56:1)]_C16:0	0	0	0	0	0	#DIV/0!	#DIV/0!	#DIV/0!
[TG(57:9),TG(56:2)]_C16:0	0	0	0	0	0	#DIV/0!	#DIV/0!	#DIV/0!
PC(19:0),LPC(20:0),PC(O-20:0)	0	0	0	0	0	#DIV/0!	#DIV/0!	#DIV/0!
[TG(46:1)]_C18:0	0	0	0	0	0	#DIV/0!	#DIV/0!	#DIV/0!
[TG(48:7),TG(47:0)]_C14:0	0	0	0	0	0	#DIV/0!	#DIV/0!	#DIV/0!
[TG(56:8)]_C18:3	0	0	0	0	0	#DIV/0!	#DIV/0!	#DIV/0!
[TG(48:4)]_C18:1	0	0	0	0	0	#DIV/0!	#DIV/0!	#DIV/0!
[TG(48:6)]_C16:0	0	0	0	0	0	#DIV/0!	#DIV/0!	#DIV/0!
CE(14:1) NH4	0	0	0	0	0	#DIV/0!	#DIV/0!	#DIV/0!
[TG(48:4)]_C18:3	0	0	0	0	0	#DIV/0!	#DIV/0!	#DIV/0!
[TG(39:0)]_C16:0	0	0	0	0	0	#DIV/0!	#DIV/0!	#DIV/0!
[TG(56:13),TG(55:6)]_C18:2	0	0	0	0	0	#DIV/0!	#DIV/0!	#DIV/0!
FA(38:6)	0	0	0	0	0	#DIV/0!	#DIV/0!	#DIV/0!
[TG(48:6)]_C18:0	0	0	0	0	0	#DIV/0!	#DIV/0!	#DIV/0!
PI(38:2),PI(P-39:1)	0	0	0	0	0	#DIV/0!	#DIV/0!	#DIV/0!
DG(36:8),DG(35:1)_C18:0	0	0	0	0	0	#DIV/0!	#DIV/0!	#DIV/0!
PC(44:7),PC(43:0)	0	0	0	0	0	#DIV/0!	#DIV/0!	#DIV/0!
[TG(37:0)]_C14:0	0	0	0	0	0	#DIV/0!	#DIV/0!	#DIV/0!
[TG(55:10),TG(54:3)]_C16:1	0	0	0	0	0	#DIV/0!	#DIV/0!	#DIV/0!
FA(10:5)	0	0	0	0	0	#DIV/0!	#DIV/0!	#DIV/0!
FA(35:0)	0	0	0	0	0	#DIV/0!	#DIV/0!	#DIV/0!
[TG(53:10),TG(52:3)]_C20:0	0	0	0	0	0	#DIV/0!	#DIV/0!	#DIV/0!
PS(25:0)	0	0	0	0	0	#DIV/0!	#DIV/0!	#DIV/0!
FA(28:0)	0	0	0	0	0	#DIV/0!	#DIV/0!	#DIV/0!
FA(32:0)	0	0	0	0	0	#DIV/0!	#DIV/0!	#DIV/0!
[TG(58:9)]_C20:4	0	0	0	0	0	#DIV/0!	#DIV/0!	#DIV/0!
[TG(54:6)]_C16:1	0	0	0	0	0	#DIV/0!	#DIV/0!	#DIV/0!
PC(44:4)	0	0	0	0	0	#DIV/0!	#DIV/0!	#DIV/0!
FA(18:2)	0	0	0	0	0	#DIV/0!	#DIV/0!	#DIV/0!
CAR(10:2)	0	0	0	0	0	#DIV/0!	#DIV/0!	#DIV/0!
FA(28:6)	0	0	0	0	0	#DIV/0!	#DIV/0!	#DIV/0!
FA(21:2)	0	0	0	0	0	#DIV/0!	#DIV/0!	#DIV/0!
FA(30:2)	0	0	0	0	0	#DIV/0!	#DIV/0!	#DIV/0!
CAR(26:0)	0	0	0	0	0	#DIV/0!	#DIV/0!	#DIV/0!
DG(37:7)_C18:1	0	0	0	0	0	#DIV/0!	#DIV/0!	#DIV/0!
DG(30:2)_C18:1	0	0	0	0	0	#DIV/0!	#DIV/0!	#DIV/0!
FA(24:0)	0	0	0	0	0	#DIV/0!	#DIV/0!	#DIV/0!
FA(24:2)	0	0	0	0	0	#DIV/0!	#DIV/0!	#DIV/0!
FA(20:6)	0	0	0	0	0	#DIV/0!	#DIV/0!	#DIV/0!
FA(24:1)	0	0	0	0	0	#DIV/0!	#DIV/0!	#DIV/0!
PG(24:0); PG(24:0)	0	0	0	0	0	#DIV/0!	#DIV/0!	#DIV/0!
[TG(40:0)]_C18:0	0	0	0	0	0	#DIV/0!	#DIV/0!	#DIV/0!
LPE(20:4)	0	0	0	0	0	#DIV/0!	#DIV/0!	#DIV/0!
PC(26:1)	0	0	0	0	0	#DIV/0!	#DIV/0!	#DIV/0!
CE(24:1)K	0	0	0	0	0	#DIV/0!	#DIV/0!	#DIV/0!
[TG(50:4)]_C20:4	0	0	0	0	0	#DIV/0!	#DIV/0!	#DIV/0!
PG(32:0),PG(O-33:0); PG(32:0)	0	0	0	0	0	#DIV/0!	#DIV/0!	#DIV/0!
[TG(44:2)]_C18:1	0	0	0	0	0	#DIV/0!	#DIV/0!	#DIV/0!

[TG(59:9),TG(58:2)]_C18:2	0	0	0	0	0	#DIV/0!	#DIV/0!	#DIV/0!
PI(36:4)	0	0	0	0	0	#DIV/0!	#DIV/0!	#DIV/0!
[TG(52:4)]_C18:0	0	0	0	0	0	#DIV/0!	#DIV/0!	#DIV/0!
[TG(53:9),TG(52:2)]_C14:0	0	0	0	0	0	#DIV/0!	#DIV/0!	#DIV/0!
PC(44:8),PC(43:1)	0	0	0	0	0	#DIV/0!	#DIV/0!	#DIV/0!
[TG(38:1)]_C16:0	0	0	0	0	0	#DIV/0!	#DIV/0!	#DIV/0!
DG(40:7),DG(39:0)_C18:0	0	0	0	0	0	#DIV/0!	#DIV/0!	#DIV/0!
LPG(15:0),LPG(O-16:0); LPG(15	0	0	0	0	0	#DIV/0!	#DIV/0!	#DIV/0!
[TG(46:6)]_C16:0	0	0	0	0	0	#DIV/0!	#DIV/0!	#DIV/0!
FA(22:5)	0	0	0	0	0	#DIV/0!	#DIV/0!	#DIV/0!
[TG(51:8)]_C22:5	0	0	0	0	0	#DIV/0!	#DIV/0!	#DIV/0!
FA(32:5)	0	0	0	0	0	#DIV/0!	#DIV/0!	#DIV/0!
FA(30:4)	0	0	0	0	0	#DIV/0!	#DIV/0!	#DIV/0!
FA(23:1)	0	0	0	0	0	#DIV/0!	#DIV/0!	#DIV/0!
FA(20:2)	0	0	0	0	0	#DIV/0!	#DIV/0!	#DIV/0!
CAR(22:5)	0	0	0	0	0	#DIV/0!	#DIV/0!	#DIV/0!
[TG(62:14),TG(61:7),TG(60:0)]_	0	0	0	0	0	#DIV/0!	#DIV/0!	#DIV/0!
CAR(20:2)	0	0	0	0	0	#DIV/0!	#DIV/0!	#DIV/0!
[TG(37:0)]_C16:0	0	0	0	0	0	#DIV/0!	#DIV/0!	#DIV/0!
DG(33:1),DG(O-34:1)_C16:1	0	0	0	0	0	#DIV/0!	#DIV/0!	#DIV/0!
DG(33:1),DG(O-34:1)_C18:1	0	0	0	0	0	#DIV/0!	#DIV/0!	#DIV/0!
DG(36:7)_C18:1	0	0	0	0	0	#DIV/0!	#DIV/0!	#DIV/0!
FA(19:1)	0	0	0	0	0	#DIV/0!	#DIV/0!	#DIV/0!
LPI(16:0)	0	0	0	0	0	#DIV/0!	#DIV/0!	#DIV/0!
[TG(41:0)]_C16:0	0	0	0	0	0	#DIV/0!	#DIV/0!	#DIV/0!
LPE(18:1)	0	0	0	0	0	#DIV/0!	#DIV/0!	#DIV/0!
FA(25:0)	0	0	0	0	0	#DIV/0!	#DIV/0!	#DIV/0!
FA(6:2)	0	0	0	0	0	#DIV/0!	#DIV/0!	#DIV/0!
FA(33:0)	0	0	0	0	0	#DIV/0!	#DIV/0!	#DIV/0!
CAR(18:0)	0	0	0	0	0	#DIV/0!	#DIV/0!	#DIV/0!
LPG(16:0); LPG(16:0)	0	0	0	0	0	#DIV/0!	#DIV/0!	#DIV/0!
FA(21:5)	0	0	0	0	0	#DIV/0!	#DIV/0!	#DIV/0!
FA(22:6)	0	0	0	0	0	#DIV/0!	#DIV/0!	#DIV/0!
FA(26:4)	0	0	0	0	0	#DIV/0!	#DIV/0!	#DIV/0!
FA(19:6)	0	0	0	0	0	#DIV/0!	#DIV/0!	#DIV/0!
[TG(57:8)]_C18:2	0	0	0	0	0	#DIV/0!	#DIV/0!	#DIV/0!
FA(20:1)	0	0	0	0	0	#DIV/0!	#DIV/0!	#DIV/0!
FA(18:1)	0	0	0	0	0	#DIV/0!	#DIV/0!	#DIV/0!
CAR(22:0)	0	0	0	0	0	#DIV/0!	#DIV/0!	#DIV/0!
CAR(5:1)	0	0	0	0	0	#DIV/0!	#DIV/0!	#DIV/0!
FA(22:2)	0	0	0	0	0	#DIV/0!	#DIV/0!	#DIV/0!
CAR(10:3)	0	0	0	0	0	#DIV/0!	#DIV/0!	#DIV/0!
FA(36:6)	0	0	0	0	0	#DIV/0!	#DIV/0!	#DIV/0!
[TG(43:1)]_C16:1	0	0	0	0	0	#DIV/0!	#DIV/0!	#DIV/0!
FA(16:6)	0	0	0	0	0	#DIV/0!	#DIV/0!	#DIV/0!
[TG(58:14),TG(57:7),TG(56:0)]_	0	0	0	0	0	#DIV/0!	#DIV/0!	#DIV/0!
FA(27:3)	0	0	0	0	0	#DIV/0!	#DIV/0!	#DIV/0!
FA(36:4)	0	0	0	0	0	#DIV/0!	#DIV/0!	#DIV/0!
FA(19:5)	0	0	0	0	0	#DIV/0!	#DIV/0!	#DIV/0!
FA(24:5)	0	0	0	0	0	#DIV/0!	#DIV/0!	#DIV/0!
CAR(14:0)	0	0	0	0	0	#DIV/0!	#DIV/0!	#DIV/0!
[TG(45:2)]_C16:1	0	0	0	0	0	#DIV/0!	#DIV/0!	#DIV/0!
DG(33:3)_C16:0	0	0	0	0	0	#DIV/0!	#DIV/0!	#DIV/0!
FA(9:0)	0	0	0	0	0	#DIV/0!	#DIV/0!	#DIV/0!
FA(14:6)	0	0	0	0	0	#DIV/0!	#DIV/0!	#DIV/0!
FA(18:6)	0	0	0	0	0	#DIV/0!	#DIV/0!	#DIV/0!
FA(18:5)	0	0	0	0	0	#DIV/0!	#DIV/0!	#DIV/0!
FA(20:3)	0	0	0	0	0	#DIV/0!	#DIV/0!	#DIV/0!
DG(34:3)_C18:1	0	0	0	0	0	#DIV/0!	#DIV/0!	#DIV/0!
PE(32:1),PE(O-33:1),PE(P-33:0)	0	0	0	0	0	#DIV/0!	#DIV/0!	#DIV/0!
LPE(22:6)	0	0	0	0	0	#DIV/0!	#DIV/0!	#DIV/0!
CE(12:0) NH4	0	0	0	0	0	#DIV/0!	#DIV/0!	#DIV/0!
DG(44:9),DG(43:2)_C18:1	0	0	0	0	0	#DIV/0!	#DIV/0!	#DIV/0!
[TG(58:8)]_C20:4	0	0	0	0	0	#DIV/0!	#DIV/0!	#DIV/0!
CE(18:2)H	0	0	0	0	0	#DIV/0!	#DIV/0!	#DIV/0!
PE(40:5)	0	0	0	0	0	#DIV/0!	#DIV/0!	#DIV/0!

DG(40:7),DG(39:0)_C16:0	0	0	0	0	0	#DIV/0!	#DIV/0!	#DIV/0!
PE(35:4),PE(O-36:4),PE(P-36:3)	0	0	0	0	0	#DIV/0!	#DIV/0!	#DIV/0!
DG(33:2)_C18:1	0	0	0	0	0	#DIV/0!	#DIV/0!	#DIV/0!
CAR(17:0)	0	0	0	0	0	#DIV/0!	#DIV/0!	#DIV/0!
[TG(44:3)]_C20:0	0	0	0	0	0	#DIV/0!	#DIV/0!	#DIV/0!
FA(14:5)	0	0	0	0	0	#DIV/0!	#DIV/0!	#DIV/0!
LPE(18:2),LPE(P-19:1)	0	0	0	0	0	#DIV/0!	#DIV/0!	#DIV/0!
[TG(40:1)]_C18:1	0	0	0	0	0	#DIV/0!	#DIV/0!	#DIV/0!
PC(43:2)	0	0	0	0	0	#DIV/0!	#DIV/0!	#DIV/0!
DG(28:2)_C18:1	0	0	0	0	0	#DIV/0!	#DIV/0!	#DIV/0!
DG(28:1)_C18:1	0	0	0	0	0	#DIV/0!	#DIV/0!	#DIV/0!
DG(31:1)_C18:1	0	0	0	0	0	#DIV/0!	#DIV/0!	#DIV/0!
CAR(22:2)	0	0	0	0	0	#DIV/0!	#DIV/0!	#DIV/0!
[TG(43:1)]_C18:1	0	0	0	0	0	#DIV/0!	#DIV/0!	#DIV/0!
[TG(38:1)]_C14:0	0	0	0	0	0	#DIV/0!	#DIV/0!	#DIV/0!
FA(40:6)	0	0	0	0	0	#DIV/0!	#DIV/0!	#DIV/0!
FA(3:0)	0	0	0	0	0	#DIV/0!	#DIV/0!	#DIV/0!
[TG(43:1)]_C16:0	0	0	0	0	0	#DIV/0!	#DIV/0!	#DIV/0!
FA(27:0)	0	0	0	0	0	#DIV/0!	#DIV/0!	#DIV/0!
DG(44:7),DG(43:0)_C18:0	0	0	0	0	0	#DIV/0!	#DIV/0!	#DIV/0!
FA(28:1)	0	0	0	0	0	#DIV/0!	#DIV/0!	#DIV/0!
FA(26:0)	0	0	0	0	0	#DIV/0!	#DIV/0!	#DIV/0!
DG(38:6),DG(dO-40:6)_C16:0	0	0	0	0	0	#DIV/0!	#DIV/0!	#DIV/0!
FA(34:1)	0	0	0	0	0	#DIV/0!	#DIV/0!	#DIV/0!
[TG(55:7),TG(54:0)]_C14:0	0	0	0	0	0	#DIV/0!	#DIV/0!	#DIV/0!
[TG(59:9),TG(58:2)]_C18:1	0	0	0	0	0	#DIV/0!	#DIV/0!	#DIV/0!
[TG(44:1)]_C16:1	0	0	0	0	0	#DIV/0!	#DIV/0!	#DIV/0!
[TG(48:8),TG(47:1)]_C14:0	0	0	0	0	0	#DIV/0!	#DIV/0!	#DIV/0!
[TG(60:15),TG(59:8),TG(58:1)]_	0	0	0	0	0	#DIV/0!	#DIV/0!	#DIV/0!
[TG(61:10),TG(60:3)]_C18:1	0	0	0	0	0	#DIV/0!	#DIV/0!	#DIV/0!
[TG(52:4)]_C14:0	0	0	0	0	0	#DIV/0!	#DIV/0!	#DIV/0!
[TG(47:2)]_C18:2	0	0	0	0	0	#DIV/0!	#DIV/0!	#DIV/0!
[TG(50:5)]_C20:4	0	0	0	0	0	#DIV/0!	#DIV/0!	#DIV/0!
[TG(42:1)]_C16:0	0	0	0	0	0	#DIV/0!	#DIV/0!	#DIV/0!
[TG(59:11),TG(58:4)]_C18:2	0	0	0	0	0	#DIV/0!	#DIV/0!	#DIV/0!
[TG(50:7)]_C18:1	0	0	0	0	0	#DIV/0!	#DIV/0!	#DIV/0!
[TG(45:0)]_C14:0	0	0	0	0	0	#DIV/0!	#DIV/0!	#DIV/0!
LPC(15:1),LPC(O-16:1),LPC(P-	0	0	0	0	0	#DIV/0!	#DIV/0!	#DIV/0!
[TG(58:10)]_C20:4	0	0	0	0	0	#DIV/0!	#DIV/0!	#DIV/0!
[TG(50:9),TG(49:2)]_C14:0	0	0	0	0	0	#DIV/0!	#DIV/0!	#DIV/0!
PI(36:3),PI(P-37:2)	0	0	0	0	0	#DIV/0!	#DIV/0!	#DIV/0!
[TG(47:6)]_C14:0	0	0	0	0	0	#DIV/0!	#DIV/0!	#DIV/0!
[TG(47:2)]_C16:0	0	0	0	0	0	#DIV/0!	#DIV/0!	#DIV/0!
[TG(59:9),TG(58:2)]_C16:0	0	0	0	0	0	#DIV/0!	#DIV/0!	#DIV/0!
[TG(43:0)]_C16:0	0	0	0	0	0	#DIV/0!	#DIV/0!	#DIV/0!
[TG(46:3)]_C14:0	0	0	0	0	0	#DIV/0!	#DIV/0!	#DIV/0!
[TG(51:8),TG(50:1)]_C20:0	0	0	0	0	0	#DIV/0!	#DIV/0!	#DIV/0!
[TG(42:2)]_C18:2	0	0	0	0	0	#DIV/0!	#DIV/0!	#DIV/0!
[TG(42:1)]_C14:0	0	0	0	0	0	#DIV/0!	#DIV/0!	#DIV/0!
[TG(45:1)]_C16:0	0	0	0	0	0	#DIV/0!	#DIV/0!	#DIV/0!
[TG(44:2)]_C16:1	0	0	0	0	0	#DIV/0!	#DIV/0!	#DIV/0!
[TG(44:2)]_C14:0	0	0	0	0	0	#DIV/0!	#DIV/0!	#DIV/0!
FA(36:5)	0	0	0	0	0	#DIV/0!	#DIV/0!	#DIV/0!
PG(O-37:2),PG(P-37:1); PG(O-3	0	0	0	0	0	#DIV/0!	#DIV/0!	#DIV/0!
[TG(47:2)]_C14:0	0	0	0	0	0	#DIV/0!	#DIV/0!	#DIV/0!
[TG(49:3)]_C18:1	0	0	0	0	0	#DIV/0!	#DIV/0!	#DIV/0!
[TG(45:1)]_C18:1	0	0	0	0	0	#DIV/0!	#DIV/0!	#DIV/0!
[TG(47:2)]_C18:1	0	0	0	0	0	#DIV/0!	#DIV/0!	#DIV/0!
[TG(52:6)]_C14:0	0	0	0	0	0	#DIV/0!	#DIV/0!	#DIV/0!
[TG(54:7)]_C16:1	0	0	0	0	0	#DIV/0!	#DIV/0!	#DIV/0!
LPC(17:1),LPC(O-18:1),LPC(P-	0	0	0	0	0	#DIV/0!	#DIV/0!	#DIV/0!
[TG(60:15),TG(59:8),TG(58:1)]_	0	0	0	0	0	#DIV/0!	#DIV/0!	#DIV/0!
CAR(16:0)	0	0	0	0	0	#DIV/0!	#DIV/0!	#DIV/0!
PG(36:3),PG(P-37:2); PG(36:3),	0	0	0	0	0	#DIV/0!	#DIV/0!	#DIV/0!
PC(50:0)	0	0	0	0	0	#DIV/0!	#DIV/0!	#DIV/0!
[TG(40:0)]_C14:0	0	0	0	0	0	#DIV/0!	#DIV/0!	#DIV/0!

[TG(47:6)]_C18:0	0	0	0	0	0	#DIV/0!	#DIV/0!	#DIV/0!
DG(44:1)_C18:1	0	0	0	0	0	#DIV/0!	#DIV/0!	#DIV/0!
FA(31:0)	0	0	0	0	0	#DIV/0!	#DIV/0!	#DIV/0!
PI(34:1),PI(O-35:1),PI(P-35:0)	0	0	0	0	0	#DIV/0!	#DIV/0!	#DIV/0!
[TG(49:3)]_C16:1	0	0	0	0	0	#DIV/0!	#DIV/0!	#DIV/0!
DG(38:8),DG(dO-40:8),DG(37:1	0	0	0	0	0	#DIV/0!	#DIV/0!	#DIV/0!
[TG(38:0)]_C16:0	0	0	0	0	0	#DIV/0!	#DIV/0!	#DIV/0!
[TG(45:4)]_C18:1	0	0	0	0	0	#DIV/0!	#DIV/0!	#DIV/0!
[TG(50:9)]_C22:6	0	0	0	0	0	#DIV/0!	#DIV/0!	#DIV/0!
[TG(40:1)]_C16:0	0	0	0	0	0	#DIV/0!	#DIV/0!	#DIV/0!
[TG(60:14),TG(59:7),TG(58:0)]_	0	0	0	0	0	#DIV/0!	#DIV/0!	#DIV/0!
PG(30:0),PG(O-31:0); PG(30:0)	0	0	0	0	0	#DIV/0!	#DIV/0!	#DIV/0!
[TG(54:12),TG(53:5)]_C22:5	0	0	0	0	0	#DIV/0!	#DIV/0!	#DIV/0!
DG(41:7),DG(40:0)_C16:0	0	0	0	0	0	#DIV/0!	#DIV/0!	#DIV/0!
[TG(44:4)]_C20:0	0	0	0	0	0	#DIV/0!	#DIV/0!	#DIV/0!
[TG(49:3)]_C20:0	0	0	0	0	0	#DIV/0!	#DIV/0!	#DIV/0!
[TG(45:4)]_C16:1	0	0	0	0	0	#DIV/0!	#DIV/0!	#DIV/0!
FA(25:4)	0	0	0	0	0	#DIV/0!	#DIV/0!	#DIV/0!
FA(30:3)	0	0	0	0	0	#DIV/0!	#DIV/0!	#DIV/0!
CE(50:3;O2)H	0	0	0	0	0	#DIV/0!	#DIV/0!	#DIV/0!
PG(26:0); PG(26:0)	0	0	0	0	0	#DIV/0!	#DIV/0!	#DIV/0!
PC(44:6)	0	0	0	0	0	#DIV/0!	#DIV/0!	#DIV/0!
[TG(40:1)]_C14:0	0	0	0	0	0	#DIV/0!	#DIV/0!	#DIV/0!
[TG(42:1)]_C16:1	0	0	0	0	0	#DIV/0!	#DIV/0!	#DIV/0!
[TG(45:1)]_C14:0	0	0	0	0	0	#DIV/0!	#DIV/0!	#DIV/0!
[TG(55:7)]_C22:6	0	0	0	0	0	#DIV/0!	#DIV/0!	#DIV/0!
CE(46:3;O2) NH4	0	0	0	0	0	#DIV/0!	#DIV/0!	#DIV/0!
FA(12:6)	0	0	0	0	0	#DIV/0!	#DIV/0!	#DIV/0!
FA(17:1)	0	0	0	0	0	#DIV/0!	#DIV/0!	#DIV/0!
FA(24:3)	0	0	0	0	0	#DIV/0!	#DIV/0!	#DIV/0!
FA(26:3)	0	0	0	0	0	#DIV/0!	#DIV/0!	#DIV/0!
FA(38:4)	0	0	0	0	0	#DIV/0!	#DIV/0!	#DIV/0!
FA(5:1)	0	0	0	0	0	#DIV/0!	#DIV/0!	#DIV/0!
DG(29:2)_C16:0	0	0	0	0	0	#DIV/0!	#DIV/0!	#DIV/0!
PG(28:0),PG(O-29:0); PG(28:0)	0	0	0	0	0	#DIV/0!	#DIV/0!	#DIV/0!
FA(15:5)	0	0	0	0	0	#DIV/0!	#DIV/0!	#DIV/0!
FA(17:6)	0	0	0	0	0	#DIV/0!	#DIV/0!	#DIV/0!
FA(26:5)	0	0	0	0	0	#DIV/0!	#DIV/0!	#DIV/0!
[TG(57:8),TG(56:1)]_C16:1	0	0	0	0	0	#DIV/0!	#DIV/0!	#DIV/0!
LPS(O-20:0)	0	0	0	0	0	#DIV/0!	#DIV/0!	#DIV/0!
LPE(12:0)	0	0	0	0	0	#DIV/0!	#DIV/0!	#DIV/0!
[TG(48:5)]_C22:5	0	0	0	0	0	#DIV/0!	#DIV/0!	#DIV/0!
[TG(50:5)]_C22:5	0	0	0	0	0	#DIV/0!	#DIV/0!	#DIV/0!
[TG(59:12),TG(58:5)]_C22:5	0	0	0	0	0	#DIV/0!	#DIV/0!	#DIV/0!
DG(34:5)_C16:1	0	0	0	0	0	#DIV/0!	#DIV/0!	#DIV/0!
DG(42:0)_C16:0	0	0	0	0	0	#DIV/0!	#DIV/0!	#DIV/0!
FA(27:2)	0	0	0	0	0	#DIV/0!	#DIV/0!	#DIV/0!
[TG(37:0)]_C20:0	0	0	0	0	0	#DIV/0!	#DIV/0!	#DIV/0!
PE(40:4)	0	0	0	0	0	#DIV/0!	#DIV/0!	#DIV/0!
DG(30:2)_C18:2	0	0	0	0	0	#DIV/0!	#DIV/0!	#DIV/0!
CE(12:0)Na	0	0	0	0	0	#DIV/0!	#DIV/0!	#DIV/0!
CE(22:3)Na	0	0	0	0	0	#DIV/0!	#DIV/0!	#DIV/0!
DG(33:3)_C18:2	0	0	0	0	0	#DIV/0!	#DIV/0!	#DIV/0!
DG(44:0)_C16:0	0	0	0	0	0	#DIV/0!	#DIV/0!	#DIV/0!
FA(14:1)	0	0	0	0	0	#DIV/0!	#DIV/0!	#DIV/0!
DG(38:7),DG(37:0)_C18:0	0	0	0	0	0	#DIV/0!	#DIV/0!	#DIV/0!
PC(18:0),LPC(19:0),PC(O-19:0)	0	0	0	0	0	#DIV/0!	#DIV/0!	#DIV/0!
PE(39:6),PE(O-40:6),PE(P-40:5)	0	0	0	0	0	#DIV/0!	#DIV/0!	#DIV/0!
[TG(45:0)]_C18:0	0	0	0	0	0	#DIV/0!	#DIV/0!	#DIV/0!
[TG(51:4)]_C16:1	0	0	0	0	0	#DIV/0!	#DIV/0!	#DIV/0!
[TG(52:9),TG(51:2)]_C14:0	0	0	0	0	0	#DIV/0!	#DIV/0!	#DIV/0!
DG(26:0)_C16:0	0	0	0	0	0	#DIV/0!	#DIV/0!	#DIV/0!
CE(16:3) NH4	0	0	0	0	0	#DIV/0!	#DIV/0!	#DIV/0!
CE(22:5)K	0	0	0	0	0	#DIV/0!	#DIV/0!	#DIV/0!
SM(d16:0/14:0)	0	0	0	0	0	#DIV/0!	#DIV/0!	#DIV/0!
CE(20:3)K	0	0	0	0	0	#DIV/0!	#DIV/0!	#DIV/0!

CE(22:0) NH4	0	0	0	0	0	#DIV/0!	#DIV/0!	#DIV/0!
DG(33:1),DG(O-34:1)_C16:0	0	0	0	0	0	#DIV/0!	#DIV/0!	#DIV/0!
DG(39:7),DG(38:0),DG(dO-40:(	0	0	0	0	0	#DIV/0!	#DIV/0!	#DIV/0!
[TG(44:4)]_C18:1	0	0	0	0	0	#DIV/0!	#DIV/0!	#DIV/0!
[TG(45:3)]_C20:0	0	0	0	0	0	#DIV/0!	#DIV/0!	#DIV/0!
[TG(51:9)]_C22:6	0	0	0	0	0	#DIV/0!	#DIV/0!	#DIV/0!
DG(32:2)_C18:0	0	0	0	0	0	#DIV/0!	#DIV/0!	#DIV/0!
FA(29:2)	0	0	0	0	0	#DIV/0!	#DIV/0!	#DIV/0!
[TG(39:0)]_C18:0	0	0	0	0	0	#DIV/0!	#DIV/0!	#DIV/0!
[TG(44:3)]_C16:1	0	0	0	0	0	#DIV/0!	#DIV/0!	#DIV/0!
DG(36:8),DG(35:1)_C16:0	0	0	0	0	0	#DIV/0!	#DIV/0!	#DIV/0!
DG(38:9),DG(dO-40:9),DG(37:2	0	0	0	0	0	#DIV/0!	#DIV/0!	#DIV/0!
FA(16:1)	0	0	0	0	0	#DIV/0!	#DIV/0!	#DIV/0!
CAR(10:1)	0	0	0	0	0	#DIV/0!	#DIV/0!	#DIV/0!
CAR(20:4)	0	0	0	0	0	#DIV/0!	#DIV/0!	#DIV/0!
[TG(44:5)]_C18:2	0	0	0	0	0	#DIV/0!	#DIV/0!	#DIV/0!
FA(11:0)	0	0	0	0	0	#DIV/0!	#DIV/0!	#DIV/0!
FA(29:0)	0	0	0	0	0	#DIV/0!	#DIV/0!	#DIV/0!
CAR	0	0	0	0	0	#DIV/0!	#DIV/0!	#DIV/0!
CAR(5:0)	0	0	0	0	0	#DIV/0!	#DIV/0!	#DIV/0!
CAR(7:0)	0	0	0	0	0	#DIV/0!	#DIV/0!	#DIV/0!
CAR(8:1)	0	0	0	0	0	#DIV/0!	#DIV/0!	#DIV/0!
CAR(8:0)	0	0	0	0	0	#DIV/0!	#DIV/0!	#DIV/0!
CAR(9:0)	0	0	0	0	0	#DIV/0!	#DIV/0!	#DIV/0!
CAR(10:0)	0	0	0	0	0	#DIV/0!	#DIV/0!	#DIV/0!
CAR(11:0)	0	0	0	0	0	#DIV/0!	#DIV/0!	#DIV/0!
CAR(12:0)	0	0	0	0	0	#DIV/0!	#DIV/0!	#DIV/0!
CAR(16:2)	0	0	0	0	0	#DIV/0!	#DIV/0!	#DIV/0!
CAR(16:1)	0	0	0	0	0	#DIV/0!	#DIV/0!	#DIV/0!
CAR(18:4)	0	0	0	0	0	#DIV/0!	#DIV/0!	#DIV/0!
CAR(18:1)	0	0	0	0	0	#DIV/0!	#DIV/0!	#DIV/0!
LPC(12:0)	0	0	0	0	0	#DIV/0!	#DIV/0!	#DIV/0!
LPG(12:0); LPG(12:0)	0	0	0	0	0	#DIV/0!	#DIV/0!	#DIV/0!
CAR(22:6)	0	0	0	0	0	#DIV/0!	#DIV/0!	#DIV/0!
LPG(14:0); LPG(14:0)	0	0	0	0	0	#DIV/0!	#DIV/0!	#DIV/0!
LPE(18:3)	0	0	0	0	0	#DIV/0!	#DIV/0!	#DIV/0!
CAR(22:4)	0	0	0	0	0	#DIV/0!	#DIV/0!	#DIV/0!
LPC(16:1),LPC(P-17:0)	0	0	0	0	0	#DIV/0!	#DIV/0!	#DIV/0!
LPE(20:5)	0	0	0	0	0	#DIV/0!	#DIV/0!	#DIV/0!
LPE(20:3)	0	0	0	0	0	#DIV/0!	#DIV/0!	#DIV/0!
LPC(18:3)	0	0	0	0	0	#DIV/0!	#DIV/0!	#DIV/0!
LPE(22:5)	0	0	0	0	0	#DIV/0!	#DIV/0!	#DIV/0!
LPG(18:1); LPG(18:1)	0	0	0	0	0	#DIV/0!	#DIV/0!	#DIV/0!
PC(18:1),LPC(19:1),PC(O-19:1)	0	0	0	0	0	#DIV/0!	#DIV/0!	#DIV/0!
LPC(20:5)	0	0	0	0	0	#DIV/0!	#DIV/0!	#DIV/0!
LPS(22:6)	0	0	0	0	0	#DIV/0!	#DIV/0!	#DIV/0!
LPG(22:6); LPG(22:6)	0	0	0	0	0	#DIV/0!	#DIV/0!	#DIV/0!
LPG(22:0); LPG(22:0)	0	0	0	0	0	#DIV/0!	#DIV/0!	#DIV/0!
LPI(19:1),LPI(P-20:0)	0	0	0	0	0	#DIV/0!	#DIV/0!	#DIV/0!
PG(26:1); PG(26:1)	0	0	0	0	0	#DIV/0!	#DIV/0!	#DIV/0!
SM(d18:0/13:0)	0	0	0	0	0	#DIV/0!	#DIV/0!	#DIV/0!
Cer(d18:1/25:0)	0	0	0	0	0	#DIV/0!	#DIV/0!	#DIV/0!
PG(28:1),PG(P-29:0); PG(28:1),	0	0	0	0	0	#DIV/0!	#DIV/0!	#DIV/0!
PG(29:0),PG(O-30:0); PG(29:0)	0	0	0	0	0	#DIV/0!	#DIV/0!	#DIV/0!
PG(30:1),PG(O-31:1),PG(P-31:(	0	0	0	0	0	#DIV/0!	#DIV/0!	#DIV/0!
PE(34:4)	0	0	0	0	0	#DIV/0!	#DIV/0!	#DIV/0!
PG(31:0),PG(O-32:0); PG(31:0)	0	0	0	0	0	#DIV/0!	#DIV/0!	#DIV/0!
PG(32:2),PG(O-33:2),PG(P-33:1	0	0	0	0	0	#DIV/0!	#DIV/0!	#DIV/0!
PC(33:5),PC(P-34:4)	0	0	0	0	0	#DIV/0!	#DIV/0!	#DIV/0!
PG(32:1),PG(O-33:1),PG(P-33:(	0	0	0	0	0	#DIV/0!	#DIV/0!	#DIV/0!
PC(33:4),PC(O-34:4),PC(O-34:4	0	0	0	0	0	#DIV/0!	#DIV/0!	#DIV/0!
PC(34:5)	0	0	0	0	0	#DIV/0!	#DIV/0!	#DIV/0!
PG(33:1),PG(O-34:1),PG(P-34:(	0	0	0	0	0	#DIV/0!	#DIV/0!	#DIV/0!
PC(34:4),PC(O-35:4)	0	0	0	0	0	#DIV/0!	#DIV/0!	#DIV/0!
PE(37:4),PE(O-38:4),PE(P-38:3)	0	0	0	0	0	#DIV/0!	#DIV/0!	#DIV/0!
PG(33:0),PG(O-34:0); PG(33:0)	0	0	0	0	0	#DIV/0!	#DIV/0!	#DIV/0!

PG(34:0),PG(O-35:0); PG(34:0)	0	0	0	0	0	#DIV/0!	#DIV/0!	#DIV/0!
PG(35:6),PG(P-36:5); PG(35:6),	0	0	0	0	0	#DIV/0!	#DIV/0!	#DIV/0!
PG(36:1),PG(O-37:1),PG(P-37:(	0	0	0	0	0	#DIV/0!	#DIV/0!	#DIV/0!
PG(37:7),PG(P-38:6),PG(36:0),I	0	0	0	0	0	#DIV/0!	#DIV/0!	#DIV/0!
PE(40:3)	0	0	0	0	0	#DIV/0!	#DIV/0!	#DIV/0!
PE(42:5)	0	0	0	0	0	#DIV/0!	#DIV/0!	#DIV/0!
PS(40:4)	0	0	0	0	0	#DIV/0!	#DIV/0!	#DIV/0!
PE(44:8),PE(43:1)	0	0	0	0	0	#DIV/0!	#DIV/0!	#DIV/0!
PE(44:7),PE(43:0)	0	0	0	0	0	#DIV/0!	#DIV/0!	#DIV/0!
PS(O-42:0)	0	0	0	0	0	#DIV/0!	#DIV/0!	#DIV/0!
PS(42:5)	0	0	0	0	0	#DIV/0!	#DIV/0!	#DIV/0!
PS(43:1)	0	0	0	0	0	#DIV/0!	#DIV/0!	#DIV/0!
PS(43:0)	0	0	0	0	0	#DIV/0!	#DIV/0!	#DIV/0!
PS(44:6)	0	0	0	0	0	#DIV/0!	#DIV/0!	#DIV/0!
PI(40:6)	0	0	0	0	0	#DIV/0!	#DIV/0!	#DIV/0!
PI(40:5)	0	0	0	0	0	#DIV/0!	#DIV/0!	#DIV/0!
[TG(63:13),TG(62:6)]_C22:6	0	0	0	0	0	#DIV/0!	#DIV/0!	#DIV/0!
[TG(38:1)]_C16:1	0	0	0	0	0	#DIV/0!	#DIV/0!	#DIV/0!
[TG(39:1)]_C18:1	0	0	0	0	0	#DIV/0!	#DIV/0!	#DIV/0!
[TG(39:1)]_C16:0	0	0	0	0	0	#DIV/0!	#DIV/0!	#DIV/0!
[TG(39:1)]_C16:1	0	0	0	0	0	#DIV/0!	#DIV/0!	#DIV/0!
[TG(39:1)]_C14:0	0	0	0	0	0	#DIV/0!	#DIV/0!	#DIV/0!
[TG(39:0)]_C14:0	0	0	0	0	0	#DIV/0!	#DIV/0!	#DIV/0!
[TG(40:2)]_C18:1	0	0	0	0	0	#DIV/0!	#DIV/0!	#DIV/0!
[TG(40:2)]_C16:1	0	0	0	0	0	#DIV/0!	#DIV/0!	#DIV/0!
[TG(40:1)]_C18:0	0	0	0	0	0	#DIV/0!	#DIV/0!	#DIV/0!
[TG(40:1)]_C16:1	0	0	0	0	0	#DIV/0!	#DIV/0!	#DIV/0!
[TG(41:2)]_C18:1	0	0	0	0	0	#DIV/0!	#DIV/0!	#DIV/0!
[TG(41:1)]_C18:1	0	0	0	0	0	#DIV/0!	#DIV/0!	#DIV/0!
[TG(41:1)]_C16:0	0	0	0	0	0	#DIV/0!	#DIV/0!	#DIV/0!
[TG(41:1)]_C16:1	0	0	0	0	0	#DIV/0!	#DIV/0!	#DIV/0!
[TG(41:1)]_C14:0	0	0	0	0	0	#DIV/0!	#DIV/0!	#DIV/0!
[TG(41:0)]_C18:0	0	0	0	0	0	#DIV/0!	#DIV/0!	#DIV/0!
[TG(41:0)]_C14:0	0	0	0	0	0	#DIV/0!	#DIV/0!	#DIV/0!
[TG(42:2)]_C16:1	0	0	0	0	0	#DIV/0!	#DIV/0!	#DIV/0!
[TG(42:2)]_C14:0	0	0	0	0	0	#DIV/0!	#DIV/0!	#DIV/0!
[TG(43:2)]_C16:1	0	0	0	0	0	#DIV/0!	#DIV/0!	#DIV/0!
[TG(43:1)]_C14:0	0	0	0	0	0	#DIV/0!	#DIV/0!	#DIV/0!
[TG(43:0)]_C18:0	0	0	0	0	0	#DIV/0!	#DIV/0!	#DIV/0!
[TG(43:0)]_C14:0	0	0	0	0	0	#DIV/0!	#DIV/0!	#DIV/0!
[TG(44:5)]_C22:5	0	0	0	0	0	#DIV/0!	#DIV/0!	#DIV/0!
[TG(44:4)]_C16:1	0	0	0	0	0	#DIV/0!	#DIV/0!	#DIV/0!
[TG(45:3)]_C16:1	0	0	0	0	0	#DIV/0!	#DIV/0!	#DIV/0!
[TG(45:2)]_C18:1	0	0	0	0	0	#DIV/0!	#DIV/0!	#DIV/0!
[TG(45:2)]_C18:2	0	0	0	0	0	#DIV/0!	#DIV/0!	#DIV/0!
[TG(45:2)]_C14:0	0	0	0	0	0	#DIV/0!	#DIV/0!	#DIV/0!
[TG(45:1)]_C18:0	0	0	0	0	0	#DIV/0!	#DIV/0!	#DIV/0!
[TG(46:6)]_C18:0	0	0	0	0	0	#DIV/0!	#DIV/0!	#DIV/0!
[TG(46:6)]_C14:0	0	0	0	0	0	#DIV/0!	#DIV/0!	#DIV/0!
[TG(47:3)]_C18:2	0	0	0	0	0	#DIV/0!	#DIV/0!	#DIV/0!
[TG(47:3)]_C16:1	0	0	0	0	0	#DIV/0!	#DIV/0!	#DIV/0!
[TG(48:8),TG(47:1)]_C18:0	0	0	0	0	0	#DIV/0!	#DIV/0!	#DIV/0!
[TG(48:7),TG(47:0)]_C18:0	0	0	0	0	0	#DIV/0!	#DIV/0!	#DIV/0!
[TG(48:7)]_C16:1	0	0	0	0	0	#DIV/0!	#DIV/0!	#DIV/0!
[TG(48:6)]_C14:0	0	0	0	0	0	#DIV/0!	#DIV/0!	#DIV/0!
[TG(48:4)]_C16:1	0	0	0	0	0	#DIV/0!	#DIV/0!	#DIV/0!
[TG(49:6)]_C14:0	0	0	0	0	0	#DIV/0!	#DIV/0!	#DIV/0!
[TG(49:4)]_C16:1	0	0	0	0	0	#DIV/0!	#DIV/0!	#DIV/0!
[TG(50:9),TG(49:2)]_C18:0	0	0	0	0	0	#DIV/0!	#DIV/0!	#DIV/0!
[TG(50:8),TG(49:1)]_C20:0	0	0	0	0	0	#DIV/0!	#DIV/0!	#DIV/0!
[TG(50:8),TG(49:1)]_C18:0	0	0	0	0	0	#DIV/0!	#DIV/0!	#DIV/0!
[TG(50:7)]_C16:1	0	0	0	0	0	#DIV/0!	#DIV/0!	#DIV/0!
[TG(50:7),TG(49:0)]_C14:0	0	0	0	0	0	#DIV/0!	#DIV/0!	#DIV/0!
[TG(50:6)]_C16:0	0	0	0	0	0	#DIV/0!	#DIV/0!	#DIV/0!
[TG(51:7)]_C22:5	0	0	0	0	0	#DIV/0!	#DIV/0!	#DIV/0!
[TG(52:8),TG(51:1)]_C16:1	0	0	0	0	0	#DIV/0!	#DIV/0!	#DIV/0!

[TG(52:8),TG(51:1)]_C14:0	0	0	0	0	0	#DIV/0!	#DIV/0!	#DIV/0!
[TG(52:7),TG(51:0)]_C14:0	0	0	0	0	0	#DIV/0!	#DIV/0!	#DIV/0!
[TG(53:8),TG(52:1)]_C14:0	0	0	0	0	0	#DIV/0!	#DIV/0!	#DIV/0!
[TG(53:7)]_C16:1	0	0	0	0	0	#DIV/0!	#DIV/0!	#DIV/0!
[TG(53:7),TG(52:0)]_C14:0	0	0	0	0	0	#DIV/0!	#DIV/0!	#DIV/0!
[TG(53:6)]_C16:0	0	0	0	0	0	#DIV/0!	#DIV/0!	#DIV/0!
[TG(54:11),TG(53:4)]_C16:1	0	0	0	0	0	#DIV/0!	#DIV/0!	#DIV/0!
[TG(54:10),TG(53:3)]_C16:1	0	0	0	0	0	#DIV/0!	#DIV/0!	#DIV/0!
[TG(54:9),TG(53:2)]_C16:1	0	0	0	0	0	#DIV/0!	#DIV/0!	#DIV/0!
[TG(54:8),TG(53:1)]_C16:1	0	0	0	0	0	#DIV/0!	#DIV/0!	#DIV/0!
[TG(54:7),TG(53:0)]_C14:0	0	0	0	0	0	#DIV/0!	#DIV/0!	#DIV/0!
[TG(55:9),TG(54:2)]_C16:1	0	0	0	0	0	#DIV/0!	#DIV/0!	#DIV/0!
[TG(55:8),TG(54:1)]_C16:1	0	0	0	0	0	#DIV/0!	#DIV/0!	#DIV/0!
[TG(56:10),TG(55:3)]_C16:1	0	0	0	0	0	#DIV/0!	#DIV/0!	#DIV/0!
[TG(56:9),TG(55:2)]_C16:1	0	0	0	0	0	#DIV/0!	#DIV/0!	#DIV/0!
[TG(56:8),TG(55:1)]_C16:1	0	0	0	0	0	#DIV/0!	#DIV/0!	#DIV/0!
[TG(56:8),TG(55:1)]_C14:0	0	0	0	0	0	#DIV/0!	#DIV/0!	#DIV/0!
[TG(56:7)]_C16:1	0	0	0	0	0	#DIV/0!	#DIV/0!	#DIV/0!
[TG(56:7),TG(55:0)]_C14:0	0	0	0	0	0	#DIV/0!	#DIV/0!	#DIV/0!
[TG(57:12),TG(56:5)]_C16:1	0	0	0	0	0	#DIV/0!	#DIV/0!	#DIV/0!
[TG(57:10),TG(56:3)]_C16:1	0	0	0	0	0	#DIV/0!	#DIV/0!	#DIV/0!
[TG(57:9),TG(56:2)]_C16:1	0	0	0	0	0	#DIV/0!	#DIV/0!	#DIV/0!
[TG(57:8),TG(56:1)]_C14:0	0	0	0	0	0	#DIV/0!	#DIV/0!	#DIV/0!
[TG(58:14),TG(57:7),TG(56:0)]_	0	0	0	0	0	#DIV/0!	#DIV/0!	#DIV/0!
[TG(58:14),TG(57:7)]_C16:1	0	0	0	0	0	#DIV/0!	#DIV/0!	#DIV/0!
[TG(58:14),TG(57:7),TG(56:0)]_	0	0	0	0	0	#DIV/0!	#DIV/0!	#DIV/0!
[TG(58:9),TG(57:2)]_C16:1	0	0	0	0	0	#DIV/0!	#DIV/0!	#DIV/0!
[TG(58:8),TG(57:1)]_C16:0	0	0	0	0	0	#DIV/0!	#DIV/0!	#DIV/0!
[TG(58:8),TG(57:1)]_C16:1	0	0	0	0	0	#DIV/0!	#DIV/0!	#DIV/0!
[TG(58:8),TG(57:1)]_C14:0	0	0	0	0	0	#DIV/0!	#DIV/0!	#DIV/0!
[TG(58:7),TG(57:0)]_C16:0	0	0	0	0	0	#DIV/0!	#DIV/0!	#DIV/0!
[TG(59:10),TG(58:3)]_C16:1	0	0	0	0	0	#DIV/0!	#DIV/0!	#DIV/0!
[TG(59:9),TG(58:2)]_C16:1	0	0	0	0	0	#DIV/0!	#DIV/0!	#DIV/0!
[TG(60:15),TG(59:8),TG(58:1)]_	0	0	0	0	0	#DIV/0!	#DIV/0!	#DIV/0!
[TG(60:15),TG(59:8),TG(58:1)]_	0	0	0	0	0	#DIV/0!	#DIV/0!	#DIV/0!
[TG(60:15),TG(59:8),TG(58:1)]_	0	0	0	0	0	#DIV/0!	#DIV/0!	#DIV/0!
[TG(60:14),TG(59:7),TG(58:0)]_	0	0	0	0	0	#DIV/0!	#DIV/0!	#DIV/0!
[TG(60:12),TG(59:5)]_C18:2	0	0	0	0	0	#DIV/0!	#DIV/0!	#DIV/0!
[TG(60:9),TG(59:2)]_C18:1	0	0	0	0	0	#DIV/0!	#DIV/0!	#DIV/0!
[TG(60:9),TG(59:2)]_C16:1	0	0	0	0	0	#DIV/0!	#DIV/0!	#DIV/0!
[TG(60:8),TG(59:1)]_C18:1	0	0	0	0	0	#DIV/0!	#DIV/0!	#DIV/0!
[TG(60:8),TG(59:1)]_C16:0	0	0	0	0	0	#DIV/0!	#DIV/0!	#DIV/0!
[TG(60:8),TG(59:1)]_C16:1	0	0	0	0	0	#DIV/0!	#DIV/0!	#DIV/0!
[TG(61:14),TG(60:7),TG(59:0)]_	0	0	0	0	0	#DIV/0!	#DIV/0!	#DIV/0!
[TG(61:13),TG(60:6)]_C20:0	0	0	0	0	0	#DIV/0!	#DIV/0!	#DIV/0!
[TG(61:11),TG(60:4)]_C20:4	0	0	0	0	0	#DIV/0!	#DIV/0!	#DIV/0!
[TG(61:11),TG(60:4)]_C18:0	0	0	0	0	0	#DIV/0!	#DIV/0!	#DIV/0!
[TG(61:10),TG(60:3)]_C16:1	0	0	0	0	0	#DIV/0!	#DIV/0!	#DIV/0!
[TG(62:15),TG(61:8),TG(60:1)]_	0	0	0	0	0	#DIV/0!	#DIV/0!	#DIV/0!
[TG(62:15),TG(61:8),TG(60:1)]_	0	0	0	0	0	#DIV/0!	#DIV/0!	#DIV/0!
DG(26:0)_C18:0	0	0	0	0	0	#DIV/0!	#DIV/0!	#DIV/0!
DG(28:2)_C18:2	0	0	0	0	0	#DIV/0!	#DIV/0!	#DIV/0!
DG(29:2)_C18:1	0	0	0	0	0	#DIV/0!	#DIV/0!	#DIV/0!
DG(29:1)_C18:1	0	0	0	0	0	#DIV/0!	#DIV/0!	#DIV/0!
DG(29:1)_C16:0	0	0	0	0	0	#DIV/0!	#DIV/0!	#DIV/0!
DG(31:2),DG(P-14:0/18:1)_C18	0	0	0	0	0	#DIV/0!	#DIV/0!	#DIV/0!
CE(12:0)H	0	0	0	0	0	#DIV/0!	#DIV/0!	#DIV/0!
DG(31:1)_C16:1	0	0	0	0	0	#DIV/0!	#DIV/0!	#DIV/0!
DG(31:0)_C16:0	0	0	0	0	0	#DIV/0!	#DIV/0!	#DIV/0!
DG(32:2)_C16:1	0	0	0	0	0	#DIV/0!	#DIV/0!	#DIV/0!
DG(33:5)_C18:1	0	0	0	0	0	#DIV/0!	#DIV/0!	#DIV/0!
DG(33:2)_C18:2	0	0	0	0	0	#DIV/0!	#DIV/0!	#DIV/0!
DG(33:2)_C16:0	0	0	0	0	0	#DIV/0!	#DIV/0!	#DIV/0!
DG(33:2)_C16:1	0	0	0	0	0	#DIV/0!	#DIV/0!	#DIV/0!
DG(35:3)_C18:1	0	0	0	0	0	#DIV/0!	#DIV/0!	#DIV/0!
DG(dO-38:9),DG(35:2)_C18:1	0	0	0	0	0	#DIV/0!	#DIV/0!	#DIV/0!

DG(dO-38:9),DG(35:2)_C18:2	0	0	0	0	0	#DIV/0!	#DIV/0!	#DIV/0!
DG(O-38:8),DG(36:1)_C16:0	0	0	0	0	0	#DIV/0!	#DIV/0!	#DIV/0!
DG(38:9),DG(dO-40:9),DG(37:2	0	0	0	0	0	#DIV/0!	#DIV/0!	#DIV/0!
DG(38:8),DG(dO-40:8),DG(37:1	0	0	0	0	0	#DIV/0!	#DIV/0!	#DIV/0!
DG(38:8),DG(dO-40:8),DG(37:1	0	0	0	0	0	#DIV/0!	#DIV/0!	#DIV/0!
DG(38:7)_C18:1	0	0	0	0	0	#DIV/0!	#DIV/0!	#DIV/0!
CE(16:3)K	0	0	0	0	0	#DIV/0!	#DIV/0!	#DIV/0!
CE(16:2)K	0	0	0	0	0	#DIV/0!	#DIV/0!	#DIV/0!
DG(39:8),DG(O-40:8),DG(38:1)	0	0	0	0	0	#DIV/0!	#DIV/0!	#DIV/0!
DG(39:8),DG(O-40:8),DG(38:1)	0	0	0	0	0	#DIV/0!	#DIV/0!	#DIV/0!
	0	0	0	0	0	#DIV/0!	#DIV/0!	#DIV/0!
CE(17:0)K	0	0	0	0	0	#DIV/0!	#DIV/0!	#DIV/0!
DG(40:9),DG(39:2)_C18:1	0	0	0	0	0	#DIV/0!	#DIV/0!	#DIV/0!
DG(40:8),DG(39:1)_C16:0	0	0	0	0	0	#DIV/0!	#DIV/0!	#DIV/0!
DG(40:8),DG(39:1)_C16:1	0	0	0	0	0	#DIV/0!	#DIV/0!	#DIV/0!
DG(40:7)_C18:1	0	0	0	0	0	#DIV/0!	#DIV/0!	#DIV/0!
DG(40:2)_C18:1	0	0	0	0	0	#DIV/0!	#DIV/0!	#DIV/0!
DG(40:2)_C16:1	0	0	0	0	0	#DIV/0!	#DIV/0!	#DIV/0!
DG(40:1)_C18:1	0	0	0	0	0	#DIV/0!	#DIV/0!	#DIV/0!
DG(40:1)_C16:0	0	0	0	0	0	#DIV/0!	#DIV/0!	#DIV/0!
DG(40:1)_C16:1	0	0	0	0	0	#DIV/0!	#DIV/0!	#DIV/0!
DG(41:7)_C18:1	0	0	0	0	0	#DIV/0!	#DIV/0!	#DIV/0!
DG(42:10),DG(41:3)_C18:1	0	0	0	0	0	#DIV/0!	#DIV/0!	#DIV/0!
DG(42:9),DG(41:2)_C18:1	0	0	0	0	0	#DIV/0!	#DIV/0!	#DIV/0!
DG(42:9),DG(41:2)_C18:2	0	0	0	0	0	#DIV/0!	#DIV/0!	#DIV/0!
DG(42:8),DG(41:1)_C16:1	0	0	0	0	0	#DIV/0!	#DIV/0!	#DIV/0!
DG(42:7),DG(41:0)_C18:0	0	0	0	0	0	#DIV/0!	#DIV/0!	#DIV/0!
DG(42:7)_C18:1	0	0	0	0	0	#DIV/0!	#DIV/0!	#DIV/0!
DG(42:2)_C18:1	0	0	0	0	0	#DIV/0!	#DIV/0!	#DIV/0!
DG(42:2)_C16:1	0	0	0	0	0	#DIV/0!	#DIV/0!	#DIV/0!
DG(42:1)_C18:1	0	0	0	0	0	#DIV/0!	#DIV/0!	#DIV/0!
DG(42:1)_C16:0	0	0	0	0	0	#DIV/0!	#DIV/0!	#DIV/0!
DG(42:1)_C16:1	0	0	0	0	0	#DIV/0!	#DIV/0!	#DIV/0!
DG(42:0)_C18:0	0	0	0	0	0	#DIV/0!	#DIV/0!	#DIV/0!
DG(43:6)_C16:0	0	0	0	0	0	#DIV/0!	#DIV/0!	#DIV/0!
DG(44:9),DG(43:2)_C16:1	0	0	0	0	0	#DIV/0!	#DIV/0!	#DIV/0!
DG(44:8),DG(43:1)_C16:1	0	0	0	0	0	#DIV/0!	#DIV/0!	#DIV/0!
DG(44:7)_C18:1	0	0	0	0	0	#DIV/0!	#DIV/0!	#DIV/0!
DG(44:6)_C16:0	0	0	0	0	0	#DIV/0!	#DIV/0!	#DIV/0!
DG(44:2)_C18:1	0	0	0	0	0	#DIV/0!	#DIV/0!	#DIV/0!
DG(44:2)_C16:1	0	0	0	0	0	#DIV/0!	#DIV/0!	#DIV/0!
DG(44:1)_C16:0	0	0	0	0	0	#DIV/0!	#DIV/0!	#DIV/0!
DG(44:1)_C16:1	0	0	0	0	0	#DIV/0!	#DIV/0!	#DIV/0!
DG(44:0)_C18:0	0	0	0	0	0	#DIV/0!	#DIV/0!	#DIV/0!
FA(7:1)	0	0	0	0	0	#DIV/0!	#DIV/0!	#DIV/0!
FA(7:0)	0	0	0	0	0	#DIV/0!	#DIV/0!	#DIV/0!
FA(8:6)	0	0	0	0	0	#DIV/0!	#DIV/0!	#DIV/0!
FA(8:1)	0	0	0	0	0	#DIV/0!	#DIV/0!	#DIV/0!
FA(8:0)	0	0	0	0	0	#DIV/0!	#DIV/0!	#DIV/0!
FA(10:6)	0	0	0	0	0	#DIV/0!	#DIV/0!	#DIV/0!
FA(10:1)	0	0	0	0	0	#DIV/0!	#DIV/0!	#DIV/0!
FA(10:0); FA(10:0)	0	0	0	0	0	#DIV/0!	#DIV/0!	#DIV/0!
FA(11:6)	0	0	0	0	0	#DIV/0!	#DIV/0!	#DIV/0!
FA(12:5)	0	0	0	0	0	#DIV/0!	#DIV/0!	#DIV/0!
FA(12:0)	0	0	0	0	0	#DIV/0!	#DIV/0!	#DIV/0!
FA(14:7)	0	0	0	0	0	#DIV/0!	#DIV/0!	#DIV/0!
FA(13:0)	0	0	0	0	0	#DIV/0!	#DIV/0!	#DIV/0!
FA(14:0)	0	0	0	0	0	#DIV/0!	#DIV/0!	#DIV/0!
FA(15:6)	0	0	0	0	0	#DIV/0!	#DIV/0!	#DIV/0!
FA(16:5)	0	0	0	0	0	#DIV/0!	#DIV/0!	#DIV/0!
FA(17:4)	0	0	0	0	0	#DIV/0!	#DIV/0!	#DIV/0!
FA(18:7)	0	0	0	0	0	#DIV/0!	#DIV/0!	#DIV/0!
FA(17:0)	0	0	0	0	0	#DIV/0!	#DIV/0!	#DIV/0!
FA(19:4)	0	0	0	0	0	#DIV/0!	#DIV/0!	#DIV/0!
FA(20:4)	0	0	0	0	0	#DIV/0!	#DIV/0!	#DIV/0!
FA(21:3)	0	0	0	0	0	#DIV/0!	#DIV/0!	#DIV/0!

FA(22:4)	0	0	0	0	0	#DIV/0!	#DIV/0!	#DIV/0!
FA(22:3)	0	0	0	0	0	#DIV/0!	#DIV/0!	#DIV/0!
FA(28:7)	0	0	0	0	0	#DIV/0!	#DIV/0!	#DIV/0!
FA(28:5)	0	0	0	0	0	#DIV/0!	#DIV/0!	#DIV/0!
FA(29:3)	0	0	0	0	0	#DIV/0!	#DIV/0!	#DIV/0!
FA(30:5)	0	0	0	0	0	#DIV/0!	#DIV/0!	#DIV/0!
FA(31:1)	0	0	0	0	0	#DIV/0!	#DIV/0!	#DIV/0!
FA(32:6)	0	0	0	0	0	#DIV/0!	#DIV/0!	#DIV/0!
FA(32:4)	0	0	0	0	0	#DIV/0!	#DIV/0!	#DIV/0!
FA(34:5)	0	0	0	0	0	#DIV/0!	#DIV/0!	#DIV/0!
FA(46:0)	0	0	0	0	0	#DIV/0!	#DIV/0!	#DIV/0!
FA(4:0)	0	0	0	0	0	#DIV/0!	#DIV/0!	#DIV/0!
CE(22:2) NH4	0	0	0	0	0	#DIV/0!	#DIV/0!	#DIV/0!

Table S6. Correlation Analysis of Fe3O4 NP-BC Lipid Abundance in Relation to Serum Concentration
BC Lipid Abundance Correlation Analysis with Female 50 nm Samples

Serum concentration (% v/v)	5	10	25	50	75			
	Group Mean Relative Lipid Abundance							
Lipid name	Female 5% 50 nm	Female 10% 50nm	Female 25% 50nm	Female 50% 50nm	Female 75% 50nm	Pearson's Correlation	R^2	p-value
[TG(53:7)]_C18:1	771.5640549	1073.312077	1416.220094	3142.472241	3934.308305	0.988857475	0.977839	0.00141
[TG(49:8),TG(48:1)]_C18:1	0	999.2200722	2217.948156	6918.000462	8588.480592	0.986011916	0.972219	0.00198
[TG(52:5)]_C18:3	0	762.368055	1718.968112	5073.236337	6100.51641	0.983309638	0.966898	0.00258
[TG(50:7),TG(49:0)]_C16:0	1502.1201	1525.080104	1835.344128	2237.164172	2387.736169	0.981297578	0.962945	0.00306
[TG(49:8),TG(48:1)]_C14:0	879.7160622	1418.55609	1421.9961	3890.512275	5378.056345	0.980711791	0.961796	0.00321
[TG(39:0)]_C20:0	0	950.2680656	1679.36412	11459.50481	16180.63325	0.980313576	0.961015	0.00331
[TG(51:8),TG(50:1)]_C18:1	3943.056276	4664.904315	8365.372618	44026.1832	60576.37985	0.977829932	0.956151	0.00395
[TG(51:8),TG(50:1)]_C18:0	1205.312087	1467.664104	1809.084122	4388.724316	5189.956395	0.977453536	0.955415	0.00405
[TG(55:9),TG(54:2)]_C18:0	2090.492151	2420.624164	4491.976318	19284.69736	24762.05365	0.977310075	0.955135	0.00409
[TG(52:4)]_C18:3	0	1179.232085	2171.216147	7856.204555	9156.832648	0.9754666	0.951535	0.0046
[TG(51:8),TG(50:1)]_C16:0	6177.612452	7033.224477	13260.88097	79091.75351	107112.4903	0.975289386	0.951189	0.00465
[TG(51:9),TG(50:2)]_C18:2	1865.748135	2492.252183	4079.588301	24712.80951	33576.69026	0.975028994	0.950682	0.00472
PC(32:0),PC(O-33:0)	0	4511.888323	8138.052553	31066.20204	36015.58652	0.973756338	0.948201	0.00508
[TG(54:10),TG(53:3)]_C18:1	0	1079.316076	1609.836124	3872.016283	4521.70832	0.973353635	0.947417	0.0052
[TG(53:8),TG(52:1)]_C18:1	4001.756284	4420.332334	7715.636551	38397.87877	49074.01134	0.973080664	0.946886	0.00528
[TG(51:9),TG(50:2)]_C16:0	3626.840237	3950.408285	8798.416599	55439.5156	72291.61313	0.97297253	0.946676	0.00531
[TG(50:3)]_C14:0	0	774.2640541	1620.576115	5401.636401	6087.932363	0.972771413	0.946284	0.00537
[TG(55:10),TG(54:3)]_C18:0	1876.14012	2121.380152	3795.020263	18358.04107	23019.00956	0.972399055	0.94556	0.00548
[TG(53:9),TG(52:2)]_C18:2	2063.980143	2462.876166	4578.296309	24262.39368	30505.73012	0.971838392	0.94447	0.00565
[TG(55:11),TG(54:4)]_C18:0	1150.108085	1164.852079	2102.704139	7549.00452	8931.59668	0.971305192	0.943434	0.00581
[TG(55:10),TG(54:3)]_C18:2	1749.112128	2079.524142	3727.492274	18384.92938	22623.03359	0.970656065	0.942173	0.00601
[TG(53:9),TG(52:2)]_C18:0	1939.02813	2245.24817	3597.452237	19101.27718	24045.9219	0.970099972	0.941094	0.00618
[TG(50:3)]_C16:0	0	1731.820135	2587.948186	11959.27285	13810.52099	0.968545271	0.93808	0.00667
[TG(52:5)]_C18:2	833.8920578	1415.216102	2233.440147	9605.212711	11088.86083	0.966940363	0.934974	0.00718
[TG(53:10),TG(52:3)]_C16:0	6258.448483	7939.432571	21010.85762	151857.256	185572.1052	0.966656868	0.934426	0.00727
[TG(52:4)]_C18:1	1606.316122	1804.584128	3361.812209	16492.93707	19509.53353	0.966249452	0.933638	0.00741
[TG(51:7),TG(50:0)]_C16:0	6224.376471	6465.740446	6739.796464	14463.68097	16937.84117	0.965707474	0.932591	0.00758
[TG(49:7),TG(48:0)]_C18:0	1172.076083	1470.036107	1498.868111	2474.580175	2672.632193	0.965063196	0.931347	0.0078
[TG(53:9),TG(52:2)]_C18:1	10640.65276	13333.46497	29898.80999	212058.6116	256837.303	0.965051318	0.931324	0.0078
[TG(53:9),TG(52:2)]_C16:0	6848.792436	7377.616518	18560.80118	124178.9531	148932.2071	0.964777066	0.930795	0.00789
[TG(52:4)]_C16:1	1141.67207	1503.296101	2275.788169	10920.86069	12791.765	0.964384258	0.930037	0.00803
[TG(54:5)]_C18:2	2540.624168	3452.260239	7186.884511	43265.17467	50923.23971	0.964164003	0.929612	0.0081
[TG(50:3)]_C18:2	1272.352081	1905.132137	2953.460226	15484.7691	18010.02534	0.963093175	0.927548	0.00846
[TG(55:10),TG(54:3)]_C18:1	5631.932397	6592.064452	15325.53306	94790.73879	110721.4189	0.962718091	0.926826	0.00859
[TG(54:5)]_C18:1	2196.648151	2736.684188	5019.672374	28761.61787	33491.94227	0.962131912	0.925698	0.0088
SM(d16:0/22:0)	0	4672.524345	13115.18895	89533.89049	101859.0353	0.962107389	0.925651	0.0088
[TG(53:7),TG(52:0)]_C18:0	3625.436217	3999.224293	4312.616299	7242.604541	7640.572533	0.962003316	0.92545	0.00884
[TG(53:10),TG(52:3)]_C18:1	6787.372482	8505.924602	20633.68143	154983.3703	181774.5729	0.960828384	0.923191	0.00925
[TG(52:4)]_C18:2	4024.120293	5405.128381	13065.37688	91275.48672	104946.5993	0.9595449	0.920726	0.00971
[TG(52:4)]_C16:0	2848.084197	3654.708261	8077.652542	52795.86372	60543.66022	0.959508228	0.920656	0.00972
[TG(54:6)]_C18:2	1528.588107	1762.656128	2950.096203	13752.55282	15471.91301	0.959325335	0.920305	0.00979
[TG(54:9),TG(53:2)]_C18:1	0	809.3800591	1612.828119	4396.276318	4571.652335	0.959112997	0.919898	0.00986
[TG(53:10),TG(52:3)]_C18:2	5729.276392	7948.164548	19941.72142	144484.8416	164927.2672	0.9582975	0.918334	0.01016
[TG(49:7),TG(48:0)]_C16:0	8039.880595	8236.260592	8705.8166	17446.41332	19087.23741	0.956598896	0.915081	0.01078
[TG(50:3)]_C18:1	1225.872089	1493.632104	2064.556149	8210.200547	9006.436569	0.955990647	0.913918	0.01101
PC(34:1),PC(O-35:1),PC(P-35:0	0	16895.49721	38742.68679	302262.2868	335348.1132	0.955907786	0.91376	0.01104
[TG(55:11),TG(54:4)]_C18:1	4542.860355	5594.696407	12621.21689	87141.51398	97790.35924	0.955489275	0.91296	0.0112
[TG(55:11),TG(54:4)]_C18:2	3177.592241	3759.700275	8081.412536	51435.93543	57362.86415	0.955260552	0.912523	0.01128
PC(O-38:9),PC(36:2),PC(O-37:2	0	13345.93701	40648.5069	366990.5173	409329.5943	0.954931496	0.911894	0.01141
PC(34:2),PC(O-35:2),PC(P-35:1	21322.46158	26027.20984	66110.91652	610092.8406	690302.4076	0.953373957	0.908922	0.012
PC(38:5)	0	2070.708131	6034.268407	59627.19641	63878.24426	0.947273521	0.897327	0.01442
PC(38:4)	0	4508.54829	16133.82517	168235.7967	179846.644	0.946413237	0.895698	0.01477
PC(36:3),PC(P-37:2)	0	6415.016449	18632.22534	197397.5657	208759.0458	0.944163183	0.891444	0.01571
PC(38:6)	2731.296191	3626.78026	7232.372488	70349.90104	75301.01763	0.943074032	0.889389	0.01616
PC(40:6)	1256.604089	1706.900125	3258.984231	22352.60964	23221.72985	0.941896677	0.887169	0.01667
PS(O-29:0)	5348.500362	7281.580492	7640.924554	8561.512594	9143.680606	0.886683815	0.786208	0.045
[TG(49:8),TG(48:1)]_C16:1	0	0	1120.744081	3333.64422	4339.224294	0.989903007	0.979908	0.00122
[TG(55:9),TG(54:2)]_C16:0	0	0	1153.54408	3140.596223	4071.168291	0.989887406	0.979877	0.00122
[TG(52:8),TG(51:1)]_C16:0	0	0	912.5560616	2720.620202	3518.332228	0.98935832	0.97883	0.00132
[TG(48:2)]_C18:2	0	0	1548.416113	4535.800303	5575.808387	0.984593169	0.969424	0.00229
[TG(55:9),TG(54:2)]_C18:2	0	0	1216.076084	3855.976266	4758.580344	0.984528214	0.969296	0.0023

[TG(57:11),TG(56:4)]_C18:1	0	0	1129.064082	3608.940249	4302.388286	0.980567539	0.961513	0.00324
[TG(48:2)]_C16:0	0	0	1811.564132	5176.436311	6125.708389	0.980125293	0.960646	0.00335
SM(d16:1/17:0)	0	0	3463.648243	12328.27693	14624.99699	0.978899719	0.958245	0.00367
[TG(56:6)]_C20:4	0	0	1201.396082	4399.072325	5215.63241	0.97847821	0.95742	0.00378
[TG(54:6)]_C18:3	0	0	813.2040512	3658.920266	4457.112305	0.978394424	0.957256	0.0038
[TG(51:7)]_C18:1	0	0	1497.84011	4225.712302	4934.836308	0.978363339	0.957195	0.00381
[TG(54:5)]_C20:4	0	0	1620.152115	5489.008385	6446.808463	0.978201091	0.956877	0.00385
[TG(51:8),TG(50:1)]_C14:0	0	0	836.8440559	2092.144138	2430.912161	0.976826892	0.954191	0.00422
[TG(51:8),TG(50:1)]_C16:1	0	0	1535.956112	3233.436237	3861.056248	0.97667088	0.953886	0.00426
[TG(55:10),TG(54:3)]_C16:0	0	0	942.5640678	4037.548267	4806.360358	0.97664375	0.953833	0.00427
[TG(54:5)]_C18:3	0	0	1552.584109	4298.924286	4939.168284	0.976020783	0.952617	0.00444
PC(28:1),PC(P-29:0)	0	0	3024.156203	9127.81664	10465.8208	0.975734176	0.952057	0.00452
[TG(46:0)]_C16:0	2303.776156	2934.348212	2915.052209	5775.540419	6923.44449	0.974984135	0.950594	0.00473
PC(31:1),PC(O-32:1),PC(P-32:0	0	0	1829.352129	6001.060451	6839.900495	0.974431083	0.949516	0.00489
PC(30:0),PC(O-31:0)	0	0	3287.568213	10637.11677	12107.58892	0.974336423	0.949331	0.00492
[TG(51:9),TG(50:2)]_C16:1	1464.15211	1409.048102	2956.380209	11926.82888	14645.30899	0.974122311	0.948914	0.00498
[TG(53:8),TG(52:1)]_C18:0	3946.392277	3847.812259	5956.756437	27962.34177	36872.17455	0.973308245	0.947329	0.00521
[TG(52:10),TG(51:3)]_C18:2	0	0	1076.812075	2909.64419	3278.880231	0.972963211	0.946657	0.00531
SM(d16:0/18:0)	0	0	7578.428539	32493.85414	37499.00634	0.972765957	0.946274	0.00537
PC(35:2),PC(O-36:2),PC(P-36:1	0	0	3228.532216	16314.74913	19245.24133	0.972765117	0.946272	0.00537
[TG(54:5)]_C16:0	0	0	934.1800652	3547.840253	4020.864299	0.972063512	0.944907	0.00558
PC(30:1),PC(O-31:1),PC(P-31:0	0	0	21895.24939	112437.8442	131735.1183	0.971654995	0.944113	0.0057
[TG(55:8),TG(54:1)]_C18:0	2467.992168	2091.072143	2778.092195	7729.856529	9780.020697	0.971491986	0.943797	0.00575
[TG(52:5)]_C16:1	0	0	1150.62407	3783.868275	4223.840316	0.971320452	0.943463	0.00581
[TG(52:5)]_C16:0	0	0	1557.096108	4936.59637	5496.588393	0.971148263	0.943129	0.00586
[TG(53:10),TG(52:3)]_C18:0	0	0	821.4080627	3002.316201	3366.056219	0.970968133	0.942779	0.00591
SM(d16:1/22:1)	0	0	3539.992271	19380.5734	22752.88165	0.970786758	0.942427	0.00597
[TG(54:6)]_C18:1	0	0	1470.664101	3671.100272	4103.752306	0.970767548	0.94239	0.00597
[TG(55:11),TG(54:4)]_C16:0	0	0	1542.084107	4161.528292	4622.592292	0.970635596	0.942133	0.00601
[TG(55:9),TG(54:2)]_C18:1	3344.404238	3214.760229	6406.232413	31192.01441	38575.97084	0.970624057	0.942111	0.00602
SM(d16:1/16:0)	0	0	5593.416376	21305.78953	23908.3936	0.970589282	0.942044	0.00603
PC(34:0),PC(O-35:0)	0	0	6038.520405	29933.85406	34569.81062	0.970437377	0.941749	0.00607
PC(33:1),PC(O-34:1),PC(P-34:0	0	0	2693.824189	9926.244731	11091.75271	0.970373916	0.941626	0.00609
[TG(49:8),TG(48:1)]_C16:0	2223.840147	2038.744141	2746.868182	10192.65277	12958.0928	0.970092975	0.94108	0.00618
[TG(53:9),TG(52:2)]_C16:1	0	0	828.4800598	3325.872249	3737.676264	0.970085786	0.941066	0.00618
SM(d18:1/19:0)	0	0	1680.884104	9173.512603	10701.43673	0.97005252	0.941002	0.00619
[TG(54:10),TG(53:3)]_C18:2	0	0	1086.612077	2826.296211	3132.820222	0.969926541	0.940757	0.00623
[TG(52:9),TG(51:2)]_C16:0	0	0	1166.668079	3288.220233	3628.57627	0.969801075	0.940514	0.00627
PC(30:2),PC(P-31:1)	0	0	3608.416237	14688.97702	16478.45307	0.969624318	0.940171	0.00633
[TG(48:2)]_C14:0	0	0	868.2160538	3282.92823	3655.94025	0.969544025	0.940016	0.00635
PC(31:0),PC(O-32:0)	0	0	2667.192184	6995.268497	7705.688531	0.968963804	0.938891	0.00653
PC(35:4),PC(O-36:4),PC(P-36:3	0	0	3548.836246	20905.89342	24401.7776	0.968792545	0.938559	0.00659
[TG(51:9),TG(50:2)]_C14:0	842.0640602	837.4800568	1503.316105	4904.056347	5607.272372	0.968692271	0.938365	0.00662
SM(d16:1/18:0)	0	0	59233.73599	312002.1827	356300.6147	0.967891807	0.936815	0.00687
SM(d16:1/20:1)	0	0	5088.400356	25791.28978	29294.41421	0.967818225	0.936672	0.0069
[TG(56:7)]_C22:6	0	0	1047.184076	2607.604179	2861.124203	0.967531003	0.936116	0.00699
[TG(53:8),TG(52:1)]_C16:0	3954.840281	3572.628267	6094.268427	29547.77429	36265.89857	0.967129009	0.935339	0.00712
[TG(51:9),TG(50:2)]_C18:1	2493.47618	2351.648164	4600.280335	22534.60562	26881.46174	0.966159671	0.933465	0.00743
LPC(18:0),PC(O-18:0),LPC(O-1	0	0	9485.188736	58653.47199	67486.3204	0.96611148	0.933371	0.00745
PC(37:7),PC(P-38:6),PC(36:0),F	0	0	1575.900126	10711.76066	12465.26881	0.965973759	0.933105	0.0075
[TG(53:10),TG(52:3)]_C16:1	1105.872074	849.800058	2147.020149	8534.296596	9814.268693	0.965958785	0.933076	0.0075
PC(O-38:8),PC(36:1),PC(O-37:1	0	0	15486.73305	98871.19877	114074.2393	0.965926957	0.933015	0.00751
SM(d18:1/17:0)	0	0	1809.492124	8554.832596	9506.212641	0.965853994	0.932874	0.00754
[TG(50:4)]_C16:1	0	0	1076.444069	2364.888169	2615.952176	0.9656634	0.932506	0.0076
PC(40:4)	0	0	1990.216135	8333.832577	9087.932674	0.964872807	0.93098	0.00786
SM(d16:1/18:1)	0	0	6304.22044	34124.87837	38122.01056	0.964322461	0.929918	0.00805
[TG(57:10),TG(56:3)]_C18:1	0	0	1196.324079	3285.372234	3507.444267	0.963845517	0.928998	0.00821
PC(38:9),PC(37:2),PC(O-38:2),I	0	0	2859.69219	19078.95726	21739.50153	0.963515402	0.928362	0.00832
Cer(d18:1/24:0)	0	0	1089.580076	3011.524207	3206.328239	0.963392412	0.928125	0.00836
SM(d16:0/24:0)	0	0	4183.844264	25497.33774	28625.11006	0.962983594	0.927337	0.0085
SM(d16:0/23:0)	0	0	1312.952089	6646.540471	7294.168533	0.962805323	0.926994	0.00856
PC(40:5)	0	0	2586.996174	13633.61693	14968.1651	0.962179052	0.925789	0.00878
[TG(54:6)]_C20:4	0	0	1472.900101	4321.284324	4553.81234	0.961790967	0.925042	0.00891
PC(32:1),PC(O-33:1),PC(P-33:0	0	0	5540.516387	31220.57811	34415.1544	0.961639428	0.92475	0.00897
[TG(55:8),TG(54:1)]_C18:1	1739.852132	1716.040121	2174.112165	5707.5884	6363.312438	0.961611163	0.924696	0.00898
SM(d16:1/20:0)	0	0	11264.44481	60243.48771	65914.96846	0.961396868	0.924284	0.00905
[TG(56:7)]_C18:2	0	0	809.7520592	2502.720193	2620.412171	0.96062637	0.922803	0.00932
SM(d16:1/24:0)	0	0	24485.15367	180755.2412	203122.574	0.95990927	0.921426	0.00958

SM(d16:1/22:0)	0	0	29906.24998	247668.4308	281472.1322	0.959685939	0.920997	0.00966
PC(29:1),PC(O-30:1),PC(P-30:0	0	0	2280.692162	5108.040363	5443.592365	0.959206627	0.920077	0.00983
SM(d16:0/20:0)	0	0	2979.808192	10706.45275	11152.38884	0.958856995	0.919407	0.00996
[TG(57:12),TG(56:5)]_C18:1	0	0	1381.200091	3525.428265	3684.744266	0.95881496	0.919326	0.00997
PC(36:8),PC(35:1),PC(O-36:1),I	0	0	3371.756226	20133.22128	21928.20544	0.958781555	0.919262	0.00998
[TG(50:3)]_C16:1	1695.240131	1250.16809	2159.720146	9516.544622	11046.21677	0.958357516	0.918449	0.01014
[TG(50:4)]_C18:2	0	0	1661.11612	5474.048393	5661.012428	0.95809597	0.917948	0.01023
SM(d18:2/24:1)	0	0	13795.189	116031.891	127171.1256	0.95415214	0.910406	0.0117
[TG(48:2)]_C18:1	913.9880615	0	1285.312087	3271.940221	3926.060299	0.952964931	0.908142	0.01216
PC(40:8),PC(39:1),PC(O-40:1),I	0	0	1115.096078	4991.304365	5110.784364	0.952900068	0.908019	0.01218
[TG(57:12),TG(56:5)]_C20:4	0	0	818.9640564	3173.076232	3216.452232	0.952880166	0.907981	0.01219
PC(38:3)	0	0	10750.03674	90881.35477	97811.89848	0.951198589	0.904779	0.01285
PC(35:3),PC(O-36:3),PC(P-36:2	0	0	1876.360136	11379.66877	11812.68886	0.950427299	0.903312	0.01315
SM(d16:1/24:1)	0	0	12888.00488	114572.7678	123238.3808	0.950353634	0.903172	0.01318
PC(37:4),PC(O-38:4),PC(P-38:3	0	0	3402.288219	19476.80537	20051.8735	0.949909648	0.902328	0.01336
[TG(52:9),TG(51:2)]_C18:1	0	824.6240585	1576.676104	4325.584331	4311.776339	0.949235908	0.901049	0.01362
PC(33:2),PC(O-34:2),PC(P-34:1	0	0	2051.86415	13413.86096	13909.00101	0.948965547	0.900536	0.01373
[TG(56:7)]_C20:4	0	0	795.9880569	3726.24825	3737.924271	0.948253835	0.899185	0.01402
PC(28:0),PC(O-29:0)	0	0	1638.488124	3282.116238	3408.700242	0.94820222	0.899087	0.01404
PC(37:5),PC(O-38:5),PC(P-38:4	0	0	2641.71217	18026.27324	18605.58541	0.947420008	0.897605	0.01436
[TG(53:7),TG(52:0)]_C16:0	2413.572179	2076.716148	2856.372206	5280.892366	5372.560387	0.945270913	0.893537	0.01524
SM(d18:2/22:1)	0	0	10573.17678	107312.02	110812.2472	0.94147639	0.886378	0.01685
PC(36:5)	0	1363.072098	2226.832156	10249.32078	10043.99271	0.940144816	0.883872	0.01742
[TG(51:7),TG(50:0)]_C18:0	4149.156303	3971.21228	4129.252285	7457.552547	7477.752447	0.921813553	0.84974	0.02593
PC(36:4),PC(O-37:4)	8385.160605	10449.32478	24756.84152	287866.0333	275881.9429	0.920395968	0.847129	0.02664
[TG(48:2)]_C16:1	1089.640082	0	869.2760628	2590.132189	3121.912225	0.913092796	0.833738	0.03035
LPG(20:0); LPG(20:0)	0	0	2277.764166	3055.976218	3113.760229	0.890456466	0.792913	0.0428
[TG(49:6)]_C16:0	935.4560661	1619.028115	1370.616099	2156.512151	2265.764149	0.886515386	0.78591	0.0451
PG(16:0),LPG(17:0),LPG(O-18:(	2248.73616	4319.912297	1817.840133	2412.31217	2950.668198	-0.105325684	0.011093	0.86614
[TG(52:8),TG(51:1)]_C18:0	0	0	0	914.6440659	1554.256102	0.967885477	0.936802	0.00688
[TG(50:5)]_C18:2	0	0	0	848.7920609	1442.800094	0.967884552	0.936801	0.00688
PC(44:10),PC(O-44:3)	0	0	0	1020.200072	1759.948116	0.967809375	0.936655	0.0069
PI(36:2),PI(O-37:2),PI(P-37:1)	0	0	0	1673.700121	2744.480201	0.967807157	0.936651	0.0069
[TG(55:7),TG(54:0)]_C20:0	0	0	0	857.6240585	1405.168105	0.967801036	0.936639	0.0069
CE(20:2)K	0	0	0	3978.972264	6465.436452	0.96772677	0.936495	0.00693
[TG(53:8),TG(52:1)]_C20:0	0	0	0	800.6840553	1397.888099	0.96770453	0.936452	0.00693
CE(19:0)H	0	0	0	159619.1884	281908.2241	0.967567122	0.936186	0.00698
CE(22:5) NH4	0	0	0	3727.824259	5945.636429	0.967483253	0.936024	0.007
PE(34:2),PE(O-35:2),PE(P-35:1)	0	0	0	992.9640663	1764.216134	0.96748222	0.936022	0.007
CE(20:1) NH4	0	0	0	6476.100447	10322.30076	0.967472882	0.936004	0.00701
CE(18:2) NH4	0	0	0	808374.0091	1444310.22	0.967394379	0.935852	0.00703
DG(O-40:9),DG(38:2)_C18:2	0	0	0	730908.8501	1312427.843	0.967309421	0.935688	0.00706
CE(18:1) NH4	0	0	0	93392.05874	171280.0919	0.9668799	0.934857	0.0072
DG(39:8),DG(O-40:8),DG(38:1)_	0	0	0	82043.72138	150769.7318	0.966833446	0.934767	0.00721
[TG(50:9),TG(49:2)]_C18:1	0	0	0	1014.424067	1554.108114	0.966579405	0.934276	0.0073
[TG(49:8),TG(48:1)]_C18:0	0	0	0	863.1320557	1605.596125	0.966531005	0.934182	0.00731
CE(16:3)Na	0	0	0	2887.020212	4368.756295	0.966195662	0.933534	0.00742
CE(16:0)K	0	0	0	20268.06946	30643.36609	0.966166168	0.933477	0.00743
DG(39:8),DG(O-40:8)_C18:2	0	0	0	33434.73045	49831.67142	0.96565133	0.932482	0.0076
PS(38:4)	0	0	0	1440.808107	2104.300151	0.964800053	0.930839	0.00789
CE(22:6) NH4	0	0	0	17296.13323	24723.33379	0.963736877	0.928789	0.00824
[TG(56:12),TG(55:5)]_C18:2	0	0	0	814.3000572	1160.456084	0.963573954	0.928475	0.0083
[TG(54:8),TG(53:1)]_C16:0	0	0	0	1111.304082	1574.244115	0.963240991	0.927833	0.00841
PC(34:6)	0	0	0	1424.028099	2013.460135	0.96313415	0.927627	0.00845
[TG(55:7)]_C18:1	0	0	0	878.8640656	1232.416091	0.962648027	0.926691	0.00862
[TG(54:9),TG(53:2)]_C18:2	0	0	0	1098.904077	1540.664097	0.962635863	0.926668	0.00862
[TG(53:8),TG(52:1)]_C16:1	0	0	0	823.0360573	1145.520076	0.962185649	0.925801	0.00878
[TG(49:7)]_C18:1	0	0	0	1177.768084	1634.908117	0.962017074	0.925477	0.00884
[TG(44:0),TG(O-45:0)]_C16:0	0	0	0	1963.200136	2701.868186	0.961451677	0.924389	0.00903
[TG(46:1)]_C14:0	0	0	0	1159.160079	1592.716106	0.961341855	0.924178	0.00907
CE(20:0) NH4	0	0	0	21420.09346	28968.08229	0.960214119	0.922011	0.00947
PE(38:6)	0	0	0	1109.872076	1458.872095	0.957951245	0.917671	0.01029
[TG(54:5)]_C18:0	0	0	0	1272.992088	1665.860112	0.95756896	0.916938	0.01042
[TG(54:10),TG(53:3)]_C16:0	0	0	0	1889.78014	2470.816171	0.957492098	0.916791	0.01045
PC(16:0),PC(O-17:0),LPC(O-18	0	0	0	2347.444173	3059.744198	0.957221381	0.916273	0.01055
Cer(d18:1/23:0)	0	0	0	1412.42009	1839.244131	0.957136869	0.916111	0.01058
[TG(37:0)]_C18:0	0	0	0	2584.604177	3340.068225	0.956447968	0.914793	0.01084
[TG(57:9),TG(56:2)]_C20:0	0	0	0	1845.752128	2384.588162	0.956422319	0.914744	0.01085

PE(38:4)	0	0	0	2977.852179	3835.18026	0.956133178	0.914191	0.01096
[TG(55:8),TG(54:1)]_C20:0	0	0	0	2871.412193	3680.256248	0.955678174	0.913321	0.01113
[TG(54:5)]_C22:5	0	0	0	1384.156099	1763.936123	0.955127829	0.912269	0.01133
[TG(55:9),TG(54:2)]_C20:0	0	0	0	2249.396154	2853.820202	0.954689717	0.911432	0.0115
[TG(56:11),TG(55:4)]_C18:1	0	0	0	1705.656123	2138.596164	0.953492603	0.909148	0.01196
PC(42:6)	0	0	0	1661.324126	2075.776154	0.953128839	0.908455	0.0121
[TG(57:12),TG(56:5)]_C22:5	0	0	0	1167.87608	1435.428101	0.951343709	0.905055	0.01279
Cer(d18:1/22:0)	0	0	0	1480.096113	1817.520132	0.951241687	0.904861	0.01283
[TG(52:10),TG(51:3)]_C16:0	0	0	0	1798.70412	2207.532159	0.951179075	0.904742	0.01285
CE(20:2)Na	0	0	0	6128.71244	7495.820565	0.950788867	0.903999	0.01301
[TG(44:1)]_C18:1	0	0	0	1032.856068	1260.456087	0.950535626	0.903518	0.01311
[TG(56:6)]_C16:0	0	0	0	1987.724153	2408.516164	0.949707537	0.901944	0.01344
[TG(54:11),TG(53:4)]_C18:1	0	0	0	1842.544126	2230.760148	0.949610276	0.90176	0.01348
PC(33:0),PC(O-34:0)	0	0	0	4117.356288	4962.008372	0.949063668	0.900722	0.01369
[TG(56:8)]_C22:6	0	0	0	2360.696168	2844.472218	0.949042287	0.900681	0.0137
[TG(54:8),TG(53:1)]_C18:0	0	0	0	1423.516105	1714.652114	0.949001398	0.900604	0.01372
[TG(57:10),TG(56:3)]_C20:0	0	0	0	1733.724125	2079.628151	0.948497976	0.899648	0.01392
[TG(38:1)]_C18:1	0	0	0	878.7200531	1053.75208	0.948464933	0.899586	0.01393
[TG(46:2)]_C18:2	0	0	0	1328.344098	1587.740107	0.948064483	0.898826	0.0141
[TG(38:0)]_C14:0	0	0	0	1245.404081	1487.272102	0.947954043	0.898617	0.01414
PI(38:3)	0	0	0	2235.772141	2668.296191	0.947876091	0.898469	0.01417
[TG(54:11),TG(53:4)]_C18:2	0	0	0	2826.020194	3370.804244	0.947805242	0.898335	0.0142
LPC(16:0),PC(O-16:0),LPC(O-1	0	0	0	65668.49639	78004.47339	0.947288703	0.897356	0.01441
[TG(50:3)]_C18:3	0	0	0	2139.040149	2539.13619	0.947203075	0.897194	0.01445
[TG(50:8),TG(49:1)]_C18:1	0	0	0	2033.188145	2399.460178	0.946461592	0.89579	0.01475
SM(d18:0/17:0)	0	0	0	4527.968339	5337.564375	0.946314594	0.895511	0.01481
[TG(57:8),TG(56:1)]_C20:0	0	0	0	1084.188077	1277.368093	0.946246784	0.895383	0.01484
[TG(55:8),TG(54:1)]_C16:0	0	0	0	1838.372119	2164.536156	0.946163462	0.895225	0.01487
[TG(57:12),TG(56:5)]_C18:2	0	0	0	2188.304163	2572.692184	0.94596901	0.894857	0.01495
CE(22:5)H	0	0	0	6094.444432	7124.864513	0.945233048	0.893466	0.01526
[TG(57:11),TG(56:4)]_C18:2	0	0	0	2277.224166	2657.800188	0.945011388	0.893047	0.01535
[TG(56:12),TG(55:5)]_C18:1	0	0	0	1438.404102	1678.02812	0.944950665	0.892932	0.01538
[TG(54:8),TG(53:1)]_C18:1	0	0	0	1641.652117	1911.276129	0.944681286	0.892423	0.01549
[TG(54:9),TG(53:2)]_C18:0	0	0	0	1443.088104	1674.872121	0.944261811	0.89163	0.01566
[TG(46:0)]_C18:0	0	0	0	1051.808076	1219.132085	0.944082548	0.891292	0.01574
PC(40:2)	0	0	0	4056.272301	4697.140328	0.943954869	0.891051	0.01579
PC(32:2),PC(O-33:2),PC(P-33:1	0	0	0	14599.36504	16857.73721	0.943564092	0.890313	0.01596
[TG(46:1)]_C18:1	0	0	0	1926.388133	2218.128165	0.943175926	0.889581	0.01612
PC(41:7),PC(P-42:6),PC(40:0),F	0	0	0	2011.312131	2315.856175	0.943172504	0.889574	0.01612
SM(d18:0/24:1)	0	0	0	52595.53137	60541.30433	0.943131292	0.889497	0.01614
[TG(52:8),TG(51:1)]_C18:1	0	0	0	2888.232209	3320.772246	0.942972744	0.889198	0.01621
SM(d18:0/24:0)	0	0	0	6472.732481	7425.244559	0.942656631	0.888602	0.01634
[TG(54:11),TG(53:4)]_C16:0	0	0	0	1586.152116	1816.616134	0.942428904	0.888172	0.01644
[TG(52:9),TG(51:2)]_C18:2	0	0	0	2068.112148	2364.148169	0.942163331	0.887672	0.01655
LPC(18:2),LPC(P-19:1)	0	0	0	5062.888367	5787.512403	0.942161044	0.887667	0.01655
[TG(57:9),TG(56:2)]_C18:1	0	0	0	1991.672145	2274.392171	0.942015553	0.887393	0.01661
[TG(53:8)]_C18:2	0	0	0	2281.388165	2604.124171	0.941955089	0.887279	0.01664
[TG(57:11),TG(56:4)]_C18:0	0	0	0	1622.320109	1851.668125	0.941943346	0.887257	0.01665
PC(O-40:9),PC(38:2),PC(P-39:1	0	0	0	67133.75279	76565.64926	0.941834115	0.887051	0.01669
[TG(57:11),TG(56:4)]_C20:0	0	0	0	1108.104079	1263.644098	0.941818106	0.887021	0.0167
[TG(56:7),TG(55:0)]_C16:0	0	0	0	1918.932135	2180.936154	0.941336711	0.886115	0.01691
SM(d18:0/26:1(17Z))	0	0	0	2785.752193	3158.812212	0.941003856	0.885488	0.01705
[TG(52:4)]_C20:4	0	0	0	2058.580132	2316.69615	0.939899084	0.88341	0.01753
[TG(49:3)]_C18:2	0	0	0	784.1640533	881.2440613	0.939690368	0.883018	0.01762
SM(d18:0/15:0)	0	0	0	2608.396181	2925.944226	0.939417433	0.882505	0.01774
PC(40:3)	0	0	0	3974.240308	4441.044299	0.938843881	0.881428	0.01799
LPC(18:1),PC(O-18:1),PC(P-18:	0	0	0	8026.95663	8937.88457	0.938304446	0.880415	0.01822
[TG(48:3)]_C18:1	0	0	0	1709.844122	1903.660121	0.9382866	0.880382	0.01823
PC(31:2),PC(O-32:2),PC(P-32:1	0	0	0	2049.652148	2280.868168	0.938211953	0.880242	0.01827
PC(39:8),PC(O-40:8),PC(38:1),I	0	0	0	25418.91373	28269.56201	0.938121374	0.880072	0.01831
[TG(46:1)]_C16:0	0	0	0	2289.360157	2545.972167	0.938113444	0.880057	0.01831
SM(d18:1/24:1(15Z))	0	0	0	144012.7144	160100.6924	0.938061694	0.87996	0.01833
[TG(46:0)]_C14:0	0	0	0	2276.77217	2528.008172	0.9378737	0.879607	0.01841
SM(d18:1/25:0)	0	0	0	5046.424345	5600.772373	0.93780492	0.879478	0.01844
[TG(51:9),TG(50:2)]_C18:0	0	0	0	2035.820142	2256.180174	0.937581934	0.87906	0.01854
[TG(58:9)]_C22:6	0	0	0	1199.236092	1327.888095	0.937447833	0.878808	0.0186
SM(d16:1/25:0)	0	0	0	28768.48597	31844.63026	0.937398894	0.878717	0.01862
PC(42:10),PC(41:3),PC(O-42:3)	0	0	0	2880.836191	3187.300237	0.937322408	0.878573	0.01866

PC(40:10),PC(39:3),PC(O-40:3)	0	0	2545.512175	14089.75699	13555.70893	0.936997598	0.877964	0.0188
[TG(51:8)]_C18:2	0	0	0	3297.920232	3635.708287	0.936765541	0.87753	0.01891
SM(d16:0/16:0)	0	0	0	3542.116244	3904.38428	0.936744268	0.87749	0.01892
PC(39:7),PC(P-40:6),PC(38:0),F	0	0	0	5945.600389	6521.008443	0.935958202	0.876018	0.01927
PC(35:6),PC(P-36:5)	0	0	0	2691.212192	2946.628197	0.935687312	0.875511	0.01939
PC(38:7),PC(37:0),PC(O-38:0)	0	0	0	3795.172303	4147.828294	0.935398153	0.87497	0.01952
PC(42:8),PC(41:1),PC(O-42:1),I	0	0	0	2287.908157	2498.716171	0.935283707	0.874756	0.01957
SM(d17:1/26:1)	0	0	0	8777.056631	9580.116676	0.935189161	0.874579	0.01961
SM(d17:1/24:1)	0	0	0	25386.18188	27589.634	0.934494217	0.873279	0.01993
PC(33:3),PC(O-34:3),PC(P-34:2	0	0	0	9940.82877	10796.54073	0.934387267	0.87308	0.01998
PC(24:0)	0	0	0	1326.280094	1440.392097	0.934381082	0.873068	0.01998
PC(42:3)	0	0	0	1980.340137	2150.448161	0.934360047	0.873029	0.01999
[TG(55:11),TG(54:4)]_C18:3	0	0	0	1951.892142	2117.640147	0.934213062	0.872754	0.02005
[TG(56:6)]_C18:2	0	0	0	2627.732185	2845.696223	0.933916887	0.872201	0.02019
[TG(52:10),TG(51:3)]_C18:1	0	0	0	2494.392169	2699.028195	0.933779502	0.871944	0.02025
LPC(20:4)	0	0	0	3738.004283	4043.220307	0.933721006	0.871835	0.02028
PC(42:5)	0	0	0	2114.772153	2287.41217	0.933718452	0.87183	0.02028
[TG(54:6)]_C16:0	0	0	0	2140.284152	2312.116164	0.933513353	0.871447	0.02037
[TG(50:8),TG(49:1)]_C16:0	0	0	0	2566.252183	2768.076196	0.933263233	0.87098	0.02049
[TG(54:9),TG(53:2)]_C16:0	0	0	0	1728.292117	1862.11613	0.933077141	0.870633	0.02057
[TG(50:9),TG(49:2)]_C16:0	0	0	0	1673.536122	1802.428133	0.933013581	0.870514	0.0206
[TG(44:2)]_C16:0	0	0	0	827.0840599	890.72006	0.93300165	0.870492	0.02061
[TG(53:7),TG(52:0)]_C20:0	0	0	0	1090.368073	1172.428085	0.932742364	0.870008	0.02073
PE(36:3),PE(P-37:2)	0	0	0	1368.67209	1470.412115	0.932599242	0.869741	0.02079
[TG(56:6)]_C22:5	0	0	1370.668093	2549.936185	2536.036172	0.932040512	0.8687	0.02105
[TG(55:11),TG(54:4)]_C20:4	0	0	0	2020.508129	2163.360149	0.932032395	0.868684	0.02105
SM(d17:0/27:0)	0	0	0	1573.47612	1679.208118	0.931479494	0.867654	0.02131
SM(d18:2/18:1)	0	0	0	2805.236198	2987.800209	0.931142578	0.867027	0.02146
[TG(46:2)]_C18:1	0	0	0	1529.132108	1628.640112	0.931141795	0.867025	0.02146
LPI(20:0)	0	0	0	2126.264142	2264.248172	0.931113077	0.866972	0.02148
SM(d16:1/23:0)	0	0	0	15718.70918	16733.90109	0.931063498	0.866879	0.0215
1-O-tricosanoyl-Cer(d18:1/16:0	0	0	0	1310.424081	1393.464102	0.930868607	0.866516	0.02159
SM(d18:1/26:1(17Z))	0	0	0	2811.152199	2989.116213	0.93085859	0.866498	0.0216
PC(36:7),PC(35:0),PC(O-36:0)	0	0	0	4003.068286	4254.388319	0.930774275	0.866341	0.02164
[TG(50:9),TG(49:2)]_C18:2	0	0	0	1734.940121	1842.444136	0.930642565	0.866096	0.0217
PC(40:9),PC(39:2),PC(O-40:2),I	0	0	0	7584.752569	8047.896515	0.930496955	0.865825	0.02177
[TG(52:6)]_C18:2	0	0	0	1204.664087	1278.100086	0.930480332	0.865794	0.02177
[TG(53:10),TG(52:3)]_C18:3	0	0	0	2330.764169	2470.960175	0.930349203	0.86555	0.02183
[TG(56:10),TG(55:3)]_C18:1	0	0	0	1744.932122	1840.784135	0.929495843	0.863963	0.02223
PC(37:3),PC(O-38:3),PC(P-38:2	0	0	3392.944244	24776.96966	23435.49351	0.928845887	0.862755	0.02254
PC(39:4),PC(O-40:4),PC(P-40:3	0	0	0	9711.804704	10198.00875	0.928687116	0.86246	0.02261
[TG(48:3)]_C16:1	0	0	0	1608.812125	1687.012115	0.928442879	0.862006	0.02273
SM(d18:2/21:0)	0	0	0	6478.608475	6792.66043	0.928420669	0.861965	0.02274
[TG(52:5)]_C18:1	0	0	0	1937.048136	2029.028153	0.928253794	0.861655	0.02282
PC(37:6),PC(O-38:6),PC(P-38:5	0	0	0	7570.796561	7912.372552	0.927852959	0.860911	0.02301
[TG(50:4)]_C18:3	0	0	0	1943.31613	2030.216154	0.927784901	0.860785	0.02304
PC(34:3),PC(P-35:2)	0	0	0	15433.50899	16106.85325	0.927599331	0.860441	0.02313
[TG(56:8)]_C20:4	0	0	0	1895.616126	1978.128136	0.927582097	0.860409	0.02314
SM(d18:2/15:0)	0	0	0	1050.016072	1094.492076	0.927381844	0.860037	0.02323
PC(38:8),PC(37:1),PC(O-38:1),I	0	0	0	19343.91751	20121.73352	0.927012495	0.859352	0.02341
[TG(57:9),TG(56:2)]_C18:0	0	0	0	1291.008085	1342.036091	0.926894321	0.859133	0.02347
[TG(54:12),TG(53:5)]_C18:2	0	0	0	833.1000572	865.5840652	0.926801912	0.858962	0.02351
PC(40:1),PC(P-41:0)	0	0	0	2849.408191	2960.184203	0.926782013	0.858925	0.02352
PC(35:5),PC(O-36:5),PC(P-36:4	0	0	0	14000.01292	14538.44496	0.926709639	0.858791	0.02355
PC(42:9),PC(41:2),PC(O-42:2),I	0	0	0	2339.644173	2429.552186	0.926704204	0.858781	0.02356
[TG(56:7)]_C22:5	0	0	0	2284.500156	2365.716169	0.926202807	0.857852	0.0238
[TG(52:6)]_C18:3	0	0	0	1552.588108	1607.604112	0.926182523	0.857814	0.02381
PC(42:2)	0	0	0	1932.712145	2000.460139	0.926115649	0.85769	0.02384
PI(38:4)	0	0	0	6866.224519	7105.480463	0.926079176	0.857623	0.02386
[TG(56:6)]_C18:0	0	0	0	1580.064114	1634.212119	0.925978075	0.857435	0.02391
PC(39:6),PC(O-40:6),PC(P-40:5	0	0	0	5188.292362	5364.516383	0.925924667	0.857336	0.02393
[TG(57:12),TG(56:5)]_C16:0	0	0	0	1727.336115	1782.180128	0.92553374	0.856613	0.02412
SM(d18:2/14:0)	0	0	0	2712.28419	2789.0122	0.924917511	0.855472	0.02442
[TG(51:4)]_C18:2	0	0	0	1880.27613	1932.724126	0.924846725	0.855341	0.02445
[TG(48:3)]_C18:2	0	0	0	2408.944172	2470.852157	0.924452199	0.854612	0.02464
[TG(50:4)]_C18:1	0	0	0	1690.380117	1732.980123	0.924362343	0.854446	0.02469
PC(29:0),PC(O-30:0)	0	0	0	2140.476163	2173.088159	0.922534669	0.85107	0.02558
[TG(48:3)]_C16:0	0	0	0	1601.964108	1625.788122	0.922466846	0.850945	0.02561

PC(42:0)	0	0	0	2103.676146	2127.540152	0.921805864	0.849726	0.02594
PE(O-38:9),PE(36:2),PE(O-37:2)	0	0	0	2525.71618	2550.528172	0.921518868	0.849197	0.02608
[TG(52:5)]_C20:4	0	0	0	1584.724118	1596.244118	0.921033693	0.848303	0.02632
[TG(49:8)]_C18:2	0	0	0	1101.11608	1107.748075	0.920795598	0.847865	0.02644
PC(44:12),PC(O-44:5)	0	0	0	3297.308218	3307.632211	0.920239753	0.846841	0.02671
[TG(58:7)]_C22:5	0	0	0	1490.236106	1491.728104	0.919827316	0.846082	0.02692
[TG(54:7)]_C20:4	0	0	0	1390.94809	1392.240099	0.919813267	0.846056	0.02693
PC(42:4)	0	0	0	2441.796169	2442.916189	0.919721846	0.845888	0.02697
PS(25:0)	4068.648285	4079.676303	4454.532314	0	0	-0.901014207	0.811827	0.03682
[TG(44:1)]_C16:0	0	0	855.1240601	1450.940102	1183.700078	0.859110148	0.73807	0.06212
[TG(49:7),TG(48:0)]_C14:0	1026.892066	0	0	1873.184132	2274.004153	0.8044146	0.647083	0.10073
[TG(42:0)]_C16:0	967.1040665	0	0	1167.756081	2120.588167	0.784328067	0.615171	0.11627
CE(15:1) NH4	4507.864304	5732.4244	5298.372371	5681.556433	0	-0.718451529	0.516173	0.17156
DG(30:3)_C16:1	22995.78567	31716.21002	28788.1581	29738.68597	0	-0.717300798	0.51452	0.17258
DG(36:8),DG(35:1)_C16:1	19337.31731	28335.74576	22694.26553	26692.66199	0	-0.689418199	0.475297	0.19781
DG(36:7)_C16:1	3791.312303	6277.852449	4662.420339	5476.860374	0	-0.680964233	0.463712	0.20565
DG(30:2)_C16:1	3704.368286	4835.764322	5749.860439	4958.740332	0	-0.663667291	0.440454	0.22195
FA(22:1)	4661.080345	4894.672342	0	3378.228242	0	-0.652015204	0.425124	0.23313
DG(41:6)_C16:1	2949.144208	4463.104312	3442.320248	4511.580306	0	-0.630073907	0.396993	0.25457
FA(19:2)	3968.332299	6190.892452	0	4759.412322	0	-0.519227974	0.269598	0.36994
[TG(40:0)]_C16:0	0	1227.572086	0	1279.016089	1044.112076	0.487633677	0.237787	0.40469
LPG(19:0),LPG(O-20:0); LPG(19	1458.532102	2704.352198	0	3005.320207	0	-0.309828479	0.095994	0.61192
FA(18:0)	23007.93363	35379.32667	0	0	21639.19364	-0.301274071	0.090766	0.62229
DG(32:5)_C18:1	0	1586.268114	0	1603.064102	0	-0.090236435	0.008143	0.88526
[TG(57:10),TG(56:3)]_C18:2	0	0	0	1644.264117	1641.616107	0.919318011	0.845146	0.02718
PC(29:2),PC(P-30:1)	0	0	0	1463.6241	1455.316093	0.918517228	0.843674	0.02758
PC(43:4),PC(O-44:4)	0	0	0	2122.364155	2103.152146	0.917845005	0.842439	0.02792
[TG(58:8)]_C22:6	0	0	0	1641.264119	1618.924116	0.916926465	0.840754	0.02838
PC(41:6),PC(O-42:6)	0	0	0	3154.796227	3111.304207	0.916891057	0.840689	0.0284
[TG(54:7)]_C18:2	0	0	0	2607.344184	2571.096174	0.916867459	0.840646	0.02841
[TG(52:7),TG(51:0)]_C18:0	0	0	0	1271.764093	1253.196085	0.916725642	0.840386	0.02848
LPC(20:2),PC(O-20:2)	0	0	0	2574.732184	2531.892174	0.916309777	0.839624	0.0287
SM(d16:0/25:0)	0	0	0	6578.312467	6452.284457	0.915792335	0.838676	0.02896
[TG(57:12),TG(56:5)]_C18:0	0	0	0	1963.668148	1911.644132	0.914264065	0.835879	0.02974
PC(39:5),PC(O-40:5),PC(P-40:4	0	0	0	12164.54081	11827.06486	0.914000515	0.835397	0.02988
[TG(50:4)]_C16:0	0	0	0	1793.528133	1741.744119	0.913761288	0.83496	0.03
[TG(58:7)]_C18:1	0	0	0	1169.436089	1131.292083	0.912962984	0.833501	0.03042
LPC(22:4)	0	0	0	2289.972162	2213.616154	0.91280717	0.833217	0.0305
PC(40:7),PC(39:0),PC(O-40:0)	0	0	0	6774.536469	6533.012431	0.912309778	0.832309	0.03076
PC(43:6)	0	0	0	2720.040195	2616.932191	0.911820622	0.831417	0.03101
[TG(50:7),TG(49:0)]_C18:0	0	0	0	903.7280632	866.5400574	0.911111648	0.830124	0.03139
[TG(56:8),TG(55:1)]_C16:0	0	0	0	1375.312094	1313.580093	0.910286585	0.828622	0.03182
[TG(50:4)]_C14:0	0	0	0	2559.344193	2436.66816	0.909607239	0.827385	0.03218
[TG(56:8)]_C18:2	0	0	0	1797.36012	1697.256127	0.907849657	0.824191	0.03311
[TG(56:9),TG(55:2)]_C18:1	0	0	0	1154.164078	1083.496075	0.906572958	0.821875	0.0338
PC(42:11),PC(41:4),PC(O-42:4)	0	0	0	4033.016283	3776.400256	0.906013023	0.82086	0.0341
PC(42:7),PC(41:0),PC(O-42:0)	0	0	0	2422.46416	2266.960164	0.905880971	0.82062	0.03417
PC(28:2)	0	0	0	2465.360168	2296.252158	0.90484272	0.81874	0.03473
[TG(54:7)]_C18:3	0	0	0	2243.820158	2065.444154	0.902215173	0.813992	0.03616
Cer(d14:2(4E,6E)/16:0)	2877.7522	3191.112242	2599.740189	2381.628187	0	-0.901134624	0.812044	0.03676
PC(41:5),PC(P-42:4)	0	0	0	5379.128378	4878.720349	0.898842283	0.807917	0.03803
[TG(48:4)]_C18:2	0	0	0	891.8040642	805.1160561	0.897775467	0.806001	0.03863
Cer(d18:1/24:1(15Z))	0	0	0	1946.972141	1730.076118	0.894058428	0.79934	0.04073
CAR(14:2)	0	0	0	2047.740146	1812.628128	0.89314324	0.797705	0.04125
[TG(52:7),TG(51:0)]_C16:0	0	0	0	1683.084114	1468.992104	0.889751462	0.791658	0.04321
PC(19:1),LPC(20:1),PC(O-20:1)	0	0	0	1803.89613	1555.732108	0.886825451	0.786459	0.04492
SM(d18:1/12:0)	0	0	0	2070.204151	1770.372123	0.884728582	0.782745	0.04616
[TG(57:10),TG(56:3)]_C18:0	0	0	0	1645.460119	1395.74409	0.882690444	0.779142	0.04737
[TG(58:8)]_C22:5	0	0	0	1426.224097	1148.724085	0.86926476	0.755621	0.05562
CAR(14:1)	0	0	0	3630.208249	2913.480212	0.868313764	0.753969	0.05622
PC(36:6)	0	0	0	2121.928147	1694.272126	0.866938627	0.751583	0.05709
PC(32:3),PC(P-33:2)	0	0	0	2205.704157	1744.392117	0.864355629	0.747111	0.05873
PC(42:1)	0	0	0	1407.264099	1096.74808	0.860354412	0.74021	0.06131
[TG(38:0)]_C18:0	0	0	0	1047.340073	788.3720573	0.850667449	0.723635	0.0677
FA(35:0)	2747.764197	3373.412244	3291.244229	2653.08418	0	-0.836091883	0.69905	0.07767
[TG(52:6)]_C16:1	0	0	0	1111.464083	790.9800552	0.834481827	0.69636	0.0788
FA(22:0)	15692.86104	29835.51397	11183.10078	0	0	-0.834405858	0.696233	0.07885
[TG(58:8),TG(57:1)]_C18:1	0	0	0	1160.308083	783.4240509	0.818812025	0.670453	0.09002

DG(36:6)_C16:0	24635.4179	34573.36243	31662.96218	25464.20162	0	-0.815856516	0.665622	0.09219
FA(20:0)	25507.74568	50627.56784	15739.76514	0	0	-0.812791576	0.66063	0.09446
DG(41:5)_C16:0	15042.41718	22595.78553	17843.67744	16422.52514	0	-0.805360903	0.648606	0.10002
PC(44:0)	0	0	0	0	1347.096092	0.801783726	0.642857	0.10273
CE(18:0)K	0	0	0	0	47762.4673	0.801783726	0.642857	0.10273
CE(20:3)Na	0	0	0	0	2535.096189	0.801783726	0.642857	0.10273
[TG(51:6)]_C18:0	0	0	0	0	926.1520653	0.801783726	0.642857	0.10273
CE(20:4) NH4	0	0	0	0	227021.7404	0.801783726	0.642857	0.10273
CE(16:1) NH4	0	0	0	0	19961.96537	0.801783726	0.642857	0.10273
CE(20:3) NH4	0	0	0	0	17452.29311	0.801783726	0.642857	0.10273
CE(22:6)H	0	0	0	0	3372.820226	0.801783726	0.642857	0.10273
DG(40:9),DG(39:2)_C18:2	0	0	0	0	2554.956181	0.801783726	0.642857	0.10273
PE(O-38:8),PE(36:1),PE(O-37:1)	0	0	0	0	1469.264104	0.801783726	0.642857	0.10273
[TG(54:7),TG(53:0)]_C18:0	0	0	0	0	1221.616089	0.801783726	0.642857	0.10273
[TG(52:9),TG(51:2)]_C16:1	0	0	0	0	1099.864075	0.801783726	0.642857	0.10273
[TG(55:11),TG(54:4)]_C16:1	0	0	0	0	851.7040604	0.801783726	0.642857	0.10273
DG(39:7)_C18:1	0	0	0	0	8312.468564	0.801783726	0.642857	0.10273
DG(O-40:9),DG(38:2)_C18:1	0	0	0	0	4205.776298	0.801783726	0.642857	0.10273
[TG(44:0),TG(O-45:0)]_C14:0	0	0	0	0	1029.528075	0.801783726	0.642857	0.10273
LPE(22:4)	0	0	0	0	945.5440704	0.801783726	0.642857	0.10273
[TG(56:8),TG(55:1)]_C18:1	0	0	0	0	819.276059	0.801783726	0.642857	0.10273
CE(18:3)Na	0	0	0	0	23460.31366	0.801783726	0.642857	0.10273
CE(20:0)H	0	0	0	0	2825.600198	0.801783726	0.642857	0.10273
DG(O-38:8),DG(36:1)_C16:1	0	0	0	0	11697.01289	0.801783726	0.642857	0.10273
PE(34:1),PE(O-35:1),PE(P-35:0)	0	0	0	0	1936.988143	0.801783726	0.642857	0.10273
[TG(46:2)]_C16:0	0	0	0	0	1625.888109	0.801783726	0.642857	0.10273
[TG(48:7),TG(47:0)]_C16:0	0	0	0	0	1615.904112	0.801783726	0.642857	0.10273
[TG(51:6)]_C16:0	0	0	0	0	1215.456081	0.801783726	0.642857	0.10273
[TG(54:5)]_C16:1	0	0	0	0	1097.464076	0.801783726	0.642857	0.10273
Cer(d18:1/16:0)	0	0	0	0	1075.624078	0.801783726	0.642857	0.10273
[TG(52:6)]_C16:0	0	0	0	0	1044.368084	0.801783726	0.642857	0.10273
[TG(46:3)]_C18:1	0	0	0	0	866.0360634	0.801783726	0.642857	0.10273
[TG(46:1)]_C16:1	0	0	0	0	1717.496128	0.801783726	0.642857	0.10273
PE(36:4),PE(O-37:4)	0	0	0	0	1717.216113	0.801783726	0.642857	0.10273
CE(20:1)H	0	0	0	0	1694.020125	0.801783726	0.642857	0.10273
PS(P-37:0)	0	0	0	0	1667.180115	0.801783726	0.642857	0.10273
LPC(20:3)	0	0	0	0	1325.832103	0.801783726	0.642857	0.10273
PE(40:6),PE(dO-40:0)	0	0	0	0	1260.65609	0.801783726	0.642857	0.10273
[TG(48:3)]_C14:0	0	0	0	0	1147.632082	0.801783726	0.642857	0.10273
PE(38:5)	0	0	0	0	1135.18008	0.801783726	0.642857	0.10273
[TG(49:7)]_C16:1	0	0	0	0	1059.640071	0.801783726	0.642857	0.10273
[TG(55:10),TG(54:3)]_C20:0	0	0	0	0	827.764061	0.801783726	0.642857	0.10273
CE(22:6)Na	0	0	0	0	2680.80419	0.801783726	0.642857	0.10273
[TG(44:0),TG(O-45:0)]_C18:0	0	0	0	0	805.4080574	0.801783726	0.642857	0.10273
FA(22:7)	25089.74174	48989.01946	15381.52106	15001.06098	0	-0.801134367	0.641816	0.10322
[TG(59:13),TG(58:6)]_C18:1	0	0	0	1397.860101	833.3360558	0.78045932	0.609117	0.11933
DG(36:7),DG(35:0)_C16:0	47379.18316	73095.69286	63799.70829	54445.26785	0	-0.779524439	0.607658	0.12008
DG(30:2)_C16:0	25652.06181	32017.02221	29789.39792	28791.27802	0	-0.778560523	0.606156	0.12085
DG(42:11),DG(41:4)_C16:0	3952.680283	6432.780463	5612.360384	4762.816343	0	-0.760233481	0.577955	0.13575
FA(21:0)	21014.08549	52706.07187	16135.97716	15714.01709	0	-0.733513292	0.538042	0.15837
CAR(20:0)	1884.576141	3302.356226	3216.708241	2495.488175	0	-0.709285942	0.503087	0.17973
FA(26:6)	0	6100.516441	0	0	0	-0.43907204	0.192784	0.45948
FA(28:0)	0	4073.500296	0	0	0	-0.43907204	0.192784	0.45948
FA(19:0)	0	26949.05388	0	0	0	-0.43907204	0.192784	0.45948
FA(21:1)	0	4411.408311	0	0	0	-0.43907204	0.192784	0.45948
PG(20:0),LPG(21:0); PG(20:0),L	0	1821.980128	0	0	0	-0.43907204	0.192784	0.45948
FA(17:2)	0	5931.444425	0	0	0	-0.43907204	0.192784	0.45948
FA(16:0)	0	30468.89427	0	0	0	-0.43907204	0.192784	0.45948
FA(6:0)	0	13603.70898	0	0	0	-0.43907204	0.192784	0.45948
Cer(d18:0/17:0)	2757.436208	0	2470.576183	2034.240148	0	-0.394359969	0.15552	0.51122
[TG(56:11),TG(55:4)]_C18:2	0	0	0	1352.924092	0	0.324531508	0.105321	0.59417
DG(36:8),DG(35:1)_C18:1	0	0	0	2528.636182	0	0.324531508	0.105321	0.59417
FA(24:4)	0	0	0	6956.416477	0	0.324531508	0.105321	0.59417
[TG(42:0)]_C14:0	0	0	0	1253.872089	0	0.324531508	0.105321	0.59417
[TG(57:11),TG(56:4)]_C16:0	0	0	0	843.8280617	0	0.324531508	0.105321	0.59417
[TG(42:1)]_C18:1	0	0	0	752.2760582	0	0.324531508	0.105321	0.59417
[TG(53:10),TG(52:3)]_C20:0	0	0	0	836.1040619	0	0.324531508	0.105321	0.59417
PC(30:3)	0	0	0	1038.28007	0	0.324531508	0.105321	0.59417

PI(36:1),PI(O-37:1),PI(P-37:0)	0	0	0	1042.34007	0	0.324531508	0.105321	0.59417
[TG(51:7),TG(50:0)]_C14:0	0	0	0	818.868055	0	0.324531508	0.105321	0.59417
DG(36:5)_C16:0	0	2832.492194	2466.964168	2063.332154	0	-0.310034306	0.096121	0.61167
FA(30:0)	0	0	3601.70425	0	0	-0.15272071	0.023324	0.80631
[TG(45:2)]_C16:0	0	0	776.7680542	0	0	-0.15272071	0.023324	0.80631
FA(37:0)	0	0	2941.772214	0	0	-0.15272071	0.023324	0.80631
DG(30:1)_C16:0	0	0	5166.144346	5034.964319	0	0.135253312	0.018293	0.82832
DG(36:6)_C16:1	0	0	1783.40013	1678.556123	0	0.128383693	0.016482	0.83699
FA(28:6)	4454.876311	4405.728316	0	0	0	-0.795348498	0.632579	0.10766
FA(18:3)	1981.208124	0	0	0	0	-0.534522484	0.285714	0.35339
CAR(18:3)	2487.54817	0	0	0	0	-0.534522484	0.285714	0.35339
DG(34:1)_C16:1	0	0	0	0	0	#DIV/0!	#DIV/0!	#DIV/0!
LPI(19:0),LPI(O-20:0)	0	0	0	0	0	#DIV/0!	#DIV/0!	#DIV/0!
DG(41:6)_C18:1	0	0	0	0	0	#DIV/0!	#DIV/0!	#DIV/0!
CE(15:0) NH4	0	0	0	0	0	#DIV/0!	#DIV/0!	#DIV/0!
[TG(48:8),TG(47:1)]_C16:1	0	0	0	0	0	#DIV/0!	#DIV/0!	#DIV/0!
[TG(48:8),TG(47:1)]_C16:0	0	0	0	0	0	#DIV/0!	#DIV/0!	#DIV/0!
PC(31:3),PC(O-32:3)	0	0	0	0	0	#DIV/0!	#DIV/0!	#DIV/0!
[TG(58:8)]_C18:2	0	0	0	0	0	#DIV/0!	#DIV/0!	#DIV/0!
[TG(45:3)]_C16:0	0	0	0	0	0	#DIV/0!	#DIV/0!	#DIV/0!
CE(20:1)Na	0	0	0	0	0	#DIV/0!	#DIV/0!	#DIV/0!
CE(16:0)H	0	0	0	0	0	#DIV/0!	#DIV/0!	#DIV/0!
FA(15:1)	0	0	0	0	0	#DIV/0!	#DIV/0!	#DIV/0!
FA(14:2)	0	0	0	0	0	#DIV/0!	#DIV/0!	#DIV/0!
FA(10:3)	0	0	0	0	0	#DIV/0!	#DIV/0!	#DIV/0!
[TG(50:9),TG(49:2)]_C16:1	0	0	0	0	0	#DIV/0!	#DIV/0!	#DIV/0!
DG(41:5)_C16:1	0	0	0	0	0	#DIV/0!	#DIV/0!	#DIV/0!
CE(22:4)H	0	0	0	0	0	#DIV/0!	#DIV/0!	#DIV/0!
LPG(18:0); LPG(18:0)	0	0	0	0	0	#DIV/0!	#DIV/0!	#DIV/0!
[TG(46:2)]_C16:1	0	0	0	0	0	#DIV/0!	#DIV/0!	#DIV/0!
[TG(48:8),TG(47:1)]_C18:1	0	0	0	0	0	#DIV/0!	#DIV/0!	#DIV/0!
CE(22:2) NH4	0	0	0	0	0	#DIV/0!	#DIV/0!	#DIV/0!
CE(22:4)Na	0	0	0	0	0	#DIV/0!	#DIV/0!	#DIV/0!
CE(17:0)Na	0	0	0	0	0	#DIV/0!	#DIV/0!	#DIV/0!
PC(44:1)	0	0	0	0	0	#DIV/0!	#DIV/0!	#DIV/0!
[TG(46:3)]_C18:2	0	0	0	0	0	#DIV/0!	#DIV/0!	#DIV/0!
[TG(58:9),TG(57:2)]_C18:1	0	0	0	0	0	#DIV/0!	#DIV/0!	#DIV/0!
[TG(57:8),TG(56:1)]_C18:0	0	0	0	0	0	#DIV/0!	#DIV/0!	#DIV/0!
[TG(49:3)]_C16:0	0	0	0	0	0	#DIV/0!	#DIV/0!	#DIV/0!
PI(34:2),PI(O-35:2),PI(P-35:1)	0	0	0	0	0	#DIV/0!	#DIV/0!	#DIV/0!
[TG(52:10),TG(51:3)]_C16:1	0	0	0	0	0	#DIV/0!	#DIV/0!	#DIV/0!
CE(24:1) NH4	0	0	0	0	0	#DIV/0!	#DIV/0!	#DIV/0!
1-O-pentacosanoyl-Cer(d18:1/1	0	0	0	0	0	#DIV/0!	#DIV/0!	#DIV/0!
[TG(50:9),TG(49:2)]_C20:0	0	0	0	0	0	#DIV/0!	#DIV/0!	#DIV/0!
[TG(47:6)]_C16:0	0	0	0	0	0	#DIV/0!	#DIV/0!	#DIV/0!
[TG(53:9),TG(52:2)]_C20:0	0	0	0	0	0	#DIV/0!	#DIV/0!	#DIV/0!
[TG(50:5)]_C18:3	0	0	0	0	0	#DIV/0!	#DIV/0!	#DIV/0!
CE(24:1)Na	0	0	0	0	0	#DIV/0!	#DIV/0!	#DIV/0!
CAR(20:1)	0	0	0	0	0	#DIV/0!	#DIV/0!	#DIV/0!
[TG(50:8),TG(49:1)]_C16:1	0	0	0	0	0	#DIV/0!	#DIV/0!	#DIV/0!
PG(25:0); PG(25:0)	0	0	0	0	0	#DIV/0!	#DIV/0!	#DIV/0!
[TG(45:0)]_C16:0	0	0	0	0	0	#DIV/0!	#DIV/0!	#DIV/0!
[TG(57:8),TG(56:1)]_C18:1	0	0	0	0	0	#DIV/0!	#DIV/0!	#DIV/0!
PG(O-35:1),PG(P-35:0); PG(O-3	0	0	0	0	0	#DIV/0!	#DIV/0!	#DIV/0!
[TG(54:7)]_C18:1	0	0	0	0	0	#DIV/0!	#DIV/0!	#DIV/0!
PE(37:5),PE(O-38:5),PE(P-38:4)	0	0	0	0	0	#DIV/0!	#DIV/0!	#DIV/0!
PC(44:3)	0	0	0	0	0	#DIV/0!	#DIV/0!	#DIV/0!
PE(38:3)	0	0	0	0	0	#DIV/0!	#DIV/0!	#DIV/0!
DG(32:2)_C18:1	0	0	0	0	0	#DIV/0!	#DIV/0!	#DIV/0!
FA(34:6)	0	0	0	0	0	#DIV/0!	#DIV/0!	#DIV/0!
[TG(42:0)]_C18:0	0	0	0	0	0	#DIV/0!	#DIV/0!	#DIV/0!
FA(23:0)	0	0	0	0	0	#DIV/0!	#DIV/0!	#DIV/0!
[TG(55:7),TG(54:0)]_C16:0	0	0	0	0	0	#DIV/0!	#DIV/0!	#DIV/0!
CE(18:3) NH4	0	0	0	0	0	#DIV/0!	#DIV/0!	#DIV/0!
DG(37:7)_C16:1	0	0	0	0	0	#DIV/0!	#DIV/0!	#DIV/0!
DG(34:2)_C16:1	0	0	0	0	0	#DIV/0!	#DIV/0!	#DIV/0!
CE(20:0)K	0	0	0	0	0	#DIV/0!	#DIV/0!	#DIV/0!
PC(44:2)	0	0	0	0	0	#DIV/0!	#DIV/0!	#DIV/0!

[TG(59:10),TG(58:3)]_C18:2	0	0	0	0	0	#DIV/0!	#DIV/0!	#DIV/0!
[TG(44:2)]_C18:2	0	0	0	0	0	#DIV/0!	#DIV/0!	#DIV/0!
[TG(50:8),TG(49:1)]_C14:0	0	0	0	0	0	#DIV/0!	#DIV/0!	#DIV/0!
[TG(52:5)]_C22:5	0	0	0	0	0	#DIV/0!	#DIV/0!	#DIV/0!
PI(38:5)	0	0	0	0	0	#DIV/0!	#DIV/0!	#DIV/0!
[TG(52:9),TG(51:2)]_C18:0	0	0	0	0	0	#DIV/0!	#DIV/0!	#DIV/0!
[TG(52:7)]_C18:1	0	0	0	0	0	#DIV/0!	#DIV/0!	#DIV/0!
LPC(14:0),PC(O-14:0),LPC(O-1!	0	0	0	0	0	#DIV/0!	#DIV/0!	#DIV/0!
CAR(18:2)	0	0	0	0	0	#DIV/0!	#DIV/0!	#DIV/0!
[TG(45:1)]_C16:1	0	0	0	0	0	#DIV/0!	#DIV/0!	#DIV/0!
FA(26:2)	0	0	0	0	0	#DIV/0!	#DIV/0!	#DIV/0!
Cer(d18:0/21:0)	0	0	0	0	0	#DIV/0!	#DIV/0!	#DIV/0!
DG(41:6)_C16:0	0	0	0	0	0	#DIV/0!	#DIV/0!	#DIV/0!
LPS(P-16:0)	0	0	0	0	0	#DIV/0!	#DIV/0!	#DIV/0!
[TG(45:4)]_C20:0	0	0	0	0	0	#DIV/0!	#DIV/0!	#DIV/0!
DG(30:1)_C16:1	0	0	0	0	0	#DIV/0!	#DIV/0!	#DIV/0!
DG(41:5)_C18:0	0	0	0	0	0	#DIV/0!	#DIV/0!	#DIV/0!
DG(35:3)_C18:0	0	0	0	0	0	#DIV/0!	#DIV/0!	#DIV/0!
FA(34:0)	0	0	0	0	0	#DIV/0!	#DIV/0!	#DIV/0!
[TG(47:2)]_C16:1	0	0	0	0	0	#DIV/0!	#DIV/0!	#DIV/0!
[TG(44:1)]_C14:0	0	0	0	0	0	#DIV/0!	#DIV/0!	#DIV/0!
CE(20:5) NH4	0	0	0	0	0	#DIV/0!	#DIV/0!	#DIV/0!
CE(18:0) NH4	0	0	0	0	0	#DIV/0!	#DIV/0!	#DIV/0!
CE(18:2)K	0	0	0	0	0	#DIV/0!	#DIV/0!	#DIV/0!
CE(19:0)Na	0	0	0	0	0	#DIV/0!	#DIV/0!	#DIV/0!
CE(20:5)Na	0	0	0	0	0	#DIV/0!	#DIV/0!	#DIV/0!
CE(22:3) NH4	0	0	0	0	0	#DIV/0!	#DIV/0!	#DIV/0!
DG(40:2)_C18:2	0	0	0	0	0	#DIV/0!	#DIV/0!	#DIV/0!
CE(22:4) NH4	0	0	0	0	0	#DIV/0!	#DIV/0!	#DIV/0!
PC(27:0),PC(O-28:0)	0	0	0	0	0	#DIV/0!	#DIV/0!	#DIV/0!
[TG(51:7)]_C16:1	0	0	0	0	0	#DIV/0!	#DIV/0!	#DIV/0!
[TG(38:0)]_C20:0	0	0	0	0	0	#DIV/0!	#DIV/0!	#DIV/0!
[TG(57:9),TG(56:2)]_C18:2	0	0	0	0	0	#DIV/0!	#DIV/0!	#DIV/0!
[TG(57:8),TG(56:1)]_C16:0	0	0	0	0	0	#DIV/0!	#DIV/0!	#DIV/0!
[TG(57:9),TG(56:2)]_C16:0	0	0	0	0	0	#DIV/0!	#DIV/0!	#DIV/0!
PC(19:0),LPC(20:0),PC(O-20:0)	0	0	0	0	0	#DIV/0!	#DIV/0!	#DIV/0!
[TG(46:1)]_C18:0	0	0	0	0	0	#DIV/0!	#DIV/0!	#DIV/0!
[TG(48:7),TG(47:0)]_C14:0	0	0	0	0	0	#DIV/0!	#DIV/0!	#DIV/0!
[TG(56:8)]_C18:3	0	0	0	0	0	#DIV/0!	#DIV/0!	#DIV/0!
[TG(48:4)]_C18:1	0	0	0	0	0	#DIV/0!	#DIV/0!	#DIV/0!
[TG(48:6)]_C16:0	0	0	0	0	0	#DIV/0!	#DIV/0!	#DIV/0!
CE(14:1) NH4	0	0	0	0	0	#DIV/0!	#DIV/0!	#DIV/0!
[TG(48:4)]_C18:3	0	0	0	0	0	#DIV/0!	#DIV/0!	#DIV/0!
[TG(39:0)]_C16:0	0	0	0	0	0	#DIV/0!	#DIV/0!	#DIV/0!
[TG(56:13),TG(55:6)]_C18:2	0	0	0	0	0	#DIV/0!	#DIV/0!	#DIV/0!
FA(38:6)	0	0	0	0	0	#DIV/0!	#DIV/0!	#DIV/0!
[TG(48:6)]_C18:0	0	0	0	0	0	#DIV/0!	#DIV/0!	#DIV/0!
PI(38:2),PI(P-39:1)	0	0	0	0	0	#DIV/0!	#DIV/0!	#DIV/0!
DG(36:8),DG(35:1)_C18:0	0	0	0	0	0	#DIV/0!	#DIV/0!	#DIV/0!
PC(44:7),PC(43:0)	0	0	0	0	0	#DIV/0!	#DIV/0!	#DIV/0!
[TG(37:0)]_C14:0	0	0	0	0	0	#DIV/0!	#DIV/0!	#DIV/0!
[TG(55:10),TG(54:3)]_C16:1	0	0	0	0	0	#DIV/0!	#DIV/0!	#DIV/0!
FA(10:5)	0	0	0	0	0	#DIV/0!	#DIV/0!	#DIV/0!
FA(32:0)	0	0	0	0	0	#DIV/0!	#DIV/0!	#DIV/0!
[TG(58:9)]_C20:4	0	0	0	0	0	#DIV/0!	#DIV/0!	#DIV/0!
[TG(54:6)]_C16:1	0	0	0	0	0	#DIV/0!	#DIV/0!	#DIV/0!
PC(44:4)	0	0	0	0	0	#DIV/0!	#DIV/0!	#DIV/0!
FA(18:2)	0	0	0	0	0	#DIV/0!	#DIV/0!	#DIV/0!
CAR(10:2)	0	0	0	0	0	#DIV/0!	#DIV/0!	#DIV/0!
FA(21:2)	0	0	0	0	0	#DIV/0!	#DIV/0!	#DIV/0!
FA(30:2)	0	0	0	0	0	#DIV/0!	#DIV/0!	#DIV/0!
CAR(26:0)	0	0	0	0	0	#DIV/0!	#DIV/0!	#DIV/0!
DG(37:7)_C18:1	0	0	0	0	0	#DIV/0!	#DIV/0!	#DIV/0!
DG(30:2)_C18:1	0	0	0	0	0	#DIV/0!	#DIV/0!	#DIV/0!
FA(24:0)	0	0	0	0	0	#DIV/0!	#DIV/0!	#DIV/0!
FA(24:2)	0	0	0	0	0	#DIV/0!	#DIV/0!	#DIV/0!
FA(20:6)	0	0	0	0	0	#DIV/0!	#DIV/0!	#DIV/0!
FA(24:1)	0	0	0	0	0	#DIV/0!	#DIV/0!	#DIV/0!

PG(24:0); PG(24:0)	0	0	0	0	0	#DIV/0!	#DIV/0!	#DIV/0!
[TG(40:0)]_C18:0	0	0	0	0	0	#DIV/0!	#DIV/0!	#DIV/0!
DG(39:7),DG(38:0),DG(dO-40:0	0	0	0	0	0	#DIV/0!	#DIV/0!	#DIV/0!
CE(18:1)K	0	0	0	0	0	#DIV/0!	#DIV/0!	#DIV/0!
CE(20:2) NH4	0	0	0	0	0	#DIV/0!	#DIV/0!	#DIV/0!
DG(37:6)_C16:0	0	0	0	0	0	#DIV/0!	#DIV/0!	#DIV/0!
LPE(20:4)	0	0	0	0	0	#DIV/0!	#DIV/0!	#DIV/0!
LPC(22:6)	0	0	0	0	0	#DIV/0!	#DIV/0!	#DIV/0!
PC(26:1)	0	0	0	0	0	#DIV/0!	#DIV/0!	#DIV/0!
CE(24:1)K	0	0	0	0	0	#DIV/0!	#DIV/0!	#DIV/0!
[TG(44:5)]_C20:0	0	0	0	0	0	#DIV/0!	#DIV/0!	#DIV/0!
[TG(59:10),TG(58:3)]_C18:1	0	0	0	0	0	#DIV/0!	#DIV/0!	#DIV/0!
[TG(46:2)]_C14:0	0	0	0	0	0	#DIV/0!	#DIV/0!	#DIV/0!
[TG(50:4)]_C20:4	0	0	0	0	0	#DIV/0!	#DIV/0!	#DIV/0!
[TG(44:2)]_C18:1	0	0	0	0	0	#DIV/0!	#DIV/0!	#DIV/0!
[TG(59:9),TG(58:2)]_C18:2	0	0	0	0	0	#DIV/0!	#DIV/0!	#DIV/0!
PI(36:4)	0	0	0	0	0	#DIV/0!	#DIV/0!	#DIV/0!
[TG(52:4)]_C18:0	0	0	0	0	0	#DIV/0!	#DIV/0!	#DIV/0!
[TG(53:9),TG(52:2)]_C14:0	0	0	0	0	0	#DIV/0!	#DIV/0!	#DIV/0!
PC(44:8),PC(43:1)	0	0	0	0	0	#DIV/0!	#DIV/0!	#DIV/0!
[TG(38:1)]_C16:0	0	0	0	0	0	#DIV/0!	#DIV/0!	#DIV/0!
DG(40:7),DG(39:0)_C18:0	0	0	0	0	0	#DIV/0!	#DIV/0!	#DIV/0!
LPG(15:0),LPG(O-16:0); LPG(15	0	0	0	0	0	#DIV/0!	#DIV/0!	#DIV/0!
[TG(46:6)]_C16:0	0	0	0	0	0	#DIV/0!	#DIV/0!	#DIV/0!
FA(22:5)	0	0	0	0	0	#DIV/0!	#DIV/0!	#DIV/0!
[TG(51:8)]_C22:5	0	0	0	0	0	#DIV/0!	#DIV/0!	#DIV/0!
FA(32:5)	0	0	0	0	0	#DIV/0!	#DIV/0!	#DIV/0!
FA(30:4)	0	0	0	0	0	#DIV/0!	#DIV/0!	#DIV/0!
FA(10:2)	0	0	0	0	0	#DIV/0!	#DIV/0!	#DIV/0!
FA(23:1)	0	0	0	0	0	#DIV/0!	#DIV/0!	#DIV/0!
FA(20:2)	0	0	0	0	0	#DIV/0!	#DIV/0!	#DIV/0!
CAR(22:5)	0	0	0	0	0	#DIV/0!	#DIV/0!	#DIV/0!
[TG(62:14),TG(61:7),TG(60:0)]_(	0	0	0	0	0	#DIV/0!	#DIV/0!	#DIV/0!
CAR(20:2)	0	0	0	0	0	#DIV/0!	#DIV/0!	#DIV/0!
[TG(37:0)]_C16:0	0	0	0	0	0	#DIV/0!	#DIV/0!	#DIV/0!
DG(32:1)_C18:1	0	0	0	0	0	#DIV/0!	#DIV/0!	#DIV/0!
DG(33:1),DG(O-34:1)_C16:1	0	0	0	0	0	#DIV/0!	#DIV/0!	#DIV/0!
DG(33:1),DG(O-34:1)_C18:1	0	0	0	0	0	#DIV/0!	#DIV/0!	#DIV/0!
DG(36:7)_C18:1	0	0	0	0	0	#DIV/0!	#DIV/0!	#DIV/0!
FA(19:1)	0	0	0	0	0	#DIV/0!	#DIV/0!	#DIV/0!
LPI(16:0)	0	0	0	0	0	#DIV/0!	#DIV/0!	#DIV/0!
[TG(41:0)]_C16:0	0	0	0	0	0	#DIV/0!	#DIV/0!	#DIV/0!
LPE(18:1)	0	0	0	0	0	#DIV/0!	#DIV/0!	#DIV/0!
FA(25:0)	0	0	0	0	0	#DIV/0!	#DIV/0!	#DIV/0!
FA(6:2)	0	0	0	0	0	#DIV/0!	#DIV/0!	#DIV/0!
FA(33:0)	0	0	0	0	0	#DIV/0!	#DIV/0!	#DIV/0!
DG(30:1)_C18:1	0	0	0	0	0	#DIV/0!	#DIV/0!	#DIV/0!
CAR(18:0)	0	0	0	0	0	#DIV/0!	#DIV/0!	#DIV/0!
LPG(16:0); LPG(16:0)	0	0	0	0	0	#DIV/0!	#DIV/0!	#DIV/0!
FA(21:5)	0	0	0	0	0	#DIV/0!	#DIV/0!	#DIV/0!
FA(22:6)	0	0	0	0	0	#DIV/0!	#DIV/0!	#DIV/0!
FA(26:4)	0	0	0	0	0	#DIV/0!	#DIV/0!	#DIV/0!
FA(19:6)	0	0	0	0	0	#DIV/0!	#DIV/0!	#DIV/0!
DG(24:0)_C18:0	0	0	0	0	0	#DIV/0!	#DIV/0!	#DIV/0!
[TG(57:8)]_C18:2	0	0	0	0	0	#DIV/0!	#DIV/0!	#DIV/0!
FA(20:1)	0	0	0	0	0	#DIV/0!	#DIV/0!	#DIV/0!
FA(18:1)	0	0	0	0	0	#DIV/0!	#DIV/0!	#DIV/0!
CAR(22:0)	0	0	0	0	0	#DIV/0!	#DIV/0!	#DIV/0!
CAR(5:1)	0	0	0	0	0	#DIV/0!	#DIV/0!	#DIV/0!
FA(22:2)	0	0	0	0	0	#DIV/0!	#DIV/0!	#DIV/0!
CAR(10:3)	0	0	0	0	0	#DIV/0!	#DIV/0!	#DIV/0!
FA(36:6)	0	0	0	0	0	#DIV/0!	#DIV/0!	#DIV/0!
[TG(43:1)]_C16:1	0	0	0	0	0	#DIV/0!	#DIV/0!	#DIV/0!
FA(16:6)	0	0	0	0	0	#DIV/0!	#DIV/0!	#DIV/0!
[TG(46:3)]_C16:1	0	0	0	0	0	#DIV/0!	#DIV/0!	#DIV/0!
[TG(58:14),TG(57:7),TG(56:0)]_(	0	0	0	0	0	#DIV/0!	#DIV/0!	#DIV/0!
FA(27:3)	0	0	0	0	0	#DIV/0!	#DIV/0!	#DIV/0!
FA(36:4)	0	0	0	0	0	#DIV/0!	#DIV/0!	#DIV/0!

FA(19:5)	0	0	0	0	0	#DIV/0!	#DIV/0!	#DIV/0!
FA(24:5)	0	0	0	0	0	#DIV/0!	#DIV/0!	#DIV/0!
CAR(14:0)	0	0	0	0	0	#DIV/0!	#DIV/0!	#DIV/0!
[TG(45:2)]_C16:1	0	0	0	0	0	#DIV/0!	#DIV/0!	#DIV/0!
DG(33:3)_C16:0	0	0	0	0	0	#DIV/0!	#DIV/0!	#DIV/0!
FA(9:0)	0	0	0	0	0	#DIV/0!	#DIV/0!	#DIV/0!
FA(14:6)	0	0	0	0	0	#DIV/0!	#DIV/0!	#DIV/0!
FA(18:6)	0	0	0	0	0	#DIV/0!	#DIV/0!	#DIV/0!
FA(18:5)	0	0	0	0	0	#DIV/0!	#DIV/0!	#DIV/0!
FA(20:3)	0	0	0	0	0	#DIV/0!	#DIV/0!	#DIV/0!
DG(32:0)_C18:0	0	0	0	0	0	#DIV/0!	#DIV/0!	#DIV/0!
DG(32:0)_C16:0	0	0	0	0	0	#DIV/0!	#DIV/0!	#DIV/0!
DG(33:0)_C16:0	0	0	0	0	0	#DIV/0!	#DIV/0!	#DIV/0!
DG(34:4),DG(dO-36:4)_C16:1	0	0	0	0	0	#DIV/0!	#DIV/0!	#DIV/0!
DG(34:0)_C18:0	0	0	0	0	0	#DIV/0!	#DIV/0!	#DIV/0!
DG(34:0)_C16:0	0	0	0	0	0	#DIV/0!	#DIV/0!	#DIV/0!
DG(35:6)_C18:0	0	0	0	0	0	#DIV/0!	#DIV/0!	#DIV/0!
DG(35:6)_C16:0	0	0	0	0	0	#DIV/0!	#DIV/0!	#DIV/0!
DG(37:7),DG(36:0)_C18:0	0	0	0	0	0	#DIV/0!	#DIV/0!	#DIV/0!
DG(37:7),DG(36:0)_C16:0	0	0	0	0	0	#DIV/0!	#DIV/0!	#DIV/0!
CE(16:0) NH4	0	0	0	0	0	#DIV/0!	#DIV/0!	#DIV/0!
DG(37:6)_C18:0	0	0	0	0	0	#DIV/0!	#DIV/0!	#DIV/0!
CE(18:2)Na	0	0	0	0	0	#DIV/0!	#DIV/0!	#DIV/0!
CE(20:5)H	0	0	0	0	0	#DIV/0!	#DIV/0!	#DIV/0!
DG(34:3)_C18:1	0	0	0	0	0	#DIV/0!	#DIV/0!	#DIV/0!
DG(30:0)_C16:0	0	0	0	0	0	#DIV/0!	#DIV/0!	#DIV/0!
DG(40:5)_C16:0	0	0	0	0	0	#DIV/0!	#DIV/0!	#DIV/0!
CE(16:0)Na	0	0	0	0	0	#DIV/0!	#DIV/0!	#DIV/0!
DG(34:1)_C16:0	0	0	0	0	0	#DIV/0!	#DIV/0!	#DIV/0!
DG(34:2)_C18:2	0	0	0	0	0	#DIV/0!	#DIV/0!	#DIV/0!
DG(O-38:9),DG(36:2)_C18:1	0	0	0	0	0	#DIV/0!	#DIV/0!	#DIV/0!
CE(18:3)H	0	0	0	0	0	#DIV/0!	#DIV/0!	#DIV/0!
CE(15:1)K	0	0	0	0	0	#DIV/0!	#DIV/0!	#DIV/0!
CE(22:1)H	0	0	0	0	0	#DIV/0!	#DIV/0!	#DIV/0!
DG(38:5)_C16:0	0	0	0	0	0	#DIV/0!	#DIV/0!	#DIV/0!
DG(34:1)_C18:1	0	0	0	0	0	#DIV/0!	#DIV/0!	#DIV/0!
DG(36:3)_C18:1	0	0	0	0	0	#DIV/0!	#DIV/0!	#DIV/0!
DG(36:4),DG(O-37:4)_C18:2	0	0	0	0	0	#DIV/0!	#DIV/0!	#DIV/0!
CE(16:1)Na	0	0	0	0	0	#DIV/0!	#DIV/0!	#DIV/0!
DG(36:3)_C18:2	0	0	0	0	0	#DIV/0!	#DIV/0!	#DIV/0!
CE(18:1)Na	0	0	0	0	0	#DIV/0!	#DIV/0!	#DIV/0!
DG(34:2)_C16:0	0	0	0	0	0	#DIV/0!	#DIV/0!	#DIV/0!
CE(20:4)H	0	0	0	0	0	#DIV/0!	#DIV/0!	#DIV/0!
DG(38:3)_C18:2	0	0	0	0	0	#DIV/0!	#DIV/0!	#DIV/0!
CE(19:0) NH4	0	0	0	0	0	#DIV/0!	#DIV/0!	#DIV/0!
CE(16:2)Na	0	0	0	0	0	#DIV/0!	#DIV/0!	#DIV/0!
DG(O-38:8),DG(36:1)_C18:1	0	0	0	0	0	#DIV/0!	#DIV/0!	#DIV/0!
CE(20:0)Na	0	0	0	0	0	#DIV/0!	#DIV/0!	#DIV/0!
CE(22:1) NH4	0	0	0	0	0	#DIV/0!	#DIV/0!	#DIV/0!
DG(42:5)_C18:0	0	0	0	0	0	#DIV/0!	#DIV/0!	#DIV/0!
DG(O-38:8),DG(36:1)_C18:0	0	0	0	0	0	#DIV/0!	#DIV/0!	#DIV/0!
CE(22:3)H	0	0	0	0	0	#DIV/0!	#DIV/0!	#DIV/0!
DG(O-38:9),DG(36:2)_C18:2	0	0	0	0	0	#DIV/0!	#DIV/0!	#DIV/0!
CE(20:4)Na	0	0	0	0	0	#DIV/0!	#DIV/0!	#DIV/0!
CE(22:2)H	0	0	0	0	0	#DIV/0!	#DIV/0!	#DIV/0!
DG(40:5)_C18:0	0	0	0	0	0	#DIV/0!	#DIV/0!	#DIV/0!
DG(36:7),DG(35:0)_C18:0	0	0	0	0	0	#DIV/0!	#DIV/0!	#DIV/0!
DG(34:2)_C18:1	0	0	0	0	0	#DIV/0!	#DIV/0!	#DIV/0!
CE(14:0) NH4	0	0	0	0	0	#DIV/0!	#DIV/0!	#DIV/0!
CE(20:5)K	0	0	0	0	0	#DIV/0!	#DIV/0!	#DIV/0!
CE(17:1) NH4	0	0	0	0	0	#DIV/0!	#DIV/0!	#DIV/0!
DG(O-38:9),DG(36:2)_C18:0	0	0	0	0	0	#DIV/0!	#DIV/0!	#DIV/0!
DG(34:3)_C18:2	0	0	0	0	0	#DIV/0!	#DIV/0!	#DIV/0!
DG(32:1)_C16:0	0	0	0	0	0	#DIV/0!	#DIV/0!	#DIV/0!
CE(19:0)K	0	0	0	0	0	#DIV/0!	#DIV/0!	#DIV/0!
CE(18:3)K	0	0	0	0	0	#DIV/0!	#DIV/0!	#DIV/0!
CE(20:1)K	0	0	0	0	0	#DIV/0!	#DIV/0!	#DIV/0!
CE(22:4)K	0	0	0	0	0	#DIV/0!	#DIV/0!	#DIV/0!

DG(34:3)_C16:1	0	0	0	0	0	#DIV/0!	#DIV/0!	#DIV/0!
CE(15:0)K	0	0	0	0	0	#DIV/0!	#DIV/0!	#DIV/0!
SM(d18:2/20:1)	0	0	0	0	0	#DIV/0!	#DIV/0!	#DIV/0!
PE(32:1),PE(O-33:1),PE(P-33:0)	0	0	0	0	0	#DIV/0!	#DIV/0!	#DIV/0!
DG(38:7),DG(37:0)_C16:0	0	0	0	0	0	#DIV/0!	#DIV/0!	#DIV/0!
DG(32:1)_C16:1	0	0	0	0	0	#DIV/0!	#DIV/0!	#DIV/0!
	0	0	0	0	0	#DIV/0!	#DIV/0!	#DIV/0!
CE(24:1)H	0	0	0	0	0	#DIV/0!	#DIV/0!	#DIV/0!
CE(22:0)K	0	0	0	0	0	#DIV/0!	#DIV/0!	#DIV/0!
LPE(22:6)	0	0	0	0	0	#DIV/0!	#DIV/0!	#DIV/0!
PC(44:5)	0	0	0	0	0	#DIV/0!	#DIV/0!	#DIV/0!
CE(12:0) NH4	0	0	0	0	0	#DIV/0!	#DIV/0!	#DIV/0!
DG(40:6),DG(dO-40:0)_C16:0	0	0	0	0	0	#DIV/0!	#DIV/0!	#DIV/0!
CE(17:0) NH4	0	0	0	0	0	#DIV/0!	#DIV/0!	#DIV/0!
PE(37:6),PE(O-38:6),PE(P-38:5)	0	0	0	0	0	#DIV/0!	#DIV/0!	#DIV/0!
DG(44:9),DG(43:2)_C18:1	0	0	0	0	0	#DIV/0!	#DIV/0!	#DIV/0!
[TG(58:8)]_C20:4	0	0	0	0	0	#DIV/0!	#DIV/0!	#DIV/0!
DG(44:7),DG(43:0)_C16:0	0	0	0	0	0	#DIV/0!	#DIV/0!	#DIV/0!
CE(18:2)H	0	0	0	0	0	#DIV/0!	#DIV/0!	#DIV/0!
DG(44:8),DG(43:1)_C18:1	0	0	0	0	0	#DIV/0!	#DIV/0!	#DIV/0!
CE(22:6)K	0	0	0	0	0	#DIV/0!	#DIV/0!	#DIV/0!
DG(34:3)_C16:0	0	0	0	0	0	#DIV/0!	#DIV/0!	#DIV/0!
PE(40:5)	0	0	0	0	0	#DIV/0!	#DIV/0!	#DIV/0!
DG(40:7),DG(39:0)_C16:0	0	0	0	0	0	#DIV/0!	#DIV/0!	#DIV/0!
PE(35:4),PE(O-36:4),PE(P-36:3)	0	0	0	0	0	#DIV/0!	#DIV/0!	#DIV/0!
DG(33:2)_C18:1	0	0	0	0	0	#DIV/0!	#DIV/0!	#DIV/0!
CAR(17:0)	0	0	0	0	0	#DIV/0!	#DIV/0!	#DIV/0!
[TG(44:3)]_C20:0	0	0	0	0	0	#DIV/0!	#DIV/0!	#DIV/0!
FA(14:5)	0	0	0	0	0	#DIV/0!	#DIV/0!	#DIV/0!
LPE(18:2),LPE(P-19:1)	0	0	0	0	0	#DIV/0!	#DIV/0!	#DIV/0!
[TG(40:1)]_C18:1	0	0	0	0	0	#DIV/0!	#DIV/0!	#DIV/0!
PC(43:2)	0	0	0	0	0	#DIV/0!	#DIV/0!	#DIV/0!
DG(28:2)_C18:1	0	0	0	0	0	#DIV/0!	#DIV/0!	#DIV/0!
DG(28:1)_C18:1	0	0	0	0	0	#DIV/0!	#DIV/0!	#DIV/0!
DG(31:1)_C18:1	0	0	0	0	0	#DIV/0!	#DIV/0!	#DIV/0!
DG(36:4),DG(O-37:4)_C18:1	0	0	0	0	0	#DIV/0!	#DIV/0!	#DIV/0!
CAR(22:2)	0	0	0	0	0	#DIV/0!	#DIV/0!	#DIV/0!
[TG(43:1)]_C18:1	0	0	0	0	0	#DIV/0!	#DIV/0!	#DIV/0!
[TG(38:1)]_C14:0	0	0	0	0	0	#DIV/0!	#DIV/0!	#DIV/0!
FA(40:6)	0	0	0	0	0	#DIV/0!	#DIV/0!	#DIV/0!
DG(42:6)_C16:0	0	0	0	0	0	#DIV/0!	#DIV/0!	#DIV/0!
FA(3:0)	0	0	0	0	0	#DIV/0!	#DIV/0!	#DIV/0!
[TG(43:1)]_C16:0	0	0	0	0	0	#DIV/0!	#DIV/0!	#DIV/0!
FA(27:0)	0	0	0	0	0	#DIV/0!	#DIV/0!	#DIV/0!
DG(44:7),DG(43:0)_C18:0	0	0	0	0	0	#DIV/0!	#DIV/0!	#DIV/0!
DG(42:7),DG(41:0)_C16:0	0	0	0	0	0	#DIV/0!	#DIV/0!	#DIV/0!
FA(28:1)	0	0	0	0	0	#DIV/0!	#DIV/0!	#DIV/0!
FA(26:0)	0	0	0	0	0	#DIV/0!	#DIV/0!	#DIV/0!
DG(38:6),DG(dO-40:6)_C16:0	0	0	0	0	0	#DIV/0!	#DIV/0!	#DIV/0!
FA(34:1)	0	0	0	0	0	#DIV/0!	#DIV/0!	#DIV/0!
[TG(55:7),TG(54:0)]_C14:0	0	0	0	0	0	#DIV/0!	#DIV/0!	#DIV/0!
[TG(59:9),TG(58:2)]_C18:1	0	0	0	0	0	#DIV/0!	#DIV/0!	#DIV/0!
[TG(48:3)]_C18:3	0	0	0	0	0	#DIV/0!	#DIV/0!	#DIV/0!
[TG(44:1)]_C16:1	0	0	0	0	0	#DIV/0!	#DIV/0!	#DIV/0!
[TG(48:8),TG(47:1)]_C14:0	0	0	0	0	0	#DIV/0!	#DIV/0!	#DIV/0!
[TG(62:16),TG(61:9),TG(60:2)]_(	0	0	0	0	0	#DIV/0!	#DIV/0!	#DIV/0!
[TG(60:15),TG(59:8),TG(58:1)]_(	0	0	0	0	0	#DIV/0!	#DIV/0!	#DIV/0!
[TG(61:10),TG(60:3)]_C18:1	0	0	0	0	0	#DIV/0!	#DIV/0!	#DIV/0!
[TG(52:4)]_C14:0	0	0	0	0	0	#DIV/0!	#DIV/0!	#DIV/0!
[TG(47:2)]_C18:2	0	0	0	0	0	#DIV/0!	#DIV/0!	#DIV/0!
[TG(50:5)]_C20:4	0	0	0	0	0	#DIV/0!	#DIV/0!	#DIV/0!
[TG(42:1)]_C16:0	0	0	0	0	0	#DIV/0!	#DIV/0!	#DIV/0!
PC(14:0),LPC(15:0),LPC(O-16:(	0	0	0	0	0	#DIV/0!	#DIV/0!	#DIV/0!
[TG(59:11),TG(58:4)]_C18:2	0	0	0	0	0	#DIV/0!	#DIV/0!	#DIV/0!
[TG(50:7)]_C18:1	0	0	0	0	0	#DIV/0!	#DIV/0!	#DIV/0!
[TG(45:0)]_C14:0	0	0	0	0	0	#DIV/0!	#DIV/0!	#DIV/0!
LPC(15:1),LPC(O-16:1),LPC(P-1	0	0	0	0	0	#DIV/0!	#DIV/0!	#DIV/0!
[TG(58:10)]_C20:4	0	0	0	0	0	#DIV/0!	#DIV/0!	#DIV/0!

[TG(50:9),TG(49:2)]_C14:0	0	0	0	0	0	#DIV/0!	#DIV/0!	#DIV/0!
PI(36:3),PI(P-37:2)	0	0	0	0	0	#DIV/0!	#DIV/0!	#DIV/0!
[TG(47:6)]_C14:0	0	0	0	0	0	#DIV/0!	#DIV/0!	#DIV/0!
[TG(47:2)]_C16:0	0	0	0	0	0	#DIV/0!	#DIV/0!	#DIV/0!
[TG(59:9),TG(58:2)]_C16:0	0	0	0	0	0	#DIV/0!	#DIV/0!	#DIV/0!
[TG(43:0)]_C16:0	0	0	0	0	0	#DIV/0!	#DIV/0!	#DIV/0!
[TG(46:3)]_C14:0	0	0	0	0	0	#DIV/0!	#DIV/0!	#DIV/0!
[TG(51:8),TG(50:1)]_C20:0	0	0	0	0	0	#DIV/0!	#DIV/0!	#DIV/0!
[TG(42:2)]_C18:2	0	0	0	0	0	#DIV/0!	#DIV/0!	#DIV/0!
[TG(42:1)]_C14:0	0	0	0	0	0	#DIV/0!	#DIV/0!	#DIV/0!
[TG(45:1)]_C16:0	0	0	0	0	0	#DIV/0!	#DIV/0!	#DIV/0!
[TG(44:2)]_C16:1	0	0	0	0	0	#DIV/0!	#DIV/0!	#DIV/0!
[TG(44:2)]_C14:0	0	0	0	0	0	#DIV/0!	#DIV/0!	#DIV/0!
FA(36:5)	0	0	0	0	0	#DIV/0!	#DIV/0!	#DIV/0!
PG(O-37:2),PG(P-37:1); PG(O-3	0	0	0	0	0	#DIV/0!	#DIV/0!	#DIV/0!
[TG(47:2)]_C14:0	0	0	0	0	0	#DIV/0!	#DIV/0!	#DIV/0!
[TG(49:3)]_C18:1	0	0	0	0	0	#DIV/0!	#DIV/0!	#DIV/0!
[TG(45:1)]_C18:1	0	0	0	0	0	#DIV/0!	#DIV/0!	#DIV/0!
[TG(47:2)]_C18:1	0	0	0	0	0	#DIV/0!	#DIV/0!	#DIV/0!
[TG(52:6)]_C14:0	0	0	0	0	0	#DIV/0!	#DIV/0!	#DIV/0!
[TG(54:7)]_C16:1	0	0	0	0	0	#DIV/0!	#DIV/0!	#DIV/0!
DG(32:2)_C18:2	0	0	0	0	0	#DIV/0!	#DIV/0!	#DIV/0!
LPC(17:1),LPC(O-18:1),LPC(P-1	0	0	0	0	0	#DIV/0!	#DIV/0!	#DIV/0!
[TG(60:15),TG(59:8),TG(58:1)]_(	0	0	0	0	0	#DIV/0!	#DIV/0!	#DIV/0!
CAR(16:0)	0	0	0	0	0	#DIV/0!	#DIV/0!	#DIV/0!
PG(36:3),PG(P-37:2); PG(36:3),I	0	0	0	0	0	#DIV/0!	#DIV/0!	#DIV/0!
PC(50:0)	0	0	0	0	0	#DIV/0!	#DIV/0!	#DIV/0!
[TG(40:0)]_C14:0	0	0	0	0	0	#DIV/0!	#DIV/0!	#DIV/0!
[TG(47:6)]_C18:0	0	0	0	0	0	#DIV/0!	#DIV/0!	#DIV/0!
DG(44:1)_C18:1	0	0	0	0	0	#DIV/0!	#DIV/0!	#DIV/0!
FA(31:0)	0	0	0	0	0	#DIV/0!	#DIV/0!	#DIV/0!
PI(34:1),PI(O-35:1),PI(P-35:0)	0	0	0	0	0	#DIV/0!	#DIV/0!	#DIV/0!
[TG(49:3)]_C16:1	0	0	0	0	0	#DIV/0!	#DIV/0!	#DIV/0!
DG(38:8),DG(dO-40:8),DG(37:1	0	0	0	0	0	#DIV/0!	#DIV/0!	#DIV/0!
[TG(38:0)]_C16:0	0	0	0	0	0	#DIV/0!	#DIV/0!	#DIV/0!
[TG(45:4)]_C18:1	0	0	0	0	0	#DIV/0!	#DIV/0!	#DIV/0!
[TG(50:9)]_C22:6	0	0	0	0	0	#DIV/0!	#DIV/0!	#DIV/0!
[TG(40:1)]_C16:0	0	0	0	0	0	#DIV/0!	#DIV/0!	#DIV/0!
[TG(60:14),TG(59:7),TG(58:0)]_(	0	0	0	0	0	#DIV/0!	#DIV/0!	#DIV/0!
PG(30:0),PG(O-31:0); PG(30:0),	0	0	0	0	0	#DIV/0!	#DIV/0!	#DIV/0!
[TG(54:12),TG(53:5)]_C22:5	0	0	0	0	0	#DIV/0!	#DIV/0!	#DIV/0!
DG(41:7),DG(40:0)_C16:0	0	0	0	0	0	#DIV/0!	#DIV/0!	#DIV/0!
[TG(44:4)]_C20:0	0	0	0	0	0	#DIV/0!	#DIV/0!	#DIV/0!
FA(28:3)	0	0	0	0	0	#DIV/0!	#DIV/0!	#DIV/0!
[TG(49:3)]_C20:0	0	0	0	0	0	#DIV/0!	#DIV/0!	#DIV/0!
[TG(45:4)]_C16:1	0	0	0	0	0	#DIV/0!	#DIV/0!	#DIV/0!
FA(25:4)	0	0	0	0	0	#DIV/0!	#DIV/0!	#DIV/0!
FA(30:3)	0	0	0	0	0	#DIV/0!	#DIV/0!	#DIV/0!
CE(50:3;O2)H	0	0	0	0	0	#DIV/0!	#DIV/0!	#DIV/0!
PG(26:0); PG(26:0)	0	0	0	0	0	#DIV/0!	#DIV/0!	#DIV/0!
PC(44:6)	0	0	0	0	0	#DIV/0!	#DIV/0!	#DIV/0!
[TG(40:1)]_C14:0	0	0	0	0	0	#DIV/0!	#DIV/0!	#DIV/0!
[TG(42:1)]_C16:1	0	0	0	0	0	#DIV/0!	#DIV/0!	#DIV/0!
[TG(45:1)]_C14:0	0	0	0	0	0	#DIV/0!	#DIV/0!	#DIV/0!
[TG(55:7)]_C22:6	0	0	0	0	0	#DIV/0!	#DIV/0!	#DIV/0!
CE(46:3;O2) NH4	0	0	0	0	0	#DIV/0!	#DIV/0!	#DIV/0!
FA(12:6)	0	0	0	0	0	#DIV/0!	#DIV/0!	#DIV/0!
FA(17:1)	0	0	0	0	0	#DIV/0!	#DIV/0!	#DIV/0!
FA(24:3)	0	0	0	0	0	#DIV/0!	#DIV/0!	#DIV/0!
FA(26:3)	0	0	0	0	0	#DIV/0!	#DIV/0!	#DIV/0!
FA(38:4)	0	0	0	0	0	#DIV/0!	#DIV/0!	#DIV/0!
FA(5:1)	0	0	0	0	0	#DIV/0!	#DIV/0!	#DIV/0!
DG(29:2)_C16:0	0	0	0	0	0	#DIV/0!	#DIV/0!	#DIV/0!
PG(28:0),PG(O-29:0); PG(28:0),	0	0	0	0	0	#DIV/0!	#DIV/0!	#DIV/0!
FA(15:5)	0	0	0	0	0	#DIV/0!	#DIV/0!	#DIV/0!
FA(17:6)	0	0	0	0	0	#DIV/0!	#DIV/0!	#DIV/0!
FA(26:5)	0	0	0	0	0	#DIV/0!	#DIV/0!	#DIV/0!
[TG(57:8),TG(56:1)]_C16:1	0	0	0	0	0	#DIV/0!	#DIV/0!	#DIV/0!

LPS(O-20:0)	0	0	0	0	0	#DIV/0!	#DIV/0!	#DIV/0!
LPE(12:0)	0	0	0	0	0	#DIV/0!	#DIV/0!	#DIV/0!
[TG(48:5)]_C22:5	0	0	0	0	0	#DIV/0!	#DIV/0!	#DIV/0!
[TG(50:5)]_C22:5	0	0	0	0	0	#DIV/0!	#DIV/0!	#DIV/0!
[TG(59:12),TG(58:5)]_C22:5	0	0	0	0	0	#DIV/0!	#DIV/0!	#DIV/0!
DG(34:5)_C16:1	0	0	0	0	0	#DIV/0!	#DIV/0!	#DIV/0!
DG(42:0)_C16:0	0	0	0	0	0	#DIV/0!	#DIV/0!	#DIV/0!
FA(27:2)	0	0	0	0	0	#DIV/0!	#DIV/0!	#DIV/0!
DG(44:8),DG(43:1)_C16:0	0	0	0	0	0	#DIV/0!	#DIV/0!	#DIV/0!
CE(16:2) NH4	0	0	0	0	0	#DIV/0!	#DIV/0!	#DIV/0!
CE(16:1)K	0	0	0	0	0	#DIV/0!	#DIV/0!	#DIV/0!
LPC(22:5)	0	0	0	0	0	#DIV/0!	#DIV/0!	#DIV/0!
DG(40:8),DG(39:1)_C18:1	0	0	0	0	0	#DIV/0!	#DIV/0!	#DIV/0!
CE(18:0)H	0	0	0	0	0	#DIV/0!	#DIV/0!	#DIV/0!
CE(20:4)K	0	0	0	0	0	#DIV/0!	#DIV/0!	#DIV/0!
DG(31:1)_C16:0	0	0	0	0	0	#DIV/0!	#DIV/0!	#DIV/0!
CE(22:5)Na	0	0	0	0	0	#DIV/0!	#DIV/0!	#DIV/0!
DG(44:9),DG(43:2)_C18:2	0	0	0	0	0	#DIV/0!	#DIV/0!	#DIV/0!
DG(42:8),DG(41:1)_C18:1	0	0	0	0	0	#DIV/0!	#DIV/0!	#DIV/0!
[TG(37:0)]_C20:0	0	0	0	0	0	#DIV/0!	#DIV/0!	#DIV/0!
PE(40:4)	0	0	0	0	0	#DIV/0!	#DIV/0!	#DIV/0!
DG(30:2)_C18:2	0	0	0	0	0	#DIV/0!	#DIV/0!	#DIV/0!
CE(12:0)Na	0	0	0	0	0	#DIV/0!	#DIV/0!	#DIV/0!
CE(22:3)Na	0	0	0	0	0	#DIV/0!	#DIV/0!	#DIV/0!
DG(33:3)_C18:2	0	0	0	0	0	#DIV/0!	#DIV/0!	#DIV/0!
DG(44:0)_C16:0	0	0	0	0	0	#DIV/0!	#DIV/0!	#DIV/0!
FA(14:1)	0	0	0	0	0	#DIV/0!	#DIV/0!	#DIV/0!
DG(38:7),DG(37:0)_C18:0	0	0	0	0	0	#DIV/0!	#DIV/0!	#DIV/0!
PC(18:0),LPC(19:0),PC(O-19:0)	0	0	0	0	0	#DIV/0!	#DIV/0!	#DIV/0!
PE(39:6),PE(O-40:6),PE(P-40:5)	0	0	0	0	0	#DIV/0!	#DIV/0!	#DIV/0!
[TG(45:0)]_C18:0	0	0	0	0	0	#DIV/0!	#DIV/0!	#DIV/0!
[TG(51:4)]_C16:1	0	0	0	0	0	#DIV/0!	#DIV/0!	#DIV/0!
[TG(52:9),TG(51:2)]_C14:0	0	0	0	0	0	#DIV/0!	#DIV/0!	#DIV/0!
DG(26:0)_C16:0	0	0	0	0	0	#DIV/0!	#DIV/0!	#DIV/0!
CE(16:3) NH4	0	0	0	0	0	#DIV/0!	#DIV/0!	#DIV/0!
CE(22:5)K	0	0	0	0	0	#DIV/0!	#DIV/0!	#DIV/0!
SM(d16:0/14:0)	0	0	0	0	0	#DIV/0!	#DIV/0!	#DIV/0!
CE(20:3)K	0	0	0	0	0	#DIV/0!	#DIV/0!	#DIV/0!
CE(22:0) NH4	0	0	0	0	0	#DIV/0!	#DIV/0!	#DIV/0!
FA(26:1)	0	0	0	0	0	#DIV/0!	#DIV/0!	#DIV/0!
DG(33:1),DG(O-34:1)_C16:0	0	0	0	0	0	#DIV/0!	#DIV/0!	#DIV/0!
DG(39:7),DG(38:0),DG(dO-40:0	0	0	0	0	0	#DIV/0!	#DIV/0!	#DIV/0!
[TG(44:4)]_C18:1	0	0	0	0	0	#DIV/0!	#DIV/0!	#DIV/0!
[TG(45:3)]_C20:0	0	0	0	0	0	#DIV/0!	#DIV/0!	#DIV/0!
[TG(51:9)]_C22:6	0	0	0	0	0	#DIV/0!	#DIV/0!	#DIV/0!
DG(32:2)_C18:0	0	0	0	0	0	#DIV/0!	#DIV/0!	#DIV/0!
FA(29:2)	0	0	0	0	0	#DIV/0!	#DIV/0!	#DIV/0!
[TG(39:0)]_C18:0	0	0	0	0	0	#DIV/0!	#DIV/0!	#DIV/0!
[TG(44:3)]_C16:1	0	0	0	0	0	#DIV/0!	#DIV/0!	#DIV/0!
DG(36:8),DG(35:1)_C16:0	0	0	0	0	0	#DIV/0!	#DIV/0!	#DIV/0!
DG(38:9),DG(dO-40:9),DG(37:2	0	0	0	0	0	#DIV/0!	#DIV/0!	#DIV/0!
FA(16:1)	0	0	0	0	0	#DIV/0!	#DIV/0!	#DIV/0!
CAR(10:1)	0	0	0	0	0	#DIV/0!	#DIV/0!	#DIV/0!
CAR(20:4)	0	0	0	0	0	#DIV/0!	#DIV/0!	#DIV/0!
[TG(44:5)]_C18:2	0	0	0	0	0	#DIV/0!	#DIV/0!	#DIV/0!
FA(11:0)	0	0	0	0	0	#DIV/0!	#DIV/0!	#DIV/0!
FA(29:0)	0	0	0	0	0	#DIV/0!	#DIV/0!	#DIV/0!
CAR	0	0	0	0	0	#DIV/0!	#DIV/0!	#DIV/0!
CAR(5:0)	0	0	0	0	0	#DIV/0!	#DIV/0!	#DIV/0!
CAR(7:0)	0	0	0	0	0	#DIV/0!	#DIV/0!	#DIV/0!
CAR(8:1)	0	0	0	0	0	#DIV/0!	#DIV/0!	#DIV/0!
CAR(8:0)	0	0	0	0	0	#DIV/0!	#DIV/0!	#DIV/0!
CAR(9:0)	0	0	0	0	0	#DIV/0!	#DIV/0!	#DIV/0!
CAR(10:0)	0	0	0	0	0	#DIV/0!	#DIV/0!	#DIV/0!
CAR(11:0)	0	0	0	0	0	#DIV/0!	#DIV/0!	#DIV/0!
CAR(12:0)	0	0	0	0	0	#DIV/0!	#DIV/0!	#DIV/0!
CAR(16:2)	0	0	0	0	0	#DIV/0!	#DIV/0!	#DIV/0!
CAR(16:1)	0	0	0	0	0	#DIV/0!	#DIV/0!	#DIV/0!

CAR(18:4)	0	0	0	0	0	#DIV/0!	#DIV/0!	#DIV/0!
CAR(18:1)	0	0	0	0	0	#DIV/0!	#DIV/0!	#DIV/0!
LPC(12:0)	0	0	0	0	0	#DIV/0!	#DIV/0!	#DIV/0!
LPG(12:0); LPG(12:0)	0	0	0	0	0	#DIV/0!	#DIV/0!	#DIV/0!
CAR(22:6)	0	0	0	0	0	#DIV/0!	#DIV/0!	#DIV/0!
LPG(14:0); LPG(14:0)	0	0	0	0	0	#DIV/0!	#DIV/0!	#DIV/0!
LPE(18:3)	0	0	0	0	0	#DIV/0!	#DIV/0!	#DIV/0!
CAR(22:4)	0	0	0	0	0	#DIV/0!	#DIV/0!	#DIV/0!
LPC(16:1),LPC(P-17:0)	0	0	0	0	0	#DIV/0!	#DIV/0!	#DIV/0!
LPE(20:5)	0	0	0	0	0	#DIV/0!	#DIV/0!	#DIV/0!
LPE(20:3)	0	0	0	0	0	#DIV/0!	#DIV/0!	#DIV/0!
LPC(18:3)	0	0	0	0	0	#DIV/0!	#DIV/0!	#DIV/0!
LPE(22:5)	0	0	0	0	0	#DIV/0!	#DIV/0!	#DIV/0!
LPG(18:1); LPG(18:1)	0	0	0	0	0	#DIV/0!	#DIV/0!	#DIV/0!
PC(18:1),LPC(19:1),PC(O-19:1)	0	0	0	0	0	#DIV/0!	#DIV/0!	#DIV/0!
LPC(20:5)	0	0	0	0	0	#DIV/0!	#DIV/0!	#DIV/0!
LPS(22:6)	0	0	0	0	0	#DIV/0!	#DIV/0!	#DIV/0!
LPG(22:6); LPG(22:6)	0	0	0	0	0	#DIV/0!	#DIV/0!	#DIV/0!
LPG(22:0); LPG(22:0)	0	0	0	0	0	#DIV/0!	#DIV/0!	#DIV/0!
LPI(19:1),LPI(P-20:0)	0	0	0	0	0	#DIV/0!	#DIV/0!	#DIV/0!
PG(26:1); PG(26:1)	0	0	0	0	0	#DIV/0!	#DIV/0!	#DIV/0!
SM(d18:0/13:0)	0	0	0	0	0	#DIV/0!	#DIV/0!	#DIV/0!
Cer(d18:1/25:0)	0	0	0	0	0	#DIV/0!	#DIV/0!	#DIV/0!
PG(28:1),PG(P-29:0); PG(28:1),	0	0	0	0	0	#DIV/0!	#DIV/0!	#DIV/0!
PG(29:0),PG(O-30:0); PG(29:0),	0	0	0	0	0	#DIV/0!	#DIV/0!	#DIV/0!
PG(30:1),PG(O-31:1),PG(P-31:0	0	0	0	0	0	#DIV/0!	#DIV/0!	#DIV/0!
PE(34:4)	0	0	0	0	0	#DIV/0!	#DIV/0!	#DIV/0!
PG(31:0),PG(O-32:0); PG(31:0),	0	0	0	0	0	#DIV/0!	#DIV/0!	#DIV/0!
PG(32:2),PG(O-33:2),PG(P-33:1	0	0	0	0	0	#DIV/0!	#DIV/0!	#DIV/0!
PC(33:5),PC(P-34:4)	0	0	0	0	0	#DIV/0!	#DIV/0!	#DIV/0!
PG(32:1),PG(O-33:1),PG(P-33:0	0	0	0	0	0	#DIV/0!	#DIV/0!	#DIV/0!
PC(33:4),PC(O-34:4),PC(O-34:4	0	0	0	0	0	#DIV/0!	#DIV/0!	#DIV/0!
PC(34:5)	0	0	0	0	0	#DIV/0!	#DIV/0!	#DIV/0!
PG(33:1),PG(O-34:1),PG(P-34:0	0	0	0	0	0	#DIV/0!	#DIV/0!	#DIV/0!
PC(34:4),PC(O-35:4)	0	0	0	0	0	#DIV/0!	#DIV/0!	#DIV/0!
PE(37:4),PE(O-38:4),PE(P-38:3)	0	0	0	0	0	#DIV/0!	#DIV/0!	#DIV/0!
PG(33:0),PG(O-34:0); PG(33:0),	0	0	0	0	0	#DIV/0!	#DIV/0!	#DIV/0!
PG(34:0),PG(O-35:0); PG(34:0),	0	0	0	0	0	#DIV/0!	#DIV/0!	#DIV/0!
PG(35:6),PG(P-36:5); PG(35:6),	0	0	0	0	0	#DIV/0!	#DIV/0!	#DIV/0!
PG(36:1),PG(O-37:1),PG(P-37:0	0	0	0	0	0	#DIV/0!	#DIV/0!	#DIV/0!
PG(37:7),PG(P-38:6),PG(36:0),F	0	0	0	0	0	#DIV/0!	#DIV/0!	#DIV/0!
PE(40:3)	0	0	0	0	0	#DIV/0!	#DIV/0!	#DIV/0!
PE(42:5)	0	0	0	0	0	#DIV/0!	#DIV/0!	#DIV/0!
PS(40:4)	0	0	0	0	0	#DIV/0!	#DIV/0!	#DIV/0!
PE(44:8),PE(43:1)	0	0	0	0	0	#DIV/0!	#DIV/0!	#DIV/0!
PE(44:7),PE(43:0)	0	0	0	0	0	#DIV/0!	#DIV/0!	#DIV/0!
PS(O-42:0)	0	0	0	0	0	#DIV/0!	#DIV/0!	#DIV/0!
PS(42:5)	0	0	0	0	0	#DIV/0!	#DIV/0!	#DIV/0!
PS(43:1)	0	0	0	0	0	#DIV/0!	#DIV/0!	#DIV/0!
PS(43:0)	0	0	0	0	0	#DIV/0!	#DIV/0!	#DIV/0!
PS(44:6)	0	0	0	0	0	#DIV/0!	#DIV/0!	#DIV/0!
PI(40:6)	0	0	0	0	0	#DIV/0!	#DIV/0!	#DIV/0!
PI(40:5)	0	0	0	0	0	#DIV/0!	#DIV/0!	#DIV/0!
[TG(63:13),TG(62:6)]_C22:6	0	0	0	0	0	#DIV/0!	#DIV/0!	#DIV/0!
[TG(38:1)]_C16:1	0	0	0	0	0	#DIV/0!	#DIV/0!	#DIV/0!
[TG(39:1)]_C18:1	0	0	0	0	0	#DIV/0!	#DIV/0!	#DIV/0!
[TG(39:1)]_C16:0	0	0	0	0	0	#DIV/0!	#DIV/0!	#DIV/0!
[TG(39:1)]_C16:1	0	0	0	0	0	#DIV/0!	#DIV/0!	#DIV/0!
[TG(39:1)]_C14:0	0	0	0	0	0	#DIV/0!	#DIV/0!	#DIV/0!
[TG(39:0)]_C14:0	0	0	0	0	0	#DIV/0!	#DIV/0!	#DIV/0!
[TG(40:2)]_C18:1	0	0	0	0	0	#DIV/0!	#DIV/0!	#DIV/0!
[TG(40:2)]_C16:1	0	0	0	0	0	#DIV/0!	#DIV/0!	#DIV/0!
[TG(40:1)]_C18:0	0	0	0	0	0	#DIV/0!	#DIV/0!	#DIV/0!
[TG(40:1)]_C16:1	0	0	0	0	0	#DIV/0!	#DIV/0!	#DIV/0!
[TG(41:2)]_C18:1	0	0	0	0	0	#DIV/0!	#DIV/0!	#DIV/0!
[TG(41:1)]_C18:1	0	0	0	0	0	#DIV/0!	#DIV/0!	#DIV/0!
[TG(41:1)]_C16:0	0	0	0	0	0	#DIV/0!	#DIV/0!	#DIV/0!
[TG(41:1)]_C16:1	0	0	0	0	0	#DIV/0!	#DIV/0!	#DIV/0!

[TG(41:1)]_C14:0	0	0	0	0	0	#DIV/0!	#DIV/0!	#DIV/0!
[TG(41:0)]_C18:0	0	0	0	0	0	#DIV/0!	#DIV/0!	#DIV/0!
[TG(41:0)]_C14:0	0	0	0	0	0	#DIV/0!	#DIV/0!	#DIV/0!
[TG(42:2)]_C16:1	0	0	0	0	0	#DIV/0!	#DIV/0!	#DIV/0!
[TG(42:2)]_C14:0	0	0	0	0	0	#DIV/0!	#DIV/0!	#DIV/0!
[TG(43:2)]_C16:1	0	0	0	0	0	#DIV/0!	#DIV/0!	#DIV/0!
[TG(43:1)]_C14:0	0	0	0	0	0	#DIV/0!	#DIV/0!	#DIV/0!
[TG(43:0)]_C18:0	0	0	0	0	0	#DIV/0!	#DIV/0!	#DIV/0!
[TG(43:0)]_C14:0	0	0	0	0	0	#DIV/0!	#DIV/0!	#DIV/0!
[TG(44:5)]_C22:5	0	0	0	0	0	#DIV/0!	#DIV/0!	#DIV/0!
[TG(44:4)]_C16:1	0	0	0	0	0	#DIV/0!	#DIV/0!	#DIV/0!
[TG(45:3)]_C16:1	0	0	0	0	0	#DIV/0!	#DIV/0!	#DIV/0!
[TG(45:2)]_C18:1	0	0	0	0	0	#DIV/0!	#DIV/0!	#DIV/0!
[TG(45:2)]_C18:2	0	0	0	0	0	#DIV/0!	#DIV/0!	#DIV/0!
[TG(45:2)]_C14:0	0	0	0	0	0	#DIV/0!	#DIV/0!	#DIV/0!
[TG(45:1)]_C18:0	0	0	0	0	0	#DIV/0!	#DIV/0!	#DIV/0!
[TG(46:6)]_C18:0	0	0	0	0	0	#DIV/0!	#DIV/0!	#DIV/0!
[TG(46:6)]_C14:0	0	0	0	0	0	#DIV/0!	#DIV/0!	#DIV/0!
[TG(47:3)]_C18:2	0	0	0	0	0	#DIV/0!	#DIV/0!	#DIV/0!
[TG(47:3)]_C16:1	0	0	0	0	0	#DIV/0!	#DIV/0!	#DIV/0!
[TG(48:8),TG(47:1)]_C18:0	0	0	0	0	0	#DIV/0!	#DIV/0!	#DIV/0!
[TG(48:7),TG(47:0)]_C18:0	0	0	0	0	0	#DIV/0!	#DIV/0!	#DIV/0!
[TG(48:7)]_C16:1	0	0	0	0	0	#DIV/0!	#DIV/0!	#DIV/0!
[TG(48:6)]_C14:0	0	0	0	0	0	#DIV/0!	#DIV/0!	#DIV/0!
[TG(48:4)]_C16:1	0	0	0	0	0	#DIV/0!	#DIV/0!	#DIV/0!
[TG(49:6)]_C14:0	0	0	0	0	0	#DIV/0!	#DIV/0!	#DIV/0!
[TG(49:4)]_C16:1	0	0	0	0	0	#DIV/0!	#DIV/0!	#DIV/0!
[TG(50:9),TG(49:2)]_C18:0	0	0	0	0	0	#DIV/0!	#DIV/0!	#DIV/0!
[TG(50:8),TG(49:1)]_C20:0	0	0	0	0	0	#DIV/0!	#DIV/0!	#DIV/0!
[TG(50:8),TG(49:1)]_C18:0	0	0	0	0	0	#DIV/0!	#DIV/0!	#DIV/0!
[TG(50:7)]_C16:1	0	0	0	0	0	#DIV/0!	#DIV/0!	#DIV/0!
[TG(50:7),TG(49:0)]_C14:0	0	0	0	0	0	#DIV/0!	#DIV/0!	#DIV/0!
[TG(50:6)]_C16:0	0	0	0	0	0	#DIV/0!	#DIV/0!	#DIV/0!
[TG(51:7)]_C22:5	0	0	0	0	0	#DIV/0!	#DIV/0!	#DIV/0!
[TG(52:8),TG(51:1)]_C16:1	0	0	0	0	0	#DIV/0!	#DIV/0!	#DIV/0!
[TG(52:8),TG(51:1)]_C14:0	0	0	0	0	0	#DIV/0!	#DIV/0!	#DIV/0!
[TG(52:7),TG(51:0)]_C14:0	0	0	0	0	0	#DIV/0!	#DIV/0!	#DIV/0!
[TG(53:8),TG(52:1)]_C14:0	0	0	0	0	0	#DIV/0!	#DIV/0!	#DIV/0!
[TG(53:7)]_C16:1	0	0	0	0	0	#DIV/0!	#DIV/0!	#DIV/0!
[TG(53:7),TG(52:0)]_C14:0	0	0	0	0	0	#DIV/0!	#DIV/0!	#DIV/0!
[TG(53:6)]_C16:0	0	0	0	0	0	#DIV/0!	#DIV/0!	#DIV/0!
[TG(54:11),TG(53:4)]_C16:1	0	0	0	0	0	#DIV/0!	#DIV/0!	#DIV/0!
[TG(54:10),TG(53:3)]_C16:1	0	0	0	0	0	#DIV/0!	#DIV/0!	#DIV/0!
[TG(54:9),TG(53:2)]_C16:1	0	0	0	0	0	#DIV/0!	#DIV/0!	#DIV/0!
[TG(54:8),TG(53:1)]_C16:1	0	0	0	0	0	#DIV/0!	#DIV/0!	#DIV/0!
[TG(54:7),TG(53:0)]_C14:0	0	0	0	0	0	#DIV/0!	#DIV/0!	#DIV/0!
[TG(55:9),TG(54:2)]_C16:1	0	0	0	0	0	#DIV/0!	#DIV/0!	#DIV/0!
[TG(55:8),TG(54:1)]_C16:1	0	0	0	0	0	#DIV/0!	#DIV/0!	#DIV/0!
[TG(56:10),TG(55:3)]_C16:1	0	0	0	0	0	#DIV/0!	#DIV/0!	#DIV/0!
[TG(56:9),TG(55:2)]_C16:1	0	0	0	0	0	#DIV/0!	#DIV/0!	#DIV/0!
[TG(56:8),TG(55:1)]_C16:1	0	0	0	0	0	#DIV/0!	#DIV/0!	#DIV/0!
[TG(56:8),TG(55:1)]_C14:0	0	0	0	0	0	#DIV/0!	#DIV/0!	#DIV/0!
[TG(56:7)]_C16:1	0	0	0	0	0	#DIV/0!	#DIV/0!	#DIV/0!
[TG(56:7),TG(55:0)]_C14:0	0	0	0	0	0	#DIV/0!	#DIV/0!	#DIV/0!
[TG(57:12),TG(56:5)]_C16:1	0	0	0	0	0	#DIV/0!	#DIV/0!	#DIV/0!
[TG(57:10),TG(56:3)]_C16:1	0	0	0	0	0	#DIV/0!	#DIV/0!	#DIV/0!
[TG(57:9),TG(56:2)]_C16:1	0	0	0	0	0	#DIV/0!	#DIV/0!	#DIV/0!
[TG(57:8),TG(56:1)]_C14:0	0	0	0	0	0	#DIV/0!	#DIV/0!	#DIV/0!
[TG(58:14),TG(57:7),TG(56:0)]_[illegible]	0	0	0	0	0	#DIV/0!	#DIV/0!	#DIV/0!
[TG(58:14),TG(57:7)]_C16:1	0	0	0	0	0	#DIV/0!	#DIV/0!	#DIV/0!
[TG(58:14),TG(57:7),TG(56:0)]_[illegible]	0	0	0	0	0	#DIV/0!	#DIV/0!	#DIV/0!
[TG(58:9),TG(57:2)]_C16:1	0	0	0	0	0	#DIV/0!	#DIV/0!	#DIV/0!
[TG(58:8),TG(57:1)]_C16:0	0	0	0	0	0	#DIV/0!	#DIV/0!	#DIV/0!
[TG(58:8),TG(57:1)]_C16:1	0	0	0	0	0	#DIV/0!	#DIV/0!	#DIV/0!
[TG(58:8),TG(57:1)]_C14:0	0	0	0	0	0	#DIV/0!	#DIV/0!	#DIV/0!
[TG(58:7),TG(57:0)]_C16:0	0	0	0	0	0	#DIV/0!	#DIV/0!	#DIV/0!
[TG(59:10),TG(58:3)]_C16:1	0	0	0	0	0	#DIV/0!	#DIV/0!	#DIV/0!
[TG(59:9),TG(58:2)]_C16:1	0	0	0	0	0	#DIV/0!	#DIV/0!	#DIV/0!

[TG(60:15),TG(59:8),TG(58:1)]_(	0	0	0	0	0	#DIV/0!	#DIV/0!	#DIV/0!
[TG(60:15),TG(59:8),TG(58:1)]_(	0	0	0	0	0	#DIV/0!	#DIV/0!	#DIV/0!
[TG(60:15),TG(59:8),TG(58:1)]_(	0	0	0	0	0	#DIV/0!	#DIV/0!	#DIV/0!
[TG(60:14),TG(59:7),TG(58:0)]_(	0	0	0	0	0	#DIV/0!	#DIV/0!	#DIV/0!
[TG(60:12),TG(59:5)]_C18:2	0	0	0	0	0	#DIV/0!	#DIV/0!	#DIV/0!
[TG(60:9),TG(59:2)]_C18:1	0	0	0	0	0	#DIV/0!	#DIV/0!	#DIV/0!
[TG(60:9),TG(59:2)]_C16:1	0	0	0	0	0	#DIV/0!	#DIV/0!	#DIV/0!
[TG(60:8),TG(59:1)]_C18:1	0	0	0	0	0	#DIV/0!	#DIV/0!	#DIV/0!
[TG(60:8),TG(59:1)]_C16:0	0	0	0	0	0	#DIV/0!	#DIV/0!	#DIV/0!
[TG(60:8),TG(59:1)]_C16:1	0	0	0	0	0	#DIV/0!	#DIV/0!	#DIV/0!
[TG(61:14),TG(60:7),TG(59:0)]_(	0	0	0	0	0	#DIV/0!	#DIV/0!	#DIV/0!
[TG(61:13),TG(60:6)]_C20:0	0	0	0	0	0	#DIV/0!	#DIV/0!	#DIV/0!
[TG(61:11),TG(60:4)]_C20:4	0	0	0	0	0	#DIV/0!	#DIV/0!	#DIV/0!
[TG(61:11),TG(60:4)]_C18:0	0	0	0	0	0	#DIV/0!	#DIV/0!	#DIV/0!
[TG(61:10),TG(60:3)]_C16:1	0	0	0	0	0	#DIV/0!	#DIV/0!	#DIV/0!
[TG(62:15),TG(61:8),TG(60:1)]_(	0	0	0	0	0	#DIV/0!	#DIV/0!	#DIV/0!
[TG(62:15),TG(61:8),TG(60:1)]_(	0	0	0	0	0	#DIV/0!	#DIV/0!	#DIV/0!
DG(26:0)_C18:0	0	0	0	0	0	#DIV/0!	#DIV/0!	#DIV/0!
DG(28:2)_C18:2	0	0	0	0	0	#DIV/0!	#DIV/0!	#DIV/0!
DG(29:2)_C18:1	0	0	0	0	0	#DIV/0!	#DIV/0!	#DIV/0!
DG(29:1)_C18:1	0	0	0	0	0	#DIV/0!	#DIV/0!	#DIV/0!
DG(29:1)_C16:0	0	0	0	0	0	#DIV/0!	#DIV/0!	#DIV/0!
DG(31:2),DG(P-14:0/18:1)_C18	0	0	0	0	0	#DIV/0!	#DIV/0!	#DIV/0!
CE(12:0)H	0	0	0	0	0	#DIV/0!	#DIV/0!	#DIV/0!
DG(31:1)_C16:1	0	0	0	0	0	#DIV/0!	#DIV/0!	#DIV/0!
DG(31:0)_C16:0	0	0	0	0	0	#DIV/0!	#DIV/0!	#DIV/0!
DG(32:2)_C16:1	0	0	0	0	0	#DIV/0!	#DIV/0!	#DIV/0!
DG(33:5)_C18:1	0	0	0	0	0	#DIV/0!	#DIV/0!	#DIV/0!
DG(33:2)_C18:2	0	0	0	0	0	#DIV/0!	#DIV/0!	#DIV/0!
DG(33:2)_C16:0	0	0	0	0	0	#DIV/0!	#DIV/0!	#DIV/0!
DG(33:2)_C16:1	0	0	0	0	0	#DIV/0!	#DIV/0!	#DIV/0!
DG(35:3)_C18:1	0	0	0	0	0	#DIV/0!	#DIV/0!	#DIV/0!
DG(dO-38:9),DG(35:2)_C18:1	0	0	0	0	0	#DIV/0!	#DIV/0!	#DIV/0!
DG(dO-38:9),DG(35:2)_C18:2	0	0	0	0	0	#DIV/0!	#DIV/0!	#DIV/0!
DG(O-38:8),DG(36:1)_C16:0	0	0	0	0	0	#DIV/0!	#DIV/0!	#DIV/0!
DG(38:9),DG(dO-40:9),DG(37:2	0	0	0	0	0	#DIV/0!	#DIV/0!	#DIV/0!
DG(38:8),DG(dO-40:8),DG(37:1	0	0	0	0	0	#DIV/0!	#DIV/0!	#DIV/0!
DG(38:8),DG(dO-40:8),DG(37:1	0	0	0	0	0	#DIV/0!	#DIV/0!	#DIV/0!
DG(38:7)_C18:1	0	0	0	0	0	#DIV/0!	#DIV/0!	#DIV/0!
CE(16:3)K	0	0	0	0	0	#DIV/0!	#DIV/0!	#DIV/0!
CE(16:2)K	0	0	0	0	0	#DIV/0!	#DIV/0!	#DIV/0!
DG(39:8),DG(O-40:8),DG(38:1)_	0	0	0	0	0	#DIV/0!	#DIV/0!	#DIV/0!
DG(39:8),DG(O-40:8),DG(38:1)_	0	0	0	0	0	#DIV/0!	#DIV/0!	#DIV/0!
	0	0	0	0	0	#DIV/0!	#DIV/0!	#DIV/0!
CE(17:0)K	0	0	0	0	0	#DIV/0!	#DIV/0!	#DIV/0!
DG(40:9),DG(39:2)_C18:1	0	0	0	0	0	#DIV/0!	#DIV/0!	#DIV/0!
DG(40:8),DG(39:1)_C16:0	0	0	0	0	0	#DIV/0!	#DIV/0!	#DIV/0!
DG(40:8),DG(39:1)_C16:1	0	0	0	0	0	#DIV/0!	#DIV/0!	#DIV/0!
DG(40:7)_C18:1	0	0	0	0	0	#DIV/0!	#DIV/0!	#DIV/0!
DG(40:2)_C18:1	0	0	0	0	0	#DIV/0!	#DIV/0!	#DIV/0!
DG(40:2)_C16:1	0	0	0	0	0	#DIV/0!	#DIV/0!	#DIV/0!
DG(40:1)_C18:1	0	0	0	0	0	#DIV/0!	#DIV/0!	#DIV/0!
DG(40:1)_C16:0	0	0	0	0	0	#DIV/0!	#DIV/0!	#DIV/0!
DG(40:1)_C16:1	0	0	0	0	0	#DIV/0!	#DIV/0!	#DIV/0!
DG(41:7)_C18:1	0	0	0	0	0	#DIV/0!	#DIV/0!	#DIV/0!
DG(42:10),DG(41:3)_C18:1	0	0	0	0	0	#DIV/0!	#DIV/0!	#DIV/0!
DG(42:9),DG(41:2)_C18:1	0	0	0	0	0	#DIV/0!	#DIV/0!	#DIV/0!
DG(42:9),DG(41:2)_C18:2	0	0	0	0	0	#DIV/0!	#DIV/0!	#DIV/0!
DG(42:8),DG(41:1)_C16:1	0	0	0	0	0	#DIV/0!	#DIV/0!	#DIV/0!
DG(42:7),DG(41:0)_C18:0	0	0	0	0	0	#DIV/0!	#DIV/0!	#DIV/0!
DG(42:7)_C18:1	0	0	0	0	0	#DIV/0!	#DIV/0!	#DIV/0!
DG(42:2)_C18:1	0	0	0	0	0	#DIV/0!	#DIV/0!	#DIV/0!
DG(42:2)_C16:1	0	0	0	0	0	#DIV/0!	#DIV/0!	#DIV/0!
DG(42:1)_C18:1	0	0	0	0	0	#DIV/0!	#DIV/0!	#DIV/0!
DG(42:1)_C16:0	0	0	0	0	0	#DIV/0!	#DIV/0!	#DIV/0!
DG(42:1)_C16:1	0	0	0	0	0	#DIV/0!	#DIV/0!	#DIV/0!
DG(42:0)_C18:0	0	0	0	0	0	#DIV/0!	#DIV/0!	#DIV/0!
DG(43:6)_C16:0	0	0	0	0	0	#DIV/0!	#DIV/0!	#DIV/0!

DG(44:9),DG(43:2)_C16:1	0	0	0	0	0	#DIV/0!	#DIV/0!	#DIV/0!
DG(44:8),DG(43:1)_C16:1	0	0	0	0	0	#DIV/0!	#DIV/0!	#DIV/0!
DG(44:7)_C18:1	0	0	0	0	0	#DIV/0!	#DIV/0!	#DIV/0!
DG(44:6)_C16:0	0	0	0	0	0	#DIV/0!	#DIV/0!	#DIV/0!
DG(44:2)_C18:1	0	0	0	0	0	#DIV/0!	#DIV/0!	#DIV/0!
DG(44:2)_C16:1	0	0	0	0	0	#DIV/0!	#DIV/0!	#DIV/0!
DG(44:1)_C16:0	0	0	0	0	0	#DIV/0!	#DIV/0!	#DIV/0!
DG(44:1)_C16:1	0	0	0	0	0	#DIV/0!	#DIV/0!	#DIV/0!
DG(44:0)_C18:0	0	0	0	0	0	#DIV/0!	#DIV/0!	#DIV/0!
FA(7:1)	0	0	0	0	0	#DIV/0!	#DIV/0!	#DIV/0!
FA(7:0)	0	0	0	0	0	#DIV/0!	#DIV/0!	#DIV/0!
FA(8:6)	0	0	0	0	0	#DIV/0!	#DIV/0!	#DIV/0!
FA(8:1)	0	0	0	0	0	#DIV/0!	#DIV/0!	#DIV/0!
FA(8:0)	0	0	0	0	0	#DIV/0!	#DIV/0!	#DIV/0!
FA(10:6)	0	0	0	0	0	#DIV/0!	#DIV/0!	#DIV/0!
FA(10:1)	0	0	0	0	0	#DIV/0!	#DIV/0!	#DIV/0!
FA(10:0); FA(10:0)	0	0	0	0	0	#DIV/0!	#DIV/0!	#DIV/0!
FA(11:6)	0	0	0	0	0	#DIV/0!	#DIV/0!	#DIV/0!
FA(12:5)	0	0	0	0	0	#DIV/0!	#DIV/0!	#DIV/0!
FA(12:0)	0	0	0	0	0	#DIV/0!	#DIV/0!	#DIV/0!
FA(14:7)	0	0	0	0	0	#DIV/0!	#DIV/0!	#DIV/0!
FA(13:0)	0	0	0	0	0	#DIV/0!	#DIV/0!	#DIV/0!
FA(14:0)	0	0	0	0	0	#DIV/0!	#DIV/0!	#DIV/0!
FA(15:6)	0	0	0	0	0	#DIV/0!	#DIV/0!	#DIV/0!
FA(16:5)	0	0	0	0	0	#DIV/0!	#DIV/0!	#DIV/0!
FA(17:4)	0	0	0	0	0	#DIV/0!	#DIV/0!	#DIV/0!
FA(18:7)	0	0	0	0	0	#DIV/0!	#DIV/0!	#DIV/0!
FA(17:0)	0	0	0	0	0	#DIV/0!	#DIV/0!	#DIV/0!
FA(19:4)	0	0	0	0	0	#DIV/0!	#DIV/0!	#DIV/0!
FA(20:4)	0	0	0	0	0	#DIV/0!	#DIV/0!	#DIV/0!
FA(21:3)	0	0	0	0	0	#DIV/0!	#DIV/0!	#DIV/0!
FA(22:4)	0	0	0	0	0	#DIV/0!	#DIV/0!	#DIV/0!
FA(22:3)	0	0	0	0	0	#DIV/0!	#DIV/0!	#DIV/0!
FA(28:7)	0	0	0	0	0	#DIV/0!	#DIV/0!	#DIV/0!
FA(28:5)	0	0	0	0	0	#DIV/0!	#DIV/0!	#DIV/0!
FA(29:3)	0	0	0	0	0	#DIV/0!	#DIV/0!	#DIV/0!
FA(30:5)	0	0	0	0	0	#DIV/0!	#DIV/0!	#DIV/0!
FA(31:1)	0	0	0	0	0	#DIV/0!	#DIV/0!	#DIV/0!
FA(32:6)	0	0	0	0	0	#DIV/0!	#DIV/0!	#DIV/0!
FA(32:4)	0	0	0	0	0	#DIV/0!	#DIV/0!	#DIV/0!
FA(34:5)	0	0	0	0	0	#DIV/0!	#DIV/0!	#DIV/0!
FA(46:0)	0	0	0	0	0	#DIV/0!	#DIV/0!	#DIV/0!
FA(4:0)	0	0	0	0	0	#DIV/0!	#DIV/0!	#DIV/0!
PG(32:0),PG(O-33:0); PG(32:0),	0	0	0	0	0			
						#DIV/0!	#DIV/0!	#DIV/0!

Table S6. Correlation Analysis of Fe3O4 NP-BC Lipid Abundance in Relation to Serum Concentration
BC Lipid Abundance Correlation Analysis with Female 100 nm Samples

Serum concentration (% v/v)	5	10	25	50	75			
	Group Mean Relative Lipid Abundance							
Lipid name	Female 5% 100nm	Female 10% 100nm	Female 25% 100nm	Female 50% 100nm	Female 75% 100nm	Pearson's Correlation	R^2	p-value
CE(18:0)K	0	1614.824113	3673.440261	20201.07743	32812.17842	0.98622683	0.972643	0.00194
CE(20:5) NH4	1494.576108	2452.436174	3148.644209	9629.77671	13000.85694	0.985508561	0.971227	0.00209
CE(22:6) NH4	2714.012189	2821.032201	3610.868263	8413.472541	11727.55683	0.985489026	0.971189	0.00209
DG(39:8),DG(O-40:8)_C18:2	0	1559.548106	3244.924233	17780.13334	29963.97803	0.985343574	0.970902	0.00213
CE(16:0)Na	0	2557.308183	3631.612267	12668.31692	16444.03715	0.984389415	0.969023	0.00234
DG(39:8),DG(O-40:8),DG(38:1	0	2783.940196	5968.292426	45840.839	80025.2932	0.980232523	0.960856	0.00333
DG(34:2)_C18:2	0	1422.668102	2714.02819	6633.000466	7748.34858	0.978538606	0.957538	0.00376
CE(20:5)H	3109.716226	3742.84026	6975.02054	38210.19464	51590.51551	0.976672606	0.953889	0.00426
CE(20:4) NH4	3683.496278	4330.360302	9665.636717	87562.71818	145279.2545	0.976264181	0.953092	0.00437
CE(18:3) NH4	2564.444188	2956.268207	4721.208339	27516.51801	37926.90275	0.97501086	0.950646	0.00472
CE(18:1) NH4	3032.504221	3375.436234	6128.108443	51427.95591	88221.66209	0.974793836	0.950223	0.00479
DG(34:1)_C18:1	2245.476147	2331.16416	2547.13218	6947.596465	9487.47265	0.974754465	0.950146	0.0048
DG(O-40:9),DG(38:2)_C18:2	10516.92075	13982.04908	36745.12276	450275.3913	764664.86	0.974743177	0.950124	0.0048
CE(18:2) NH4	10888.8848	15843.65712	40315.9828	492993.1167	846835.1808	0.974658342	0.949959	0.00482
CE(16:0) NH4	2899.40821	3174.380224	3507.252246	11661.05276	16085.63307	0.974483833	0.949619	0.00487
CE(19:0)H	4044.820285	4563.192324	9580.012689	93320.35096	164478.1942	0.974128465	0.948926	0.00498
CE(18:2)Na	3042.988216	3698.212254	6938.796482	37964.09486	48754.57953	0.973336063	0.947383	0.00521
DG(37:7),DG(36:0)_C16:0	3094.724214	3200.424217	3449.228238	10771.86069	16284.77709	0.972969349	0.946669	0.00531
CE(22:2) NH4	0	2028.448139	3396.756233	11157.38881	12079.38881	0.964347704	0.929966	0.00804
DG(O-38:8),DG(36:1)_C16:1	0	0	1994.612138	7101.112508	10799.85272	0.994642436	0.989314	0.00047
CE(16:1) NH4	0	0	2020.480141	7921.42459	12369.85684	0.993338313	0.986721	0.00065
CE(18:3)Na	0	0	3084.560229	12364.0809	18131.19333	0.992192789	0.984447	0.00083
CE(20:0) NH4	0	0	1834.828135	4942.268336	6536.51649	0.991505374	0.983083	0.00094
DG(O-38:9),DG(36:2)_C18:1	0	0	3141.452227	7347.080509	9575.540704	0.98946502	0.979041	0.0013
CE(16:0)K	0	0	1577.352107	6788.688474	9463.520666	0.989260591	0.978637	0.00133
CE(20:3) NH4	0	0	2155.596152	11223.18076	17317.94136	0.988711503	0.97755	0.00144
DG(34:1)_C16:0	2530.14818	1867.556137	3474.480246	7065.436499	9596.992686	0.986815701	0.973805	0.00181
CE(18:3)H	0	0	3544.128236	12963.1849	16649.04113	0.986610696	0.973401	0.00186
CE(15:1)K	0	0	3440.828245	12004.67283	15025.44905	0.984942558	0.970112	0.00221
CE(22:1)H	0	0	2928.472198	8702.524597	10644.22072	0.983983652	0.968224	0.00243
DG(36:4),DG(O-37:4)_C18:2	0	0	1356.672093	3868.112273	4586.752313	0.980386781	0.961158	0.00329
DG(36:3)_C18:1	0	0	2009.220135	5670.996391	6578.688474	0.977443982	0.955397	0.00405
CE(16:1)Na	0	0	1377.792089	5339.564378	6084.600414	0.972560908	0.945875	0.00543
DG(39:7),DG(38:0),DG(dO-40:	1977.516146	0	1528.83611	4384.580301	5219.808375	0.901735646	0.813127	0.03643
DG(40:5)_C16:0	2809.316201	3408.268242	3434.992223	3398.86023	3521.66824	0.63031526	0.397297	0.25433
DG(30:0)_C16:0	2575.444173	3283.112236	3397.656252	3432.836242	3382.004233	0.604718555	0.365685	0.27996
DG(38:5)_C16:0	3055.344219	3059.464207	2528.396191	3077.572205	3399.708235	0.517195555	0.267491	0.37215
DG(37:6)_C18:0	3454.044247	3692.888263	3258.328232	3523.004246	3700.732259	0.353155427	0.124719	0.55988
FA(15:1)	10415.90877	11437.95681	0	11239.81683	11914.43688	0.23070227	0.053224	0.70889
PC(34:3),PC(P-35:2)	0	0	0	12061.30087	20239.54949	0.967900035	0.93683	0.00687
CE(20:4)Na	0	0	0	1825.512123	3046.576229	0.967892153	0.936815	0.00687
[TG(56:8)]_C22:6	0	0	0	1399.972102	2387.312166	0.96787345	0.936779	0.00688
DG(O-38:8),DG(36:1)_C18:0	0	0	0	1851.740131	3065.59621	0.967864707	0.936762	0.00688
CE(22:5)H	0	0	0	2043.948143	3518.212252	0.967824512	0.936684	0.00689
CE(20:0)H	0	0	0	1580.432114	2782.076192	0.967609901	0.936269	0.00696
DG(O-38:9),DG(36:2)_C18:2	0	0	0	1651.128123	2942.45621	0.967435826	0.935932	0.00702
PS(P-37:0)	0	0	0	1107.500076	1985.880148	0.967333725	0.935735	0.00705
PS(38:4)	0	0	0	1123.39608	1767.496121	0.967236027	0.935546	0.00708
CE(22:3)H	0	0	0	1474.352106	2771.044193	0.966247568	0.933634	0.00741
PE(38:4)	0	0	0	2308.308167	4345.140273	0.966203258	0.933549	0.00742
CE(16:3)Na	0	0	0	2468.696181	3734.344283	0.966183303	0.93351	0.00743
CE(20:5)K	0	0	0	1385.404092	2077.032151	0.965871807	0.932908	0.00753
CE(20:2)Na	0	0	0	2298.864172	3397.952235	0.965320415	0.931844	0.00771
PC(39:7),PC(P-40:6),PC(38:0)	0	0	0	3611.780251	5176.608371	0.963878679	0.929062	0.0082
DG(39:7)_C18:1	0	0	0	3861.61227	5425.544396	0.962763939	0.926914	0.00858
PC(35:2),PC(O-36:2),PC(P-36:	0	0	0	13451.10901	18890.15738	0.962736824	0.926862	0.00859
CE(22:1) NH4	0	0	0	2073.87214	2885.528206	0.9621652	0.925762	0.00878
CE(20:0)Na	0	0	0	2035.776144	2820.696201	0.961897195	0.925246	0.00888
PC(34:2),PC(O-35:2),PC(P-35:	0	0	0	631405.9475	872954.9526	0.961755496	0.924974	0.00893
PC(38:6)	0	0	0	56874.48037	78381.54992	0.961543919	0.924567	0.009
CE(18:0) NH4	0	0	0	4082.05229	5518.056386	0.960181546	0.921949	0.00948
CE(20:2)K	0	0	0	3390.660237	4518.396331	0.959081345	0.919837	0.00987
CE(22:6)H	0	0	0	1990.636135	2633.800182	0.958500546	0.918723	0.01008
PC(39:5),PC(O-40:5),PC(P-40:	0	0	0	10274.47671	13499.81299	0.957916682	0.917604	0.0103

PE(34:2),PE(O-35:2),PE(P-35:1	0	0	0	1822.944136	2352.440164	0.956317371	0.914543	0.01089
CE(16:2)Na	0	0	0	3610.06825	4649.980301	0.956144552	0.914212	0.01095
CE(20:5)Na	0	0	0	3728.868285	4712.480327	0.954301428	0.910691	0.01165
DG(34:2)_C16:0	0	0	0	5371.376361	6757.208521	0.953835757	0.909803	0.01182
CE(18:1)Na	0	0	0	5722.148435	6869.228467	0.948594068	0.899831	0.01388
CE(19:0) NH4	0	0	0	3389.32424	4013.456283	0.946893754	0.896608	0.01457
CE(18:1)K	0	0	0	3768.288274	4430.964319	0.945991148	0.894899	0.01494
CE(19:0)Na	0	0	0	3976.632291	4592.104321	0.943573893	0.890332	0.01595
CE(20:4)H	0	0	0	5716.096395	6598.444476	0.943525091	0.89024	0.01597
DG(40:9),DG(39:2)_C18:2	0	0	0	2893.092207	3333.032238	0.943250604	0.889722	0.01609
CE(20:1) NH4	0	0	0	3502.148244	4003.212299	0.942154697	0.887655	0.01656
DG(36:3)_C18:2	0	0	0	5251.676416	5930.860413	0.940413415	0.884377	0.0173
CE(22:5) NH4	0	0	0	3656.136259	4088.144304	0.938938482	0.881605	0.01795
DG(O-40:9),DG(38:2)_C18:1	0	0	0	2805.876202	3136.256211	0.938882747	0.881501	0.01797
CE(19:0)K	0	0	0	1503.856097	1670.124132	0.937903191	0.879662	0.0184
DG(38:3)_C18:2	0	0	0	3395.564256	3735.252254	0.93642618	0.876894	0.01906
DG(O-38:8),DG(36:1)_C18:1	0	0	0	3030.624218	3330.116244	0.936252076	0.876568	0.01914
CE(14:0) NH4	0	0	0	2261.264166	2455.104169	0.934333669	0.872979	0.02
CE(20:2) NH4	0	0	0	2006.376144	2177.584154	0.934275305	0.87287	0.02003
CE(22:4)Na	0	0	0	3862.900272	4115.208302	0.931180248	0.867097	0.02145
CE(22:3) NH4	0	0	0	3012.280212	3203.388235	0.930880513	0.866539	0.02159
DG(37:6)_C16:0	0	0	0	1607.116118	1707.740118	0.930746871	0.86629	0.02165
CE(18:2)K	0	0	0	5109.584351	5369.064382	0.928807458	0.862683	0.02256
DG(34:0)_C18:0	123407.073	114875.6121	114009.2522	122594.7568	137995.0929	0.771363122	0.595001	0.12664
DG(34:0)_C16:0	96790.89475	88773.65833	88124.39824	96795.11155	107401.8443	0.763620904	0.583117	0.13296
DG(37:7),DG(36:0)_C18:0	65507.91643	60805.54025	59581.77622	63102.93671	77501.51777	0.734541981	0.539552	0.15748
DG(32:0)_C16:0	155887.1476	149713.2267	142234.1538	154216.0394	177407.0481	0.732125708	0.536008	0.15957
DG(32:0)_C18:0	3157.164219	3117.924224	3050.320211	3130.684227	3361.416222	0.718605194	0.516393	0.17142
DG(35:6)_C18:0	4594.76833	4249.18431	4217.272285	4301.188311	5060.440346	0.614012602	0.377011	0.27058
DG(36:7),DG(35:0)_C18:0	2329.592168	0	1773.668128	2799.332195	2241.684164	0.490281613	0.240376	0.40175
DG(32:2)_C18:1	2523.288179	2865.884209	1750.452117	2861.980185	2311.188152	-0.089465155	0.008004	0.88624
PG(16:0),LPG(17:0),LPG(O-18:	3141.464215	2700.808198	1614.848106	2321.436175	3014.360213			
CE(18:3)K	0	0	0	1810.460132	1803.788125	0.918910577	0.844397	0.02738
DG(34:2)_C18:1	0	0	0	2775.764198	2376.756173	0.88504445	0.783304	0.04597
SM(d18:2/18:1)	0	0	0	0	3435.03224	0.801783726	0.642857	0.10273
[TG(53:10),TG(52:3)]_C18:1	0	0	0	0	135842.006	0.801783726	0.642857	0.10273
[TG(55:9),TG(54:2)]_C18:1	0	0	0	0	19582.83733	0.801783726	0.642857	0.10273
[TG(55:10),TG(54:3)]_C18:2	0	0	0	0	15486.19294	0.801783726	0.642857	0.10273
SM(d18:1/19:0)	0	0	0	0	8676.448701	0.801783726	0.642857	0.10273
[TG(56:11),TG(55:4)]_C18:2	0	0	0	0	1173.19608	0.801783726	0.642857	0.10273
PE(38:5)	0	0	0	0	1311.004092	0.801783726	0.642857	0.10273
[TG(53:9),TG(52:2)]_C18:2	0	0	0	0	21430.10548	0.801783726	0.642857	0.10273
SM(d18:2/20:1)	0	0	0	0	18530.91727	0.801783726	0.642857	0.10273
[TG(52:4)]_C16:1	0	0	0	0	10615.2128	0.801783726	0.642857	0.10273
SM(d16:0/23:0)	0	0	0	0	7946.408546	0.801783726	0.642857	0.10273
CE(22:2)H	0	0	0	0	2443.780171	0.801783726	0.642857	0.10273
DG(32:1)_C16:0	0	0	0	0	2119.560151	0.801783726	0.642857	0.10273
CE(20:3)Na	0	0	0	0	2116.908154	0.801783726	0.642857	0.10273
DG(34:3)_C18:2	0	0	0	0	2066.716145	0.801783726	0.642857	0.10273
SM(d18:0/26:1(17Z))	0	0	0	0	1744.460119	0.801783726	0.642857	0.10273
CE(20:1)K	0	0	0	0	1586.484114	0.801783726	0.642857	0.10273
CE(15:0)K	0	0	0	0	1441.912103	0.801783726	0.642857	0.10273
PE(O-38:8),PE(36:1),PE(O-37::	0	0	0	0	1338.1201	0.801783726	0.642857	0.10273
PE(38:6)	0	0	0	0	1299.356094	0.801783726	0.642857	0.10273
[TG(52:4)]_C18:2	0	0	0	0	83111.12103	0.801783726	0.642857	0.10273
[TG(55:10),TG(54:3)]_C18:1	0	0	0	0	77526.26597	0.801783726	0.642857	0.10273
[TG(55:11),TG(54:4)]_C18:1	0	0	0	0	59510.329	0.801783726	0.642857	0.10273
[TG(52:4)]_C16:0	0	0	0	0	48397.22366	0.801783726	0.642857	0.10273
[TG(53:8),TG(52:1)]_C18:1	0	0	0	0	25509.77365	0.801783726	0.642857	0.10273
PC(32:2),PC(O-33:2),PC(P-33:	0	0	0	0	18064.88522	0.801783726	0.642857	0.10273
[TG(53:9),TG(52:2)]_C18:0	0	0	0	0	17228.29714	0.801783726	0.642857	0.10273
FA(14:2)	0	0	0	0	11354.13282	0.801783726	0.642857	0.10273
PC(33:1),PC(O-34:1),PC(P-34:	0	0	0	0	8851.592577	0.801783726	0.642857	0.10273
LPC(18:2),LPC(P-19:1)	0	0	0	0	6033.304446	0.801783726	0.642857	0.10273
[TG(55:8),TG(54:1)]_C18:0	0	0	0	0	5598.944404	0.801783726	0.642857	0.10273
[TG(55:8),TG(54:1)]_C18:1	0	0	0	0	4421.784305	0.801783726	0.642857	0.10273
[TG(52:5)]_C16:1	0	0	0	0	3344.968239	0.801783726	0.642857	0.10273
[TG(57:11),TG(56:4)]_C18:1	0	0	0	0	2447.132157	0.801783726	0.642857	0.10273
[TG(56:6)]_C18:2	0	0	0	0	2371.920173	0.801783726	0.642857	0.10273
PE(34:1),PE(O-35:1),PE(P-35:(	0	0	0	0	2196.300154	0.801783726	0.642857	0.10273
SM(d18:2/14:0)	0	0	0	0	2191.952155	0.801783726	0.642857	0.10273

[TG(56:6)]_C22:5	0	0	0	0	2081.480151	0.801783726	0.642857	0.10273
[TG(57:12),TG(56:5)]_C20:4	0	0	0	0	2056.696157	0.801783726	0.642857	0.10273
DG(37:7)_C16:1	0	0	0	0	1736.184124	0.801783726	0.642857	0.10273
CE(22:4) NH4	0	0	0	0	1463.248106	0.801783726	0.642857	0.10273
[TG(58:8)]_C22:6	0	0	0	0	986.5840744	0.801783726	0.642857	0.10273
SM(d16:1/22:0)	0	0	0	0	296653.725	0.801783726	0.642857	0.10273
[TG(53:10),TG(52:3)]_C16:0	0	0	0	0	106858.8077	0.801783726	0.642857	0.10273
[TG(55:9),TG(54:2)]_C18:0	0	0	0	0	15367.83712	0.801783726	0.642857	0.10273
CE(17:1) NH4	0	0	0	0	2213.08415	0.801783726	0.642857	0.10273
[TG(53:8)]_C18:2	0	0	0	0	1492.140105	0.801783726	0.642857	0.10273
LPE(20:4)	0	0	0	0	1448.240105	0.801783726	0.642857	0.10273
[TG(53:8),TG(52:1)]_C18:0	0	0	0	0	24381.3335	0.801783726	0.642857	0.10273
[TG(56:7)]_C22:5	0	0	0	0	1861.596136	0.801783726	0.642857	0.10273
[TG(54:8),TG(53:1)]_C18:1	0	0	0	0	1179.332085	0.801783726	0.642857	0.10273
SM(d16:1/22:1)	0	0	0	0	24034.31362	0.801783726	0.642857	0.10273
PC(33:2),PC(O-34:2),PC(P-34:	0	0	0	0	11238.97276	0.801783726	0.642857	0.10273
[TG(53:10),TG(52:3)]_C16:1	0	0	0	0	7587.384577	0.801783726	0.642857	0.10273
DG(34:2)_C16:1	0	0	0	0	2578.328185	0.801783726	0.642857	0.10273
DG(O-38:9),DG(36:2)_C18:0	0	0	0	0	2234.384163	0.801783726	0.642857	0.10273
PC(41:7),PC(P-42:6),PC(40:0)	0	0	0	0	1634.864117	0.801783726	0.642857	0.10273
CE(22:4)K	0	0	0	0	1456.208104	0.801783726	0.642857	0.10273
[TG(56:11),TG(55:4)]_C18:1	0	0	0	0	1106.17608	0.801783726	0.642857	0.10273
[TG(53:10),TG(52:3)]_C18:2	0	0	0	0	125865.4126	0.801783726	0.642857	0.10273
[TG(54:5)]_C18:2	0	0	0	0	31881.45016	0.801783726	0.642857	0.10273
[TG(52:4)]_C18:1	0	0	0	0	15066.62919	0.801783726	0.642857	0.10273
LPC(20:4)	0	0	0	0	4440.732294	0.801783726	0.642857	0.10273
CAR(10:2)	0	0	0	0	2081.024156	0.801783726	0.642857	0.10273
[TG(53:7)]_C18:1	0	0	0	0	2007.284136	0.801783726	0.642857	0.10273
DG(40:2)_C18:2	0	0	0	0	1938.808142	0.801783726	0.642857	0.10273
[TG(56:7),TG(55:0)]_C16:0	0	0	0	0	1767.748122	0.801783726	0.642857	0.10273
DG(34:3)_C16:1	0	0	0	0	1405.172099	0.801783726	0.642857	0.10273
PC(27:0),PC(O-28:0)	0	0	0	0	1383.724107	0.801783726	0.642857	0.10273
PC(33:3),PC(O-34:3),PC(P-34:	0	0	0	0	10218.8247	0.801783726	0.642857	0.10273
PC(36:4),PC(O-37:4)	0	0	0	0	230523.0849	0.801783726	0.642857	0.10273
[TG(52:5)]_C18:2	0	0	0	0	9294.048649	0.801783726	0.642857	0.10273
FA(20:0)	19380.21341	24632.4256	0	0	0	-0.776475735	0.602915	0.12252
DG(34:3)_C18:1	4072.548295	3869.716274	3928.424273	3915.720267	3748.572263	-0.765160524	0.585471	0.13169
DG(40:5)_C18:0	2735.560194	2482.664178	2711.340192	2680.776182	2057.376136	-0.702699686	0.493787	0.18567
DG(42:5)_C18:0	2820.080194	2815.064203	2667.248181	1608.852112	2646.044189	-0.443776463	0.196938	0.4541
DG(35:6)_C16:0	4345.04831	4193.032282	4167.7803	4091.508298	4411.512322	0.219298931	0.048092	0.72303
LPG(19:0),LPG(O-20:0); LPG(1	3756.520259	2472.736189	0	2230.064178	2130.384129	-0.218952342	0.04794	0.72346
PG(20:0),LPG(21:0); PG(20:0),	0	0	1317.644089	0	0	-0.15272071	0.023324	0.80631
DG(33:0)_C16:0	3158.904217	3007.324216	2814.456192	3109.37223	3069.164202	0.104204152	0.010859	0.86756
DG(34:4),DG(dO-36:4)_C16:1	4520.436295	4187.716313	3628.224238	3278.35624	3158.644215	-0.925940398	0.857366	0.02392
FA(22:7)	18565.01729	16703.1491	0	0	0	-0.797201588	0.63553	0.10623
FA(17:2)	6940.696507	6293.720448	0	0	0	-0.797160532	0.635465	0.10626
FA(21:0)	19194.17341	17639.45725	0	0	0	-0.797043693	0.635279	0.10635
FA(6:0)	13036.65693	0	0	0	0	-0.534522484	0.285714	0.35339
FA(18:0)	32335.31011	0	0	0	0	-0.534522484	0.285714	0.35339
FA(19:2)	7889.460632	0	0	0	0	-0.534522484	0.285714	0.35339
[TG(53:9),TG(52:2)]_C18:1	0	0	0	0	0	#DIV/0!	#DIV/0!	#DIV/0!
[TG(54:5)]_C18:1	0	0	0	0	0	#DIV/0!	#DIV/0!	#DIV/0!
[TG(55:10),TG(54:3)]_C18:0	0	0	0	0	0	#DIV/0!	#DIV/0!	#DIV/0!
[TG(53:9),TG(52:2)]_C16:0	0	0	0	0	0	#DIV/0!	#DIV/0!	#DIV/0!
[TG(53:8),TG(52:1)]_C16:0	0	0	0	0	0	#DIV/0!	#DIV/0!	#DIV/0!
[TG(55:11),TG(54:4)]_C18:2	0	0	0	0	0	#DIV/0!	#DIV/0!	#DIV/0!
[TG(51:7),TG(50:0)]_C18:0	0	0	0	0	0	#DIV/0!	#DIV/0!	#DIV/0!
[TG(49:7),TG(48:0)]_C16:0	0	0	0	0	0	#DIV/0!	#DIV/0!	#DIV/0!
[TG(51:9),TG(50:2)]_C18:1	0	0	0	0	0	#DIV/0!	#DIV/0!	#DIV/0!
[TG(51:7),TG(50:0)]_C16:0	0	0	0	0	0	#DIV/0!	#DIV/0!	#DIV/0!
[TG(51:9),TG(50:2)]_C18:2	0	0	0	0	0	#DIV/0!	#DIV/0!	#DIV/0!
[TG(51:9),TG(50:2)]_C16:0	0	0	0	0	0	#DIV/0!	#DIV/0!	#DIV/0!
[TG(51:8),TG(50:1)]_C18:1	0	0	0	0	0	#DIV/0!	#DIV/0!	#DIV/0!
[TG(54:6)]_C18:2	0	0	0	0	0	#DIV/0!	#DIV/0!	#DIV/0!
[TG(49:8),TG(48:1)]_C16:0	0	0	0	0	0	#DIV/0!	#DIV/0!	#DIV/0!
[TG(51:8),TG(50:1)]_C16:0	0	0	0	0	0	#DIV/0!	#DIV/0!	#DIV/0!
PS(O-29:0)	0	0	0	0	0	#DIV/0!	#DIV/0!	#DIV/0!
[TG(46:0)]_C16:0	0	0	0	0	0	#DIV/0!	#DIV/0!	#DIV/0!
[TG(50:3)]_C18:1	0	0	0	0	0	#DIV/0!	#DIV/0!	#DIV/0!
[TG(50:3)]_C18:2	0	0	0	0	0	#DIV/0!	#DIV/0!	#DIV/0!
[TG(51:8),TG(50:1)]_C18:0	0	0	0	0	0	#DIV/0!	#DIV/0!	#DIV/0!

[TG(53:7),TG(52:0)]_C18:0	0	0	0	0	0	#DIV/0!	#DIV/0!	#DIV/0!
PC(34:1),PC(O-35:1),PC(P-35:	0	0	0	0	0	#DIV/0!	#DIV/0!	#DIV/0!
[TG(50:3)]_C16:0	0	0	0	0	0	#DIV/0!	#DIV/0!	#DIV/0!
PC(32:0),PC(O-33:0)	0	0	0	0	0	#DIV/0!	#DIV/0!	#DIV/0!
[TG(50:3)]_C16:1	0	0	0	0	0	#DIV/0!	#DIV/0!	#DIV/0!
[TG(51:9),TG(50:2)]_C16:1	0	0	0	0	0	#DIV/0!	#DIV/0!	#DIV/0!
[TG(55:11),TG(54:4)]_C18:0	0	0	0	0	0	#DIV/0!	#DIV/0!	#DIV/0!
[TG(50:7),TG(49:0)]_C16:0	0	0	0	0	0	#DIV/0!	#DIV/0!	#DIV/0!
[TG(53:7),TG(52:0)]_C16:0	0	0	0	0	0	#DIV/0!	#DIV/0!	#DIV/0!
PC(40:6)	0	0	0	0	0	#DIV/0!	#DIV/0!	#DIV/0!
[TG(54:10),TG(53:3)]_C18:1	0	0	0	0	0	#DIV/0!	#DIV/0!	#DIV/0!
PC(36:3),PC(P-37:2)	0	0	0	0	0	#DIV/0!	#DIV/0!	#DIV/0!
[TG(52:4)]_C18:3	0	0	0	0	0	#DIV/0!	#DIV/0!	#DIV/0!
SM(d16:0/22:0)	0	0	0	0	0	#DIV/0!	#DIV/0!	#DIV/0!
PC(O-38:9),PC(36:2),PC(O-37	0	0	0	0	0	#DIV/0!	#DIV/0!	#DIV/0!
PC(36:5)	0	0	0	0	0	#DIV/0!	#DIV/0!	#DIV/0!
[TG(51:9),TG(50:2)]_C14:0	0	0	0	0	0	#DIV/0!	#DIV/0!	#DIV/0!
[TG(54:9),TG(53:2)]_C18:1	0	0	0	0	0	#DIV/0!	#DIV/0!	#DIV/0!
[TG(50:3)]_C14:0	0	0	0	0	0	#DIV/0!	#DIV/0!	#DIV/0!
[TG(52:9),TG(51:2)]_C18:1	0	0	0	0	0	#DIV/0!	#DIV/0!	#DIV/0!
[TG(49:8),TG(48:1)]_C18:1	0	0	0	0	0	#DIV/0!	#DIV/0!	#DIV/0!
[TG(39:0)]_C20:0	0	0	0	0	0	#DIV/0!	#DIV/0!	#DIV/0!
PC(38:4)	0	0	0	0	0	#DIV/0!	#DIV/0!	#DIV/0!
PC(38:5)	0	0	0	0	0	#DIV/0!	#DIV/0!	#DIV/0!
[TG(52:5)]_C18:3	0	0	0	0	0	#DIV/0!	#DIV/0!	#DIV/0!
[TG(51:7)]_C18:1	0	0	0	0	0	#DIV/0!	#DIV/0!	#DIV/0!
SM(d18:2/22:1)	0	0	0	0	0	#DIV/0!	#DIV/0!	#DIV/0!
LPG(20:0); LPG(20:0)	0	0	0	0	0	#DIV/0!	#DIV/0!	#DIV/0!
[TG(50:4)]_C18:2	0	0	0	0	0	#DIV/0!	#DIV/0!	#DIV/0!
[TG(54:5)]_C18:3	0	0	0	0	0	#DIV/0!	#DIV/0!	#DIV/0!
PC(40:4)	0	0	0	0	0	#DIV/0!	#DIV/0!	#DIV/0!
[TG(52:5)]_C16:0	0	0	0	0	0	#DIV/0!	#DIV/0!	#DIV/0!
[TG(48:2)]_C16:0	0	0	0	0	0	#DIV/0!	#DIV/0!	#DIV/0!
[TG(57:12),TG(56:5)]_C18:1	0	0	0	0	0	#DIV/0!	#DIV/0!	#DIV/0!
PC(37:5),PC(O-38:5),PC(P-38:	0	0	0	0	0	#DIV/0!	#DIV/0!	#DIV/0!
[TG(54:6)]_C18:1	0	0	0	0	0	#DIV/0!	#DIV/0!	#DIV/0!
PC(34:0),PC(O-35:0)	0	0	0	0	0	#DIV/0!	#DIV/0!	#DIV/0!
[TG(54:6)]_C20:4	0	0	0	0	0	#DIV/0!	#DIV/0!	#DIV/0!
SM(d16:1/16:0)	0	0	0	0	0	#DIV/0!	#DIV/0!	#DIV/0!
[TG(48:2)]_C18:2	0	0	0	0	0	#DIV/0!	#DIV/0!	#DIV/0!
[TG(51:8),TG(50:1)]_C16:1	0	0	0	0	0	#DIV/0!	#DIV/0!	#DIV/0!
PC(28:1),PC(P-29:0)	0	0	0	0	0	#DIV/0!	#DIV/0!	#DIV/0!
PC(37:3),PC(O-38:3),PC(P-38:	0	0	0	0	0	#DIV/0!	#DIV/0!	#DIV/0!
PC(40:10),PC(39:3),PC(O-40:3	0	0	0	0	0	#DIV/0!	#DIV/0!	#DIV/0!
SM(d16:1/20:1)	0	0	0	0	0	#DIV/0!	#DIV/0!	#DIV/0!
PC(37:4),PC(O-38:4),PC(P-38:	0	0	0	0	0	#DIV/0!	#DIV/0!	#DIV/0!
PC(30:2),PC(P-31:1)	0	0	0	0	0	#DIV/0!	#DIV/0!	#DIV/0!
SM(d18:2/24:1)	0	0	0	0	0	#DIV/0!	#DIV/0!	#DIV/0!
[TG(54:5)]_C20:4	0	0	0	0	0	#DIV/0!	#DIV/0!	#DIV/0!
SM(d16:1/18:0)	0	0	0	0	0	#DIV/0!	#DIV/0!	#DIV/0!
SM(d16:1/24:1)	0	0	0	0	0	#DIV/0!	#DIV/0!	#DIV/0!
PC(38:3)	0	0	0	0	0	#DIV/0!	#DIV/0!	#DIV/0!
SM(d16:0/18:0)	0	0	0	0	0	#DIV/0!	#DIV/0!	#DIV/0!
PC(40:5)	0	0	0	0	0	#DIV/0!	#DIV/0!	#DIV/0!
SM(d16:1/24:0)	0	0	0	0	0	#DIV/0!	#DIV/0!	#DIV/0!
PC(30:1),PC(O-31:1),PC(P-31:	0	0	0	0	0	#DIV/0!	#DIV/0!	#DIV/0!
PC(O-38:8),PC(36:1),PC(O-37	0	0	0	0	0	#DIV/0!	#DIV/0!	#DIV/0!
PC(30:0),PC(O-31:0)	0	0	0	0	0	#DIV/0!	#DIV/0!	#DIV/0!
SM(d16:1/17:0)	0	0	0	0	0	#DIV/0!	#DIV/0!	#DIV/0!
[TG(55:11),TG(54:4)]_C16:0	0	0	0	0	0	#DIV/0!	#DIV/0!	#DIV/0!
PC(35:4),PC(O-36:4),PC(P-36:	0	0	0	0	0	#DIV/0!	#DIV/0!	#DIV/0!
SM(d16:0/20:0)	0	0	0	0	0	#DIV/0!	#DIV/0!	#DIV/0!
SM(d16:1/20:0)	0	0	0	0	0	#DIV/0!	#DIV/0!	#DIV/0!
PC(29:1),PC(O-30:1),PC(P-30:	0	0	0	0	0	#DIV/0!	#DIV/0!	#DIV/0!
SM(d16:1/18:1)	0	0	0	0	0	#DIV/0!	#DIV/0!	#DIV/0!
PC(31:0),PC(O-32:0)	0	0	0	0	0	#DIV/0!	#DIV/0!	#DIV/0!
[TG(49:8),TG(48:1)]_C14:0	0	0	0	0	0	#DIV/0!	#DIV/0!	#DIV/0!
[TG(49:6)]_C16:0	0	0	0	0	0	#DIV/0!	#DIV/0!	#DIV/0!
[TG(49:7),TG(48:0)]_C18:0	0	0	0	0	0	#DIV/0!	#DIV/0!	#DIV/0!
[TG(56:6)]_C20:4	0	0	0	0	0	#DIV/0!	#DIV/0!	#DIV/0!
PC(28:0),PC(O-29:0)	0	0	0	0	0	#DIV/0!	#DIV/0!	#DIV/0!

[TG(48:2)]_C18:1	0	0	0	0	0	#DIV/0!	#DIV/0!	#DIV/0!
[TG(55:9),TG(54:2)]_C16:0	0	0	0	0	0	#DIV/0!	#DIV/0!	#DIV/0!
[TG(55:9),TG(54:2)]_C18:2	0	0	0	0	0	#DIV/0!	#DIV/0!	#DIV/0!
[TG(56:7)]_C22:6	0	0	0	0	0	#DIV/0!	#DIV/0!	#DIV/0!
[TG(52:10),TG(51:3)]_C18:2	0	0	0	0	0	#DIV/0!	#DIV/0!	#DIV/0!
[TG(52:9),TG(51:2)]_C16:0	0	0	0	0	0	#DIV/0!	#DIV/0!	#DIV/0!
PC(38:9),PC(37:2),PC(O-38:2)	0	0	0	0	0	#DIV/0!	#DIV/0!	#DIV/0!
PC(36:8),PC(35:1),PC(O-36:1)	0	0	0	0	0	#DIV/0!	#DIV/0!	#DIV/0!
[TG(57:10),TG(56:3)]_C18:1	0	0	0	0	0	#DIV/0!	#DIV/0!	#DIV/0!
[TG(54:10),TG(53:3)]_C18:2	0	0	0	0	0	#DIV/0!	#DIV/0!	#DIV/0!
[TG(50:4)]_C16:1	0	0	0	0	0	#DIV/0!	#DIV/0!	#DIV/0!
PC(35:3),PC(O-36:3),PC(P-36:	0	0	0	0	0	#DIV/0!	#DIV/0!	#DIV/0!
PC(31:1),PC(O-32:1),PC(P-32:	0	0	0	0	0	#DIV/0!	#DIV/0!	#DIV/0!
PC(32:1),PC(O-33:1),PC(P-33:	0	0	0	0	0	#DIV/0!	#DIV/0!	#DIV/0!
SM(d16:0/24:0)	0	0	0	0	0	#DIV/0!	#DIV/0!	#DIV/0!
[TG(48:2)]_C16:1	0	0	0	0	0	#DIV/0!	#DIV/0!	#DIV/0!
[TG(49:8),TG(48:1)]_C16:1	0	0	0	0	0	#DIV/0!	#DIV/0!	#DIV/0!
[TG(52:8),TG(51:1)]_C16:0	0	0	0	0	0	#DIV/0!	#DIV/0!	#DIV/0!
[TG(56:7)]_C20:4	0	0	0	0	0	#DIV/0!	#DIV/0!	#DIV/0!
[TG(51:8),TG(50:1)]_C14:0	0	0	0	0	0	#DIV/0!	#DIV/0!	#DIV/0!
PC(40:8),PC(39:1),PC(O-40:1)	0	0	0	0	0	#DIV/0!	#DIV/0!	#DIV/0!
[TG(53:9),TG(52:2)]_C16:1	0	0	0	0	0	#DIV/0!	#DIV/0!	#DIV/0!
[TG(53:10),TG(52:3)]_C18:0	0	0	0	0	0	#DIV/0!	#DIV/0!	#DIV/0!
[TG(55:10),TG(54:3)]_C16:0	0	0	0	0	0	#DIV/0!	#DIV/0!	#DIV/0!
[TG(56:7)]_C18:2	0	0	0	0	0	#DIV/0!	#DIV/0!	#DIV/0!
LPC(18:0),PC(O-18:0),LPC(O-:	0	0	0	0	0	#DIV/0!	#DIV/0!	#DIV/0!
[TG(48:2)]_C14:0	0	0	0	0	0	#DIV/0!	#DIV/0!	#DIV/0!
[TG(54:6)]_C18:3	0	0	0	0	0	#DIV/0!	#DIV/0!	#DIV/0!
PC(37:7),PC(P-38:6),PC(36:0),	0	0	0	0	0	#DIV/0!	#DIV/0!	#DIV/0!
[TG(54:5)]_C16:0	0	0	0	0	0	#DIV/0!	#DIV/0!	#DIV/0!
SM(d18:1/17:0)	0	0	0	0	0	#DIV/0!	#DIV/0!	#DIV/0!
Cer(d18:1/24:0)	0	0	0	0	0	#DIV/0!	#DIV/0!	#DIV/0!
[TG(49:7),TG(48:0)]_C14:0	0	0	0	0	0	#DIV/0!	#DIV/0!	#DIV/0!
[TG(46:2)]_C18:1	0	0	0	0	0	#DIV/0!	#DIV/0!	#DIV/0!
[TG(50:8),TG(49:1)]_C18:1	0	0	0	0	0	#DIV/0!	#DIV/0!	#DIV/0!
[TG(48:3)]_C16:1	0	0	0	0	0	#DIV/0!	#DIV/0!	#DIV/0!
[TG(54:11),TG(53:4)]_C18:2	0	0	0	0	0	#DIV/0!	#DIV/0!	#DIV/0!
[TG(57:11),TG(56:4)]_C18:2	0	0	0	0	0	#DIV/0!	#DIV/0!	#DIV/0!
[TG(50:4)]_C14:0	0	0	0	0	0	#DIV/0!	#DIV/0!	#DIV/0!
SM(d18:1/24:1(15Z))	0	0	0	0	0	#DIV/0!	#DIV/0!	#DIV/0!
PC(O-40:9),PC(38:2),PC(P-39:	0	0	0	0	0	#DIV/0!	#DIV/0!	#DIV/0!
PC(41:6),PC(O-42:6)	0	0	0	0	0	#DIV/0!	#DIV/0!	#DIV/0!
[TG(57:12),TG(56:5)]_C18:2	0	0	0	0	0	#DIV/0!	#DIV/0!	#DIV/0!
[TG(56:10),TG(55:3)]_C18:1	0	0	0	0	0	#DIV/0!	#DIV/0!	#DIV/0!
LPC(20:2),PC(O-20:2)	0	0	0	0	0	#DIV/0!	#DIV/0!	#DIV/0!
[TG(52:8),TG(51:1)]_C18:1	0	0	0	0	0	#DIV/0!	#DIV/0!	#DIV/0!
SM(d17:1/24:1)	0	0	0	0	0	#DIV/0!	#DIV/0!	#DIV/0!
SM(d17:1/26:1)	0	0	0	0	0	#DIV/0!	#DIV/0!	#DIV/0!
PC(41:5),PC(P-42:4)	0	0	0	0	0	#DIV/0!	#DIV/0!	#DIV/0!
PC(42:3)	0	0	0	0	0	#DIV/0!	#DIV/0!	#DIV/0!
PC(44:12),PC(O-44:5)	0	0	0	0	0	#DIV/0!	#DIV/0!	#DIV/0!
[TG(52:10),TG(51:3)]_C18:1	0	0	0	0	0	#DIV/0!	#DIV/0!	#DIV/0!
PC(39:4),PC(O-40:4),PC(P-40:	0	0	0	0	0	#DIV/0!	#DIV/0!	#DIV/0!
[TG(54:7)]_C18:2	0	0	0	0	0	#DIV/0!	#DIV/0!	#DIV/0!
[TG(48:3)]_C18:2	0	0	0	0	0	#DIV/0!	#DIV/0!	#DIV/0!
[TG(52:4)]_C20:4	0	0	0	0	0	#DIV/0!	#DIV/0!	#DIV/0!
[TG(50:3)]_C18:3	0	0	0	0	0	#DIV/0!	#DIV/0!	#DIV/0!
[TG(54:7)]_C18:3	0	0	0	0	0	#DIV/0!	#DIV/0!	#DIV/0!
[TG(50:9),TG(49:2)]_C18:2	0	0	0	0	0	#DIV/0!	#DIV/0!	#DIV/0!
[TG(51:9),TG(50:2)]_C18:0	0	0	0	0	0	#DIV/0!	#DIV/0!	#DIV/0!
[TG(54:10),TG(53:3)]_C16:0	0	0	0	0	0	#DIV/0!	#DIV/0!	#DIV/0!
[TG(52:5)]_C18:1	0	0	0	0	0	#DIV/0!	#DIV/0!	#DIV/0!
PC(40:3)	0	0	0	0	0	#DIV/0!	#DIV/0!	#DIV/0!
[TG(55:8),TG(54:1)]_C16:0	0	0	0	0	0	#DIV/0!	#DIV/0!	#DIV/0!
SM(d16:0/16:0)	0	0	0	0	0	#DIV/0!	#DIV/0!	#DIV/0!
SM(d18:0/17:0)	0	0	0	0	0	#DIV/0!	#DIV/0!	#DIV/0!
[TG(56:6)]_C16:0	0	0	0	0	0	#DIV/0!	#DIV/0!	#DIV/0!
[TG(57:11),TG(56:4)]_C18:0	0	0	0	0	0	#DIV/0!	#DIV/0!	#DIV/0!
PC(29:0),PC(O-30:0)	0	0	0	0	0	#DIV/0!	#DIV/0!	#DIV/0!
[TG(46:1)]_C18:1	0	0	0	0	0	#DIV/0!	#DIV/0!	#DIV/0!
[TG(46:0)]_C14:0	0	0	0	0	0	#DIV/0!	#DIV/0!	#DIV/0!

[TG(52:6)]_C18:3	0	0	0	0	0	#DIV/0!	#DIV/0!	#DIV/0!
[TG(50:8),TG(49:1)]_C16:0	0	0	0	0	0	#DIV/0!	#DIV/0!	#DIV/0!
[TG(57:12),TG(56:5)]_C16:0	0	0	0	0	0	#DIV/0!	#DIV/0!	#DIV/0!
[TG(46:1)]_C16:0	0	0	0	0	0	#DIV/0!	#DIV/0!	#DIV/0!
[TG(54:9),TG(53:2)]_C18:0	0	0	0	0	0	#DIV/0!	#DIV/0!	#DIV/0!
SM(d16:0/25:0)	0	0	0	0	0	#DIV/0!	#DIV/0!	#DIV/0!
PC(40:7),PC(39:0),PC(O-40:0)	0	0	0	0	0	#DIV/0!	#DIV/0!	#DIV/0!
PC(38:7),PC(37:0),PC(O-38:0)	0	0	0	0	0	#DIV/0!	#DIV/0!	#DIV/0!
PC(42:4)	0	0	0	0	0	#DIV/0!	#DIV/0!	#DIV/0!
[TG(54:11),TG(53:4)]_C18:1	0	0	0	0	0	#DIV/0!	#DIV/0!	#DIV/0!
SM(d18:2/21:0)	0	0	0	0	0	#DIV/0!	#DIV/0!	#DIV/0!
PC(40:1),PC(P-41:0)	0	0	0	0	0	#DIV/0!	#DIV/0!	#DIV/0!
[TG(56:8)]_C18:2	0	0	0	0	0	#DIV/0!	#DIV/0!	#DIV/0!
PC(31:2),PC(O-32:2),PC(P-32:	0	0	0	0	0	#DIV/0!	#DIV/0!	#DIV/0!
PC(39:6),PC(O-40:6),PC(P-40:	0	0	0	0	0	#DIV/0!	#DIV/0!	#DIV/0!
PC(37:6),PC(O-38:6),PC(P-38:	0	0	0	0	0	#DIV/0!	#DIV/0!	#DIV/0!
[TG(51:8)]_C18:2	0	0	0	0	0	#DIV/0!	#DIV/0!	#DIV/0!
PC(38:8),PC(37:1),PC(O-38:1)	0	0	0	0	0	#DIV/0!	#DIV/0!	#DIV/0!
PC(36:7),PC(35:0),PC(O-36:0)	0	0	0	0	0	#DIV/0!	#DIV/0!	#DIV/0!
[TG(57:10),TG(56:3)]_C18:2	0	0	0	0	0	#DIV/0!	#DIV/0!	#DIV/0!
[TG(51:4)]_C18:2	0	0	0	0	0	#DIV/0!	#DIV/0!	#DIV/0!
PI(38:4)	0	0	0	0	0	#DIV/0!	#DIV/0!	#DIV/0!
SM(d18:1/26:1(17Z))	0	0	0	0	0	#DIV/0!	#DIV/0!	#DIV/0!
[TG(54:11),TG(53:4)]_C16:0	0	0	0	0	0	#DIV/0!	#DIV/0!	#DIV/0!
PC(28:2)	0	0	0	0	0	#DIV/0!	#DIV/0!	#DIV/0!
[TG(56:8),TG(55:1)]_C16:0	0	0	0	0	0	#DIV/0!	#DIV/0!	#DIV/0!
PC(42:11),PC(41:4),PC(O-42:4	0	0	0	0	0	#DIV/0!	#DIV/0!	#DIV/0!
PC(40:9),PC(39:2),PC(O-40:2)	0	0	0	0	0	#DIV/0!	#DIV/0!	#DIV/0!
PE(O-38:9),PE(36:2),PE(O-37:2	0	0	0	0	0	#DIV/0!	#DIV/0!	#DIV/0!
[TG(48:3)]_C16:0	0	0	0	0	0	#DIV/0!	#DIV/0!	#DIV/0!
[TG(50:4)]_C16:0	0	0	0	0	0	#DIV/0!	#DIV/0!	#DIV/0!
[TG(57:9),TG(56:2)]_C18:1	0	0	0	0	0	#DIV/0!	#DIV/0!	#DIV/0!
[TG(50:9),TG(49:2)]_C16:0	0	0	0	0	0	#DIV/0!	#DIV/0!	#DIV/0!
LPI(20:0)	0	0	0	0	0	#DIV/0!	#DIV/0!	#DIV/0!
[TG(55:11),TG(54:4)]_C18:3	0	0	0	0	0	#DIV/0!	#DIV/0!	#DIV/0!
[TG(37:0)]_C18:0	0	0	0	0	0	#DIV/0!	#DIV/0!	#DIV/0!
[TG(48:3)]_C18:1	0	0	0	0	0	#DIV/0!	#DIV/0!	#DIV/0!
[TG(53:10),TG(52:3)]_C18:3	0	0	0	0	0	#DIV/0!	#DIV/0!	#DIV/0!
[TG(52:9),TG(51:2)]_C18:2	0	0	0	0	0	#DIV/0!	#DIV/0!	#DIV/0!
[TG(55:9),TG(54:2)]_C20:0	0	0	0	0	0	#DIV/0!	#DIV/0!	#DIV/0!
[TG(57:9),TG(56:2)]_C20:0	0	0	0	0	0	#DIV/0!	#DIV/0!	#DIV/0!
[TG(57:10),TG(56:3)]_C20:0	0	0	0	0	0	#DIV/0!	#DIV/0!	#DIV/0!
[TG(54:6)]_C16:0	0	0	0	0	0	#DIV/0!	#DIV/0!	#DIV/0!
[TG(52:10),TG(51:3)]_C16:0	0	0	0	0	0	#DIV/0!	#DIV/0!	#DIV/0!
[TG(55:11),TG(54:4)]_C20:4	0	0	0	0	0	#DIV/0!	#DIV/0!	#DIV/0!
PC(33:0),PC(O-34:0)	0	0	0	0	0	#DIV/0!	#DIV/0!	#DIV/0!
[TG(52:5)]_C20:4	0	0	0	0	0	#DIV/0!	#DIV/0!	#DIV/0!
LPC(16:0),PC(O-16:0),LPC(O-1	0	0	0	0	0	#DIV/0!	#DIV/0!	#DIV/0!
PC(35:5),PC(O-36:5),PC(P-36:	0	0	0	0	0	#DIV/0!	#DIV/0!	#DIV/0!
SM(d18:0/24:1)	0	0	0	0	0	#DIV/0!	#DIV/0!	#DIV/0!
[TG(50:4)]_C18:1	0	0	0	0	0	#DIV/0!	#DIV/0!	#DIV/0!
SM(d16:1/25:0)	0	0	0	0	0	#DIV/0!	#DIV/0!	#DIV/0!
SM(d18:1/25:0)	0	0	0	0	0	#DIV/0!	#DIV/0!	#DIV/0!
PC(39:8),PC(O-40:8),PC(38:1)	0	0	0	0	0	#DIV/0!	#DIV/0!	#DIV/0!
PC(40:2)	0	0	0	0	0	#DIV/0!	#DIV/0!	#DIV/0!
SM(d16:1/23:0)	0	0	0	0	0	#DIV/0!	#DIV/0!	#DIV/0!
SM(d18:0/15:0)	0	0	0	0	0	#DIV/0!	#DIV/0!	#DIV/0!
SM(d18:0/24:0)	0	0	0	0	0	#DIV/0!	#DIV/0!	#DIV/0!
[TG(56:8)]_C20:4	0	0	0	0	0	#DIV/0!	#DIV/0!	#DIV/0!
PC(42:10),PC(41:3),PC(O-42:3	0	0	0	0	0	#DIV/0!	#DIV/0!	#DIV/0!
[TG(50:4)]_C18:3	0	0	0	0	0	#DIV/0!	#DIV/0!	#DIV/0!
[TG(57:12),TG(56:5)]_C18:0	0	0	0	0	0	#DIV/0!	#DIV/0!	#DIV/0!
[TG(54:9),TG(53:2)]_C16:0	0	0	0	0	0	#DIV/0!	#DIV/0!	#DIV/0!
[TG(55:8),TG(54:1)]_C20:0	0	0	0	0	0	#DIV/0!	#DIV/0!	#DIV/0!
LPC(18:1),PC(O-18:1),PC(P-18	0	0	0	0	0	#DIV/0!	#DIV/0!	#DIV/0!
[TG(54:7)]_C20:4	0	0	0	0	0	#DIV/0!	#DIV/0!	#DIV/0!
PC(35:6),PC(P-36:5)	0	0	0	0	0	#DIV/0!	#DIV/0!	#DIV/0!
PC(42:9),PC(41:2),PC(O-42:2)	0	0	0	0	0	#DIV/0!	#DIV/0!	#DIV/0!
PC(42:8),PC(41:1),PC(O-42:1)	0	0	0	0	0	#DIV/0!	#DIV/0!	#DIV/0!
PC(43:6)	0	0	0	0	0	#DIV/0!	#DIV/0!	#DIV/0!
PC(42:0)	0	0	0	0	0	#DIV/0!	#DIV/0!	#DIV/0!

[TG(56:12),TG(55:5)]_C18:1	0	0	0	0	0	#DIV/0!	#DIV/0!	#DIV/0!
LPC(22:4)	0	0	0	0	0	#DIV/0!	#DIV/0!	#DIV/0!
PC(42:2)	0	0	0	0	0	#DIV/0!	#DIV/0!	#DIV/0!
PC(42:7),PC(41:0),PC(O-42:0)	0	0	0	0	0	#DIV/0!	#DIV/0!	#DIV/0!
PC(42:5)	0	0	0	0	0	#DIV/0!	#DIV/0!	#DIV/0!
PC(43:4),PC(O-44:4)	0	0	0	0	0	#DIV/0!	#DIV/0!	#DIV/0!
[TG(56:6)]_C18:0	0	0	0	0	0	#DIV/0!	#DIV/0!	#DIV/0!
[TG(58:7)]_C22:5	0	0	0	0	0	#DIV/0!	#DIV/0!	#DIV/0!
[TG(44:0),TG(O-45:0)]_C16:0	0	0	0	0	0	#DIV/0!	#DIV/0!	#DIV/0!
[TG(54:8),TG(53:1)]_C18:0	0	0	0	0	0	#DIV/0!	#DIV/0!	#DIV/0!
[TG(54:5)]_C22:5	0	0	0	0	0	#DIV/0!	#DIV/0!	#DIV/0!
[TG(38:0)]_C14:0	0	0	0	0	0	#DIV/0!	#DIV/0!	#DIV/0!
[TG(54:8),TG(53:1)]_C16:0	0	0	0	0	0	#DIV/0!	#DIV/0!	#DIV/0!
[TG(57:12),TG(56:5)]_C22:5	0	0	0	0	0	#DIV/0!	#DIV/0!	#DIV/0!
[TG(54:9),TG(53:2)]_C18:2	0	0	0	0	0	#DIV/0!	#DIV/0!	#DIV/0!
Cer(d18:1/23:0)	0	0	0	0	0	#DIV/0!	#DIV/0!	#DIV/0!
[TG(58:9)]_C22:6	0	0	0	0	0	#DIV/0!	#DIV/0!	#DIV/0!
[TG(46:2)]_C18:2	0	0	0	0	0	#DIV/0!	#DIV/0!	#DIV/0!
[TG(46:1)]_C14:0	0	0	0	0	0	#DIV/0!	#DIV/0!	#DIV/0!
[TG(49:7)]_C18:1	0	0	0	0	0	#DIV/0!	#DIV/0!	#DIV/0!
[TG(54:5)]_C18:0	0	0	0	0	0	#DIV/0!	#DIV/0!	#DIV/0!
PC(16:0),PC(O-17:0),LPC(O-1	0	0	0	0	0	#DIV/0!	#DIV/0!	#DIV/0!
Cer(d18:1/22:0)	0	0	0	0	0	#DIV/0!	#DIV/0!	#DIV/0!
PC(42:6)	0	0	0	0	0	#DIV/0!	#DIV/0!	#DIV/0!
PC(34:6)	0	0	0	0	0	#DIV/0!	#DIV/0!	#DIV/0!
PI(38:3)	0	0	0	0	0	#DIV/0!	#DIV/0!	#DIV/0!
[TG(42:0)]_C16:0	0	0	0	0	0	#DIV/0!	#DIV/0!	#DIV/0!
[TG(50:9),TG(49:2)]_C18:1	0	0	0	0	0	#DIV/0!	#DIV/0!	#DIV/0!
[TG(49:8),TG(48:1)]_C18:0	0	0	0	0	0	#DIV/0!	#DIV/0!	#DIV/0!
[TG(52:8),TG(51:1)]_C18:0	0	0	0	0	0	#DIV/0!	#DIV/0!	#DIV/0!
[TG(55:7),TG(54:0)]_C20:0	0	0	0	0	0	#DIV/0!	#DIV/0!	#DIV/0!
PI(36:2),PI(O-37:2),PI(P-37:1)	0	0	0	0	0	#DIV/0!	#DIV/0!	#DIV/0!
[TG(53:8),TG(52:1)]_C20:0	0	0	0	0	0	#DIV/0!	#DIV/0!	#DIV/0!
[TG(50:5)]_C18:2	0	0	0	0	0	#DIV/0!	#DIV/0!	#DIV/0!
PC(44:10),PC(O-44:3)	0	0	0	0	0	#DIV/0!	#DIV/0!	#DIV/0!
[TG(46:1)]_C16:1	0	0	0	0	0	#DIV/0!	#DIV/0!	#DIV/0!
CE(22:6)Na	0	0	0	0	0	#DIV/0!	#DIV/0!	#DIV/0!
[TG(46:2)]_C16:0	0	0	0	0	0	#DIV/0!	#DIV/0!	#DIV/0!
[TG(44:1)]_C16:0	0	0	0	0	0	#DIV/0!	#DIV/0!	#DIV/0!
CAR(14:1)	0	0	0	0	0	#DIV/0!	#DIV/0!	#DIV/0!
[TG(52:7),TG(51:0)]_C16:0	0	0	0	0	0	#DIV/0!	#DIV/0!	#DIV/0!
[TG(57:10),TG(56:3)]_C18:0	0	0	0	0	0	#DIV/0!	#DIV/0!	#DIV/0!
PC(36:6)	0	0	0	0	0	#DIV/0!	#DIV/0!	#DIV/0!
PC(19:1),LPC(20:1),PC(O-20:1	0	0	0	0	0	#DIV/0!	#DIV/0!	#DIV/0!
SM(d18:1/12:0)	0	0	0	0	0	#DIV/0!	#DIV/0!	#DIV/0!
PC(32:3),PC(P-33:2)	0	0	0	0	0	#DIV/0!	#DIV/0!	#DIV/0!
[TG(58:8)]_C22:5	0	0	0	0	0	#DIV/0!	#DIV/0!	#DIV/0!
Cer(d18:1/24:1(15Z))	0	0	0	0	0	#DIV/0!	#DIV/0!	#DIV/0!
[TG(46:0)]_C18:0	0	0	0	0	0	#DIV/0!	#DIV/0!	#DIV/0!
[TG(56:9),TG(55:2)]_C18:1	0	0	0	0	0	#DIV/0!	#DIV/0!	#DIV/0!
[TG(52:7),TG(51:0)]_C18:0	0	0	0	0	0	#DIV/0!	#DIV/0!	#DIV/0!
PC(29:2),PC(P-30:1)	0	0	0	0	0	#DIV/0!	#DIV/0!	#DIV/0!
[TG(57:9),TG(56:2)]_C18:0	0	0	0	0	0	#DIV/0!	#DIV/0!	#DIV/0!
[TG(49:8)]_C18:2	0	0	0	0	0	#DIV/0!	#DIV/0!	#DIV/0!
[TG(52:6)]_C18:2	0	0	0	0	0	#DIV/0!	#DIV/0!	#DIV/0!
[TG(57:11),TG(56:4)]_C20:0	0	0	0	0	0	#DIV/0!	#DIV/0!	#DIV/0!
[TG(53:7),TG(52:0)]_C20:0	0	0	0	0	0	#DIV/0!	#DIV/0!	#DIV/0!
[TG(57:8),TG(56:1)]_C20:0	0	0	0	0	0	#DIV/0!	#DIV/0!	#DIV/0!
PE(36:3),PE(P-37:2)	0	0	0	0	0	#DIV/0!	#DIV/0!	#DIV/0!
1-O-tricosanoyl-Cer(d18:1/16:	0	0	0	0	0	#DIV/0!	#DIV/0!	#DIV/0!
SM(d17:0/27:0)	0	0	0	0	0	#DIV/0!	#DIV/0!	#DIV/0!
[TG(58:7)]_C18:1	0	0	0	0	0	#DIV/0!	#DIV/0!	#DIV/0!
[TG(38:1)]_C18:1	0	0	0	0	0	#DIV/0!	#DIV/0!	#DIV/0!
[TG(53:8),TG(52:1)]_C16:1	0	0	0	0	0	#DIV/0!	#DIV/0!	#DIV/0!
[TG(56:12),TG(55:5)]_C18:2	0	0	0	0	0	#DIV/0!	#DIV/0!	#DIV/0!
[TG(44:1)]_C18:1	0	0	0	0	0	#DIV/0!	#DIV/0!	#DIV/0!
[TG(55:7)]_C18:1	0	0	0	0	0	#DIV/0!	#DIV/0!	#DIV/0!
[TG(54:7),TG(53:0)]_C18:0	0	0	0	0	0	#DIV/0!	#DIV/0!	#DIV/0!
[TG(51:6)]_C16:0	0	0	0	0	0	#DIV/0!	#DIV/0!	#DIV/0!
[TG(54:5)]_C16:1	0	0	0	0	0	#DIV/0!	#DIV/0!	#DIV/0!
[TG(48:3)]_C14:0	0	0	0	0	0	#DIV/0!	#DIV/0!	#DIV/0!

[TG(49:7)]_C16:1	0	0	0	0	0	#DIV/0!	#DIV/0!	#DIV/0!
PC(44:0)	0	0	0	0	0	#DIV/0!	#DIV/0!	#DIV/0!
PE(40:6),PE(dO-40:0)	0	0	0	0	0	#DIV/0!	#DIV/0!	#DIV/0!
[TG(52:9),TG(51:2)]_C16:1	0	0	0	0	0	#DIV/0!	#DIV/0!	#DIV/0!
PE(36:4),PE(O-37:4)	0	0	0	0	0	#DIV/0!	#DIV/0!	#DIV/0!
[TG(52:6)]_C16:0	0	0	0	0	0	#DIV/0!	#DIV/0!	#DIV/0!
[TG(59:13),TG(58:6)]_C18:1	0	0	0	0	0	#DIV/0!	#DIV/0!	#DIV/0!
[TG(40:0)]_C16:0	0	0	0	0	0	#DIV/0!	#DIV/0!	#DIV/0!
[TG(38:0)]_C18:0	0	0	0	0	0	#DIV/0!	#DIV/0!	#DIV/0!
[TG(52:6)]_C16:1	0	0	0	0	0	#DIV/0!	#DIV/0!	#DIV/0!
PC(42:1)	0	0	0	0	0	#DIV/0!	#DIV/0!	#DIV/0!
[TG(58:8),TG(57:1)]_C18:1	0	0	0	0	0	#DIV/0!	#DIV/0!	#DIV/0!
CAR(14:2)	0	0	0	0	0	#DIV/0!	#DIV/0!	#DIV/0!
[TG(50:7),TG(49:0)]_C18:0	0	0	0	0	0	#DIV/0!	#DIV/0!	#DIV/0!
[TG(44:2)]_C16:0	0	0	0	0	0	#DIV/0!	#DIV/0!	#DIV/0!
SM(d18:2/15:0)	0	0	0	0	0	#DIV/0!	#DIV/0!	#DIV/0!
[TG(48:4)]_C18:2	0	0	0	0	0	#DIV/0!	#DIV/0!	#DIV/0!
[TG(49:3)]_C18:2	0	0	0	0	0	#DIV/0!	#DIV/0!	#DIV/0!
PC(24:0)	0	0	0	0	0	#DIV/0!	#DIV/0!	#DIV/0!
[TG(54:12),TG(53:5)]_C18:2	0	0	0	0	0	#DIV/0!	#DIV/0!	#DIV/0!
[TG(51:6)]_C18:0	0	0	0	0	0	#DIV/0!	#DIV/0!	#DIV/0!
[TG(44:0),TG(O-45:0)]_C18:0	0	0	0	0	0	#DIV/0!	#DIV/0!	#DIV/0!
[TG(56:8),TG(55:1)]_C18:1	0	0	0	0	0	#DIV/0!	#DIV/0!	#DIV/0!
[TG(46:3)]_C18:1	0	0	0	0	0	#DIV/0!	#DIV/0!	#DIV/0!
[TG(55:11),TG(54:4)]_C16:1	0	0	0	0	0	#DIV/0!	#DIV/0!	#DIV/0!
[TG(48:7),TG(47:0)]_C16:0	0	0	0	0	0	#DIV/0!	#DIV/0!	#DIV/0!
[TG(44:0),TG(O-45:0)]_C14:0	0	0	0	0	0	#DIV/0!	#DIV/0!	#DIV/0!
LPC(20:3)	0	0	0	0	0	#DIV/0!	#DIV/0!	#DIV/0!
[TG(55:10),TG(54:3)]_C20:0	0	0	0	0	0	#DIV/0!	#DIV/0!	#DIV/0!
Cer(d18:1/16:0)	0	0	0	0	0	#DIV/0!	#DIV/0!	#DIV/0!
LPE(22:4)	0	0	0	0	0	#DIV/0!	#DIV/0!	#DIV/0!
CE(20:1)H	0	0	0	0	0	#DIV/0!	#DIV/0!	#DIV/0!
DG(30:2)_C16:0	0	0	0	0	0	#DIV/0!	#DIV/0!	#DIV/0!
DG(30:3)_C16:1	0	0	0	0	0	#DIV/0!	#DIV/0!	#DIV/0!
DG(36:8),DG(35:1)_C16:1	0	0	0	0	0	#DIV/0!	#DIV/0!	#DIV/0!
[TG(42:0)]_C14:0	0	0	0	0	0	#DIV/0!	#DIV/0!	#DIV/0!
CAR(20:0)	0	0	0	0	0	#DIV/0!	#DIV/0!	#DIV/0!
DG(36:7),DG(35:0)_C16:0	0	0	0	0	0	#DIV/0!	#DIV/0!	#DIV/0!
DG(36:6)_C16:0	0	0	0	0	0	#DIV/0!	#DIV/0!	#DIV/0!
DG(41:5)_C16:0	0	0	0	0	0	#DIV/0!	#DIV/0!	#DIV/0!
DG(42:11),DG(41:4)_C16:0	0	0	0	0	0	#DIV/0!	#DIV/0!	#DIV/0!
DG(30:2)_C16:1	0	0	0	0	0	#DIV/0!	#DIV/0!	#DIV/0!
DG(36:7)_C16:1	0	0	0	0	0	#DIV/0!	#DIV/0!	#DIV/0!
CE(15:1) NH4	0	0	0	0	0	#DIV/0!	#DIV/0!	#DIV/0!
DG(41:6)_C16:1	0	0	0	0	0	#DIV/0!	#DIV/0!	#DIV/0!
DG(36:8),DG(35:1)_C18:1	0	0	0	0	0	#DIV/0!	#DIV/0!	#DIV/0!
Cer(d18:0/17:0)	0	0	0	0	0	#DIV/0!	#DIV/0!	#DIV/0!
DG(36:5)_C16:0	0	0	0	0	0	#DIV/0!	#DIV/0!	#DIV/0!
DG(30:1)_C16:0	0	0	0	0	0	#DIV/0!	#DIV/0!	#DIV/0!
DG(36:6)_C16:1	0	0	0	0	0	#DIV/0!	#DIV/0!	#DIV/0!
DG(32:5)_C18:1	0	0	0	0	0	#DIV/0!	#DIV/0!	#DIV/0!
FA(24:4)	0	0	0	0	0	#DIV/0!	#DIV/0!	#DIV/0!
[TG(57:11),TG(56:4)]_C16:0	0	0	0	0	0	#DIV/0!	#DIV/0!	#DIV/0!
PC(30:3)	0	0	0	0	0	#DIV/0!	#DIV/0!	#DIV/0!
FA(30:0)	0	0	0	0	0	#DIV/0!	#DIV/0!	#DIV/0!
FA(18:3)	0	0	0	0	0	#DIV/0!	#DIV/0!	#DIV/0!
DG(34:1)_C16:1	0	0	0	0	0	#DIV/0!	#DIV/0!	#DIV/0!
LPI(19:0),LPI(O-20:0)	0	0	0	0	0	#DIV/0!	#DIV/0!	#DIV/0!
DG(41:6)_C18:1	0	0	0	0	0	#DIV/0!	#DIV/0!	#DIV/0!
CE(15:0) NH4	0	0	0	0	0	#DIV/0!	#DIV/0!	#DIV/0!
[TG(48:8),TG(47:1)]_C16:1	0	0	0	0	0	#DIV/0!	#DIV/0!	#DIV/0!
[TG(48:8),TG(47:1)]_C16:0	0	0	0	0	0	#DIV/0!	#DIV/0!	#DIV/0!
PC(31:3),PC(O-32:3)	0	0	0	0	0	#DIV/0!	#DIV/0!	#DIV/0!
[TG(58:8)]_C18:2	0	0	0	0	0	#DIV/0!	#DIV/0!	#DIV/0!
[TG(45:3)]_C16:0	0	0	0	0	0	#DIV/0!	#DIV/0!	#DIV/0!
CE(20:1)Na	0	0	0	0	0	#DIV/0!	#DIV/0!	#DIV/0!
CE(16:0)H	0	0	0	0	0	#DIV/0!	#DIV/0!	#DIV/0!
FA(10:3)	0	0	0	0	0	#DIV/0!	#DIV/0!	#DIV/0!
[TG(50:9),TG(49:2)]_C16:1	0	0	0	0	0	#DIV/0!	#DIV/0!	#DIV/0!
DG(41:5)_C16:1	0	0	0	0	0	#DIV/0!	#DIV/0!	#DIV/0!
CE(22:4)H	0	0	0	0	0	#DIV/0!	#DIV/0!	#DIV/0!

LPG(18:0); LPG(18:0)	0	0	0	0	0	#DIV/0!	#DIV/0!	#DIV/0!
[TG(46:2)]_C16:1	0	0	0	0	0	#DIV/0!	#DIV/0!	#DIV/0!
[TG(48:8),TG(47:1)]_C18:1	0	0	0	0	0	#DIV/0!	#DIV/0!	#DIV/0!
CE(17:0)Na	0	0	0	0	0	#DIV/0!	#DIV/0!	#DIV/0!
PC(44:1)	0	0	0	0	0	#DIV/0!	#DIV/0!	#DIV/0!
[TG(46:3)]_C18:2	0	0	0	0	0	#DIV/0!	#DIV/0!	#DIV/0!
[TG(58:9),TG(57:2)]_C18:1	0	0	0	0	0	#DIV/0!	#DIV/0!	#DIV/0!
[TG(57:8),TG(56:1)]_C18:0	0	0	0	0	0	#DIV/0!	#DIV/0!	#DIV/0!
[TG(49:3)]_C16:0	0	0	0	0	0	#DIV/0!	#DIV/0!	#DIV/0!
PI(34:2),PI(O-35:2),PI(P-35:1)	0	0	0	0	0	#DIV/0!	#DIV/0!	#DIV/0!
[TG(52:10),TG(51:3)]_C16:1	0	0	0	0	0	#DIV/0!	#DIV/0!	#DIV/0!
CE(24:1) NH4	0	0	0	0	0	#DIV/0!	#DIV/0!	#DIV/0!
1-O-pentacosanoyl-Cer(d18:1/	0	0	0	0	0	#DIV/0!	#DIV/0!	#DIV/0!
FA(16:0)	0	0	0	0	0	#DIV/0!	#DIV/0!	#DIV/0!
[TG(50:9),TG(49:2)]_C20:0	0	0	0	0	0	#DIV/0!	#DIV/0!	#DIV/0!
[TG(47:6)]_C16:0	0	0	0	0	0	#DIV/0!	#DIV/0!	#DIV/0!
[TG(53:9),TG(52:2)]_C20:0	0	0	0	0	0	#DIV/0!	#DIV/0!	#DIV/0!
[TG(50:5)]_C18:3	0	0	0	0	0	#DIV/0!	#DIV/0!	#DIV/0!
CE(24:1)Na	0	0	0	0	0	#DIV/0!	#DIV/0!	#DIV/0!
Cer(d14:2(4E,6E)/16:0)	0	0	0	0	0	#DIV/0!	#DIV/0!	#DIV/0!
FA(22:1)	0	0	0	0	0	#DIV/0!	#DIV/0!	#DIV/0!
[TG(51:7),TG(50:0)]_C14:0	0	0	0	0	0	#DIV/0!	#DIV/0!	#DIV/0!
[TG(42:1)]_C18:1	0	0	0	0	0	#DIV/0!	#DIV/0!	#DIV/0!
PI(36:1),PI(O-37:1),PI(P-37:0)	0	0	0	0	0	#DIV/0!	#DIV/0!	#DIV/0!
CAR(20:1)	0	0	0	0	0	#DIV/0!	#DIV/0!	#DIV/0!
[TG(50:8),TG(49:1)]_C16:1	0	0	0	0	0	#DIV/0!	#DIV/0!	#DIV/0!
PG(25:0); PG(25:0)	0	0	0	0	0	#DIV/0!	#DIV/0!	#DIV/0!
[TG(45:0)]_C16:0	0	0	0	0	0	#DIV/0!	#DIV/0!	#DIV/0!
[TG(57:8),TG(56:1)]_C18:1	0	0	0	0	0	#DIV/0!	#DIV/0!	#DIV/0!
PG(O-35:1),PG(P-35:0); PG(O-	0	0	0	0	0	#DIV/0!	#DIV/0!	#DIV/0!
[TG(54:7)]_C18:1	0	0	0	0	0	#DIV/0!	#DIV/0!	#DIV/0!
PE(37:5),PE(O-38:5),PE(P-38:4	0	0	0	0	0	#DIV/0!	#DIV/0!	#DIV/0!
PC(44:3)	0	0	0	0	0	#DIV/0!	#DIV/0!	#DIV/0!
PE(38:3)	0	0	0	0	0	#DIV/0!	#DIV/0!	#DIV/0!
FA(22:0)	0	0	0	0	0	#DIV/0!	#DIV/0!	#DIV/0!
FA(37:0)	0	0	0	0	0	#DIV/0!	#DIV/0!	#DIV/0!
CAR(18:3)	0	0	0	0	0	#DIV/0!	#DIV/0!	#DIV/0!
FA(34:6)	0	0	0	0	0	#DIV/0!	#DIV/0!	#DIV/0!
[TG(42:0)]_C18:0	0	0	0	0	0	#DIV/0!	#DIV/0!	#DIV/0!
FA(19:0)	0	0	0	0	0	#DIV/0!	#DIV/0!	#DIV/0!
FA(26:6)	0	0	0	0	0	#DIV/0!	#DIV/0!	#DIV/0!
FA(23:0)	0	0	0	0	0	#DIV/0!	#DIV/0!	#DIV/0!
[TG(55:7),TG(54:0)]_C16:0	0	0	0	0	0	#DIV/0!	#DIV/0!	#DIV/0!
CE(20:0)K	0	0	0	0	0	#DIV/0!	#DIV/0!	#DIV/0!
PC(44:2)	0	0	0	0	0	#DIV/0!	#DIV/0!	#DIV/0!
[TG(59:10),TG(58:3)]_C18:2	0	0	0	0	0	#DIV/0!	#DIV/0!	#DIV/0!
[TG(44:2)]_C18:2	0	0	0	0	0	#DIV/0!	#DIV/0!	#DIV/0!
[TG(50:8),TG(49:1)]_C14:0	0	0	0	0	0	#DIV/0!	#DIV/0!	#DIV/0!
[TG(52:5)]_C22:5	0	0	0	0	0	#DIV/0!	#DIV/0!	#DIV/0!
PI(38:5)	0	0	0	0	0	#DIV/0!	#DIV/0!	#DIV/0!
[TG(52:9),TG(51:2)]_C18:0	0	0	0	0	0	#DIV/0!	#DIV/0!	#DIV/0!
[TG(52:7)]_C18:1	0	0	0	0	0	#DIV/0!	#DIV/0!	#DIV/0!
LPC(14:0),PC(O-14:0),LPC(O-1	0	0	0	0	0	#DIV/0!	#DIV/0!	#DIV/0!
[TG(45:2)]_C16:0	0	0	0	0	0	#DIV/0!	#DIV/0!	#DIV/0!
FA(21:1)	0	0	0	0	0	#DIV/0!	#DIV/0!	#DIV/0!
CAR(18:2)	0	0	0	0	0	#DIV/0!	#DIV/0!	#DIV/0!
[TG(45:1)]_C16:1	0	0	0	0	0	#DIV/0!	#DIV/0!	#DIV/0!
FA(26:2)	0	0	0	0	0	#DIV/0!	#DIV/0!	#DIV/0!
Cer(d18:0/21:0)	0	0	0	0	0	#DIV/0!	#DIV/0!	#DIV/0!
DG(41:6)_C16:0	0	0	0	0	0	#DIV/0!	#DIV/0!	#DIV/0!
LPS(P-16:0)	0	0	0	0	0	#DIV/0!	#DIV/0!	#DIV/0!
[TG(45:4)]_C20:0	0	0	0	0	0	#DIV/0!	#DIV/0!	#DIV/0!
DG(30:1)_C16:1	0	0	0	0	0	#DIV/0!	#DIV/0!	#DIV/0!
DG(41:5)_C18:0	0	0	0	0	0	#DIV/0!	#DIV/0!	#DIV/0!
DG(35:3)_C18:0	0	0	0	0	0	#DIV/0!	#DIV/0!	#DIV/0!
FA(34:0)	0	0	0	0	0	#DIV/0!	#DIV/0!	#DIV/0!
[TG(47:2)]_C16:1	0	0	0	0	0	#DIV/0!	#DIV/0!	#DIV/0!
[TG(44:1)]_C14:0	0	0	0	0	0	#DIV/0!	#DIV/0!	#DIV/0!
[TG(51:7)]_C16:1	0	0	0	0	0	#DIV/0!	#DIV/0!	#DIV/0!
[TG(38:0)]_C20:0	0	0	0	0	0	#DIV/0!	#DIV/0!	#DIV/0!
[TG(57:9),TG(56:2)]_C18:2	0	0	0	0	0	#DIV/0!	#DIV/0!	#DIV/0!

[TG(57:8),TG(56:1)]_C16:0	0	0	0	0	0	#DIV/0!	#DIV/0!	#DIV/0!
[TG(57:9),TG(56:2)]_C16:0	0	0	0	0	0	#DIV/0!	#DIV/0!	#DIV/0!
PC(19:0),LPC(20:0),PC(O-20:(	0	0	0	0	0	#DIV/0!	#DIV/0!	#DIV/0!
[TG(46:1)]_C18:0	0	0	0	0	0	#DIV/0!	#DIV/0!	#DIV/0!
[TG(48:7),TG(47:0)]_C14:0	0	0	0	0	0	#DIV/0!	#DIV/0!	#DIV/0!
[TG(56:8)]_C18:3	0	0	0	0	0	#DIV/0!	#DIV/0!	#DIV/0!
[TG(48:4)]_C18:1	0	0	0	0	0	#DIV/0!	#DIV/0!	#DIV/0!
[TG(48:6)]_C16:0	0	0	0	0	0	#DIV/0!	#DIV/0!	#DIV/0!
CE(14:1) NH4	0	0	0	0	0	#DIV/0!	#DIV/0!	#DIV/0!
[TG(48:4)]_C18:3	0	0	0	0	0	#DIV/0!	#DIV/0!	#DIV/0!
[TG(39:0)]_C16:0	0	0	0	0	0	#DIV/0!	#DIV/0!	#DIV/0!
[TG(56:13),TG(55:6)]_C18:2	0	0	0	0	0	#DIV/0!	#DIV/0!	#DIV/0!
FA(38:6)	0	0	0	0	0	#DIV/0!	#DIV/0!	#DIV/0!
[TG(48:6)]_C18:0	0	0	0	0	0	#DIV/0!	#DIV/0!	#DIV/0!
PI(38:2),PI(P-39:1)	0	0	0	0	0	#DIV/0!	#DIV/0!	#DIV/0!
DG(36:8),DG(35:1)_C18:0	0	0	0	0	0	#DIV/0!	#DIV/0!	#DIV/0!
PC(44:7),PC(43:0)	0	0	0	0	0	#DIV/0!	#DIV/0!	#DIV/0!
[TG(37:0)]_C14:0	0	0	0	0	0	#DIV/0!	#DIV/0!	#DIV/0!
[TG(55:10),TG(54:3)]_C16:1	0	0	0	0	0	#DIV/0!	#DIV/0!	#DIV/0!
FA(10:5)	0	0	0	0	0	#DIV/0!	#DIV/0!	#DIV/0!
FA(35:0)	0	0	0	0	0	#DIV/0!	#DIV/0!	#DIV/0!
[TG(53:10),TG(52:3)]_C20:0	0	0	0	0	0	#DIV/0!	#DIV/0!	#DIV/0!
PS(25:0)	0	0	0	0	0	#DIV/0!	#DIV/0!	#DIV/0!
FA(28:0)	0	0	0	0	0	#DIV/0!	#DIV/0!	#DIV/0!
FA(32:0)	0	0	0	0	0	#DIV/0!	#DIV/0!	#DIV/0!
[TG(58:9)]_C20:4	0	0	0	0	0	#DIV/0!	#DIV/0!	#DIV/0!
[TG(54:6)]_C16:1	0	0	0	0	0	#DIV/0!	#DIV/0!	#DIV/0!
PC(44:4)	0	0	0	0	0	#DIV/0!	#DIV/0!	#DIV/0!
FA(18:2)	0	0	0	0	0	#DIV/0!	#DIV/0!	#DIV/0!
FA(28:6)	0	0	0	0	0	#DIV/0!	#DIV/0!	#DIV/0!
FA(21:2)	0	0	0	0	0	#DIV/0!	#DIV/0!	#DIV/0!
FA(30:2)	0	0	0	0	0	#DIV/0!	#DIV/0!	#DIV/0!
CAR(26:0)	0	0	0	0	0	#DIV/0!	#DIV/0!	#DIV/0!
DG(37:7)_C18:1	0	0	0	0	0	#DIV/0!	#DIV/0!	#DIV/0!
DG(30:2)_C18:1	0	0	0	0	0	#DIV/0!	#DIV/0!	#DIV/0!
FA(24:0)	0	0	0	0	0	#DIV/0!	#DIV/0!	#DIV/0!
FA(24:2)	0	0	0	0	0	#DIV/0!	#DIV/0!	#DIV/0!
FA(20:6)	0	0	0	0	0	#DIV/0!	#DIV/0!	#DIV/0!
FA(24:1)	0	0	0	0	0	#DIV/0!	#DIV/0!	#DIV/0!
PG(24:0); PG(24:0)	0	0	0	0	0	#DIV/0!	#DIV/0!	#DIV/0!
[TG(40:0)]_C18:0	0	0	0	0	0	#DIV/0!	#DIV/0!	#DIV/0!
LPC(22:6)	0	0	0	0	0	#DIV/0!	#DIV/0!	#DIV/0!
PC(26:1)	0	0	0	0	0	#DIV/0!	#DIV/0!	#DIV/0!
CE(24:1)K	0	0	0	0	0	#DIV/0!	#DIV/0!	#DIV/0!
[TG(44:5)]_C20:0	0	0	0	0	0	#DIV/0!	#DIV/0!	#DIV/0!
[TG(59:10),TG(58:3)]_C18:1	0	0	0	0	0	#DIV/0!	#DIV/0!	#DIV/0!
[TG(46:2)]_C14:0	0	0	0	0	0	#DIV/0!	#DIV/0!	#DIV/0!
[TG(50:4)]_C20:4	0	0	0	0	0	#DIV/0!	#DIV/0!	#DIV/0!
PG(32:0),PG(O-33:0); PG(32:0	0	0	0	0	0	#DIV/0!	#DIV/0!	#DIV/0!
[TG(44:2)]_C18:1	0	0	0	0	0	#DIV/0!	#DIV/0!	#DIV/0!
[TG(59:9),TG(58:2)]_C18:2	0	0	0	0	0	#DIV/0!	#DIV/0!	#DIV/0!
PI(36:4)	0	0	0	0	0	#DIV/0!	#DIV/0!	#DIV/0!
[TG(52:4)]_C18:0	0	0	0	0	0	#DIV/0!	#DIV/0!	#DIV/0!
[TG(53:9),TG(52:2)]_C14:0	0	0	0	0	0	#DIV/0!	#DIV/0!	#DIV/0!
PC(44:8),PC(43:1)	0	0	0	0	0	#DIV/0!	#DIV/0!	#DIV/0!
[TG(38:1)]_C16:0	0	0	0	0	0	#DIV/0!	#DIV/0!	#DIV/0!
DG(40:7),DG(39:0)_C18:0	0	0	0	0	0	#DIV/0!	#DIV/0!	#DIV/0!
LPG(15:0),LPG(O-16:0); LPG(1	0	0	0	0	0	#DIV/0!	#DIV/0!	#DIV/0!
[TG(46:6)]_C16:0	0	0	0	0	0	#DIV/0!	#DIV/0!	#DIV/0!
FA(22:5)	0	0	0	0	0	#DIV/0!	#DIV/0!	#DIV/0!
[TG(51:8)]_C22:5	0	0	0	0	0	#DIV/0!	#DIV/0!	#DIV/0!
FA(32:5)	0	0	0	0	0	#DIV/0!	#DIV/0!	#DIV/0!
FA(30:4)	0	0	0	0	0	#DIV/0!	#DIV/0!	#DIV/0!
FA(10:2)	0	0	0	0	0	#DIV/0!	#DIV/0!	#DIV/0!
FA(23:1)	0	0	0	0	0	#DIV/0!	#DIV/0!	#DIV/0!
FA(20:2)	0	0	0	0	0	#DIV/0!	#DIV/0!	#DIV/0!
CAR(22:5)	0	0	0	0	0	#DIV/0!	#DIV/0!	#DIV/0!
[TG(62:14),TG(61:7),TG(60:0)]_	0	0	0	0	0	#DIV/0!	#DIV/0!	#DIV/0!
CAR(20:2)	0	0	0	0	0	#DIV/0!	#DIV/0!	#DIV/0!
[TG(37:0)]_C16:0	0	0	0	0	0	#DIV/0!	#DIV/0!	#DIV/0!
DG(32:1)_C18:1	0	0	0	0	0	#DIV/0!	#DIV/0!	#DIV/0!

DG(33:1),DG(O-34:1)_C16:1	0	0	0	0	0	#DIV/0!	#DIV/0!	#DIV/0!
DG(33:1),DG(O-34:1)_C18:1	0	0	0	0	0	#DIV/0!	#DIV/0!	#DIV/0!
DG(36:7)_C18:1	0	0	0	0	0	#DIV/0!	#DIV/0!	#DIV/0!
FA(19:1)	0	0	0	0	0	#DIV/0!	#DIV/0!	#DIV/0!
LPI(16:0)	0	0	0	0	0	#DIV/0!	#DIV/0!	#DIV/0!
[TG(41:0)]_C16:0	0	0	0	0	0	#DIV/0!	#DIV/0!	#DIV/0!
LPE(18:1)	0	0	0	0	0	#DIV/0!	#DIV/0!	#DIV/0!
FA(25:0)	0	0	0	0	0	#DIV/0!	#DIV/0!	#DIV/0!
FA(6:2)	0	0	0	0	0	#DIV/0!	#DIV/0!	#DIV/0!
FA(33:0)	0	0	0	0	0	#DIV/0!	#DIV/0!	#DIV/0!
DG(30:1)_C18:1	0	0	0	0	0	#DIV/0!	#DIV/0!	#DIV/0!
CAR(18:0)	0	0	0	0	0	#DIV/0!	#DIV/0!	#DIV/0!
LPG(16:0); LPG(16:0)	0	0	0	0	0	#DIV/0!	#DIV/0!	#DIV/0!
FA(21:5)	0	0	0	0	0	#DIV/0!	#DIV/0!	#DIV/0!
FA(22:6)	0	0	0	0	0	#DIV/0!	#DIV/0!	#DIV/0!
FA(26:4)	0	0	0	0	0	#DIV/0!	#DIV/0!	#DIV/0!
FA(19:6)	0	0	0	0	0	#DIV/0!	#DIV/0!	#DIV/0!
DG(24:0)_C18:0	0	0	0	0	0	#DIV/0!	#DIV/0!	#DIV/0!
[TG(57:8)]_C18:2	0	0	0	0	0	#DIV/0!	#DIV/0!	#DIV/0!
FA(20:1)	0	0	0	0	0	#DIV/0!	#DIV/0!	#DIV/0!
FA(18:1)	0	0	0	0	0	#DIV/0!	#DIV/0!	#DIV/0!
CAR(22:0)	0	0	0	0	0	#DIV/0!	#DIV/0!	#DIV/0!
CAR(5:1)	0	0	0	0	0	#DIV/0!	#DIV/0!	#DIV/0!
FA(22:2)	0	0	0	0	0	#DIV/0!	#DIV/0!	#DIV/0!
CAR(10:3)	0	0	0	0	0	#DIV/0!	#DIV/0!	#DIV/0!
FA(36:6)	0	0	0	0	0	#DIV/0!	#DIV/0!	#DIV/0!
[TG(43:1)]_C16:1	0	0	0	0	0	#DIV/0!	#DIV/0!	#DIV/0!
FA(16:6)	0	0	0	0	0	#DIV/0!	#DIV/0!	#DIV/0!
[TG(46:3)]_C16:1	0	0	0	0	0	#DIV/0!	#DIV/0!	#DIV/0!
[TG(58:14),TG(57:7),TG(56:0)]_	0	0	0	0	0	#DIV/0!	#DIV/0!	#DIV/0!
FA(27:3)	0	0	0	0	0	#DIV/0!	#DIV/0!	#DIV/0!
FA(36:4)	0	0	0	0	0	#DIV/0!	#DIV/0!	#DIV/0!
FA(19:5)	0	0	0	0	0	#DIV/0!	#DIV/0!	#DIV/0!
FA(24:5)	0	0	0	0	0	#DIV/0!	#DIV/0!	#DIV/0!
CAR(14:0)	0	0	0	0	0	#DIV/0!	#DIV/0!	#DIV/0!
[TG(45:2)]_C16:1	0	0	0	0	0	#DIV/0!	#DIV/0!	#DIV/0!
DG(33:3)_C16:0	0	0	0	0	0	#DIV/0!	#DIV/0!	#DIV/0!
FA(9:0)	0	0	0	0	0	#DIV/0!	#DIV/0!	#DIV/0!
FA(14:6)	0	0	0	0	0	#DIV/0!	#DIV/0!	#DIV/0!
FA(18:6)	0	0	0	0	0	#DIV/0!	#DIV/0!	#DIV/0!
FA(18:5)	0	0	0	0	0	#DIV/0!	#DIV/0!	#DIV/0!
FA(20:3)	0	0	0	0	0	#DIV/0!	#DIV/0!	#DIV/0!
PE(32:1),PE(O-33:1),PE(P-33:(	0	0	0	0	0	#DIV/0!	#DIV/0!	#DIV/0!
DG(38:7),DG(37:0)_C16:0	0	0	0	0	0	#DIV/0!	#DIV/0!	#DIV/0!
DG(32:1)_C16:1	0	0	0	0	0	#DIV/0!	#DIV/0!	#DIV/0!
	0	0	0	0	0	#DIV/0!	#DIV/0!	#DIV/0!
CE(24:1)H	0	0	0	0	0	#DIV/0!	#DIV/0!	#DIV/0!
CE(22:0)K	0	0	0	0	0	#DIV/0!	#DIV/0!	#DIV/0!
LPE(22:6)	0	0	0	0	0	#DIV/0!	#DIV/0!	#DIV/0!
PC(44:5)	0	0	0	0	0	#DIV/0!	#DIV/0!	#DIV/0!
CE(12:0) NH4	0	0	0	0	0	#DIV/0!	#DIV/0!	#DIV/0!
DG(40:6),DG(dO-40:0)_C16:0	0	0	0	0	0	#DIV/0!	#DIV/0!	#DIV/0!
CE(17:0) NH4	0	0	0	0	0	#DIV/0!	#DIV/0!	#DIV/0!
PE(37:6),PE(O-38:6),PE(P-38:5	0	0	0	0	0	#DIV/0!	#DIV/0!	#DIV/0!
DG(44:9),DG(43:2)_C18:1	0	0	0	0	0	#DIV/0!	#DIV/0!	#DIV/0!
[TG(58:8)]_C20:4	0	0	0	0	0	#DIV/0!	#DIV/0!	#DIV/0!
DG(44:7),DG(43:0)_C16:0	0	0	0	0	0	#DIV/0!	#DIV/0!	#DIV/0!
CE(18:2)H	0	0	0	0	0	#DIV/0!	#DIV/0!	#DIV/0!
DG(44:8),DG(43:1)_C18:1	0	0	0	0	0	#DIV/0!	#DIV/0!	#DIV/0!
CE(22:6)K	0	0	0	0	0	#DIV/0!	#DIV/0!	#DIV/0!
DG(34:3)_C16:0	0	0	0	0	0	#DIV/0!	#DIV/0!	#DIV/0!
PE(40:5)	0	0	0	0	0	#DIV/0!	#DIV/0!	#DIV/0!
DG(40:7),DG(39:0)_C16:0	0	0	0	0	0	#DIV/0!	#DIV/0!	#DIV/0!
PE(35:4),PE(O-36:4),PE(P-36:3	0	0	0	0	0	#DIV/0!	#DIV/0!	#DIV/0!
DG(33:2)_C18:1	0	0	0	0	0	#DIV/0!	#DIV/0!	#DIV/0!
CAR(17:0)	0	0	0	0	0	#DIV/0!	#DIV/0!	#DIV/0!
[TG(44:3)]_C20:0	0	0	0	0	0	#DIV/0!	#DIV/0!	#DIV/0!
FA(14:5)	0	0	0	0	0	#DIV/0!	#DIV/0!	#DIV/0!
LPE(18:2),LPE(P-19:1)	0	0	0	0	0	#DIV/0!	#DIV/0!	#DIV/0!
[TG(40:1)]_C18:1	0	0	0	0	0	#DIV/0!	#DIV/0!	#DIV/0!
PC(43:2)	0	0	0	0	0	#DIV/0!	#DIV/0!	#DIV/0!

DG(28:2)_C18:1	0	0	0	0	0	#DIV/0!	#DIV/0!	#DIV/0!
DG(28:1)_C18:1	0	0	0	0	0	#DIV/0!	#DIV/0!	#DIV/0!
DG(31:1)_C18:1	0	0	0	0	0	#DIV/0!	#DIV/0!	#DIV/0!
DG(36:4),DG(O-37:4)_C18:1	0	0	0	0	0	#DIV/0!	#DIV/0!	#DIV/0!
CAR(22:2)	0	0	0	0	0	#DIV/0!	#DIV/0!	#DIV/0!
[TG(43:1)]_C18:1	0	0	0	0	0	#DIV/0!	#DIV/0!	#DIV/0!
[TG(38:1)]_C14:0	0	0	0	0	0	#DIV/0!	#DIV/0!	#DIV/0!
FA(40:6)	0	0	0	0	0	#DIV/0!	#DIV/0!	#DIV/0!
DG(42:6)_C16:0	0	0	0	0	0	#DIV/0!	#DIV/0!	#DIV/0!
FA(3:0)	0	0	0	0	0	#DIV/0!	#DIV/0!	#DIV/0!
[TG(43:1)]_C16:0	0	0	0	0	0	#DIV/0!	#DIV/0!	#DIV/0!
FA(27:0)	0	0	0	0	0	#DIV/0!	#DIV/0!	#DIV/0!
DG(44:7),DG(43:0)_C18:0	0	0	0	0	0	#DIV/0!	#DIV/0!	#DIV/0!
DG(42:7),DG(41:0)_C16:0	0	0	0	0	0	#DIV/0!	#DIV/0!	#DIV/0!
FA(28:1)	0	0	0	0	0	#DIV/0!	#DIV/0!	#DIV/0!
FA(26:0)	0	0	0	0	0	#DIV/0!	#DIV/0!	#DIV/0!
DG(38:6),DG(dO-40:6)_C16:0	0	0	0	0	0	#DIV/0!	#DIV/0!	#DIV/0!
FA(34:1)	0	0	0	0	0	#DIV/0!	#DIV/0!	#DIV/0!
[TG(55:7),TG(54:0)]_C14:0	0	0	0	0	0	#DIV/0!	#DIV/0!	#DIV/0!
[TG(59:9),TG(58:2)]_C18:1	0	0	0	0	0	#DIV/0!	#DIV/0!	#DIV/0!
[TG(48:3)]_C18:3	0	0	0	0	0	#DIV/0!	#DIV/0!	#DIV/0!
[TG(44:1)]_C16:1	0	0	0	0	0	#DIV/0!	#DIV/0!	#DIV/0!
[TG(48:8),TG(47:1)]_C14:0	0	0	0	0	0	#DIV/0!	#DIV/0!	#DIV/0!
[TG(62:16),TG(61:9),TG(60:2)]_	0	0	0	0	0	#DIV/0!	#DIV/0!	#DIV/0!
[TG(60:15),TG(59:8),TG(58:1)]_	0	0	0	0	0	#DIV/0!	#DIV/0!	#DIV/0!
[TG(61:10),TG(60:3)]_C18:1	0	0	0	0	0	#DIV/0!	#DIV/0!	#DIV/0!
[TG(52:4)]_C14:0	0	0	0	0	0	#DIV/0!	#DIV/0!	#DIV/0!
[TG(47:2)]_C18:2	0	0	0	0	0	#DIV/0!	#DIV/0!	#DIV/0!
[TG(50:5)]_C20:4	0	0	0	0	0	#DIV/0!	#DIV/0!	#DIV/0!
[TG(42:1)]_C16:0	0	0	0	0	0	#DIV/0!	#DIV/0!	#DIV/0!
PC(14:0),LPC(15:0),LPC(O-16:	0	0	0	0	0	#DIV/0!	#DIV/0!	#DIV/0!
[TG(59:11),TG(58:4)]_C18:2	0	0	0	0	0	#DIV/0!	#DIV/0!	#DIV/0!
[TG(50:7)]_C18:1	0	0	0	0	0	#DIV/0!	#DIV/0!	#DIV/0!
[TG(45:0)]_C14:0	0	0	0	0	0	#DIV/0!	#DIV/0!	#DIV/0!
LPC(15:1),LPC(O-16:1),LPC(P-	0	0	0	0	0	#DIV/0!	#DIV/0!	#DIV/0!
[TG(58:10)]_C20:4	0	0	0	0	0	#DIV/0!	#DIV/0!	#DIV/0!
[TG(50:9),TG(49:2)]_C14:0	0	0	0	0	0	#DIV/0!	#DIV/0!	#DIV/0!
PI(36:3),PI(P-37:2)	0	0	0	0	0	#DIV/0!	#DIV/0!	#DIV/0!
[TG(47:6)]_C14:0	0	0	0	0	0	#DIV/0!	#DIV/0!	#DIV/0!
[TG(47:2)]_C16:0	0	0	0	0	0	#DIV/0!	#DIV/0!	#DIV/0!
[TG(59:9),TG(58:2)]_C16:0	0	0	0	0	0	#DIV/0!	#DIV/0!	#DIV/0!
[TG(43:0)]_C16:0	0	0	0	0	0	#DIV/0!	#DIV/0!	#DIV/0!
[TG(46:3)]_C14:0	0	0	0	0	0	#DIV/0!	#DIV/0!	#DIV/0!
[TG(51:8),TG(50:1)]_C20:0	0	0	0	0	0	#DIV/0!	#DIV/0!	#DIV/0!
[TG(42:2)]_C18:2	0	0	0	0	0	#DIV/0!	#DIV/0!	#DIV/0!
[TG(42:1)]_C14:0	0	0	0	0	0	#DIV/0!	#DIV/0!	#DIV/0!
[TG(45:1)]_C16:0	0	0	0	0	0	#DIV/0!	#DIV/0!	#DIV/0!
[TG(44:2)]_C16:1	0	0	0	0	0	#DIV/0!	#DIV/0!	#DIV/0!
[TG(44:2)]_C14:0	0	0	0	0	0	#DIV/0!	#DIV/0!	#DIV/0!
FA(36:5)	0	0	0	0	0	#DIV/0!	#DIV/0!	#DIV/0!
PG(O-37:2),PG(P-37:1); PG(O-	0	0	0	0	0	#DIV/0!	#DIV/0!	#DIV/0!
[TG(47:2)]_C14:0	0	0	0	0	0	#DIV/0!	#DIV/0!	#DIV/0!
[TG(49:3)]_C18:1	0	0	0	0	0	#DIV/0!	#DIV/0!	#DIV/0!
[TG(45:1)]_C18:1	0	0	0	0	0	#DIV/0!	#DIV/0!	#DIV/0!
[TG(47:2)]_C18:1	0	0	0	0	0	#DIV/0!	#DIV/0!	#DIV/0!
[TG(52:6)]_C14:0	0	0	0	0	0	#DIV/0!	#DIV/0!	#DIV/0!
[TG(54:7)]_C16:1	0	0	0	0	0	#DIV/0!	#DIV/0!	#DIV/0!
DG(32:2)_C18:2	0	0	0	0	0	#DIV/0!	#DIV/0!	#DIV/0!
LPC(17:1),LPC(O-18:1),LPC(P-	0	0	0	0	0	#DIV/0!	#DIV/0!	#DIV/0!
[TG(60:15),TG(59:8),TG(58:1)]_	0	0	0	0	0	#DIV/0!	#DIV/0!	#DIV/0!
CAR(16:0)	0	0	0	0	0	#DIV/0!	#DIV/0!	#DIV/0!
PG(36:3),PG(P-37:2); PG(36:3)	0	0	0	0	0	#DIV/0!	#DIV/0!	#DIV/0!
PC(50:0)	0	0	0	0	0	#DIV/0!	#DIV/0!	#DIV/0!
[TG(40:0)]_C14:0	0	0	0	0	0	#DIV/0!	#DIV/0!	#DIV/0!
[TG(47:6)]_C18:0	0	0	0	0	0	#DIV/0!	#DIV/0!	#DIV/0!
DG(44:1)_C18:1	0	0	0	0	0	#DIV/0!	#DIV/0!	#DIV/0!
FA(31:0)	0	0	0	0	0	#DIV/0!	#DIV/0!	#DIV/0!
PI(34:1),PI(O-35:1),PI(P-35:0)	0	0	0	0	0	#DIV/0!	#DIV/0!	#DIV/0!
[TG(49:3)]_C16:1	0	0	0	0	0	#DIV/0!	#DIV/0!	#DIV/0!
DG(38:8),DG(dO-40:8),DG(37:	0	0	0	0	0	#DIV/0!	#DIV/0!	#DIV/0!
[TG(38:0)]_C16:0	0	0	0	0	0	#DIV/0!	#DIV/0!	#DIV/0!

[TG(45:4)]_C18:1	0	0	0	0	0	#DIV/0!	#DIV/0!	#DIV/0!
[TG(50:9)]_C22:6	0	0	0	0	0	#DIV/0!	#DIV/0!	#DIV/0!
[TG(40:1)]_C16:0	0	0	0	0	0	#DIV/0!	#DIV/0!	#DIV/0!
[TG(60:14),TG(59:7),TG(58:0)]_	0	0	0	0	0	#DIV/0!	#DIV/0!	#DIV/0!
PG(30:0),PG(O-31:0); PG(30:0	0	0	0	0	0	#DIV/0!	#DIV/0!	#DIV/0!
[TG(54:12),TG(53:5)]_C22:5	0	0	0	0	0	#DIV/0!	#DIV/0!	#DIV/0!
DG(41:7),DG(40:0)_C16:0	0	0	0	0	0	#DIV/0!	#DIV/0!	#DIV/0!
[TG(44:4)]_C20:0	0	0	0	0	0	#DIV/0!	#DIV/0!	#DIV/0!
FA(28:3)	0	0	0	0	0	#DIV/0!	#DIV/0!	#DIV/0!
[TG(49:3)]_C20:0	0	0	0	0	0	#DIV/0!	#DIV/0!	#DIV/0!
[TG(45:4)]_C16:1	0	0	0	0	0	#DIV/0!	#DIV/0!	#DIV/0!
FA(25:4)	0	0	0	0	0	#DIV/0!	#DIV/0!	#DIV/0!
FA(30:3)	0	0	0	0	0	#DIV/0!	#DIV/0!	#DIV/0!
CE(50:3;O2)H	0	0	0	0	0	#DIV/0!	#DIV/0!	#DIV/0!
PG(26:0); PG(26:0)	0	0	0	0	0	#DIV/0!	#DIV/0!	#DIV/0!
PC(44:6)	0	0	0	0	0	#DIV/0!	#DIV/0!	#DIV/0!
[TG(40:1)]_C14:0	0	0	0	0	0	#DIV/0!	#DIV/0!	#DIV/0!
[TG(42:1)]_C16:1	0	0	0	0	0	#DIV/0!	#DIV/0!	#DIV/0!
[TG(45:1)]_C14:0	0	0	0	0	0	#DIV/0!	#DIV/0!	#DIV/0!
[TG(55:7)]_C22:6	0	0	0	0	0	#DIV/0!	#DIV/0!	#DIV/0!
CE(46:3;O2) NH4	0	0	0	0	0	#DIV/0!	#DIV/0!	#DIV/0!
FA(12:6)	0	0	0	0	0	#DIV/0!	#DIV/0!	#DIV/0!
FA(17:1)	0	0	0	0	0	#DIV/0!	#DIV/0!	#DIV/0!
FA(24:3)	0	0	0	0	0	#DIV/0!	#DIV/0!	#DIV/0!
FA(26:3)	0	0	0	0	0	#DIV/0!	#DIV/0!	#DIV/0!
FA(38:4)	0	0	0	0	0	#DIV/0!	#DIV/0!	#DIV/0!
FA(5:1)	0	0	0	0	0	#DIV/0!	#DIV/0!	#DIV/0!
DG(29:2)_C16:0	0	0	0	0	0	#DIV/0!	#DIV/0!	#DIV/0!
PG(28:0),PG(O-29:0); PG(28:0	0	0	0	0	0	#DIV/0!	#DIV/0!	#DIV/0!
FA(15:5)	0	0	0	0	0	#DIV/0!	#DIV/0!	#DIV/0!
FA(17:6)	0	0	0	0	0	#DIV/0!	#DIV/0!	#DIV/0!
FA(26:5)	0	0	0	0	0	#DIV/0!	#DIV/0!	#DIV/0!
[TG(57:8),TG(56:1)]_C16:1	0	0	0	0	0	#DIV/0!	#DIV/0!	#DIV/0!
LPS(O-20:0)	0	0	0	0	0	#DIV/0!	#DIV/0!	#DIV/0!
LPE(12:0)	0	0	0	0	0	#DIV/0!	#DIV/0!	#DIV/0!
[TG(48:5)]_C22:5	0	0	0	0	0	#DIV/0!	#DIV/0!	#DIV/0!
[TG(50:5)]_C22:5	0	0	0	0	0	#DIV/0!	#DIV/0!	#DIV/0!
[TG(59:12),TG(58:5)]_C22:5	0	0	0	0	0	#DIV/0!	#DIV/0!	#DIV/0!
DG(34:5)_C16:1	0	0	0	0	0	#DIV/0!	#DIV/0!	#DIV/0!
DG(42:0)_C16:0	0	0	0	0	0	#DIV/0!	#DIV/0!	#DIV/0!
FA(27:2)	0	0	0	0	0	#DIV/0!	#DIV/0!	#DIV/0!
DG(44:8),DG(43:1)_C16:0	0	0	0	0	0	#DIV/0!	#DIV/0!	#DIV/0!
CE(16:2) NH4	0	0	0	0	0	#DIV/0!	#DIV/0!	#DIV/0!
CE(16:1)K	0	0	0	0	0	#DIV/0!	#DIV/0!	#DIV/0!
LPC(22:5)	0	0	0	0	0	#DIV/0!	#DIV/0!	#DIV/0!
DG(40:8),DG(39:1)_C18:1	0	0	0	0	0	#DIV/0!	#DIV/0!	#DIV/0!
CE(18:0)H	0	0	0	0	0	#DIV/0!	#DIV/0!	#DIV/0!
CE(20:4)K	0	0	0	0	0	#DIV/0!	#DIV/0!	#DIV/0!
DG(31:1)_C16:0	0	0	0	0	0	#DIV/0!	#DIV/0!	#DIV/0!
CE(22:5)Na	0	0	0	0	0	#DIV/0!	#DIV/0!	#DIV/0!
DG(44:9),DG(43:2)_C18:2	0	0	0	0	0	#DIV/0!	#DIV/0!	#DIV/0!
DG(42:8),DG(41:1)_C18:1	0	0	0	0	0	#DIV/0!	#DIV/0!	#DIV/0!
[TG(37:0)]_C20:0	0	0	0	0	0	#DIV/0!	#DIV/0!	#DIV/0!
PE(40:4)	0	0	0	0	0	#DIV/0!	#DIV/0!	#DIV/0!
DG(30:2)_C18:2	0	0	0	0	0	#DIV/0!	#DIV/0!	#DIV/0!
CE(12:0)Na	0	0	0	0	0	#DIV/0!	#DIV/0!	#DIV/0!
CE(22:3)Na	0	0	0	0	0	#DIV/0!	#DIV/0!	#DIV/0!
DG(33:3)_C18:2	0	0	0	0	0	#DIV/0!	#DIV/0!	#DIV/0!
DG(44:0)_C16:0	0	0	0	0	0	#DIV/0!	#DIV/0!	#DIV/0!
FA(14:1)	0	0	0	0	0	#DIV/0!	#DIV/0!	#DIV/0!
DG(38:7),DG(37:0)_C18:0	0	0	0	0	0	#DIV/0!	#DIV/0!	#DIV/0!
PC(18:0),LPC(19:0),PC(O-19:(	0	0	0	0	0	#DIV/0!	#DIV/0!	#DIV/0!
PE(39:6),PE(O-40:6),PE(P-40:5	0	0	0	0	0	#DIV/0!	#DIV/0!	#DIV/0!
[TG(45:0)]_C18:0	0	0	0	0	0	#DIV/0!	#DIV/0!	#DIV/0!
[TG(51:4)]_C16:1	0	0	0	0	0	#DIV/0!	#DIV/0!	#DIV/0!
[TG(52:9),TG(51:2)]_C14:0	0	0	0	0	0	#DIV/0!	#DIV/0!	#DIV/0!
DG(26:0)_C16:0	0	0	0	0	0	#DIV/0!	#DIV/0!	#DIV/0!
CE(16:3) NH4	0	0	0	0	0	#DIV/0!	#DIV/0!	#DIV/0!
CE(22:5)K	0	0	0	0	0	#DIV/0!	#DIV/0!	#DIV/0!
SM(d16:0/14:0)	0	0	0	0	0	#DIV/0!	#DIV/0!	#DIV/0!
CE(20:3)K	0	0	0	0	0	#DIV/0!	#DIV/0!	#DIV/0!

CE(22:0) NH4	0	0	0	0	0	#DIV/0!	#DIV/0!	#DIV/0!
FA(26:1)	0	0	0	0	0	#DIV/0!	#DIV/0!	#DIV/0!
DG(33:1),DG(O-34:1)_C16:0	0	0	0	0	0	#DIV/0!	#DIV/0!	#DIV/0!
DG(39:7),DG(38:0),DG(dO-40:	0	0	0	0	0	#DIV/0!	#DIV/0!	#DIV/0!
[TG(44:4)]_C18:1	0	0	0	0	0	#DIV/0!	#DIV/0!	#DIV/0!
[TG(45:3)]_C20:0	0	0	0	0	0	#DIV/0!	#DIV/0!	#DIV/0!
[TG(51:9)]_C22:6	0	0	0	0	0	#DIV/0!	#DIV/0!	#DIV/0!
DG(32:2)_C18:0	0	0	0	0	0	#DIV/0!	#DIV/0!	#DIV/0!
FA(29:2)	0	0	0	0	0	#DIV/0!	#DIV/0!	#DIV/0!
[TG(39:0)]_C18:0	0	0	0	0	0	#DIV/0!	#DIV/0!	#DIV/0!
[TG(44:3)]_C16:1	0	0	0	0	0	#DIV/0!	#DIV/0!	#DIV/0!
DG(36:8),DG(35:1)_C16:0	0	0	0	0	0	#DIV/0!	#DIV/0!	#DIV/0!
DG(38:9),DG(dO-40:9),DG(37:	0	0	0	0	0	#DIV/0!	#DIV/0!	#DIV/0!
FA(16:1)	0	0	0	0	0	#DIV/0!	#DIV/0!	#DIV/0!
CAR(10:1)	0	0	0	0	0	#DIV/0!	#DIV/0!	#DIV/0!
CAR(20:4)	0	0	0	0	0	#DIV/0!	#DIV/0!	#DIV/0!
[TG(44:5)]_C18:2	0	0	0	0	0	#DIV/0!	#DIV/0!	#DIV/0!
FA(11:0)	0	0	0	0	0	#DIV/0!	#DIV/0!	#DIV/0!
FA(29:0)	0	0	0	0	0	#DIV/0!	#DIV/0!	#DIV/0!
CAR	0	0	0	0	0	#DIV/0!	#DIV/0!	#DIV/0!
CAR(5:0)	0	0	0	0	0	#DIV/0!	#DIV/0!	#DIV/0!
CAR(7:0)	0	0	0	0	0	#DIV/0!	#DIV/0!	#DIV/0!
CAR(8:1)	0	0	0	0	0	#DIV/0!	#DIV/0!	#DIV/0!
CAR(8:0)	0	0	0	0	0	#DIV/0!	#DIV/0!	#DIV/0!
CAR(9:0)	0	0	0	0	0	#DIV/0!	#DIV/0!	#DIV/0!
CAR(10:0)	0	0	0	0	0	#DIV/0!	#DIV/0!	#DIV/0!
CAR(11:0)	0	0	0	0	0	#DIV/0!	#DIV/0!	#DIV/0!
CAR(12:0)	0	0	0	0	0	#DIV/0!	#DIV/0!	#DIV/0!
CAR(16:2)	0	0	0	0	0	#DIV/0!	#DIV/0!	#DIV/0!
CAR(16:1)	0	0	0	0	0	#DIV/0!	#DIV/0!	#DIV/0!
CAR(18:4)	0	0	0	0	0	#DIV/0!	#DIV/0!	#DIV/0!
CAR(18:1)	0	0	0	0	0	#DIV/0!	#DIV/0!	#DIV/0!
LPC(12:0)	0	0	0	0	0	#DIV/0!	#DIV/0!	#DIV/0!
LPG(12:0); LPG(12:0)	0	0	0	0	0	#DIV/0!	#DIV/0!	#DIV/0!
CAR(22:6)	0	0	0	0	0	#DIV/0!	#DIV/0!	#DIV/0!
LPG(14:0); LPG(14:0)	0	0	0	0	0	#DIV/0!	#DIV/0!	#DIV/0!
LPE(18:3)	0	0	0	0	0	#DIV/0!	#DIV/0!	#DIV/0!
CAR(22:4)	0	0	0	0	0	#DIV/0!	#DIV/0!	#DIV/0!
LPC(16:1),LPC(P-17:0)	0	0	0	0	0	#DIV/0!	#DIV/0!	#DIV/0!
LPE(20:5)	0	0	0	0	0	#DIV/0!	#DIV/0!	#DIV/0!
LPE(20:3)	0	0	0	0	0	#DIV/0!	#DIV/0!	#DIV/0!
LPC(18:3)	0	0	0	0	0	#DIV/0!	#DIV/0!	#DIV/0!
LPE(22:5)	0	0	0	0	0	#DIV/0!	#DIV/0!	#DIV/0!
LPG(18:1); LPG(18:1)	0	0	0	0	0	#DIV/0!	#DIV/0!	#DIV/0!
PC(18:1),LPC(19:1),PC(O-19:1	0	0	0	0	0	#DIV/0!	#DIV/0!	#DIV/0!
LPC(20:5)	0	0	0	0	0	#DIV/0!	#DIV/0!	#DIV/0!
LPS(22:6)	0	0	0	0	0	#DIV/0!	#DIV/0!	#DIV/0!
LPG(22:6); LPG(22:6)	0	0	0	0	0	#DIV/0!	#DIV/0!	#DIV/0!
LPG(22:0); LPG(22:0)	0	0	0	0	0	#DIV/0!	#DIV/0!	#DIV/0!
LPI(19:1),LPI(P-20:0)	0	0	0	0	0	#DIV/0!	#DIV/0!	#DIV/0!
PG(26:1); PG(26:1)	0	0	0	0	0	#DIV/0!	#DIV/0!	#DIV/0!
SM(d18:0/13:0)	0	0	0	0	0	#DIV/0!	#DIV/0!	#DIV/0!
Cer(d18:1/25:0)	0	0	0	0	0	#DIV/0!	#DIV/0!	#DIV/0!
PG(28:1),PG(P-29:0); PG(28:1)	0	0	0	0	0	#DIV/0!	#DIV/0!	#DIV/0!
PG(29:0),PG(O-30:0); PG(29:0	0	0	0	0	0	#DIV/0!	#DIV/0!	#DIV/0!
PG(30:1),PG(O-31:1),PG(P-31:	0	0	0	0	0	#DIV/0!	#DIV/0!	#DIV/0!
PE(34:4)	0	0	0	0	0	#DIV/0!	#DIV/0!	#DIV/0!
PG(31:0),PG(O-32:0); PG(31:0	0	0	0	0	0	#DIV/0!	#DIV/0!	#DIV/0!
PG(32:2),PG(O-33:2),PG(P-33:	0	0	0	0	0	#DIV/0!	#DIV/0!	#DIV/0!
PC(33:5),PC(P-34:4)	0	0	0	0	0	#DIV/0!	#DIV/0!	#DIV/0!
PG(32:1),PG(O-33:1),PG(P-33:	0	0	0	0	0	#DIV/0!	#DIV/0!	#DIV/0!
PC(33:4),PC(O-34:4),PC(O-34	0	0	0	0	0	#DIV/0!	#DIV/0!	#DIV/0!
PC(34:5)	0	0	0	0	0	#DIV/0!	#DIV/0!	#DIV/0!
PG(33:1),PG(O-34:1),PG(P-34:	0	0	0	0	0	#DIV/0!	#DIV/0!	#DIV/0!
PC(34:4),PC(O-35:4)	0	0	0	0	0	#DIV/0!	#DIV/0!	#DIV/0!
PE(37:4),PE(O-38:4),PE(P-38:3	0	0	0	0	0	#DIV/0!	#DIV/0!	#DIV/0!
PG(33:0),PG(O-34:0); PG(33:0	0	0	0	0	0	#DIV/0!	#DIV/0!	#DIV/0!
PG(34:0),PG(O-35:0); PG(34:0	0	0	0	0	0	#DIV/0!	#DIV/0!	#DIV/0!
PG(35:6),PG(P-36:5); PG(35:6)	0	0	0	0	0	#DIV/0!	#DIV/0!	#DIV/0!
PG(36:1),PG(O-37:1),PG(P-37:	0	0	0	0	0	#DIV/0!	#DIV/0!	#DIV/0!
PG(37:7),PG(P-38:6),PG(36:0),	0	0	0	0	0	#DIV/0!	#DIV/0!	#DIV/0!

PE(40:3)	0	0	0	0	0	#DIV/0!	#DIV/0!	#DIV/0!
PE(42:5)	0	0	0	0	0	#DIV/0!	#DIV/0!	#DIV/0!
PS(40:4)	0	0	0	0	0	#DIV/0!	#DIV/0!	#DIV/0!
PE(44:8),PE(43:1)	0	0	0	0	0	#DIV/0!	#DIV/0!	#DIV/0!
PE(44:7),PE(43:0)	0	0	0	0	0	#DIV/0!	#DIV/0!	#DIV/0!
PS(O-42:0)	0	0	0	0	0	#DIV/0!	#DIV/0!	#DIV/0!
PS(42:5)	0	0	0	0	0	#DIV/0!	#DIV/0!	#DIV/0!
PS(43:1)	0	0	0	0	0	#DIV/0!	#DIV/0!	#DIV/0!
PS(43:0)	0	0	0	0	0	#DIV/0!	#DIV/0!	#DIV/0!
PS(44:6)	0	0	0	0	0	#DIV/0!	#DIV/0!	#DIV/0!
PI(40:6)	0	0	0	0	0	#DIV/0!	#DIV/0!	#DIV/0!
PI(40:5)	0	0	0	0	0	#DIV/0!	#DIV/0!	#DIV/0!
[TG(63:13),TG(62:6)]_C22:6	0	0	0	0	0	#DIV/0!	#DIV/0!	#DIV/0!
[TG(38:1)]_C16:1	0	0	0	0	0	#DIV/0!	#DIV/0!	#DIV/0!
[TG(39:1)]_C18:1	0	0	0	0	0	#DIV/0!	#DIV/0!	#DIV/0!
[TG(39:1)]_C16:0	0	0	0	0	0	#DIV/0!	#DIV/0!	#DIV/0!
[TG(39:1)]_C16:1	0	0	0	0	0	#DIV/0!	#DIV/0!	#DIV/0!
[TG(39:1)]_C14:0	0	0	0	0	0	#DIV/0!	#DIV/0!	#DIV/0!
[TG(39:0)]_C14:0	0	0	0	0	0	#DIV/0!	#DIV/0!	#DIV/0!
[TG(40:2)]_C18:1	0	0	0	0	0	#DIV/0!	#DIV/0!	#DIV/0!
[TG(40:2)]_C16:1	0	0	0	0	0	#DIV/0!	#DIV/0!	#DIV/0!
[TG(40:1)]_C18:0	0	0	0	0	0	#DIV/0!	#DIV/0!	#DIV/0!
[TG(40:1)]_C16:1	0	0	0	0	0	#DIV/0!	#DIV/0!	#DIV/0!
[TG(41:2)]_C18:1	0	0	0	0	0	#DIV/0!	#DIV/0!	#DIV/0!
[TG(41:1)]_C18:1	0	0	0	0	0	#DIV/0!	#DIV/0!	#DIV/0!
[TG(41:1)]_C16:0	0	0	0	0	0	#DIV/0!	#DIV/0!	#DIV/0!
[TG(41:1)]_C16:1	0	0	0	0	0	#DIV/0!	#DIV/0!	#DIV/0!
[TG(41:1)]_C14:0	0	0	0	0	0	#DIV/0!	#DIV/0!	#DIV/0!
[TG(41:0)]_C18:0	0	0	0	0	0	#DIV/0!	#DIV/0!	#DIV/0!
[TG(41:0)]_C14:0	0	0	0	0	0	#DIV/0!	#DIV/0!	#DIV/0!
[TG(42:2)]_C16:1	0	0	0	0	0	#DIV/0!	#DIV/0!	#DIV/0!
[TG(42:2)]_C14:0	0	0	0	0	0	#DIV/0!	#DIV/0!	#DIV/0!
[TG(43:2)]_C16:1	0	0	0	0	0	#DIV/0!	#DIV/0!	#DIV/0!
[TG(43:1)]_C14:0	0	0	0	0	0	#DIV/0!	#DIV/0!	#DIV/0!
[TG(43:0)]_C18:0	0	0	0	0	0	#DIV/0!	#DIV/0!	#DIV/0!
[TG(43:0)]_C14:0	0	0	0	0	0	#DIV/0!	#DIV/0!	#DIV/0!
[TG(44:5)]_C22:5	0	0	0	0	0	#DIV/0!	#DIV/0!	#DIV/0!
[TG(44:4)]_C16:1	0	0	0	0	0	#DIV/0!	#DIV/0!	#DIV/0!
[TG(45:3)]_C16:1	0	0	0	0	0	#DIV/0!	#DIV/0!	#DIV/0!
[TG(45:2)]_C18:1	0	0	0	0	0	#DIV/0!	#DIV/0!	#DIV/0!
[TG(45:2)]_C18:2	0	0	0	0	0	#DIV/0!	#DIV/0!	#DIV/0!
[TG(45:2)]_C14:0	0	0	0	0	0	#DIV/0!	#DIV/0!	#DIV/0!
[TG(45:1)]_C18:0	0	0	0	0	0	#DIV/0!	#DIV/0!	#DIV/0!
[TG(46:6)]_C18:0	0	0	0	0	0	#DIV/0!	#DIV/0!	#DIV/0!
[TG(46:6)]_C14:0	0	0	0	0	0	#DIV/0!	#DIV/0!	#DIV/0!
[TG(47:3)]_C18:2	0	0	0	0	0	#DIV/0!	#DIV/0!	#DIV/0!
[TG(47:3)]_C16:1	0	0	0	0	0	#DIV/0!	#DIV/0!	#DIV/0!
[TG(48:8),TG(47:1)]_C18:0	0	0	0	0	0	#DIV/0!	#DIV/0!	#DIV/0!
[TG(48:7),TG(47:0)]_C18:0	0	0	0	0	0	#DIV/0!	#DIV/0!	#DIV/0!
[TG(48:7)]_C16:1	0	0	0	0	0	#DIV/0!	#DIV/0!	#DIV/0!
[TG(48:6)]_C14:0	0	0	0	0	0	#DIV/0!	#DIV/0!	#DIV/0!
[TG(48:4)]_C16:1	0	0	0	0	0	#DIV/0!	#DIV/0!	#DIV/0!
[TG(49:6)]_C14:0	0	0	0	0	0	#DIV/0!	#DIV/0!	#DIV/0!
[TG(49:4)]_C16:1	0	0	0	0	0	#DIV/0!	#DIV/0!	#DIV/0!
[TG(50:9),TG(49:2)]_C18:0	0	0	0	0	0	#DIV/0!	#DIV/0!	#DIV/0!
[TG(50:8),TG(49:1)]_C20:0	0	0	0	0	0	#DIV/0!	#DIV/0!	#DIV/0!
[TG(50:8),TG(49:1)]_C18:0	0	0	0	0	0	#DIV/0!	#DIV/0!	#DIV/0!
[TG(50:7)]_C16:1	0	0	0	0	0	#DIV/0!	#DIV/0!	#DIV/0!
[TG(50:7),TG(49:0)]_C14:0	0	0	0	0	0	#DIV/0!	#DIV/0!	#DIV/0!
[TG(50:6)]_C16:0	0	0	0	0	0	#DIV/0!	#DIV/0!	#DIV/0!
[TG(51:7)]_C22:5	0	0	0	0	0	#DIV/0!	#DIV/0!	#DIV/0!
[TG(52:8),TG(51:1)]_C16:1	0	0	0	0	0	#DIV/0!	#DIV/0!	#DIV/0!
[TG(52:8),TG(51:1)]_C14:0	0	0	0	0	0	#DIV/0!	#DIV/0!	#DIV/0!
[TG(52:7),TG(51:0)]_C14:0	0	0	0	0	0	#DIV/0!	#DIV/0!	#DIV/0!
[TG(53:8),TG(52:1)]_C14:0	0	0	0	0	0	#DIV/0!	#DIV/0!	#DIV/0!
[TG(53:7)]_C16:1	0	0	0	0	0	#DIV/0!	#DIV/0!	#DIV/0!
[TG(53:7),TG(52:0)]_C14:0	0	0	0	0	0	#DIV/0!	#DIV/0!	#DIV/0!
[TG(53:6)]_C16:0	0	0	0	0	0	#DIV/0!	#DIV/0!	#DIV/0!
[TG(54:11),TG(53:4)]_C16:1	0	0	0	0	0	#DIV/0!	#DIV/0!	#DIV/0!
[TG(54:10),TG(53:3)]_C16:1	0	0	0	0	0	#DIV/0!	#DIV/0!	#DIV/0!
[TG(54:9),TG(53:2)]_C16:1	0	0	0	0	0	#DIV/0!	#DIV/0!	#DIV/0!

[TG(54:8),TG(53:1)]_C16:1	0	0	0	0	0	#DIV/0!	#DIV/0!	#DIV/0!
[TG(54:7),TG(53:0)]_C14:0	0	0	0	0	0	#DIV/0!	#DIV/0!	#DIV/0!
[TG(55:9),TG(54:2)]_C16:1	0	0	0	0	0	#DIV/0!	#DIV/0!	#DIV/0!
[TG(55:8),TG(54:1)]_C16:1	0	0	0	0	0	#DIV/0!	#DIV/0!	#DIV/0!
[TG(56:10),TG(55:3)]_C16:1	0	0	0	0	0	#DIV/0!	#DIV/0!	#DIV/0!
[TG(56:9),TG(55:2)]_C16:1	0	0	0	0	0	#DIV/0!	#DIV/0!	#DIV/0!
[TG(56:8),TG(55:1)]_C16:1	0	0	0	0	0	#DIV/0!	#DIV/0!	#DIV/0!
[TG(56:8),TG(55:1)]_C14:0	0	0	0	0	0	#DIV/0!	#DIV/0!	#DIV/0!
[TG(56:7)]_C16:1	0	0	0	0	0	#DIV/0!	#DIV/0!	#DIV/0!
[TG(56:7),TG(55:0)]_C14:0	0	0	0	0	0	#DIV/0!	#DIV/0!	#DIV/0!
[TG(57:12),TG(56:5)]_C16:1	0	0	0	0	0	#DIV/0!	#DIV/0!	#DIV/0!
[TG(57:10),TG(56:3)]_C16:1	0	0	0	0	0	#DIV/0!	#DIV/0!	#DIV/0!
[TG(57:9),TG(56:2)]_C16:1	0	0	0	0	0	#DIV/0!	#DIV/0!	#DIV/0!
[TG(57:8),TG(56:1)]_C14:0	0	0	0	0	0	#DIV/0!	#DIV/0!	#DIV/0!
[TG(58:14),TG(57:7),TG(56:0)]_	0	0	0	0	0	#DIV/0!	#DIV/0!	#DIV/0!
[TG(58:14),TG(57:7)]_C16:1	0	0	0	0	0	#DIV/0!	#DIV/0!	#DIV/0!
[TG(58:14),TG(57:7),TG(56:0)]_	0	0	0	0	0	#DIV/0!	#DIV/0!	#DIV/0!
[TG(58:9),TG(57:2)]_C16:1	0	0	0	0	0	#DIV/0!	#DIV/0!	#DIV/0!
[TG(58:8),TG(57:1)]_C16:0	0	0	0	0	0	#DIV/0!	#DIV/0!	#DIV/0!
[TG(58:8),TG(57:1)]_C16:1	0	0	0	0	0	#DIV/0!	#DIV/0!	#DIV/0!
[TG(58:8),TG(57:1)]_C14:0	0	0	0	0	0	#DIV/0!	#DIV/0!	#DIV/0!
[TG(58:7),TG(57:0)]_C16:0	0	0	0	0	0	#DIV/0!	#DIV/0!	#DIV/0!
[TG(59:10),TG(58:3)]_C16:1	0	0	0	0	0	#DIV/0!	#DIV/0!	#DIV/0!
[TG(59:9),TG(58:2)]_C16:1	0	0	0	0	0	#DIV/0!	#DIV/0!	#DIV/0!
[TG(60:15),TG(59:8),TG(58:1)]_	0	0	0	0	0	#DIV/0!	#DIV/0!	#DIV/0!
[TG(60:15),TG(59:8),TG(58:1)]_	0	0	0	0	0	#DIV/0!	#DIV/0!	#DIV/0!
[TG(60:15),TG(59:8),TG(58:1)]_	0	0	0	0	0	#DIV/0!	#DIV/0!	#DIV/0!
[TG(60:14),TG(59:7),TG(58:0)]_	0	0	0	0	0	#DIV/0!	#DIV/0!	#DIV/0!
[TG(60:12),TG(59:5)]_C18:2	0	0	0	0	0	#DIV/0!	#DIV/0!	#DIV/0!
[TG(60:9),TG(59:2)]_C18:1	0	0	0	0	0	#DIV/0!	#DIV/0!	#DIV/0!
[TG(60:9),TG(59:2)]_C16:1	0	0	0	0	0	#DIV/0!	#DIV/0!	#DIV/0!
[TG(60:8),TG(59:1)]_C18:1	0	0	0	0	0	#DIV/0!	#DIV/0!	#DIV/0!
[TG(60:8),TG(59:1)]_C16:0	0	0	0	0	0	#DIV/0!	#DIV/0!	#DIV/0!
[TG(60:8),TG(59:1)]_C16:1	0	0	0	0	0	#DIV/0!	#DIV/0!	#DIV/0!
[TG(61:14),TG(60:7),TG(59:0)]_	0	0	0	0	0	#DIV/0!	#DIV/0!	#DIV/0!
[TG(61:13),TG(60:6)]_C20:0	0	0	0	0	0	#DIV/0!	#DIV/0!	#DIV/0!
[TG(61:11),TG(60:4)]_C20:4	0	0	0	0	0	#DIV/0!	#DIV/0!	#DIV/0!
[TG(61:11),TG(60:4)]_C18:0	0	0	0	0	0	#DIV/0!	#DIV/0!	#DIV/0!
[TG(61:10),TG(60:3)]_C16:1	0	0	0	0	0	#DIV/0!	#DIV/0!	#DIV/0!
[TG(62:15),TG(61:8),TG(60:1)]_	0	0	0	0	0	#DIV/0!	#DIV/0!	#DIV/0!
[TG(62:15),TG(61:8),TG(60:1)]_	0	0	0	0	0	#DIV/0!	#DIV/0!	#DIV/0!
DG(26:0)_C18:0	0	0	0	0	0	#DIV/0!	#DIV/0!	#DIV/0!
DG(28:2)_C18:2	0	0	0	0	0	#DIV/0!	#DIV/0!	#DIV/0!
DG(29:2)_C18:1	0	0	0	0	0	#DIV/0!	#DIV/0!	#DIV/0!
DG(29:1)_C18:1	0	0	0	0	0	#DIV/0!	#DIV/0!	#DIV/0!
DG(29:1)_C16:0	0	0	0	0	0	#DIV/0!	#DIV/0!	#DIV/0!
DG(31:2),DG(P-14:0/18:1)_C1	0	0	0	0	0	#DIV/0!	#DIV/0!	#DIV/0!
CE(12:0)H	0	0	0	0	0	#DIV/0!	#DIV/0!	#DIV/0!
DG(31:1)_C16:1	0	0	0	0	0	#DIV/0!	#DIV/0!	#DIV/0!
DG(31:0)_C16:0	0	0	0	0	0	#DIV/0!	#DIV/0!	#DIV/0!
DG(32:2)_C16:1	0	0	0	0	0	#DIV/0!	#DIV/0!	#DIV/0!
DG(33:5)_C18:1	0	0	0	0	0	#DIV/0!	#DIV/0!	#DIV/0!
DG(33:2)_C18:2	0	0	0	0	0	#DIV/0!	#DIV/0!	#DIV/0!
DG(33:2)_C16:0	0	0	0	0	0	#DIV/0!	#DIV/0!	#DIV/0!
DG(33:2)_C16:1	0	0	0	0	0	#DIV/0!	#DIV/0!	#DIV/0!
DG(35:3)_C18:1	0	0	0	0	0	#DIV/0!	#DIV/0!	#DIV/0!
DG(dO-38:9),DG(35:2)_C18:1	0	0	0	0	0	#DIV/0!	#DIV/0!	#DIV/0!
DG(dO-38:9),DG(35:2)_C18:2	0	0	0	0	0	#DIV/0!	#DIV/0!	#DIV/0!
DG(O-38:8),DG(36:1)_C16:0	0	0	0	0	0	#DIV/0!	#DIV/0!	#DIV/0!
DG(38:9),DG(dO-40:9),DG(37:	0	0	0	0	0	#DIV/0!	#DIV/0!	#DIV/0!
DG(38:8),DG(dO-40:8),DG(37:	0	0	0	0	0	#DIV/0!	#DIV/0!	#DIV/0!
DG(38:8),DG(dO-40:8),DG(37:	0	0	0	0	0	#DIV/0!	#DIV/0!	#DIV/0!
DG(38:7)_C18:1	0	0	0	0	0	#DIV/0!	#DIV/0!	#DIV/0!
CE(16:3)K	0	0	0	0	0	#DIV/0!	#DIV/0!	#DIV/0!
CE(16:2)K	0	0	0	0	0	#DIV/0!	#DIV/0!	#DIV/0!
DG(39:8),DG(O-40:8),DG(38:1	0	0	0	0	0	#DIV/0!	#DIV/0!	#DIV/0!
DG(39:8),DG(O-40:8),DG(38:1	0	0	0	0	0	#DIV/0!	#DIV/0!	#DIV/0!
	0	0	0	0	0	#DIV/0!	#DIV/0!	#DIV/0!
CE(17:0)K	0	0	0	0	0	#DIV/0!	#DIV/0!	#DIV/0!
DG(40:9),DG(39:2)_C18:1	0	0	0	0	0	#DIV/0!	#DIV/0!	#DIV/0!
DG(40:8),DG(39:1)_C16:0	0	0	0	0	0	#DIV/0!	#DIV/0!	#DIV/0!

DG(40:8),DG(39:1)_C16:1	0	0	0	0	0	#DIV/0!	#DIV/0!	#DIV/0!
DG(40:7)_C18:1	0	0	0	0	0	#DIV/0!	#DIV/0!	#DIV/0!
DG(40:2)_C18:1	0	0	0	0	0	#DIV/0!	#DIV/0!	#DIV/0!
DG(40:2)_C16:1	0	0	0	0	0	#DIV/0!	#DIV/0!	#DIV/0!
DG(40:1)_C18:1	0	0	0	0	0	#DIV/0!	#DIV/0!	#DIV/0!
DG(40:1)_C16:0	0	0	0	0	0	#DIV/0!	#DIV/0!	#DIV/0!
DG(40:1)_C16:1	0	0	0	0	0	#DIV/0!	#DIV/0!	#DIV/0!
DG(41:7)_C18:1	0	0	0	0	0	#DIV/0!	#DIV/0!	#DIV/0!
DG(42:10),DG(41:3)_C18:1	0	0	0	0	0	#DIV/0!	#DIV/0!	#DIV/0!
DG(42:9),DG(41:2)_C18:1	0	0	0	0	0	#DIV/0!	#DIV/0!	#DIV/0!
DG(42:9),DG(41:2)_C18:2	0	0	0	0	0	#DIV/0!	#DIV/0!	#DIV/0!
DG(42:8),DG(41:1)_C16:1	0	0	0	0	0	#DIV/0!	#DIV/0!	#DIV/0!
DG(42:7),DG(41:0)_C18:0	0	0	0	0	0	#DIV/0!	#DIV/0!	#DIV/0!
DG(42:7)_C18:1	0	0	0	0	0	#DIV/0!	#DIV/0!	#DIV/0!
DG(42:2)_C18:1	0	0	0	0	0	#DIV/0!	#DIV/0!	#DIV/0!
DG(42:2)_C16:1	0	0	0	0	0	#DIV/0!	#DIV/0!	#DIV/0!
DG(42:1)_C18:1	0	0	0	0	0	#DIV/0!	#DIV/0!	#DIV/0!
DG(42:1)_C16:0	0	0	0	0	0	#DIV/0!	#DIV/0!	#DIV/0!
DG(42:1)_C16:1	0	0	0	0	0	#DIV/0!	#DIV/0!	#DIV/0!
DG(42:0)_C18:0	0	0	0	0	0	#DIV/0!	#DIV/0!	#DIV/0!
DG(43:6)_C16:0	0	0	0	0	0	#DIV/0!	#DIV/0!	#DIV/0!
DG(44:9),DG(43:2)_C16:1	0	0	0	0	0	#DIV/0!	#DIV/0!	#DIV/0!
DG(44:8),DG(43:1)_C16:1	0	0	0	0	0	#DIV/0!	#DIV/0!	#DIV/0!
DG(44:7)_C18:1	0	0	0	0	0	#DIV/0!	#DIV/0!	#DIV/0!
DG(44:6)_C16:0	0	0	0	0	0	#DIV/0!	#DIV/0!	#DIV/0!
DG(44:2)_C18:1	0	0	0	0	0	#DIV/0!	#DIV/0!	#DIV/0!
DG(44:2)_C16:1	0	0	0	0	0	#DIV/0!	#DIV/0!	#DIV/0!
DG(44:1)_C16:0	0	0	0	0	0	#DIV/0!	#DIV/0!	#DIV/0!
DG(44:1)_C16:1	0	0	0	0	0	#DIV/0!	#DIV/0!	#DIV/0!
DG(44:0)_C18:0	0	0	0	0	0	#DIV/0!	#DIV/0!	#DIV/0!
FA(7:1)	0	0	0	0	0	#DIV/0!	#DIV/0!	#DIV/0!
FA(7:0)	0	0	0	0	0	#DIV/0!	#DIV/0!	#DIV/0!
FA(8:6)	0	0	0	0	0	#DIV/0!	#DIV/0!	#DIV/0!
FA(8:1)	0	0	0	0	0	#DIV/0!	#DIV/0!	#DIV/0!
FA(8:0)	0	0	0	0	0	#DIV/0!	#DIV/0!	#DIV/0!
FA(10:6)	0	0	0	0	0	#DIV/0!	#DIV/0!	#DIV/0!
FA(10:1)	0	0	0	0	0	#DIV/0!	#DIV/0!	#DIV/0!
FA(10:0); FA(10:0)	0	0	0	0	0	#DIV/0!	#DIV/0!	#DIV/0!
FA(11:6)	0	0	0	0	0	#DIV/0!	#DIV/0!	#DIV/0!
FA(12:5)	0	0	0	0	0	#DIV/0!	#DIV/0!	#DIV/0!
FA(12:0)	0	0	0	0	0	#DIV/0!	#DIV/0!	#DIV/0!
FA(14:7)	0	0	0	0	0	#DIV/0!	#DIV/0!	#DIV/0!
FA(13:0)	0	0	0	0	0	#DIV/0!	#DIV/0!	#DIV/0!
FA(14:0)	0	0	0	0	0	#DIV/0!	#DIV/0!	#DIV/0!
FA(15:6)	0	0	0	0	0	#DIV/0!	#DIV/0!	#DIV/0!
FA(16:5)	0	0	0	0	0	#DIV/0!	#DIV/0!	#DIV/0!
FA(17:4)	0	0	0	0	0	#DIV/0!	#DIV/0!	#DIV/0!
FA(18:7)	0	0	0	0	0	#DIV/0!	#DIV/0!	#DIV/0!
FA(17:0)	0	0	0	0	0	#DIV/0!	#DIV/0!	#DIV/0!
FA(19:4)	0	0	0	0	0	#DIV/0!	#DIV/0!	#DIV/0!
FA(20:4)	0	0	0	0	0	#DIV/0!	#DIV/0!	#DIV/0!
FA(21:3)	0	0	0	0	0	#DIV/0!	#DIV/0!	#DIV/0!
FA(22:4)	0	0	0	0	0	#DIV/0!	#DIV/0!	#DIV/0!
FA(22:3)	0	0	0	0	0	#DIV/0!	#DIV/0!	#DIV/0!
FA(28:7)	0	0	0	0	0	#DIV/0!	#DIV/0!	#DIV/0!
FA(28:5)	0	0	0	0	0	#DIV/0!	#DIV/0!	#DIV/0!
FA(29:3)	0	0	0	0	0	#DIV/0!	#DIV/0!	#DIV/0!
FA(30:5)	0	0	0	0	0	#DIV/0!	#DIV/0!	#DIV/0!
FA(31:1)	0	0	0	0	0	#DIV/0!	#DIV/0!	#DIV/0!
FA(32:6)	0	0	0	0	0	#DIV/0!	#DIV/0!	#DIV/0!
FA(32:4)	0	0	0	0	0	#DIV/0!	#DIV/0!	#DIV/0!
FA(34:5)	0	0	0	0	0	#DIV/0!	#DIV/0!	#DIV/0!
FA(46:0)	0	0	0	0	0	#DIV/0!	#DIV/0!	#DIV/0!
FA(4:0)	0	0	0	0	0	#DIV/0!	#DIV/0!	#DIV/0!
						#DIV/0!	#DIV/0!	#DIV/0!
