# Supplementary material for: Elucidating the Influence of Serum Concentration, Sex, and Particle Size on Iron Oxide Nanoparticle–Lipid Biocorona Formation
Source: Nanomaterials (Basel). 2026 Jun 1;16(11):683. doi: 10.3390/nano16110683 (PMC13258708; doi:10.3390/nano16110683)
Supplement: Supplementary file 1 [file nanomaterials-16-00683-s001.zip › nanomaterials-4334647-supplementary - 副本/Table S7. Comparison of Lipid Corona Profiles Between Sexes.pdf]

Table S7. Comparison of Lipid Corona Profiles Between Sexes
50 nm 5% BC Samples

Unique Lipids in Males	Unique Lipds in Females	Shared Lipids
[TG(39:0)]_C20:0	FA(28:6)	[TG(53:9),TG(52:2)]_C18:0
[TG(54:6)]_C18:1	[TG(42:0)]_C16:0	[TG(54:6)]_C18:2
LPG(20:0); LPG(20:0)	FA(18:0)	PS(25:0)
[TG(54:5)]_C18:3	CAR(18:3)	DG(30:3)_C16:1
[TG(52:4)]_C18:3	LPG(19:0),LPG(O-20:0); LPG(19:0),LPG(O-20:0)	[TG(53:7),TG(52:0)]_C16:0
[TG(50:3)]_C14:0		[TG(50:3)]_C18:2
[TG(49:8),TG(48:1)]_C18:1		DG(30:2)_C16:1
[TG(54:9),TG(53:2)]_C18:1		[TG(49:6)]_C16:0
PC(32:0),PC(O-33:0)		[TG(53:7)]_C18:1
PC(34:0),PC(O-35:0)		[TG(55:11),TG(54:4)]_C18:2
[TG(48:2)]_C14:0		[TG(51:8),TG(50:1)]_C18:0
FA(17:2)		[TG(54:5)]_C18:1
PC(34:1),PC(O-35:1),PC(P-35:0)		[TG(52:4)]_C16:1
SM(d18:2/22:1)		CAR(20:0)
SM(d16:0/22:0)		[TG(53:9),TG(52:2)]_C18:2
[TG(52:5)]_C18:3		[TG(51:9),TG(50:2)]_C16:0
[TG(50:4)]_C18:2		[TG(51:7),TG(50:0)]_C16:0
[TG(48:2)]_C18:2		FA(35:0)
[TG(44:1)]_C16:0		DG(36:8),DG(35:1)_C16:1
[TG(46:2)]_C18:1		FA(22:7)
[TG(50:3)]_C16:0		[TG(53:9),TG(52:2)]_C18:1
[TG(54:8),TG(53:1)]_C18:1		FA(22:1)
[TG(52:9),TG(51:2)]_C18:1		[TG(55:9),TG(54:2)]_C18:1
[TG(51:7)]_C18:1		Cer(d18:0/17:0)
[TG(50:7),TG(49:0)]_C18:0		[TG(53:8),TG(52:1)]_C16:0
[TG(48:2)]_C16:0		[TG(55:10),TG(54:3)]_C18:1
CAR(14:2)		[TG(51:9),TG(50:2)]_C16:1
[TG(54:5)]_C20:4		DG(36:7),DG(35:0)_C16:0
[TG(46:0)]_C14:0		[TG(52:4)]_C18:1
PC(36:3),PC(P-37:2)		[TG(49:7),TG(48:0)]_C16:0
LPC(18:0),PC(O-18:0),LPC(O-19:0)		[TG(49:8),TG(48:1)]_C14:0
PC(38:5)		DG(41:6)_C16:1
		[TG(49:7),TG(48:0)]_C18:0
		[TG(51:8),TG(50:1)]_C16:0
		[TG(55:9),TG(54:2)]_C18:0
		PC(36:4),PC(O-37:4)
		FA(19:2)
		[TG(53:10),TG(52:3)]_C18:2
		[TG(51:9),TG(50:2)]_C18:2
		[TG(53:7),TG(52:0)]_C18:0
		PC(34:2),PC(O-35:2),PC(P-35:1)
		DG(41:5)_C16:0
		[TG(49:8),TG(48:1)]_C16:0
		[TG(52:4)]_C18:2
		CE(15:1) NH4
		[TG(49:7),TG(48:0)]_C14:0
		[TG(55:10),TG(54:3)]_C18:0
		[TG(53:10),TG(52:3)]_C18:1
		Cer(d14:2(4E,6E)/16:0)
		[TG(51:8),TG(50:1)]_C18:1
		FA(21:0)
		[TG(53:8),TG(52:1)]_C18:1
		[TG(50:7),TG(49:0)]_C16:0
		[TG(51:7),TG(50:0)]_C18:0
		PC(40:6)
		DG(42:11),DG(41:4)_C16:0
		[TG(54:5)]_C18:2
		[TG(55:10),TG(54:3)]_C18:2
		[TG(55:8),TG(54:1)]_C18:1
		[TG(53:8),TG(52:1)]_C18:0
		[TG(53:10),TG(52:3)]_C16:0
		[TG(48:2)]_C18:1
		PC(38:6)
		FA(18:3)
		[TG(55:8),TG(54:1)]_C18:0
		FA(22:0)
		[TG(51:9),TG(50:2)]_C14:0
		[TG(52:4)]_C16:0
		[TG(48:2)]_C16:1
		[TG(52:5)]_C18:2
		[TG(50:3)]_C18:1
		PS(O-29:0)
		[TG(53:9),TG(52:2)]_C16:0
		DG(30:2)_C16:0
		[TG(51:9),TG(50:2)]_C18:1
		[TG(50:3)]_C16:1
		DG(36:6)_C16:0
		[TG(53:10),TG(52:3)]_C16:1

		[TG(55:11),TG(54:4)]_C18:1
		FA(20:0)
		[TG(46:0)]_C16:0
		DG(36:7)_C16:1
		[TG(55:11),TG(54:4)]_C18:0
		PG(16:0),LPG(17:0),LPG(O-18:0); PG(16:0),LPG(17:0),LPG(O-18:0)

Comparison of Lipid Corona Profiles Between Sexes

Table S7. Comparison of Lipid Corona Profiles Between Sexes

Unique Lipids in Males	Unique Lipds in Females	Shared Lipids
PC(38:3)	FA(21:1)	[TG(53:9),TG(52:2)]_C18:0
[TG(46:1)]_C14:0	DG(30:2)_C16:1	[TG(54:6)]_C18:2
SM(d16:1/17:0)	FA(19:0)	FA(28:6)
[TG(48:3)]_C16:0	FA(17:2)	[TG(53:7),TG(52:0)]_C16:0
[TG(57:12),TG(56:5)]_C18:1	FA(26:6)	[TG(50:3)]_C18:2
PC(35:2),PC(O-36:2),PC(P-36:1)	DG(32:5)_C18:1	[TG(49:6)]_C16:0
[TG(50:4)]_C14:0	FA(16:0)	[TG(53:7)]_C18:1
[TG(42:0)]_C16:0	PS(25:0)	[TG(51:8),TG(50:1)]_C18:0
[TG(54:5)]_C18:3	CAR(20:0)	[TG(52:4)]_C16:1
PC(O-38:8),PC(36:1),PC(O-37:1),PC(P-37:0)	FA(22:7)	[TG(52:4)]_C18:3
SM(d16:1/16:0)	FA(18:0)	[TG(51:7),TG(50:0)]_C16:0
[TG(55:11),TG(54:4)]_C16:0	DG(41:6)_C16:1	FA(35:0)
[TG(55:9),TG(54:2)]_C16:0	FA(6:0)	DG(36:8),DG(35:1)_C16:1
[TG(52:8),TG(51:1)]_C16:0	FA(19:2)	PC(O-38:9),PC(36:2),PC(O-37:2),PC(P-37:1)
[TG(49:8),TG(48:1)]_C18:0	FA(21:0)	PC(32:0),PC(O-33:0)
[TG(48:2)]_C14:0	FA(22:0)	[TG(53:9),TG(52:2)]_C18:1
[TG(52:4)]_C20:4	FA(28:0)	[TG(55:9),TG(54:2)]_C18:1
PC(30:0),PC(O-31:0)	[TG(40:0)]_C16:0	[TG(51:9),TG(50:2)]_C16:1
[TG(46:1)]_C18:1	FA(20:0)	DG(36:7),DG(35:0)_C16:0
SM(d16:1/24:0)		PC(36:5)
CAR(18:3)		[TG(52:4)]_C18:1
LPC(20:2),PC(O-20:2)		[TG(49:8),TG(48:1)]_C14:0
[TG(54:6)]_C20:4		[TG(51:8),TG(50:1)]_C16:0
SM(d18:2/22:1)		[TG(55:9),TG(54:2)]_C18:0
[TG(52:5)]_C16:0		[TG(54:10),TG(53:3)]_C18:1
[TG(50:4)]_C18:2		[TG(53:10),TG(52:3)]_C18:2
[TG(48:2)]_C18:2		[TG(53:7),TG(52:0)]_C18:0
[TG(56:6)]_C20:4		[TG(51:9),TG(50:2)]_C18:2
[TG(49:8),TG(48:1)]_C16:1		[TG(52:5)]_C18:3
CAR(14:1)		DG(41:5)_C16:0
PC(29:1),PC(O-30:1),PC(P-30:0)		[TG(49:8),TG(48:1)]_C16:0
PC(28:0),PC(O-29:0)		[TG(52:4)]_C18:2
[TG(48:3)]_C18:2		[TG(55:10),TG(54:3)]_C18:0
[TG(48:2)]_C16:0		[TG(50:3)]_C16:0
SM(d16:0/20:0)		[TG(53:10),TG(52:3)]_C18:1
PC(40:4)		Cer(d14:2(4E,6E)/16:0)
[TG(50:4)]_C16:0		[TG(53:8),TG(52:1)]_C18:1
[TG(46:1)]_C16:0		DG(42:11),DG(41:4)_C16:0
PC(35:4),PC(O-36:4),PC(P-36:3)		[TG(55:10),TG(54:3)]_C18:2
[TG(50:8),TG(49:1)]_C16:0		[TG(55:8),TG(54:1)]_C18:1
[TG(50:4)]_C16:1		[TG(55:8),TG(54:1)]_C18:0
LPC(18:0),PC(O-18:0),LPC(O-19:0)		PG(20:0),LPG(21:0); PG(20:0),LPG(21:0)
DG(30:1)_C16:0		[TG(52:4)]_C16:0
PC(28:1),PC(P-29:0)		PC(36:3),PC(P-37:2)
[TG(44:0),TG(O-45:0)]_C16:0		DG(36:7)_C16:1
[TG(50:8),TG(49:1)]_C18:1		DG(30:3)_C16:1
[TG(53:10),TG(52:3)]_C18:0		[TG(39:0)]_C20:0
[TG(46:2)]_C18:2		[TG(55:11),TG(54:4)]_C18:2
[TG(54:6)]_C18:1		[TG(54:5)]_C18:1
PC(37:7),PC(P-38:6),PC(36:0),PC(O-37:0)		[TG(50:3)]_C14:0
SM(d16:1/22:0)		[TG(49:8),TG(48:1)]_C18:1
LPG(20:0); LPG(20:0)		[TG(51:9),TG(50:2)]_C16:0
PC(30:1),PC(O-31:1),PC(P-31:0)		[TG(53:9),TG(52:2)]_C18:2
SM(d16:0/18:0)		[TG(54:9),TG(53:2)]_C18:1
[TG(51:8),TG(50:1)]_C16:1		FA(22:1)
PC(34:0),PC(O-35:0)		[TG(53:8),TG(52:1)]_C16:0
PC(40:5)		[TG(55:10),TG(54:3)]_C18:1
Cer(d18:0/17:0)		PC(34:1),PC(O-35:1),PC(P-35:0)
CAR(10:2)		[TG(49:7),TG(48:0)]_C16:0
PC(37:5),PC(O-38:5),PC(P-38:4)		[TG(49:7),TG(48:0)]_C18:0
[TG(48:3)]_C16:1		PC(36:4),PC(O-37:4)
[TG(54:7)]_C18:2		SM(d16:0/22:0)
[TG(50:3)]_C18:3		PC(34:2),PC(O-35:2),PC(P-35:1)
[TG(57:11),TG(56:4)]_C18:2		CE(15:1) NH4
[TG(44:1)]_C16:0		[TG(51:8),TG(50:1)]_C18:1
[TG(54:6)]_C18:3		PC(38:4)
[TG(53:8)]_C18:2		[TG(50:7),TG(49:0)]_C16:0
[TG(46:2)]_C18:1		PC(40:6)
[TG(49:7),TG(48:0)]_C14:0		[TG(51:7),TG(50:0)]_C18:0
[TG(52:5)]_C16:1		[TG(54:5)]_C18:2
PC(35:3),PC(O-36:3),PC(P-36:2)		LPG(19:0),LPG(O-20:0); LPG(19:0),LPG(O-20:0)
SM(d16:1/24:1)		[TG(52:9),TG(51:2)]_C18:1
PC(31:1),PC(O-32:1),PC(P-32:0)		[TG(53:8),TG(52:1)]_C18:0
[TG(51:7)]_C18:1		[TG(53:10),TG(52:3)]_C16:0
[TG(48:2)]_C18:1		PC(38:6)
[TG(55:9),TG(54:2)]_C18:2		[TG(51:9),TG(50:2)]_C14:0
SM(d16:1/18:0)		[TG(52:5)]_C18:2
SM(d16:1/20:0)		[TG(50:3)]_C18:1

[TG(52:9),TG(51:2)]_C16:0		PS(O-29:0)
[TG(48:2)]_C16:1		[TG(53:9),TG(52:2)]_C16:0
[TG(52:10),TG(51:3)]_C18:2		DG(30:2)_C16:0
[TG(46:1)]_C16:1		[TG(51:9),TG(50:2)]_C18:1
PC(30:2),PC(P-31:1)		[TG(50:3)]_C16:1
CAR(14:2)		DG(36:6)_C16:0
[TG(53:9),TG(52:2)]_C16:1		[TG(53:10),TG(52:3)]_C16:1
[TG(56:7)]_C20:4		DG(36:5)_C16:0
[TG(54:5)]_C20:4		[TG(55:11),TG(54:4)]_C18:1
[TG(46:0)]_C14:0		[TG(46:0)]_C16:0
		[TG(55:11),TG(54:4)]_C18:0
		PG(16:0),LPG(17:0),LPG(O-18:0); PG(16:0),LPG(17:0),LPG(O-18:0)
		PC(38:5)

Comparison of Lipid Corona Profiles Between Sexes

Table S7. Comparison of Lipid Corona Profiles Between Sexes

Unique Lipids in Males	Unique Lipds in Females	Shared Lipids
[TG(46:2)]_C16:0	DG(30:2)_C16:1	[TG(54:6)]_C18:2
SM(d18:0/17:0)	DG(36:5)_C16:0	PC(33:2),PC(O-34:2),PC(P-34:1)
[TG(54:5)]_C18:0	[TG(45:2)]_C16:0	[TG(53:7),TG(52:0)]_C16:0
FA(28:6)	PS(25:0)	[TG(57:12),TG(56:5)]_C18:1
[TG(54:11),TG(53:4)]_C18:2	FA(21:0)	PC(35:2),PC(O-36:2),PC(P-36:1)
SM(d18:0/26:1(17Z))	FA(22:0)	[TG(52:4)]_C16:1
[TG(56:12),TG(55:5)]_C18:1	FA(20:0)	[TG(54:5)]_C18:3
PC(39:8),PC(O-40:8),PC(38:1),PC(O-39:1),PC(P-39:0)		[TG(52:4)]_C18:3
[TG(57:12),TG(56:5)]_C16:0		SM(d16:1/16:0)
LPI(20:0)		[TG(51:7),TG(50:0)]_C16:0
[TG(53:7),TG(52:0)]_C20:0		FA(35:0)
[TG(50:9),TG(49:2)]_C16:0		PC(O-38:9),PC(36:2),PC(O-37:2),PC(P-37:1)
[TG(44:1)]_C18:1		[TG(52:8),TG(51:1)]_C16:0
PC(39:4),PC(O-40:4),PC(P-40:3)		[TG(57:11),TG(56:4)]_C18:1
[TG(50:9),TG(49:2)]_C18:1		[TG(48:2)]_C14:0
PC(35:5),PC(O-36:5),PC(P-36:4)		[TG(51:9),TG(50:2)]_C16:1
LPC(16:0),PC(O-16:0),LPC(O-17:0)		[TG(52:4)]_C18:1
[TG(46:0)]_C18:0		[TG(49:8),TG(48:1)]_C14:0
[TG(52:4)]_C20:4		PC(30:0),PC(O-31:0)
[TG(54:10),TG(53:3)]_C16:0		[TG(57:12),TG(56:5)]_C20:4
[TG(48:8),TG(47:1)]_C18:1		[TG(55:9),TG(54:2)]_C18:0
PC(40:1),PC(P-41:0)		SM(d16:1/24:0)
[TG(59:9),TG(58:2)]_C18:1		[TG(54:10),TG(53:3)]_C18:1
[TG(46:1)]_C18:1		[TG(53:10),TG(52:3)]_C18:2
[TG(57:9),TG(56:2)]_C16:0		Cer(d18:1/24:0)
[TG(52:6)]_C18:2		[TG(53:7),TG(52:0)]_C18:0
[TG(54:11),TG(53:4)]_C16:0		[TG(52:5)]_C16:0
PC(36:7),PC(35:0),PC(O-36:0)		[TG(52:5)]_C18:3
[TG(52:6)]_C16:0		[TG(56:6)]_C20:4
[TG(44:2)]_C18:2		[TG(49:8),TG(48:1)]_C16:1
[TG(50:8),TG(49:1)]_C16:1		[TG(49:8),TG(48:1)]_C16:0
SM(d16:0/25:0)		[TG(55:10),TG(54:3)]_C18:0
LPC(22:4)		[TG(53:8),TG(52:1)]_C18:1
[TG(48:7),TG(47:0)]_C16:0		DG(42:11),DG(41:4)_C16:0
PC(39:6),PC(O-40:6),PC(P-40:5)		[TG(55:10),TG(54:3)]_C18:2
PI(38:4)		[TG(55:8),TG(54:1)]_C18:1
[TG(54:8),TG(53:1)]_C18:1		[TG(48:2)]_C16:0
[TG(52:10),TG(51:3)]_C18:1		[TG(55:8),TG(54:1)]_C18:0
[TG(45:0)]_C16:0		DG(36:6)_C16:1
[TG(48:3)]_C18:2		PC(36:3),PC(P-37:2)
SM(d16:1/25:0)		[TG(50:4)]_C16:1
[TG(51:8)]_C18:2		LPC(18:0),PC(O-18:0),LPC(O-19:0)
PC(41:6),PC(O-42:6)		DG(30:1)_C16:0
PC(39:7),PC(P-40:6),PC(38:0),PC(O-39:0)		PC(32:1),PC(O-33:1),PC(P-33:0)
[TG(49:7)]_C16:1		[TG(53:10),TG(52:3)]_C18:0
CE(22:5)H		[TG(39:0)]_C20:0
SM(d16:1/23:0)		[TG(55:11),TG(54:4)]_C18:2
[TG(46:1)]_C16:0		[TG(54:6)]_C18:1
[TG(56:6)]_C18:2		LPG(20:0); LPG(20:0)
SM(d18:2/14:0)		[TG(50:3)]_C14:0
PC(37:6),PC(O-38:6),PC(P-38:5)		[TG(49:8),TG(48:1)]_C18:1
[TG(57:9),TG(56:2)]_C18:2		[TG(53:9),TG(52:2)]_C18:2
[TG(44:0),TG(O-45:0)]_C16:0		[TG(56:7)]_C18:2
[TG(56:11),TG(55:4)]_C18:1		[TG(51:8),TG(50:1)]_C16:1
[TG(50:8),TG(49:1)]_C18:1		SM(d16:0/18:0)
[TG(56:8)]_C20:4		[TG(54:9),TG(53:2)]_C18:1
[TG(50:4)]_C18:1		PC(34:0),PC(O-35:0)
PG(O-35:1),PG(P-35:0); PG(O-35:1),PG(P-35:0)		PC(40:5)
[TG(49:8)]_C18:2		FA(30:0)
[TG(48:3)]_C14:0		[TG(53:8),TG(52:1)]_C16:0
[TG(55:7),TG(54:0)]_C20:0		PC(34:1),PC(O-35:1),PC(P-35:0)
[TG(54:7)]_C20:4		[TG(51:8),TG(50:1)]_C14:0
[TG(61:10),TG(60:3)]_C18:1		SM(d18:1/19:0)
[TG(56:8)]_C22:6		[TG(56:7)]_C22:6
[TG(44:0),TG(O-45:0)]_C18:0		[TG(54:6)]_C18:3
[TG(54:11),TG(53:4)]_C18:1		CE(15:1) NH4
SM(d18:0/15:0)		[TG(52:5)]_C16:1
SM(d18:2/18:1)		PC(35:3),PC(O-36:3),PC(P-36:2)
PC(42:2)		PC(38:4)
SM(d17:1/24:1)		[TG(50:7),TG(49:0)]_C16:0
CE(20:2)Na		[TG(54:5)]_C18:2
PC(32:2),PC(O-33:2),PC(P-33:1)		SM(d16:1/20:1)
PC(44:12),PC(O-44:5)		[TG(51:7)]_C18:1
[TG(50:4)]_C18:3		[TG(53:10),TG(52:3)]_C16:0
[TG(56:6)]_C16:0		[TG(48:2)]_C18:1
[TG(59:10),TG(58:3)]_C18:2		SM(d16:1/20:0)
PC(19:1),LPC(20:1),PC(O-20:1),PC(P-20:0)		[TG(51:9),TG(50:2)]_C14:0
[TG(48:3)]_C16:1		SM(d18:2/24:1)

[TG(54:7)]_C18:2
PC(28:2)
PC(35:6),PC(P-36:5)
SM(d17:1/26:1)
[TG(53:10),TG(52:3)]_C18:3
[TG(48:4)]_C18:2
[TG(55:9),TG(54:2)]_C20:0
[TG(52:9),TG(51:2)]_C18:2
[TG(57:9),TG(56:2)]_C18:0
[TG(57:10),TG(56:3)]_C20:0
[TG(44:0),TG(O-45:0)]_C14:0
PC(O-40:9),PC(38:2),PC(P-39:1)
PC(42:10),PC(41:3),PC(O-42:3),PC(P-42:2)
[TG(44:2)]_C16:0
[TG(57:9),TG(56:2)]_C18:1
PC(42:9),PC(41:2),PC(O-42:2),PC(P-42:1)
SM(d16:0/16:0)
[TG(37:0)]_C18:0
SM(d18:0/24:0)
[TG(46:1)]_C16:1
[TG(48:3)]_C18:1
CAR(14:2)
[TG(46:2)]_C14:0
[TG(46:0)]_C14:0
PC(40:7),PC(39:0),PC(O-40:0)
PC(42:11),PC(41:4),PC(O-42:4)
[TG(46:3)]_C18:2
[TG(58:8)]_C22:6
[TG(46:1)]_C14:0
[TG(59:11),TG(58:4)]_C18:2
[TG(54:5)]_C22:5
CE(20:0) NH4
[TG(48:3)]_C16:0
[TG(54:7),TG(53:0)]_C18:0
[TG(52:8),TG(51:1)]_C18:1
[TG(57:11),TG(56:4)]_C18:0
[TG(57:9),TG(56:2)]_C20:0
[TG(50:4)]_C14:0
[TG(42:0)]_C16:0
[TG(52:5)]_C20:4
[TG(46:2)]_C16:1
[TG(54:9),TG(53:2)]_C18:2
[TG(52:7),TG(51:0)]_C16:0
[TG(49:8),TG(48:1)]_C18:0
PC(42:3)
[TG(54:6)]_C16:0
[TG(44:1)]_C16:1
[TG(56:10),TG(55:3)]_C18:1
PC(39:5),PC(O-40:5),PC(P-40:4)
[TG(55:11),TG(54:4)]_C20:4
[TG(57:12),TG(56:5)]_C18:0
[TG(57:11),TG(56:4)]_C20:0
[TG(59:10),TG(58:3)]_C18:1
LPC(20:2),PC(O-20:2)
[TG(49:7)]_C18:1
PC(38:8),PC(37:1),PC(O-38:1),PC(P-38:0)
[TG(56:6)]_C18:0
[TG(52:10),TG(51:3)]_C16:0
[TG(54:7)]_C18:3
PC(38:7),PC(37:0),PC(O-38:0)
PC(42:4)
[TG(52:5)]_C18:1
SM(d18:2/21:0)
[TG(51:4)]_C18:2
[TG(50:4)]_C16:0
[TG(48:7),TG(47:0)]_C14:0
CE(22:6)Na
[TG(50:8),TG(49:1)]_C16:0
[TG(55:8),TG(54:1)]_C16:0
PC(40:2)
[TG(51:6)]_C16:0
[TG(42:0)]_C14:0
PC(40:3)
PC(33:0),PC(O-34:0)
[TG(57:12),TG(56:5)]_C18:2
[TG(57:10),TG(56:3)]_C18:2
[TG(46:2)]_C18:2
SM(d18:1/24:1(15Z))
[TG(56:8),TG(55:1)]_C16:0
SM(d18:1/26:1(17Z))
PC(41:5),PC(P-42:4)
[TG(50:9),TG(49:2)]_C18:2
PC(37:4),PC(O-38:4),PC(P-38:3)
[TG(52:5)]_C18:2
[TG(50:3)]_C18:1
[TG(52:10),TG(51:3)]_C18:2
SM(d16:0/24:0)
[TG(54:5)]_C20:4
[TG(53:10),TG(52:3)]_C16:1
[TG(46:0)]_C16:0
[TG(55:11),TG(54:4)]_C18:0
FA(37:0)
PC(38:5)
PC(38:3)
SM(d16:1/17:0)
[TG(53:9),TG(52:2)]_C18:0
[TG(50:3)]_C18:2
[TG(49:6)]_C16:0
[TG(53:7)]_C18:1
[TG(51:8),TG(50:1)]_C18:0
PC(O-38:8),PC(36:1),PC(O-37:1),PC(P-37:0)
[TG(54:5)]_C16:0
[TG(55:11),TG(54:4)]_C16:0
[TG(55:9),TG(54:2)]_C16:0
DG(36:8),DG(35:1)_C16:1
PC(32:0),PC(O-33:0)
[TG(53:9),TG(52:2)]_C18:1
[TG(55:9),TG(54:2)]_C18:1
DG(36:7),DG(35:0)_C16:0
SM(d16:1/18:1)
PC(36:5)
[TG(51:8),TG(50:1)]_C16:0
SM(d18:2/22:1)
[TG(54:6)]_C20:4
PC(40:10),PC(39:3),PC(O-40:3),PC(P-40:2)
[TG(51:9),TG(50:2)]_C18:2
[TG(50:4)]_C18:2
[TG(48:2)]_C18:2
DG(41:5)_C16:0
[TG(52:4)]_C18:2
SM(d18:1/17:0)
[TG(50:3)]_C16:0
[TG(53:10),TG(52:3)]_C18:1
Cer(d14:2(4E,6E)/16:0)
[TG(55:10),TG(54:3)]_C16:0
PC(29:1),PC(O-30:1),PC(P-30:0)
PC(28:0),PC(O-29:0)
SM(d16:0/20:0)
PC(40:4)
[TG(52:4)]_C16:0
PC(35:4),PC(O-36:4),PC(P-36:3)
DG(36:7)_C16:1
PC(28:1),PC(P-29:0)
DG(30:3)_C16:1
PC(38:9),PC(37:2),PC(O-38:2),PC(P-38:1)
SM(d16:1/22:1)
PC(36:8),PC(35:1),PC(O-36:1),PC(P-36:0)
[TG(56:6)]_C22:5
PC(37:7),PC(P-38:6),PC(36:0),PC(O-37:0)
SM(d16:1/22:0)
[TG(54:5)]_C18:1
PC(30:1),PC(O-31:1),PC(P-31:0)
CAR(20:0)
[TG(51:9),TG(50:2)]_C16:0
FA(22:7)
PC(33:1),PC(O-34:1),PC(P-34:0)
SM(d16:0/23:0)
Cer(d18:0/17:0)
[TG(55:10),TG(54:3)]_C18:1
[TG(54:10),TG(53:3)]_C18:2
PC(37:5),PC(O-38:5),PC(P-38:4)
[TG(49:7),TG(48:0)]_C16:0
DG(41:6)_C16:1
[TG(49:7),TG(48:0)]_C18:0
PC(36:4),PC(O-37:4)
PC(31:0),PC(O-32:0)
SM(d16:0/22:0)
PC(34:2),PC(O-35:2),PC(P-35:1)
[TG(44:1)]_C16:0
SM(d16:1/24:1)
PC(31:1),PC(O-32:1),PC(P-32:0)
[TG(51:8),TG(50:1)]_C18:1
PC(40:6)
[TG(51:7),TG(50:0)]_C18:0

[TG(52:6)]_C18:3		[TG(52:9),TG(51:2)]_C18:1
FA(22:1)		PC(40:8),PC(39:1),PC(O-40:1),PC(P-40:0)
CAR(10:2)		[TG(53:8),TG(52:1)]_C18:0
[TG(54:9),TG(53:2)]_C16:0		PC(38:6)
[TG(42:1)]_C18:1		[TG(55:9),TG(54:2)]_C18:2
LPC(18:1),PC(O-18:1),PC(P-18:0)		SM(d16:1/18:0)
[TG(44:1)]_C14:0		[TG(52:9),TG(51:2)]_C16:0
[TG(50:5)]_C18:2		PC(37:3),PC(O-38:3),PC(P-38:2)
SM(d18:1/25:0)		[TG(48:2)]_C16:1
[TG(50:3)]_C18:3		PS(O-29:0)
[TG(57:11),TG(56:4)]_C18:2		[TG(53:9),TG(52:2)]_C16:0
[TG(48:8),TG(47:1)]_C16:0		DG(30:2)_C16:0
[TG(55:8),TG(54:1)]_C20:0		PC(30:2),PC(P-31:1)
[TG(53:8)]_C18:2		[TG(51:9),TG(50:2)]_C18:1
[TG(46:2)]_C18:1		[TG(50:3)]_C16:1
[TG(49:7),TG(48:0)]_C14:0		[TG(53:9),TG(52:2)]_C16:1
[TG(47:6)]_C16:0		DG(36:6)_C16:0
[TG(48:3)]_C18:3		[TG(56:7)]_C20:4
[TG(56:7)]_C22:5		[TG(57:10),TG(56:3)]_C18:1
[TG(51:9),TG(50:2)]_C18:0		[TG(55:11),TG(54:4)]_C18:1
PC(42:8),PC(41:1),PC(O-42:1),PC(P-42:0)		PG(16:0),LPG(17:0),LPG(O-18:0); PG(16:0),LPG(17:0),LPG(O-18:0)
[TG(51:7),TG(50:0)]_C14:0		
SM(d18:0/24:1)		
[TG(55:11),TG(54:4)]_C18:3		
[TG(50:7),TG(49:0)]_C18:0		
PC(16:0),PC(O-17:0),LPC(O-18:0)		
PC(33:3),PC(O-34:3),PC(P-34:2)		
CE(20:1) NH4		
[TG(57:8),TG(56:1)]_C18:1		
CE(16:0)K		
PC(40:9),PC(39:2),PC(O-40:2),PC(P-40:1)		
[TG(48:8),TG(47:1)]_C14:0		
[TG(40:0)]_C16:0		
[TG(52:8),TG(51:1)]_C18:0		
[TG(50:4)]_C20:4		

Comparison of Lipid Corona Profiles Between Sexes

Table S7. Comparison of Lipid Corona Profiles Between Sexes

Unique Lipids in Males	Unique Lipds in Females	Shared Lipids
[TG(44:2)]_C18:1	[TG(59:13),TG(58:6)]_C18:1	PC(44:10),PC(O-44:3)
[TG(50:9),TG(49:2)]_C16:1	[TG(38:0)]_C18:0	[TG(54:5)]_C18:0
[TG(59:9),TG(58:2)]_C18:1	PC(42:1)	[TG(54:6)]_C18:2
[TG(59:9),TG(58:2)]_C18:2	[TG(57:12),TG(56:5)]_C22:5	[TG(57:12),TG(56:5)]_C18:1
[TG(52:6)]_C16:0	[TG(57:11),TG(56:4)]_C16:0	PC(35:2),PC(O-36:2),PC(P-36:1)
[TG(57:8),TG(56:1)]_C16:0	[TG(58:7)]_C18:1	[TG(56:12),TG(55:5)]_C18:1
[TG(48:7),TG(47:0)]_C16:0	FA(22:7)	PC(39:8),PC(O-40:8),PC(38:1),PC(O-39:1),PC(P-39:0)
[TG(45:1)]_C16:0	FA(22:1)	DG(30:2)_C16:1
[TG(45:0)]_C16:0	PC(36:6)	LPI(20:0)
[TG(49:7)]_C16:1	FA(19:2)	[TG(52:4)]_C16:1
CE(20:5) NH4	PI(36:1),PI(O-37:1),PI(P-37:0)	[TG(57:11),TG(56:4)]_C18:1
CE(22:3)H	PC(29:2),PC(P-30:1)	LPC(16:0),PC(O-16:0),LPC(O-17:0)
[TG(57:9),TG(56:2)]_C18:2	[TG(38:0)]_C14:0	[TG(48:2)]_C14:0
PG(O-35:1),PG(P-35:0); PG(O-35:1),PG(P-35:0)	DG(32:5)_C18:1	[TG(52:4)]_C20:4
CE(14:0) NH4	PS(38:4)	[TG(52:4)]_C18:1
CE(20:0)H	PE(38:6)	[TG(49:8),TG(48:1)]_C14:0
CE(18:0)K	[TG(54:12),TG(53:5)]_C18:2	[TG(57:12),TG(56:5)]_C20:4
[TG(50:9),TG(49:2)]_C14:0	[TG(58:8),TG(57:1)]_C18:1	PC(40:1),PC(P-41:0)
[TG(59:10),TG(58:3)]_C18:2	SM(d18:2/15:0)	[TG(55:9),TG(54:2)]_C18:0
CE(16:1) NH4	PC(30:3)	SM(d16:1/24:0)
[TG(50:8),TG(49:1)]_C14:0	[TG(56:6)]_C18:0	[TG(53:10),TG(52:3)]_C18:2
[TG(44:0),TG(O-45:0)]_C14:0	PE(34:2),PE(O-35:2),PE(P-35:1)	[TG(54:11),TG(53:4)]_C16:0
[TG(52:4)]_C14:0	FA(21:0)	PC(36:7),PC(35:0),PC(O-36:0)
[TG(46:1)]_C16:1	PC(24:0)	SM(d16:0/25:0)
[TG(46:2)]_C14:0	PE(36:3),PE(P-37:2)	Cer(d18:1/24:0)
[TG(42:1)]_C16:0		[TG(56:6)]_C20:4
[TG(59:11),TG(58:4)]_C18:2		[TG(49:8),TG(48:1)]_C16:1
[TG(54:7),TG(53:0)]_C18:0		[TG(56:11),TG(55:4)]_C18:2
[TG(52:9),TG(51:2)]_C16:1		[TG(58:9)]_C22:6
DG(O-38:8),DG(36:1)_C16:1		PC(39:6),PC(O-40:6),PC(P-40:5)
CE(20:4) NH4		PI(38:4)
[TG(58:9)]_C20:4		[TG(54:8),TG(53:1)]_C18:1
CE(22:2) NH4		[TG(52:10),TG(51:3)]_C18:1
[TG(44:1)]_C16:1		DG(42:11),DG(41:4)_C16:0
[TG(53:9),TG(52:2)]_C20:0		[TG(55:7)]_C18:1
[TG(55:7),TG(54:0)]_C16:0		[TG(55:10),TG(54:3)]_C18:2
[TG(52:9),TG(51:2)]_C18:0		[TG(48:3)]_C18:2
[TG(57:8),TG(56:1)]_C18:0		[TG(55:8),TG(54:1)]_C18:1
[TG(48:7),TG(47:0)]_C14:0		[TG(48:2)]_C16:0
CE(22:6)Na		[TG(56:12),TG(55:5)]_C18:2
[TG(51:6)]_C16:0		DG(36:6)_C16:1
[TG(58:14),TG(57:7),TG(56:0)]_C16:0		SM(d16:1/23:0)
[TG(55:10),TG(54:3)]_C20:0		[TG(46:1)]_C16:0
[TG(48:4)]_C18:3		[TG(56:6)]_C18:2
[TG(48:8),TG(47:1)]_C16:0		SM(d18:2/14:0)
FA(31:0)		[TG(50:4)]_C16:1
[TG(54:7)]_C18:1		LPC(18:0),PC(O-18:0),LPC(O-19:0)
[TG(48:8),TG(47:1)]_C14:0		[TG(44:0),TG(O-45:0)]_C16:0
CE(19:0)Na		[TG(56:11),TG(55:4)]_C18:1
[TG(46:2)]_C16:0		[TG(54:9),TG(53:2)]_C18:0
[TG(47:2)]_C18:2		[TG(38:1)]_C18:1
[TG(41:0)]_C16:0		PC(32:1),PC(O-33:1),PC(P-33:0)
[TG(48:8),TG(47:1)]_C18:1		[TG(53:10),TG(52:3)]_C18:0
[TG(57:9),TG(56:2)]_C16:0		[TG(39:0)]_C20:0
CE(20:3) NH4		[TG(54:6)]_C18:1
[TG(44:2)]_C18:2		[TG(50:3)]_C14:0
CE(22:1) NH4		[TG(50:4)]_C18:1
DG(39:7)_C18:1		[TG(49:8),TG(48:1)]_C18:1
CE(18:3)Na		[TG(49:8)]_C18:2
CE(15:0) NH4		PC(42:6)
[TG(46:3)]_C18:1		[TG(56:8)]_C22:6
[TG(48:3)]_C14:0		PC(34:0),PC(O-35:0)
[TG(61:10),TG(60:3)]_C18:1		[TG(54:11),TG(53:4)]_C18:1
[TG(44:0),TG(O-45:0)]_C18:0		SM(d18:0/15:0)
PC(44:0)		SM(d18:2/18:1)
FA(6:0)		[TG(53:8),TG(52:1)]_C16:0
DG(37:6)_C16:0		LPC(20:4)
[TG(62:16),TG(61:9),TG(60:2)]_C18:1		[TG(56:7),TG(55:0)]_C16:0
[TG(57:8)]_C18:2		PC(42:2)
[TG(46:3)]_C18:2		PC(34:1),PC(O-35:1),PC(P-35:0)
CE(18:1)K		SM(d17:1/24:1)
LPG(18:0); LPG(18:0)		CE(20:2)Na
[TG(54:5)]_C16:1		[TG(48:3)]_C16:1
PE(O-38:8),PE(36:1),PE(O-37:1),PE(P-37:0)		[TG(52:6)]_C16:1
[TG(46:2)]_C16:1		PC(32:3),PC(P-33:2)
[TG(52:4)]_C18:0		PC(35:6),PC(P-36:5)
CAR(18:3)		SM(d17:1/26:1)
[TG(59:10),TG(58:3)]_C18:1		[TG(53:10),TG(52:3)]_C18:3

CE(22:3) NH4		[TG(54:6)]_C18:3
PI(34:1),PI(O-35:1),PI(P-35:0)		[TG(55:9),TG(54:2)]_C20:0
PG(20:0),LPG(21:0); PG(20:0),LPG(21:0)		[TG(52:9),TG(51:2)]_C18:2
[TG(48:8),TG(47:1)]_C16:1		[TG(57:9),TG(56:2)]_C18:0
[TG(51:6)]_C18:0		[TG(50:7),TG(49:0)]_C16:0
[TG(46:1)]_C18:0		LPG(19:0),LPG(O-20:0); LPG(19:0),LPG(O-20:0)
PG(32:0),PG(O-33:0); PG(32:0),PG(O-33:0)		PC(O-40:9),PC(38:2),PC(P-39:1)
CE(22:4)Na		[TG(51:7)]_C18:1
[TG(46:3)]_C16:1		PC(42:10),PC(41:3),PC(O-42:3),PC(P-42:2)
[TG(42:0)]_C18:0		[TG(44:2)]_C16:0
[TG(44:1)]_C14:0		SM(d16:1/20:0)
[TG(50:5)]_C20:4		[TG(51:9),TG(50:2)]_C14:0
[TG(47:6)]_C16:0		SM(d16:0/16:0)
[TG(48:3)]_C18:3		PC(37:4),PC(O-38:4),PC(P-38:3)
[TG(57:8),TG(56:1)]_C18:1		[TG(52:5)]_C18:2
[TG(50:4)]_C20:4		[TG(37:0)]_C18:0
		[TG(50:3)]_C18:1
		[TG(52:10),TG(51:3)]_C18:2
		SM(d16:0/24:0)
		[TG(48:3)]_C18:1
		[TG(58:8)]_C22:5
		[TG(46:0)]_C14:0
		DG(36:5)_C16:0
		[TG(58:7)]_C22:5
		PC(42:11),PC(41:4),PC(O-42:4)
		LPC(18:2),LPC(P-19:1)
		[TG(46:1)]_C14:0
		SM(d16:1/17:0)
		[TG(56:9),TG(55:2)]_C18:1
		[TG(54:5)]_C22:5
		[TG(53:8),TG(52:1)]_C16:1
		[TG(49:6)]_C16:0
		[TG(51:8),TG(50:1)]_C18:0
		[TG(52:8),TG(51:1)]_C18:1
		[TG(57:9),TG(56:2)]_C20:0
		[TG(57:11),TG(56:4)]_C18:0
		[TG(50:4)]_C14:0
		[TG(42:0)]_C16:0
		PC(O-38:8),PC(36:1),PC(O-37:1),PC(P-37:0)
		[TG(55:11),TG(54:4)]_C16:0
		[TG(55:9),TG(54:2)]_C16:0
		DG(36:8),DG(35:1)_C16:1
		[TG(52:5)]_C20:4
		[TG(53:9),TG(52:2)]_C18:1
		[TG(52:7),TG(51:0)]_C16:0
		[TG(55:9),TG(54:2)]_C18:1
		PC(36:5)
		PC(42:3)
		[TG(53:8),TG(52:1)]_C20:0
		[TG(54:6)]_C16:0
		[TG(56:10),TG(55:3)]_C18:1
		[TG(51:8),TG(50:1)]_C16:0
		[TG(55:11),TG(54:4)]_C20:4
		[TG(57:12),TG(56:5)]_C18:0
		PC(40:10),PC(39:3),PC(O-40:3),PC(P-40:2)
		[TG(49:7)]_C18:1
		[TG(50:4)]_C18:2
		PC(38:8),PC(37:1),PC(O-38:1),PC(P-38:0)
		DG(41:5)_C16:0
		[TG(52:4)]_C18:2
		SM(d18:1/17:0)
		[TG(50:3)]_C16:0
		[TG(53:10),TG(52:3)]_C18:1
		PC(38:7),PC(37:0),PC(O-38:0)
		DG(36:8),DG(35:1)_C18:1
		[TG(52:5)]_C18:1
		PC(28:0),PC(O-29:0)
		PC(34:6)
		SM(d16:0/20:0)
		PC(40:4)
		PC(35:4),PC(O-36:4),PC(P-36:3)
		[TG(50:8),TG(49:1)]_C16:0
		DG(36:7)_C16:1
		DG(39:8),DG(O-40:8),DG(38:1)_C18:1
		PC(41:7),PC(P-42:6),PC(40:0),PC(O-41:0)
		[TG(42:0)]_C14:0
		PC(40:3)
		PC(33:0),PC(O-34:0)
		DG(30:3)_C16:1
		[TG(57:12),TG(56:5)]_C18:2
		PC(34:3),PC(P-35:2)
		PC(36:8),PC(35:1),PC(O-36:1),PC(P-36:0)

		[TG(56:6)]_C22:5
		SM(d18:1/24:1(15Z))
		[TG(54:5)]_C18:1
		SM(d16:1/22:0)
		PC(30:1),PC(O-31:1),PC(P-31:0)
		CAR(20:0)
		[TG(51:9),TG(50:2)]_C16:0
		PC(33:1),PC(O-34:1),PC(P-34:0)
		SM(d16:0/23:0)
		[TG(50:9),TG(49:2)]_C18:2
		Cer(d18:0/17:0)
		[TG(55:10),TG(54:3)]_C18:1
		[TG(54:10),TG(53:3)]_C18:2
		PC(37:5),PC(O-38:5),PC(P-38:4)
		[TG(49:7),TG(48:0)]_C16:0
		[TG(49:7),TG(48:0)]_C18:0
		[TG(50:5)]_C18:2
		PC(31:0),PC(O-32:0)
		PC(34:2),PC(O-35:2),PC(P-35:1)
		[TG(55:8),TG(54:1)]_C20:0
		[TG(44:1)]_C16:0
		PC(42:0)
		[TG(53:8)]_C18:2
		[TG(46:2)]_C18:1
		[TG(56:7)]_C22:5
		PC(31:1),PC(O-32:1),PC(P-32:0)
		[TG(51:8),TG(50:1)]_C18:1
		PC(40:6)
		[TG(51:7),TG(50:0)]_C18:0
		[TG(52:9),TG(51:2)]_C18:1
		[TG(51:7),TG(50:0)]_C14:0
		[TG(55:11),TG(54:4)]_C18:3
		PC(38:6)
		[TG(55:9),TG(54:2)]_C18:2
		SM(d16:1/18:0)
		CE(20:1) NH4
		[TG(52:9),TG(51:2)]_C16:0
		[TG(48:2)]_C16:1
		[TG(53:9),TG(52:2)]_C16:0
		1-O-tricosanoyl-Cer(d18:1/16:0)
		PC(43:6)
		PC(30:2),PC(P-31:1)
		[TG(50:3)]_C16:1
		DG(36:6)_C16:0
		[TG(56:7)]_C20:4
		[TG(57:10),TG(56:3)]_C18:1
		[TG(40:0)]_C16:0
		[TG(52:8),TG(51:1)]_C18:0
		SM(d18:1/12:0)
		PE(38:4)
		SM(d18:0/17:0)
		PI(38:3)
		CE(18:1) NH4
		PI(36:2),PI(O-37:2),PI(P-37:1)
		Cer(d18:1/22:0)
		PC(33:2),PC(O-34:2),PC(P-34:1)
		SM(d18:0/26:1(17Z))
		[TG(54:11),TG(53:4)]_C18:2
		[TG(53:7),TG(52:0)]_C16:0
		DG(39:8),DG(O-40:8)_C18:2
		[TG(57:12),TG(56:5)]_C16:0
		[TG(52:4)]_C18:3
		[TG(53:7),TG(52:0)]_C20:0
		[TG(54:5)]_C18:3
		SM(d16:1/16:0)
		[TG(44:1)]_C18:1
		[TG(50:9),TG(49:2)]_C16:0
		PC(39:4),PC(O-40:4),PC(P-40:3)
		[TG(50:9),TG(49:2)]_C18:1
		[TG(51:7),TG(50:0)]_C16:0
		PC(O-38:9),PC(36:2),PC(O-37:2),PC(P-37:1)
		FA(35:0)
		PC(31:2),PC(O-32:2),PC(P-32:1)
		PC(35:5),PC(O-36:5),PC(P-36:4)
		[TG(52:8),TG(51:1)]_C16:0
		[TG(46:0)]_C18:0
		[TG(51:9),TG(50:2)]_C16:1
		[TG(54:10),TG(53:3)]_C16:0
		PC(42:5)
		PC(30:0),PC(O-31:0)
		PC(43:4),PC(O-44:4)
		[TG(46:1)]_C18:1

		[TG(54:10),TG(53:3)]_C18:1
		[TG(52:6)]_C18:2
		[TG(53:7),TG(52:0)]_C18:0
		Cer(d18:1/23:0)
		[TG(52:5)]_C18:3
		[TG(52:5)]_C16:0
		LPC(22:4)
		[TG(49:8),TG(48:1)]_C16:0
		[TG(55:10),TG(54:3)]_C18:0
		CAR(14:1)
		[TG(53:8),TG(52:1)]_C18:1
		SM(d16:1/25:0)
		[TG(51:8)]_C18:2
		PC(41:6),PC(O-42:6)
		PC(39:7),PC(P-40:6),PC(38:0),PC(O-39:0)
		[TG(55:8),TG(54:1)]_C18:0
		CE(22:5)H
		[TG(56:8)]_C18:2
		PC(36:3),PC(P-37:2)
		CE(22:5) NH4
		DG(30:1)_C16:0
		PC(37:6),PC(O-38:6),PC(P-38:5)
		Cer(d18:1/24:1(15Z))
		SM(d17:0/27:0)
		[TG(50:8),TG(49:1)]_C18:1
		[TG(56:8)]_C20:4
		[TG(55:11),TG(54:4)]_C18:2
		[TG(57:8),TG(56:1)]_C20:0
		LPG(20:0); LPG(20:0)
		[TG(53:9),TG(52:2)]_C18:2
		[TG(52:7),TG(51:0)]_C18:0
		[TG(55:7),TG(54:0)]_C20:0
		[TG(56:7)]_C18:2
		SM(d16:0/18:0)
		[TG(51:8),TG(50:1)]_C16:1
		[TG(54:9),TG(53:2)]_C18:1
		[TG(54:7)]_C20:4
		PC(40:5)
		[TG(57:10),TG(56:3)]_C18:0
		PC(32:2),PC(O-33:2),PC(P-33:1)
		[TG(51:8),TG(50:1)]_C14:0
		SM(d18:1/19:0)
		PC(44:12),PC(O-44:5)
		[TG(56:7)]_C22:6
		[TG(50:4)]_C18:3
		[TG(56:6)]_C16:0
		PC(19:1),LPC(20:1),PC(O-20:1),PC(P-20:0)
		[TG(54:7)]_C18:2
		PC(28:2)
		[TG(54:8),TG(53:1)]_C18:0
		CE(15:1) NH4
		[TG(52:5)]_C16:1
		PC(35:3),PC(O-36:3),PC(P-36:2)
		[TG(48:4)]_C18:2
		PC(38:4)
		[TG(57:10),TG(56:3)]_C20:0
		[TG(54:5)]_C18:2
		SM(d16:1/20:1)
		CE(16:3)Na
		[TG(53:10),TG(52:3)]_C16:0
		[TG(48:2)]_C18:1
		FA(24:4)
		SM(d18:2/24:1)
		[TG(57:9),TG(56:2)]_C18:1
		CE(22:6) NH4
		PC(42:9),PC(41:2),PC(O-42:2),PC(P-42:1)
		SM(d18:0/24:0)
		CAR(14:2)
		[TG(54:5)]_C20:4
		[TG(53:10),TG(52:3)]_C16:1
		PC(40:7),PC(39:0),PC(O-40:0)
		[TG(46:0)]_C16:0
		[TG(55:11),TG(54:4)]_C18:0
		DG(O-40:9),DG(38:2)_C18:2
		PC(38:5)
		PC(38:3)
		[TG(58:8)]_C22:6
		[TG(53:9),TG(52:2)]_C18:0
		CE(20:0) NH4
		[TG(48:3)]_C16:0
		[TG(50:3)]_C18:2
		[TG(53:7)]_C18:1

		[TG(54:5)]_C16:0
		PC(29:0),PC(O-30:0)
		PC(32:0),PC(O-33:0)
		[TG(54:9),TG(53:2)]_C18:2
		[TG(49:8),TG(48:1)]_C18:0
		SM(d16:1/18:1)
		DG(36:7),DG(35:0)_C16:0
		PC(39:5),PC(O-40:5),PC(P-40:4)
		[TG(57:11),TG(56:4)]_C20:0
		LPC(20:2),PC(O-20:2)
		SM(d18:2/22:1)
		[TG(54:6)]_C20:4
		[TG(51:9),TG(50:2)]_C18:2
		[TG(48:2)]_C18:2
		[TG(54:8),TG(53:1)]_C16:0
		[TG(52:10),TG(51:3)]_C16:0
		[TG(54:7)]_C18:3
		PC(42:4)
		[TG(55:10),TG(54:3)]_C16:0
		Cer(d14:2(4E,6E)/16:0)
		PC(29:1),PC(O-30:1),PC(P-30:0)
		SM(d18:2/21:0)
		[TG(51:4)]_C18:2
		[TG(52:4)]_C16:0
		[TG(50:4)]_C16:0
		[TG(49:3)]_C18:2
		PC(40:2)
		[TG(55:8),TG(54:1)]_C16:0
		PC(28:1),PC(P-29:0)
		PC(38:9),PC(37:2),PC(O-38:2),PC(P-38:1)
		SM(d16:1/22:1)
		[TG(46:2)]_C18:2
		[TG(57:10),TG(56:3)]_C18:2
		PC(37:7),PC(P-38:6),PC(36:0),PC(O-37:0)
		[TG(56:8),TG(55:1)]_C16:0
		SM(d18:1/26:1(17Z))
		PC(41:5),PC(P-42:4)
		PC(42:7),PC(41:0),PC(O-42:0)
		CE(18:2) NH4
		[TG(52:6)]_C18:3
		PE(O-38:9),PE(36:2),PE(O-37:2),PE(P-37:1)
		CE(19:0)H
		[TG(54:9),TG(53:2)]_C16:0
		[TG(42:1)]_C18:1
		LPC(18:1),PC(O-18:1),PC(P-18:0)
		DG(41:6)_C16:1
		PC(36:4),PC(O-37:4)
		SM(d18:1/25:0)
		[TG(50:3)]_C18:3
		SM(d16:0/22:0)
		[TG(57:11),TG(56:4)]_C18:2
		[TG(53:10),TG(52:3)]_C20:0
		[TG(49:7),TG(48:0)]_C14:0
		SM(d16:1/24:1)
		[TG(51:9),TG(50:2)]_C18:0
		PC(42:8),PC(41:1),PC(O-42:1),PC(P-42:0)
		SM(d18:0/24:1)
		PC(40:8),PC(39:1),PC(O-40:1),PC(P-40:0)
		[TG(53:8),TG(52:1)]_C18:0
		[TG(50:7),TG(49:0)]_C18:0
		PC(16:0),PC(O-17:0),LPC(O-18:0)
		PC(33:3),PC(O-34:3),PC(P-34:2)
		PC(37:3),PC(O-38:3),PC(P-38:2)
		PS(O-29:0)
		CE(16:0)K
		DG(30:2)_C16:0
		[TG(51:9),TG(50:2)]_C18:1
		PC(40:9),PC(39:2),PC(O-40:2),PC(P-40:1)
		CE(20:2)K
		[TG(53:9),TG(52:2)]_C16:1
		[TG(55:11),TG(54:4)]_C18:1
		PG(16:0),LPG(17:0),LPG(O-18:0); PG(16:0),LPG(17:0),LPG(O-18:0)

Comparison of Lipid Corona Profiles Between Sexes

Table S7. Comparison of Lipid Corona Profiles Between Sexes

Unique Lipids in Males	Unique Lipds in Females	Shared Lipids	Male Average of Shared Lipids	Female Average of Shared Lipids	p-value of Shared Lipids
CE(20:5)Na	[TG(38:0)]_C18:0	[TG(46:0)]_C14:0	6133.424438	3128.910883	1.60452E-06
[TG(44:2)]_C18:1	[TG(55:7)]_C18:1	[TG(44:0),TG(O-45:0)]_C16:0	8434.620634	3657.326928	2.83528E-06
DG(30:2)_C16:1	PC(42:1)	[TG(46:1)]_C16:0	8222.172603	3492.005573	1.51969E-05
[TG(50:9),TG(49:2)]_C16:1	[TG(38:1)]_C18:1	[TG(46:1)]_C18:1	7110.968481	3033.601551	2.74451E-05
[TG(59:9),TG(58:2)]_C18:1	CE(20:3)Na	[TG(46:1)]_C14:0	4150.412289	2018.998803	4.40355E-05
[TG(59:9),TG(58:2)]_C18:2	PE(36:4),PE(O-37:4)	[TG(49:7),TG(48:0)]_C14:0	3624.636253	2499.109503	5.06245E-05
[TG(57:8),TG(56:1)]_C16:0	[TG(56:9),TG(55:2)]_C18:1	[TG(49:8),TG(48:1)]_C18:1	26659.61368	11600.33611	5.38666E-05
[TG(56:11),TG(55:4)]_C18:2	Cer(d18:1/16:0)	[TG(51:7),TG(50:0)]_C18:0	10532.23675	7986.833164	5.57944E-05
[TG(45:0)]_C16:0	[TG(54:7),TG(53:0)]_C18:0	[TG(49:8),TG(48:1)]_C14:0	14342.68504	6872.161128	6.07004E-05
DG(42:11),DG(41:4)_C16:0	[TG(58:7)]_C18:1	[TG(49:8),TG(48:1)]_C16:0	35817.03874	16767.91712	7.09776E-05
LPC(15:1),LPC(O-16:1),LPC(P-16:0)	PS(P-37:0)	[TG(48:2)]_C16:0	17391.6172	8003.359857	9.30764E-05
DG(36:6)_C16:1	PE(34:1),PE(O-35:1),PE(P-35:0)	[TG(49:7),TG(48:0)]_C16:0	36535.25848	21995.24092	0.000112905
CE(20:5) NH4	PE(38:5)	[TG(46:2)]_C18:2	3929.084297	1977.964139	0.0001224
CE(22:3)H	PC(29:2),PC(P-30:1)	[TG(46:0)]_C16:0	18346.20938	8827.238638	0.000158587
[TG(57:9),TG(56:2)]_C18:2	[TG(38:0)]_C14:0	[TG(48:3)]_C18:1	3237.368246	2125.944809	0.000159837
PG(O-35:1),PG(P-35:0); PG(O-35:1),PG(P-35:0)	PE(38:4)	[TG(46:1)]_C16:1	3114.448226	1950.321478	0.000172737
CE(14:0) NH4	PS(38:4)	LPC(18:0),PC(O-18:0),LPC(O-19:0)	133033.3145	78410.81941	0.000195595
DG(37:7)_C16:1	PE(38:6)	[TG(52:5)]_C20:4	2404.08417	1730.884127	0.000228837
PC(19:0),LPC(20:0),PC(O-20:0)	[TG(58:8),TG(57:1)]_C18:1	[TG(51:7),TG(50:0)]_C16:0	25281.58979	18328.46594	0.000231366
[TG(59:10),TG(58:3)]_C18:2	PC(44:0)	[TG(50:8),TG(49:1)]_C18:1	4365.200307	2727.083533	0.000247079
[TG(49:3)]_C16:0	[TG(55:11),TG(54:4)]_C16:1	[TG(50:8),TG(49:1)]_C16:0	4857.844357	3116.370889	0.000292863
[TG(52:5)]_C22:5	PE(O-38:8),PE(36:1),PE(O-37:1),PE(P-37:0)	[TG(50:9),TG(49:2)]_C18:2	2622.492188	1972.452145	0.000386694
LPG(19:0),LPG(O-20:0); LPG(19:0),LPG(O-20:0)	DG(40:9),DG(39:2)_C18:2	[TG(48:2)]_C14:0	8496.180574	4462.646971	0.000432145
[TG(50:8),TG(49:1)]_C14:0	FA(18:0)	[TG(48:3)]_C18:2	5331.408376	2947.611527	0.000482771
[TG(60:15),TG(59:8),TG(58:1)]_C18:1	CE(20:1)H	[TG(48:3)]_C16:0	3103.004205	1871.990802	0.000488539
[TG(57:11),TG(56:4)]_C16:0	PE(34:2),PE(O-35:2),PE(P-35:1)	[TG(44:0),TG(O-45:0)]_C14:0	3472.872269	1436.752108	0.000539439
[TG(52:4)]_C14:0	PE(40:6),PE(dO-40:0)	[TG(49:7),TG(48:0)]_C18:0	4769.780321	3022.156881	0.000539957
[TG(46:2)]_C14:0	LPE(22:4)	[TG(46:2)]_C16:0	3361.344242	1915.130798	0.000573348
DG(36:5)_C16:0		[TG(48:2)]_C16:1	5571.480366	3530.173581	0.000592078
[TG(42:1)]_C16:0		[TG(52:4)]_C20:4	3814.532265	2566.335503	0.000653038
[TG(59:11),TG(58:4)]_C18:2		[TG(54:7)]_C18:3	3626.284264	2325.584173	0.000664284
DG(36:8),DG(35:1)_C16:1		[TG(49:7)]_C18:1	2760.268214	1822.468133	0.00068684
[TG(58:9)]_C20:4		[TG(42:0)]_C16:0	3624.752254	2371.282181	0.00074108
CE(22:2) NH4		[TG(50:4)]_C14:0	4253.012293	2739.392182	0.000772814
[TG(44:1)]_C16:1		[TG(49:8),TG(48:1)]_C18:0	2241.132167	1711.518799	0.000870204
[TG(45:0)]_C14:0		[TG(50:7),TG(49:0)]_C16:0	3580.240266	2586.486852	0.000963482
[TG(53:9),TG(52:2)]_C20:0		[TG(57:10),TG(56:3)]_C18:2	2493.124168	1783.534117	0.001227705
DG(41:5)_C16:0		[TG(50:4)]_C16:0	2690.8522	1899.928799	0.001502937
PI(36:4)		[TG(51:9),TG(50:2)]_C14:0	9672.488721	6284.808431	0.001617846
[TG(57:8),TG(56:1)]_C18:0		[TG(48:3)]_C16:1	2489.588172	1820.774791	0.001798386
[TG(58:8)]_C20:4		[TG(50:4)]_C18:1	2583.316178	1874.702799	0.001996509
[TG(48:7),TG(47:0)]_C14:0		[TG(48:2)]_C18:2	13583.96091	6910.500475	0.00214046
DG(36:7)_C16:1		[TG(49:8),TG(48:1)]_C16:1	6665.336488	4726.909659	0.002391646
[TG(42:0)]_C14:0		[TG(48:2)]_C18:1	9800.632693	4905.155698	0.002440637
DG(30:3)_C16:1		[TG(44:1)]_C18:1	2795.892197	1516.362106	0.002596632
CAR(20:0)		[TG(56:7),TG(55:0)]_C16:0	1731.704131	2106.06415	0.002717527
FA(22:7)		[TG(44:1)]_C16:0	3017.764196	1489.377431	0.003360084
Cer(d18:0/17:0)		[TG(48:4)]_C18:2	2206.488145	1038.678071	0.003536845
CAR(10:2)		[TG(50:9),TG(49:2)]_C16:0	3121.232219	2022.228814	0.003781194
[TG(58:10)]_C20:4		[TG(51:8),TG(50:1)]_C16:0	173829.088	118231.9232	0.003812669
[TG(54:6)]_C16:1		[TG(51:8),TG(50:1)]_C16:1	5044.748363	4058.338268	0.003818969
PI(36:1),PI(O-37:1),PI(P-37:0)		[TG(50:3)]_C18:3	4585.012356	2880.115551	0.004107806
[TG(48:8),TG(47:1)]_C16:0		[TG(50:3)]_C18:1	14871.913	9984.015975	0.004231763
[TG(54:7)]_C18:1		[TG(48:3)]_C14:0	2519.196172	1376.226097	0.004255701
[TG(51:7),TG(50:0)]_C14:0		[TG(57:11),TG(56:4)]_C18:1	2842.088226	4059.004943	0.004512072
[TG(56:8)]_C18:3		LPC(16:0),PC(O-16:0),LPC(O-17:0)	115949.3561	84328.62051	0.004744344
PI(38:5)		[TG(51:8),TG(50:1)]_C14:0	3427.900245	2597.076842	0.004816483
[TG(48:8),TG(47:1)]_C14:0		[TG(57:10),TG(56:3)]_C20:0	2997.124242	2232.544166	0.005364151
DG(36:6)_C16:0		[TG(50:4)]_C18:3	3161.73621	2218.80283	0.005984208
CE(19:0)Na		[TG(54:7)]_C18:2	4314.544326	2861.670866	0.006582453
[TG(47:2)]_C18:2		[TG(46:0)]_C18:0	2369.016169	1410.779433	0.007627233

[TG(41:0)]_C16:0
[TG(48:8),TG(47:1)]_C18:1
[TG(57:9),TG(56:2)]_C16:0
[TG(44:2)]_C18:2
CE(22:1) NH4
[TG(48:4)]_C18:1
DG(30:1)_C16:0
[TG(61:10),TG(60:3)]_C18:1
CE(18:3) NH4
CE(15:1) NH4
[TG(62:16),TG(61:9),TG(60:2)]_C18:1
[TG(46:3)]_C18:2
CE(18:1)K
CE(20:1)Na
CE(18:0) NH4
[TG(46:2)]_C16:1
DG(36:7),DG(35:0)_C16:0
[TG(59:10),TG(58:3)]_C18:1
Cer(d14:2(4E,6E)/16:0)
[TG(50:7)]_C18:1
[TG(48:8),TG(47:1)]_C16:1
[TG(46:1)]_C18:0
PG(32:0),PG(O-33:0); PG(32:0),PG(O-33:0)
CE(22:4)Na
[TG(42:1)]_C18:1
[TG(44:1)]_C14:0
DG(41:6)_C16:1
DG(36:3)_C18:2
PC(14:0),LPC(15:0),LPC(O-16:0)
[TG(50:5)]_C20:4
[TG(47:6)]_C16:0
[TG(48:3)]_C18:3
FA(21:0)
[TG(58:9),TG(57:2)]_C18:1
[TG(57:8),TG(56:1)]_C18:1
DG(30:2)_C16:0
[TG(50:4)]_C20:4

[TG(51:8),TG(50:1)]_C18:0	6971.812502	5486.932413	0.008049446
LPC(18:1),PC(O-18:1),PC(P-18:0)	11896.28078	9430.950604	0.008117575
PC(35:2),PC(O-36:2),PC(P-36:1)	12649.47698	18145.94727	0.008787426
SM(d18:2/18:1)	2344.016164	2880.502868	0.008904241
[TG(55:8),TG(54:1)]_C18:0	7636.33652	9422.740001	0.009143786
PC(38:6)	51535.37183	71340.07666	0.009379177
[TG(50:3)]_C14:0	9437.672664	6646.222413	0.009678742
[TG(49:6)]_C16:0	3321.940217	2441.793494	0.009792991
[TG(54:6)]_C18:1	6163.816449	4447.096329	0.010024375
[TG(57:9),TG(56:2)]_C20:0	3263.836229	2531.129507	0.010703842
PC(34:2),PC(O-35:2),PC(P-35:1)	465272.8968	652797.4892	0.010795703
[TG(51:9),TG(50:2)]_C18:1	42349.86297	29459.52862	0.011401388
[TG(50:3)]_C16:0	19366.7294	14736.55573	0.012507182
[TG(54:6)]_C18:3	6304.284417	4764.974324	0.012910184
[TG(51:7)]_C18:1	6993.180455	5277.893665	0.013316599
[TG(56:8)]_C22:6	1995.148136	2702.918204	0.013775445
[TG(54:6)]_C20:4	6176.220473	4824.213695	0.014005834
[TG(56:8)]_C20:4	2290.000154	2030.106806	0.014648076
[TG(56:11),TG(55:4)]_C18:1	1578.968113	2045.324822	0.015828837
[TG(51:8),TG(50:1)]_C18:1	87561.54621	65073.90758	0.016864603
[TG(46:2)]_C18:1	2346.256184	1748.242791	0.017429805
PC(32:0),PC(O-33:0)	51274.60723	38558.75663	0.01761889
PC(39:5),PC(O-40:5),PC(P-40:4)	8794.060622	11321.56415	0.017652236
[TG(49:8)]_C18:2	1988.412142	1254.52542	0.017782562
[TG(48:7),TG(47:0)]_C16:0	3898.780285	1996.383474	0.018436785
[TG(51:9),TG(50:2)]_C16:0	106197.0242	77942.51497	0.020269884
[TG(54:6)]_C16:0	2909.388206	2411.661505	0.020332232
[TG(52:9),TG(51:2)]_C18:1	5858.376423	4569.54302	0.021767561
SM(d16:1/20:1)	22072.74555	28090.80277	0.021948835
[TG(52:4)]_C18:3	11703.06069	9581.203988	0.022348058
[TG(54:5)]_C18:3	6720.57248	5236.068983	0.02267626
CAR(14:2)	4012.268279	2179.23482	0.025439809
[TG(54:6)]_C18:2	22428.9616	16631.42111	0.027244661
[TG(52:5)]_C16:0	7652.828522	5855.961748	0.028519015
[TG(50:4)]_C18:2	7988.18055	6048.873781	0.029363785
[TG(51:9),TG(50:2)]_C16:1	20440.00148	15611.09107	0.030102366
[TG(54:5)]_C16:0	5617.696419	4287.002986	0.030337123
[TG(52:8),TG(51:1)]_C18:1	4212.224303	3469.347589	0.030745219
SM(d16:1/22:0)	206393.5003	268959.0268	0.036403515
SM(d16:1/22:1)	16581.64919	21724.34291	0.037273567
[TG(56:6)]_C18:2	2297.240155	2754.286878	0.037829174
[TG(54:7)]_C20:4	1647.636107	1434.8061	0.038442654
[TG(50:3)]_C18:2	24569.95376	19103.34674	0.040761169
[TG(54:10),TG(53:3)]_C16:0	1993.73215	2391.302167	0.042014466
[TG(54:5)]_C20:4	8757.92859	6831.995151	0.042244088
PC(34:1),PC(O-35:1),PC(P-35:0)	249627.5345	321061.3501	0.043559321
[TG(56:7)]_C22:5	1905.256138	2288.97283	0.049326691
[TG(50:7),TG(49:0)]_C18:0	1701.264121	1005.660735	0.051061414
[TG(52:5)]_C16:1	3385.092231	4084.048969	0.051370753
[TG(52:8),TG(51:1)]_C16:0	4295.016279	3647.77957	0.05332598
[TG(55:11),TG(54:4)]_C18:3	2592.864198	2196.844155	0.055319343
[TG(55:9),TG(54:2)]_C18:0	19670.80143	23913.51162	0.056118589
[TG(55:8),TG(54:1)]_C20:0	4660.632284	3843.652254	0.060808692
[TG(52:5)]_C18:3	7762.268568	6377.475103	0.062182391
[TG(52:6)]_C18:3	2101.004145	1689.837451	0.063582422
[TG(51:9),TG(50:2)]_C18:0	2678.696202	2326.599512	0.064274574
[TG(56:6)]_C16:0	2082.064137	2354.107493	0.064361007
PC(32:2),PC(O-33:2),PC(P-33:1)	13501.713	16298.39984	0.06536074
[TG(53:10),TG(52:3)]_C18:3	2915.7122	2545.085513	0.066852373
[TG(51:9),TG(50:2)]_C18:2	43293.85123	35196.21709	0.068327645
[TG(44:2)]_C16:0	1762.364127	1035.994071	0.068885292
[TG(57:12),TG(56:5)]_C18:2	2082.276157	2490.95618	0.069112473
CE(16:0)K	24472.47373	29614.88403	0.069671813
PC(42:4)	2071.864156	2381.074183	0.07234798
PC(35:6),PC(P-36:5)	3749.116283	3080.376211	0.074579881

		PE(O-38:9),PE(36:2),PE(O-37:2),PE(P-37:1)	2134.720153	2481.226835	0.074625499
		PC(37:3),PC(O-38:3),PC(P-38:2)	18418.38128	22599.30814	0.075447263
		PC(16:0),PC(O-17:0),LPC(O-18:0)	3861.716288	3193.406213	0.076428626
		[TG(56:7)]_C22:6	2449.968179	2792.598199	0.079980818
		PS(O-29:0)	11571.04082	9548.240641	0.081012905
		SM(d16:0/22:0)	79545.13737	98140.05234	0.082942749
		[TG(50:9),TG(49:2)]_C18:1	1832.116122	1600.442782	0.083238582
		[TG(54:8),TG(53:1)]_C16:0	1447.124095	1553.057445	0.088629232
		[TG(51:8)]_C18:2	4391.068305	3761.601623	0.091022846
		PC(36:8),PC(35:1),PC(O-36:1),PC(P-36:0)	17900.10928	21256.85608	0.092293602
		PC(42:5)	1522.488093	2159.924824	0.097169778
		SM(d16:1/18:0)	461115.2662	373769.7233	0.09795578
		[TG(51:6)]_C18:0	1658.308113	1048.178073	0.101823467
		PC(39:4),PC(O-40:4),PC(P-40:3)	8472.308606	9910.392061	0.102390074
		[TG(57:9),TG(56:2)]_C18:1	2809.908188	2363.644841	0.102470416
		[TG(58:7)]_C22:5	849.176062	1384.636097	0.103993551
		PC(38:9),PC(37:2),PC(O-38:2),PC(P-38:1)	18294.57726	21165.34749	0.104357648
		PC(34:3),PC(P-35:2)	13505.40898	15673.27921	0.108043923
		SM(d18:2/21:0)	5505.484364	6578.131086	0.109420411
		[TG(54:9),TG(53:2)]_C18:2	1350.132095	1508.908763	0.111844232
		PC(41:5),PC(P-42:4)	4097.312309	4748.485676	0.113206577
		[TG(53:8),TG(52:1)]_C18:1	40655.9908	47671.00791	0.116358598
		[TG(49:7)]_C16:1	1539.800103	1139.666743	0.117627236
		LPC(20:4)	2744.740195	3826.806955	0.119206065
		[TG(53:7),TG(52:0)]_C20:0	1746.052123	1268.032091	0.122188136
		[TG(53:7)]_C18:1	3489.824237	3860.227627	0.122886581
		[TG(54:5)]_C22:5	1966.992142	1797.778793	0.125875655
		PC(30:0),PC(O-31:0)	15187.99701	12620.99027	0.126705128
		PC(42:0)	1944.652145	2097.058817	0.128034996
		[TG(40:0)]_C16:0	1796.604127	1169.527418	0.1285478
		SM(d16:1/16:0)	29232.44176	24795.73496	0.130038052
		[TG(52:4)]_C16:1	10307.12078	12377.65763	0.130755953
		[TG(54:5)]_C18:0	2038.56813	1727.978115	0.131983089
		[TG(55:7),TG(54:0)]_C20:0	1540.632104	1427.745438	0.132798053
		[TG(50:3)]_C16:1	13439.2569	11445.05679	0.134616508
		SM(d18:2/22:1)	91084.0539	107524.215	0.135201165
		SM(d18:2/14:0)	1922.812138	2644.645523	0.136416526
		[TG(58:8)]_C22:6	1389.216095	1580.639446	0.137571042
		[TG(53:9),TG(52:2)]_C18:0	20146.08942	23395.94982	0.13780068
		PC(34:0),PC(O-35:0)	41447.27512	35716.0547	0.142726668
		PC(40:7),PC(39:0),PC(O-40:0)	5285.900388	6325.160424	0.142932784
		DG(O-40:9),DG(38:2)_C18:1	2514.596175	3923.912944	0.146154828
		PI(38:3)	1598.540107	2490.00351	0.146303341
		PC(33:2),PC(O-34:2),PC(P-34:1)	11500.48081	13507.58098	0.146428446
		PC(37:4),PC(O-38:4),PC(P-38:3)	16776.20919	19505.92944	0.146858746
		PC(40:2)	5567.932384	4842.272337	0.147650024
		[TG(56:7)]_C20:4	4364.364279	3842.330939	0.148304163
		[TG(52:5)]_C18:1	2438.024188	2097.194159	0.148465318
		[TG(54:11),TG(53:4)]_C18:1	1579.784108	2122.264141	0.151763849
		PC(38:7),PC(37:0),PC(O-38:0)	3596.460242	4055.933619	0.154094094
		SM(d18:1/19:0)	8732.764702	10373.32472	0.154921454
		LPC(18:2),LPC(P-19:1)	5201.392377	5689.825732	0.159797921
		[TG(53:8)]_C18:2	2334.272155	2559.148835	0.162570267
		PC(33:1),PC(O-34:1),PC(P-34:0)	9068.884634	10754.60803	0.163166182
		DG(O-40:9),DG(38:2)_C18:2	795787.6651	1226321.146	0.165634185
		[TG(52:9),TG(51:2)]_C16:0	4318.464288	3743.557607	0.167506929
		[TG(53:9),TG(52:2)]_C18:2	25672.57769	29700.20471	0.167869404
		[TG(49:3)]_C18:2	1435.680103	973.6500682	0.168642793
		PC(40:10),PC(39:3),PC(O-40:3),PC(P-40:2)	11143.55274	13153.6829	0.170409088
		SM(d17:1/24:1)	21907.07345	26642.54057	0.171145182
		PC(30:1),PC(O-31:1),PC(P-31:0)	161111.2383	136631.1383	0.171938604
		[TG(55:8),TG(54:1)]_C18:1	5721.240409	6256.300433	0.172377091
		[TG(54:8),TG(53:1)]_C18:0	1567.936115	1690.199448	0.172510427
		[TG(53:8),TG(52:1)]_C18:0	31749.67828	36018.42517	0.175243419
		PC(42:2)	1450.644107	1908.824133	0.17530448

	PC(38:5)	52729.04355	62020.04414	0.17647998
	[TG(57:12),TG(56:5)]_C18:1	3306.352232	3621.678927	0.18260652
	[TG(57:11),TG(56:4)]_C18:0	1638.132111	1816.07879	0.185096742
	CE(20:0)H	3897.85228	3004.308879	0.185232502
	PC(35:5),PC(O-36:5),PC(P-36:4)	17107.02511	14966.54165	0.18645634
	[TG(56:8),TG(55:1)]_C18:1	1307.156096	900.5893984	0.187160396
	[TG(52:6)]_C18:2	1777.960128	1361.410093	0.188191982
	SM(d16:0/16:0)	4496.740335	4003.110289	0.188561701
	SM(d17:1/26:1)	8138.280631	9339.810668	0.196738137
	PC(28:1),PC(P-29:0)	11877.64092	10701.12416	0.198290328
	SM(d16:0/18:0)	45305.67894	38800.11844	0.198331951
	[TG(57:12),TG(56:5)]_C22:5	909.6320663	1347.795428	0.199545944
	PC(40:9),PC(39:2),PC(O-40:2),PC(P-40:1)	6778.38049	7836.310511	0.202203872
	PC(40:1),PC(P-41:0)	3332.844223	3022.294207	0.202752022
	PC(O-38:9),PC(36:2),PC(O-37:2),PC(P-37:1)	348632.6332	399213.4341	0.202823238
	SM(d16:0/23:0)	6379.100405	7141.657178	0.20882368
	[TG(57:11),TG(56:4)]_C20:0	1750.972127	1344.865436	0.209245633
	[TG(57:12),TG(56:5)]_C20:4	2835.032219	3152.88223	0.209789588
	PC(33:3),PC(O-34:3),PC(P-34:2)	9372.148661	10559.14205	0.210065751
	PC(24:0)	2252.064162	1575.670774	0.212472656
	[TG(54:5)]_C18:1	39876.13904	34555.97507	0.2142936
	[TG(55:10),TG(54:3)]_C18:0	19675.42127	22461.74485	0.216242849
	[TG(37:0)]_C18:0	3908.820276	3434.860234	0.221388569
	PC(29:1),PC(O-30:1),PC(P-30:0)	6148.192442	5561.025711	0.221452437
	[TG(58:9)]_C22:6	860.1440575	1249.930755	0.222710079
	[TG(52:4)]_C18:2	87187.45404	101986.7417	0.224869981
	[TG(54:9),TG(53:2)]_C16:0	2106.17215	1902.792133	0.225013007
	[TG(50:4)]_C16:1	2957.900215	2672.943516	0.225599845
	PC(31:0),PC(O-32:0)	8660.192655	7864.772552	0.225866554
	LPC(20:2),PC(O-20:2)	2380.476158	2506.656171	0.227266612
	LPC(22:4)	1644.992116	2118.845481	0.233032482
	PC(43:4),PC(O-44:4)	1582.060115	2016.303474	0.236727973
	CE(19:0)H	207317.0219	269476.3571	0.244845827
	CE(18:0)K	31178.19025	44998.42112	0.246445995
	DG(39:8),DG(O-40:8)_C18:2	36697.24634	47642.60057	0.247392036
	LPI(20:0)	2087.300141	2234.756834	0.254174231
	CE(22:5) NH4	4541.216325	5711.566411	0.262724017
	Cer(d18:1/22:0)	1912.548138	1833.358133	0.266248748
	PC(29:0),PC(O-30:0)	1656.940118	2087.063486	0.268069905
	PC(42:7),PC(41:0),PC(O-42:0)	1732.264123	2177.844157	0.270737243
	[TG(53:8),TG(52:1)]_C20:0	1543.268107	1422.1181	0.271733691
	[TG(54:8),TG(53:1)]_C18:1	2101.90416	1943.047468	0.278388736
	CE(22:6) NH4	21328.90559	24157.59576	0.278620709
	PC(28:2)	2074.808141	2259.344822	0.281965868
	SM(d18:0/24:0)	8401.488634	7587.951905	0.282612744
	PC(31:2),PC(O-32:2),PC(P-32:1)	2074.896152	2246.539499	0.284953019
	SM(d18:2/24:1)	111534.6769	124565.0508	0.29045052
	[TG(56:10),TG(55:3)]_C18:1	1711.440118	1819.226799	0.292179792
	CE(20:4) NH4	153563.3779	214778.68	0.292790512
	PC(44:12),PC(O-44:5)	3005.304195	3257.244208	0.297535769
	[TG(55:8),TG(54:1)]_C16:0	2433.700168	2209.396825	0.297713555
	[TG(54:5)]_C18:2	59780.23622	52399.4058	0.302649479
	CE(18:2) NH4	1269357.343	1415151.407	0.303349059
	CAR(14:1)	3727.604277	3049.167557	0.311514324
	[TG(55:11),TG(54:4)]_C20:4	2455.304169	2212.017486	0.311937485
	PC(42:10),PC(41:3),PC(O-42:3),PC(P-42:2)	2926.968236	3143.91157	0.315418575
	PC(44:10),PC(O-44:3)	1378.560101	1696.383447	0.316869496
	[TG(55:11),TG(54:4)]_C16:0	5131.09237	4707.342305	0.317545435
	PC(41:6),PC(O-42:6)	2825.244204	3063.62754	0.317866357
	PC(40:4)	10092.92076	9255.430688	0.320976994
	PC(39:6),PC(O-40:6),PC(P-40:5)	4899.228407	5286.968387	0.322308559
	[TG(54:11),TG(53:4)]_C18:2	3007.816232	3310.306242	0.324067002
	[TG(52:7),TG(51:0)]_C18:0	1589.876111	1309.309422	0.32830684
	CE(20:1) NH4	8447.360639	10009.81074	0.330418186
	Cer(d18:1/24:1(15Z))	1083.300078	1622.280111	0.333298419

	[TG(51:6)]_C16:0	1527.340109	1267.436752	0.338884404
	CE(22:6)H	2120.912146	3164.168879	0.340218622
	PC(36:4),PC(O-37:4)	237591.0012	269500.1193	0.350249439
	PC(38:4)	159989.1512	176537.0619	0.353344299
	[TG(55:9),TG(54:2)]_C18:1	33658.20643	37756.34344	0.355926517
	SM(d16:1/23:0)	14968.26503	16439.62842	0.35598621
	[TG(53:10),TG(52:3)]_C16:1	8635.140569	9617.747339	0.365114491
	[TG(57:8),TG(56:1)]_C20:0	1590.08411	1329.487429	0.368927648
	SM(d18:1/25:0)	5075.716346	5513.263035	0.369116771
	[TG(57:10),TG(56:3)]_C18:1	3944.556283	3580.296269	0.37131622
	PC(36:7),PC(35:0),PC(O-36:0)	3824.564277	4182.750979	0.379998107
	[TG(52:7),TG(51:0)]_C16:0	1820.324127	1527.547441	0.384617453
	PC(34:6)	1626.896111	1949.032798	0.387930225
	PC(19:1),LPC(20:1),PC(O-20:1),PC(P-20:0)	1916.416137	1615.846113	0.390225564
	[TG(53:7),TG(52:0)]_C16:0	5745.540427	5434.723727	0.392109146
	[TG(53:9),TG(52:2)]_C16:1	3416.104257	3684.08093	0.400217995
	[TG(52:8),TG(51:1)]_C18:0	1693.152117	1577.405438	0.410097873
	[TG(54:9),TG(53:2)]_C18:0	1555.43211	1654.965453	0.410306242
	[TG(52:9),TG(51:2)]_C18:2	2627.020187	2407.960172	0.413186913
	[TG(55:9),TG(54:2)]_C20:0	3196.328233	2910.904874	0.421192526
	SM(d16:1/24:1)	112030.912	121370.4694	0.422487694
	PI(36:2),PI(O-37:2),PI(P-37:1)	2926.704208	2774.850869	0.426537322
	[TG(56:8)]_C18:2	1849.440132	1722.620128	0.426572194
	[TG(52:10),TG(51:3)]_C16:0	2405.788171	2240.574828	0.429191225
	PC(42:9),PC(41:2),PC(O-42:2),PC(P-42:1)	2242.356163	2398.352849	0.429552581
	CE(20:2)K	5259.84034	6264.503767	0.429856622
	PC(40:3)	4821.66037	4504.480311	0.431883166
	Cer(d18:1/23:0)	1935.456138	1855.279465	0.438643773
	SM(d16:1/18:1)	34137.98651	37458.00655	0.439342862
	SM(d16:1/17:0)	16172.0491	14882.83901	0.449265784
	[TG(57:10),TG(56:3)]_C18:0	957.6160698	1322.722753	0.454217543
	[TG(54:10),TG(53:3)]_C18:1	4222.864306	4471.900984	0.454525735
	[TG(55:10),TG(54:3)]_C18:2	20528.56958	22273.95625	0.457085396
	PC(39:8),PC(O-40:8),PC(38:1),PC(O-39:1),PC(P-39:0)	30838.18624	28697.66604	0.457481372
	SM(d18:1/12:0)	2134.664147	1831.08746	0.459526389
	[TG(57:12),TG(56:5)]_C16:0	1894.92013	1800.970128	0.472795214
	[TG(56:6)]_C18:0	1355.996087	1587.84278	0.473091163
	PC(38:3)	107460.0681	99419.92676	0.474962768
	[TG(56:8),TG(55:1)]_C16:0	1104.488075	1278.731424	0.476344226
	[TG(53:9),TG(52:2)]_C16:0	164098.7919	151459.9712	0.501706911
	PC(39:7),PC(P-40:6),PC(38:0),PC(O-39:0)	6123.356425	6454.733107	0.503757506
	[TG(56:6)]_C20:4	4914.584341	5165.457732	0.503975199
	SM(d18:0/24:1)	65945.01252	61441.92236	0.507341942
	[TG(44:0),TG(O-45:0)]_C18:0	1099.828076	854.4780605	0.512288936
	[TG(54:11),TG(53:4)]_C16:0	1677.264126	1793.390799	0.513920201
	[TG(53:10),TG(52:3)]_C18:0	3601.856216	3405.356218	0.516108049
	SM(d18:0/26:1(17Z))	3370.964246	3194.170885	0.523014354
	PC(37:7),PC(P-38:6),PC(36:0),PC(O-37:0)	13633.22493	12659.92816	0.526582693
	CE(22:5)H	7558.652528	7197.162516	0.533069657
	[TG(52:10),TG(51:3)]_C18:1	2912.972202	2734.68553	0.535980934
	CE(20:2)Na	7023.188535	7417.04856	0.537133371
	[TG(54:9),TG(53:2)]_C18:1	4396.004336	4542.377668	0.543762488
	LPC(20:3)	1766.424122	1399.264106	0.546363838
	PC(40:5)	16007.96517	15141.46512	0.553752957
	[TG(55:10),TG(54:3)]_C18:1	102265.4914	109312.0977	0.554490111
	CE(16:3)Na	3291.360246	4189.190287	0.554576519
	[TG(55:10),TG(54:3)]_C16:0	4529.588304	4760.231683	0.557742465
	PE(36:3),PE(P-37:2)	1699.236117	1508.549449	0.559287917
	[TG(46:3)]_C18:1	1143.17208	912.2253995	0.561455758
	SM(d17:0/27:0)	1273.748085	1611.631446	0.561839002
	CE(16:1) NH4	17220.85305	19505.11332	0.5696417
	PC(33:0),PC(O-34:0)	4738.20031	4924.707029	0.571942587
	SM(d16:0/24:0)	30780.70217	28984.37541	0.573612729
	[TG(56:12),TG(55:5)]_C18:1	1583.480116	1662.270119	0.580453436
	[TG(53:8),TG(52:1)]_C16:1	882.7760605	1101.729407	0.586017357

	SM(d16:1/20:0)	71006.11227	66763.49243	0.613194094
	PC(42:11),PC(41:4),PC(O-42:4)	3621.264246	3750.544254	0.622660292
	PC(35:4),PC(O-36:4),PC(P-36:3)	25855.44176	24644.05496	0.624001073
	[TG(54:5)]_C16:1	870.5200577	1059.640073	0.627153033
	PC(35:3),PC(O-36:3),PC(P-36:2)	11178.76079	11707.03418	0.629615463
	PC(42:6)	2130.928149	2084.968153	0.632663894
	[TG(57:9),TG(56:2)]_C18:0	1508.844101	1369.837426	0.648271227
	PC(40:6)	21805.09753	22985.62446	0.651342088
	[TG(52:5)]_C18:2	10415.64466	10976.65813	0.652082534
	PC(32:3),PC(P-33:2)	1941.684136	1777.27412	0.673847456
	[TG(55:11),TG(54:4)]_C18:2	53729.60755	56757.32138	0.679501131
	[TG(52:6)]_C16:0	1224.956088	1074.466085	0.680803633
	[TG(53:10),TG(52:3)]_C18:2	154818.7563	163242.5154	0.684558263
	CE(20:3) NH4	22210.60972	18245.34588	0.692582962
	PC(28:0),PC(O-29:0)	3559.012263	3433.752245	0.695392123
	[TG(53:10),TG(52:3)]_C18:1	173276.4409	180358.2175	0.701899185
	PC(30:2),PC(P-31:1)	15726.64516	16353.15175	0.706023784
	[TG(52:4)]_C18:1	18581.81721	19354.91414	0.706362239
	CE(18:1) NH4	163135.7643	169922.704	0.707328998
	SM(d16:1/24:0)	194260.1047	201645.4958	0.712212633
	[TG(51:4)]_C18:2	2010.020145	1945.606796	0.713659741
	[TG(57:11),TG(56:4)]_C18:2	2562.440192	2641.906855	0.715433367
	[TG(55:9),TG(54:2)]_C18:2	4541.900333	4722.467009	0.71561301
	PC(42:8),PC(41:1),PC(O-42:1),PC(P-42:0)	2554.764178	2508.057505	0.728605243
	[TG(50:5)]_C18:2	1321.832095	1422.638761	0.732089805
	[TG(52:10),TG(51:3)]_C18:2	3419.944235	3302.390898	0.737499352
	PC(40:8),PC(39:1),PC(O-40:1),PC(P-40:0)	5313.320372	5144.540365	0.741956866
	[TG(53:8),TG(52:1)]_C16:0	37614.88252	36490.72923	0.743733958
	SM(d18:1/17:0)	9837.500657	9561.42731	0.751306096
	PC(32:1),PC(O-33:1),PC(P-33:0)	35834.04254	34651.63576	0.752466002
	[TG(58:8)]_C22:5	1028.416074	1128.67275	0.764914898
	SM(d18:0/15:0)	2843.98421	2912.284223	0.774016007
	[TG(53:10),TG(52:3)]_C16:0	178293.6412	184359.0279	0.780851395
	PC(42:3)	2192.068153	2157.384827	0.785724732
	PC(36:3),PC(P-37:2)	201735.5286	207588.4596	0.787657431
	SM(d16:0/20:0)	11485.3087	11207.87548	0.794509934
	PC(36:5)	9770.044671	9998.334704	0.808573712
	CE(22:6)Na	2710.800187	2685.803523	0.822745889
	PC(38:8),PC(37:1),PC(O-38:1),PC(P-38:0)	19668.82139	20046.24817	0.834659351
	PG(16:0),LPG(17:0),LPG(O-18:0); PG(16:0),LPG(17:0),LPG(O-18:0)	3305.524234	3009.810871	0.834931045
	PC(37:5),PC(O-38:5),PC(P-38:4)	19023.00532	18675.15539	0.843509074
	[TG(55:9),TG(54:2)]_C16:0	3971.076266	4054.486287	0.84417981
	[TG(56:6)]_C22:5	2473.752168	2525.655504	0.847261753
	PC(41:7),PC(P-42:6),PC(40:0),PC(O-41:0)	2155.564159	2289.140839	0.851486031
	[TG(39:0)]_C20:0	15714.01704	16102.86388	0.853731534
	Cer(d18:1/24:0)	3252.788235	3214.071571	0.860038225
	[TG(55:11),TG(54:4)]_C18:1	95535.93858	97414.62246	0.866231016
	[TG(52:4)]_C16:0	59099.62046	60302.98693	0.873769003
	DG(39:7)_C18:1	8455.228554	8336.261895	0.875429268
	[TG(57:12),TG(56:5)]_C18:0	1879.676134	1906.316132	0.87846509
	[TG(54:12),TG(53:5)]_C18:2	796.2520569	854.0287305	0.889655551
	SM(d18:1/26:1(17Z))	3036.124213	2996.95088	0.89050709
	SM(d18:0/17:0)	5403.796391	5348.603044	0.893218227
	CE(18:3)Na	21960.18946	23210.29296	0.894584664
	SM(d16:0/25:0)	6536.764431	6466.364452	0.898830935
	DG(O-38:8),DG(36:1)_C16:1	12566.55685	11841.93688	0.905587999
	CE(20:0) NH4	28552.606	28898.83624	0.909233078
	[TG(55:10),TG(54:3)]_C20:0	775.9840584	819.1340605	0.913948596
	[TG(52:6)]_C16:1	843.5880608	799.7480562	0.91405225
	PC(O-38:8),PC(36:1),PC(O-37:1),PC(P-37:0)	115545.8238	114319.5033	0.91434652
	LPG(20:0); LPG(20:0)	3078.076225	3107.812895	0.914915321
	[TG(54:10),TG(53:3)]_C18:2	3100.996242	3127.516226	0.918403801
	PC(O-40:9),PC(38:2),PC(P-39:1)	77542.47316	76728.45325	0.924461216
	SM(d18:1/24:1(15Z))	161983.1121	160414.429	0.928300435
	[TG(59:13),TG(58:6)]_C18:1	793.948056	826.7713891	0.935464085

	PC(43:6)	2630.488203	2619.191527	0.943115194
	SM(d16:1/25:0)	32084.23427	31884.56426	0.944853005
	DG(39:8),DG(O-40:8),DG(38:1)_C18:1	149415.5674	150544.0377	0.945079275
	PC(31:1),PC(O-32:1),PC(P-32:0)	6882.408476	6846.985159	0.955989402
	[TG(56:7)]_C18:2	2632.176189	2622.372841	0.961566639
	PC(36:6)	1671.432116	1690.465458	0.970680354
	[TG(53:7),TG(52:0)]_C18:0	7662.456539	7644.219867	0.979060475
	[TG(55:11),TG(54:4)]_C18:0	8960.024686	8936.334681	0.981102602
	[TG(52:9),TG(51:2)]_C16:1	1107.924075	1101.207409	0.984171235
	PC(37:6),PC(O-38:6),PC(P-38:5)	7898.236519	7910.016547	0.984731867
	1-O-tricosanoyl-Cer(d18:1/16:0)	1400.968097	1394.714768	0.988360462
	PI(38:4)	7099.38047	7104.463798	0.993444595
	[TG(53:9),TG(52:2)]_C18:1	256596.3567	256797.1453	0.994112537
	SM(d18:2/15:0)	1098.092075	1095.092076	0.995632922
	[TG(56:12),TG(55:5)]_C18:2	1161.252077	1160.588749	0.998521989

Comparison of Lipid Corona Profiles Between Sexes

Table S7. Comparison of Lipid Corona Profiles Between Sexes

Unique Lipids in Males	Unique Lipds in Females	Shared Lipids
DG(34:2)_C18:2	DG(32:2)_C18:1	DG(37:7),DG(36:0)_C16:0
DG(39:8),DG(O-40:8)_C18:2	FA(18:0)	DG(35:6)_C18:0
CE(15:1)K	FA(15:1)	DG(40:5)_C18:0
CE(18:0) NH4	FA(21:0)	CE(18:1) NH4
CE(18:3)H	DG(34:3)_C18:1	DG(38:5)_C16:0
CE(18:0)K	FA(20:0)	DG(32:0)_C16:0
DG(O-38:8),DG(36:1)_C16:1		FA(22:7)
DG(36:3)_C18:1		DG(30:0)_C16:0
CE(22:1)H		CE(18:2) NH4
CE(16:0)Na		DG(39:7),DG(38:0),DG(dO-40:0)_C18:0
DG(40:6),DG(dO-40:0)_C16:0		DG(34:0)_C18:0
DG(42:6)_C16:0		CE(20:4) NH4
CE(22:2) NH4		DG(33:0)_C16:0
CE(20:2)Na		CE(19:0)H
DG(O-38:8),DG(36:1)_C18:0		CE(18:2)Na
CE(20:3) NH4		FA(17:2)
DG(O-38:8),DG(36:1)_C18:1		CE(18:3) NH4
DG(32:1)_C16:0		CE(20:5)H
CE(16:1) NH4		DG(32:0)_C18:0
FA(26:1)		DG(37:7),DG(36:0)_C18:0
CE(18:3)Na		CE(16:0) NH4
CE(16:0)K		FA(6:0)
[TG(38:0)]_C20:0		FA(19:2)
DG(O-38:9),DG(36:2)_C18:1		DG(37:6)_C18:0
CE(20:2)K		DG(34:1)_C16:0
FA(28:3)		DG(35:6)_C16:0
CE(22:5) NH4		LPG(19:0),LPG(O-20:0); LPG(19:0),LPG(O-20:0)
DG(39:8),DG(O-40:8),DG(38:1)_C18:1		CE(22:6) NH4
CE(18:1)Na		DG(34:1)_C18:1
		DG(42:5)_C18:0
		DG(34:0)_C16:0
		DG(36:7),DG(35:0)_C18:0
		DG(40:5)_C16:0
		CE(20:5) NH4
		DG(34:4),DG(dO-36:4)_C16:1
		DG(O-40:9),DG(38:2)_C18:2
		PG(16:0),LPG(17:0),LPG(O-18:0); PG(16:0),LPG(17:0),LPG(O-18:0)

Comparison of Lipid Corona Profiles Between Sexes

Table S7. Comparison of Lipid Corona Profiles Between Sexes

Unique Lipids in Males	Unique Lipds in Females	Shared Lipids
CE(20:5)Na	FA(22:7)	DG(37:7),DG(36:0)_C16:0
CE(20:0) NH4	DG(32:2)_C18:1	DG(35:6)_C18:0
CE(16:2)Na	FA(17:2)	DG(34:2)_C18:2
CE(15:1)K	FA(15:1)	DG(40:5)_C18:0
CE(18:0) NH4	FA(21:0)	CE(18:1) NH4
CE(18:3)H	LPG(19:0),LPG(O-20:0); LPG(19:0),LPG(O-20:0)	DG(39:8),DG(O-40:8)_C18:2
DG(39:7),DG(38:0),DG(dO-40:0)_C18:0	DG(34:3)_C18:1	DG(38:5)_C16:0
DG(O-38:8),DG(36:1)_C16:1	FA(20:0)	DG(32:0)_C16:0
CE(22:4)Na	PG(16:0),LPG(17:0),LPG(O-18:0); PG(16:0),LPG(17:0),LPG(O-18:0)	DG(30:0)_C16:0
CE(18:2)K		CE(18:2) NH4
CE(22:1)H		CE(18:0)K
DG(36:3)_C18:1		DG(34:0)_C18:0
DG(40:6),DG(dO-40:0)_C16:0		CE(20:4) NH4
DG(34:2)_C16:0		DG(33:0)_C16:0
DG(36:3)_C18:2		CE(16:0)Na
DG(O-38:8),DG(36:1)_C18:0		CE(19:0)H
CE(20:3) NH4		CE(18:2)Na
DG(O-38:8),DG(36:1)_C18:1		CE(22:2) NH4
DG(32:1)_C16:0		CE(18:3) NH4
CE(16:1)Na		CE(20:5)H
DG(37:6)_C16:0		DG(32:0)_C18:0
CE(16:1) NH4		DG(37:7),DG(36:0)_C18:0
DG(39:7)_C18:1		CE(16:0) NH4
CE(16:3)Na		DG(37:6)_C18:0
CE(18:3)Na		DG(34:1)_C16:0
DG(36:4),DG(O-37:4)_C18:2		DG(35:6)_C16:0
CE(16:0)K		CE(22:6) NH4
[TG(38:0)]_C20:0		DG(34:1)_C18:1
[TG(44:5)]_C20:0		DG(42:5)_C18:0
CE(20:2)K		DG(34:0)_C16:0
DG(O-38:9),DG(36:2)_C18:1		DG(40:5)_C16:0
DG(36:7),DG(35:0)_C18:0		CE(20:5) NH4
CE(20:4)H		DG(34:4),DG(dO-36:4)_C16:1
CE(19:0)Na		DG(39:8),DG(O-40:8),DG(38:1)_C18:1
CE(22:5) NH4		DG(O-40:9),DG(38:2)_C18:2
CE(18:1)Na		

Table S7. Comparison of Lipid Corona Profiles Between Sexes
100 nm 25% BC Samples

Unique Lipids in Males	Unique Lipds in Females	Shared Lipids
CE(18:1)K	PG(20:0),LPG(21:0); PG(20:0),LPG(21:0)	DG(37:7),DG(36:0)_C16:0
CE(20:5)Na	DG(34:3)_C18:1	CE(20:0) NH4
CE(17:0) NH4	PG(16:0),LPG(17:0),LPG(O-18:0); PG(16:0),LPG(17:0),LPG(O-18:0)	DG(35:6)_C18:0
CE(19:0) NH4		DG(34:2)_C18:2
CE(19:0)K		DG(40:5)_C18:0
CE(16:2)Na		CE(18:1) NH4
CE(18:3)K		DG(39:8),DG(O-40:8)_C18:2
CE(22:6)H		DG(38:5)_C16:0
DG(34:2)_C18:1		CE(15:1)K
CE(22:4)K		DG(32:0)_C16:0
CE(20:5)K		CE(18:3)H
709.686225 -> 369.2		DG(39:7),DG(38:0),DG(dO-40:0)_C18:0
CE(18:0) NH4		CE(18:2) NH4
CE(14:0) NH4		DG(30:0)_C16:0
CE(20:0)H		CE(18:0)K
DG(38:3)_C18:2		DG(32:2)_C18:1
CE(20:3)Na		DG(O-38:8),DG(36:1)_C16:1
CE(22:4)Na		DG(34:0)_C18:0
CE(18:2)K		CE(20:4) NH4
DG(34:2)_C16:1		CE(22:1)H
DG(32:1)_C16:1		DG(33:0)_C16:0
DG(40:6),DG(dO-40:0)_C16:0		DG(36:3)_C18:1
[TG(49:7),TG(48:0)]_C16:0		CE(16:0)Na
DG(34:2)_C16:0		CE(19:0)H
FA(14:2)		CE(18:2)Na
CE(20:2)Na		CE(22:2) NH4
DG(36:3)_C18:2		CE(18:3) NH4
FA(6:0)		CE(20:5)H
FA(10:3)		DG(32:0)_C18:0
DG(O-38:8),DG(36:1)_C18:0		DG(37:7),DG(36:0)_C18:0
CE(20:0)Na		CE(16:0) NH4
DG(O-38:8),DG(36:1)_C18:1		CE(20:3) NH4
DG(32:5)_C18:1		DG(37:6)_C18:0
CE(22:3) NH4		DG(34:1)_C16:0
DG(32:1)_C16:0		CE(16:1)Na
CE(20:4)Na		CE(16:1) NH4
DG(40:9),DG(39:2)_C18:2		DG(35:6)_C16:0
FA(15:1)		CE(18:3)Na
DG(38:7),DG(37:0)_C16:0		DG(36:4),DG(O-37:4)_C18:2
CE(22:1) NH4		CE(22:6) NH4
DG(37:6)_C16:0		DG(34:1)_C18:1
CE(20:1)K		CE(16:0)K
Cer(d14:2(4E,6E)/16:0)		DG(42:5)_C18:0
DG(39:7)_C18:1		DG(34:0)_C16:0
DG(44:7),DG(43:0)_C16:0		DG(O-38:9),DG(36:2)_C18:1
CE(16:3)Na		DG(36:7),DG(35:0)_C18:0
DG(O-38:9),DG(36:2)_C18:2		DG(40:5)_C16:0
DG(O-40:9),DG(38:2)_C18:1		CE(20:5) NH4
CE(20:1) NH4		DG(34:4),DG(dO-36:4)_C16:1
CE(15:0)K		DG(39:8),DG(O-40:8),DG(38:1)_C18:1
DG(32:1)_C18:1		DG(O-40:9),DG(38:2)_C18:2
[TG(38:0)]_C20:0		
CE(20:2)K		
CE(20:2) NH4		
Cer(d18:0/21:0)		
CE(20:4)H		
CE(19:0)Na		
CE(22:5) NH4		
CE(22:2)H		
DG(O-38:9),DG(36:2)_C18:0		
CE(22:3)H		
CE(18:1)Na		

Table S7. Comparison of Lipid Corona Profiles Between Sexes
100 nm 50% BC Samples

Unique Lipids in Males	Unique Lipds in Females	Shared Lipids
CE(17:0) NH4	PE(38:4)	CE(18:1)K
[TG(57:12),TG(56:5)]_C16:0	PC(35:2),PC(O-36:2),PC(P-36:1)	CE(20:5)Na
CE(18:0)H	DG(32:2)_C18:1	CE(20:0) NH4
709.686225 -> 369.2	PC(39:5),PC(O-40:5),PC(P-40:4)	DG(34:2)_C18:2
[TG(52:5)]_C20:4	PS(38:4)	DG(40:5)_C18:0
DG(32:1)_C16:1	PS(P-37:0)	CE(19:0)K
DG(40:2)_C18:2	PE(34:2),PE(O-35:2),PE(P-35:1)	CE(18:1) NH4
[TG(56:10),TG(55:3)]_C18:1	LPG(19:0),LPG(O-20:0); LPG(19:0),LPG(O-20:0)	CE(18:3)K
DG(42:7),DG(41:0)_C16:0	PC(38:6)	CE(22:6)H
[TG(59:10),TG(58:3)]_C18:1	DG(34:3)_C18:1	DG(39:8),DG(O-40:8)_C18:2
CE(20:0)K	PG(16:0),LPG(17:0),LPG(O-18:0); PG(16:0),LPG(17:0),LPG(O-18:0)	DG(38:5)_C16:0
DG(32:1)_C16:0		CE(15:1)K
DG(30:1)_C18:1		CE(18:0) NH4
[TG(55:8),TG(54:1)]_C18:1		CE(18:3)H
CE(20:4)K		DG(38:3)_C18:2
[TG(50:4)]_C16:0		DG(30:0)_C16:0
DG(32:1)_C18:1		DG(O-38:8),DG(36:1)_C16:1
CE(22:4) NH4		CE(18:2)K
CE(22:6)Na		CE(20:4) NH4
[TG(56:11),TG(55:4)]_C18:1		DG(33:0)_C16:0
[TG(57:10),TG(56:3)]_C18:2		DG(36:3)_C18:1
[TG(56:6)]_C22:5		CE(18:2)Na
CE(22:4)K		CE(22:2) NH4
CE(20:3)Na		CE(16:0) NH4
[TG(54:7)]_C20:4		DG(O-38:8),DG(36:1)_C18:0
DG(24:0)_C18:0		CE(20:3) NH4
[TG(54:11),TG(53:4)]_C18:1		CE(20:0)Na
LPC(20:4)		DG(O-38:8),DG(36:1)_C18:1
DG(40:6),DG(dO-40:0)_C16:0		CE(22:3) NH4
DG(37:7)_C16:1		DG(40:9),DG(39:2)_C18:2
CE(16:2) NH4		CE(22:1) NH4
[TG(49:7),TG(48:0)]_C16:0		DG(39:7)_C18:1
DG(36:4),DG(O-37:4)_C18:1		DG(O-38:9),DG(36:2)_C18:2
PC(14:0),LPC(15:0),LPC(O-16:0)		PC(39:7),PC(P-40:6),PC(38:0),PC(O-39:0)
[TG(59:10),TG(58:3)]_C18:2		CE(18:3)Na
DG(32:2)_C18:2		CE(22:5)H
[TG(50:3)]_C18:3		DG(34:0)_C16:0
[TG(48:8),TG(47:1)]_C16:0		CE(20:2) NH4
DG(40:8),DG(39:1)_C18:1		DG(40:5)_C16:0
[TG(48:4)]_C18:2		CE(20:4)H
[TG(48:3)]_C18:3		CE(20:5) NH4
CE(20:1)K		CE(22:5) NH4
[TG(51:7),TG(50:0)]_C18:0		DG(34:4),DG(dO-36:4)_C16:1
DG(44:7),DG(43:0)_C16:0		DG(39:8),DG(O-40:8),DG(38:1)_C18:1
CE(24:1)H		CE(22:3)H
DG(44:8),DG(43:1)_C16:0		CE(18:1)Na
[TG(54:5)]_C20:4		DG(37:7),DG(36:0)_C16:0
DG(34:3)_C18:2		DG(35:6)_C18:0
CE(17:1) NH4		CE(19:0) NH4
[TG(52:8),TG(51:1)]_C18:0		CE(16:2)Na
DG(O-38:9),DG(36:2)_C18:0		DG(34:2)_C18:1
CE(22:2)H		PC(34:3),PC(P-35:2)
		CE(20:5)K
		DG(32:0)_C16:0
		CE(14:0) NH4
		CE(20:0)H
		CE(18:2) NH4
		DG(39:7),DG(38:0),DG(dO-40:0)_C18:0
		CE(18:0)K
		DG(34:0)_C18:0
		CE(22:4)Na
		CE(22:1)H
		[TG(56:8)]_C22:6
		CE(16:0)Na
		CE(19:0)H
		CE(18:3) NH4
		DG(34:2)_C16:0
		CE(20:5)H
		DG(32:0)_C18:0
		CE(20:2)Na
		DG(37:7),DG(36:0)_C18:0
		DG(36:3)_C18:2
		DG(37:6)_C18:0
		PC(34:2),PC(O-35:2),PC(P-35:1)
		CE(20:4)Na
		FA(15:1)
		DG(34:1)_C16:0
		CE(16:1)Na

		DG(37:6)_C16:0
		CE(16:1) NH4
		DG(35:6)_C16:0
		CE(16:3)Na
		DG(O-40:9),DG(38:2)_C18:1
		DG(36:4),DG(O-37:4)_C18:2
		CE(20:1) NH4
		CE(22:6) NH4
		CE(16:0)K
		DG(34:1)_C18:1
		DG(42:5)_C18:0
		DG(36:7),DG(35:0)_C18:0
		DG(O-38:9),DG(36:2)_C18:1
		CE(20:2)K
		CE(19:0)Na
		DG(O-40:9),DG(38:2)_C18:2

Table S7. Comparison of Lipid Corona Profiles Between Sexes
100 nm 75% Serum BC Samples

Unique Lipids in Males	Unique Lipds in Females	Shared Lipids	Male Average of Shared Lipids	Female Average of Shared Lipids	p-value of Shared Lipids
[TG(46:2)]_C16:0	DG(32:2)_C18:1	DG(35:6)_C16:0	6298.628462	4726.031679	7.90074E-06
SM(d18:1/12:0)	[TG(57:11),TG(56:4)]_C18:1	CE(22:3) NH4	5115.116364	3522.00959	1.11142E-05
PC(44:10),PC(O-44:3)	PS(38:4)	CE(16:1)Na	12307.92487	7121.821156	1.14966E-05
[TG(54:6)]_C18:2	[TG(56:11),TG(55:4)]_C18:2	DG(34:2)_C18:2	12456.52892	8533.045304	2.11415E-05
PI(36:2),PI(O-37:2),PI(P-37:1)	PC(27:0),PC(O-28:0)	DG(O-38:8),DG(36:1)_C18:1	4730.07634	3563.442926	2.43209E-05
[TG(54:11),TG(53:4)]_C18:2	[TG(54:8),TG(53:1)]_C18:1	DG(39:7),DG(38:0),DG(dO-40:0)_C18:0	8760.124622	5809.861083	2.62441E-05
[TG(57:12),TG(56:5)]_C18:1	SM(d18:2/14:0)	DG(34:2)_C16:0	10149.24472	7322.547888	4.5177E-05
PC(39:8),PC(O-40:8),PC(38:1),PC(O-39:1),PC(P-39:0)	LPG(19:0),LPG(O-20:0); LPG(19:0),LPG(O-20:0)	CE(18:2)K	8425.772578	5878.515748	5.20457E-05
[TG(57:12),TG(56:5)]_C16:0	[TG(58:8)]_C22:6	CE(16:0)K	15174.24112	10415.30741	8.27984E-05
LPI(20:0)	[TG(53:7)]_C18:1	CE(22:2) NH4	23080.4416	13912.89761	9.69514E-05
[TG(54:5)]_C18:3	PE(O-38:8),PE(36:1),PE(O-37:1),PE(P-37:0)	DG(34:0)_C16:0	196528.2469	122256.2447	9.87363E-05
[TG(52:4)]_C18:3	LPE(20:4)	DG(32:0)_C16:0	324944.1593	201996.5666	9.94456E-05
SM(d16:1/16:0)	PS(P-37:0)	CE(20:4)H	12581.32889	7595.591878	0.000140093
PC(39:4),PC(O-40:4),PC(P-40:3)	PE(34:1),PE(O-35:1),PE(P-35:0)	DG(36:3)_C18:2	8871.000618	6420.883781	0.000149411
CE(18:0)H	CAR(10:2)	CE(18:1)K	7006.472491	4860.215681	0.000170476
[TG(51:7),TG(50:0)]_C16:0	[TG(53:8)]_C18:2	CE(18:2)Na	86738.53798	55085.23927	0.000184196
PC(O-38:9),PC(36:2),PC(O-37:2),PC(P-37:1)	PE(38:5)	CE(18:1)Na	12224.98886	7761.8552	0.000190173
PC(35:5),PC(O-36:5),PC(P-36:4)	DG(34:3)_C18:1	DG(34:0)_C18:0	249059.6319	156505.8494	0.000208702
LPC(16:0),PC(O-16:0),LPC(O-17:0)	PG(16:0),LPG(17:0),LPG(O-18:0); PG(16:0),LPG(17:0),LPG(O-18:0)	DG(O-38:9),DG(36:2)_C18:1	15594.96913	10578.77877	0.000218254
CE(22:5)Na		CE(20:5) NH4	23870.2857	14812.4284	0.000236679
[TG(48:2)]_C14:0		DG(34:1)_C18:1	17422.62129	10809.99742	0.000245678
[TG(52:4)]_C20:4		CE(22:2)H	3605.740264	2637.440187	0.000288078
PC(42:5)		CE(16:0) NH4	27148.53396	17929.44989	0.000364617
[TG(51:9),TG(50:2)]_C16:1		CE(19:0)Na	7757.136572	5119.609696	0.000405922
[TG(48:8),TG(47:1)]_C18:1		CE(20:5)H	93052.4907	58500.8447	0.000505477
PC(30:0),PC(O-31:0)		CE(20:5)Na	7086.768502	5108.195023	0.000517206
PC(40:1),PC(P-41:0)		DG(37:7),DG(36:0)_C18:0	127242.5932	85791.69701	0.000615106
[TG(46:1)]_C18:1		CE(18:3) NH4	64223.10452	42309.60305	0.000620269
SM(d16:1/24:0)		CE(15:1)K	23921.97367	16508.20316	0.000649247
[TG(54:10),TG(53:3)]_C18:1		CE(18:3)H	26459.13391	18284.05659	0.000763064
DG(42:7),DG(41:0)_C16:0		DG(34:1)_C16:0	16649.28926	10772.37545	0.000827526
PC(36:7),PC(35:0),PC(O-36:0)		CE(20:0)Na	3614.50825	2952.998209	0.000930499
SM(d16:0/25:0)		DG(37:7),DG(36:0)_C16:0	25499.68191	17820.59456	0.000978013
[TG(52:5)]_C16:0		CE(22:1) NH4	3561.864245	2998.250879	0.001012937
[TG(52:5)]_C18:3		CE(16:0)Na	24878.65374	17849.80658	0.001036801
[TG(56:6)]_C20:4		DG(O-38:9),DG(36:2)_C18:2	3595.54824	3051.304882	0.001098726
[TG(49:8),TG(48:1)]_C16:0		CE(20:3) NH4	30076.80601	19444.4188	0.001133476
[TG(55:10),TG(54:3)]_C18:0		CE(18:3)Na	30824.71826	20246.78082	0.001167096
PC(39:6),PC(O-40:6),PC(P-40:5)		CE(22:1)H	16531.79316	11625.4828	0.001198097
PI(38:4)		CE(22:5) NH4	5330.880392	4295.266985	0.001922215
[TG(52:10),TG(51:3)]_C18:1		DG(37:6)_C18:0	5015.300372	3919.826944	0.001990685
[TG(48:3)]_C18:2		DG(O-38:8),DG(36:1)_C18:0	4474.044323	3300.337563	0.002095075
SM(d16:1/25:0)		DG(30:0)_C16:0	4526.396336	3572.73625	0.002106004
[TG(51:8)]_C18:2		CE(22:3)H	3419.644241	2879.144201	0.002336683
PC(41:6),PC(O-42:6)		DG(O-38:8),DG(36:1)_C16:1	16846.47315	11807.62279	0.002386439
[TG(48:2)]_C16:0		DG(36:4),DG(O-37:4)_C18:2	6049.688441	4830.575001	0.002667483
PC(44:5)		DG(36:3)_C18:1	9659.548689	7092.165177	0.00267987
[TG(56:12),TG(55:5)]_C18:2		DG(35:6)_C18:0	6708.75649	5335.159703	0.003092277
CE(20:4)K		CE(20:4)Na	3988.084277	3203.494237	0.003264789
SM(d16:1/23:0)		DG(33:0)_C16:0	3606.852248	3158.778876	0.00344687
[TG(56:8)]_C18:2		CE(18:0) NH4	8001.360563	5931.940415	0.003624637
Cer(d18:0/21:0)		CE(22:4)Na	6123.240451	4449.880327	0.005106258
LPC(20:3)		CE(20:1) NH4	5240.856355	4209.486309	0.006197147
PC(42:1)		CE(16:2)Na	6480.508434	4955.068323	0.007280608
PC(36:3),PC(P-37:2)		CE(18:1) NH4	141879.8946	97164.70085	0.009436037
[TG(50:4)]_C16:1		DG(39:8),DG(O-40:8),DG(38:1)_C18:1	125933.7728	87676.70646	0.010867589
LPC(18:0),PC(O-18:0),LPC(O-19:0)		CE(20:4) NH4	222259.1083	158109.2301	0.010911645
PE(37:6),PE(O-38:6),PE(P-38:5)		DG(32:0)_C18:0	3986.876281	3465.659565	0.014112047
PC(37:6),PC(O-38:6),PC(P-38:5)		LPC(20:4)	10276.10868	5413.295025	0.014916465
LPC(22:5)		CE(18:0)K	48200.71954	35376.93527	0.015073778
PC(32:1),PC(O-33:1),PC(P-33:0)		CE(20:0) NH4	8636.836626	6886.569846	0.018580858

[TG(39:0)]_C20:0		LPC(18:2),LPC(P-19:1)	13609.90494	7296.071195	0.018802665
[TG(55:11),TG(54:4)]_C18:2		CE(16:1) NH4	17575.73325	13237.50291	0.019992467
[TG(54:6)]_C18:1		CE(19:0) NH4	4898.844346	4161.02096	0.021431787
[TG(50:3)]_C14:0		DG(38:3)_C18:2	4705.416342	3896.946268	0.021804823
[TG(49:8),TG(48:1)]_C18:1		CE(16:3)Na	4533.844326	3867.59429	0.023789972
[TG(50:4)]_C18:1		[TG(54:5)]_C18:2	70373.66449	38296.81921	0.023889221
[TG(48:3)]_C14:0		CE(18:3)K	3904.340287	2153.880152	0.024441602
SM(d16:0/18:0)		DG(39:7)_C18:1	7459.004502	5764.454414	0.027552487
[TG(54:9),TG(53:2)]_C18:1		PC(36:4),PC(O-37:4)	486896.8339	273252.0431	0.028463657
[TG(54:7)]_C20:4		CE(19:0)K	3545.704259	1982.72082	0.028739952
PC(34:0),PC(O-35:0)		DG(O-40:9),DG(38:2)_C18:1	3744.044254	3237.554218	0.028870965
[TG(54:11),TG(53:4)]_C18:1		DG(32:1)_C16:0	3566.220251	2360.670168	0.03119988
SM(d18:0/15:0)		DG(38:5)_C16:0	4042.032301	3506.762246	0.033311768
PC(40:5)		DG(O-38:9),DG(36:2)_C18:0	3647.904238	2469.970842	0.036063487
DG(34:3)_C16:0		CE(18:2) NH4	1179381.418	902259.5537	0.038449362
[TG(53:8),TG(52:1)]_C16:0		CE(22:6)H	3109.944217	2713.157521	0.040286543
PC(34:1),PC(O-35:1),PC(P-35:0)		CE(20:0)H	3113.116213	2837.249529	0.041381818
LPC(22:6)		CE(14:0) NH4	4024.612282	2716.688854	0.041402446
SM(d17:1/24:1)		DG(39:8),DG(O-40:8)_C18:2	40939.56292	31793.24218	0.041774061
[TG(51:8),TG(50:1)]_C14:0		CE(19:0)H	231770.1843	175693.5259	0.043402711
FA(6:0)		CE(22:4)K	2865.956213	1691.166122	0.047149314
[TG(56:7)]_C22:6		DG(O-40:9),DG(38:2)_C18:2	1043488.794	811135.5157	0.055265226
DG(36:4),DG(O-37:4)_C18:1		CE(22:4) NH4	2795.548207	1685.298123	0.056955835
CE(17:0)Na		DG(40:5)_C18:0	3322.840224	2268.286817	0.057081911
[TG(48:3)]_C16:1		[TG(56:11),TG(55:4)]_C18:1	2132.212154	1277.182092	0.060874692
[TG(59:10),TG(58:3)]_C18:2		SM(d16:0/23:0)	12386.31284	8686.392595	0.063952818
[TG(54:7)]_C18:2		[TG(53:10),TG(52:3)]_C16:0	205668.9347	123327.1622	0.064476972
SM(d17:1/26:1)		DG(34:4),DG(dO-36:4)_C16:1	3719.864262	3252.180889	0.06854316
[TG(54:6)]_C18:3		CE(20:2)Na	3856.440266	3474.366907	0.069520818
DG(40:8),DG(39:1)_C18:1		CE(22:6) NH4	14845.93309	12247.28621	0.073002921
PC(35:3),PC(O-36:3),PC(P-36:2)		DG(34:3)_C16:1	2549.572193	1595.905448	0.082063952
[TG(48:4)]_C18:2		CE(20:2) NH4	3262.420239	2358.390168	0.084244608
[TG(55:9),TG(54:2)]_C20:0		PC(33:1),PC(O-34:1),PC(P-34:0)	16044.19708	10050.35999	0.084960977
[TG(57:9),TG(56:2)]_C18:0		CE(20:3)Na	3187.184219	2295.287498	0.088840308
PC(38:4)		SM(d18:1/19:0)	15719.50518	9850.291447	0.088892125
FA(10:2)		PC(33:2),PC(O-34:2),PC(P-34:1)	20388.30546	12763.86155	0.090654712
[TG(57:10),TG(56:3)]_C20:0		PC(41:7),PC(P-42:6),PC(40:0),PC(O-41:0)	2916.640211	1848.493466	0.096070926
[TG(62:16),TG(61:9),TG(60:2)]_C18:1		[TG(55:10),TG(54:3)]_C18:1	116710.6511	84056.99682	0.097702492
DG(44:7),DG(43:0)_C16:0		CE(17:1) NH4	3291.448239	2392.811498	0.098488404
PC(O-40:9),PC(38:2),PC(P-39:1)		CE(20:1)K	2810.3882	1790.468128	0.099991861
SM(d16:1/20:1)		CE(20:5)K	3024.640218	2234.966829	0.109699652
[TG(51:7)]_C18:1		CE(15:0)K	2475.472179	1614.172116	0.120188271
[TG(48:2)]_C18:1		DG(37:6)_C16:0	2885.624203	1904.054132	0.141419297
SM(d16:1/20:0)		CE(22:5)H	3883.728267	3579.131588	0.144641001
[TG(51:9),TG(50:2)]_C14:0		[TG(53:10),TG(52:3)]_C18:1	194985.5681	145699.2664	0.148778975
SM(d18:2/24:1)		PC(34:3),PC(P-35:2)	21971.31764	20528.17751	0.149684185
[TG(57:9),TG(56:2)]_C18:1		PC(38:6)	110997.5005	83817.54168	0.150372076
PC(37:4),PC(O-38:4),PC(P-38:3)		PC(39:5),PC(O-40:5),PC(P-40:4)	19038.99726	14423.01037	0.158343053
CE(24:1)H		[TG(52:4)]_C16:0	68329.00024	51719.18642	0.158746106
SM(d18:0/24:0)		[TG(55:10),TG(54:3)]_C18:2	21947.2374	16563.03369	0.158856756
[TG(50:3)]_C18:1		DG(36:7),DG(35:0)_C18:0	3132.352221	2390.12884	0.158917151
[TG(52:10),TG(51:3)]_C18:2		[TG(52:4)]_C18:1	21149.78558	16080.48859	0.169503817
SM(d16:0/24:0)		PC(39:7),PC(P-40:6),PC(38:0),PC(O-39:0)	7260.016536	5523.843065	0.17035891
[TG(48:3)]_C18:1		DG(37:7)_C16:1	2803.900198	1914.136803	0.178212215
DG(44:8),DG(43:1)_C16:0		DG(40:9),DG(39:2)_C18:2	3639.860254	3384.170241	0.205759419
CE(24:1) NH4		DG(34:2)_C18:1	3221.720227	2517.583515	0.212600279
[TG(54:5)]_C20:4		SM(d16:1/22:1)	31929.63441	25350.20042	0.24102655
[TG(46:2)]_C14:0		[TG(52:5)]_C18:2	12336.47678	9801.120004	0.264469995
PC(40:7),PC(39:0),PC(O-40:0)		PC(32:2),PC(O-33:2),PC(P-33:1)	23660.43763	18997.47729	0.272281948
PC(42:11),PC(41:4),PC(O-42:4)		PC(35:2),PC(O-36:2),PC(P-36:1)	24436.70586	19814.58213	0.28443538
[TG(46:0)]_C16:0		PE(34:2),PE(O-35:2),PE(P-35:1)	3032.436211	2465.772839	0.285889468
[TG(46:3)]_C18:2		PC(34:2),PC(O-35:2),PC(P-35:1)	934686.2254	883243.4981	0.290824072
PC(38:5)		[TG(53:8),TG(52:1)]_C18:1	37394.97846	27490.64112	0.296586644
PC(38:3)		DG(34:3)_C18:2	2646.964189	2163.424153	0.307228136
SM(d16:1/17:0)		DG(34:2)_C16:1	4200.176316	2848.636206	0.316478565

CE(17:0) NH4		[TG(55:11),TG(54:4)]_C18:1	96845.03498	65732.78	0.320095307
[TG(48:3)]_C16:0		[TG(55:9),TG(54:2)]_C18:0	19640.22936	16079.9025	0.324744165
[TG(50:3)]_C18:2		CE(20:2)K	4891.140321	4580.520329	0.330008163
[TG(52:8),TG(51:1)]_C18:1		[TG(56:6)]_C22:5	2649.132173	2176.088822	0.3324798
[TG(57:9),TG(56:2)]_C20:0		DG(40:2)_C18:2	2433.924162	2021.327479	0.349123258
[TG(50:4)]_C14:0		[TG(55:8),TG(54:1)]_C18:0	3598.384256	5265.517713	0.371527484
[TG(54:5)]_C16:0		[TG(56:6)]_C18:2	2983.048234	2473.77485	0.375060393
PC(O-38:8),PC(36:1),PC(O-37:1),PC(P-37:0)		[TG(52:4)]_C18:2	103095.2355	86441.80679	0.39201346
709.686225 -> 369.2		FA(14:2)	16941.74512	12285.40154	0.426476798
PC(32:0),PC(O-33:0)		[TG(53:9),TG(52:2)]_C18:2	26191.55379	22223.6802	0.4292372
[TG(52:5)]_C20:4		[TG(56:7),TG(55:0)]_C16:0	2124.20416	1827.157462	0.450176064
[TG(46:2)]_C16:1		FA(15:1)	17132.60924	12784.13228	0.463019508
[TG(53:9),TG(52:2)]_C18:1		[TG(55:9),TG(54:2)]_C18:1	27316.45006	20871.77279	0.488391204
DG(44:9),DG(43:2)_C18:2		DG(40:5)_C16:0	3652.412276	3543.458913	0.489397885
DG(32:1)_C16:1		[TG(56:7)]_C22:5	1345.624091	1775.600795	0.500291227
PC(42:3)		[TG(52:4)]_C16:1	7699.224627	10129.21477	0.500413312
PC(36:5)		DG(42:5)_C18:0	2719.752187	2658.328855	0.504142001
SM(d16:1/18:1)		SM(d18:2/18:1)	3529.036234	3450.699572	0.619610465
[TG(54:6)]_C16:0		[TG(53:10),TG(52:3)]_C18:2	149867.0301	129865.6822	0.647524174
DG(31:1)_C16:0		[TG(55:8),TG(54:1)]_C18:1	4962.860368	4511.963649	0.654889774
[TG(51:8),TG(50:1)]_C16:0		PE(38:4)	4501.060343	4371.126951	0.676733634
[TG(56:10),TG(55:3)]_C18:1		[TG(57:12),TG(56:5)]_C20:4	2509.196178	2132.112827	0.685610204
CE(22:6)K		[TG(53:8),TG(52:1)]_C18:0	21786.48141	23948.85815	0.766734306
[TG(54:6)]_C20:4		SM(d16:1/22:0)	330080.6392	302224.8774	0.77550814
SM(d18:2/22:1)		PC(33:3),PC(O-34:3),PC(P-34:2)	11061.47275	10359.26604	0.830584045
[TG(51:9),TG(50:2)]_C18:2		[TG(53:10),TG(52:3)]_C16:1	8092.356538	7671.546571	0.861847155
PC(40:10),PC(39:3),PC(O-40:3),PC(P-40:2)		[TG(52:5)]_C16:1	3496.196278	3370.172912	0.905268713
[TG(50:4)]_C18:2		[TG(56:8)]_C22:6	2363.792157	2383.392164	0.907757124
[TG(48:2)]_C18:2		[TG(53:9),TG(52:2)]_C18:0	16611.56527	17125.50849	0.921997201
PC(38:8),PC(37:1),PC(O-38:1),PC(P-38:0)		SM(d18:2/20:1)	18882.1533	18589.45661	0.974937577
CE(20:0)K		SM(d18:0/26:1(17Z))	1770.076135	1748.729455	0.980520135
DG(30:1)_C18:1		PE(38:6)	1314.684098	1301.910762	0.984335945
SM(d18:1/17:0)					
DG(38:7),DG(37:0)_C16:0					
[TG(50:3)]_C16:0					
[TG(54:7)]_C18:3					
PC(38:7),PC(37:0),PC(O-38:0)					
PC(42:4)					
[TG(55:10),TG(54:3)]_C16:0					
[TG(52:5)]_C18:1					
SM(d18:2/21:0)					
SM(d16:0/20:0)					
PC(40:4)					
[TG(50:4)]_C16:0					
DG(32:1)_C18:1					
[TG(38:0)]_C20:0					
[TG(49:3)]_C18:2					
PI(34:2),PI(O-35:2),PI(P-35:1)					
PC(35:4),PC(O-36:4),PC(P-36:3)					
CE(22:6)Na					
PC(40:2)					
PC(40:3)					
PC(28:1),PC(P-29:0)					
PC(38:9),PC(37:2),PC(O-38:2),PC(P-38:1)					
PC(36:8),PC(35:1),PC(O-36:1),PC(P-36:0)					
[TG(57:10),TG(56:3)]_C18:2					
[TG(46:2)]_C18:2					
PC(37:7),PC(P-38:6),PC(36:0),PC(O-37:0)					
SM(d18:1/24:1(15Z))					
[TG(54:5)]_C18:1					
DG(42:8),DG(41:1)_C18:1					
SM(d18:1/26:1(17Z))					
PC(41:5),PC(P-42:4)					
PC(30:1),PC(O-31:1),PC(P-31:0)					
[TG(51:9),TG(50:2)]_C16:0					

PC(44:2)					
[TG(50:9),TG(49:2)]_C18:2					
CE(20:1)H					
[TG(46:3)]_C16:1					
PE(O-38:9),PE(36:2),PE(O-37:2),PE(P-37:1)					
DG(40:6),DG(dO-40:0)_C16:0					
[TG(54:10),TG(53:3)]_C18:2					
PC(37:5),PC(O-38:5),PC(P-38:4)					
[TG(49:7),TG(48:0)]_C16:0					
CE(16:2) NH4					
LPC(18:1),PC(O-18:1),PC(P-18:0)					
[TG(49:7),TG(48:0)]_C18:0					
[TG(50:5)]_C18:2					
PC(14:0),LPC(15:0),LPC(O-16:0)					
DG(32:2)_C18:2					
SM(d18:1/25:0)					
PI(36:1),PI(O-37:1),PI(P-37:0)					
[TG(50:3)]_C18:3					
SM(d16:0/22:0)					
[TG(48:8),TG(47:1)]_C16:0					
[TG(57:11),TG(56:4)]_C18:2					
[TG(48:3)]_C18:3					
SM(d16:1/24:1)					
CE(16:1)K					
PC(31:1),PC(O-32:1),PC(P-32:0)					
[TG(51:8),TG(50:1)]_C18:1					
[TG(51:9),TG(50:2)]_C18:0					
[TG(51:7),TG(50:0)]_C18:0					
PC(40:6)					
[TG(52:9),TG(51:2)]_C18:1					
PC(40:8),PC(39:1),PC(O-40:1),PC(P-40:0)					
SM(d18:0/24:1)					
SM(d16:1/18:0)					
[TG(52:9),TG(51:2)]_C16:0					
PC(37:3),PC(O-38:3),PC(P-38:2)					
DG(44:8),DG(43:1)_C18:1					
[TG(48:2)]_C16:1					
[TG(53:9),TG(52:2)]_C16:0					
CE(22:0)K					
PE(36:3),PE(P-37:2)					
PC(30:2),PC(P-31:1)					
PC(40:9),PC(39:2),PC(O-40:2),PC(P-40:1)					
[TG(51:9),TG(50:2)]_C18:1					
[TG(50:3)]_C16:1					
[TG(53:9),TG(52:2)]_C16:1					
[TG(56:7)]_C20:4					
[TG(57:10),TG(56:3)]_C18:1					
