# Supplementary material for: Elucidating the Influence of Serum Concentration, Sex, and Particle Size on Iron Oxide Nanoparticle–Lipid Biocorona Formation
Source: Nanomaterials (Basel). 2026 Jun 1;16(11):683. doi: 10.3390/nano16110683 (PMC13258708; doi:10.3390/nano16110683)
Supplement: Supplementary file 1 [file nanomaterials-16-00683-s001.zip › nanomaterials-4334647-supplementary - 副本/Table S8. Comparison of Lipid Corona Profiles Between Nanoparticle Sizes.pdf]

Table S8. Comparison of Lipid Corona Profiles Between Nanoparticle Sizes
Male 5% BC Samples

Unique Lipids in 50 nm BC	Unique Lipds in 100 nm BC	Shared Lipids
[TG(53:9),TG(52:2)]_C18:0	DG(34:2)_C18:2	FA(17:2)
[TG(54:6)]_C18:2	DG(40:5)_C18:0	FA(22:7)
[TG(53:7),TG(52:0)]_C16:0	CE(18:1) NH4	FA(19:2)
[TG(50:3)]_C18:2	DG(39:8),DG(O-40:8)_C18:2	PG(16:0),LPG(17:0),LPG(O-18:0); PG(16:0),LPG(17:0),LPG(O-18:0)
DG(30:2)_C16:1	DG(38:5)_C16:0	
[TG(49:6)]_C16:0	CE(15:1)K	
[TG(53:7)]_C18:1	CE(18:0) NH4	
[TG(51:8),TG(50:1)]_C18:0	CE(18:3)H	
[TG(52:4)]_C16:1	DG(30:0)_C16:0	
[TG(54:5)]_C18:3	DG(O-38:8),DG(36:1)_C16:1	
[TG(52:4)]_C18:3	CE(20:4) NH4	
[TG(51:7),TG(50:0)]_C16:0	DG(36:3)_C18:1	
FA(35:0)	DG(33:0)_C16:0	
DG(36:8),DG(35:1)_C16:1	CE(18:2)Na	
PC(32:0),PC(O-33:0)	CE(22:2) NH4	
[TG(53:9),TG(52:2)]_C18:1	CE(16:0) NH4	
[TG(55:9),TG(54:2)]_C18:1	DG(O-38:8),DG(36:1)_C18:0	
[TG(48:2)]_C14:0	CE(20:3) NH4	
[TG(51:9),TG(50:2)]_C16:1	DG(O-38:8),DG(36:1)_C18:1	
DG(36:7),DG(35:0)_C16:0	DG(32:1)_C16:0	
[TG(52:4)]_C18:1	CE(18:3)Na	
[TG(49:8),TG(48:1)]_C14:0	[TG(38:0)]_C20:0	
[TG(51:8),TG(50:1)]_C16:0	DG(34:0)_C16:0	
[TG(55:9),TG(54:2)]_C18:0	DG(40:5)_C16:0	
[TG(53:10),TG(52:3)]_C18:2	CE(20:5) NH4	
SM(d18:2/22:1)	CE(22:5) NH4	
[TG(51:9),TG(50:2)]_C18:2	DG(34:4),DG(dO-36:4)_C16:1	
[TG(53:7),TG(52:0)]_C18:0	DG(39:8),DG(O-40:8),DG(38:1)_C18:1	
[TG(52:5)]_C18:3	CE(18:1)Na	
[TG(50:4)]_C18:2	DG(37:7),DG(36:0)_C16:0	
[TG(48:2)]_C18:2	DG(35:6)_C18:0	
DG(41:5)_C16:0	DG(32:0)_C16:0	
[TG(49:8),TG(48:1)]_C16:0	DG(39:7),DG(38:0),DG(dO-40:0)_C18:0	
[TG(52:4)]_C18:2	CE(18:2) NH4	
[TG(55:10),TG(54:3)]_C18:0	CE(18:0)K	
[TG(50:3)]_C16:0	DG(34:0)_C18:0	
[TG(53:10),TG(52:3)]_C18:1	CE(22:1)H	
[TG(54:8),TG(53:1)]_C18:1	CE(16:0)Na	
Cer(d14:2(4E,6E)/16:0)	CE(19:0)H	
[TG(53:8),TG(52:1)]_C18:1	DG(40:6),DG(dO-40:0)_C16:0	
DG(42:11),DG(41:4)_C16:0	DG(42:6)_C16:0	
[TG(55:10),TG(54:3)]_C18:2	CE(18:3) NH4	
[TG(55:8),TG(54:1)]_C18:1	CE(20:5)H	
[TG(48:2)]_C16:0	DG(32:0)_C18:0	
FA(18:3)	CE(20:2)Na	
[TG(55:8),TG(54:1)]_C18:0	DG(37:7),DG(36:0)_C18:0	
[TG(52:4)]_C16:0	FA(6:0)	
PC(36:3),PC(P-37:2)	DG(37:6)_C18:0	
LPC(18:0),PC(O-18:0),LPC(O-19:0)	DG(34:1)_C16:0	
DG(36:7)_C16:1	CE(16:1) NH4	
PS(25:0)	DG(35:6)_C16:0	
DG(30:3)_C16:1	FA(26:1)	
[TG(39:0)]_C20:0	LPG(19:0),LPG(O-20:0); LPG(19:0),LPG(O-20:0)	
[TG(55:11),TG(54:4)]_C18:2	CE(22:6) NH4	
[TG(54:6)]_C18:1	CE(16:0)K	
LPG(20:0); LPG(20:0)	DG(34:1)_C18:1	
[TG(54:5)]_C18:1	DG(42:5)_C18:0	
[TG(50:3)]_C14:0	CE(20:2)K	
[TG(49:8),TG(48:1)]_C18:1	DG(O-38:9),DG(36:2)_C18:1	
CAR(20:0)	DG(36:7),DG(35:0)_C18:0	
[TG(51:9),TG(50:2)]_C16:0	FA(28:3)	
[TG(53:9),TG(52:2)]_C18:2	DG(O-40:9),DG(38:2)_C18:2	
[TG(54:9),TG(53:2)]_C18:1		
PC(34:0),PC(O-35:0)		
FA(22:1)		
[TG(53:8),TG(52:1)]_C16:0		
Cer(d18:0/17:0)		
[TG(55:10),TG(54:3)]_C18:1		
[TG(49:7),TG(48:0)]_C16:0		
PC(34:1),PC(O-35:1),PC(P-35:0)		
[TG(49:7),TG(48:0)]_C18:0		
DG(41:6)_C16:1		
PC(36:4),PC(O-37:4)		
SM(d16:0/22:0)		
PC(34:2),PC(O-35:2),PC(P-35:1)		
[TG(44:1)]_C16:0		
[TG(46:2)]_C18:1		
[TG(49:7),TG(48:0)]_C14:0		

CE(15:1) NH4		
[TG(51:8),TG(50:1)]_C18:1		
FA(21:0)		
PC(40:6)		
[TG(51:7),TG(50:0)]_C18:0		
[TG(50:7),TG(49:0)]_C16:0		
[TG(54:5)]_C18:2		
[TG(52:9),TG(51:2)]_C18:1		
[TG(53:8),TG(52:1)]_C18:0		
[TG(51:7)]_C18:1		
[TG(53:10),TG(52:3)]_C16:0		
[TG(50:7),TG(49:0)]_C18:0		
[TG(48:2)]_C18:1		
PC(38:6)		
[TG(51:9),TG(50:2)]_C14:0		
FA(22:0)		
[TG(52:5)]_C18:2		
[TG(48:2)]_C16:1		
[TG(50:3)]_C18:1		
[TG(53:9),TG(52:2)]_C16:0		
PS(O-29:0)		
DG(30:2)_C16:0		
CAR(14:2)		
[TG(51:9),TG(50:2)]_C18:1		
[TG(50:3)]_C16:1		
DG(36:6)_C16:0		
[TG(54:5)]_C20:4		
[TG(53:10),TG(52:3)]_C16:1		
[TG(46:0)]_C14:0		
[TG(55:11),TG(54:4)]_C18:1		
[TG(46:0)]_C16:0		
FA(20:0)		
[TG(55:11),TG(54:4)]_C18:0		
PC(38:5)		

Table S8. Comparison of Lipid Corona Profiles Between Nanoparticle Sizes
Male 10% BC Samples

Unique Lipids in 50 nm BC	Unique Lipds in 100 nm BC	Shared Lipids
PC(38:3)	CE(20:5)Na	
[TG(46:1)]_C14:0	CE(20:0) NH4	
SM(d16:1/17:0)	DG(34:2)_C18:2	
[TG(53:9),TG(52:2)]_C18:0	DG(40:5)_C18:0	
[TG(54:6)]_C18:2	CE(18:1) NH4	
[TG(48:3)]_C16:0	DG(39:8),DG(O-40:8)_C18:2	
FA(28:6)	DG(38:5)_C16:0	
[TG(53:7),TG(52:0)]_C16:0	CE(15:1)K	
[TG(50:3)]_C18:2	CE(18:0) NH4	
[TG(57:12),TG(56:5)]_C18:1	CE(18:3)H	
PC(35:2),PC(O-36:2),PC(P-36:1)	DG(30:0)_C16:0	
[TG(49:6)]_C16:0	DG(O-38:8),DG(36:1)_C16:1	
[TG(53:7)]_C18:1	CE(18:2)K	
[TG(51:8),TG(50:1)]_C18:0	CE(20:4) NH4	
[TG(50:4)]_C14:0	DG(36:3)_C18:1	
[TG(52:4)]_C16:1	DG(33:0)_C16:0	
[TG(42:0)]_C16:0	CE(18:2)Na	
[TG(54:5)]_C18:3	CE(22:2) NH4	
[TG(52:4)]_C18:3	CE(16:0) NH4	
PC(O-38:8),PC(36:1),PC(O-37:1),PC(P-37:0)	DG(O-38:8),DG(36:1)_C18:0	
SM(d16:1/16:0)	CE(20:3) NH4	
[TG(55:11),TG(54:4)]_C16:0	DG(O-38:8),DG(36:1)_C18:1	
[TG(55:9),TG(54:2)]_C16:0	DG(32:1)_C16:0	
[TG(51:7),TG(50:0)]_C16:0	DG(39:7)_C18:1	
FA(35:0)	CE(18:3)Na	
DG(36:8),DG(35:1)_C16:1	[TG(38:0)]_C20:0	
PC(O-38:9),PC(36:2),PC(O-37:2),PC(P-37:1)	[TG(44:5)]_C20:0	
PC(32:0),PC(O-33:0)	DG(34:0)_C16:0	
[TG(53:9),TG(52:2)]_C18:1	DG(40:5)_C16:0	
[TG(52:8),TG(51:1)]_C16:0	CE(20:4)H	
[TG(55:9),TG(54:2)]_C18:1	CE(20:5) NH4	
[TG(49:8),TG(48:1)]_C18:0	CE(22:5) NH4	
[TG(48:2)]_C14:0	DG(34:4),DG(dO-36:4)_C16:1	
[TG(52:4)]_C20:4	DG(39:8),DG(O-40:8),DG(38:1)_C18:1	
[TG(51:9),TG(50:2)]_C16:1	CE(18:1)Na	
DG(36:7),DG(35:0)_C16:0	DG(37:7),DG(36:0)_C16:0	
PC(36:5)	DG(35:6)_C18:0	
[TG(52:4)]_C18:1	CE(16:2)Na	
PC(30:0),PC(O-31:0)	DG(32:0)_C16:0	
[TG(49:8),TG(48:1)]_C14:0	DG(39:7),DG(38:0),DG(dO-40:0)_C18:0	
[TG(46:1)]_C18:1	CE(18:2) NH4	
[TG(51:8),TG(50:1)]_C16:0	CE(18:0)K	
[TG(55:9),TG(54:2)]_C18:0	CE(22:4)Na	
SM(d16:1/24:0)	DG(34:0)_C18:0	
[TG(54:10),TG(53:3)]_C18:1	CE(22:1)H	
CAR(18:3)	CE(16:0)Na	
[TG(53:10),TG(52:3)]_C18:2	CE(19:0)H	
LPC(20:2),PC(O-20:2)	DG(40:6),DG(dO-40:0)_C16:0	
[TG(54:6)]_C20:4	CE(18:3) NH4	
SM(d18:2/22:1)	DG(34:2)_C16:0	
[TG(53:7),TG(52:0)]_C18:0	CE(20:5)H	
[TG(51:9),TG(50:2)]_C18:2	DG(32:0)_C18:0	
[TG(52:5)]_C16:0	DG(36:3)_C18:2	
[TG(52:5)]_C18:3	DG(37:7),DG(36:0)_C18:0	
[TG(50:4)]_C18:2	DG(37:6)_C18:0	
[TG(48:2)]_C18:2	DG(34:1)_C16:0	
[TG(56:6)]_C20:4	CE(16:1)Na	
[TG(49:8),TG(48:1)]_C16:1	DG(37:6)_C16:0	
DG(41:5)_C16:0	CE(16:1) NH4	
[TG(49:8),TG(48:1)]_C16:0	DG(35:6)_C16:0	
[TG(52:4)]_C18:2	CE(16:3)Na	
[TG(55:10),TG(54:3)]_C18:0	DG(36:4),DG(O-37:4)_C18:2	
[TG(50:3)]_C16:0	CE(22:6) NH4	
[TG(53:10),TG(52:3)]_C18:1	CE(16:0)K	
CAR(14:1)	DG(34:1)_C18:1	
Cer(d14:2(4E,6E)/16:0)	DG(42:5)_C18:0	
[TG(53:8),TG(52:1)]_C18:1	CE(20:2)K	
PC(29:1),PC(O-30:1),PC(P-30:0)	DG(36:7),DG(35:0)_C18:0	
DG(42:11),DG(41:4)_C16:0	DG(O-38:9),DG(36:2)_C18:1	
PC(28:0),PC(O-29:0)	CE(19:0)Na	
[TG(48:3)]_C18:2	DG(O-40:9),DG(38:2)_C18:2	
[TG(55:10),TG(54:3)]_C18:2		
[TG(55:8),TG(54:1)]_C18:1		
[TG(48:2)]_C16:0		
[TG(55:8),TG(54:1)]_C18:0		
PG(20:0),LPG(21:0); PG(20:0),LPG(21:0)		
SM(d16:0/20:0)		
[TG(52:4)]_C16:0		

PC(40:4)		
[TG(50:4)]_C16:0		
[TG(46:1)]_C16:0		
PC(36:3),PC(P-37:2)		
PC(35:4),PC(O-36:4),PC(P-36:3)		
[TG(50:8),TG(49:1)]_C16:0		
[TG(50:4)]_C16:1		
LPC(18:0),PC(O-18:0),LPC(O-19:0)		
DG(36:7)_C16:1		
DG(30:1)_C16:0		
PC(28:1),PC(P-29:0)		
[TG(44:0),TG(O-45:0)]_C16:0		
DG(30:3)_C16:1		
[TG(50:8),TG(49:1)]_C18:1		
[TG(53:10),TG(52:3)]_C18:0		
[TG(39:0)]_C20:0		
[TG(46:2)]_C18:2		
[TG(55:11),TG(54:4)]_C18:2		
[TG(54:6)]_C18:1		
PC(37:7),PC(P-38:6),PC(36:0),PC(O-37:0)		
SM(d16:1/22:0)		
LPG(20:0); LPG(20:0)		
[TG(54:5)]_C18:1		
[TG(50:3)]_C14:0		
[TG(49:8),TG(48:1)]_C18:1		
PC(30:1),PC(O-31:1),PC(P-31:0)		
[TG(51:9),TG(50:2)]_C16:0		
[TG(53:9),TG(52:2)]_C18:2		
SM(d16:0/18:0)		
[TG(51:8),TG(50:1)]_C16:1		
[TG(54:9),TG(53:2)]_C18:1		
PC(34:0),PC(O-35:0)		
PC(40:5)		
FA(22:1)		
Cer(d18:0/17:0)		
[TG(53:8),TG(52:1)]_C16:0		
[TG(55:10),TG(54:3)]_C18:1		
CAR(10:2)		
PC(37:5),PC(O-38:5),PC(P-38:4)		
PC(34:1),PC(O-35:1),PC(P-35:0)		
[TG(49:7),TG(48:0)]_C16:0		
[TG(49:7),TG(48:0)]_C18:0		
PC(36:4),PC(O-37:4)		
[TG(48:3)]_C16:1		
[TG(54:7)]_C18:2		
[TG(50:3)]_C18:3		
SM(d16:0/22:0)		
[TG(57:11),TG(56:4)]_C18:2		
PC(34:2),PC(O-35:2),PC(P-35:1)		
[TG(44:1)]_C16:0		
[TG(54:6)]_C18:3		
[TG(53:8)]_C18:2		
[TG(46:2)]_C18:1		
CE(15:1) NH4		
[TG(49:7),TG(48:0)]_C14:0		
[TG(52:5)]_C16:1		
PC(35:3),PC(O-36:3),PC(P-36:2)		
SM(d16:1/24:1)		
PC(31:1),PC(O-32:1),PC(P-32:0)		
[TG(51:8),TG(50:1)]_C18:1		
PC(38:4)		
[TG(50:7),TG(49:0)]_C16:0		
PC(40:6)		
[TG(51:7),TG(50:0)]_C18:0		
[TG(54:5)]_C18:2		
LPG(19:0),LPG(O-20:0); LPG(19:0),LPG(O-20:0)		
[TG(52:9),TG(51:2)]_C18:1		
[TG(51:7)]_C18:1		
[TG(53:8),TG(52:1)]_C18:0		
[TG(53:10),TG(52:3)]_C16:0		
[TG(48:2)]_C18:1		
PC(38:6)		
[TG(55:9),TG(54:2)]_C18:2		
SM(d16:1/18:0)		
SM(d16:1/20:0)		
[TG(52:9),TG(51:2)]_C16:0		
[TG(51:9),TG(50:2)]_C14:0		
[TG(48:2)]_C16:1		
[TG(52:5)]_C18:2		
[TG(50:3)]_C18:1		
PS(O-29:0)		
[TG(53:9),TG(52:2)]_C16:0		

[TG(52:10),TG(51:3)]_C18:2		
DG(30:2)_C16:0		
[TG(46:1)]_C16:1		
PC(30:2),PC(P-31:1)		
CAR(14:2)		
[TG(51:9),TG(50:2)]_C18:1		
[TG(53:9),TG(52:2)]_C16:1		
[TG(50:3)]_C16:1		
DG(36:6)_C16:0		
[TG(56:7)]_C20:4		
[TG(54:5)]_C20:4		
[TG(46:0)]_C14:0		
[TG(53:10),TG(52:3)]_C16:1		
DG(36:5)_C16:0		
[TG(55:11),TG(54:4)]_C18:1		
[TG(46:0)]_C16:0		
[TG(55:11),TG(54:4)]_C18:0		
PG(16:0),LPG(17:0),LPG(O-18:0); PG(16:0),LPG(17:0),LPG(O-18:0)		
PC(38:5)		

Table S8. Comparison of Lipid Corona Profiles Between Nanoparticle Sizes
Male 25% BC Samples

Unique Lipids in 50 nm BC	Unique Lipds in 100 nm BC	Shared Lipids
[TG(46:2)]_C16:0	CE(20:5)Na	CE(20:2)Na
SM(d18:0/17:0)	DG(34:2)_C18:2	CE(20:0) NH4
[TG(54:5)]_C18:0	CE(18:1) NH4	Cer(d14:2(4E,6E)/16:0)
[TG(54:6)]_C18:2	DG(39:8),DG(O-40:8)_C18:2	[TG(49:7),TG(48:0)]_C16:0
FA(28:6)	CE(15:1)K	CE(20:1) NH4
[TG(54:11),TG(53:4)]_C18:2	CE(18:3)H	CE(16:0)K
SM(d18:0/26:1(17Z))	DG(30:0)_C16:0	
PC(33:2),PC(O-34:2),PC(P-34:1)	DG(32:2)_C18:1	
[TG(53:7),TG(52:0)]_C16:0	DG(36:3)_C18:1	
[TG(57:12),TG(56:5)]_C18:1	CE(18:2)Na	
PC(35:2),PC(O-36:2),PC(P-36:1)	CE(16:0) NH4	
[TG(56:12),TG(55:5)]_C18:1	CE(20:3) NH4	
PC(39:8),PC(O-40:8),PC(38:1),PC(O-39:1),PC(P-39:0)	DG(O-38:8),DG(36:1)_C18:1	
[TG(57:12),TG(56:5)]_C16:0	DG(32:5)_C18:1	
LPI(20:0)	DG(32:1)_C16:0	
[TG(52:4)]_C16:1	CE(22:1) NH4	
[TG(53:7),TG(52:0)]_C20:0	DG(39:7)_C18:1	
[TG(54:5)]_C18:3	DG(O-38:9),DG(36:2)_C18:2	
[TG(52:4)]_C18:3	CE(18:3)Na	
[TG(50:9),TG(49:2)]_C16:0	DG(34:0)_C16:0	
[TG(44:1)]_C18:1	CE(20:2) NH4	
SM(d16:1/16:0)	DG(40:5)_C16:0	
PC(39:4),PC(O-40:4),PC(P-40:3)	Cer(d18:0/21:0)	
[TG(50:9),TG(49:2)]_C18:1	CE(20:5) NH4	
[TG(51:7),TG(50:0)]_C16:0	CE(22:5) NH4	
FA(35:0)	CE(22:3)H	
PC(O-38:9),PC(36:2),PC(O-37:2),PC(P-37:1)	DG(37:7),DG(36:0)_C16:0	
[TG(52:8),TG(51:1)]_C16:0	DG(34:2)_C18:1	
PC(35:5),PC(O-36:5),PC(P-36:4)	CE(20:5)K	
[TG(57:11),TG(56:4)]_C18:1	DG(32:0)_C16:0	
LPC(16:0),PC(O-16:0),LPC(O-17:0)	CE(14:0) NH4	
[TG(46:0)]_C18:0	CE(20:0)H	
[TG(48:2)]_C14:0	DG(39:7),DG(38:0),DG(dO-40:0)_C18:0	
[TG(52:4)]_C20:4	CE(18:0)K	
[TG(54:10),TG(53:3)]_C16:0	CE(20:3)Na	
[TG(51:9),TG(50:2)]_C16:1	DG(34:0)_C18:0	
[TG(52:4)]_C18:1	CE(16:0)Na	
[TG(48:8),TG(47:1)]_C18:1	CE(18:3) NH4	
[TG(49:8),TG(48:1)]_C14:0	DG(32:0)_C18:0	
PC(30:0),PC(O-31:0)	FA(14:2)	
[TG(57:12),TG(56:5)]_C20:4	FA(6:0)	
PC(40:1),PC(P-41:0)	DG(37:6)_C18:0	
[TG(59:9),TG(58:2)]_C18:1	FA(15:1)	
[TG(46:1)]_C18:1	DG(34:1)_C16:0	
[TG(55:9),TG(54:2)]_C18:0	CE(16:1)Na	
SM(d16:1/24:0)	DG(37:6)_C16:0	
[TG(57:9),TG(56:2)]_C16:0	CE(16:1) NH4	
[TG(54:10),TG(53:3)]_C18:1	DG(44:7),DG(43:0)_C16:0	
[TG(52:6)]_C18:2	CE(16:3)Na	
[TG(53:10),TG(52:3)]_C18:2	DG(O-40:9),DG(38:2)_C18:1	
[TG(54:11),TG(53:4)]_C16:0	DG(36:4),DG(O-37:4)_C18:2	
PC(36:7),PC(35:0),PC(O-36:0)	CE(22:6) NH4	
[TG(52:6)]_C16:0	DG(O-38:9),DG(36:2)_C18:1	
[TG(44:2)]_C18:2	DG(36:7),DG(35:0)_C18:0	
[TG(50:8),TG(49:1)]_C16:1	CE(22:2)H	
SM(d16:0/25:0)	DG(O-38:9),DG(36:2)_C18:0	
Cer(d18:1/24:0)	DG(O-40:9),DG(38:2)_C18:2	
[TG(53:7),TG(52:0)]_C18:0	CE(18:1)K	
[TG(52:5)]_C16:0	CE(17:0) NH4	
[TG(52:5)]_C18:3	DG(40:5)_C18:0	
LPC(22:4)	CE(19:0)K	
[TG(56:6)]_C20:4	CE(18:3)K	
[TG(49:8),TG(48:1)]_C16:1	CE(22:6)H	
[TG(49:8),TG(48:1)]_C16:0	DG(38:5)_C16:0	
[TG(55:10),TG(54:3)]_C18:0	709.686225 -> 369.2	
[TG(48:7),TG(47:0)]_C16:0	CE(18:0) NH4	
PC(39:6),PC(O-40:6),PC(P-40:5)	DG(38:3)_C18:2	
PI(38:4)	DG(O-38:8),DG(36:1)_C16:1	
[TG(54:8),TG(53:1)]_C18:1	CE(18:2)K	
[TG(52:10),TG(51:3)]_C18:1	CE(20:4) NH4	
[TG(45:0)]_C16:0	DG(33:0)_C16:0	
[TG(53:8),TG(52:1)]_C18:1	DG(34:2)_C16:1	
DG(42:11),DG(41:4)_C16:0	DG(32:1)_C16:1	
[TG(55:10),TG(54:3)]_C18:2	CE(22:2) NH4	
[TG(48:3)]_C18:2	FA(10:3)	
SM(d16:1/25:0)	DG(O-38:8),DG(36:1)_C18:0	
[TG(51:8)]_C18:2	CE(20:0)Na	
[TG(55:8),TG(54:1)]_C18:1	CE(22:3) NH4	

PC(41:6),PC(O-42:6)	DG(40:9),DG(39:2)_C18:2
[TG(48:2)]_C16:0	DG(38:7),DG(37:0)_C16:0
PC(39:7),PC(P-40:6),PC(38:0),PC(O-39:0)	CE(15:0)K
[TG(55:8),TG(54:1)]_C18:0	DG(32:1)_C18:1
[TG(49:7)]_C16:1	[TG(38:0)]_C20:0
CE(22:5)H	CE(20:4)H
DG(36:6)_C16:1	DG(34:4),DG(dO-36:4)_C16:1
SM(d16:1/23:0)	DG(39:8),DG(O-40:8),DG(38:1)_C18:1
[TG(46:1)]_C16:0	CE(18:1)Na
[TG(56:6)]_C18:2	DG(35:6)_C18:0
PC(36:3),PC(P-37:2)	CE(19:0) NH4
SM(d18:2/14:0)	CE(16:2)Na
[TG(50:4)]_C16:1	CE(22:4)K
LPC(18:0),PC(O-18:0),LPC(O-19:0)	CE(18:2) NH4
DG(30:1)_C16:0	CE(22:4)Na
PC(37:6),PC(O-38:6),PC(P-38:5)	CE(22:1)H
[TG(57:9),TG(56:2)]_C18:2	CE(19:0)H
[TG(44:0),TG(O-45:0)]_C16:0	DG(40:6),DG(dO-40:0)_C16:0
[TG(56:11),TG(55:4)]_C18:1	CE(20:5)H
[TG(50:8),TG(49:1)]_C18:1	DG(34:2)_C16:0
PC(32:1),PC(O-33:1),PC(P-33:0)	DG(37:7),DG(36:0)_C18:0
[TG(56:8)]_C20:4	DG(36:3)_C18:2
[TG(53:10),TG(52:3)]_C18:0	CE(20:4)Na
[TG(39:0)]_C20:0	CE(20:1)K
[TG(55:11),TG(54:4)]_C18:2	DG(35:6)_C16:0
[TG(54:6)]_C18:1	DG(34:1)_C18:1
LPG(20:0); LPG(20:0)	DG(42:5)_C18:0
[TG(50:3)]_C14:0	CE(20:2)K
[TG(50:4)]_C18:1	CE(19:0)Na
[TG(49:8),TG(48:1)]_C18:1	
PG(O-35:1),PG(P-35:0); PG(O-35:1),PG(P-35:0)	
[TG(53:9),TG(52:2)]_C18:2	
[TG(49:8)]_C18:2	
[TG(48:3)]_C14:0	
[TG(55:7),TG(54:0)]_C20:0	
[TG(56:7)]_C18:2	
[TG(51:8),TG(50:1)]_C16:1	
SM(d16:0/18:0)	
[TG(54:9),TG(53:2)]_C18:1	
[TG(54:7)]_C20:4	
[TG(61:10),TG(60:3)]_C18:1	
[TG(56:8)]_C22:6	
PC(34:0),PC(O-35:0)	
[TG(44:0),TG(O-45:0)]_C18:0	
[TG(54:11),TG(53:4)]_C18:1	
SM(d18:0/15:0)	
PC(40:5)	
SM(d18:2/18:1)	
FA(30:0)	
[TG(53:8),TG(52:1)]_C16:0	
PC(42:2)	
PC(34:1),PC(O-35:1),PC(P-35:0)	
SM(d17:1/24:1)	
PC(32:2),PC(O-33:2),PC(P-33:1)	
[TG(51:8),TG(50:1)]_C14:0	
SM(d18:1/19:0)	
PC(44:12),PC(O-44:5)	
[TG(56:7)]_C22:6	
[TG(50:4)]_C18:3	
[TG(56:6)]_C16:0	
[TG(59:10),TG(58:3)]_C18:2	
PC(19:1),LPC(20:1),PC(O-20:1),PC(P-20:0)	
[TG(48:3)]_C16:1	
[TG(54:7)]_C18:2	
PC(28:2)	
PC(35:6),PC(P-36:5)	
SM(d17:1/26:1)	
[TG(53:10),TG(52:3)]_C18:3	
[TG(54:6)]_C18:3	
CE(15:1) NH4	
[TG(52:5)]_C16:1	
PC(35:3),PC(O-36:3),PC(P-36:2)	
[TG(48:4)]_C18:2	
[TG(55:9),TG(54:2)]_C20:0	
[TG(52:9),TG(51:2)]_C18:2	
[TG(57:9),TG(56:2)]_C18:0	
PC(38:4)	
[TG(57:10),TG(56:3)]_C20:0	
[TG(50:7),TG(49:0)]_C16:0	
[TG(54:5)]_C18:2	
[TG(44:0),TG(O-45:0)]_C14:0	
PC(O-40:9),PC(38:2),PC(P-39:1)	

SM(d16:1/20:1)
[TG(51:7)]_C18:1
[TG(53:10),TG(52:3)]_C16:0
[TG(48:2)]_C18:1
PC(42:10),PC(41:3),PC(O-42:3),PC(P-42:2)
[TG(44:2)]_C16:0
SM(d16:1/20:0)
[TG(51:9),TG(50:2)]_C14:0
[TG(57:9),TG(56:2)]_C18:1
SM(d18:2/24:1)
PC(42:9),PC(41:2),PC(O-42:2),PC(P-42:1)
SM(d16:0/16:0)
PC(37:4),PC(O-38:4),PC(P-38:3)
[TG(52:5)]_C18:2
[TG(37:0)]_C18:0
[TG(50:3)]_C18:1
SM(d18:0/24:0)
[TG(52:10),TG(51:3)]_C18:2
[TG(46:1)]_C16:1
SM(d16:0/24:0)
[TG(48:3)]_C18:1
CAR(14:2)
[TG(54:5)]_C20:4
[TG(53:10),TG(52:3)]_C16:1
[TG(46:2)]_C14:0
[TG(46:0)]_C14:0
PC(40:7),PC(39:0),PC(O-40:0)
PC(42:11),PC(41:4),PC(O-42:4)
[TG(46:0)]_C16:0
[TG(55:11),TG(54:4)]_C18:0
[TG(46:3)]_C18:2
FA(37:0)
PC(38:5)
PC(38:3)
[TG(58:8)]_C22:6
[TG(46:1)]_C14:0
[TG(59:11),TG(58:4)]_C18:2
SM(d16:1/17:0)
[TG(53:9),TG(52:2)]_C18:0
[TG(54:5)]_C22:5
[TG(48:3)]_C16:0
[TG(54:7),TG(53:0)]_C18:0
[TG(50:3)]_C18:2
[TG(49:6)]_C16:0
[TG(53:7)]_C18:1
[TG(51:8),TG(50:1)]_C18:0
[TG(52:8),TG(51:1)]_C18:1
[TG(57:11),TG(56:4)]_C18:0
[TG(57:9),TG(56:2)]_C20:0
[TG(50:4)]_C14:0
[TG(42:0)]_C16:0
PC(O-38:8),PC(36:1),PC(O-37:1),PC(P-37:0)
[TG(54:5)]_C16:0
[TG(55:11),TG(54:4)]_C16:0
[TG(55:9),TG(54:2)]_C16:0
DG(36:8),DG(35:1)_C16:1
PC(32:0),PC(O-33:0)
[TG(52:5)]_C20:4
[TG(46:2)]_C16:1
[TG(53:9),TG(52:2)]_C18:1
[TG(54:9),TG(53:2)]_C18:2
[TG(52:7),TG(51:0)]_C16:0
[TG(55:9),TG(54:2)]_C18:1
[TG(49:8),TG(48:1)]_C18:0
PC(42:3)
DG(36:7),DG(35:0)_C16:0
SM(d16:1/18:1)
PC(36:5)
[TG(54:6)]_C16:0
[TG(44:1)]_C16:1
[TG(56:10),TG(55:3)]_C18:1
[TG(51:8),TG(50:1)]_C16:0
PC(39:5),PC(O-40:5),PC(P-40:4)
[TG(55:11),TG(54:4)]_C20:4
[TG(57:12),TG(56:5)]_C18:0
[TG(57:11),TG(56:4)]_C20:0
[TG(59:10),TG(58:3)]_C18:1
LPC(20:2),PC(O-20:2)
SM(d18:2/22:1)
[TG(54:6)]_C20:4
PC(40:10),PC(39:3),PC(O-40:3),PC(P-40:2)
[TG(49:7)]_C18:1

[TG(51:9),TG(50:2)]_C18:2
[TG(50:4)]_C18:2
PC(38:8),PC(37:1),PC(O-38:1),PC(P-38:0)
[TG(48:2)]_C18:2
DG(41:5)_C16:0
[TG(56:6)]_C18:0
[TG(52:4)]_C18:2
SM(d18:1/17:0)
[TG(52:10),TG(51:3)]_C16:0
[TG(50:3)]_C16:0
[TG(53:10),TG(52:3)]_C18:1
[TG(54:7)]_C18:3
PC(38:7),PC(37:0),PC(O-38:0)
PC(42:4)
[TG(55:10),TG(54:3)]_C16:0
PC(29:1),PC(O-30:1),PC(P-30:0)
[TG(52:5)]_C18:1
PC(28:0),PC(O-29:0)
SM(d18:2/21:0)
[TG(51:4)]_C18:2
SM(d16:0/20:0)
PC(40:4)
[TG(52:4)]_C16:0
[TG(50:4)]_C16:0
[TG(48:7),TG(47:0)]_C14:0
PC(35:4),PC(O-36:4),PC(P-36:3)
CE(22:6)Na
[TG(50:8),TG(49:1)]_C16:0
[TG(55:8),TG(54:1)]_C16:0
PC(40:2)
DG(36:7)_C16:1
[TG(51:6)]_C16:0
[TG(42:0)]_C14:0
PC(40:3)
PC(28:1),PC(P-29:0)
PC(33:0),PC(O-34:0)
DG(30:3)_C16:1
PC(38:9),PC(37:2),PC(O-38:2),PC(P-38:1)
[TG(57:12),TG(56:5)]_C18:2
SM(d16:1/22:1)
PC(36:8),PC(35:1),PC(O-36:1),PC(P-36:0)
[TG(57:10),TG(56:3)]_C18:2
[TG(56:6)]_C22:5
[TG(46:2)]_C18:2
PC(37:7),PC(P-38:6),PC(36:0),PC(O-37:0)
SM(d18:1/24:1(15Z))
SM(d16:1/22:0)
[TG(54:5)]_C18:1
[TG(56:8),TG(55:1)]_C16:0
SM(d18:1/26:1(17Z))
PC(41:5),PC(P-42:4)
PC(30:1),PC(O-31:1),PC(P-31:0)
CAR(20:0)
[TG(51:9),TG(50:2)]_C16:0
FA(22:7)
PC(33:1),PC(O-34:1),PC(P-34:0)
SM(d16:0/23:0)
[TG(50:9),TG(49:2)]_C18:2
[TG(52:6)]_C18:3
FA(22:1)
Cer(d18:0/17:0)
[TG(55:10),TG(54:3)]_C18:1
[TG(54:10),TG(53:3)]_C18:2
CAR(10:2)
PC(37:5),PC(O-38:5),PC(P-38:4)
[TG(54:9),TG(53:2)]_C16:0
[TG(42:1)]_C18:1
LPC(18:1),PC(O-18:1),PC(P-18:0)
DG(41:6)_C16:1
[TG(44:1)]_C14:0
[TG(49:7),TG(48:0)]_C18:0
[TG(50:5)]_C18:2
PC(36:4),PC(O-37:4)
PC(31:0),PC(O-32:0)
SM(d18:1/25:0)
SM(d16:0/22:0)
[TG(50:3)]_C18:3
[TG(57:11),TG(56:4)]_C18:2
[TG(48:8),TG(47:1)]_C16:0
PC(34:2),PC(O-35:2),PC(P-35:1)
[TG(55:8),TG(54:1)]_C20:0
[TG(44:1)]_C16:0

[TG(53:8)]_C18:2		
[TG(46:2)]_C18:1		
[TG(49:7),TG(48:0)]_C14:0		
[TG(47:6)]_C16:0		
[TG(48:3)]_C18:3		
SM(d16:1/24:1)		
[TG(56:7)]_C22:5		
PC(31:1),PC(O-32:1),PC(P-32:0)		
[TG(51:8),TG(50:1)]_C18:1		
[TG(51:9),TG(50:2)]_C18:0		
PC(40:6)		
[TG(51:7),TG(50:0)]_C18:0		
PC(42:8),PC(41:1),PC(O-42:1),PC(P-42:0)		
[TG(52:9),TG(51:2)]_C18:1		
[TG(51:7),TG(50:0)]_C14:0		
PC(40:8),PC(39:1),PC(O-40:1),PC(P-40:0)		
SM(d18:0/24:1)		
[TG(55:11),TG(54:4)]_C18:3		
[TG(53:8),TG(52:1)]_C18:0		
[TG(50:7),TG(49:0)]_C18:0		
PC(16:0),PC(O-17:0),LPC(O-18:0)		
PC(38:6)		
[TG(55:9),TG(54:2)]_C18:2		
SM(d16:1/18:0)		
PC(33:3),PC(O-34:3),PC(P-34:2)		
[TG(52:9),TG(51:2)]_C16:0		
PC(37:3),PC(O-38:3),PC(P-38:2)		
[TG(57:8),TG(56:1)]_C18:1		
[TG(48:2)]_C16:1		
PS(O-29:0)		
[TG(53:9),TG(52:2)]_C16:0		
DG(30:2)_C16:0		
PC(30:2),PC(P-31:1)		
PC(40:9),PC(39:2),PC(O-40:2),PC(P-40:1)		
[TG(51:9),TG(50:2)]_C18:1		
[TG(48:8),TG(47:1)]_C14:0		
[TG(50:3)]_C16:1		
[TG(53:9),TG(52:2)]_C16:1		
DG(36:6)_C16:0		
[TG(56:7)]_C20:4		
[TG(57:10),TG(56:3)]_C18:1		
[TG(40:0)]_C16:0		
[TG(55:11),TG(54:4)]_C18:1		
[TG(52:8),TG(51:1)]_C18:0		
PG(16:0),LPG(17:0),LPG(O-18:0); PG(16:0),LPG(17:0),LPG(O-18:0)		
[TG(50:4)]_C20:4		

Table S8. Comparison of Lipid Corona Profiles Between Nanoparticle Sizes
Male 50% BC Samples

Unique Lipids in 50 nm BC	Unique Lipds in 100 nm BC	Shared Lipids
PC(44:10),PC(O-44:3)	CE(20:5)Na	[TG(55:8),TG(54:1)]_C18:1
[TG(44:2)]_C18:1	DG(O-38:8),DG(36:1)_C18:1	CE(20:5) NH4
[TG(54:5)]_C18:0	DG(32:1)_C16:0	CE(22:3)H
[TG(54:6)]_C18:2	CE(20:4)K	[TG(56:11),TG(55:4)]_C18:1
[TG(57:12),TG(56:5)]_C18:1	CE(20:2) NH4	CE(14:0) NH4
PC(35:2),PC(O-36:2),PC(P-36:1)	DG(40:5)_C16:0	CE(20:0)H
[TG(56:12),TG(55:5)]_C18:1	DG(32:0)_C16:0	CE(18:0)K
PC(39:8),PC(O-40:8),PC(38:1),PC(O-39:1),PC(P-39:0)	DG(39:7),DG(38:0),DG(dO-40:0)_C18:0	[TG(56:8)]_C22:6
DG(30:2)_C16:1	CE(20:3)Na	[TG(54:11),TG(53:4)]_C18:1
LPI(20:0)	DG(24:0)_C18:0	LPC(20:4)
[TG(52:4)]_C16:1	CE(16:0)Na	CE(20:2)Na
[TG(50:9),TG(49:2)]_C16:1	DG(37:7)_C16:1	[TG(59:10),TG(58:3)]_C18:2
[TG(57:11),TG(56:4)]_C18:1	DG(36:4),DG(O-37:4)_C18:1	CE(16:1) NH4
LPC(16:0),PC(O-16:0),LPC(O-17:0)	DG(44:7),DG(43:0)_C16:0	[TG(52:5)]_C20:4
[TG(48:2)]_C14:0	DG(O-40:9),DG(38:2)_C18:1	DG(O-38:8),DG(36:1)_C16:1
[TG(52:4)]_C20:4	DG(36:4),DG(O-37:4)_C18:2	CE(20:4) NH4
[TG(52:4)]_C18:1	CE(17:1) NH4	CE(22:2) NH4
[TG(49:8),TG(48:1)]_C14:0	CE(22:2)H	[TG(56:10),TG(55:3)]_C18:1
[TG(57:12),TG(56:5)]_C20:4	CE(17:0) NH4	CE(22:6)Na
PC(40:1),PC(P-41:0)	CE(19:0)K	DG(39:8),DG(O-40:8),DG(38:1)_C18:1
[TG(59:9),TG(58:2)]_C18:1	CE(18:3)K	PC(34:3),PC(P-35:2)
[TG(55:9),TG(54:2)]_C18:0	CE(22:6)H	[TG(56:6)]_C22:5
SM(d16:1/24:0)	DG(38:5)_C16:0	[TG(49:7),TG(48:0)]_C16:0
[TG(59:9),TG(58:2)]_C18:2	709.686225 -> 369.2	PC(34:2),PC(O-35:2),PC(P-35:1)
[TG(53:10),TG(52:3)]_C18:2	DG(38:3)_C18:2	[TG(48:8),TG(47:1)]_C16:0
[TG(54:11),TG(53:4)]_C16:0	CE(18:2)K	[TG(51:7),TG(50:0)]_C18:0
PC(36:7),PC(35:0),PC(O-36:0)	DG(40:2)_C18:2	CE(20:1) NH4
[TG(52:6)]_C16:0	DG(34:4),DG(dO-36:4)_C16:1	CE(19:0)Na
SM(d16:0/25:0)	CE(18:1)Na	[TG(52:8),TG(51:1)]_C18:0
[TG(57:8),TG(56:1)]_C16:0	CE(19:0) NH4	CE(18:1) NH4
Cer(d18:1/24:0)	CE(16:2)Na	DG(39:8),DG(O-40:8)_C18:2
[TG(56:6)]_C20:4	CE(22:1)H	[TG(57:12),TG(56:5)]_C16:0
[TG(49:8),TG(48:1)]_C16:1	DG(40:6),DG(dO-40:0)_C16:0	CE(20:3) NH4
[TG(56:11),TG(55:4)]_C18:2	CE(16:2) NH4	CE(22:1) NH4
[TG(48:7),TG(47:0)]_C16:0	CE(20:5)H	DG(39:7)_C18:1
[TG(58:9)]_C22:6	DG(34:2)_C16:0	PC(39:7),PC(P-40:6),PC(38:0),PC(O-39:0)
PC(39:6),PC(O-40:6),PC(P-40:5)	DG(37:7),DG(36:0)_C18:0	CE(18:3)Na
PI(38:4)	DG(32:2)_C18:2	CE(22:5)H
[TG(45:1)]_C16:0	CE(20:1)K	CE(22:5) NH4
[TG(54:8),TG(53:1)]_C18:1	DG(42:5)_C18:0	[TG(54:7)]_C20:4
[TG(52:10),TG(51:3)]_C18:1	DG(34:2)_C18:2	[TG(48:4)]_C18:2
[TG(45:0)]_C16:0	CE(15:1)K	DG(37:6)_C16:0
DG(42:11),DG(41:4)_C16:0	CE(18:0)H	CE(16:3)Na
[TG(55:7)]_C18:1	CE(18:3)H	CE(22:6) NH4
[TG(55:10),TG(54:3)]_C18:2	DG(30:0)_C16:0	[TG(54:5)]_C20:4
[TG(48:3)]_C18:2	DG(36:3)_C18:1	DG(O-40:9),DG(38:2)_C18:2
[TG(48:2)]_C16:0	CE(18:2)Na	CE(18:1)K
[TG(49:7)]_C16:1	CE(16:0) NH4	CE(20:0) NH4
[TG(56:12),TG(55:5)]_C18:2	DG(42:7),DG(41:0)_C16:0	[TG(59:10),TG(58:3)]_C18:1
DG(36:6)_C16:1	DG(O-38:9),DG(36:2)_C18:2	CE(22:3) NH4
SM(d16:1/23:0)	CE(22:4) NH4	[TG(50:4)]_C16:0
[TG(46:1)]_C16:0	DG(34:0)_C16:0	[TG(57:10),TG(56:3)]_C18:2
[TG(56:6)]_C18:2	DG(37:7),DG(36:0)_C16:0	CE(18:2) NH4
SM(d18:2/14:0)	DG(34:2)_C18:1	CE(22:4)Na
[TG(50:4)]_C16:1	CE(20:5)K	CE(19:0)H
LPC(18:0),PC(O-18:0),LPC(O-19:0)	DG(34:0)_C18:0	[TG(50:3)]_C18:3
[TG(57:9),TG(56:2)]_C18:2	CE(18:3) NH4	[TG(48:3)]_C18:3
[TG(44:0),TG(O-45:0)]_C16:0	DG(32:0)_C18:0	CE(16:0)K
[TG(54:9),TG(53:2)]_C18:0	DG(37:6)_C18:0	CE(20:2)K
[TG(38:1)]_C18:1	FA(15:1)	
PC(32:1),PC(O-33:1),PC(P-33:0)	DG(40:8),DG(39:1)_C18:1	
[TG(53:10),TG(52:3)]_C18:0	DG(34:1)_C16:0	
[TG(39:0)]_C20:0	CE(16:1)Na	
[TG(54:6)]_C18:1	CE(24:1)H	
[TG(50:3)]_C14:0	DG(44:8),DG(43:1)_C16:0	
[TG(50:4)]_C18:1	DG(36:7),DG(35:0)_C18:0	
[TG(49:8),TG(48:1)]_C18:1	DG(O-38:9),DG(36:2)_C18:1	
PG(O-35:1),PG(P-35:0); PG(O-35:1),PG(P-35:0)	DG(O-38:9),DG(36:2)_C18:0	
[TG(49:8)]_C18:2	DG(40:5)_C18:0	
PC(42:6)	CE(18:0) NH4	
PC(34:0),PC(O-35:0)	DG(33:0)_C16:0	
SM(d18:0/15:0)	DG(32:1)_C16:1	
SM(d18:2/18:1)	DG(O-38:8),DG(36:1)_C18:0	
[TG(53:8),TG(52:1)]_C16:0	CE(20:0)Na	
[TG(56:7),TG(55:0)]_C16:0	CE(20:0)K	
PC(42:2)	DG(40:9),DG(39:2)_C18:2	
PC(34:1),PC(O-35:1),PC(P-35:0)	DG(30:1)_C18:1	
SM(d17:1/24:1)	DG(32:1)_C18:1	

[TG(50:9),TG(49:2)]_C14:0	CE(20:4)H	
[TG(48:3)]_C16:1	DG(35:6)_C18:0	
[TG(52:6)]_C16:1	CE(22:4)K	
PC(32:3),PC(P-33:2)	DG(36:3)_C18:2	
PC(35:6),PC(P-36:5)	PC(14:0),LPC(15:0),LPC(O-16:0)	
SM(d17:1/26:1)	CE(20:4)Na	
[TG(53:10),TG(52:3)]_C18:3	DG(35:6)_C16:0	
[TG(54:6)]_C18:3	DG(34:1)_C18:1	
[TG(55:9),TG(54:2)]_C20:0	DG(34:3)_C18:2	
[TG(52:9),TG(51:2)]_C18:2		
[TG(57:9),TG(56:2)]_C18:0		
[TG(50:7),TG(49:0)]_C16:0		
[TG(50:8),TG(49:1)]_C14:0		
[TG(44:0),TG(O-45:0)]_C14:0		
LPG(19:0),LPG(O-20:0); LPG(19:0),LPG(O-20:0)		
PC(O-40:9),PC(38:2),PC(P-39:1)		
[TG(51:7)]_C18:1		
PC(42:10),PC(41:3),PC(O-42:3),PC(P-42:2)		
[TG(44:2)]_C16:0		
SM(d16:1/20:0)		
[TG(51:9),TG(50:2)]_C14:0		
SM(d16:0/16:0)		
PC(37:4),PC(O-38:4),PC(P-38:3)		
[TG(52:5)]_C18:2		
[TG(37:0)]_C18:0		
[TG(50:3)]_C18:1		
[TG(52:4)]_C14:0		
[TG(52:10),TG(51:3)]_C18:2		
[TG(46:1)]_C16:1		
SM(d16:0/24:0)		
[TG(48:3)]_C18:1		
[TG(58:8)]_C22:5		
[TG(46:0)]_C14:0		
[TG(46:2)]_C14:0		
DG(36:5)_C16:0		
[TG(58:7)]_C22:5		
PC(42:11),PC(41:4),PC(O-42:4)		
LPC(18:2),LPC(P-19:1)		
[TG(42:1)]_C16:0		
[TG(46:1)]_C14:0		
[TG(59:11),TG(58:4)]_C18:2		
SM(d16:1/17:0)		
[TG(56:9),TG(55:2)]_C18:1		
[TG(54:5)]_C22:5		
[TG(54:7),TG(53:0)]_C18:0		
[TG(53:8),TG(52:1)]_C16:1		
[TG(52:9),TG(51:2)]_C16:1		
[TG(49:6)]_C16:0		
[TG(51:8),TG(50:1)]_C18:0		
[TG(52:8),TG(51:1)]_C18:1		
[TG(57:9),TG(56:2)]_C20:0		
[TG(57:11),TG(56:4)]_C18:0		
[TG(50:4)]_C14:0		
[TG(42:0)]_C16:0		
PC(O-38:8),PC(36:1),PC(O-37:1),PC(P-37:0)		
[TG(55:11),TG(54:4)]_C16:0		
[TG(55:9),TG(54:2)]_C16:0		
DG(36:8),DG(35:1)_C16:1		
[TG(53:9),TG(52:2)]_C18:1		
[TG(52:7),TG(51:0)]_C16:0		
[TG(55:9),TG(54:2)]_C18:1		
[TG(58:9)]_C20:4		
PC(36:5)		
PC(42:3)		
[TG(53:8),TG(52:1)]_C20:0		
[TG(54:6)]_C16:0		
[TG(44:1)]_C16:1		
[TG(51:8),TG(50:1)]_C16:0		
[TG(55:11),TG(54:4)]_C20:4		
[TG(57:12),TG(56:5)]_C18:0		
PC(40:10),PC(39:3),PC(O-40:3),PC(P-40:2)		
[TG(49:7)]_C18:1		
[TG(53:9),TG(52:2)]_C20:0		
[TG(50:4)]_C18:2		
PC(38:8),PC(37:1),PC(O-38:1),PC(P-38:0)		
[TG(55:7),TG(54:0)]_C16:0		
DG(41:5)_C16:0		
[TG(52:4)]_C18:2		
SM(d18:1/17:0)		
[TG(50:3)]_C16:0		
[TG(53:10),TG(52:3)]_C18:1		
PC(38:7),PC(37:0),PC(O-38:0)		

DG(36:8),DG(35:1)_C18:1		
[TG(52:5)]_C18:1		
PC(28:0),PC(O-29:0)		
PC(34:6)		
[TG(52:9),TG(51:2)]_C18:0		
[TG(57:8),TG(56:1)]_C18:0		
SM(d16:0/20:0)		
PC(40:4)		
[TG(48:7),TG(47:0)]_C14:0		
PC(35:4),PC(O-36:4),PC(P-36:3)		
[TG(50:8),TG(49:1)]_C16:0		
DG(36:7)_C16:1		
[TG(51:6)]_C16:0		
PC(41:7),PC(P-42:6),PC(40:0),PC(O-41:0)		
[TG(42:0)]_C14:0		
PC(40:3)		
PC(33:0),PC(O-34:0)		
DG(30:3)_C16:1		
[TG(57:12),TG(56:5)]_C18:2		
PC(36:8),PC(35:1),PC(O-36:1),PC(P-36:0)		
SM(d18:1/24:1(15Z))		
[TG(54:5)]_C18:1		
SM(d16:1/22:0)		
PC(30:1),PC(O-31:1),PC(P-31:0)		
CAR(20:0)		
[TG(58:14),TG(57:7),TG(56:0)]_C16:0		
[TG(51:9),TG(50:2)]_C16:0		
PC(33:1),PC(O-34:1),PC(P-34:0)		
SM(d16:0/23:0)		
[TG(50:9),TG(49:2)]_C18:2		
[TG(55:10),TG(54:3)]_C20:0		
Cer(d18:0/17:0)		
[TG(55:10),TG(54:3)]_C18:1		
[TG(54:10),TG(53:3)]_C18:2		
PC(37:5),PC(O-38:5),PC(P-38:4)		
[TG(49:7),TG(48:0)]_C18:0		
[TG(50:5)]_C18:2		
PC(31:0),PC(O-32:0)		
[TG(48:4)]_C18:3		
[TG(55:8),TG(54:1)]_C20:0		
[TG(44:1)]_C16:0		
PC(42:0)		
[TG(53:8)]_C18:2		
[TG(46:2)]_C18:1		
FA(31:0)		
[TG(54:7)]_C18:1		
[TG(56:7)]_C22:5		
PC(31:1),PC(O-32:1),PC(P-32:0)		
[TG(51:8),TG(50:1)]_C18:1		
PC(40:6)		
[TG(52:9),TG(51:2)]_C18:1		
[TG(51:7),TG(50:0)]_C14:0		
[TG(55:11),TG(54:4)]_C18:3		
PC(38:6)		
[TG(55:9),TG(54:2)]_C18:2		
SM(d16:1/18:0)		
[TG(52:9),TG(51:2)]_C16:0		
[TG(48:2)]_C16:1		
[TG(53:9),TG(52:2)]_C16:0		
1-O-tricosanoyl-Cer(d18:1/16:0)		
PC(43:6)		
PC(30:2),PC(P-31:1)		
[TG(48:8),TG(47:1)]_C14:0		
[TG(50:3)]_C16:1		
DG(36:6)_C16:0		
[TG(56:7)]_C20:4		
[TG(57:10),TG(56:3)]_C18:1		
[TG(40:0)]_C16:0		
[TG(46:2)]_C16:0		
SM(d18:1/12:0)		
PE(38:4)		
SM(d18:0/17:0)		
PI(38:3)		
PI(36:2),PI(O-37:2),PI(P-37:1)		
Cer(d18:1/22:0)		
PC(33:2),PC(O-34:2),PC(P-34:1)		
SM(d18:0/26:1(17Z))		
[TG(54:11),TG(53:4)]_C18:2		
[TG(47:2)]_C18:2		
[TG(53:7),TG(52:0)]_C16:0		
[TG(41:0)]_C16:0		
[TG(52:4)]_C18:3		

[TG(53:7),TG(52:0)]_C20:0		
[TG(54:5)]_C18:3		
SM(d16:1/16:0)		
[TG(44:1)]_C18:1		
[TG(50:9),TG(49:2)]_C16:0		
PC(39:4),PC(O-40:4),PC(P-40:3)		
[TG(50:9),TG(49:2)]_C18:1		
[TG(51:7),TG(50:0)]_C16:0		
PC(O-38:9),PC(36:2),PC(O-37:2),PC(P-37:1)		
FA(35:0)		
PC(31:2),PC(O-32:2),PC(P-32:1)		
PC(35:5),PC(O-36:5),PC(P-36:4)		
[TG(52:8),TG(51:1)]_C16:0		
[TG(46:0)]_C18:0		
[TG(51:9),TG(50:2)]_C16:1		
[TG(54:10),TG(53:3)]_C16:0		
PC(42:5)		
PC(30:0),PC(O-31:0)		
[TG(48:8),TG(47:1)]_C18:1		
PC(43:4),PC(O-44:4)		
[TG(46:1)]_C18:1		
[TG(54:10),TG(53:3)]_C18:1		
[TG(57:9),TG(56:2)]_C16:0		
[TG(52:6)]_C18:2		
[TG(44:2)]_C18:2		
[TG(53:7),TG(52:0)]_C18:0		
Cer(d18:1/23:0)		
[TG(52:5)]_C18:3		
[TG(52:5)]_C16:0		
LPC(22:4)		
[TG(49:8),TG(48:1)]_C16:0		
[TG(55:10),TG(54:3)]_C18:0		
CAR(14:1)		
[TG(53:8),TG(52:1)]_C18:1		
SM(d16:1/25:0)		
[TG(51:8)]_C18:2		
PC(41:6),PC(O-42:6)		
[TG(55:8),TG(54:1)]_C18:0		
[TG(56:8)]_C18:2		
CE(15:0) NH4		
PC(36:3),PC(P-37:2)		
DG(30:1)_C16:0		
PC(37:6),PC(O-38:6),PC(P-38:5)		
Cer(d18:1/24:1(15Z))		
SM(d17:0/27:0)		
[TG(50:8),TG(49:1)]_C18:1		
[TG(56:8)]_C20:4		
[TG(55:11),TG(54:4)]_C18:2		
[TG(57:8),TG(56:1)]_C20:0		
LPG(20:0); LPG(20:0)		
[TG(46:3)]_C18:1		
[TG(53:9),TG(52:2)]_C18:2		
[TG(48:3)]_C14:0		
[TG(52:7),TG(51:0)]_C18:0		
[TG(55:7),TG(54:0)]_C20:0		
[TG(56:7)]_C18:2		
SM(d16:0/18:0)		
[TG(51:8),TG(50:1)]_C16:1		
[TG(54:9),TG(53:2)]_C18:1		
[TG(61:10),TG(60:3)]_C18:1		
PC(40:5)		
[TG(44:0),TG(O-45:0)]_C18:0		
[TG(57:10),TG(56:3)]_C18:0		
PC(44:0)		
PC(32:2),PC(O-33:2),PC(P-33:1)		
[TG(51:8),TG(50:1)]_C14:0		
SM(d18:1/19:0)		
PC(44:12),PC(O-44:5)		
[TG(56:7)]_C22:6		
FA(6:0)		
[TG(50:4)]_C18:3		
[TG(56:6)]_C16:0		
PC(19:1),LPC(20:1),PC(O-20:1),PC(P-20:0)		
[TG(54:7)]_C18:2		
PC(28:2)		
[TG(54:8),TG(53:1)]_C18:0		
CE(15:1) NH4		
[TG(52:5)]_C16:1		
PC(35:3),PC(O-36:3),PC(P-36:2)		
PC(38:4)		
[TG(57:10),TG(56:3)]_C20:0		
[TG(54:5)]_C18:2		

[TG(62:16),TG(61:9),TG(60:2)]_C18:1		
[TG(57:8)]_C18:2		
SM(d16:1/20:1)		
[TG(53:10),TG(52:3)]_C16:0		
[TG(48:2)]_C18:1		
FA(24:4)		
SM(d18:2/24:1)		
[TG(57:9),TG(56:2)]_C18:1		
PC(42:9),PC(41:2),PC(O-42:2),PC(P-42:1)		
SM(d18:0/24:0)		
CAR(14:2)		
[TG(53:10),TG(52:3)]_C16:1		
PC(40:7),PC(39:0),PC(O-40:0)		
[TG(46:0)]_C16:0		
[TG(46:3)]_C18:2		
[TG(55:11),TG(54:4)]_C18:0		
PC(38:5)		
PC(38:3)		
[TG(58:8)]_C22:6		
[TG(53:9),TG(52:2)]_C18:0		
LPG(18:0); LPG(18:0)		
[TG(48:3)]_C16:0		
[TG(50:3)]_C18:2		
[TG(53:7)]_C18:1		
[TG(54:5)]_C16:1		
[TG(54:5)]_C16:0		
PC(29:0),PC(O-30:0)		
PE(O-38:8),PE(36:1),PE(O-37:1),PE(P-37:0)		
PC(32:0),PC(O-33:0)		
[TG(46:2)]_C16:1		
[TG(54:9),TG(53:2)]_C18:2		
[TG(49:8),TG(48:1)]_C18:0		
SM(d16:1/18:1)		
DG(36:7),DG(35:0)_C16:0		
[TG(52:4)]_C18:0		
PC(39:5),PC(O-40:5),PC(P-40:4)		
[TG(57:11),TG(56:4)]_C20:0		
CAR(18:3)		
LPC(20:2),PC(O-20:2)		
SM(d18:2/22:1)		
[TG(54:6)]_C20:4		
[TG(51:9),TG(50:2)]_C18:2		
[TG(48:2)]_C18:2		
[TG(54:8),TG(53:1)]_C16:0		
[TG(52:10),TG(51:3)]_C16:0		
[TG(54:7)]_C18:3		
PC(42:4)		
[TG(55:10),TG(54:3)]_C16:0		
Cer(d14:2(4E,6E)/16:0)		
PI(34:1),PI(O-35:1),PI(P-35:0)		
PC(29:1),PC(O-30:1),PC(P-30:0)		
SM(d18:2/21:0)		
[TG(51:4)]_C18:2		
PG(20:0),LPG(21:0); PG(20:0),LPG(21:0)		
[TG(52:4)]_C16:0		
[TG(49:3)]_C18:2		
PC(40:2)		
[TG(55:8),TG(54:1)]_C16:0		
[TG(48:8),TG(47:1)]_C16:1		
[TG(51:6)]_C18:0		
PC(28:1),PC(P-29:0)		
PC(38:9),PC(37:2),PC(O-38:2),PC(P-38:1)		
SM(d16:1/22:1)		
[TG(46:2)]_C18:2		
PC(37:7),PC(P-38:6),PC(36:0),PC(O-37:0)		
[TG(46:1)]_C18:0		
[TG(56:8),TG(55:1)]_C16:0		
SM(d18:1/26:1(17Z))		
PC(41:5),PC(P-42:4)		
PC(42:7),PC(41:0),PC(O-42:0)		
PG(32:0),PG(O-33:0); PG(32:0),PG(O-33:0)		
[TG(52:6)]_C18:3		
[TG(46:3)]_C16:1		
PE(O-38:9),PE(36:2),PE(O-37:2),PE(P-37:1)		
[TG(42:0)]_C18:0		
[TG(54:9),TG(53:2)]_C16:0		
[TG(42:1)]_C18:1		
LPC(18:1),PC(O-18:1),PC(P-18:0)		
[TG(44:1)]_C14:0		
DG(41:6)_C16:1		
PC(36:4),PC(O-37:4)		
SM(d18:1/25:0)		

SM(d16:0/22:0)		
[TG(57:11),TG(56:4)]_C18:2		
[TG(50:5)]_C20:4		
[TG(53:10),TG(52:3)]_C20:0		
[TG(49:7),TG(48:0)]_C14:0		
[TG(47:6)]_C16:0		
SM(d16:1/24:1)		
[TG(51:9),TG(50:2)]_C18:0		
PC(42:8),PC(41:1),PC(O-42:1),PC(P-42:0)		
SM(d18:0/24:1)		
PC(40:8),PC(39:1),PC(O-40:1),PC(P-40:0)		
[TG(53:8),TG(52:1)]_C18:0		
[TG(50:7),TG(49:0)]_C18:0		
PC(16:0),PC(O-17:0),LPC(O-18:0)		
PC(33:3),PC(O-34:3),PC(P-34:2)		
PC(37:3),PC(O-38:3),PC(P-38:2)		
[TG(57:8),TG(56:1)]_C18:1		
PS(O-29:0)		
DG(30:2)_C16:0		
[TG(51:9),TG(50:2)]_C18:1		
PC(40:9),PC(39:2),PC(O-40:2),PC(P-40:1)		
[TG(53:9),TG(52:2)]_C16:1		
[TG(55:11),TG(54:4)]_C18:1		
[TG(50:4)]_C20:4		
PG(16:0),LPG(17:0),LPG(O-18:0); PG(16:0),LPG(17:0),LPG(O-18:0)		

Table S8. Comparison of Lipid Corona Profiles Between Nanoparticle Sizes
Male 75% Serum BC Samples

Unique Lipids in 50 nm BC	Unique Lipds in 100 nm BC	Shared Lipids	50 nm Average of Shared Lipids	100 nm Average of Shared Lipids	p-value of Shared Lipids
[TG(44:2)]_C18:1	CE(22:5)Na	PC(40:10),PC(39:3),PC(O-40:3),PC(P-40:2)	11143.55274	25275.9638	2.60319E-07
[TG(54:5)]_C18:0	DG(O-38:8),DG(36:1)_C18:1	PC(37:3),PC(O-38:3),PC(P-38:2)	18418.38128	41692.99911	6.47181E-07
[TG(59:13),TG(58:6)]_C18:1	DG(32:1)_C16:0	LPC(18:1),PC(O-18:1),PC(P-18:0)	11896.28078	21684.43815	7.65335E-07
[TG(56:12),TG(55:5)]_C18:1	CE(20:4)K	PE(O-38:9),PE(36:2),PE(O-37:2),PE(P-37:1)	2134.720153	3730.093585	2.33471E-06
DG(30:2)_C16:1	CE(20:2) NH4	LPC(16:0),PC(O-16:0),LPC(O-17:0)	115949.3561	224380.6737	2.67169E-06
[TG(50:9),TG(49:2)]_C16:1	DG(40:5)_C16:0	SM(d16:0/25:0)	6536.764431	11183.51151	3.2541E-06
[TG(57:11),TG(56:4)]_C18:1	Cer(d18:0/21:0)	SM(d17:1/26:1)	8138.280631	14648.2843	3.32736E-06
[TG(49:8),TG(48:1)]_C14:0	PC(42:1)	PC(41:5),PC(P-42:4)	4097.312309	7709.739242	4.11401E-06
[TG(59:9),TG(58:2)]_C18:1	LPC(22:5)	PC(39:4),PC(O-40:4),PC(P-40:3)	8472.308606	16387.76916	5.70199E-06
[TG(59:9),TG(58:2)]_C18:2	DG(32:0)_C16:0	LPC(18:2),LPC(P-19:1)	5201.392377	12208.48618	7.60684E-06
[TG(54:11),TG(53:4)]_C16:0	DG(39:7),DG(38:0),DG(dO-40:0)_C18:0	LPC(18:0),PC(O-18:0),LPC(O-19:0)	133033.3145	253310.9485	8.19459E-06
[TG(52:6)]_C16:0	CE(20:3)Na	PC(38:7),PC(37:0),PC(O-38:0)	3596.460242	6262.290391	9.37835E-06
[TG(57:8),TG(56:1)]_C16:0	CE(16:0)Na	SM(d16:1/25:0)	32084.23427	57033.23623	9.84006E-06
Cer(d18:1/24:0)	DG(36:4),DG(O-37:4)_C18:1	PC(35:4),PC(O-36:4),PC(P-36:3)	25855.44176	45150.24654	1.06657E-05
[TG(49:8),TG(48:1)]_C16:1	DG(44:7),DG(43:0)_C16:0	PC(36:4),PC(O-37:4)	237591.0012	445345.8618	1.12883E-05
[TG(56:11),TG(55:4)]_C18:2	DG(36:4),DG(O-37:4)_C18:2	PC(38:6)	51535.37183	101087.1457	1.2675E-05
[TG(48:7),TG(47:0)]_C16:0	SM(d18:2/20:1)	PC(38:4)	159989.1512	296191.2342	1.27682E-05
[TG(58:9)]_C22:6	CE(24:1) NH4	PC(36:8),PC(35:1),PC(O-36:1),PC(P-36:0)	17900.10928	34821.70719	1.34573E-05
[TG(54:8),TG(53:1)]_C18:1	CE(17:1) NH4	SM(d16:0/23:0)	6379.100405	11385.11077	1.51645E-05
[TG(45:0)]_C16:0	CE(22:2)H	PC(34:2),PC(O-35:2),PC(P-35:1)	465272.8968	856450.6706	1.60784E-05
DG(42:11),DG(41:4)_C16:0	CE(17:0) NH4	PC(42:11),PC(41:4),PC(O-42:4)	3621.264246	6635.574496	1.65428E-05
LPC(15:1),LPC(O-16:1),LPC(P-16:0)	CE(19:0)K	PC(40:6)	21805.09753	38604.42856	1.84766E-05
[TG(49:7)]_C16:1	CE(18:3)K	PC(30:2),PC(P-31:1)	15726.64516	26744.30929	1.93569E-05
DG(36:6)_C16:1	DG(38:5)_C16:0	PC(35:2),PC(O-36:2),PC(P-36:1)	12649.47698	22472.16771	1.96638E-05
[TG(46:1)]_C16:0	709.686225 -> 369.2	SM(d16:1/24:1)	112030.912	207953.9396	2.02675E-05
SM(d18:2/14:0)	DG(38:3)_C18:2	PC(38:5)	52729.04355	108059.8049	2.15751E-05
[TG(57:9),TG(56:2)]_C18:2	CE(18:2)K	PC(36:3),PC(P-37:2)	201735.5286	374181.8853	2.24382E-05
[TG(44:0),TG(O-45:0)]_C16:0	DG(34:2)_C16:1	PC(O-38:9),PC(36:2),PC(O-37:2),PC(P-37:1)	348632.6332	635198.0898	2.40601E-05
[TG(54:9),TG(53:2)]_C18:0	DG(40:2)_C18:2	LPC(20:4)	2744.740195	9020.880598	2.41157E-05
[TG(53:10),TG(52:3)]_C18:0	DG(38:7),DG(37:0)_C16:0	PC(33:1),PC(O-34:1),PC(P-34:0)	9068.884634	14881.64501	2.42532E-05
PG(O-35:1),PG(P-35:0); PG(O-35:1),PG(P-35:0)	CE(15:0)K	SM(d16:1/18:1)	34137.98651	65896.95525	2.5068E-05
[TG(49:8)]_C18:2	[TG(38:0)]_C20:0	SM(d18:2/24:1)	111534.6769	207152.2002	2.80081E-05
PC(42:6)	PI(34:2),PI(O-35:2),PI(P-35:1)	PC(39:5),PC(O-40:5),PC(P-40:4)	8794.060622	17331.50782	3.00907E-05
PC(42:2)	DG(34:4),DG(dO-36:4)_C16:1	SM(d18:2/22:1)	91084.0539	165992.4866	3.04561E-05
PC(19:0),LPC(20:0),PC(O-20:0)	CE(18:1)Na	PC(37:4),PC(O-38:4),PC(P-38:3)	16776.20919	30523.07698	3.64043E-05
[TG(52:6)]_C16:1	CE(19:0) NH4	PC(34:1),PC(O-35:1),PC(P-35:0)	249627.5345	467748.6791	3.67861E-05
[TG(49:3)]_C16:0	CE(16:2)Na	PC(38:8),PC(37:1),PC(O-38:1),PC(P-38:0)	19668.82139	37342.54293	4.17923E-05
PC(35:6),PC(P-36:5)	PC(44:2)	SM(d16:0/22:0)	79545.13737	140710.1978	4.66469E-05
PC(32:3),PC(P-33:2)	CE(22:1)H	PC(36:7),PC(35:0),PC(O-36:0)	3824.564277	5902.107756	5.13719E-05
[TG(53:10),TG(52:3)]_C18:3	DG(40:6),DG(dO-40:0)_C16:0	[TG(51:7),TG(50:0)]_C18:0	10532.23675	8323.616575	5.22054E-05
[TG(57:12),TG(56:5)]_C22:5	CE(20:5)H	PC(38:3)	107460.0681	199010.8107	5.47717E-05
[TG(52:9),TG(51:2)]_C18:2	CE(16:2) NH4	PC(O-38:8),PC(36:1),PC(O-37:1),PC(P-37:0)	115545.8238	202460.523	5.98118E-05
[TG(52:5)]_C22:5	DG(34:2)_C16:0	PC(38:9),PC(37:2),PC(O-38:2),PC(P-38:1)	18294.57726	35860.89524	6.68591E-05
[TG(50:7),TG(49:0)]_C16:0	DG(37:7),DG(36:0)_C18:0	SM(d16:1/24:0)	194260.1047	344896.5149	6.92317E-05
LPG(19:0),LPG(O-20:0); LPG(19:0),LPG(O-20:0)	DG(32:2)_C18:2	PC(36:5)	9770.044671	16334.55846	7.33836E-05
[TG(50:8),TG(49:1)]_C14:0	CE(20:1)K	PC(39:6),PC(O-40:6),PC(P-40:5)	4899.228407	7467.241855	8.35721E-05
[TG(44:0),TG(O-45:0)]_C14:0	DG(44:8),DG(43:1)_C18:1	SM(d16:1/22:1)	16581.64919	29371.63687	8.62208E-05
[TG(60:15),TG(59:8),TG(58:1)]_C18:1	DG(42:5)_C18:0	SM(d17:1/24:1)	21907.07345	43540.96169	9.90835E-05
PC(42:10),PC(41:3),PC(O-42:3),PC(P-42:2)	PE(38:4)	PC(35:3),PC(O-36:3),PC(P-36:2)	11178.76079	19862.06801	0.000104524
[TG(44:2)]_C16:0	DG(34:2)_C18:2	SM(d18:1/24:1(15Z))	161983.1121	283779.5483	0.000105414
SM(d16:0/16:0)	DG(34:3)_C16:1	PC(O-40:9),PC(38:2),PC(P-39:1)	77542.47316	129942.0397	0.000125601
[TG(37:0)]_C18:0	CE(15:1)K	PC(40:4)	10092.92076	15813.20109	0.000145234
[TG(57:11),TG(56:4)]_C16:0	CE(18:0)H	PC(40:7),PC(39:0),PC(O-40:0)	5285.900388	8391.900524	0.00014668
[TG(52:4)]_C14:0	CE(18:3)H	SM(d16:1/16:0)	29232.44176	48838.57656	0.000148253
[TG(46:1)]_C16:1	DG(30:0)_C16:0	[TG(39:0)]_C20:0	15714.01704	6372.943114	0.000187871
[TG(58:8)]_C22:5	DG(36:3)_C18:1	CE(20:0) NH4	28552.606	11956.13152	0.000193017
[TG(46:0)]_C14:0	CE(18:2)Na	SM(d18:0/24:1)	65945.01252	111687.3324	0.000203501
[TG(58:7)]_C22:5	CE(16:0) NH4	SM(d18:2/18:1)	2344.016164	3331.532889	0.00022037
DG(36:5)_C16:0	DG(42:7),DG(41:0)_C16:0	PC(34:3),PC(P-35:2)	13505.40898	20560.33286	0.000262289
[TG(42:1)]_C16:0	DG(O-38:9),DG(36:2)_C18:2	PC(32:2),PC(O-33:2),PC(P-33:1)	13501.713	21967.31686	0.000288034
[TG(46:1)]_C14:0	PC(44:5)	CE(22:5)H	7558.652528	4496.215644	0.000352163

[TG(59:11),TG(58:4)]_C18:2	PE(38:6)	SM(d18:1/12:0)	2134.664147	2726.957524	0.00035772
[TG(54:5)]_C22:5	CE(22:4) NH4	PC(33:2),PC(O-34:2),PC(P-34:1)	11500.48081	18907.00135	0.000383725
[TG(53:8),TG(52:1)]_C16:1	DG(34:0)_C16:0	SM(d18:1/19:0)	8732.764702	14555.04843	0.000643788
[TG(52:9),TG(51:2)]_C16:1	PE(37:6),PE(O-38:6),PE(P-38:5)	CE(20:2)Na	7023.188535	4384.231644	0.001231913
[TG(49:6)]_C16:0	DG(37:7),DG(36:0)_C16:0	[TG(56:7),TG(55:0)]_C16:0	1731.704131	2058.787489	0.002756764
[TG(51:8),TG(50:1)]_C18:0	DG(34:2)_C18:1	PC(35:5),PC(O-36:5),PC(P-36:4)	17107.02511	23021.79226	0.004021088
[TG(57:11),TG(56:4)]_C18:0	CE(20:5)K	CE(16:0)K	24472.47373	16723.94656	0.004607191
[TG(42:0)]_C16:0	DG(34:0)_C18:0	CE(20:0)H	3897.85228	3243.905558	0.006450413
[TG(55:11),TG(54:4)]_C16:0	DG(34:3)_C16:0	[TG(51:7),TG(50:0)]_C16:0	25281.58979	21349.67955	0.008217117
[TG(55:9),TG(54:2)]_C16:0	LPC(22:6)	[TG(49:7),TG(48:0)]_C16:0	36535.25848	29244.00134	0.010137039
DG(36:8),DG(35:1)_C16:1	DG(32:0)_C18:0	[TG(56:10),TG(55:3)]_C18:1	1711.440118	2018.030146	0.011407314
[TG(52:7),TG(51:0)]_C16:0	FA(14:2)	PC(37:7),PC(P-38:6),PC(36:0),PC(O-37:0)	13633.22493	17899.07518	0.011607989
[TG(58:9)]_C20:4	FA(6:0)	[TG(56:11),TG(55:4)]_C18:1	1578.968113	2040.004814	0.016610833
[TG(53:8),TG(52:1)]_C20:0	CE(17:0)Na	[TG(55:9),TG(54:2)]_C20:0	3196.328233	1952.371473	0.017799369
[TG(44:1)]_C16:1	DG(37:6)_C18:0	[TG(57:9),TG(56:2)]_C20:0	3263.836229	2011.812799	0.018563212
[TG(45:0)]_C14:0	DG(40:8),DG(39:1)_C18:1	[TG(52:5)]_C18:3	7762.268568	9741.482018	0.027765867
[TG(55:11),TG(54:4)]_C20:4	FA(15:1)	[TG(55:8),TG(54:1)]_C18:0	7636.33652	4271.3763	0.029792484
[TG(57:12),TG(56:5)]_C18:0	DG(34:1)_C16:0	[TG(55:8),TG(54:1)]_C18:1	5721.240409	5089.257042	0.031857315
[TG(49:7)]_C18:1	CE(16:1)Na	PC(40:3)	4821.66037	5861.38375	0.033386899
[TG(53:9),TG(52:2)]_C20:0	DG(37:6)_C16:0	CE(22:6) NH4	21328.90559	15926.42851	0.034090292
DG(41:5)_C16:0	FA(10:2)	PC(39:7),PC(P-40:6),PC(38:0),PC(O-39:0)	6123.356425	7070.573184	0.038527836
PI(36:4)	CE(24:1)H	[TG(56:8)]_C22:6	1995.148136	2302.351487	0.041746553
PC(28:0),PC(O-29:0)	DG(44:8),DG(43:1)_C16:0	CE(20:1) NH4	8447.360639	5775.273736	0.042617623
PC(34:6)	DG(O-38:9),DG(36:2)_C18:1	[TG(56:8)]_C18:2	1849.440132	2242.893494	0.043344142
[TG(57:8),TG(56:1)]_C18:0	DG(36:7),DG(35:0)_C18:0	[TG(56:6)]_C18:2	2297.240155	2868.746887	0.04459126
[TG(58:8)]_C20:4	DG(O-38:9),DG(36:2)_C18:0	[TG(49:7),TG(48:0)]_C18:0	4769.780321	2772.243539	0.048796896
[TG(48:7),TG(47:0)]_C14:0	DG(40:5)_C18:0	[TG(57:12),TG(56:5)]_C16:0	1894.92013	2201.553488	0.050225275
[TG(50:8),TG(49:1)]_C16:0	DG(44:9),DG(43:2)_C18:2	[TG(48:4)]_C18:2	2206.488145	2558.421527	0.065703562
DG(36:7)_C16:1	DG(33:0)_C16:0	SM(d18:0/26:1(17Z))	3370.964246	2036.89082	0.067611809
[TG(51:6)]_C16:0	DG(32:1)_C16:1	DG(36:3)_C18:2	4760.364308	8185.894566	0.069123761
[TG(42:0)]_C14:0	DG(31:1)_C16:0	[TG(46:0)]_C16:0	18346.20938	15367.08245	0.072000442
PC(33:0),PC(O-34:0)	DG(O-38:8),DG(36:1)_C18:0	[TG(57:10),TG(56:3)]_C20:0	2997.124242	1820.567469	0.07386825
DG(30:3)_C16:1	CE(20:0)Na	CE(22:3)H	3764.420255	3477.10691	0.074561247
[TG(57:12),TG(56:5)]_C18:2	CE(22:6)K	[TG(50:4)]_C18:1	2583.316178	2913.67954	0.079287424
CAR(20:0)	CE(20:0)K	[TG(51:8),TG(50:1)]_C16:0	173829.088	107526.7232	0.090790472
FA(22:7)	CE(22:3) NH4	[TG(57:11),TG(56:4)]_C18:2	2562.440192	2951.603541	0.09453518
[TG(55:10),TG(54:3)]_C20:0	DG(40:9),DG(39:2)_C18:2	CE(18:3) NH4	36562.02644	59612.92484	0.10919658
Cer(d18:0/17:0)	DG(30:1)_C18:1	PC(40:9),PC(39:2),PC(O-40:2),PC(P-40:1)	6778.38049	11677.54407	0.110692027
CAR(10:2)	DG(32:1)_C18:1	[TG(54:6)]_C18:1	6163.816449	7171.893233	0.114390323
[TG(58:10)]_C20:4	CE(20:4)H	CE(20:5) NH4	13704.41702	22175.97426	0.117586412
PC(36:6)	DG(35:6)_C18:0	[TG(54:9),TG(53:2)]_C18:1	4396.004336	4993.661058	0.122517529
PC(31:0),PC(O-32:0)	CE(22:4)K	[TG(62:16),TG(61:9),TG(60:2)]_C18:1	1411.820098	923.386732	0.122727398
[TG(54:6)]_C16:1	DG(42:8),DG(41:1)_C18:1	[TG(53:8),TG(52:1)]_C16:0	37614.88252	24233.16161	0.12524701
[TG(55:8),TG(54:1)]_C20:0	CE(20:1)H	[TG(57:10),TG(56:3)]_C18:2	2493.124168	1837.764121	0.126994707
[TG(44:1)]_C16:0	[TG(46:3)]_C16:1	[TG(54:7)]_C18:2	4314.544326	4989.224377	0.12943017
PC(42:0)	PE(34:2),PE(O-35:2),PE(P-35:1)	[TG(53:8),TG(52:1)]_C18:0	31749.67828	23447.01422	0.137534877
[TG(53:8)]_C18:2	CE(20:4)Na	[TG(48:3)]_C18:2	5331.408376	6214.521731	0.142497205
[TG(46:2)]_C18:1	CE(16:1)K	[TG(51:7)]_C18:1	6993.180455	4602.913632	0.146113065
[TG(54:7)]_C18:1	DG(35:6)_C16:0	CE(22:2) NH4	13836.30505	21539.75218	0.149628216
[TG(55:11),TG(54:4)]_C18:3	CE(22:0)K	CE(19:0)Na	4656.920335	7240.433865	0.15084109
[TG(51:7),TG(50:0)]_C14:0	DG(34:1)_C18:1	[TG(55:10),TG(54:3)]_C18:0	19675.42127	22597.7681	0.152551502
[TG(55:9),TG(54:2)]_C18:2	DG(34:3)_C18:2	LPC(20:3)	1766.424122	3023.027527	0.154055733
[TG(56:8)]_C18:3		[TG(54:5)]_C18:1	39876.13904	46788.23275	0.16425476
PI(38:5)		[TG(50:3)]_C18:1	14871.913	17025.57991	0.164800681
1-O-tricosanoyl-Cer(d18:1/16:0)		SM(d16:1/20:1)	22072.74555	33718.09969	0.170760825
PC(43:6)		[TG(48:8),TG(47:1)]_C18:1	1781.424132	1207.817416	0.173816539
[TG(48:8),TG(47:1)]_C14:0		[TG(52:5)]_C18:2	10415.64466	12016.33809	0.174174999
DG(36:6)_C16:0		PI(36:1),PI(O-37:1),PI(P-37:0)	1126.468085	2005.15482	0.1824126
[TG(40:0)]_C16:0		DG(39:8),DG(O-40:8),DG(38:1)_C18:1	149415.5674	129847.4052	0.182653909
[TG(52:8),TG(51:1)]_C18:0		SM(d16:1/22:0)	206393.5003	309466.116	0.190001247
SM(d18:0/17:0)		PC(40:5)	16007.96517	23677.5159	0.194081563
PI(38:3)		SM(d18:2/21:0)	5505.484364	8049.091196	0.19791074
Cer(d18:1/22:0)		[TG(54:5)]_C18:2	59780.23622	68608.09311	0.198444196
[TG(47:2)]_C18:2		PC(40:2)	5567.932384	4308.302302	0.203059884
[TG(53:7),TG(52:0)]_C16:0		[TG(52:4)]_C18:2	87187.45404	100443.9386	0.203691157

[TG(41:0)]_C16:0	
[TG(53:7),TG(52:0)]_C20:0	
[TG(44:1)]_C18:1	
[TG(50:9),TG(49:2)]_C16:0	
[TG(50:9),TG(49:2)]_C18:1	
PC(31:2),PC(O-32:2),PC(P-32:1)	
[TG(52:8),TG(51:1)]_C16:0	
[TG(46:0)]_C18:0	
[TG(54:10),TG(53:3)]_C16:0	
PC(43:4),PC(O-44:4)	
[TG(57:9),TG(56:2)]_C16:0	
[TG(52:6)]_C18:2	
[TG(44:2)]_C18:2	
[TG(53:7),TG(52:0)]_C18:0	
Cer(d18:1/23:0)	
LPC(22:4)	
CAR(14:1)	
[TG(48:4)]_C18:1	
[TG(54:12),TG(53:5)]_C18:2	
DG(30:1)_C16:0	
Cer(d18:1/24:1(15Z))	
SM(d17:0/27:0)	
[TG(50:8),TG(49:1)]_C18:1	
[TG(56:8)]_C20:4	
[TG(57:8),TG(56:1)]_C20:0	
LPG(20:0); LPG(20:0)	
[TG(46:3)]_C18:1	
[TG(52:7),TG(51:0)]_C18:0	
[TG(55:7),TG(54:0)]_C20:0	
[TG(56:7)]_C18:2	
[TG(51:8),TG(50:1)]_C16:1	
[TG(61:10),TG(60:3)]_C18:1	
[TG(44:0),TG(O-45:0)]_C18:0	
[TG(57:10),TG(56:3)]_C18:0	
PC(44:12),PC(O-44:5)	
[TG(50:4)]_C18:3	
[TG(56:6)]_C16:0	
PC(19:1),LPC(20:1),PC(O-20:1),PC(P-20:0)	
PC(28:2)	
[TG(54:8),TG(53:1)]_C18:0	
CE(15:1) NH4	
PC(42:9),PC(41:2),PC(O-42:2),PC(P-42:1)	
CAR(14:2)	
[TG(55:11),TG(54:4)]_C18:0	
[TG(58:8)]_C22:6	
CE(20:1)Na	
[TG(53:7)]_C18:1	
SM(d18:2/15:0)	
[TG(54:5)]_C16:1	
PC(29:0),PC(O-30:0)	
[TG(54:9),TG(53:2)]_C18:2	
[TG(49:8),TG(48:1)]_C18:0	
DG(36:7),DG(35:0)_C16:0	
[TG(57:11),TG(56:4)]_C20:0	
LPC(20:2),PC(O-20:2)	
[TG(59:10),TG(58:3)]_C18:1	
[TG(54:8),TG(53:1)]_C16:0	
[TG(56:6)]_C18:0	
[TG(52:10),TG(51:3)]_C16:0	
Cer(d14:2(4E,6E)/16:0)	
PC(29:1),PC(O-30:1),PC(P-30:0)	
[TG(51:4)]_C18:2	
[TG(56:8),TG(55:1)]_C18:1	
[TG(50:7)]_C18:1	
[TG(55:8),TG(54:1)]_C16:0	

[TG(54:5)]_C20:4	8757.92859	9842.185379	0.204220214
PC(41:7),PC(P-42:6),PC(40:0),PC(O-41:0)	2155.564159	2789.794202	0.208660456
[TG(51:9),TG(50:2)]_C14:0	9672.488721	10968.40545	0.209079507
CE(18:0) NH4	5104.008363	7518.46853	0.210638084
PC(37:5),PC(O-38:5),PC(P-38:4)	19023.00532	27733.85921	0.210686233
[TG(52:5)]_C18:1	2438.024188	2687.740869	0.216611624
[TG(52:4)]_C18:1	18581.81721	20721.79085	0.221712285
PC(30:1),PC(O-31:1),PC(P-31:0)	161111.2383	231443.9523	0.223487611
[TG(51:8),TG(50:1)]_C18:1	87561.54621	67864.20162	0.225944765
[TG(52:4)]_C16:0	59099.62046	66790.77028	0.227284552
SM(d16:0/24:0)	30780.70217	43876.18625	0.229770894
CE(18:1)K	4553.088293	6597.575125	0.23305485
CE(18:0)K	31178.19025	45363.63133	0.233857576
[TG(49:8),TG(48:1)]_C16:0	35817.03874	25164.37432	0.234189862
CE(18:1) NH4	163135.7643	145422.5396	0.242608808
[TG(50:3)]_C18:2	24569.95376	27547.44075	0.243491075
SM(d16:0/18:0)	45305.67894	63791.84064	0.245885789
[TG(50:3)]_C18:3	4585.012356	3223.172245	0.246048464
[TG(48:2)]_C18:1	9800.632693	11251.94944	0.247575108
DG(39:7)_C18:1	8455.228554	7625.041844	0.248151315
[TG(55:10),TG(54:3)]_C18:1	102265.4914	114303.1245	0.248691273
SM(d16:1/18:0)	461115.2662	645581.784	0.24890934
PC(40:1),PC(P-41:0)	3332.844223	2649.290844	0.253959588
CE(20:5)Na	4724.360338	6693.033808	0.259527618
[TG(49:8),TG(48:1)]_C18:1	26659.61368	19061.54005	0.260484143
PI(38:4)	7099.38047	9751.514045	0.262070869
[TG(52:8),TG(51:1)]_C18:1	4212.224303	3346.554236	0.264609784
DG(O-40:9),DG(38:2)_C18:1	2514.596175	3539.136241	0.273360485
[TG(48:3)]_C14:0	2519.196172	2010.916138	0.275265553
[TG(53:10),TG(52:3)]_C16:0	178293.6412	201106.3858	0.277982667
[TG(51:9),TG(50:2)]_C18:1	42349.86297	46795.95628	0.278263818
[TG(46:3)]_C18:2	1592.832111	1159.602076	0.280168421
SM(d16:0/20:0)	11485.3087	15532.70583	0.281692692
[TG(46:2)]_C18:2	3929.084297	4442.550975	0.282376598
[TG(52:5)]_C20:4	2404.08417	2573.767523	0.284590242
[TG(53:9),TG(52:2)]_C16:1	3416.104257	3706.550947	0.301437565
CE(22:6)H	2120.912146	2945.105538	0.306003157
PC(39:8),PC(O-40:8),PC(38:1),PC(O-39:1),PC(P-39:0)	30838.18624	40810.02696	0.311370308
SM(d18:1/25:0)	5075.716346	6706.816475	0.313687905
CE(20:4) NH4	153563.3779	210809.8199	0.316366315
PC(28:1),PC(P-29:0)	11877.64092	15550.7844	0.318454094
[TG(59:10),TG(58:3)]_C18:2	2192.58016	2059.503484	0.319227727
CE(22:4)Na	4767.980334	5897.363765	0.331088664
[TG(53:10),TG(52:3)]_C18:1	173276.4409	191367.3802	0.332612815
[TG(51:9),TG(50:2)]_C16:1	20440.00148	22327.13501	0.336039102
SM(d18:1/17:0)	9837.500657	12754.44423	0.337968208
[TG(46:1)]_C18:1	7110.968481	5325.878355	0.340069325
[TG(54:5)]_C16:0	5617.696419	4190.399645	0.34371435
[TG(57:9),TG(56:2)]_C18:0	1508.844101	1140.337411	0.347659402
[TG(52:9),TG(51:2)]_C18:1	5858.376423	4416.916355	0.348177873
[TG(53:9),TG(52:2)]_C18:1	256596.3567	280447.3032	0.34850325
[TG(56:7)]_C22:5	1905.256138	1438.896099	0.352739909
[TG(48:8),TG(47:1)]_C16:0	1810.200117	1492.566772	0.354163537
CE(18:3)Na	21960.18946	29347.29679	0.360542117
[TG(49:3)]_C18:2	1435.680103	1098.940077	0.370009943
[TG(51:9),TG(50:2)]_C18:2	43293.85123	35910.08726	0.379961121
[TG(46:2)]_C14:0	1991.728133	1532.781437	0.380605116
PC(37:6),PC(O-38:6),PC(P-38:5)	7898.236519	9888.900056	0.388577233
CE(16:3)Na	3291.360246	4326.763646	0.38895818
DG(37:7)_C16:1	2042.416149	2676.98619	0.390625152
CE(14:0) NH4	2931.144199	3842.367601	0.392542474
CE(22:1) NH4	2601.076186	3401.732902	0.394664324
[TG(52:9),TG(51:2)]_C16:0	4318.464288	3331.607551	0.397425893
[TG(52:10),TG(51:3)]_C18:2	3419.944235	2638.150849	0.401665847
[TG(53:8),TG(52:1)]_C18:1	40655.9908	37938.48052	0.402756884

[TG(48:8),TG(47:1)]_C16:1	
[TG(51:6)]_C18:0	
[TG(56:8),TG(55:1)]_C16:0	
[TG(46:1)]_C18:0	
PC(42:7),PC(41:0),PC(O-42:0)	
PG(32:0),PG(O-33:0); PG(32:0),PG(O-33:0)	
[TG(52:6)]_C18:3	
[TG(54:9),TG(53:2)]_C16:0	
[TG(42:1)]_C18:1	
[TG(44:1)]_C14:0	
DG(41:6)_C16:1	
[TG(50:5)]_C20:4	
[TG(49:7),TG(48:0)]_C14:0	
[TG(47:6)]_C16:0	
FA(21:0)	
PC(24:0)	
PC(42:8),PC(41:1),PC(O-42:1),PC(P-42:0)	
[TG(58:9),TG(57:2)]_C18:1	
[TG(50:7),TG(49:0)]_C18:0	
PC(16:0),PC(O-17:0),LPC(O-18:0)	
[TG(57:8),TG(56:1)]_C18:1	
PS(O-29:0)	
DG(30:2)_C16:0	
[TG(50:4)]_C20:4	
PG(16:0),LPG(17:0),LPG(O-18:0); PG(16:0),LPG(17:0),LPG(O-18:0)	

[TG(56:7)]_C22:6	2449.968179	1904.271471	0.407144974
[TG(54:6)]_C16:0	2909.388206	2265.521505	0.408655932
[TG(53:9),TG(52:2)]_C16:0	164098.7919	178113.8253	0.419483659
CE(20:3) NH4	22210.60972	28765.77329	0.419984806
[TG(48:2)]_C14:0	8496.180574	6612.557191	0.422299834
[TG(55:9),TG(54:2)]_C18:1	33658.20643	28373.40945	0.427684835
DG(O-38:8),DG(36:1)_C16:1	12566.55685	16133.15376	0.437821776
[TG(52:5)]_C16:0	7652.828522	6001.631695	0.438165932
[TG(48:3)]_C16:0	3103.004205	3299.987561	0.439782142
[TG(53:9),TG(52:2)]_C18:0	20146.08942	17200.65263	0.443179031
[TG(50:9),TG(49:2)]_C18:2	2622.492188	2768.78553	0.447325086
[TG(52:4)]_C16:1	10307.12078	8133.873986	0.450135487
[TG(48:3)]_C18:3	2202.256149	2295.219491	0.467198402
[TG(54:7)]_C20:4	1647.636107	1421.992761	0.467277144
DG(O-40:9),DG(38:2)_C18:2	795787.6651	1002205.273	0.477249496
[TG(51:9),TG(50:2)]_C16:0	106197.0242	91241.36918	0.48461539
[TG(55:10),TG(54:3)]_C16:0	4529.588304	3931.361616	0.49740449
PC(14:0),LPC(15:0),LPC(O-16:0)	2477.912179	3088.788885	0.499039502
[TG(55:11),TG(54:4)]_C18:2	53729.60755	43149.67718	0.500394168
CE(22:5) NH4	4541.216325	5199.269714	0.514154746
[TG(52:4)]_C20:4	3814.532265	3337.572243	0.51470424
[TG(48:2)]_C16:0	17391.6172	15146.6438	0.521763141
CE(18:2) NH4	1269357.343	1194377.406	0.533678824
[TG(56:6)]_C22:5	2473.752168	2619.902172	0.537866
SM(d18:1/26:1(17Z))	3036.124213	2508.030853	0.551001778
[TG(55:10),TG(54:3)]_C18:2	20528.56958	21710.79276	0.560009598
[TG(54:11),TG(53:4)]_C18:2	3007.816232	3119.772878	0.56782083
PC(33:3),PC(O-34:3),PC(P-34:2)	9372.148661	10779.91874	0.573657878
[TG(51:8),TG(50:1)]_C14:0	3427.900245	3061.476854	0.592232275
LPI(20:0)	2087.300141	1768.710133	0.59919421
[TG(50:4)]_C16:0	2690.8522	2425.835512	0.630448815
[TG(57:12),TG(56:5)]_C20:4	2835.032219	2563.502185	0.65372865
[TG(54:11),TG(53:4)]_C18:1	1579.784108	1325.830755	0.655670398
SM(d16:1/23:0)	14968.26503	17873.68857	0.659418446
[TG(48:2)]_C18:2	13583.96091	12302.26092	0.673659983
CE(19:0)H	207317.0219	227694.6572	0.679169364
DG(39:8),DG(O-40:8)_C18:2	36697.24634	40232.51016	0.679951012
SM(d16:1/17:0)	16172.0491	19060.09274	0.68369457
PC(42:4)	2071.864156	1816.320793	0.688575346
[TG(50:4)]_C16:1	2957.900215	2719.683526	0.696095059
[TG(46:2)]_C16:1	2038.156152	1878.099462	0.70600673
[TG(50:3)]_C16:0	19366.7294	17903.19586	0.717904939
[TG(52:10),TG(51:3)]_C18:1	2912.972202	2686.542189	0.723682743
[TG(48:2)]_C16:1	5571.480366	5191.68368	0.744711286
PC(42:5)	1522.488093	1750.278129	0.751336256
PC(44:10),PC(O-44:3)	1378.560101	1219.683414	0.757420386
CE(22:6)Na	2710.800187	2668.053525	0.758817589
[TG(54:7)]_C18:3	3626.284264	3398.254251	0.764926532
PC(32:0),PC(O-33:0)	51274.60723	57503.03831	0.766534828
PC(34:0),PC(O-35:0)	41447.27512	46340.30522	0.771397247
[TG(57:9),TG(56:2)]_C18:1	2809.908188	2747.081515	0.776146758
PC(32:1),PC(O-33:1),PC(P-33:0)	35834.04254	39744.50598	0.787163066
[TG(56:6)]_C20:4	4914.584341	4648.160996	0.791000638
CE(20:2)K	5259.84034	4952.590324	0.79680695
PE(36:3),PE(P-37:2)	1699.236117	1560.572766	0.798424783
[TG(50:3)]_C14:0	9437.672664	10027.17599	0.799984965
[TG(54:6)]_C20:4	6176.220473	5850.693742	0.805061608
SM(d16:1/20:0)	71006.11227	77790.78998	0.812137556
[TG(54:10),TG(53:3)]_C18:1	4222.864306	4011.220927	0.812717322
[TG(53:10),TG(52:3)]_C16:1	8635.140569	8182.820543	0.815270938
[TG(50:3)]_C16:1	13439.2569	12763.60686	0.81861433
[TG(51:8)]_C18:2	4391.068305	4459.698299	0.829938647
PC(42:3)	2192.068153	2098.568146	0.835778552
[TG(51:9),TG(50:2)]_C18:0	2678.696202	2565.462832	0.842139783
[TG(50:5)]_C18:2	1321.832095	1225.408757	0.851778454

	[TG(53:9),TG(52:2)]_C18:2	25672.57769	26105.05778	0.853213134
	PC(30:0),PC(O-31:0)	15187.99701	16267.02372	0.855987956
	[TG(48:3)]_C16:1	2489.588172	2399.001492	0.867562738
	[TG(56:12),TG(55:5)]_C18:2	1161.252077	1218.05542	0.879124782
	[TG(57:10),TG(56:3)]_C18:1	3944.556283	4001.839602	0.879696296
	[TG(52:4)]_C18:3	11703.06069	11371.35404	0.894966928
	[TG(54:6)]_C18:2	22428.9616	23091.30511	0.903461089
	PC(40:8),PC(39:1),PC(O-40:1),PC(P-40:0)	5313.320372	5100.950361	0.905932647
	[TG(57:12),TG(56:5)]_C18:1	3306.352232	3221.992227	0.907056786
	[TG(52:5)]_C16:1	3385.092231	3477.678937	0.909154287
	[TG(53:10),TG(52:3)]_C18:2	154818.7563	150692.3178	0.909776097
	[TG(50:4)]_C18:2	7988.18055	8201.713975	0.910391928
	SM(d18:0/24:0)	8401.488634	8104.485267	0.91864024
	PI(36:2),PI(O-37:2),PI(P-37:1)	2926.704208	2829.44754	0.922502378
	[TG(48:3)]_C18:1	3237.368246	3296.811578	0.935259563
	[TG(54:5)]_C18:3	6720.57248	6847.445854	0.936305229
	CE(16:1) NH4	17220.85305	17516.58655	0.941239952
	[TG(46:2)]_C16:0	3361.344242	3312.854225	0.949450109
	SM(d18:0/15:0)	2843.98421	2782.6242	0.950520415
	[TG(55:11),TG(54:4)]_C18:1	95535.93858	96626.85224	0.962506652
	[TG(54:6)]_C18:3	6304.284417	6357.781087	0.970178769
	[TG(50:4)]_C14:0	4253.012293	4218.722313	0.971245461
	PC(41:6),PC(O-42:6)	2825.244204	2792.70754	0.973872807
	[TG(54:10),TG(53:3)]_C18:2	3100.996242	3083.559572	0.979984854
	PC(31:1),PC(O-32:1),PC(P-32:0)	6882.408476	6832.255152	0.983672653
	[TG(55:9),TG(54:2)]_C18:0	19670.80143	19645.3247	0.986908335
	[TG(56:7)]_C20:4	4364.364279	4369.974279	0.995521338

Table S8. Comparison of Lipid Corona Profiles Between Nanoparticle Sizes
Female 5% BC Samples

Unique Lipids in 50 nm BC	Unique Lipds in 100 nm BC	Shared Lipids
[TG(53:9),TG(52:2)]_C18:0	DG(37:7),DG(36:0)_C16:0	FA(22:7)
[TG(54:6)]_C18:2	DG(35:6)_C18:0	FA(18:0)
PS(25:0)	DG(40:5)_C18:0	FA(19:2)
DG(30:3)_C16:1	CE(18:1) NH4	FA(21:0)
FA(28:6)	DG(38:5)_C16:0	LPG(19:0),LPG(O-20:0); LPG(19:0),LPG(O-20:0)
[TG(53:7),TG(52:0)]_C16:0	DG(32:0)_C16:0	FA(20:0)
[TG(50:3)]_C18:2	DG(39:7),DG(38:0),DG(dO-40:0)_C18:0	PG(16:0),LPG(17:0),LPG(O-18:0); PG(16:0),LPG(17:0),LPG(O-18:0)
DG(30:2)_C16:1	DG(30:0)_C16:0	
[TG(49:6)]_C16:0	CE(18:2) NH4	
[TG(53:7)]_C18:1	DG(32:2)_C18:1	
[TG(55:11),TG(54:4)]_C18:2	DG(34:0)_C18:0	
[TG(51:8),TG(50:1)]_C18:0	CE(20:4) NH4	
[TG(54:5)]_C18:1	DG(33:0)_C16:0	
[TG(52:4)]_C16:1	CE(19:0)H	
[TG(42:0)]_C16:0	CE(18:2)Na	
CAR(20:0)	FA(17:2)	
[TG(53:9),TG(52:2)]_C18:2	CE(18:3) NH4	
[TG(51:9),TG(50:2)]_C16:0	CE(20:5)H	
[TG(51:7),TG(50:0)]_C16:0	DG(32:0)_C18:0	
FA(35:0)	DG(37:7),DG(36:0)_C18:0	
DG(36:8),DG(35:1)_C16:1	CE(16:0) NH4	
[TG(53:9),TG(52:2)]_C18:1	FA(6:0)	
FA(22:1)	DG(37:6)_C18:0	
[TG(55:9),TG(54:2)]_C18:1	FA(15:1)	
Cer(d18:0/17:0)	DG(34:1)_C16:0	
[TG(53:8),TG(52:1)]_C16:0	DG(35:6)_C16:0	
[TG(55:10),TG(54:3)]_C18:1	DG(34:3)_C18:1	
[TG(51:9),TG(50:2)]_C16:1	CE(22:6) NH4	
DG(36:7),DG(35:0)_C16:0	DG(34:1)_C18:1	
[TG(52:4)]_C18:1	DG(42:5)_C18:0	
[TG(49:7),TG(48:0)]_C16:0	DG(34:0)_C16:0	
[TG(49:8),TG(48:1)]_C14:0	DG(40:5)_C16:0	
DG(41:6)_C16:1	DG(36:7),DG(35:0)_C18:0	
[TG(49:7),TG(48:0)]_C18:0	CE(20:5) NH4	
[TG(51:8),TG(50:1)]_C16:0	DG(34:4),DG(dO-36:4)_C16:1	
[TG(55:9),TG(54:2)]_C18:0	DG(O-40:9),DG(38:2)_C18:2	
PC(36:4),PC(O-37:4)		
CAR(18:3)		
[TG(53:10),TG(52:3)]_C18:2		
[TG(51:9),TG(50:2)]_C18:2		
[TG(53:7),TG(52:0)]_C18:0		
PC(34:2),PC(O-35:2),PC(P-35:1)		
[TG(49:8),TG(48:1)]_C16:0		
DG(41:5)_C16:0		
[TG(52:4)]_C18:2		
CE(15:1) NH4		
[TG(49:7),TG(48:0)]_C14:0		
[TG(55:10),TG(54:3)]_C18:0		
[TG(53:10),TG(52:3)]_C18:1		
[TG(51:8),TG(50:1)]_C18:1		
Cer(d14:2(4E,6E)/16:0)		
[TG(53:8),TG(52:1)]_C18:1		
PC(40:6)		
[TG(51:7),TG(50:0)]_C18:0		
[TG(50:7),TG(49:0)]_C16:0		
DG(42:11),DG(41:4)_C16:0		
[TG(54:5)]_C18:2		
[TG(55:10),TG(54:3)]_C18:2		
[TG(53:8),TG(52:1)]_C18:0		
[TG(55:8),TG(54:1)]_C18:1		
[TG(53:10),TG(52:3)]_C16:0		
[TG(48:2)]_C18:1		
PC(38:6)		
FA(18:3)		
[TG(55:8),TG(54:1)]_C18:0		
[TG(51:9),TG(50:2)]_C14:0		
FA(22:0)		
[TG(52:4)]_C16:0		
[TG(52:5)]_C18:2		
[TG(48:2)]_C16:1		
[TG(50:3)]_C18:1		
[TG(53:9),TG(52:2)]_C16:0		
PS(O-29:0)		
DG(30:2)_C16:0		
[TG(51:9),TG(50:2)]_C18:1		
[TG(50:3)]_C16:1		
DG(36:6)_C16:0		
[TG(53:10),TG(52:3)]_C16:1		

[TG(55:11),TG(54:4)]_C18:1		
[TG(46:0)]_C16:0		
DG(36:7)_C16:1		
[TG(55:11),TG(54:4)]_C18:0		

Table S8. Comparison of Lipid Corona Profiles Between Nanoparticle Sizes
Female 10% BC Samples

Unique Lipids in 50 nm BC	Unique Lipds in 100 nm BC	Shared Lipids
[TG(53:9),TG(52:2)]_C18:0	DG(34:2)_C18:2	FA(17:2)
[TG(54:6)]_C18:2	DG(40:5)_C18:0	FA(22:7)
FA(21:1)	CE(18:1) NH4	FA(21:0)
FA(28:6)	DG(39:8),DG(O-40:8)_C18:2	LPG(19:0),LPG(O-20:0); LPG(19:0),LPG(O-20:0)
[TG(53:7),TG(52:0)]_C16:0	DG(38:5)_C16:0	FA(20:0)
[TG(50:3)]_C18:2	DG(30:0)_C16:0	PG(16:0),LPG(17:0),LPG(O-18:0); PG(16:0),LPG(17:0),LPG(O-18:0)
DG(30:2)_C16:1	DG(32:2)_C18:1	
[TG(49:6)]_C16:0	CE(20:4) NH4	
[TG(53:7)]_C18:1	DG(33:0)_C16:0	
[TG(51:8),TG(50:1)]_C18:0	CE(18:2)Na	
[TG(52:4)]_C16:1	CE(22:2) NH4	
[TG(52:4)]_C18:3	CE(16:0) NH4	
FA(19:0)	DG(34:0)_C16:0	
[TG(51:7),TG(50:0)]_C16:0	DG(40:5)_C16:0	
FA(35:0)	CE(20:5) NH4	
DG(36:8),DG(35:1)_C16:1	DG(34:4),DG(dO-36:4)_C16:1	
PC(O-38:9),PC(36:2),PC(O-37:2),PC(P-37:1)	DG(39:8),DG(O-40:8),DG(38:1)_C18:1	
PC(32:0),PC(O-33:0)	DG(37:7),DG(36:0)_C16:0	
[TG(53:9),TG(52:2)]_C18:1	DG(35:6)_C18:0	
[TG(55:9),TG(54:2)]_C18:1	DG(32:0)_C16:0	
[TG(51:9),TG(50:2)]_C16:1	CE(18:2) NH4	
DG(36:7),DG(35:0)_C16:0	CE(18:0)K	
PC(36:5)	DG(34:0)_C18:0	
[TG(52:4)]_C18:1	CE(16:0)Na	
[TG(49:8),TG(48:1)]_C14:0	CE(19:0)H	
[TG(51:8),TG(50:1)]_C16:0	CE(18:3) NH4	
[TG(55:9),TG(54:2)]_C18:0	CE(20:5)H	
[TG(54:10),TG(53:3)]_C18:1	DG(32:0)_C18:0	
[TG(53:10),TG(52:3)]_C18:2	DG(37:7),DG(36:0)_C18:0	
FA(26:6)	DG(37:6)_C18:0	
[TG(51:9),TG(50:2)]_C18:2	FA(15:1)	
[TG(53:7),TG(52:0)]_C18:0	DG(34:1)_C16:0	
DG(32:5)_C18:1	DG(35:6)_C16:0	
[TG(52:5)]_C18:3	DG(34:3)_C18:1	
DG(41:5)_C16:0	CE(22:6) NH4	
[TG(49:8),TG(48:1)]_C16:0	DG(34:1)_C18:1	
[TG(52:4)]_C18:2	DG(42:5)_C18:0	
[TG(55:10),TG(54:3)]_C18:0	DG(O-40:9),DG(38:2)_C18:2	
[TG(50:3)]_C16:0		
[TG(53:10),TG(52:3)]_C18:1		
Cer(d14:2(4E,6E)/16:0)		
FA(16:0)		
[TG(53:8),TG(52:1)]_C18:1		
DG(42:11),DG(41:4)_C16:0		
[TG(55:10),TG(54:3)]_C18:2		
[TG(55:8),TG(54:1)]_C18:1		
[TG(55:8),TG(54:1)]_C18:0		
PG(20:0),LPG(21:0); PG(20:0),LPG(21:0)		
[TG(52:4)]_C16:0		
PC(36:3),PC(P-37:2)		
DG(36:7)_C16:1		
PS(25:0)		
DG(30:3)_C16:1		
[TG(39:0)]_C20:0		
[TG(55:11),TG(54:4)]_C18:2		
[TG(54:5)]_C18:1		
[TG(50:3)]_C14:0		
[TG(49:8),TG(48:1)]_C18:1		
CAR(20:0)		
[TG(51:9),TG(50:2)]_C16:0		
[TG(53:9),TG(52:2)]_C18:2		
[TG(54:9),TG(53:2)]_C18:1		
FA(18:0)		
FA(22:1)		
[TG(53:8),TG(52:1)]_C16:0		
[TG(55:10),TG(54:3)]_C18:1		
PC(34:1),PC(O-35:1),PC(P-35:0)		
[TG(49:7),TG(48:0)]_C16:0		
[TG(49:7),TG(48:0)]_C18:0		
DG(41:6)_C16:1		
FA(6:0)		
PC(36:4),PC(O-37:4)		
FA(19:2)		
SM(d16:0/22:0)		
PC(34:2),PC(O-35:2),PC(P-35:1)		
CE(15:1) NH4		
[TG(51:8),TG(50:1)]_C18:1		
PC(38:4)		
PC(40:6)		
[TG(51:7),TG(50:0)]_C18:0		
[TG(50:7),TG(49:0)]_C16:0		
[TG(54:5)]_C18:2		
[TG(52:9),TG(51:2)]_C18:1		

[TG(53:8),TG(52:1)]_C18:0		
[TG(53:10),TG(52:3)]_C16:0		
PC(38:6)		
FA(22:0)		
[TG(51:9),TG(50:2)]_C14:0		
[TG(52:5)]_C18:2		
[TG(50:3)]_C18:1		
[TG(53:9),TG(52:2)]_C16:0		
PS(O-29:0)		
DG(30:2)_C16:0		
FA(28:0)		
[TG(51:9),TG(50:2)]_C18:1		
[TG(50:3)]_C16:1		
DG(36:6)_C16:0		
[TG(53:10),TG(52:3)]_C16:1		
[TG(40:0)]_C16:0		
DG(36:5)_C16:0		
[TG(55:11),TG(54:4)]_C18:1		
[TG(46:0)]_C16:0		
[TG(55:11),TG(54:4)]_C18:0		
PC(38:5)		

Table S8. Comparison of Lipid Corona Profiles Between Nanoparticle Sizes
Female 25% BC Samples

Unique Lipids in 50 nm BC	Unique Lipds in 100 nm BC	Shared Lipids
PC(38:3)	CE(20:0) NH4	PG(16:0),LPG(17:0),LPG(O-18:0); PG(16:0),LPG(17:0),LPG(O-18:0)
[TG(53:9),TG(52:2)]_C18:0	DG(34:2)_C18:2	
SM(d16:1/17:0)	DG(40:5)_C18:0	
[TG(54:6)]_C18:2	CE(18:1) NH4	
PC(33:2),PC(O-34:2),PC(P-34:1)	DG(39:8),DG(O-40:8)_C18:2	
[TG(53:7),TG(52:0)]_C16:0	DG(38:5)_C16:0	
[TG(50:3)]_C18:2	CE(15:1)K	
[TG(57:12),TG(56:5)]_C18:1	CE(18:3)H	
PC(35:2),PC(O-36:2),PC(P-36:1)	DG(30:0)_C16:0	
DG(30:2)_C16:1	DG(32:2)_C18:1	
[TG(49:6)]_C16:0	DG(O-38:8),DG(36:1)_C16:1	
[TG(53:7)]_C18:1	CE(20:4) NH4	
[TG(51:8),TG(50:1)]_C18:0	DG(36:3)_C18:1	
[TG(52:4)]_C16:1	DG(33:0)_C16:0	
[TG(54:5)]_C18:3	CE(18:2)Na	
[TG(52:4)]_C18:3	CE(22:2) NH4	
[TG(54:5)]_C16:0	CE(16:0) NH4	
PC(O-38:8),PC(36:1),PC(O-37:1),PC(P-37:0)	CE(20:3) NH4	
SM(d16:1/16:0)	CE(18:3)Na	
[TG(55:11),TG(54:4)]_C16:0	PG(20:0),LPG(21:0); PG(20:0),LPG(21:0)	
[TG(55:9),TG(54:2)]_C16:0	DG(34:0)_C16:0	
[TG(51:7),TG(50:0)]_C16:0	DG(40:5)_C16:0	
FA(35:0)	CE(20:5) NH4	
DG(36:8),DG(35:1)_C16:1	DG(34:4),DG(dO-36:4)_C16:1	
PC(O-38:9),PC(36:2),PC(O-37:2),PC(P-37:1)	DG(39:8),DG(O-40:8),DG(38:1)_C18:1	
PC(32:0),PC(O-33:0)	DG(37:7),DG(36:0)_C16:0	
[TG(53:9),TG(52:2)]_C18:1	DG(35:6)_C18:0	
[TG(52:8),TG(51:1)]_C16:0	DG(32:0)_C16:0	
[TG(57:11),TG(56:4)]_C18:1	DG(39:7),DG(38:0),DG(dO-40:0)_C18:0	
[TG(55:9),TG(54:2)]_C18:1	CE(18:2) NH4	
[TG(48:2)]_C14:0	CE(18:0)K	
[TG(51:9),TG(50:2)]_C16:1	DG(34:0)_C18:0	
DG(36:7),DG(35:0)_C16:0	CE(22:1)H	
[TG(52:4)]_C18:1	CE(16:0)Na	
SM(d16:1/18:1)	CE(19:0)H	
PC(36:5)	CE(18:3) NH4	
[TG(49:8),TG(48:1)]_C14:0	CE(20:5)H	
PC(30:0),PC(O-31:0)	DG(32:0)_C18:0	
[TG(57:12),TG(56:5)]_C20:4	DG(37:7),DG(36:0)_C18:0	
[TG(51:8),TG(50:1)]_C16:0	DG(37:6)_C18:0	
[TG(55:9),TG(54:2)]_C18:0	DG(34:1)_C16:0	
SM(d16:1/24:0)	CE(16:1)Na	
[TG(54:10),TG(53:3)]_C18:1	CE(16:1) NH4	
[TG(53:10),TG(52:3)]_C18:2	DG(35:6)_C16:0	
Cer(d18:1/24:0)	DG(36:4),DG(O-37:4)_C18:2	
[TG(54:6)]_C20:4	DG(34:3)_C18:1	
SM(d18:2/22:1)	CE(22:6) NH4	
[TG(53:7),TG(52:0)]_C18:0	CE(16:0)K	
PC(40:10),PC(39:3),PC(O-40:3),PC(P-40:2)	DG(34:1)_C18:1	
[TG(51:9),TG(50:2)]_C18:2	DG(42:5)_C18:0	
[TG(52:5)]_C16:0	DG(36:7),DG(35:0)_C18:0	
[TG(52:5)]_C18:3	DG(O-38:9),DG(36:2)_C18:1	
[TG(50:4)]_C18:2	DG(O-40:9),DG(38:2)_C18:2	
[TG(48:2)]_C18:2		
[TG(49:8),TG(48:1)]_C16:1		
[TG(56:6)]_C20:4		
DG(41:5)_C16:0		
[TG(49:8),TG(48:1)]_C16:0		
[TG(52:4)]_C18:2		
SM(d18:1/17:0)		
[TG(55:10),TG(54:3)]_C18:0		
[TG(50:3)]_C16:0		
[TG(53:10),TG(52:3)]_C18:1		
[TG(55:10),TG(54:3)]_C16:0		
Cer(d14:2(4E,6E)/16:0)		
[TG(53:8),TG(52:1)]_C18:1		
PC(29:1),PC(O-30:1),PC(P-30:0)		
DG(42:11),DG(41:4)_C16:0		
PC(28:0),PC(O-29:0)		
[TG(55:10),TG(54:3)]_C18:2		
[TG(55:8),TG(54:1)]_C18:1		
[TG(48:2)]_C16:0		
[TG(55:8),TG(54:1)]_C18:0		
SM(d16:0/20:0)		
[TG(52:4)]_C16:0		
PC(40:4)		
DG(36:6)_C16:1		
PC(36:3),PC(P-37:2)		
PC(35:4),PC(O-36:4),PC(P-36:3)		
LPC(18:0),PC(O-18:0),LPC(O-19:0)		
[TG(50:4)]_C16:1		
DG(36:7)_C16:1		
DG(30:1)_C16:0		
[TG(45:2)]_C16:0		

PC(28:1),PC(P-29:0)		
PS(25:0)		
DG(30:3)_C16:1		
PC(38:9),PC(37:2),PC(O-38:2),PC(P-38:1)		
PC(32:1),PC(O-33:1),PC(P-33:0)		
[TG(53:10),TG(52:3)]_C18:0		
[TG(39:0)]_C20:0		
SM(d16:1/22:1)		
PC(36:8),PC(35:1),PC(O-36:1),PC(P-36:0)		
[TG(55:11),TG(54:4)]_C18:2		
[TG(56:6)]_C22:5		
PC(37:7),PC(P-38:6),PC(36:0),PC(O-37:0)		
[TG(54:6)]_C18:1		
LPG(20:0); LPG(20:0)		
SM(d16:1/22:0)		
[TG(54:5)]_C18:1		
[TG(50:3)]_C14:0		
PC(30:1),PC(O-31:1),PC(P-31:0)		
[TG(49:8),TG(48:1)]_C18:1		
CAR(20:0)		
[TG(51:9),TG(50:2)]_C16:0		
[TG(53:9),TG(52:2)]_C18:2		
[TG(56:7)]_C18:2		
SM(d16:0/18:0)		
[TG(51:8),TG(50:1)]_C16:1		
FA(22:7)		
[TG(54:9),TG(53:2)]_C18:1		
PC(33:1),PC(O-34:1),PC(P-34:0)		
SM(d16:0/23:0)		
PC(34:0),PC(O-35:0)		
PC(40:5)		
FA(30:0)		
[TG(53:8),TG(52:1)]_C16:0		
Cer(d18:0/17:0)		
[TG(55:10),TG(54:3)]_C18:1		
[TG(54:10),TG(53:3)]_C18:2		
PC(37:5),PC(O-38:5),PC(P-38:4)		
PC(34:1),PC(O-35:1),PC(P-35:0)		
[TG(49:7),TG(48:0)]_C16:0		
DG(41:6)_C16:1		
[TG(49:7),TG(48:0)]_C18:0		
[TG(51:8),TG(50:1)]_C14:0		
SM(d18:1/19:0)		
[TG(56:7)]_C22:6		
PC(36:4),PC(O-37:4)		
PC(31:0),PC(O-32:0)		
SM(d16:0/22:0)		
PC(34:2),PC(O-35:2),PC(P-35:1)		
[TG(44:1)]_C16:0		
[TG(54:6)]_C18:3		
CE(15:1) NH4		
[TG(52:5)]_C16:1		
PC(35:3),PC(O-36:3),PC(P-36:2)		
SM(d16:1/24:1)		
[TG(51:8),TG(50:1)]_C18:1		
PC(31:1),PC(O-32:1),PC(P-32:0)		
PC(38:4)		
FA(21:0)		
[TG(50:7),TG(49:0)]_C16:0		
PC(40:6)		
[TG(51:7),TG(50:0)]_C18:0		
[TG(54:5)]_C18:2		
[TG(52:9),TG(51:2)]_C18:1		
PC(40:8),PC(39:1),PC(O-40:1),PC(P-40:0)		
SM(d16:1/20:1)		
[TG(51:7)]_C18:1		
[TG(53:8),TG(52:1)]_C18:0		
[TG(53:10),TG(52:3)]_C16:0		
[TG(48:2)]_C18:1		
PC(38:6)		
[TG(55:9),TG(54:2)]_C18:2		
SM(d16:1/18:0)		
SM(d16:1/20:0)		
FA(22:0)		
[TG(52:9),TG(51:2)]_C16:0		
[TG(51:9),TG(50:2)]_C14:0		
SM(d18:2/24:1)		
PC(37:3),PC(O-38:3),PC(P-38:2)		
PC(37:4),PC(O-38:4),PC(P-38:3)		
[TG(48:2)]_C16:1		
[TG(52:5)]_C18:2		
[TG(50:3)]_C18:1		
PS(O-29:0)		
[TG(53:9),TG(52:2)]_C16:0		
[TG(52:10),TG(51:3)]_C18:2		
DG(30:2)_C16:0		
SM(d16:0/24:0)		
PC(30:2),PC(P-31:1)		

[TG(51:9),TG(50:2)]_C18:1		
[TG(53:9),TG(52:2)]_C16:1		
[TG(50:3)]_C16:1		
[TG(56:7)]_C20:4		
[TG(54:5)]_C20:4		
DG(36:6)_C16:0		
[TG(57:10),TG(56:3)]_C18:1		
[TG(53:10),TG(52:3)]_C16:1		
DG(36:5)_C16:0		
[TG(55:11),TG(54:4)]_C18:1		
FA(20:0)		
[TG(46:0)]_C16:0		
[TG(55:11),TG(54:4)]_C18:0		
FA(37:0)		
PC(38:5)		

Table S8. Comparison of Lipid Corona Profiles Between Nanoparticle Sizes
Female 50% BC Samples

Unique Lipids in 50 nm BC	Unique Lipds in 100 nm BC	Shared Lipids
SM(d18:1/12:0)	CE(20:5)Na	PE(38:4)
SM(d18:0/17:0)	DG(34:2)_C18:2	CE(18:1) NH4
PC(44:10),PC(O-44:3)	CE(15:1)K	DG(39:8),DG(O-40:8)_C18:2
[TG(54:5)]_C18:0	CE(18:3)H	PC(35:2),PC(O-36:2),PC(P-36:1)
PI(38:3)	DG(30:0)_C16:0	PS(38:4)
[TG(59:13),TG(58:6)]_C18:1	DG(32:2)_C18:1	PC(39:7),PC(P-40:6),PC(38:0),PC(O-39:0)
[TG(54:6)]_C18:2	DG(36:3)_C18:1	CE(22:5)H
PI(36:2),PI(O-37:2),PI(P-37:1)	CE(18:2)Na	CE(22:5) NH4
Cer(d18:1/22:0)	CE(16:0) NH4	[TG(56:8)]_C22:6
[TG(54:11),TG(53:4)]_C18:2	CE(20:3) NH4	CE(20:2)Na
SM(d18:0/26:1(17Z))	DG(O-38:8),DG(36:1)_C18:1	LPG(19:0),LPG(O-20:0); LPG(19:0),LPG(O-20:0)
PC(33:2),PC(O-34:2),PC(P-34:1)	CE(22:1) NH4	CE(16:3)Na
[TG(53:7),TG(52:0)]_C16:0	DG(39:7)_C18:1	CE(22:6) NH4
[TG(57:12),TG(56:5)]_C18:1	DG(O-38:9),DG(36:2)_C18:2	DG(O-40:9),DG(38:2)_C18:2
[TG(56:12),TG(55:5)]_C18:1	CE(18:3)Na	CE(20:0) NH4
PC(39:8),PC(O-40:8),PC(38:1),PC(O-39:1),PC(P-39:0)	DG(34:0)_C16:0	PC(39:5),PC(O-40:5),PC(P-40:4)
DG(30:2)_C16:1	CE(20:2) NH4	DG(39:8),DG(O-40:8),DG(38:1)_C18:1
[TG(57:12),TG(56:5)]_C16:0	DG(40:5)_C16:0	PC(34:3),PC(P-35:2)
LPI(20:0)	CE(20:5) NH4	CE(18:2) NH4
[TG(52:4)]_C16:1	CE(22:3)H	CE(19:0)H
[TG(53:7),TG(52:0)]_C20:0	DG(37:7),DG(36:0)_C16:0	PE(34:2),PE(O-35:2),PE(P-35:1)
[TG(54:5)]_C18:3	DG(34:2)_C18:1	PC(34:2),PC(O-35:2),PC(P-35:1)
[TG(52:4)]_C18:3	CE(20:5)K	PC(38:6)
[TG(44:1)]_C18:1	DG(32:0)_C16:0	CE(20:1) NH4
[TG(50:9),TG(49:2)]_C16:0	CE(14:0) NH4	CE(16:0)K
SM(d16:1/16:0)	CE(20:0)H	CE(20:2)K
PC(39:4),PC(O-40:4),PC(P-40:3)	DG(39:7),DG(38:0),DG(dO-40:0)_C18:0	PG(16:0),LPG(17:0),LPG(O-18:0); PG(16:0),LPG(17:0),LPG(O-18:0)
[TG(50:9),TG(49:2)]_C18:1	CE(18:0)K	
[TG(51:7),TG(50:0)]_C16:0	DG(34:0)_C18:0	
FA(35:0)	CE(16:0)Na	
PC(O-38:9),PC(36:2),PC(O-37:2),PC(P-37:1)	CE(18:3) NH4	
[TG(52:8),TG(51:1)]_C16:0	DG(32:0)_C18:0	
PC(35:5),PC(O-36:5),PC(P-36:4)	DG(37:6)_C18:0	
PC(31:2),PC(O-32:2),PC(P-32:1)	FA(15:1)	
[TG(57:11),TG(56:4)]_C18:1	DG(34:1)_C16:0	
[TG(38:0)]_C18:0	CE(16:1)Na	
LPC(16:0),PC(O-16:0),LPC(O-17:0)	DG(37:6)_C16:0	
[TG(46:0)]_C18:0	CE(16:1) NH4	
[TG(48:2)]_C14:0	DG(O-40:9),DG(38:2)_C18:1	
[TG(52:4)]_C20:4	DG(36:4),DG(O-37:4)_C18:2	
[TG(54:10),TG(53:3)]_C16:0	DG(O-38:9),DG(36:2)_C18:1	
[TG(51:9),TG(50:2)]_C16:1	DG(36:7),DG(35:0)_C18:0	
PC(42:5)	CE(18:1)K	
[TG(52:4)]_C18:1	DG(40:5)_C18:0	
[TG(49:8),TG(48:1)]_C14:0	CE(19:0)K	
PC(43:4),PC(O-44:4)	CE(18:3)K	
PC(30:0),PC(O-31:0)	CE(22:6)H	
[TG(57:12),TG(56:5)]_C20:4	DG(38:5)_C16:0	
PC(40:1),PC(P-41:0)	CE(18:0) NH4	
[TG(46:1)]_C18:1	DG(38:3)_C18:2	
[TG(55:9),TG(54:2)]_C18:0	DG(O-38:8),DG(36:1)_C16:1	
SM(d16:1/24:0)	CE(18:2)K	
[TG(54:10),TG(53:3)]_C18:1	CE(20:4) NH4	
[TG(52:6)]_C18:2	DG(33:0)_C16:0	
[TG(53:10),TG(52:3)]_C18:2	CE(22:2) NH4	
[TG(54:11),TG(53:4)]_C16:0	DG(O-38:8),DG(36:1)_C18:0	
PC(36:7),PC(35:0),PC(O-36:0)	CE(20:0)Na	
SM(d16:0/25:0)	CE(22:3) NH4	
Cer(d18:1/24:0)	DG(40:9),DG(39:2)_C18:2	
[TG(53:7),TG(52:0)]_C18:0	CE(20:4)H	
DG(32:5)_C18:1	DG(34:4),DG(dO-36:4)_C16:1	
Cer(d18:1/23:0)	CE(18:1)Na	
[TG(52:5)]_C16:0	PS(P-37:0)	
[TG(52:5)]_C18:3	DG(35:6)_C18:0	
[TG(56:6)]_C20:4	CE(19:0) NH4	
[TG(49:8),TG(48:1)]_C16:1	CE(16:2)Na	
LPC(22:4)	CE(22:4)Na	
[TG(56:11),TG(55:4)]_C18:2	CE(22:1)H	
[TG(49:8),TG(48:1)]_C16:0	CE(20:5)H	
[TG(55:10),TG(54:3)]_C18:0	DG(34:2)_C16:0	
[TG(58:9)]_C22:6	DG(37:7),DG(36:0)_C18:0	
CAR(14:1)	DG(36:3)_C18:2	
PC(39:6),PC(O-40:6),PC(P-40:5)	CE(20:4)Na	
PI(38:4)	DG(35:6)_C16:0	
[TG(54:8),TG(53:1)]_C18:1	DG(34:3)_C18:1	
[TG(52:10),TG(51:3)]_C18:1	DG(34:1)_C18:1	
[TG(53:8),TG(52:1)]_C18:1	DG(42:5)_C18:0	
DG(42:11),DG(41:4)_C16:0	CE(19:0)Na	
[TG(55:7)]_C18:1		
[TG(55:10),TG(54:3)]_C18:2		
[TG(48:3)]_C18:2		
SM(d16:1/25:0)		
[TG(51:8)]_C18:2		

[TG(55:8),TG(54:1)]_C18:1		
PC(41:6),PC(O-42:6)		
[TG(48:2)]_C16:0		
[TG(55:8),TG(54:1)]_C18:0		
[TG(56:12),TG(55:5)]_C18:2		
PE(38:6)		
DG(36:6)_C16:1		
SM(d16:1/23:0)		
[TG(54:12),TG(53:5)]_C18:2		
[TG(46:1)]_C16:0		
[TG(56:8)]_C18:2		
[TG(56:6)]_C18:2		
PC(42:1)		
PC(36:3),PC(P-37:2)		
SM(d18:2/14:0)		
[TG(50:4)]_C16:1		
LPC(18:0),PC(O-18:0),LPC(O-19:0)		
DG(30:1)_C16:0		
PC(37:6),PC(O-38:6),PC(P-38:5)		
[TG(44:0),TG(O-45:0)]_C16:0		
Cer(d18:1/24:1(15Z))		
SM(d17:0/27:0)		
[TG(56:11),TG(55:4)]_C18:1		
[TG(54:9),TG(53:2)]_C18:0		
[TG(50:8),TG(49:1)]_C18:1		
[TG(38:1)]_C18:1		
PC(32:1),PC(O-33:1),PC(P-33:0)		
[TG(56:8)]_C20:4		
[TG(53:10),TG(52:3)]_C18:0		
[TG(39:0)]_C20:0		
[TG(55:11),TG(54:4)]_C18:2		
[TG(57:8),TG(56:1)]_C20:0		
[TG(54:6)]_C18:1		
LPG(20:0); LPG(20:0)		
[TG(50:3)]_C14:0		
[TG(50:4)]_C18:1		
[TG(49:8),TG(48:1)]_C18:1		
[TG(49:8)]_C18:2		
[TG(53:9),TG(52:2)]_C18:2		
PC(42:6)		
[TG(52:7),TG(51:0)]_C18:0		
[TG(55:7),TG(54:0)]_C20:0		
[TG(56:7)]_C18:2		
[TG(51:8),TG(50:1)]_C16:1		
SM(d16:0/18:0)		
[TG(54:9),TG(53:2)]_C18:1		
[TG(54:7)]_C20:4		
PC(34:0),PC(O-35:0)		
[TG(54:11),TG(53:4)]_C18:1		
SM(d18:0/15:0)		
PC(40:5)		
SM(d18:2/18:1)		
[TG(53:8),TG(52:1)]_C16:0		
LPC(20:4)		
[TG(56:7),TG(55:0)]_C16:0		
PC(42:2)		
[TG(58:8),TG(57:1)]_C18:1		
[TG(57:10),TG(56:3)]_C18:0		
PC(34:1),PC(O-35:1),PC(P-35:0)		
SM(d17:1/24:1)		
PC(32:2),PC(O-33:2),PC(P-33:1)		
[TG(51:8),TG(50:1)]_C14:0		
SM(d18:1/19:0)		
PC(44:12),PC(O-44:5)		
[TG(56:7)]_C22:6		
[TG(50:4)]_C18:3		
[TG(56:6)]_C16:0		
[TG(48:3)]_C16:1		
PC(19:1),LPC(20:1),PC(O-20:1),PC(P-20:0)		
[TG(54:7)]_C18:2		
[TG(52:6)]_C16:1		
PC(28:2)		
PC(35:6),PC(P-36:5)		
PC(32:3),PC(P-33:2)		
[TG(54:8),TG(53:1)]_C18:0		
SM(d17:1/26:1)		
[TG(53:10),TG(52:3)]_C18:3		
[TG(54:6)]_C18:3		
CE(15:1) NH4		
[TG(57:12),TG(56:5)]_C22:5		
[TG(52:5)]_C16:1		
[TG(48:4)]_C18:2		
PC(35:3),PC(O-36:3),PC(P-36:2)		
[TG(55:9),TG(54:2)]_C20:0		
[TG(52:9),TG(51:2)]_C18:2		
[TG(57:9),TG(56:2)]_C18:0		
PC(38:4)		

[TG(57:10),TG(56:3)]_C20:0		
[TG(50:7),TG(49:0)]_C16:0		
[TG(54:5)]_C18:2		
PC(O-40:9),PC(38:2),PC(P-39:1)		
SM(d16:1/20:1)		
[TG(51:7)]_C18:1		
[TG(53:10),TG(52:3)]_C16:0		
FA(24:4)		
[TG(48:2)]_C18:1		
PC(42:10),PC(41:3),PC(O-42:3),PC(P-42:2)		
[TG(44:2)]_C16:0		
SM(d16:1/20:0)		
[TG(51:9),TG(50:2)]_C14:0		
[TG(57:9),TG(56:2)]_C18:1		
SM(d18:2/24:1)		
SM(d16:0/16:0)		
PC(37:4),PC(O-38:4),PC(P-38:3)		
PC(42:9),PC(41:2),PC(O-42:2),PC(P-42:1)		
[TG(52:5)]_C18:2		
[TG(37:0)]_C18:0		
[TG(50:3)]_C18:1		
SM(d18:0/24:0)		
[TG(57:11),TG(56:4)]_C16:0		
[TG(52:10),TG(51:3)]_C18:2		
SM(d16:0/24:0)		
CAR(14:2)		
[TG(48:3)]_C18:1		
[TG(58:8)]_C22:5		
[TG(54:5)]_C20:4		
[TG(53:10),TG(52:3)]_C16:1		
[TG(46:0)]_C14:0		
DG(36:5)_C16:0		
[TG(58:7)]_C22:5		
[TG(46:0)]_C16:0		
PC(40:7),PC(39:0),PC(O-40:0)		
PC(42:11),PC(41:4),PC(O-42:4)		
[TG(55:11),TG(54:4)]_C18:0		
PC(38:5)		
LPC(18:2),LPC(P-19:1)		
PC(38:3)		
[TG(58:8)]_C22:6		
[TG(46:1)]_C14:0		
SM(d16:1/17:0)		
[TG(53:9),TG(52:2)]_C18:0		
[TG(56:9),TG(55:2)]_C18:1		
[TG(54:5)]_C22:5		
[TG(48:3)]_C16:0		
[TG(53:8),TG(52:1)]_C16:1		
[TG(50:3)]_C18:2		
[TG(49:6)]_C16:0		
[TG(53:7)]_C18:1		
[TG(51:8),TG(50:1)]_C18:0		
[TG(52:8),TG(51:1)]_C18:1		
[TG(57:9),TG(56:2)]_C20:0		
[TG(57:11),TG(56:4)]_C18:0		
[TG(50:4)]_C14:0		
SM(d18:2/15:0)		
[TG(42:0)]_C16:0		
[TG(54:5)]_C16:0		
PC(O-38:8),PC(36:1),PC(O-37:1),PC(P-37:0)		
[TG(55:11),TG(54:4)]_C16:0		
PC(29:0),PC(O-30:0)		
[TG(55:9),TG(54:2)]_C16:0		
DG(36:8),DG(35:1)_C16:1		
PC(32:0),PC(O-33:0)		
[TG(52:5)]_C20:4		
[TG(53:9),TG(52:2)]_C18:1		
[TG(54:9),TG(53:2)]_C18:2		
[TG(52:7),TG(51:0)]_C16:0		
[TG(55:9),TG(54:2)]_C18:1		
[TG(49:8),TG(48:1)]_C18:0		
DG(36:7),DG(35:0)_C16:0		
SM(d16:1/18:1)		
PC(36:5)		
PC(42:3)		
[TG(53:8),TG(52:1)]_C20:0		
[TG(54:6)]_C16:0		
[TG(56:10),TG(55:3)]_C18:1		
[TG(51:8),TG(50:1)]_C16:0		
[TG(55:11),TG(54:4)]_C20:4		
[TG(57:12),TG(56:5)]_C18:0		
[TG(57:11),TG(56:4)]_C20:0		
LPC(20:2),PC(O-20:2)		
SM(d18:2/22:1)		
[TG(54:6)]_C20:4		
[TG(49:7)]_C18:1		
PC(40:10),PC(39:3),PC(O-40:3),PC(P-40:2)		

[TG(51:9),TG(50:2)]_C18:2		
[TG(50:4)]_C18:2		
PC(38:8),PC(37:1),PC(O-38:1),PC(P-38:0)		
[TG(48:2)]_C18:2		
PC(30:3)		
DG(41:5)_C16:0		
[TG(54:8),TG(53:1)]_C16:0		
[TG(56:6)]_C18:0		
[TG(52:4)]_C18:2		
SM(d18:1/17:0)		
[TG(52:10),TG(51:3)]_C16:0		
[TG(50:3)]_C16:0		
[TG(53:10),TG(52:3)]_C18:1		
[TG(54:7)]_C18:3		
PC(38:7),PC(37:0),PC(O-38:0)		
PC(42:4)		
Cer(d14:2(4E,6E)/16:0)		
[TG(55:10),TG(54:3)]_C16:0		
DG(36:8),DG(35:1)_C18:1		
PC(29:1),PC(O-30:1),PC(P-30:0)		
[TG(52:5)]_C18:1		
PC(28:0),PC(O-29:0)		
SM(d18:2/21:0)		
PC(34:6)		
[TG(51:4)]_C18:2		
SM(d16:0/20:0)		
[TG(58:7)]_C18:1		
PC(40:4)		
[TG(52:4)]_C16:0		
[TG(50:4)]_C16:0		
[TG(49:3)]_C18:2		
PC(35:4),PC(O-36:4),PC(P-36:3)		
[TG(50:8),TG(49:1)]_C16:0		
[TG(55:8),TG(54:1)]_C16:0		
PC(40:2)		
DG(36:7)_C16:1		
PC(41:7),PC(P-42:6),PC(40:0),PC(O-41:0)		
[TG(42:0)]_C14:0		
PC(40:3)		
PC(28:1),PC(P-29:0)		
PC(33:0),PC(O-34:0)		
DG(30:3)_C16:1		
PC(38:9),PC(37:2),PC(O-38:2),PC(P-38:1)		
[TG(57:12),TG(56:5)]_C18:2		
SM(d16:1/22:1)		
PC(36:8),PC(35:1),PC(O-36:1),PC(P-36:0)		
[TG(46:2)]_C18:2		
[TG(57:10),TG(56:3)]_C18:2		
[TG(56:6)]_C22:5		
PC(37:7),PC(P-38:6),PC(36:0),PC(O-37:0)		
SM(d18:1/24:1(15Z))		
SM(d16:1/22:0)		
[TG(54:5)]_C18:1		
[TG(56:8),TG(55:1)]_C16:0		
PC(41:5),PC(P-42:4)		
SM(d18:1/26:1(17Z))		
PC(30:1),PC(O-31:1),PC(P-31:0)		
CAR(20:0)		
PC(42:7),PC(41:0),PC(O-42:0)		
[TG(51:9),TG(50:2)]_C16:0		
FA(22:7)		
PC(33:1),PC(O-34:1),PC(P-34:0)		
SM(d16:0/23:0)		
[TG(50:9),TG(49:2)]_C18:2		
[TG(52:6)]_C18:3		
FA(22:1)		
PE(O-38:9),PE(36:2),PE(O-37:2),PE(P-37:1)		
Cer(d18:0/17:0)		
[TG(55:10),TG(54:3)]_C18:1		
[TG(54:10),TG(53:3)]_C18:2		
PC(37:5),PC(O-38:5),PC(P-38:4)		
[TG(54:9),TG(53:2)]_C16:0		
[TG(42:1)]_C18:1		
[TG(49:7),TG(48:0)]_C16:0		
LPC(18:1),PC(O-18:1),PC(P-18:0)		
DG(41:6)_C16:1		
[TG(49:7),TG(48:0)]_C18:0		
PC(36:6)		
[TG(50:5)]_C18:2		
PC(36:4),PC(O-37:4)		
PC(31:0),PC(O-32:0)		
FA(19:2)		
SM(d18:1/25:0)		
PI(36:1),PI(O-37:1),PI(P-37:0)		
SM(d16:0/22:0)		
[TG(50:3)]_C18:3		
[TG(57:11),TG(56:4)]_C18:2		

[TG(55:8),TG(54:1)]_C20:0		
[TG(44:1)]_C16:0		
PC(42:0)		
[TG(53:10),TG(52:3)]_C20:0		
[TG(53:8)]_C18:2		
[TG(46:2)]_C18:1		
[TG(49:7),TG(48:0)]_C14:0		
SM(d16:1/24:1)		
[TG(56:7)]_C22:5		
PC(31:1),PC(O-32:1),PC(P-32:0)		
[TG(51:8),TG(50:1)]_C18:1		
FA(21:0)		
[TG(51:9),TG(50:2)]_C18:0		
PC(40:6)		
[TG(51:7),TG(50:0)]_C18:0		
PC(24:0)		
PC(42:8),PC(41:1),PC(O-42:1),PC(P-42:0)		
[TG(52:9),TG(51:2)]_C18:1		
[TG(51:7),TG(50:0)]_C14:0		
[TG(55:11),TG(54:4)]_C18:3		
PC(40:8),PC(39:1),PC(O-40:1),PC(P-40:0)		
SM(d18:0/24:1)		
[TG(53:8),TG(52:1)]_C18:0		
[TG(50:7),TG(49:0)]_C18:0		
PC(16:0),PC(O-17:0),LPC(O-18:0)		
[TG(55:9),TG(54:2)]_C18:2		
SM(d16:1/18:0)		
PC(33:3),PC(O-34:3),PC(P-34:2)		
[TG(52:9),TG(51:2)]_C16:0		
PC(37:3),PC(O-38:3),PC(P-38:2)		
[TG(48:2)]_C16:1		
PS(O-29:0)		
[TG(53:9),TG(52:2)]_C16:0		
DG(30:2)_C16:0		
PE(36:3),PE(P-37:2)		
1-O-tricosanoyl-Cer(d18:1/16:0)		
PC(30:2),PC(P-31:1)		
PC(43:6)		
PC(29:2),PC(P-30:1)		
PC(40:9),PC(39:2),PC(O-40:2),PC(P-40:1)		
[TG(51:9),TG(50:2)]_C18:1		
[TG(50:3)]_C16:1		
[TG(53:9),TG(52:2)]_C16:1		
DG(36:6)_C16:0		
[TG(56:7)]_C20:4		
[TG(57:10),TG(56:3)]_C18:1		
[TG(40:0)]_C16:0		
[TG(38:0)]_C14:0		
[TG(55:11),TG(54:4)]_C18:1		
[TG(52:8),TG(51:1)]_C18:0		

Table S8. Comparison of Lipid Corona Profiles Between Nanoparticle Sizes
Female 75% Serum BC Samples

Unique Lipids in 50 nm BC	Unique Lipds in 100 nm BC	Shared Lipids	50 nm Average of Shared Lipids	100 nm Average of Shared Lipids	p-value of Shared Lipids
[TG(54:5)]_C18:0	CE(20:5)Na	CE(20:0) NH4	28968.08229	10275.11079	2.1994E-06
PC(44:10),PC(O-44:3)	DG(32:2)_C18:1	CE(16:0)K	30643.36609	12993.4949	3.66757E-06
[TG(59:13),TG(58:6)]_C18:1	DG(O-38:8),DG(36:1)_C18:1	CE(22:5)H	7124.864513	4119.320962	4.49159E-06
[TG(54:6)]_C18:2	DG(32:1)_C16:0	CE(20:2)Na	7495.820565	4080.93029	7.65153E-06
[TG(57:12),TG(56:5)]_C18:1	[TG(56:11),TG(55:4)]_C18:2	CE(22:6) NH4	24723.33379	13893.51966	0.000252465
[TG(56:12),TG(55:5)]_C18:1	CE(20:2) NH4	CE(22:5) NH4	5945.636429	4397.726325	0.000322136
PC(39:8),PC(O-40:8),PC(38:1),PC(O-39:1),PC(P-39:0)	DG(40:5)_C16:0	CE(20:1) NH4	10322.30076	5056.39371	0.00108865
LPI(20:0)	CE(20:5) NH4	CE(16:1) NH4	19961.96537	13635.20826	0.001979752
[TG(38:0)]_C18:0	CE(22:3)H	DG(O-40:9),DG(38:2)_C18:1	4205.776298	3314.509559	0.002816512
LPC(16:0),PC(O-16:0),LPC(O-17:0)	DG(32:0)_C16:0	CE(18:1) NH4	171280.0919	102064.7337	0.003289496
[TG(48:2)]_C14:0	CE(14:0) NH4	DG(39:8),DG(O-40:8),DG(38:1)_C18:1	150769.7318	91816.03296	0.003432361
[TG(52:4)]_C20:4	DG(39:7),DG(38:0),DG(dO-40:0)_C18:0	DG(O-40:9),DG(38:2)_C18:2	1312427.843	855958.6905	0.004582354
[TG(49:8),TG(48:1)]_C14:0	CE(16:0)Na	CE(18:2) NH4	1444310.22	946414.354	0.004954513
PC(40:1),PC(P-41:0)	DG(37:7)_C16:1	CE(19:0)H	281908.2241	184049.8658	0.007129401
SM(d16:1/24:0)	LPG(19:0),LPG(O-20:0); LPG(19:0),LPG(O-20:0)	PC(34:3),PC(P-35:2)	16106.85325	19550.76678	0.007841883
[TG(54:11),TG(53:4)]_C16:0	DG(36:4),DG(O-37:4)_C18:2	CE(20:2)K	6465.436452	4842.903018	0.008364176
PC(36:7),PC(35:0),PC(O-36:0)	SM(d18:2/20:1)	DG(39:8),DG(O-40:8)_C18:2	49831.67142	33275.26026	0.008649613
[TG(52:6)]_C16:0	CE(17:1) NH4	DG(39:7)_C18:1	8312.468564	5906.698424	0.009013761
SM(d16:0/25:0)	CE(22:2)H	CE(20:4) NH4	227021.7404	158903.0021	0.013410289
Cer(d18:1/24:0)	CE(19:0)K	CE(18:0)K	47762.4673	35303.89323	0.019027129
[TG(56:6)]_C20:4	CE(18:3)K	[TG(55:8),TG(54:1)]_C18:0	9780.020697	6295.790453	0.029935281
[TG(49:8),TG(48:1)]_C16:1	DG(38:5)_C16:0	PC(34:2),PC(O-35:2),PC(P-35:1)	690302.4076	842512.8618	0.032468868
[TG(48:7),TG(47:0)]_C16:0	DG(38:3)_C18:2	SM(d18:2/18:1)	2987.800209	3360.493568	0.040310627
[TG(58:9)]_C22:6	CE(18:2)K	[TG(53:7)]_C18:1	3934.308305	2328.454831	0.053951875
PC(39:6),PC(O-40:6),PC(P-40:5)	DG(34:2)_C16:1	[TG(56:11),TG(55:4)]_C18:1	2138.596164	1278.246094	0.059632597
PI(38:4)	CE(22:2) NH4	[TG(55:9),TG(54:2)]_C18:0	24762.05365	16933.53988	0.060600954
[TG(52:10),TG(51:3)]_C18:1	DG(40:2)_C18:2	[TG(53:8),TG(52:1)]_C18:1	49074.01134	29437.1466	0.065926961
[TG(55:7)]_C18:1	LPE(20:4)	[TG(55:9),TG(54:2)]_C18:1	38575.97084	22748.35958	0.067808468
[TG(48:3)]_C18:2	CE(15:0)K	SM(d18:0/26:1(17Z))	3158.812212	1980.185468	0.095595362
[TG(48:2)]_C16:0	DG(34:4),DG(dO-36:4)_C16:1	[TG(53:8),TG(52:1)]_C18:0	36872.17455	26463.14034	0.105547949
[TG(49:7)]_C16:1	CE(18:1)Na	[TG(53:8)]_C18:2	2604.124171	1677.470783	0.109752579
[TG(56:12),TG(55:5)]_C18:2	CE(19:0) NH4	[TG(57:11),TG(56:4)]_C18:1	4302.388286	2756.341512	0.114664017
SM(d16:1/23:0)	CE(16:2)Na	[TG(53:10),TG(52:3)]_C16:0	185572.1052	119977.6906	0.132792682
[TG(46:1)]_C16:0	CE(22:1)H	[TG(55:10),TG(54:3)]_C18:2	22623.03359	16675.66638	0.140514581
LPC(20:3)	CAR(10:2)	[TG(55:8),TG(54:1)]_C18:1	6363.312438	4745.372327	0.146393267
PC(42:1)	CE(20:5)H	[TG(56:8)]_C22:6	2844.472218	2463.505508	0.163126505
[TG(50:4)]_C16:1	DG(34:2)_C16:0	[TG(53:9),TG(52:2)]_C18:2	30505.73012	22942.70959	0.166143023
LPC(18:0),PC(O-18:0),LPC(O-19:0)	DG(37:7),DG(36:0)_C18:0	[TG(58:8)]_C22:6	1618.924116	1091.974081	0.170187734
[TG(44:0),TG(O-45:0)]_C16:0	CE(20:1)K	[TG(55:10),TG(54:3)]_C18:1	110721.4189	83058.79146	0.172102668
[TG(54:9),TG(53:2)]_C18:0	DG(42:5)_C18:0	[TG(54:8),TG(53:1)]_C18:1	1911.276129	1301.322759	0.17422527
[TG(38:1)]_C18:1	CE(19:0)Na	[TG(55:11),TG(54:4)]_C18:1	97790.35924	65890.33404	0.180289974
PC(32:1),PC(O-33:1),PC(P-33:0)	DG(34:2)_C18:2	PE(38:4)	3835.18026	4260.146937	0.197748559
[TG(53:10),TG(52:3)]_C18:0	DG(34:3)_C16:1	[TG(53:9),TG(52:2)]_C18:0	24045.9219	18364.56793	0.198486412
[TG(39:0)]_C20:0	CE(15:1)K	CAR(10:2)_QUAL	4183.760297	4457.020306	0.207302262
[TG(54:6)]_C18:1	CE(18:3)H	[TG(54:5)]_C18:2	50923.23971	35055.08175	0.214167438
[TG(50:3)]_C14:0	DG(30:0)_C16:0	[TG(57:12),TG(56:5)]_C20:4	3216.452232	2249.988836	0.217416193
[TG(50:4)]_C18:1	DG(36:3)_C18:1	[TG(53:10),TG(52:3)]_C18:1	181774.5729	143497.4338	0.252274827
[TG(49:8),TG(48:1)]_C18:1	CE(18:2)Na	[TG(53:10),TG(52:3)]_C18:2	164927.2672	132375.7217	0.316536546
[TG(49:8)]_C18:2	CE(16:0) NH4	[TG(53:10),TG(52:3)]_C16:1	9814.268693	7958.53193	0.316845138
PC(42:6)	CE(22:1) NH4	[TG(52:4)]_C18:1	19509.53353	15807.11325	0.327120863
PC(34:0),PC(O-35:0)	PC(27:0),PC(O-28:0)	[TG(52:5)]_C16:1	4223.840316	3491.446918	0.342917985
[TG(54:11),TG(53:4)]_C18:1	DG(O-38:9),DG(36:2)_C18:2	[TG(56:7)]_C22:5	2365.716169	1945.616141	0.344761638
SM(d18:0/15:0)	CE(22:4) NH4	PE(34:2),PE(O-35:2),PE(P-35:1)	1764.216134	2254.402826	0.349032269
[TG(53:8),TG(52:1)]_C16:0	DG(34:0)_C16:0	PC(39:7),PC(P-40:6),PC(38:0),PC(O-39:0)	6521.008443	5400.67505	0.37232356
PC(42:2)	DG(37:7),DG(36:0)_C16:0	[TG(52:4)]_C18:2	104946.5993	86750.3674	0.374090919
PC(34:1),PC(O-35:1),PC(P-35:0)	DG(34:2)_C18:1	[TG(56:7),TG(55:0)]_C16:0	2180.936154	1836.612794	0.387238176
SM(d17:1/24:1)	CE(20:5)K	[TG(52:4)]_C16:0	60543.66022	50421.62975	0.406440098
[TG(48:3)]_C16:1	DG(34:0)_C18:0	CE(22:6)H	3372.820226	2756.97019	0.416453426
[TG(52:6)]_C16:1	CE(18:3) NH4	CE(18:3)Na	23460.31366	19019.38006	0.441945585
PC(32:3),PC(P-33:2)	DG(32:0)_C18:0	[TG(56:6)]_C22:5	2536.036172	2157.239488	0.449333875
PC(35:6),PC(P-36:5)	FA(14:2)	[TG(56:6)]_C18:2	2845.696223	2450.882848	0.469235101

SM(d17:1/26:1)	DG(37:6)_C18:0	[TG(52:4)]_C16:1	12791.765	10977.9715	0.475767527
[TG(53:10),TG(52:3)]_C18:3	FA(15:1)	PC(41:7),PC(P-42:6),PC(40:0),PC(O-41:0)	2315.856175	1748.362793	0.478573485
[TG(54:6)]_C18:3	DG(34:1)_C16:0	PS(P-37:0)	1667.180115	1932.763476	0.480377864
[TG(57:12),TG(56:5)]_C22:5	CE(16:1)Na	PS(38:4)	2104.300151	1823.630126	0.481721586
[TG(55:9),TG(54:2)]_C20:0	DG(37:6)_C16:0	DG(40:9),DG(39:2)_C18:2	2554.956181	3203.352895	0.482231544
[TG(52:9),TG(51:2)]_C18:2	DG(O-38:9),DG(36:2)_C18:1	[TG(52:5)]_C18:2	11088.86083	9593.184012	0.511833585
[TG(57:9),TG(56:2)]_C18:0	DG(36:7),DG(35:0)_C18:0	SM(d18:2/14:0)	2789.0122	2291.462162	0.532470098
[TG(50:7),TG(49:0)]_C16:0	DG(O-38:9),DG(36:2)_C18:0	PC(33:1),PC(O-34:1),PC(P-34:0)	11091.75271	9224.952599	0.572343783
[TG(44:0),TG(O-45:0)]_C14:0	CE(18:1)K	PC(33:2),PC(O-34:2),PC(P-34:1)	13909.00101	11683.97747	0.5894419
PE(36:4),PE(O-37:4)	DG(40:5)_C18:0	CE(16:3)Na	4368.756295	3840.079619	0.589501395
PC(O-40:9),PC(38:2),PC(P-39:1)	CE(18:0) NH4	SM(d18:1/19:0)	10701.43673	9013.946705	0.591611074
[TG(51:7)]_C18:1	DG(33:0)_C16:0	PC(39:5),PC(O-40:5),PC(P-40:4)	11827.06486	13221.02164	0.645249978
PC(42:10),PC(41:3),PC(O-42:3),PC(P-42:2)	DG(O-38:8),DG(36:1)_C18:0	PE(34:1),PE(O-35:1),PE(P-35:0)	1936.988143	2153.081486	0.656379345
[TG(44:2)]_C16:0	CE(20:0)Na	PC(36:4),PC(O-37:4)	275881.9429	238082.8946	0.663029189
SM(d16:1/20:0)	CE(22:3) NH4	CE(20:3)Na	2535.096189	2186.606159	0.730467724
[TG(51:9),TG(50:2)]_C14:0	CE(20:4)H	SM(d16:0/23:0)	7294.168533	7837.701877	0.761101295
SM(d16:0/16:0)	DG(35:6)_C18:0	PC(32:2),PC(O-33:2),PC(P-33:1)	16857.73721	17863.69389	0.804384002
PC(37:4),PC(O-38:4),PC(P-38:3)	CE(22:4)K	PE(38:5)	1135.18008	1281.70009	0.810850813
[TG(37:0)]_C18:0	CE(22:4)Na	PE(38:6)	1458.872095	1325.942094	0.811356052
[TG(50:3)]_C18:1	DG(36:3)_C18:2	LPC(20:4)	4043.220307	4374.480296	0.83400188
[TG(52:10),TG(51:3)]_C18:2	CE(20:4)Na	PC(33:3),PC(O-34:3),PC(P-34:2)	10796.54073	10315.11071	0.836350867
[TG(46:1)]_C16:1	DG(35:6)_C16:0	SM(d16:1/22:1)	22752.88165	23820.74163	0.845520328
SM(d16:0/24:0)	DG(34:3)_C18:1	PE(O-38:8),PE(36:1),PE(O-37:1),PE(P-37:0)	1469.264104	1359.977434	0.847908337
[TG(48:3)]_C18:1	DG(34:1)_C18:1	SM(d16:1/22:0)	281472.1322	294123.4595	0.852800238
[TG(58:8)]_C22:5	DG(34:3)_C18:2	DG(O-38:8),DG(36:1)_C16:1	11697.01289	10949.37942	0.861780059
[TG(46:0)]_C14:0		PC(38:6)	75301.01763	77868.12787	0.885581025
[TG(58:7)]_C22:5		LPC(18:2),LPC(P-19:1)	5787.512403	5992.339105	0.923253283
PC(42:11),PC(41:4),PC(O-42:4)		PC(35:2),PC(O-36:2),PC(P-36:1)	19245.24133	18949.33804	0.945455099
[TG(46:1)]_C14:0		CE(20:0)H	2825.600198	2789.330193	0.953338071
SM(d16:1/17:0)		PG(16:0),LPG(17:0),LPG(O-18:0); PG(16:0),LPG(17:0),LPG(O-18:0)	2950.668198	3003.744877	0.970303675
[TG(56:9),TG(55:2)]_C18:1		CAR(14:2)_QUAL	3261.656231	3291.376238	0.971483134
[TG(54:5)]_C22:5		CE(20:3) NH4	17452.29311	17340.33332	0.986084678
Cer(d18:1/16:0)					
[TG(54:7),TG(53:0)]_C18:0					
[TG(53:8),TG(52:1)]_C16:1					
[TG(52:9),TG(51:2)]_C16:1					
[TG(49:6)]_C16:0					
[TG(51:8),TG(50:1)]_C18:0					
[TG(52:8),TG(51:1)]_C18:1					
[TG(57:9),TG(56:2)]_C20:0					
[TG(57:11),TG(56:4)]_C18:0					
[TG(50:4)]_C14:0					
[TG(42:0)]_C16:0					
PC(O-38:8),PC(36:1),PC(O-37:1),PC(P-37:0)					
[TG(55:11),TG(54:4)]_C16:0					
[TG(55:9),TG(54:2)]_C16:0					
[TG(52:5)]_C20:4					
[TG(53:9),TG(52:2)]_C18:1					
[TG(52:7),TG(51:0)]_C16:0					
PC(36:5)					
PC(42:3)					
[TG(53:8),TG(52:1)]_C20:0					
[TG(54:6)]_C16:0					
[TG(56:10),TG(55:3)]_C18:1					
[TG(51:8),TG(50:1)]_C16:0					
[TG(55:11),TG(54:4)]_C20:4					
[TG(57:12),TG(56:5)]_C18:0					
PC(40:10),PC(39:3),PC(O-40:3),PC(P-40:2)					
[TG(49:7)]_C18:1					
[TG(50:4)]_C18:2					
PC(38:8),PC(37:1),PC(O-38:1),PC(P-38:0)					
SM(d18:1/17:0)					
[TG(50:3)]_C16:0					
PC(38:7),PC(37:0),PC(O-38:0)					
[TG(52:5)]_C18:1					

PC(28:0),PC(O-29:0)				
PC(34:6)				
SM(d16:0/20:0)				
[TG(58:7)]_C18:1				
PC(40:4)				
PC(35:4),PC(O-36:4),PC(P-36:3)				
CE(22:6)Na				
[TG(50:8),TG(49:1)]_C16:0				
[TG(51:6)]_C16:0				
PC(40:3)				
PC(33:0),PC(O-34:0)				
[TG(57:12),TG(56:5)]_C18:2				
PC(36:8),PC(35:1),PC(O-36:1),PC(P-36:0)				
SM(d18:1/24:1(15Z))				
[TG(54:5)]_C18:1				
PC(30:1),PC(O-31:1),PC(P-31:0)				
[TG(51:9),TG(50:2)]_C16:0				
[TG(50:9),TG(49:2)]_C18:2				
[TG(55:10),TG(54:3)]_C20:0				
[TG(54:10),TG(53:3)]_C18:2				
PC(37:5),PC(O-38:5),PC(P-38:4)				
[TG(49:7),TG(48:0)]_C16:0				
[TG(49:7),TG(48:0)]_C18:0				
PC(36:6)				
[TG(50:5)]_C18:2				
PC(31:0),PC(O-32:0)				
[TG(44:1)]_C16:0				
[TG(55:8),TG(54:1)]_C20:0				
PC(42:0)				
[TG(46:2)]_C18:1				
PC(31:1),PC(O-32:1),PC(P-32:0)				
[TG(51:8),TG(50:1)]_C18:1				
PC(40:6)				
[TG(51:7),TG(50:0)]_C18:0				
[TG(52:9),TG(51:2)]_C18:1				
[TG(55:11),TG(54:4)]_C18:3				
[TG(55:9),TG(54:2)]_C18:2				
SM(d16:1/18:0)				
[TG(52:9),TG(51:2)]_C16:0				
[TG(48:2)]_C16:1				
[TG(53:9),TG(52:2)]_C16:0				
1-O-tricosanoyl-Cer(d18:1/16:0)				
PC(30:2),PC(P-31:1)				
PC(43:6)				
PC(29:2),PC(P-30:1)				
[TG(50:3)]_C16:1				
[TG(56:7)]_C20:4				
[TG(57:10),TG(56:3)]_C18:1				
[TG(40:0)]_C16:0				
[TG(38:0)]_C14:0				
[TG(52:8),TG(51:1)]_C18:0				
[TG(46:2)]_C16:0				
SM(d18:1/12:0)				
SM(d18:0/17:0)				
PI(38:3)				
PI(36:2),PI(O-37:2),PI(P-37:1)				
Cer(d18:1/22:0)				
[TG(54:11),TG(53:4)]_C18:2				
[TG(53:7),TG(52:0)]_C16:0				
[TG(57:12),TG(56:5)]_C16:0				
[TG(52:4)]_C18:3				
[TG(54:5)]_C18:3				
[TG(53:7),TG(52:0)]_C20:0				
SM(d16:1/16:0)				
[TG(50:9),TG(49:2)]_C16:0				

[TG(44:1)]_C18:1					
PC(39:4),PC(O-40:4),PC(P-40:3)					
[TG(50:9),TG(49:2)]_C18:1					
[TG(51:7),TG(50:0)]_C16:0					
PC(O-38:9),PC(36:2),PC(O-37:2),PC(P-37:1)					
PC(31:2),PC(O-32:2),PC(P-32:1)					
PC(35:5),PC(O-36:5),PC(P-36:4)					
[TG(52:8),TG(51:1)]_C16:0					
[TG(46:0)]_C18:0					
PC(42:5)					
[TG(51:9),TG(50:2)]_C16:1					
[TG(54:10),TG(53:3)]_C16:0					
PC(30:0),PC(O-31:0)					
PC(43:4),PC(O-44:4)					
[TG(46:1)]_C18:1					
[TG(54:10),TG(53:3)]_C18:1					
[TG(52:6)]_C18:2					
[TG(53:7),TG(52:0)]_C18:0					
Cer(d18:1/23:0)					
[TG(52:5)]_C18:3					
[TG(52:5)]_C16:0					
LPC(22:4)					
[TG(49:8),TG(48:1)]_C16:0					
[TG(55:10),TG(54:3)]_C18:0					
CAR(14:1)					
SM(d16:1/25:0)					
[TG(51:8)]_C18:2					
PC(41:6),PC(O-42:6)					
[TG(54:12),TG(53:5)]_C18:2					
[TG(56:8)]_C18:2					
PC(36:3),PC(P-37:2)					
PC(37:6),PC(O-38:6),PC(P-38:5)					
Cer(d18:1/24:1(15Z))					
SM(d17:0/27:0)					
[TG(50:8),TG(49:1)]_C18:1					
[TG(56:8)]_C20:4					
[TG(55:11),TG(54:4)]_C18:2					
[TG(57:8),TG(56:1)]_C20:0					
LPG(20:0); LPG(20:0)					
[TG(46:3)]_C18:1					
[TG(48:3)]_C14:0					
[TG(52:7),TG(51:0)]_C18:0					
[TG(55:7),TG(54:0)]_C20:0					
[TG(56:7)]_C18:2					
SM(d16:0/18:0)					
[TG(51:8),TG(50:1)]_C16:1					
[TG(54:9),TG(53:2)]_C18:1					
[TG(54:7)]_C20:4					
PC(40:5)					
[TG(44:0),TG(O-45:0)]_C18:0					
[TG(57:10),TG(56:3)]_C18:0					
[TG(58:8),TG(57:1)]_C18:1					
PC(44:0)					
[TG(51:8),TG(50:1)]_C14:0					
PC(44:12),PC(O-44:5)					
[TG(56:7)]_C22:6					
[TG(50:4)]_C18:3					
[TG(56:6)]_C16:0					
PC(19:1),LPC(20:1),PC(O-20:1),PC(P-20:0)					
[TG(54:7)]_C18:2					
PC(28:2)					
[TG(54:8),TG(53:1)]_C18:0					
PC(35:3),PC(O-36:3),PC(P-36:2)					
[TG(48:4)]_C18:2					
PC(38:4)					

[TG(57:10),TG(56:3)]_C20:0				
SM(d16:1/20:1)				
[TG(48:2)]_C18:1				
SM(d18:2/24:1)				
[TG(57:9),TG(56:2)]_C18:1				
PC(42:9),PC(41:2),PC(O-42:2),PC(P-42:1)				
[TG(55:11),TG(54:4)]_C16:1				
SM(d18:0/24:0)				
CAR(14:2)				
[TG(54:5)]_C20:4				
PC(40:7),PC(39:0),PC(O-40:0)				
[TG(46:0)]_C16:0				
[TG(55:11),TG(54:4)]_C18:0				
PC(38:5)				
PC(38:3)				
[TG(48:3)]_C16:0				
[TG(50:3)]_C18:2				
SM(d18:2/15:0)				
[TG(54:5)]_C16:1				
[TG(54:5)]_C16:0				
PC(29:0),PC(O-30:0)				
PC(32:0),PC(O-33:0)				
[TG(54:9),TG(53:2)]_C18:2				
[TG(49:8),TG(48:1)]_C18:0				
SM(d16:1/18:1)				
[TG(57:11),TG(56:4)]_C20:0				
LPC(20:2),PC(O-20:2)				
SM(d18:2/22:1)				
[TG(54:6)]_C20:4				
[TG(51:9),TG(50:2)]_C18:2				
[TG(48:2)]_C18:2				
[TG(54:8),TG(53:1)]_C16:0				
[TG(56:6)]_C18:0				
[TG(52:10),TG(51:3)]_C16:0				
[TG(54:7)]_C18:3				
PC(42:4)				
[TG(55:10),TG(54:3)]_C16:0				
PC(29:1),PC(O-30:1),PC(P-30:0)				
SM(d18:2/21:0)				
[TG(51:4)]_C18:2				
[TG(56:8),TG(55:1)]_C18:1				
[TG(50:4)]_C16:0				
[TG(49:3)]_C18:2				
PC(40:2)				
[TG(55:8),TG(54:1)]_C16:0				
[TG(51:6)]_C18:0				
PC(28:1),PC(P-29:0)				
PC(38:9),PC(37:2),PC(O-38:2),PC(P-38:1)				
[TG(46:2)]_C18:2				
[TG(57:10),TG(56:3)]_C18:2				
PC(37:7),PC(P-38:6),PC(36:0),PC(O-37:0)				
[TG(56:8),TG(55:1)]_C16:0				
SM(d18:1/26:1(17Z))				
PC(41:5),PC(P-42:4)				
PC(42:7),PC(41:0),PC(O-42:0)				
FA(18:0)				
[TG(52:6)]_C18:3				
CE(20:1)H				
PE(O-38:9),PE(36:2),PE(O-37:2),PE(P-37:1)				
[TG(54:9),TG(53:2)]_C16:0				
LPC(18:1),PC(O-18:1),PC(P-18:0)				
SM(d18:1/25:0)				
PE(40:6),PE(dO-40:0)				
[TG(50:3)]_C18:3				
SM(d16:0/22:0)				

[TG(57:11),TG(56:4)]_C18:2					
LPE(22:4)					
[TG(49:7),TG(48:0)]_C14:0					
SM(d16:1/24:1)					
[TG(51:9),TG(50:2)]_C18:0					
PC(42:8),PC(41:1),PC(O-42:1),PC(P-42:0)					
PC(24:0)					
SM(d18:0/24:1)					
PC(40:8),PC(39:1),PC(O-40:1),PC(P-40:0)					
[TG(50:7),TG(49:0)]_C18:0					
PC(16:0),PC(O-17:0),LPC(O-18:0)					
PC(37:3),PC(O-38:3),PC(P-38:2)					
PS(O-29:0)					
PE(36:3),PE(P-37:2)					
[TG(51:9),TG(50:2)]_C18:1					
PC(40:9),PC(39:2),PC(O-40:2),PC(P-40:1)					
[TG(53:9),TG(52:2)]_C16:1					
